# Enantioselective Michael Spirocyclization of Palladium Enolates

**DOI:** 10.1021/acscatal.5c02758

**Published:** 2025-07-06

**Authors:** Christian Santiago Strong, Peng-Jui Chen, Sanzhar Bissenali, William A. Goddard III, Brian M. Stoltz

**Affiliations:** † The Warren and Katharine Schlinger Laboratory for Chemistry and Chemical Engineering, Division of Chemistry and Chemical Engineering, 6469 California Institute of Technology, Pasadena, California 91125, United States; ‡ Materials and Process Simulation Center, Beckman Institute, California Institute of Technology, Pasadena, California 91125, United States

**Keywords:** asymmetric catalysis, palladium catalysis, Michael cyclizations, quantum mechanics calculations, spirocyclizations

## Abstract

We report an enantioselective and diastereoselective Michael spirocyclization reaction of tetrasubstituted palladium enolates. This allows for the formation of adjacent all-carbon quaternary and tertiary stereocenters in good yield, dr, and ee. Various subsequent cyclization reactions enable access to a diverse range of tricyclic scaffolds. The mechanism of this transformation and the origins of stereoselectivity are investigated via quantum mechanics calculations.

## Graphical abstract

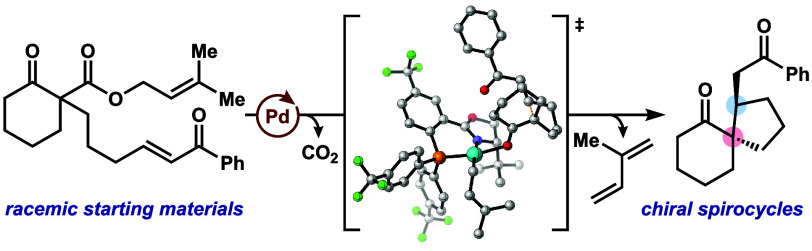

## Introduction

The enantioselective and diastereoselective construction of spirocycles bearing all-carbon quaternary centers remains a persistent challenge in synthetic organic chemistry.^1^ The prevalence of these motifs in complex natural products and other medicinally relevant molecules necessitates the development of new stereoselective reactions that can form these ring systems effectively.^2^ The enantioselective Pd-catalyzed decarboxylative allylic alkylation reaction has proven to be a valuable tool for the construction of sterically congested all-carbon quaternary centers from racemic or prochiral starting materials.^3,4^ Following extensive experimental and computational mechanistic studies, we have become interested in employing the chiral *O*-bound Pd enolate implicated in these transformations in the context of other reactions beyond allylic alkylation.^5^

Recently, our group reported a Pd-catalyzed decarboxylative [4 + 2] cycloaddition of Pd dienolates with tethered dienophiles (**TS1**), resulting in the formation of tricyclic scaffolds in high yields and good enantioselectivities and diastereoselectivities (Figure 1A).^6^ In 1989, Tsuji and co-workers reported a nonenantioselective Pd-catalyzed Michael spirocyclization reaction which, depending on conditions, could favor formation of either protonolysis product **5** or allylated product **6** (Figure 1B).^7^ The diastereomeric outcome, mechanism of catalyst turnover, and broader scope beyond a 5/5 spirane were not discussed.

**Figure 1:**
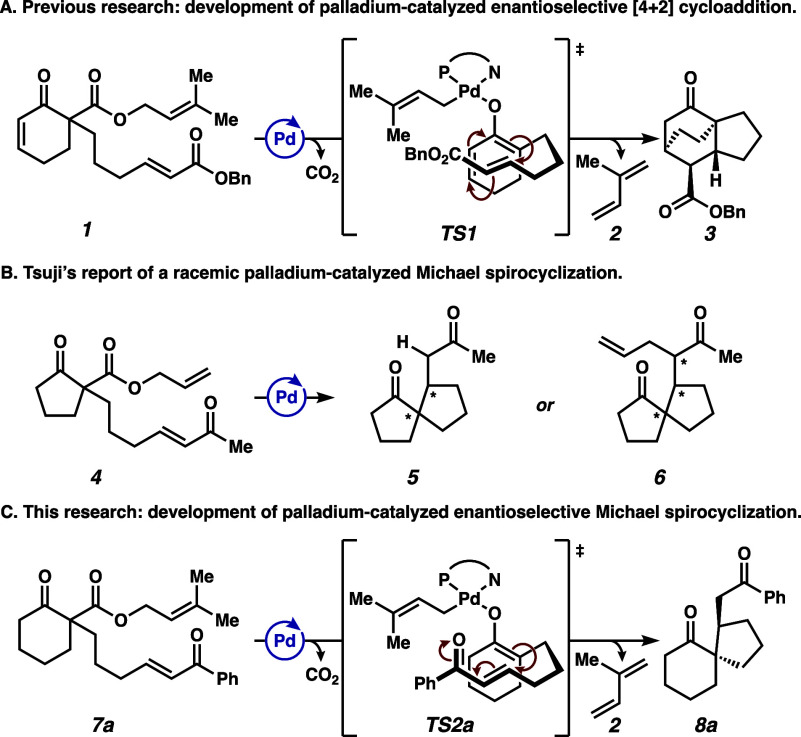
(A) Pd-catalyzed enantioselective [4 + 2] cycloaddition. (B) Racemic Pd-catalyzed Michael spirocyclization reported by Tsuji et al. (C) Development of a new Pd-catalyzed enantioselective and diastereoselective Michael spirocyclization.

Inspired by these findings, we sought to develop an enantioselective and diastereoselective spirocyclization that could efficiently forge vicinal quaternary and tertiary stereocenters (Figure 1C). We envisioned that enantioselectivity could be induced in a similar manner to the previously studied [4 + 2] cycloaddition.^5,6^ On the other hand, the development of Michael cyclizations can be challenging due to the various diastereoselectivity considerations, in contrast to the well-established endo rule of Diels–Alder reactions.^8,9^ For this reason, generating stereoenriched spiranes through a Pd-catalyzed Michael cyclization via an unstabilized enolate would represent a significant advance, complementary to other approaches.^10^

## Results and Discussion

Designing the reaction substrate, we proposed applying our previously developed prenyl β-ketoester technology to achieve additive-free catalyst turnover.^6^ Consequently, we synthesized the corresponding racemic substrate **7a** with a tethered phenyl enone as a Michael acceptor to evaluate the reaction conditions. We found that performing the reaction at 0.025 mmol scale with Pd_2_(dba)_3_ (2.5 mol %) and *(S)*-(CF_3_)_3_-*t*-BuPHOX ligand (6.5 mol %) in toluene (0.1 M) at 40 °C for 16 h is optimal, affording **8a** in 89% ^1^H NMR yield, 93% ee, and >20:1 dr (Table 1, entry 1). The reaction proceeds similarly in THF albeit with a slightly diminished yield and selectivity (Table 1, entry 2). At 21 °C, the conversion is reduced, although the enantioselectivity is marginally higher (Table 1, entry 3).^11^ Raising reaction temperature to 60 °C proves detrimental to both yield and selectivity (Table 1, entry 4). Employing the more electron-rich *(S)*-*t*-BuPHOX results in a similar selectivity but lower yield (Table 1, entry 5). Further dilution of the reaction is inconsequential (Table 1, entry 6). When the reaction is performed at a 0.1 mmol scale, the isolated yield and ee are comparable to what is observed on smaller scale (Table 1, entry 7). The absolute configuration of **8a** was assigned via an X-ray crystal structure (XRD; see Figure 2A).

**Figure 2:**
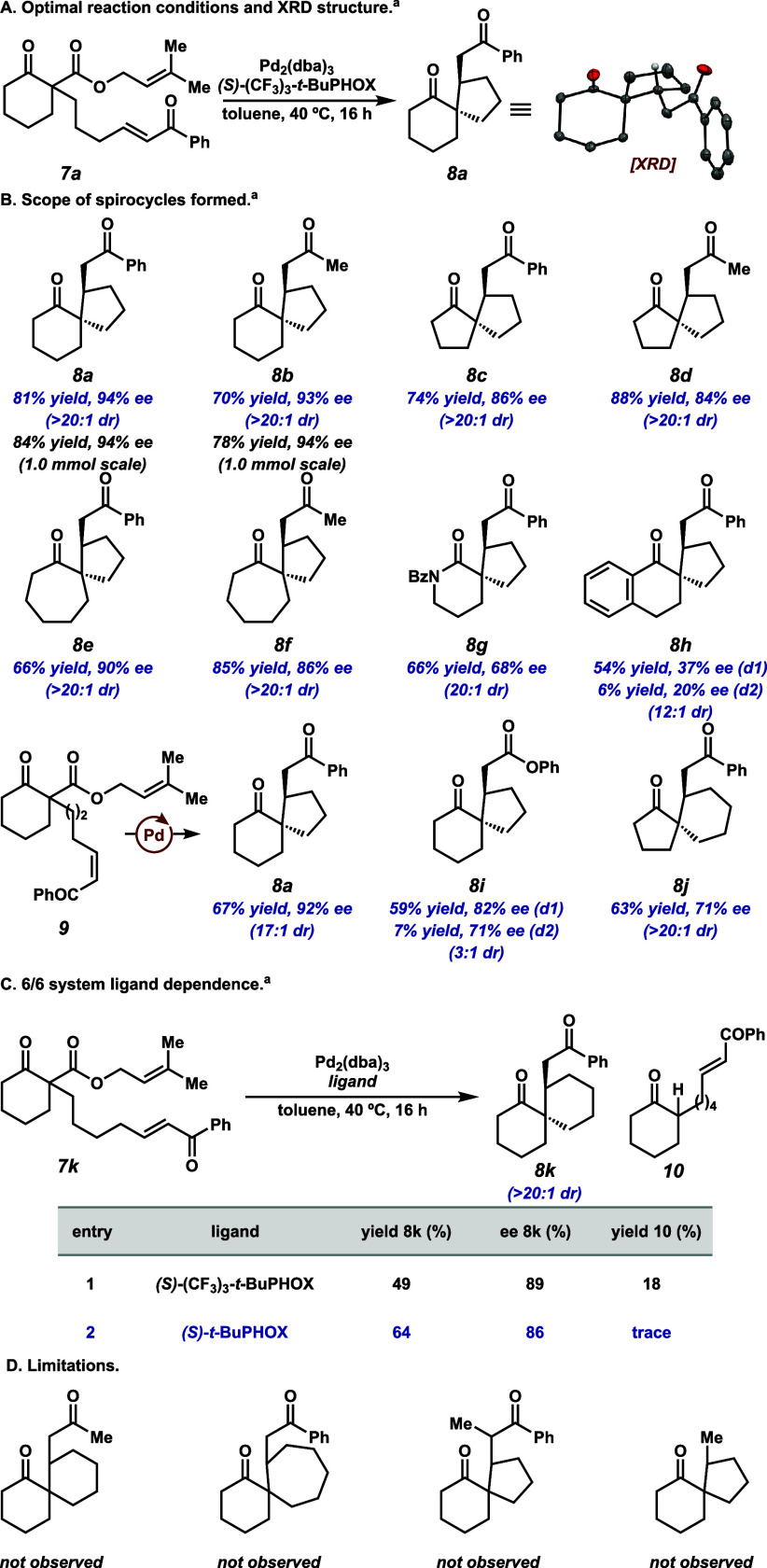
(A) Optimal reaction conditions and XRD structure. (B) Scope of spirocycles formed via Michael spirocyclization reaction. (C) Ligand-dependent product distribution for 6/6 system. (D) Limitations of this transformation. ^*a*^Conditions: 0.1 mmol **7**, 2.5 mol % Pd_2_(dba)_3_, 6.5 mol % ligand, in 0.25 mL of toluene (0.1 M). Isolated yields reported followed by dr’s based on NMR ratios of unpurified reaction mixtures.

**Table 1:**
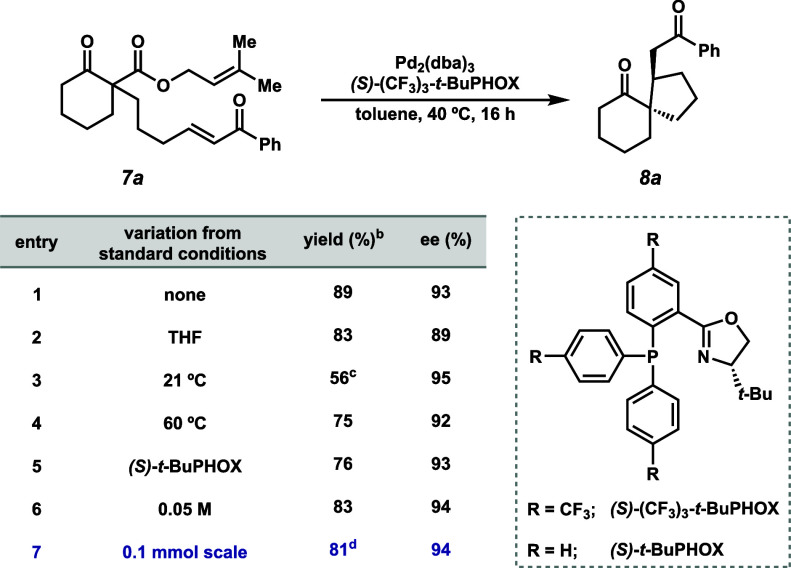
Optimization of [4 + 2] Reaction Conditions^a^

a Conditions: 0.025 mmol **7a**, 2.5 mol % Pd_2_(dba)_3_, 6.5 mol % ligand, in 0.25 mL of solvent (0.1 M). *^b^* Yields determined by ^1^H NMR with respect to 1,3,5-trimethoxybenzene as internal standard.^12^ *^c^* Incomplete conversion was observed.^11^ *^d^* Isolated yield on 0.1 mmol scale.

With the optimal conditions in hand, we began exploring the generality of this transformation (Figure 2B). The phenyl enone can be replaced with a methyl enone to form spirocycle **8b** in a good yield and similar selectivity. Gratifyingly, reactions of both substrates can be scaled to 1.0 mmol, resulting in slightly improved yields with maintained selectivities. 5/5 spirocycles can be accessed (**8c** and **8d**), albeit with slightly lowered enantioselectivities. In addition, 7/5 systems can also be formed (**8e** and **8f**). Spirolactam **8g** is formed in modest yield and lowered enantioselectivity, and tetralone-derived spirocycles are generated in slightly diminished dr and low ee (**8h d1** and **8h d2**, 12:1 dr based on crude NMR).^13^ When *Z*-enone **9** is employed, the same diastereomer of product (**8a**) is obtained in modest yield with similar selectivities.^14^ Employing a phenyl enoate, diastereoselectivity is greatly eroded (**8i d1** and **8i d2**, 3:1 dr based on crude NMR),^13^ while moderate levels of enantioselectivity are maintained. The 5/6 system (derived from a cyclopentanone starting material) forms in a modestly lower yield and ee (**8j**). Interestingly, when employing the optimal ligand toward the 6/6 system (Figure 2C, entry 1, **7k → 8k**), some undesired protonation of the initial Pd-enolate occurs, generating **10**. This can be suppressed, however, by employing the more electron-rich *(S)*-*t*-BuPHOX ligand (Figure 2C, entry 2). There were several systems investigated in which appreciable amounts of spirocycles are not clearly observed (Figure 2D). In these cases, direct protonation of the initial Pd-enolate is observed as the major product.

Inspired by Tsuji’s enone difunctionalization (**6**, Figure 1B), we opted to investigate whether it would be possible to selectively form a stereotriad by employing a cinnamyl β-ketoester (**11a**, Figure 3A).^7^ We found that employing the same catalytic conditions with **11a** resulted in formation of the desired difunctionalized spirocycle **12a**, isolated as a single diastereomer in 44% yield.^15^ The absolute configuration was confirmed via X-ray crystallography (XRD; see Figure 3A). Additionally, the corresponding 6/6 spirane (**12b**) was also formed under the same conditions. Through this cascade transformation, racemic cinnamyl β-ketoesters were transformed to spirocycles bearing vicinal stereotriads. Further studies of these systems are currently underway.

**Figure 3:**
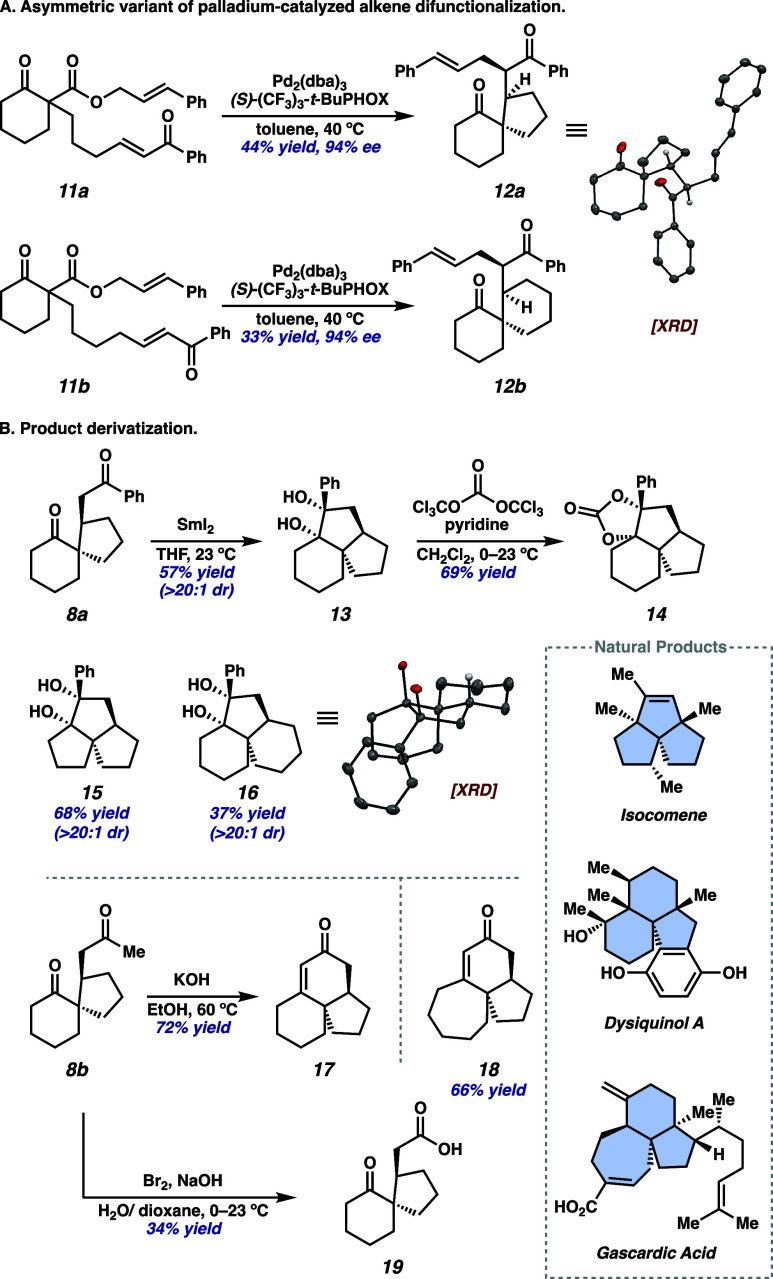
(A) Application of this reaction system to asymmetric enone difunctionalization. (B) Product derivatizations toward versatile functional groups and diverse tricycles.

With a diverse range of Michael adducts bearing dicarbonyls in hand, we sought to explore the utility of these scaffolds (Figure 3B). Treatment of spirane **8a** with stoichiometric samarium diiodide generates pinacol product **13**.^16^ The *cis* diol is formed in this process, as suggested by the successful formation of cyclic carbonate **14** upon the subjection of **13** to triphosgene and pyridine. In addition to the 6/5/5 system (**13**), 5/5/5 and 6/5/6 angular ring systems (**15** and **16**) are also accessible using this method with the latter’s structure (**16**) confirmed via XRD. These ring systems bear four contiguous stereocenters, three of which are fully substituted. Additionally, aldol condensation was performed on methyl ketone **8b**, resulting in tricyclic enone **17**. This allows for differentiation of the carbonyls and access to the corresponding 6/6/5 (**17**) and 7/6/5 (**18**) angular ring systems. Lastly, methyl ketone **8b** can be converted to the corresponding carboxylic acid **19** through a haloform reaction, providing two orthogonal functionalities. These carbocyclic ring systems are found in numerous natural products such as isocomene, dysiquinol A, gascardic acid, and others.^2,17^

Interested in the utility of this Michael spirocyclization, we sought to further understand the mechanism through which it proceeds by proposing a potential catalytic cycle. Using previous studies^4–6^ as a starting point, we propose that the mechanism begins with oxidative addition to prenyl β-ketoester **20** to generate Pd-carboxylate **21** (Figure 4). Subsequent decarboxylation generates *O*-bound Pd enolate **22**, which can undergo intramolecular Michael spirocyclization to provide enolate **23a**. Following this, isomerization might occur to form *O*-bound Pd enolate **24** before a proton transfer liberates product **8a** and concomitantly generates isoprene bound to Pd^0^ (**25**). Upon ligand exchange with another equivalent of substrate (**7a**), catalyst turnover can be achieved.

**Figure 4:**
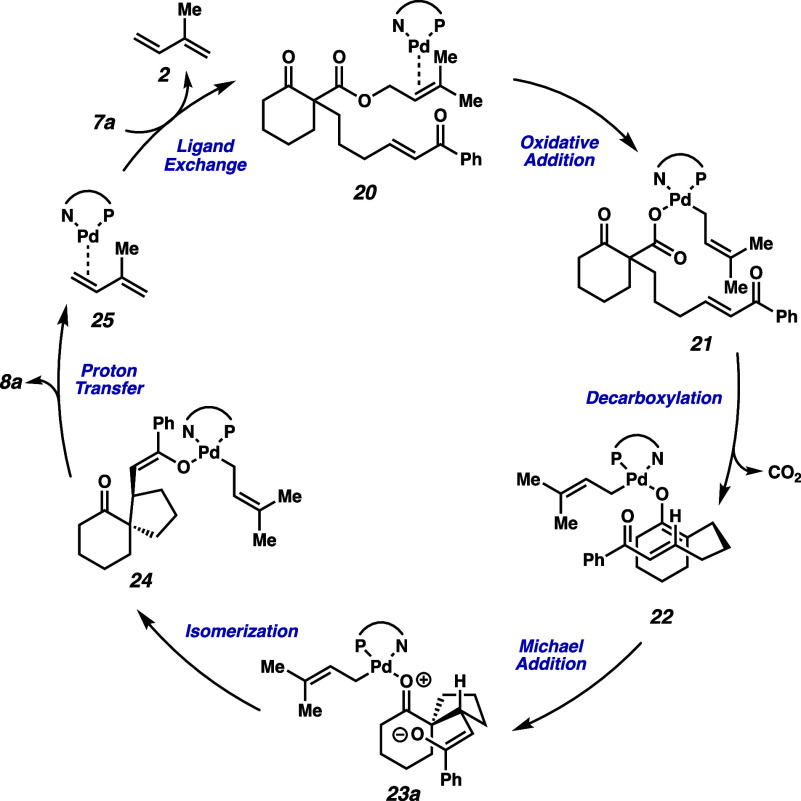
Proposed catalytic cycle.

Intrigued by the Michael spirocyclization proposed in this cycle and the influence of our chiral ligand on this process, we opted to investigate potential Michael addition transition states leading to four possible stereoisomers **23a**–**23d** through DFT calculations (Figure 5). Computations were performed at the PBE0-D4/def2-TZVPP (Pd), ma-def2-TZVPP (O), def2-TZVPP/CPCM­(PhMe)//PBE0-D4/def2-TZVP (Pd), ma-def2-SVP (O), def2-SVP level of theory, as it has proven to be a robust method in our prior studies of similar Pd systems^4^ (see the for more details on computational methods and additional preliminary results).

**Figure 5:**
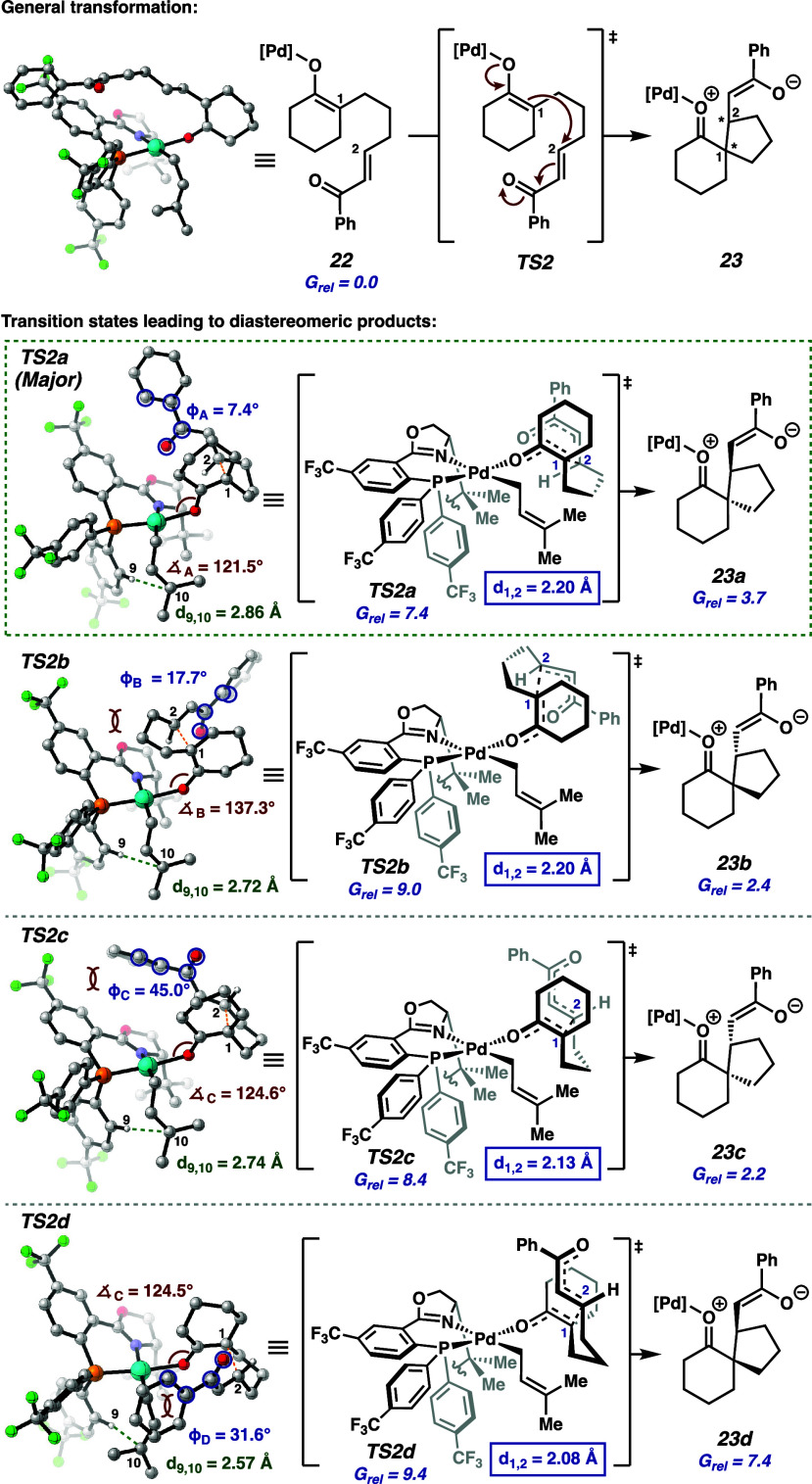
Computational evaluation of the four possible Michael addition transition states leading to the formation of stereoisomers at the PBE0-D4/def2-TZVPP (Pd), ma-def2-TZVPP (O), def2-TZVPP/CPCM­(PhMe)//PBE0-D4/def2-TZVP (Pd), ma-def2-SVP (O), def2-SVP level of theory.

Notably, the minimum energy transition structure to each product features an *s*-cis geometry of the Michael acceptor, resulting in the *Z*-enolate geometries of intermediates **23a**–**23d**. The lowest energy transition state **TS2a** features *syn* alignment of the Michael acceptor tether, relative to the *t*-Bu substituent of the PHOX ligand, and the electrophile approaches from the more sterically accessible *external* face of the enolate. These characteristics are consistent with observations in our previously studied allylic alkylation and Diels–Alder transformations.^4,6^ In comparison, **TS2b** requires the dienophile tether be placed *anti* to the *t*-Bu group of the PHOX ligand to prevent steric clashing that would result from the *internal* electrophile approach (see the for more details). As such, interaction of the tether and the ligand backbone in **TS2b** leads to a distortion of 15.8° at the Pd–O–C bond angle, relative to **TS2a**, reflecting an increased level of steric congestion that contributes to an increased energy barrier of 9.0 kcal/mol (ΔΔ*G* = 1.6 kcal/mol).

Steric interactions between the phenyl ketone of the Michael acceptor and the ligand backbone in **TS2c** lead to a distorted dihedral angle (ϕ_c_) of 45° between the planes of the carbonyl and the phenyl ring. The *internal* electrophile approach in **TS2d** results in a steric clash with the Pd complex, which is reflected by the shortened interatomic distance (*d*_9,10_ = 2.57 Å) between the ligand aryl group and the prenyl moiety. These undesired interactions contribute to the increased 8.4 and 9.4 kcal/mol energy barriers in **TS2c** and **TS2d**, respectively. These computational studies predict **23a** to be the major stereoisomer, which aligns with the experimentally observed product. The calculated 1.6 kcal/mol preference for **TS2a** over **TS2b** is in good agreement with the experimentally observed 94% ee (which corresponds to a 2.1 kcal/mol difference in energy barriers). Further computational and experimental investigations related to the mechanism of catalyst turnover and the reversibility of the Michael addition are currently underway and will be reported in due course.

## Conclusions

In summary, we report an asymmetric Pd-catalyzed Michael spirocyclization reaction that allows for the efficient construction of vicinal quaternary and tertiary stereodiads. This reaction provides access to a broad scope of bicyclic scaffolds in good yield, dr, and ee. The diversification of these products toward complex tricycles relevant to natural product synthesis is also disclosed. Furthermore, our mechanistic understanding allowed for the development of a cinnamyl-based cascade reaction that generates a third stereocenter. We evaluated a stereochemical model of the Michael addition transition states, which suggest that the stereoselectivity of C–C bond formation could be governed by the Michael acceptor’s conformation, placement of the Michael acceptor tether, relative to the *t*-Bu substituent of the PHOX ligand, and the bias toward the *external* versus *internal* of the electrophile.

By applying the rational design framework previously established,^5,6^ we were able to expand the utility of Pd enolates through their application in a highly stereoselective Michael spirocyclization. We envision that this discovery will expedite the synthesis of stereochemically complex and medicinally relevant polycyclic scaffolds. The insights gained from this study broaden our knowledge regarding the stereochemical interactions of different enolate substrates with the Pd–PHOX catalyst system, which will be leveraged to accelerate the future development of synthetically useful asymmetric transformations.

## Supplementary Material















## Notes

Cartesian coordinates of the calculated structures (ZIP)
X-ray data for CCDC 2421482 (CIF)
X-ray data for CCDC 2421483 (CIF)
X-ray data for CCDC 2444207 (CIF)
X-ray data for CCDC 2444208 (CIF)
Additional experimental and computational details, characterization of new compounds (PDF)
QM energies and analysis (XLSX)

## Notes

CCDC Nos. 2421482, 2421483, 2444207, and 2444208 contain the supplementary data for this paper. These data can be obtained free of charge via http://www.ccdc.cam.ac.uk/data_request/cif, or by emailing data_request@ccdc.cam.ac.uk, or by contacting The Cambridge Crystallographic Data Centre, 12 Union Road, Cambridge CB2 1EZ, UK; fax: + 44 1223 336033.
