# Supplementary material for: Enantioselective Michael Spirocyclization of Palladium Enolates
Source: ACS Catal. 2025 Jul 6;15(14):12231–7. doi: 10.1021/acscatal.5c02758 (PMC12854706; doi:10.1021/acscatal.5c02758)
Supplement: Supplementary file 6 [file cs5c02758_si_006.pdf]

Supporting Information for:
Enantioselective Michael Spirocyclization of Palladium Enolates.

Christian Santiago Strong[a], Peng-Jui Chen[a], Sanzhar Bissenali[a], William A. Goddard III[b]*, and Brian M. Stoltz[a]*

[a] *The Warren and Katharine Schlinger Laboratory for Chemistry and Chemical Engineering, Division of Chemistry and Chemical Engineering, California Institute of Technology, Pasadena, California 91125, United States*

[b] *Materials and Process Simulation Center, Beckman Institute, California Institute of Technology, Pasadena, California 91125, United States*

stoltz@caltech.edu
wag@caltech.edu

Table of Contents:

Materials and Methods: 2
Instrumentation and Analysis: 2
List of Abbreviations: 2
Pd-Catalyzed Decarboxylative Michael Cyclization: 3
Protonated Byproducts: 22
Pd-Catalyzed Decarboxylative Alkene Difunctionalization: 25
Preparation of Enone Starting Materials: 28
Preparation of Aldehyde Precursors: 39
Product Derivatizations: 46
General Notes – Quantum Mechanics Calculations: 51
Comparison of Michael Addition Transition State Barriers: 52
Computed Energies for Intermediates Post Michael Addition: 53
Investigation of the Mechansim of Proton Transfer: 53
Preliminary Computational Investigation of the Thermodynamics of the Reaction Post Decarboxylation: 54
2D NMR Analysis of Select Compounds: 55
Preliminary Experimental Mechanistic Investigations: 61
References: 65
NMR and IR Spectra of New Compounds: 68
X-ray Crystal Structure Data: 180

Materials and Methods:
Unless otherwise stated, reactions were performed in flame-dried glassware under a nitrogen atmosphere using dry, deoxygenated solvents. Solvents were dried by passage through an activated alumina column under argon. Reaction progress was monitored by thin-layer chromatography (TLC) or Agilent 1290 UHPLC-MS. TLC was performed using E. Merck silica gel 60 F254 precoated glass plates (0.25 mm) and visualized by UV fluorescence quenching, $KMnO_4$ staining, or *p*-anisaldehyde staining. Silicycle Silia*Flash*® P60 Academic Silica gel (particle size 40–63 nm) was used for flash chromatography. The absolute configurations of **8a**, **8b**, **12a** and **16** were determined by X-ray crystallography, and all other products were assigned by analogy. Reagents were purchased from commercial sources and used as received unless otherwise stated. Ligands were prepared according to literature procedures.[1] HG-II was graciously provided by Umicore.
Instrumentation and Analysis:
^{1}H NMR spectra were recorded on Varian Inova 600 MHz and Bruker 400 MHz spectrometers and are reported relative to residual non-deuterated solvent $CHCl_3$ (δ 7.26 ppm) or C_6H_6 (δ 7.16 ppm). ^{13}C NMR spectra were recorded on a Bruker 400 MHz spectrometer (100 MHz) and are reported relative to solvent $CDCl_3$ (δ 77.16 ppm) or C_6D_6 (δ 128.06 ppm). Data for ^{1}H NMR are reported as follows: chemical shift (δ ppm) (multiplicity, coupling constant (Hz), integration). Multiplicities are reported as the peaks appear as follows: s = singlet, d = doublet, t = triplet, q = quartet, p = pentet, sept = septuplet, m = multiplet, br s = broad singlet, br d = broad doublet. Some reported spectra include minor impurities of water (δ 1.56 ppm), ethyl acetate (δ 4.12, 2.05, 1.26 ppm), methylene chloride (δ 5.30 ppm), acetone (δ 2.17 ppm), grease (δ 1.26, 0.86 ppm), and/or silicon grease (δ 0.07 ppm), which do not impact product assignments. Data for ^{13}C NMR are reported in terms of chemical shifts (δ ppm). IR spectra were obtained by use of a Perkin Elmer Spectrum BXII spectrometer or Nicolet 6700 FTIR spectrometer using thin films deposited on NaCl plates and reported in frequency of absorption (cm^{-1}). Optical rotations were measured with a Jasco P-2000 polarimeter operating on the sodium D-line (589 nm), using a 100 mm path-length cell. Analytical SFC and SFC-MS studies were performed with an Agilent 1260 Infinity II G6125B SFC-MS supercritical CO_2 analytical chromatography system utilizing Chiralpak (IC-3, AD-3, ID-3, IF-3, IG-3, IH-3) or Chiralcel (OD-3, OJ-3) columns (4.6 mm x 25 cm) obtained from Daicel Chemical Industries, Ltd.[2] High resolution mass spectra (HRMS) were obtained from the Caltech Mass Spectral Facility using a JEOL JMS-T2000GC AccuTOFTM GC-Alpha High Resolution Mass Spectrometer in field ionization (FI+) or field desorption (FD+) mode, or an Agilent 6230 Series TOF with an Agilent Jet Stream ion source in electrospray ionization (ESI+). Low-temperature diffraction data (ϕ-and ω-scans) were collected on a Bruker AXS D8 VENTURE KAPPA diffractometer coupled to a PHOTON II CPAD detector with Cu K_α radiation (λ = 1.54178 Å) from an IμS micro-source for the structure of compound V25038. The structure was solved by direct methods using SHELXS[3] and refined against F^2 on all data by full-matrix least squares with SHELXL-2019[4] using established refinement techniques.[5] All non-hydrogen atoms were refined anisotropically. All hydrogen atoms were included into the model at geometrically calculated positions and refined using a riding model. The isotropic displacement parameters of all hydrogen atoms were fixed to 1.2 times the U value of the atoms they are linked to (1.5 times for methyl groups).
List of Abbreviations:
ee – enantiomeric excess, dr – diastereomeric ratio, SFC – supercritical fluid chromatography, HPLC – high-performance liquid chromatography, TLC – thin-layer chromatography, IPA – isopropanol, EtOAc – ethyl acetate.

Pd-Catalyzed Decarboxylative Michael Cyclization:

General Procedure A: Asymmetric Pd-Catalyzed Decarboxylative Michael Cyclization

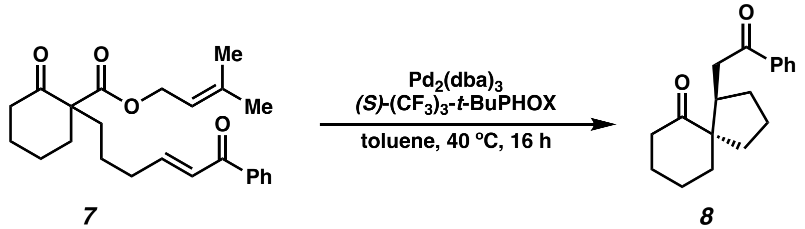

7 *8*

In a nitrogen filled glovebox, an oven-dried 4 mL vial was charged with a stir bar, $Pd_2(dba)_3$ (2.3 mg, 0.0025 mmol, 2.5 mol %), *(S)*-$(CF_3)_3$-*t*-BuPHOX (3.8 mg, 0.0065 mmol, 6.5 mol %), and toluene (0.6 mL). The catalyst solution was stirred at 23 ºC for 20 min. A solution of substrate **7** (0.1 mmol, 1 equiv) in toluene (0.4 mL) was added to the catalyst solution. The resulting mixture was sealed, pumped out of the glovebox, and then heated to 40 ºC for 16 h. The solution was then cooled to 23 ºC and concentrated under reduced pressure. The crude mixture was purified by silica gel flash column chromatography to yield **8**.

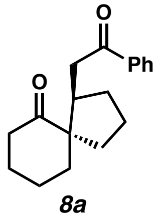

8a

(1*R*,5*S*)-1-(2-oxo-2-phenylethyl)spiro[4.5]decan-6-one (8a)

Prepared from **7a** following General Procedure A. Purification by flash column chromatography (0–60% Et_2O/hexanes) afforded the title compound as a white crystalline solid (21.9 mg, 0.081 mmol, 81% yield, 94% ee). Absolute and relative stereochemistry were assigned by X-ray crystallography (vide infra).

^{1}H NMR (600 MHz, $CDCl_3$): δ 8.12 – 8.07 (m, 2H), 7.58 – 7.53 (m, 1H), 7.51 – 7.45 (m, 2H), 3.21 (dd, J = 14.0, 3.6 Hz, 1H), 3.01 – 2.92 (m, 1H), 2.52 (dd, J = 13.9, 11.2 Hz, 1H), 2.47 – 2.38 (m, 2H), 2.07 – 2.00 (m, 1H), 1.99 – 1.92 (m, 1H), 1.89 – 1.82 (m, 1H), 1.82 – 1.55 (m, 8H), 1.38 – 1.30 (m, 1H).

^{13}C NMR (100 MHz, $CDCl_3$): δ 214.3, 200.8, 136.9, 133.1, 128.71, 128.69, 58.8, 40.5, 40.2, 39.9, 34.9, 31.5, 29.3, 27.3, 21.8, 21.7.

IR (Neat Film, NaCl): 2938, 2862, 1699, 1684, 1448, 1279, 1213, 1128 cm^{-1}.

HRMS (ESI+): *m/z* calc'd for $C_{18}H_{23}O_2$ $[M+H]^+$: 271.1693, found 271.1681.

Optical Rotation: $[\alpha]_D^{23}$ –23.9 (c = 0.69, $CHCl_3$).

SFC conditions: 20% IPA, 2.5 mL/min, Chiralpak IC-3 column, λ = 254 nm, t_R (min): minor = 6.99, major = 8.67

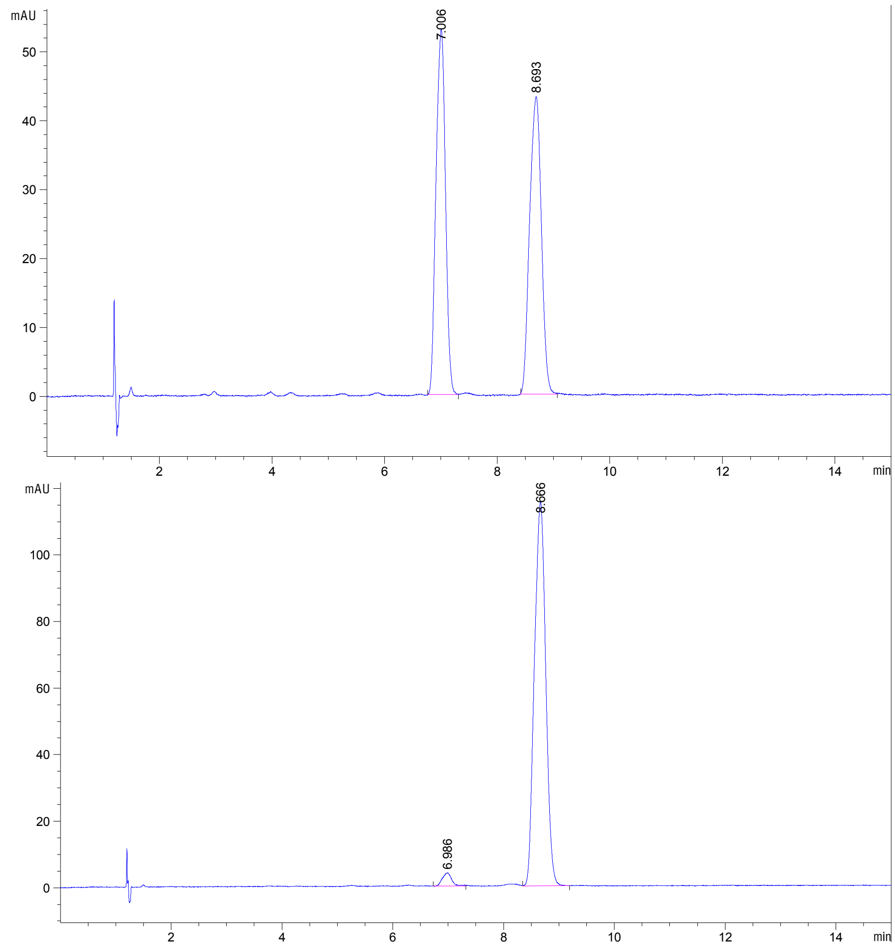

Peak #	RetTime [min]	Type	Width [min]	Area [mAU*s]	Height [mAU]	Area %
1	6.986	BB	0.1773	46.46515	3.94907	2.7559
2	8.666	VB	0.2341	1639.56604	115.17040	97.2441

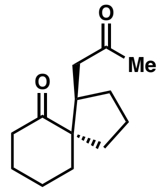

8b

(1*R*,5*S*)-1-(2-oxopropyl)spiro[4.5]decan-6-one (8b)

Prepared from **7b** following General Procedure A. Purification by flash column chromatography (0–60% Et_2O/hexanes) afforded the title compound as a pale yellow crystalline solid (14.5 mg, 0.070 mmol, 70% yield, 93% ee). Absolute and relative stereochemistry were assigned by X-ray crystallography (vide infra).

^{1}H NMR (400 MHz, $CDCl_3$): δ 2.95 – 2.79 (m, 1H), 2.49 (dd, *J* = 14.9, 4.2 Hz, 1H), 2.44 – 2.31 (m, 2H), 2.21 – 2.10 (m, 4H), 2.04 – 1.95 (m, 1H), 1.94 – 1.81 (m, 2H), 1.80 – 1.71 (m, 2H), 1.71 – 1.45 (m, 6H), 1.27 – 1.14 (m, 1H).

^{13}C NMR (100 MHz, $CDCl_3$): δ 214.0, 209.2, 58.3, 45.0, 40.3, 38.8, 35.1, 31.5, 30.0, 29.7, 27.1, 21.9, 21.7.

IR (Neat Film, NaCl): 2941, 2864, 1701, 1447, 1355, 1164, 1129 cm^{-1}.

HRMS (ESI+): *m/z* calc'd for $C_{13}H_{21}O_2$ $[M+H]^+$: 209.1536, found 209.1526.

Optical Rotation: $[\alpha]_D^{23}$ –5.3 (c = 0.49, $CHCl_3$).

SFC conditions: 30% IPA, 2.5 mL/min, Chiralpak IC-3 column, MSD1 TIC[2], t_R (min): major = 3.39, minor = 3.98

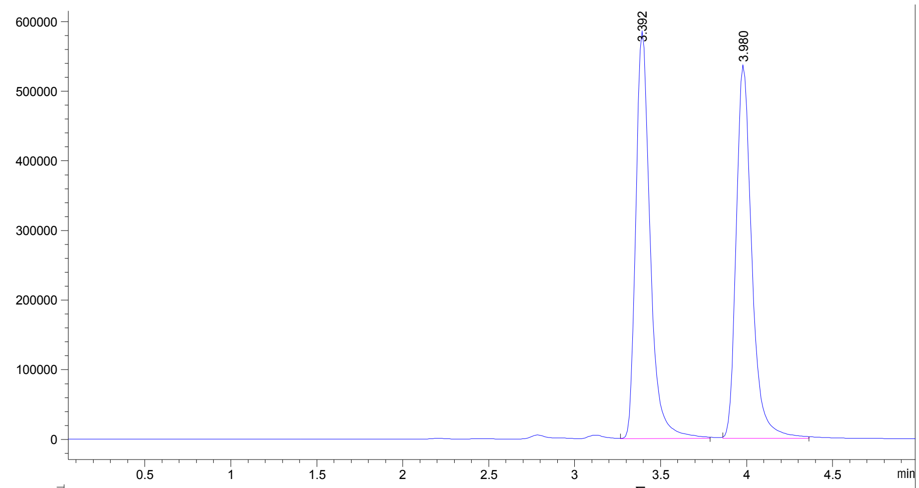

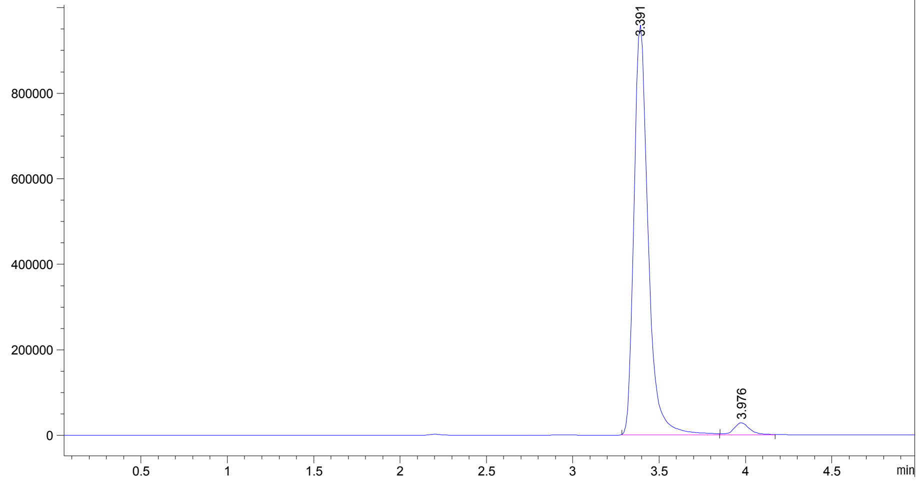

Peak #	RetTime [min]	Type	Width [min]	Area	Height	Area %
1	3.391	BB	0.0863	5.51818e6	9.59211e5	96.7329
2	3.976	BB	0.0993	1.86372e5	2.82916e4	3.2671

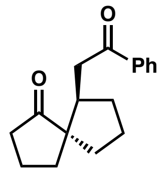

8c

(5*R*,6*R*)-6-(2-oxo-2-phenylethyl)spiro[4.4]nonan-1-one (8c)
Prepared from **7c** following General Procedure A. Purification by flash column chromatography (0–60% Et_2O/hexanes) afforded the title compound as a colorless oil (18.9 mg, 0.074 mmol, 74% yield, 86% ee).

^{1}H NMR (400 MHz, $CDCl_3$): δ 7.96 – 7.88 (m, 2H), 7.58 – 7.51 (m, 1H), 7.49 – 7.42 (m, 2H), 2.98 – 2.83 (m, 2H), 2.82 – 2.69 (m, 1H), 2.42 – 2.30 (m, 1H), 2.29 – 2.15 (m, 1H), 2.10 – 2.01 (m, 1H), 2.00 – 1.92 (m, 1H), 1.92 – 1.78 (m, 2H), 1.77 – 1.65 (m, 4H), 1.63 – 1.51 (m, 1H), 1.41 – 1.29 (m, 1H).

^{13}C NMR (100 MHz, $CDCl_3$): δ 222.9, 199.8, 136.9, 133.2, 128.8, 128.3, 58.8, 41.2, 40.5, 38.0, 37.8, 32.2, 31.5, 23.1, 19.6.

IR (Neat Film, NaCl): 2955, 2870, 1729, 1683, 1596, 1448, 1213, 1157 cm^{-1}.

HRMS (ESI+): *m/z* calc'd for $C_{17}H_{20}O_2Na$ $[M+Na]^+$: 279.1356, found 279.1345.

Optical Rotation: $[\alpha]_D^{23}$ +44.9 (c = 0.74, $CHCl_3$).

SFC conditions: 20% IPA, 2.5 mL/min, Chiralpak IC-3 column, λ = 254 nm, t_R (min): minor = 7.78, major = 10.03

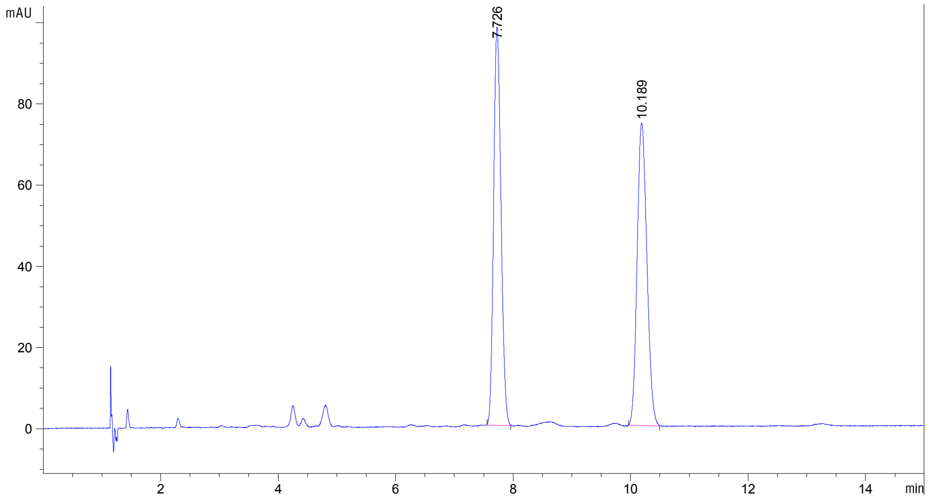

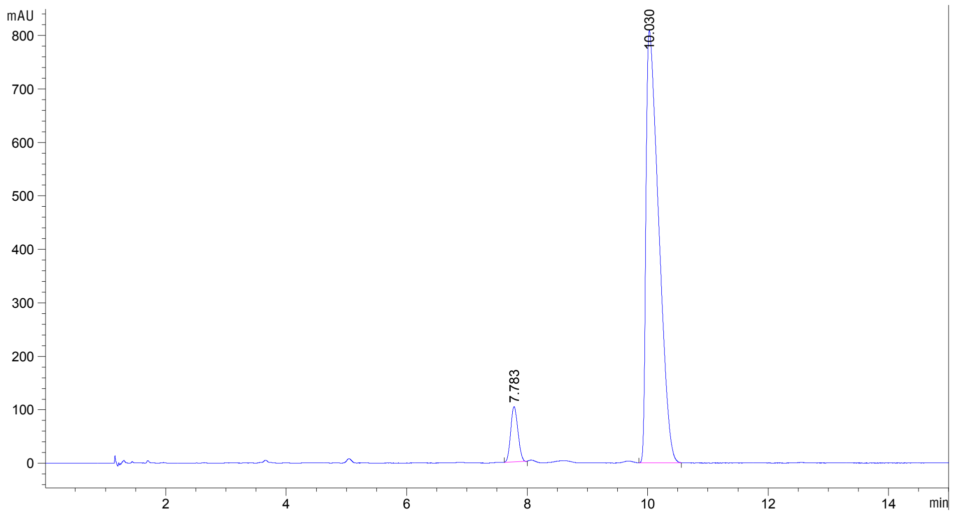

Peak #	RetTime [min]	Type	Width [min]	Area [mAU*s]	Height [mAU]	Area %
1	7.783	BB	0.1301	859.65576	103.33488	6.7571
2	10.030	BB	0.2302	1.18625e4	808.23297	93.2429

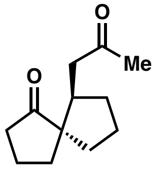

8d

(5*R*,6*R*)-6-(2-oxopropyl)spiro[4.4]nonan-1-one (8d)

Prepared from **7d** following General Procedure A. Purification by flash column chromatography (0–60% Et_2O/hexanes) afforded the title compound as a colorless oil (17.1 mg, 0.088 mmol, 88% yield, 84% ee).

^{1}H NMR (400 MHz, $CDCl_3$): δ 2.64 – 2.51 (m, 1H), 2.42 – 2.23 (m, 3H), 2.19 – 2.07 (m, 4H), 2.06 – 1.97 (m, 1H), 1.97 – 1.87 (m, 1H), 1.85 – 1.73 (m, 2H), 1.73 – 1.60 (m, 4H), 1.59 – 1.47 (m, 1H), 1.30 – 1.13 (m, 1H).

^{13}C NMR (100 MHz, $CDCl_3$): δ 222.9, 208.5, 58.7, 45.6, 40.6, 37.9, 37.7, 32.1, 31.2, 29.9, 22.9, 19.5.

IR (Neat Film, NaCl): 2955, 2872, 1730, 1406, 1357, 1158 cm^{-1}.

HRMS (ESI+): *m/z* calc'd for $C_{12}H_{19}O_2$ $[M+H]^+$: 195.1380, found 195.1370.

Optical Rotation: $[\alpha]_D^{23}$ +66.6 (c = 0.59, $CHCl_3$).

SFC conditions: 25% IPA, 2.5 mL/min, Chiralpak IC-3 column, MSD1 TIC[2], t_R (min): major = 4.16, minor = 4.78

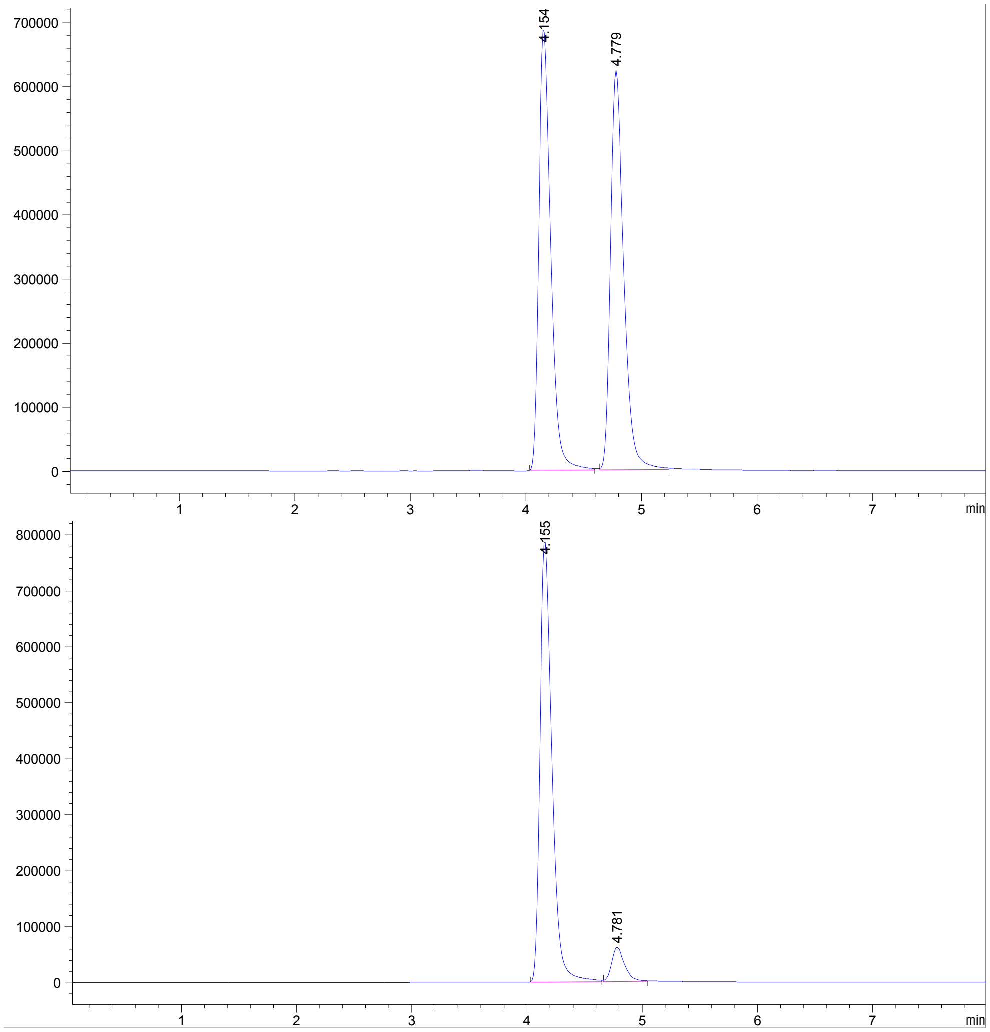

Peak #	RetTime [min]	Type	Width [min]	Area	Height	Area %
1	4.155	BB	0.1094	5.68562e6	7.91363e5	92.1278
2	4.781	BB	0.1177	4.85827e5	6.15655e4	7.8722

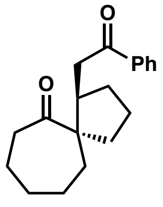

8e

(1*R*,5*S*)-1-(2-oxo-2-phenylethyl)spiro[4.6]undecan-6-one (8e)

Prepared from **7e** following General Procedure A. Purification by flash column chromatography (0–60% Et_2O/hexanes) afforded the title compound as a colorless oil (18.7 mg, 0.066 mmol, 66% yield, 90% ee).

^{1}H NMR (400 MHz, CDCl$_3$): δ 8.06 – 7.99 (m, 2H), 7.60 – 7.52 (m, 1H), 7.51 – 7.43 (m, 2H), 3.25 (dd, J = 14.5, 4.3 Hz, 1H), 2.85 – 2.72 (m, 1H), 2.67 – 2.52 (m, 3H), 2.01 – 1.79 (m, 3H), 1.78 – 1.55 (m, 9H), 1.54 – 1.46 (m, 1H), 1.44 – 1.30 (m, 1H).

^{13}C NMR (100 MHz, CDCl$_3$): δ 217.2, 200.3, 136.9, 133.1, 128.7, 128.5, 60.7, 42.7, 42.7, 40.3, 37.5, 31.5, 31.0, 30.2, 26.4, 25.8, 22.2.

IR (Neat Film, NaCl): 2931, 2858, 1686, 1596, 1579, 1448, 1287, 1215 cm^{-1}.

HRMS (ESI+): *m/z* calc'd for $C_{19}H_{25}O_2$ $[M+H]^+$: 285.1849, found 285.1841.

Optical Rotation: $[\alpha]_D^{23}$ +16.2 (c = 1.56, $CHCl_3$).

SFC conditions: 20% IPA, 2.5 mL/min, Chiralpak IC-3 column, λ = 254 nm, t_R (min): major = 10.28, minor = 11.76

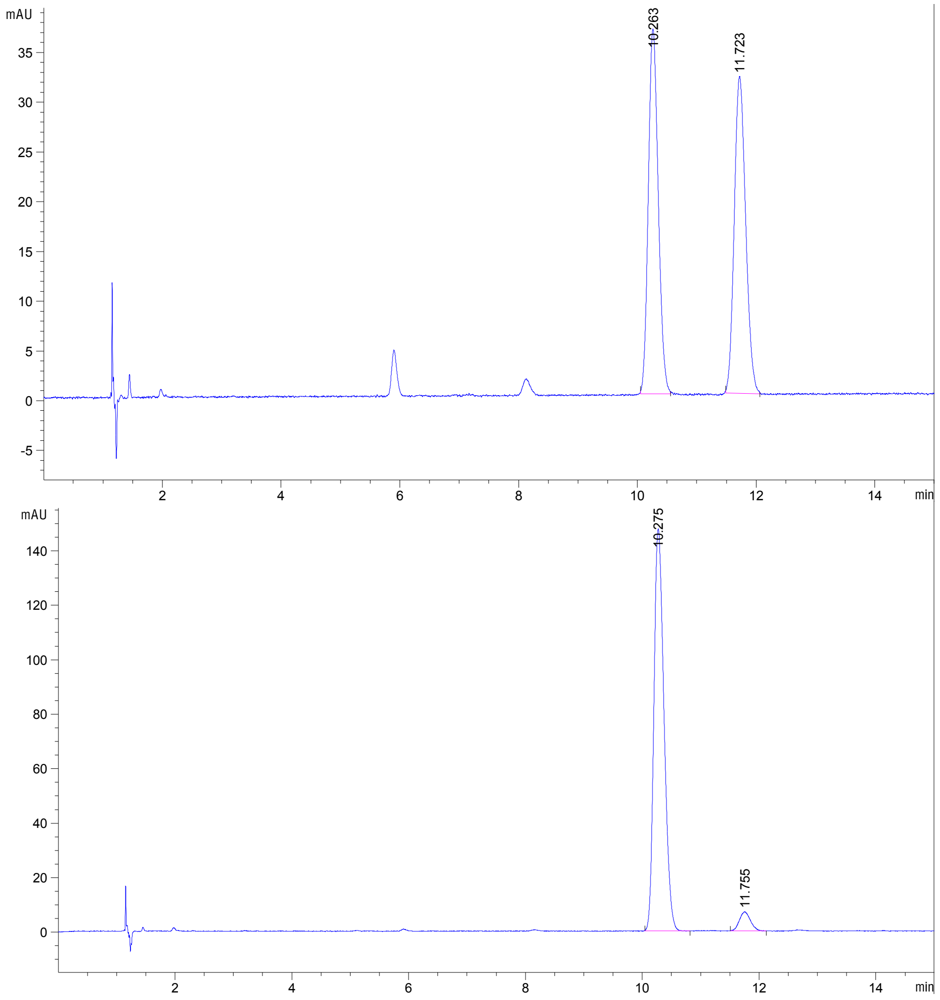

Peak #	RetTime [min]	Type	Width [min]	Area [mAU*s]	Height [mAU]	Area %
1	10.275	BB	0.1881	1770.56360	147.45111	95.0521
2	11.755	BB	0.2015	92.16619	7.00383	4.9479

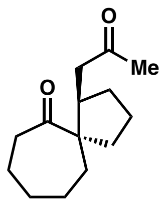

8f

(1*R*,5*S*)-1-(2-oxopropyl)spiro[4.6]undecan-6-one (8f)
Prepared from **7f** following General Procedure A. Purification by flash column chromatography (0–60% Et_2O/hexanes) afforded the title compound as a colorless oil (18.8 mg, 0.085 mmol, 85% yield, 86% ee).

^{1}H NMR (400 MHz, $CDCl_3$): δ 2.70 – 2.46 (m, 4H), 2.23 (dd, *J* = 15.2, 9.9 Hz, 1H), 2.14 (s, 3H), 2.02 – 1.85 (m, 2H), 1.81 – 1.72 (m, 1H), 1.72 – 1.44 (m, 10H), 1.30 – 1.17 (m, 1H).

^{13}C NMR (100 MHz, $CDCl_3$): δ 217.1, 208.7, 60.5, 45.3, 42.7, 42.1, 37.1, 31.3, 31.0, 30.6, 30.0, 26.3, 25.7, 22.3.

IR (Neat Film, NaCl): 2931, 2858, 1713, 1697, 1453, 1354, 1159 cm^{-1}.

HRMS (ESI+): *m/z* calc'd for $C_{14}H_{23}O_2$ $[M+H]^+$: 223.1693, found 223.1683.

Optical Rotation: $[\alpha]_D^{23}$ +30.9 (c = 0.73, $CHCl_3$).

SFC conditions: 30% IPA, 2.5 mL/min, Chiralpak IC-3 column, MSD1 TIC[2], t_R (min): major = 3.90, minor = 6.06

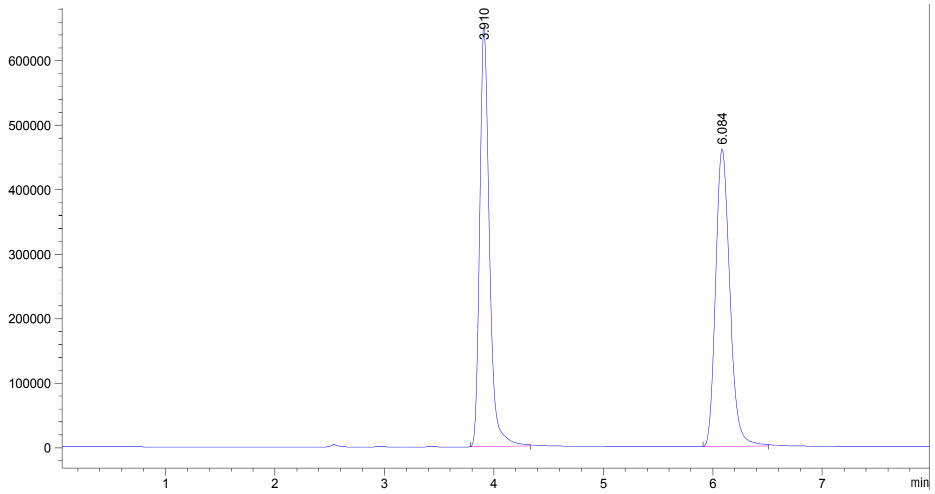

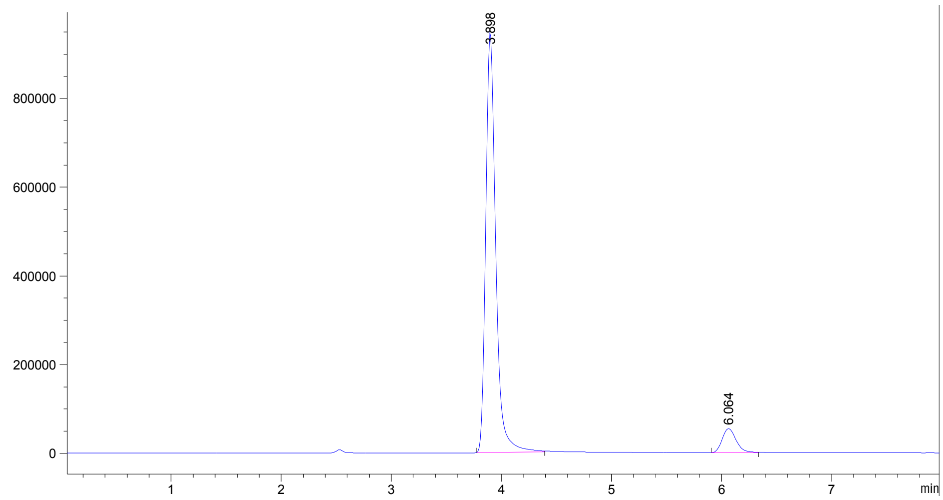

Peak #	RetTime [min]	Type	Width [min]	Area	Height	Area %
1	3.898	BB	0.1002	6.08396e6	9.51561e5	92.8255
2	6.064	BB	0.1331	4.70229e5	5.42479e4	7.1745

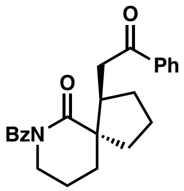

8g

(1*R*,5*R*)-7-benzoyl-1-(2-oxo-2-phenylethyl)-7-azaspiro[4.5]decan-6-one (8g)
Prepared from **7g** following General Procedure A. Purification by flash column chromatography (0–80% Et_2O/hexanes) afforded the title compound as a white foam (24.6 mg, 0.066 mmol, 66% yield, 68% ee).

^{1}H NMR (600 MHz, $CDCl_3$): δ 7.93 – 7.89 (m, 2H), 7.59 – 7.55 (m, 2H), 7.54 – 7.50 (m, 1H), 7.47 – 7.40 (m, 3H), 7.37 (t, *J* = 7.7 Hz, 2H), 3.96 (dtd, *J* = 12.7, 4.5, 1.7 Hz, 1H), 3.70 (ddd, *J* = 12.7, 10.9, 4.8 Hz, 1H), 3.19 – 3.11 (m, 1H), 3.06 (dd, *J* = 15.0, 5.2 Hz, 1H), 2.80 (dd, *J* = 15.0, 9.6 Hz, 1H), 2.11 – 1.93 (m, 5H), 1.89 – 1.82 (m, 1H), 1.80 – 1.71 (m, 3H), 1.40 – 1.32 (m, 1H).

^{13}C NMR (100 MHz, $CDCl_3$): δ 199.6, 178.5, 175.5, 136.8, 136.8, 133.1, 131.4, 128.7, 128.3, 128.3, 127.6, 53.8, 46.5, 41.9, 39.7, 38.4, 29.9, 27.4, 22.3, 20.1.

IR (Neat Film, NaCl): 3059, 2951, 2872, 1681, 1597, 1475, 1448, 1386, 1277, 1169, 1151 cm^{-1}.

HRMS (ESI+): *m/z* calc'd for $C_{24}H_{26}NO_3$ $[M+H]^+$: 376.1907, found 376.1907.

Optical Rotation: $[\alpha]_D^{23}$ +51.6 (c = 2.05, $CHCl_3$).

SFC conditions: 40% IPA, 2.5 mL/min, Chiralpak IC-3 column, λ = 254 nm, t_R (min): minor = 5.93, major = 9.07

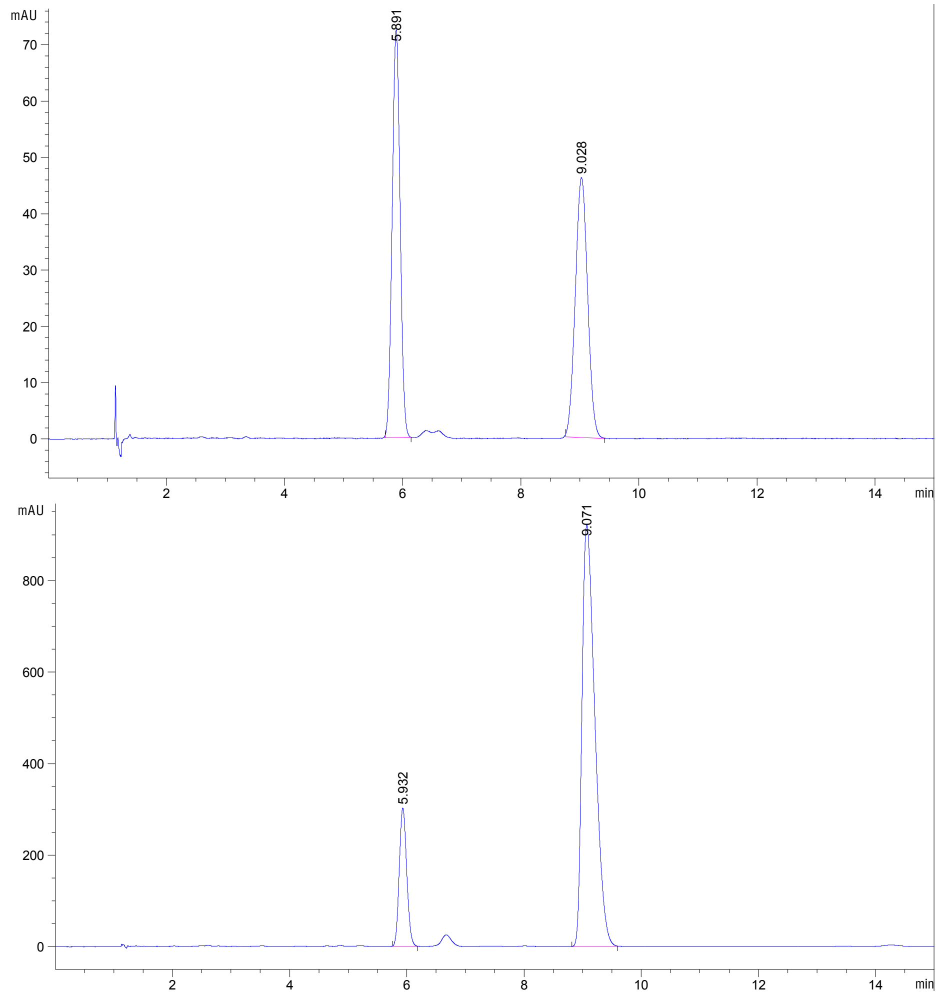

Peak #	RetTime [min]	Type	Width [min]	Area [mAU*s]	Height [mAU]	Area %
1	5.932	BB	0.1371	2673.71484	302.54599	15.9689
2	9.071	BB	0.2361	1.40695e4	921.51959	84.0311

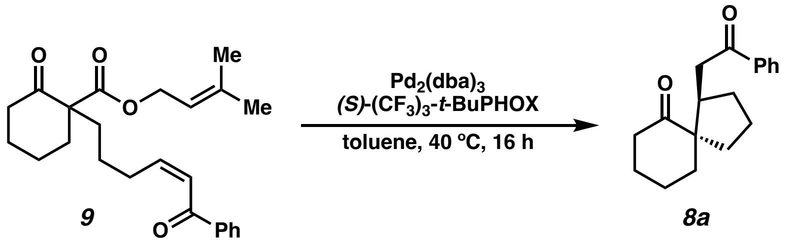

(1*R*,5*S*)-1-(2-oxo-2-phenylethyl)spiro[4.5]decan-6-one (8a)

Prepared from **9** following General Procedure A. Purification by flash column chromatography (0–55% Et_2O/hexanes) afforded the title compound as a colorless oil (18.2 mg, 0.067 mmol, 67% yield, 92% ee).

Note: see NMR, IR, HRMS, Optical Rotation data in reaction of 7a to 8a earlier in SI.

SFC conditions: 20% IPA, 2.5 mL/min, Chiralpak IC-3 column, λ = 254 nm, t_R (min): minor = 6.82, major = 8.44

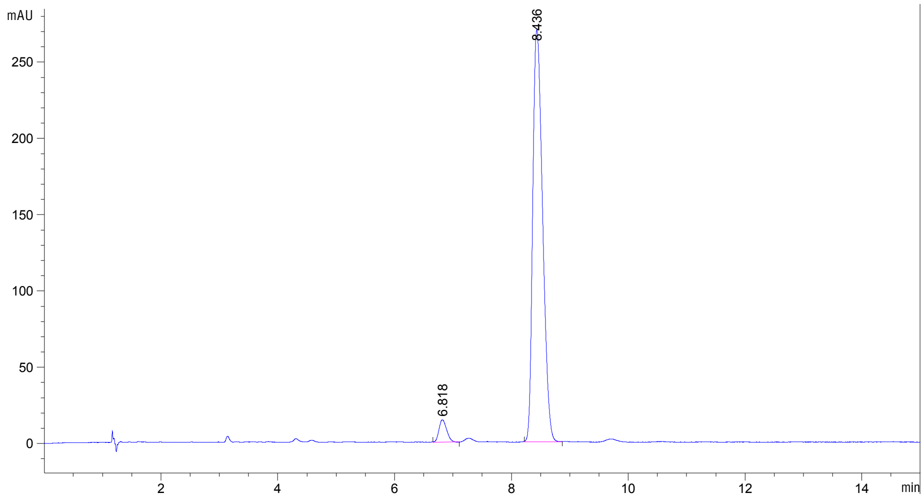

Peak #	RetTime [min]	Type	Width [min]	Area [mAU*s]	Height [mAU]	Area %
1	6.818	BB	0.1399	132.92445	14.64637	4.0317
2	8.436	BB	0.1847	3164.02612	270.01862	95.9683

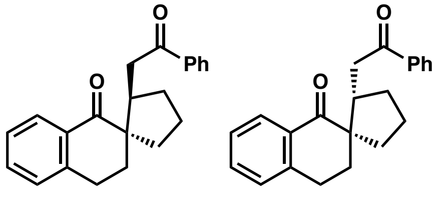

8h (d1) ***8h (d2)***

(1*R*,2*R*)-2-(2-oxo-2-phenylethyl)-3',4'-dihydro-1'*H*-spiro[cyclopentane-1,2'-naphthalen]-1'-one (8h, *d1* and *d2*)

Prepared from **7h** following General Procedure A. Crude analysis by ^{1}H NMR affords a 12:1 ratio of *d1* to *d2*. The diastereomers were subsequently separated by flash column chromatography (0–60% Et_2O/hexanes) to afford the *d1* as a colorless oil (17.3 mg, 0.054 mmol, 54% yield, 37% ee) and *d2* as a colorless oil (1.9 mg, 0.006 mmol, 6% yield, 20% ee). The absolute configuration of 8h *d1* is assigned by analogy to **8a**. The relative configuration of **8h *d2*** is assigned by comparison to ^{1}H NMR of **8h *d1***, the absolute configuration is not determined.

8h (*d1*):

^{1}H NMR (400 MHz, $CDCl_3$): δ 8.08 (dd, *J* = 7.8, 1.4 Hz, 1H), 8.05 – 7.97 (m, 2H), 7.60 – 7.52 (m, 1H), 7.51 – 7.43 (m, 3H), 7.32 (t, *J* = 7.6 Hz, 1H), 7.24 (d, *J* = 7.8 Hz, 1H), 3.40 – 3.29 (m, 1H), 3.21 – 3.09 (m, 2H), 3.00 – 2.89 (m, 1H), 2.68 (dd, *J* = 14.5, 10.6 Hz, 1H), 2.14 – 1.89 (m, 4H), 1.82 – 1.68 (m, 2H), 1.68 – 1.60 (m, 1H), 1.50 – 1.35 (m, 1H).

^{13}C NMR (100 MHz, $CDCl_3$): δ 201.3, 200.2, 143.3, 136.9, 133.3, 133.1, 132.6, 128.8, 128.7, 128.5, 128.1, 126.8, 55.1, 40.8, 40.0, 33.6, 29.8, 27.1, 25.8, 22.5.

IR (Neat Film, NaCl): 3061, 2933, 2868, 1679, 1597, 1448, 1288, 1224 cm^{-1}.

HRMS (ESI+): *m/z* calc'd for $C_{22}H_{23}O_2$ $[M+H]^+$: 319.1693, found 319.1687.

Optical Rotation: $[\alpha]_D^{23}$ –9.7 (c = 1.44, $CHCl_3$).

SFC conditions: 25% IPA, 2.5 mL/min, Chiralpak IC-3 column, λ = 210 nm, t_R (min): major = 8.97, minor = 10.37

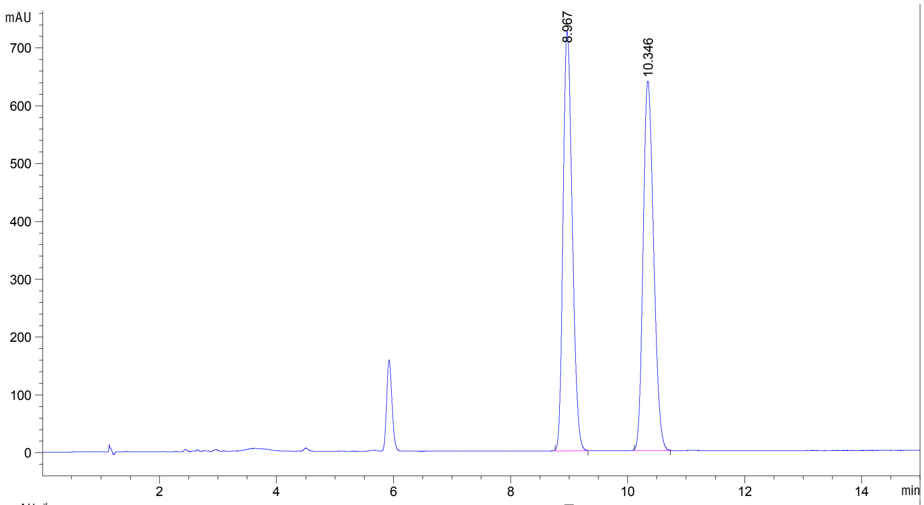

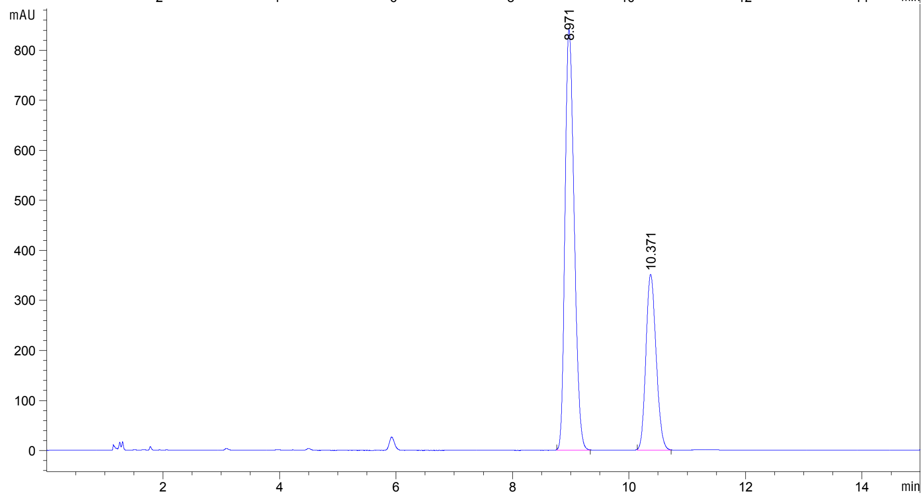

Peak #	RetTime [min]	Type	Width [min]	Area [mAU*s]	Height [mAU]	Area %
1	8.971	BB	0.1720	9226.19922	841.01697	68.3331
2	10.371	BB	0.1899	4275.60645	351.56262	31.6669

8h (*d2*):

^{1}H NMR (400 MHz, $CDCl_3$): δ 8.04 (dd, *J* = 7.9, 1.5 Hz, 1H), 7.94 – 7.80 (m, 2H), 7.55 – 7.36 (m, 4H), 7.36 – 7.28 (m, 1H), 7.24 – 7.19 (m, 1H), 3.27 (dd, *J* = 17.1, 10.0 Hz, 1H), 3.04 (t, *J* = 6.2 Hz, 2H), 2.84 (dd, *J* = 17.1, 3.5 Hz, 1H), 2.58 – 2.46 (m, 1H), 2.34 – 2.21 (m, 2H), 2.20 – 2.08 (m, 1H), 1.94 (dt, *J* = 13.5, 5.3 Hz, 1H), 1.84 – 1.62 (m, 4H).

^{13}C NMR (100 MHz, $CDCl_3$): δ 201.7, 200.2, 143.6, 137.4, 133.3, 133.0, 132.6, 128.8, 128.6, 128.2, 127.8, 126.8, 55.7, 44.6, 40.2, 36.0, 35.3, 32.8, 27.0, 22.8.

Note: A grease signal is observed in the ^{1}H and ^{13}C spectra.

IR (Neat Film, NaCl): 2922, 2851, 1681, 1598, 1448, 1279, 1219 cm^{-1}.

HRMS (FD+): *m/z* calc'd for $C_{22}H_{22}O_2$ $[M]^{+\cdot}$: 318.16143, found 318.16025.

Note: The optical rotation measurement for this compound had a standard deviation greater than its magnitude (3.76) and is therefore unreliable.

Optical Rotation: $[\alpha]_D^{23}$ +0.04 (c = 0.16, $CHCl_3$).

SFC conditions: 25% IPA, 2.5 mL/min, Chiralpak IC-3 column, λ = 210 nm, t_R (min): minor = 3.85, major = 4.15

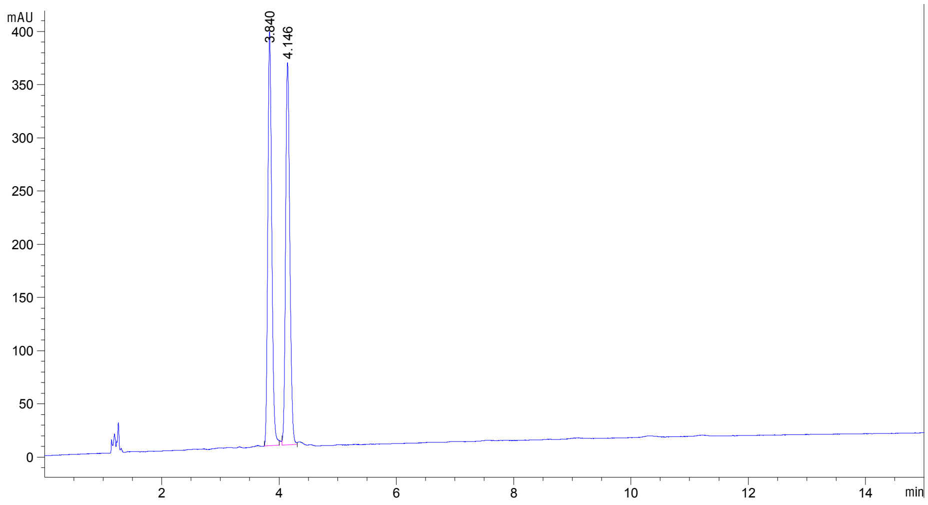

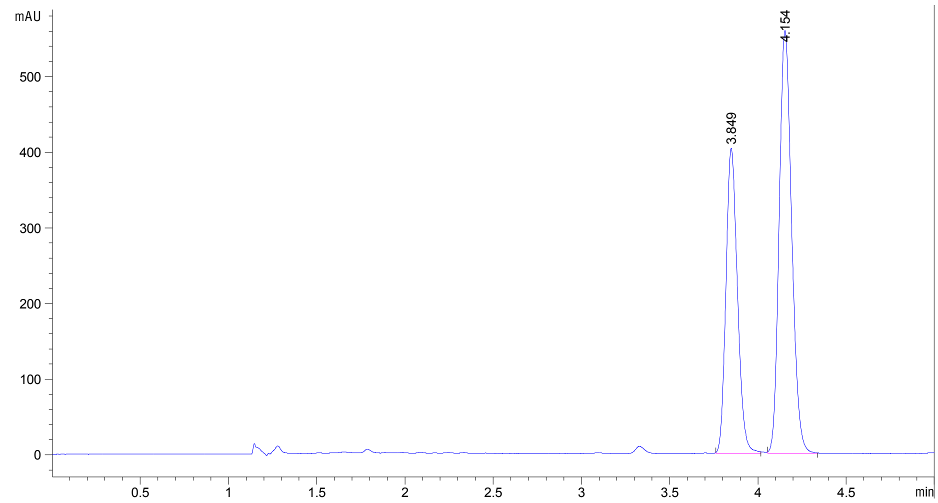

Peak #	RetTime [min]	Type	Width [min]	Area [mAU*s]	Height [mAU]	Area %
1	3.849	BB	0.0699	1828.53516	403.17871	39.9390
2	4.154	BB	0.0764	2749.78320	558.60046	60.0610

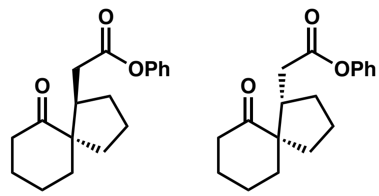

8i (d1) ***8i (d2)***

phenyl 2-((1*R*,5*S*)-6-oxospiro[4.5]decan-1-yl)acetate (8i, *d1* and *d2*)

Prepared from **7i** following General Procedure A. Crude analysis by ^{1}H NMR affords a 3:1 ratio of *d1* to *d2*. The diastereomers were subsequently separated by flash column chromatography (0–60% Et_2O/hexanes) to afford the *d1* as a colorless oil (16.9 mg, 0.059 mmol, 59% yield, 82% ee) and, after subsequent preparative TLC purification (5% Et_2O, 47.5% toluene, 47.5% hexanes) *d2* as a colorless oil (1.9 mg, 0.007 mmol, 7% yield, 71% ee). The absolute configuration of **8i *d1*** is assigned by analogy to **8a**. The relative configuration of **8i *d2*** is assigned by comparison to ^{1}H NMR of **8i *d1***, the absolute configuration is not determined.

8i (*d1*):

^{1}H NMR (400 MHz, $CDCl_3$): δ 7.42 – 7.32 (m, 2H), 7.25 – 7.16 (m, 1H), 7.14 – 7.06 (m, 2H), 3.12 – 3.00 (m, 1H), 2.64 (dd, J = 14.6, 5.4 Hz, 1H), 2.49 – 2.28 (m, 3H), 2.09 – 1.85 (m, 3H), 1.83 – 1.54 (m, 8H), 1.49 – 1.35 (m, 1H).

^{13}C NMR (100 MHz, $CDCl_3$): δ 213.5, 171.6, 150.8, 129.4, 125.7, 121.7, 58.1, 40.0, 39.4, 35.3, 35.3, 31.5, 29.9, 26.9, 21.8, 21.7.

IR (Neat Film, NaCl): 2938, 2865, 1757, 1702, 1593, 1492, 1455, 1230, 1195, 1126 cm^{-1}.

HRMS (ESI+): *m/z* calc'd for $C_{18}H_{23}O_3$ $[M+H]^+$: 287.1642, found 287.1629.

Optical Rotation: $[\alpha]_D^{23}$ +5.0 (c = 1.41, $CHCl_3$).

SFC conditions: 20% IPA, 2.5 mL/min, Chiralpak OD-3 column, λ = 210 nm, t_R (min): major = 2.80, minor = 2.99

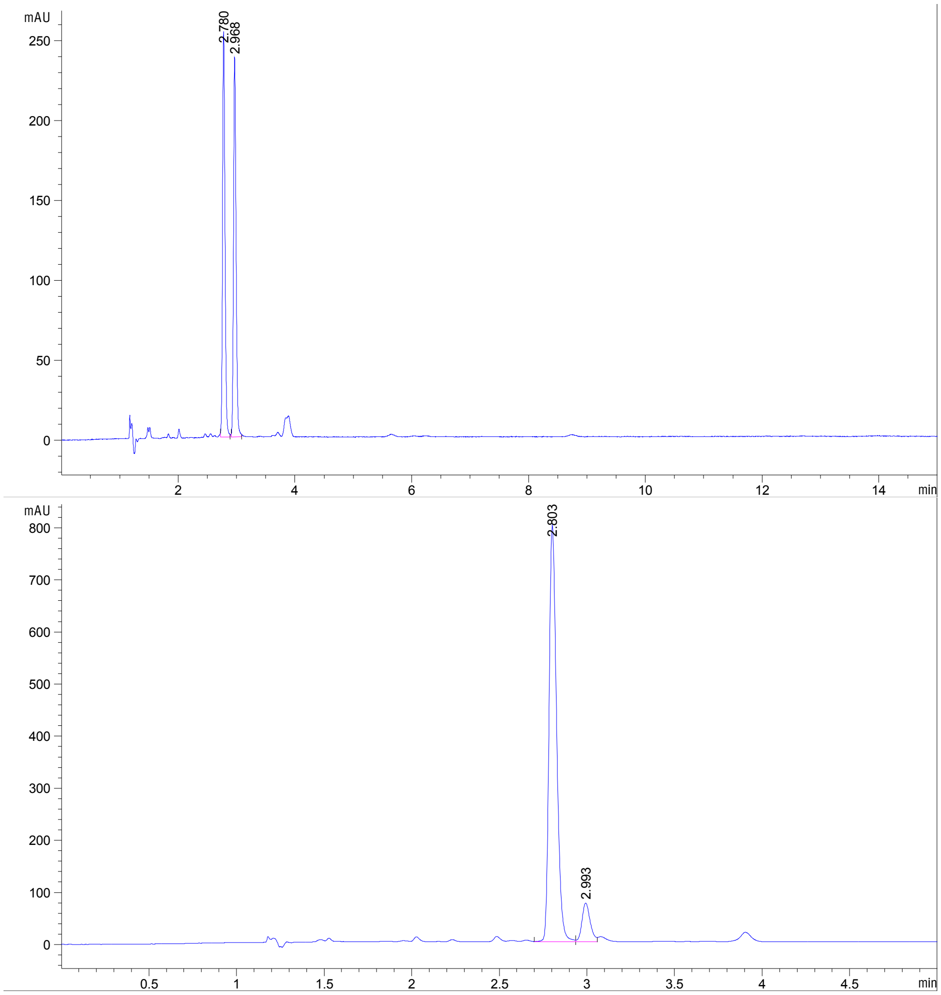

Peak #	RetTime [min]	Type	Width [min]	Area [mAU*s]	Height [mAU]	Area %
1	2.803	VV	0.0469	2389.49536	799.23358	90.7891
2	2.993	VV	0.0501	242.42239	74.26399	9.2109

8i (*d2*):

^{1}H NMR (600 MHz, $CDCl_3$): δ 7.37 (t, *J* = 7.8 Hz, 2H), 7.22 (t, *J* = 7.4 Hz, 1H), 7.10 – 7.03 (m, 2H), 2.80 (dd, *J* = 15.9, 10.6 Hz, 1H), 2.61 – 2.45 (m, 2H), 2.29 (dt, *J* = 14.9, 5.6 Hz, 1H), 2.25 – 2.15 (m, 2H), 2.13 – 2.03 (m, 1H), 1.96 (ddd, *J* = 13.7, 9.8, 3.8 Hz, 1H), 1.92 – 1.80 (m, 2H), 1.79 – 1.64 (m, 5H), 1.63 – 1.58 (m, 1H), 1.53 – 1.47 (m, 1H).

^{13}C NMR (100 MHz, CDCl$_3$): δ 213.9, 172.2, 150.8, 129.4, 125.7, 121.6, 59.3, 44.6, 40.4, 38.2, 36.3, 35.3, 30.3, 26.5, 22.6, 21.5.

IR (Neat Film, NaCl): 2925, 2855, 1756, 1697, 1592, 1492, 1197, 1126 cm^{-1}.

HRMS (FD+): *m/z* calc'd for $C_{18}H_{22}O_3$ $[M]^{+\cdot}$: 286.15635, 286.15495 found.

Optical Rotation: $[\alpha]_D^{23}$ –7.9 (c = 0.16 $CHCl_3$).

SFC conditions: 10% IPA, 2.5 mL/min, Chiralpak OD-3 column, λ = 210 nm, t_R (min): major = 3.99, minor = 4.23

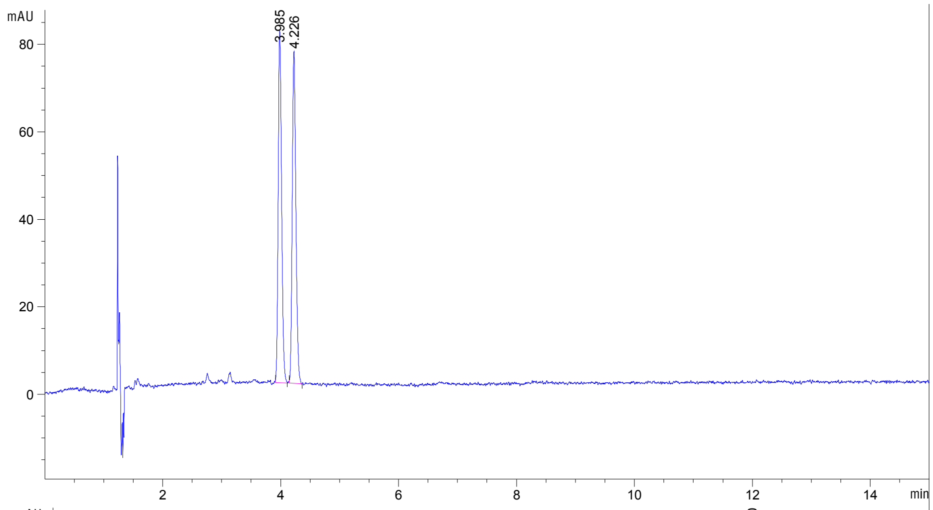

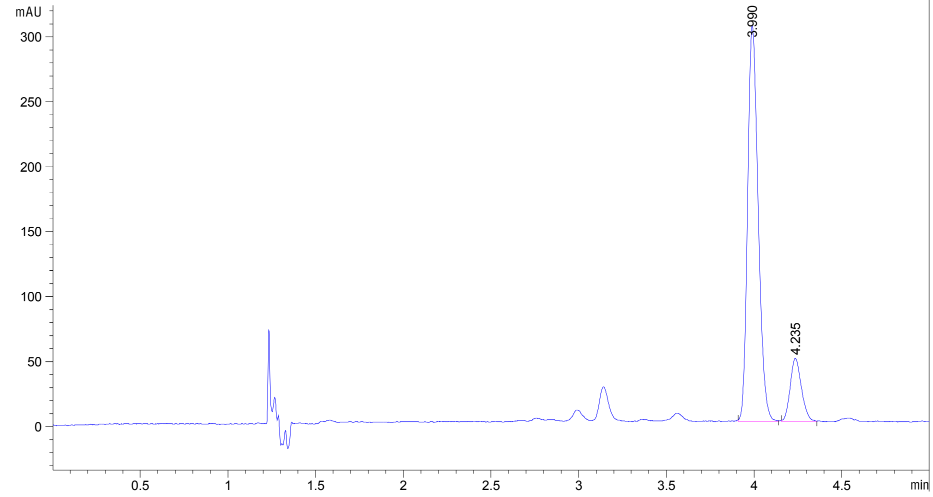

Peak #	RetTime [min]	Type	Width [min]	Area [mAU*s]	Height [mAU]	Area %
1	3.990	BB	0.0649	1275.57300	304.15570	85.4887
2	4.235	BB	0.0681	216.52251	48.50142	14.5113

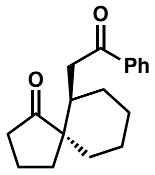

8j

(5*R*,6*R*)-6-(2-oxo-2-phenylethyl)spiro[4.5]decan-1-one (8j)
Prepared from **7j** following General Procedure A. Purification by flash column chromatography (0–60% Et_2O/hexanes) afforded the title compound as a colorless oil (16.9 mg, 0.063 mmol, 63% yield, 71% ee).

^{1}H NMR (400 MHz, $CDCl_3$): δ 7.99 – 7.88 (m, 2H), 7.59 – 7.51 (m, 1H), 7.50 – 7.42 (m, 2H), 2.70 (dd, J = 13.6, 2.9 Hz, 1H), 2.57 – 2.32 (m, 3H), 2.23 – 2.11 (m, 1H), 2.06 – 1.83 (m, 4H), 1.72 – 1.51 (m, 4H), 1.43 – 1.18 (m, 3H), 1.13 – 0.99 (m, 1H).

^{13}C NMR (100 MHz, $CDCl_3$): δ 223.9, 199.6, 136.7, 133.2, 128.7, 128.6, 54.0, 42.1, 38.9, 38.1, 33.4, 28.4, 27.7, 25.7, 21.8, 19.1.

IR (Neat Film, NaCl): 2929, 2854, 1728, 1683, 1447, 1291, 1209 cm^{-1}.

HRMS (ESI+): *m/z* calc'd for $C_{18}H_{23}O_2$ $[M+H]^+$: 271.1693, found 271.1681.

Note: The optical rotation measurement for this compound had a standard deviation greater than its magnitude (0.73) and is therefore unreliable.

Optical Rotation: $[\alpha]_D^{23}$ –0.38 (c = 0.97, $CHCl_3$).

SFC conditions: 20% IPA, 2.5 mL/min, Chiralpak IC-3 column, λ = 230 nm, t_R (min): minor = 4.95, major = 8.16

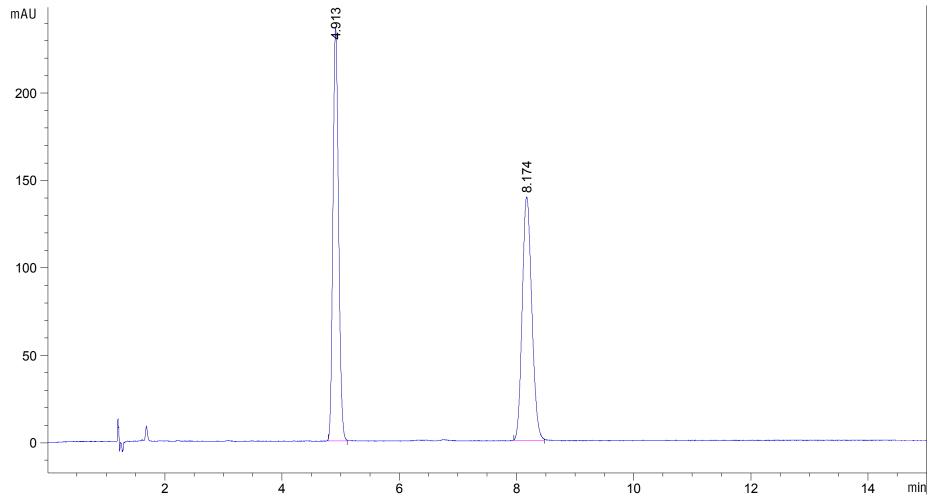

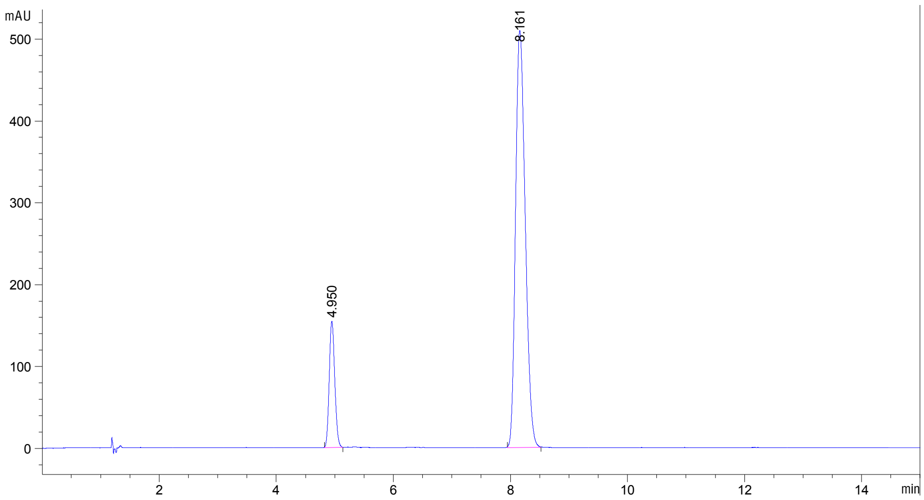

Peak #	RetTime [min]	Type	Width [min]	Area [mAU*s]	Height [mAU]	Area %
1	4.950	BB	0.1017	1013.17133	154.21021	14.7376
2	8.161	BB	0.1802	5861.57813	509.57901	85.2624

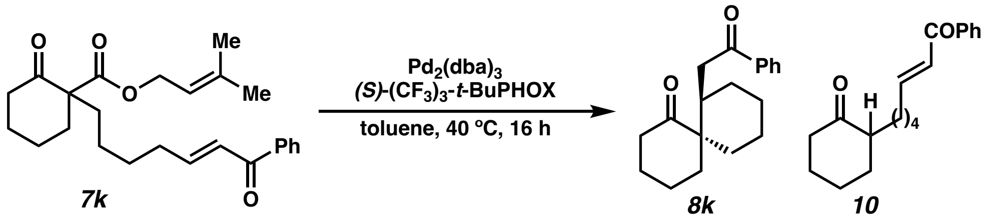

(6*R*,7*R*)-7-(2-oxo-2-phenylethyl)spiro[5.5]undecan-1-one (8k) and (*E*)-2-(7-oxo-7-phenylhept-5-en-1-yl)cyclohexan-1-one (10)

Prepared from **7k** following General Procedure A. Purification by flash column chromatography (0–60% Et_2O/hexanes) afforded the compound **8k** as a colorless oil (13.8 mg, 0.049 mmol, 49% yield, 89% ee) and compound **10** as a colorless oil (5.1 mg, 0.018 mmol, 18% yield).

8k:

^{1}H NMR (400 MHz, $CDCl_3$): δ 8.16 – 8.07 (m, 2H), 7.58 – 7.52 (m, 1H), 7.51 – 7.44 (m, 2H), 3.03 (dd, *J* = 14.1, 2.5 Hz, 1H), 2.69 (tt, *J* = 11.1, 3.2 Hz, 1H), 2.60 – 2.41 (m, 2H), 2.37 – 2.26 (m, 1H), 2.19 – 1.96 (m, 3H), 1.82 – 1.70 (m, 2H), 1.70 – 1.59 (m, 2H), 1.59 – 1.46 (m, 4H), 1.28 – 1.08 (m, 3H).

^{13}C NMR (100 MHz, $CDCl_3$): δ 215.9, 200.7, 137.1, 133.0, 128.7, 128.6, 52.2, 41.8, 38.7, 36.8, 33.1, 27.2, 26.6, 26.4, 25.4, 20.8, 20.3.

IR (Neat Film, NaCl): 2931, 2858, 1697, 1596, 1447, 1287, 1126 cm^{-1}.

HRMS (ESI+): *m/z* calc'd for $C_{19}H_{25}O_2$ $[M+H]^+$: 285.1849, found 285.1841.

Optical Rotation: $[\alpha]_D^{23}$ –2.5 (c = 0.425, $CHCl_3$).

SFC conditions: 20% IPA, 2.5 mL/min, Chiralpak IC-3 column, λ = 210 nm, t_R (min): minor = 7.13, major = 11.24

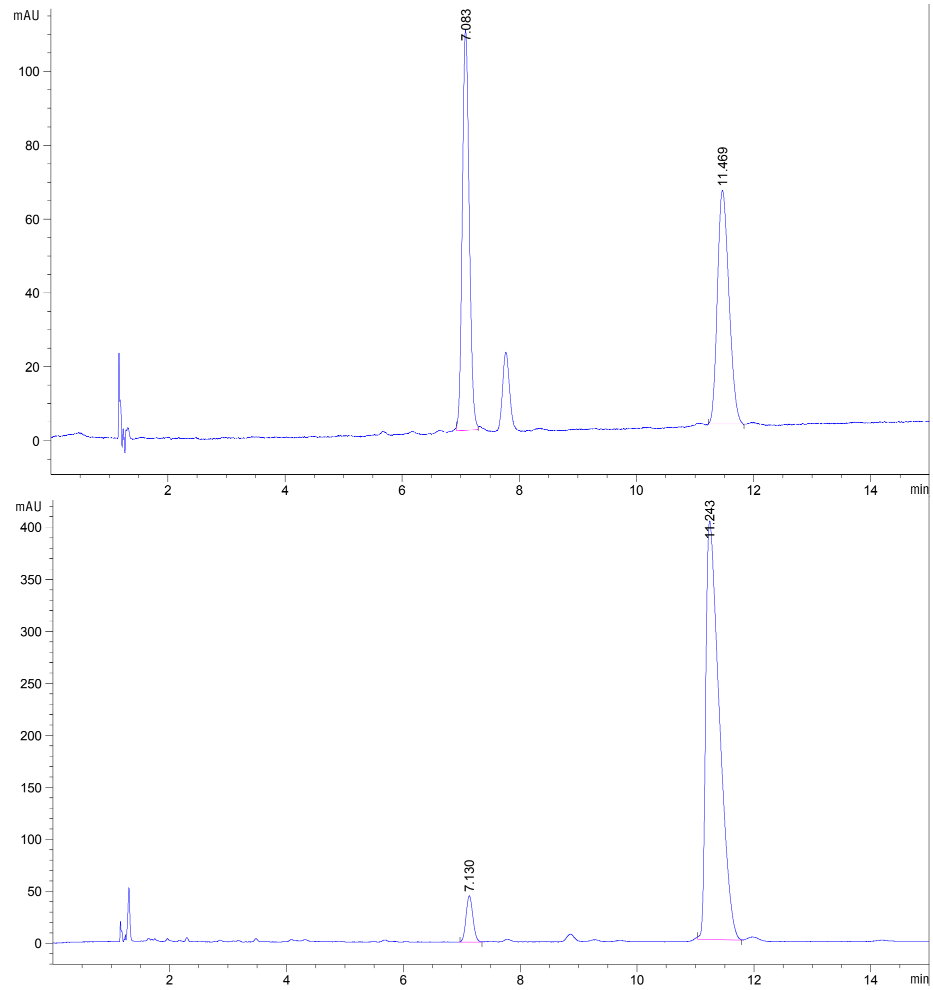

Peak #	RetTime [min]	Type	Width [min]	Area [mAU*s]	Height [mAU]	Area %
1	7.130	BB	0.1255	360.87848	44.55806	5.2579
2	11.243	BB	0.2394	6502.66650	402.65048	94.7421

10:

^{1}H NMR (400 MHz, $CDCl_3$): δ 7.96 – 7.90 (m, 2H), 7.59 – 7.52 (m, 1H), 7.51 – 7.43 (m, 2H), 7.05 (dt, *J* = 15.3, 6.9 Hz, 1H), 6.87 (dt, *J* = 15.4, 1.4 Hz, 1H), 2.44 – 2.21 (m, 5H), 2.15 – 1.97 (m, 2H), 1.92 – 1.75 (m, 2H), 1.73 – 1.62 (m, 2H), 1.57 – 1.48 (m, 2H), 1.41 – 1.29 (m, 3H), 1.27 – 1.16 (m, 1H).

^{13}C NMR (100 MHz, $CDCl_3$): δ 213.5, 191.1, 150.0, 138.1, 132.7, 128.7, 128.7, 126.1, 50.8, 42.2, 34.1, 32.9, 29.3, 28.5, 28.2, 27.0, 25.1.

IR (Neat Film, NaCl): 2931, 2857, 1707, 1669, 1618, 1447, 1338, 1286, 1224, 1128 cm^{-1}.

HRMS (ESI+): *m/z* calc'd for $C_{19}H_{25}O_2$ $[M+H]^+$: 285,1849, found 285.1835.

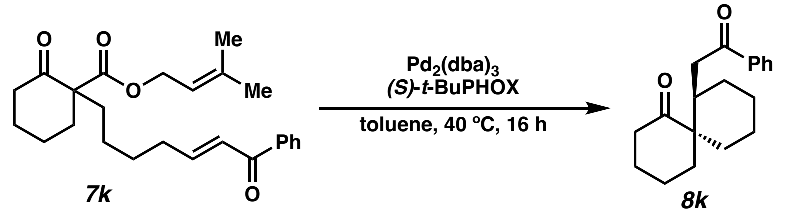

(6*R*,7*R*)-7-(2-oxo-2-phenylethyl)spiro[5.5]undecan-1-one (8k)

In a nitrogen filled glovebox, an oven-dried 4 mL vial was charged with a stir bar, $Pd_2(dba)_3$ (2.3 mg, 0.0025 mmol, 2.5 mol %), *(S)-t*-BuPHOX (2.5 mg, 0.0065 mmol, 6.5 mol %), and toluene (0.6 mL). The catalyst solution was stirred at 23 ºC for 20 min. A solution of substrate **7k** (0.1 mmol, 1 equiv) in toluene (0.4 mL) was added to the catalyst solution. The resulting mixture was sealed, pumped out of the glovebox, and then heated to 40 ºC for 16 h. The solution was then cooled to 23 ºC and concentrated under reduced pressure. Purification by flash column chromatography (0–60% Et_2O/hexanes) afforded the compound 8k as a colorless oil (18.3 mg, 0.064 mmol, 64% yield, 86% ee).

Note: see NMR, IR, HRMS, Optical Rotation data in reaction of 7k to 8k and 10 earlier in SI.

SFC conditions: 20% IPA, 2.5 mL/min, Chiralpak IC-3 column, λ = 210 nm, t_R (min): minor = 7.23, major = 11.41

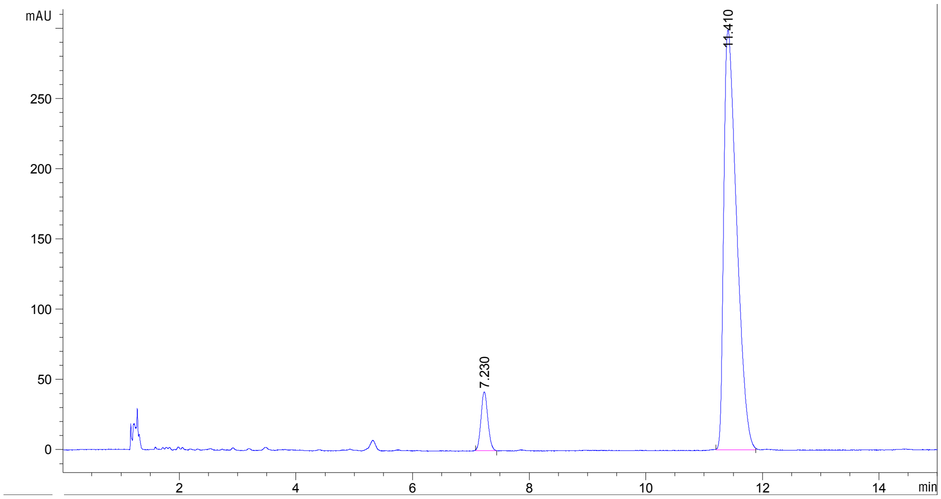

Peak #	RetTime [min]	Type	Width [min]	Area [mAU*s]	Height [mAU]	Area %
1	7.230	BB	0.1276	343.79590	41.95350	6.9891
2	11.410	BB	0.2316	4575.23535	298.83871	93.0109

Protonated Byproducts:

General Procedure B: Undesired Pd-Catalyzed Decarboxylative Protonation

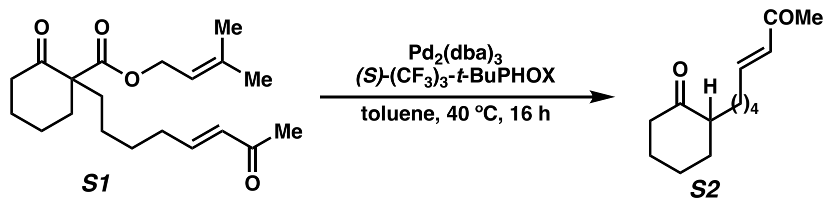

In a nitrogen filled glovebox, an oven-dried 4 mL vial was charged with a stir bar, $Pd_2(dba)_3$ (2.3 mg, 0.0025 mmol, 2.5 mol %), *(S)*-$(CF_3)_3$-*t*-BuPHOX (3.8 mg, 0.0065 mmol, 6.5 mol %), and toluene (0.6 mL). The catalyst solution was stirred at 23 ºC for 20 min. A solution of substrate **S1** (0.1 mmol, 1 equiv) in toluene (0.4 mL) was added to the catalyst solution. The resulting mixture was sealed, pumped out of the glovebox, and then heated to 40 ºC for 16 h. The solution was then cooled to 23 ºC and concentrated under reduced pressure. The crude reaction mixture was loaded directly onto a flash column and the product (**S2**) was isolated by silica gel flash column chromatography.

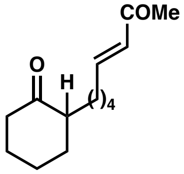

S2a

(*E*)-2-(7-oxooct-5-en-1-yl)cyclohexan-1-one (S2a)
Prepared from **S1a** following General Procedure B. Purification by flash column chromatography (0–60% Et_2O/hexanes) afforded the title compound as a colorless oil (13.8 mg, 0.062 mmol, 62% yield).

^{1}H NMR (400 MHz, $CDCl_3$): δ 6.78 (dt, J = 15.9, 6.8 Hz, 1H), 6.05 (dt, J = 16.0, 1.5 Hz, 1H), 2.43 – 2.34 (m, 1H), 2.33 – 2.16 (m, 7H), 2.14 – 1.96 (m, 2H), 1.91 – 1.71 (m, 2H), 1.70 – 1.57 (m, 2H), 1.54 – 1.41 (m, 2H), 1.39 – 1.13 (m, 4H).

^{13}C NMR (100 MHz, $CDCl_3$): δ 213.4, 198.9, 148.5, 131.5, 50.8, 42.2, 34.1, 32.5, 29.3, 28.4, 28.2, 27.0, 26.9, 25.1.

IR (Neat Film, NaCl): 2931, 2858, 1707, 1673, 1625, 1430, 1360, 1253, 1127 cm^{-1}.

HRMS (ESI+): *m/z* calc'd for $C_{14}H_{22}O_2$ $[M+H]^+$: 223.1693, found 223.1686.

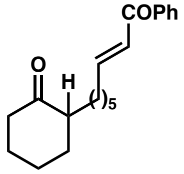

S2b

(*E*)-2-(8-oxo-8-phenyloct-6-en-1-yl)cyclohexan-1-one (S2b)
Prepared from **S1b** following General Procedure B. Purification by flash column chromatography (0–60% Et_2O/hexanes) afforded the title compound as a colorless oil (17.7 mg, 0.059 mmol, 59% yield).

^{1}H NMR (400 MHz, CDCl$_3$): δ 7.97 – 7.88 (m, 2H), 7.59 – 7.51 (m, 1H), 7.49 – 7.42 (m, 2H), 7.05 (dt, *J* = 15.5, 6.9 Hz, 1H), 6.87 (dt, *J* = 15.4, 1.4 Hz, 1H), 2.46 – 2.18 (m, 5H), 2.16 – 1.94 (m, 2H), 1.92 – 1.58 (m, 4H), 1.57 – 1.47 (m, 2H), 1.44 – 1.11 (m, 6H).

Note: 2 carbon signals appear to be coincident.

^{13}C NMR (100 MHz, CDCl$_3$): δ 213.6, 191.1, 150.1, 138.1, 132.7, 128.7, 128.6, 126.1, 50.8, 42.1, 34.1, 32.9, 29.5, 29.5, 28.2, 27.1, 25.0.

IR (Neat Film, NaCl): 2930, 2856, 1708, 1669, 1619, 1447, 1338, 1282, 1222, 1127 cm^{-1}.

HRMS (ESI+): *m/z* calc'd for $C_{20}H_{26}O_2$ [M+H]$^+$: 299.2006, found 299.2002.

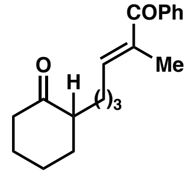

S2c

(*E*)-2-(8-oxo-8-phenyloct-6-en-1-yl)cyclohexan-1-one (S2c)
Prepared from **S1c** following General Procedure B. Purification by flash column chromatography (0–60% Et_2O/hexanes) afforded the title compound as a colorless oil (13.4 mg, 0.047 mmol, 47% yield).

^{1}H NMR (600 MHz, CDCl$_3$): δ 7.62 (d, *J* = 7.6 Hz, 2H), 7.49 (t, *J* = 7.4 Hz, 1H), 7.41 (t, *J* = 7.6 Hz, 2H), 6.28 (t, *J* = 7.4 Hz, 1H), 2.42 – 2.34 (m, 1H), 2.33 – 2.23 (m, 4H), 2.11 – 2.01 (m, 2H), 1.96 (s, 3H), 1.89 – 1.76 (m, 2H), 1.70 – 1.61 (m, 2H), 1.47 – 1.33 (m, 3H), 1.24 – 1.17 (m, 1H).

^{13}C NMR (100 MHz, CDCl$_3$): δ 213.2, 199.2, 146.6, 138.9, 136.7, 131.5, 129.4, 128.2, 50.7, 42.2, 34.2, 29.5, 29.5, 28.2, 26.5, 25.1, 12.6.

IR (Neat Film, NaCl): 2929, 2856, 1707, 1646, 1446, 1284, 1167 cm^{-1}.

HRMS (ESI+): *m/z* calc'd for $C_{19}H_{25}O_2$ [M+H]$^+$: 285.1849, found 285.1841.

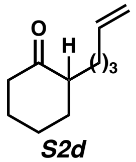

S2d

2-(pent-4-en-1-yl)cyclohexan-1-one (S2d)
Prepared from **S1d** following General Procedure B. Purification by flash column chromatography (0–60% Et_2O/hexanes) afforded the title compound as a colorless oil (5.4 mg, 0.032 mmol, 32% yield).

^{1}H NMR (400 MHz, $CDCl_3$): δ 5.80 (ddt, *J* = 16.9, 10.2, 6.6 Hz, 1H), 5.06 – 4.88 (m, 2H), 2.44 – 2.35 (m, 1H), 2.34 – 2.20 (m, 2H), 2.16 – 1.94 (m, 3H), 1.90 – 1.73 (m, 2H), 1.66 (tddd, *J* = 14.0, 11.6, 7.7, 5.2 Hz, 2H), 1.45 – 1.31 (m, 3H), 1.28 – 1.14 (m, 1H).

Note: 2 carbon signals appear to be coincident.

^{13}C NMR (100 MHz, $CDCl_3$): δ 213.6, 138.9, 114.6, 50.8, 42.2, 34.0, 29.1, 28.2, 26.7, 25.0.

IR (Neat Film, NaCl): 2931, 2859, 1710, 1639, 1447, 1125 cm^{-1}.

HRMS (FI+): *m/z* calc'd for $C_{11}H_{18}O$ $[M]^{+\cdot}$: 166.13522, found 166.13526.

Pd-Catalyzed Decarboxylative Alkene Difunctionalization:
General Procedure C: Asymmetric Pd-Catalyzed Decarboxylative Alkene Difunctionalization

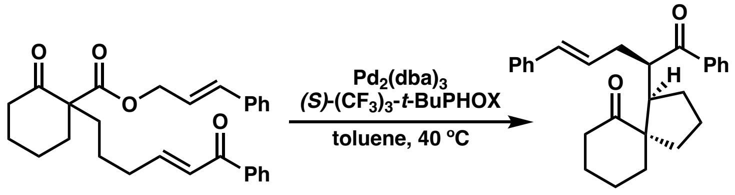

11 **12**

In a nitrogen filled glovebox, an oven-dried 4 mL vial was charged with a stir bar, $Pd_2(dba)_3$ (2.3 mg, 0.0025 mmol, 2.5 mol %), *(S)*-$(CF_3)_3$-*t*-BuPHOX (3.8 mg, 0.0065 mmol, 6.5 mol %), and toluene (0.6 mL). The catalyst solution was stirred at 23 ºC for 20 min. A solution of substrate **11** (0.1 mmol, 1 equiv) in toluene (0.4 mL) was added to the catalyst solution. The resulting mixture was sealed, pumped out of the glovebox, and then heated to 40 ºC for 16 h. The solution was then cooled to 23 ºC and concentrated under reduced pressure. The crude reaction mixture was purified via preparative TLC to yield product **12**.

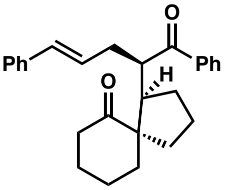

12a

(1*R*,5*S*)-1-((*R*,*E*)-1-oxo-1,5-diphenylpent-4-en-2-yl)spiro[4.5]decan-6-one (12a)
Prepared from **11a** following General Procedure C. Purification by preparative TLC (10% Et_2O, 45% toluene, 4% hexanes) afforded the title compound as a white crystalline solid (16.9 mg, 0.044 mmol, 44% yield, 94% ee). Absolute and relative stereochemistry were assigned by X-ray crystallography (vide infra).

^{1}H NMR (600 MHz, $CDCl_3$): δ 7.93 (dd, *J* = 7.6, 1.6 Hz, 2H), 7.55 – 7.50 (m, 1H), 7.44 (t, *J* = 7.7 Hz, 2H), 7.24 – 7.10 (m, 5H), 6.31 (d, *J* = 15.7 Hz, 1H), 6.02 (dt, *J* = 15.2, 7.3 Hz, 1H), 3.55 (ddd, *J* = 9.2, 7.5, 3.6 Hz, 1H), 3.14 (dt, *J* = 10.4, 7.5 Hz, 1H), 2.76 (dddd, *J* = 14.3, 9.1, 7.1, 1.4 Hz, 1H), 2.53 – 2.44 (m, 1H), 2.40 – 2.24 (m, 2H), 2.07 – 1.95 (m, 1H), 1.87 – 1.79 (m, 1H), 1.79 – 1.72 (m, 3H), 1.72 – 1.47 (m, 6H), 1.37 – 1.27 (m, 1H).

^{13}C NMR (100 MHz, CDCl$_3$): δ 213.2, 203.6, 138.0, 137.5, 132.8, 131.9, 128.7, 128.7, 128.5, 127.6, 127.2, 126.2, 58.3, 47.4, 44.2, 39.4, 38.5, 34.6, 33.0, 28.7, 25.4, 22.7, 21.6.

IR (Neat Film, NaCl): 2932, 2858, 1697, 1681, 1596, 1493, 1446, 1221 cm^{-1}.

HRMS (ESI+): *m/z* calc'd for $C_{27}H_{31}O_2$ [M+H]$^+$: 387.2319, found 387.2319.

Optical Rotation: $[\alpha]_D^{23}$ –10.7 (c = 0.53, CHCl$_3$).

SFC conditions: 30% IPA, 2.5 mL/min, Chiralpak AD-3 column, λ = 254 nm, t_R (min): major = 5.07, minor = 6.14

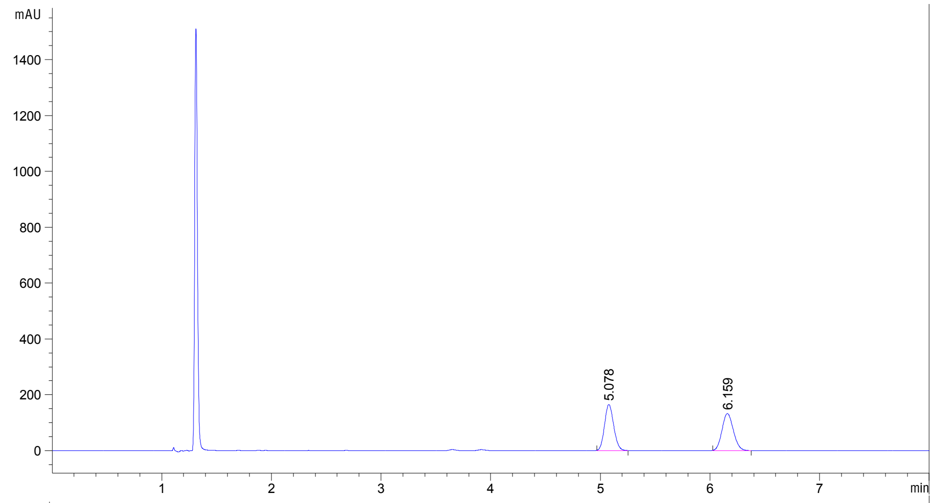

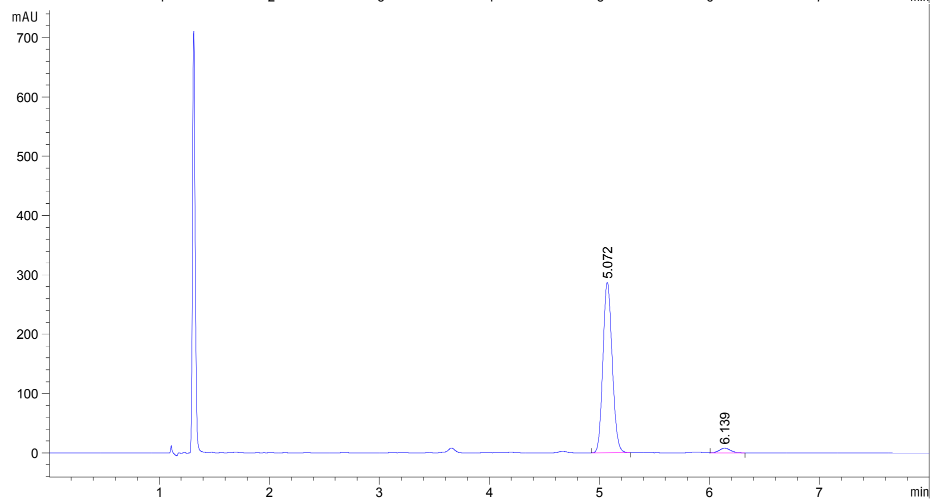

Peak #	RetTime [min]	Type	Width [min]	Area [mAU*s]	Height [mAU]	Area %
1	5.072	BB	0.0912	1678.95935	287.05362	96.8172
2	6.139	VB	0.1061	55.19526	7.83916	3.1828

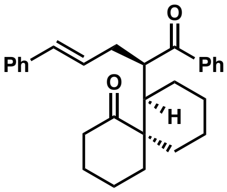

12b

(6*R*,7*R*)-7-((*R*,*E*)-1-oxo-1,5-diphenylpent-4-en-2-yl)spiro[5.5]undecan-1-one (12b)
Prepared from **11b** following General Procedure C. Purification by preparative TLC (5% Et_2O, 47.5% toluene, 47.5% hexanes) afforded the title compound as a colorless oil (13.3 mg, 0.033 mmol, 33% yield, 94% ee).

^{1}H NMR (400 MHz, $CDCl_3$): δ 7.96 – 7.89 (m, 2H), 7.56 – 7.48 (m, 1H), 7.46 – 7.39 (m, 2H), 7.23 – 7.16 (m, 2H), 7.15 – 7.05 (m, 3H), 6.30 (dd, *J* = 15.8, 1.6 Hz, 1H), 5.93 (dt, *J* = 15.7, 7.2 Hz, 1H), 3.48 (ddd, *J* = 10.9, 3.4, 2.3 Hz, 1H), 2.70 (dddd, *J* = 14.1, 10.9, 7.1, 1.4 Hz, 1H), 2.56 – 2.39 (m, 3H), 2.34 – 2.16 (m, 2H), 2.11 – 1.90 (m, 3H), 1.82 – 1.62 (m, 5H), 1.55 – 1.36 (m, 3H), 1.31 – 1.20 (m, 1H), 1.13 (td, *J* = 12.6, 4.9 Hz, 1H).

^{13}C NMR (100 MHz, $CDCl_3$): δ 215.2, 205.1, 138.1, 137.5, 132.7, 131.4, 128.9, 128.6, 128.5, 128.4, 127.1, 126.1, 54.1, 47.3, 40.7, 38.5, 33.8, 33.5, 28.5, 26.1, 26.0, 22.8, 21.1, 20.3.

IR (Neat Film, NaCl): 2929, 2855, 1698, 1673, 1596, 1446, 1222 cm^{-1}.

HRMS (ESI+): *m/z* calc'd for $C_{28}H_{33}O_2$ $[M+H]^+$: 401.2475, found 401.2471.

Optical Rotation: $[\alpha]_D^{23}$ –18.2 (c = 0.23, $CHCl_3$).

SFC conditions: 30% IPA, 2.5 mL/min, Chiralpak AD-3 column, λ = 254 nm, t_R (min): minor = 5.29, minor = 6.18

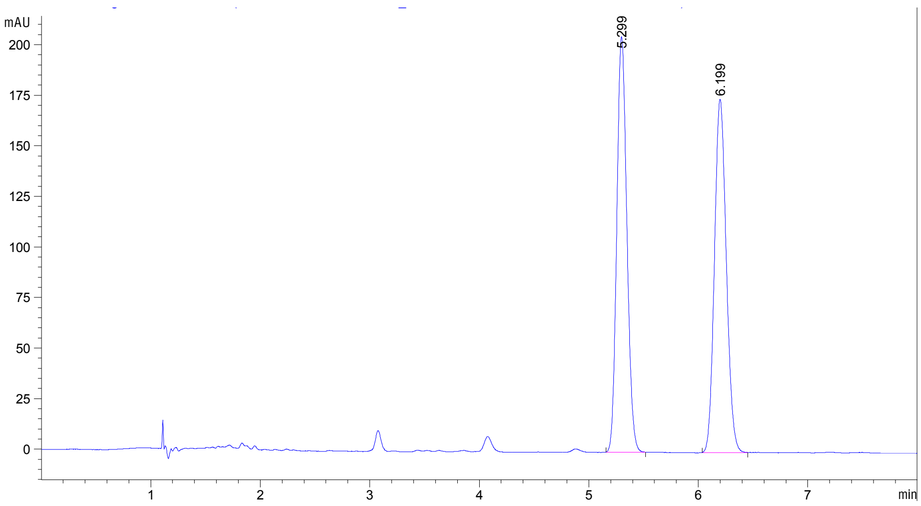

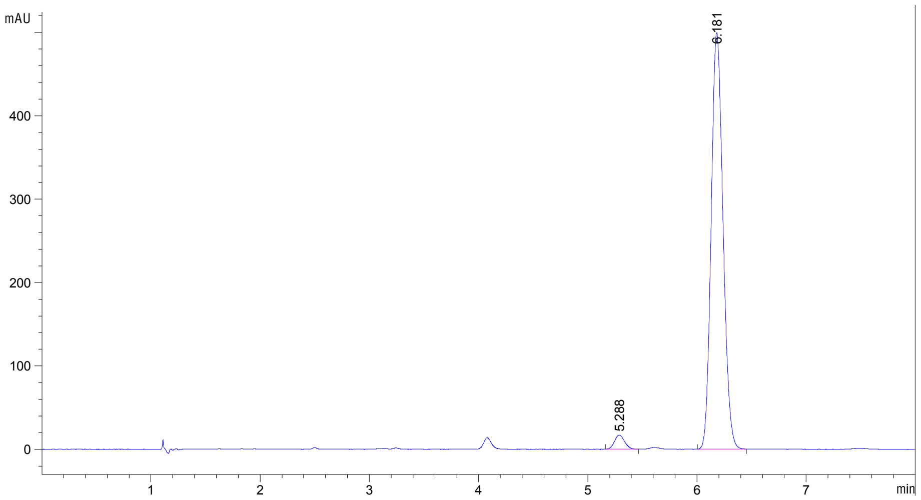

Peak #	RetTime [min]	Type	Width [min]	Area [mAU*s]	Height [mAU]	Area %
1	5.288	BB	0.1009	110.38866	17.08781	2.8547
2	6.181	BB	0.1168	3756.49658	499.33173	97.1453

Preparation of Enone Starting Materials:

General Procedure D: Horner–Wadsworth–Emmons Olefination

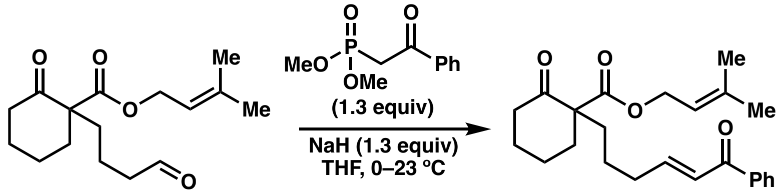

S3 **7**

To a suspension of NaH (60% by weight in mineral oil, 1.3 equiv) in THF (0.65 M) at 23 ºC was added dropwise a solution of the corresponding phosphonate ester (1.3 equiv) in THF (1.3 M). *Note: Caution, hydrogen evolution occurs.* This is stirred at 23 ºC for 30 minutes before cooling to 0 ºC. To this reaction was then added a solution of aldehyde **S3** (1.0 equiv) in THF (0.35 M) dropwise. The reaction is gradually warmed to 23 ºC. Upon complete consumption of starting material by TLC, the reaction mixture was cooled to 0 ºC, diluted with a saturated solution of $NaHCO_3$, and extracted with EtOAc (3x). The combined organic layers were dried over Na_2SO_4, filtered, and concentrated under reduced pressure. Product **7** was purified by silica gel flash column chromatography.

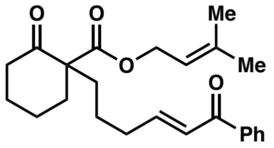

7a

3-methylbut-2-en-1-yl (*E*)-2-oxo-1-(6-oxo-6-phenylhex-4-en-1-yl)cyclohexane-1-carboxylate (7a)

Prepared from **S3a** and dimethyl (2-oxo-2-phenylethyl)phosphonate[6] following General Procedure D. Purification by flash column chromatography (0–50% EtOAc/hexanes) afforded the title compound as a colorless oil (1.41 g, 3.68 mmol, 64% yield).

^{1}H NMR (400 MHz, CDCl$_3$): δ 7.96 – 7.89 (m, 2H), 7.58 – 7.51 (m, 1H), 7.50 – 7.42 (m, 2H), 7.01 (dt, *J* = 15.5, 6.7 Hz, 1H), 6.88 (d, *J* = 15.4 Hz, 1H), 5.31 (tt, *J* = 7.3, 1.4 Hz, 1H), 4.70 – 4.55 (m, 2H), 2.57 – 2.37 (m, 3H), 2.37 – 2.22 (m, 2H), 2.01 (ddq, *J* = 12.4, 6.3, 3.2 Hz, 1H), 1.91 (td, *J* = 12.2, 3.1 Hz, 1H), 1.71 (dd, *J* = 12.7, 1.3 Hz, 7H), 1.67 – 1.48 (m, 4H), 1.48 – 1.32 (m, 2H).

^{13}C NMR (100 MHz, CDCl$_3$): δ 208.1, 191.0, 172.1, 149.2, 140.1, 138.1, 132.7, 128.7, 128.6, 126.3, 118.1, 62.2, 60.9, 41.3, 36.6, 34.6, 33.2, 27.8, 25.8, 23.1, 22.8, 18.2.

IR (Neat Film, NaCl): 2937, 2864, 1712, 1670, 1620, 1447, 1340, 1289, 1208, 1173, 1135 cm^{-1}.

HRMS (ESI+): *m/z* calc'd for $C_{24}H_{30}O_4Na$ [M+Na]$^+$: 405.2036, found 405.2045.

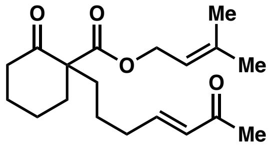

7b

3-methylbut-2-en-1-yl (*E*)-2-oxo-1-(6-oxohept-4-en-1-yl)cyclohexane-1-carboxylate (7b)
Prepared from **S3a** and dimethyl acetylmethylphosphonate following General Procedure D. Purification by flash column chromatography (0–50% EtOAc/hexanes) afforded the title compound as a colorless oil (406 mg, 1.27 mmol, 71% yield).

^{1}H NMR (400 MHz, CDCl$_3$): δ 6.76 (dt, *J* = 16.0, 6.8 Hz, 1H), 6.06 (dt, *J* = 16.0, 1.6 Hz, 1H), 5.37 – 5.27 (m, 1H), 4.69 – 4.57 (m, 2H), 2.55 – 2.37 (m, 3H), 2.27 – 2.17 (m, 4H), 2.06 – 1.95 (m, 1H), 1.86 (td, *J* = 12.6, 3.6 Hz, 1H), 1.79 – 1.72 (m, 4H), 1.70 (s, 3H), 1.69 – 1.27 (m, 7H).

^{13}C NMR (100 MHz, CDCl$_3$): δ 208.1, 198.8, 172.1, 147.8, 140.2, 131.7, 118.1, 62.2, 60.9, 41.3, 36.6, 34.5, 32.8, 27.8, 27.0, 25.9, 23.1, 22.8, 18.2.

IR (Neat Film, NaCl): 2937, 2865, 1708, 1675, 1625, 1438, 1362, 1253, 1207, 1173, 1135 cm^{-1}.

HRMS (ESI+): *m/z* calc'd for $C_{19}H_{28}O_4Na$ [M+Na]$^+$: 343.1880, found 343.1884.

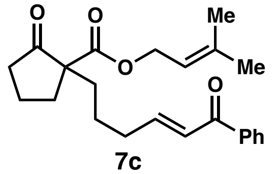

7c

3-methylbut-2-en-1-yl (*E*)-2-oxo-1-(6-oxo-6-phenylhex-4-en-1-yl)cyclopentane-1-carboxylate (7c)
Prepared from **S3b** and dimethyl (2-oxo-2-phenylethyl)phosphonate[6] following General Procedure D. Purification by flash column chromatography (0–50% EtOAc/hexanes) afforded the title compound as a colorless oil (631 mg, 2.37 mmol, 47% yield).

^{1}H NMR (400 MHz, CDCl$_3$): δ 8.00 – 7.84 (m, 2H), 7.60 – 7.51 (m, 1H), 7.51 – 7.41 (m, 2H), 7.01 (dt, J = 15.4, 6.7 Hz, 1H), 6.88 (dt, J = 15.4, 1.4 Hz, 1H), 5.33 – 5.23 (m, 1H), 4.60 (d, J = 7.1 Hz, 2H), 2.58 – 2.49 (m, 1H), 2.48 – 2.37 (m, 1H), 2.37 – 2.17 (m, 3H), 2.09 – 1.83 (m, 4H), 1.73 (s, 3H), 1.70 – 1.57 (m, 5H), 1.52 – 1.38 (m, 1H).

^{13}C NMR (100 MHz, CDCl$_3$): δ 214.9, 190.9, 171.1, 148.9, 139.7, 138.0, 132.8, 128.7, 128.7, 126.4, 118.3, 62.5, 60.5, 38.1, 33.6, 33.2, 33.1, 25.9, 23.6, 19.8, 18.2.

IR (Neat Film, NaCl): 2961, 1749, 1718, 1670, 1618, 1447, 1379, 1284, 1224, 1159 cm^{-1}.

HRMS (ESI+): *m/z* calc'd for $C_{23}H_{28}O_4Na$ [M+Na]$^+$: 391.1880, found 391.1879.

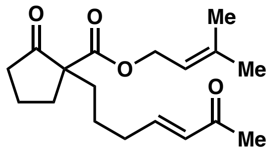

7d

3-methylbut-2-en-1-yl (*E*)-2-oxo-1-(6-oxohept-4-en-1-yl)cyclopentane-1-carboxylate (7d)
Prepared from **S3b** and dimethyl acetylmethylphosphonate following General Procedure D. Purification by flash column chromatography (0–50% EtOAc/hexanes) afforded the title compound as a colorless oil (120 mg, 0.390 mmol, 52% yield).

^{1}H NMR (400 MHz, CDCl$_3$): δ 6.75 (dt, J = 15.9, 6.8 Hz, 1H), 6.06 (dt, J = 16.1, 1.6 Hz, 1H), 5.34 – 5.24 (m, 1H), 4.59 (d, J = 7.1 Hz, 2H), 2.57 – 2.36 (m, 2H), 2.30 – 2.15 (m, 6H), 2.08 – 1.81 (m, 4H), 1.74 (s, 3H), 1.69 (s, 3H), 1.64 – 1.57 (m, 1H), 1.56 – 1.48 (m, 1H), 1.39 (qdd, J = 11.9, 8.2, 4.5 Hz, 1H).

^{13}C NMR (100 MHz, CDCl$_3$): δ 214.9, 198.7, 171.1, 147.4, 139.7, 131.8, 118.2, 62.5, 60.4, 38.1, 33.5, 33.2, 32.7, 27.1, 25.9, 23.6, 19.8, 18.2.

IR (Neat Film, NaCl): 2962, 1750, 1718, 1675, 1626, 1448, 1362, 1253, 1225, 1161 cm^{-1}.

HRMS (ESI+): *m/z* calc'd for $C_{18}H_{26}O_4Na$ [M+Na]$^+$: 329.1723, found 329.1722.

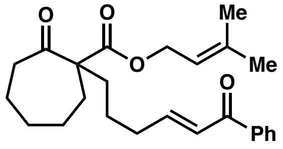

7e

3-methylbut-2-en-1-yl (*E*)-2-oxo-1-(6-oxo-6-phenylhex-4-en-1-yl)cycloheptane-1-carboxylate (7e)
Prepared from **S3c** and dimethyl (2-oxo-2-phenylethyl)phosphonate[6] following General Procedure D. Purification by flash column chromatography (0–50% EtOAc/hexanes) afforded the title compound as a colorless oil (494 mg, 1.24 mmol, 73% yield).

^{1}H NMR (400 MHz, CDCl$_3$): δ 7.97 – 7.87 (m, 2H), 7.59 – 7.52 (m, 1H), 7.51 – 7.42 (m, 2H), 7.01 (dt, J = 15.5, 6.7 Hz, 1H), 6.88 (dt, J = 15.4, 1.3 Hz, 1H), 5.38 – 5.23 (m, 1H), 4.71 – 4.54

(m, 2H), 2.71 – 2.59 (m, 1H), 2.50 – 2.41 (m, 1H), 2.38 – 2.23 (m, 2H), 2.21 – 2.10 (m, 1H), 2.08 – 1.96 (m, 1H), 1.80 – 1.71 (m, 5H), 1.71 – 1.58 (m, 7H), 1.56 – 1.37 (m, 4H).

^{13}C NMR (100 MHz, CDCl$_3$): δ 209.6, 191.0, 172.6, 149.2, 139.9, 138.1, 132.8, 128.7, 128.7, 126.4, 118.2, 62.9, 62.1, 42.3, 35.3, 33.2, 33.2, 29.9, 25.8, 25.6, 25.0, 23.5, 18.2.

IR (Neat Film, NaCl): 2932, 2860, 1707, 1670, 1619, 1447, 1377, 1344, 1286, 1222, 1165 cm^{-1}.

HRMS (ESI+): *m/z* calc'd for $C_{25}H_{32}O_4Na$ [M+Na]$^+$: 419.2193, found 419.2194.

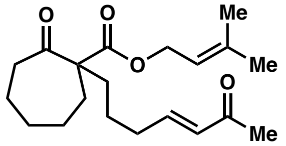

7f

3-methylbut-2-en-1-yl (*E*)-2-oxo-1-(6-oxohept-4-en-1-yl)cycloheptane-1-carboxylate (7f)
Prepared from **S3c** and dimethyl acetylmethylphosphonate following General Procedure D. Purification by flash column chromatography (0–50% EtOAc/hexanes) afforded the title compound as a colorless oil (409 mg, 1.22 mmol, 72% yield).

^{1}H NMR (400 MHz, CDCl$_3$): δ 6.76 (dt, *J* = 16.0, 6.8 Hz, 1H), 6.06 (dt, *J* = 16.0, 1.5 Hz, 1H), 5.38 – 5.24 (m, 1H), 4.69 – 4.53 (m, 2H), 2.74 – 2.54 (m, 1H), 2.52 – 2.38 (m, 1H), 2.30 – 2.06 (m, 6H), 2.05 – 1.90 (m, 1H), 1.83 – 1.72 (m, 4H), 1.70 (d, *J* = 1.4 Hz, 4H), 1.66 – 1.49 (m, 5H), 1.49 – 1.31 (m, 3H).

^{13}C NMR (100 MHz, CDCl$_3$): δ 209.6, 198.8, 172.5, 147.7, 139.9, 131.7, 118.2, 62.8, 62.1, 42.3, 35.2, 33.2, 32.9, 29.9, 27.0, 25.8, 25.6, 25.0, 23.5, 18.2.

IR (Neat Film, NaCl): 2930, 2862, 1707, 1675, 1626, 1445, 1359, 1253, 1219, 1166 cm^{-1}.

HRMS (ESI+): *m/z* calc'd for $C_{20}H_{30}O_4Na$ [M+Na]$^+$: 357.2036, found 357.2041.

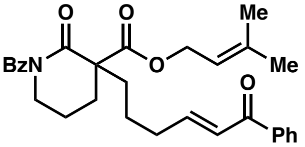

7g

3-methylbut-2-en-1-yl (*E*)-1-benzoyl-2-oxo-3-(6-oxo-6-phenylhex-4-en-1-yl)piperidine-3-carboxylate (7g)
Prepared from **S3d** and dimethyl (2-oxo-2-phenylethyl)phosphonate[6] following General Procedure D. Purification by flash column chromatography (0–50% EtOAc/hexanes) afforded the title compound as a colorless oil (317 mg, 0.650 mmol, 61% yield).

^{1}H NMR (400 MHz, CDCl$_3$): δ 7.96 – 7.84 (m, 2H), 7.78 – 7.70 (m, 2H), 7.59 – 7.51 (m, 1H), 7.50 – 7.40 (m, 3H), 7.40 – 7.33 (m, 2H), 6.97 (dt, *J* = 15.5, 6.6 Hz, 1H), 6.85 (dt, *J* = 15.4, 1.4 Hz, 1H), 5.49 – 5.35 (m, 1H), 4.81 – 4.64 (m, 2H), 3.90 – 3.70 (m, 2H), 2.51 – 2.39 (m, 1H), 2.28

(q, J = 7.2 Hz, 2H), 2.04 – 1.82 (m, 5H), 1.79 (s, 3H), 1.75 (s, 3H), 1.70 – 1.60 (m, 1H), 1.51 – 1.37 (m, 1H).

^{13}C NMR (100 MHz, $CDCl_3$): δ 190.9, 175.1, 172.3, 172.2, 148.8, 140.7, 138.0, 136.0, 132.8, 131.8, 128.7, 128.6, 128.3, 128.2, 126.4, 117.8, 63.0, 56.7, 46.6, 35.4, 33.0, 30.9, 26.0, 23.5, 20.5, 18.3.

IR (Neat Film, NaCl): 2937, 1724, 1701, 1676, 1618, 1447, 1386, 1348, 1277, 1189, 1166, 1149 cm^{-1}.

HRMS (ESI+): *m/z* calc'd for $C_{30}H_{33}NO_5Na$ $[M+Na]^+$: 510.2251, found 510.2264.

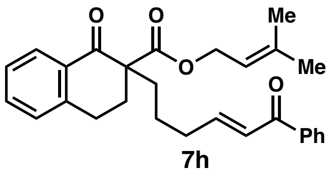

7h

3-methylbut-2-en-1-yl (*E*)-1-oxo-2-(6-oxo-6-phenylhex-4-en-1-yl)-1,2,3,4-tetrahydronaphthalene-2-carboxylate (7h)

Prepared from **S3e** and dimethyl (2-oxo-2-phenylethyl)phosphonate[6] following General Procedure D. Purification by flash column chromatography (0–50% EtOAc/hexanes) afforded the title compound as a colorless oil (248 mg, 0.576 mmol, 58% yield).

^{1}H NMR (400 MHz, $CDCl_3$): δ 8.04 (dd, J = 7.9, 1.4 Hz, 1H), 7.97 – 7.88 (m, 2H), 7.59 – 7.51 (m, 1H), 7.50 – 7.42 (m, 3H), 7.31 (t, J = 7.6 Hz, 1H), 7.20 (d, J = 7.7 Hz, 1H), 7.04 (dt, J = 15.5, 6.6 Hz, 1H), 6.91 (dt, J = 15.3, 1.4 Hz, 1H), 5.26 – 5.14 (m, 1H), 4.63 – 4.49 (m, 2H), 3.07 (ddd, J = 15.5, 10.4, 4.7 Hz, 1H), 2.91 (dt, J = 17.4, 4.9 Hz, 1H), 2.56 (dt, J = 13.7, 4.9 Hz, 1H), 2.36 (q, J = 7.5 Hz, 2H), 2.22 – 2.09 (m, 1H), 2.07 – 1.88 (m, 2H), 1.80 – 1.64 (m, 4H), 1.63 – 1.56 (m, 4H).

^{13}C NMR (100 MHz, $CDCl_3$): δ 195.6, 191.0, 172.0, 149.1, 143.1, 139.9, 138.1, 133.5, 132.8, 132.3, 128.8, 128.7, 128.7, 128.2, 126.9, 126.4, 118.2, 62.3, 57.6, 33.9, 33.3, 31.1, 26.1, 25.8, 23.5, 18.1.

IR (Neat Film, NaCl): 2931, 1725, 1685, 1670, 1618, 1599, 1447, 1351,1292,1230, 1174 cm^{-1}.

HRMS (ESI+): *m/z* calc'd for $C_{28}H_{30}O_4Na$ $[M+Na]^+$: 453.2036, found 453.2035.

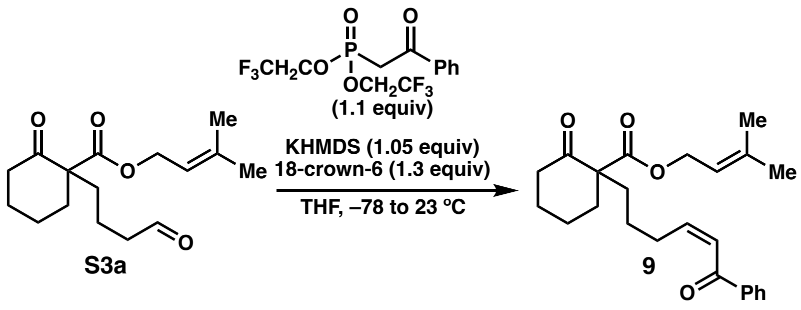

3-methylbut-2-en-1-yl (*Z*)-2-oxo-1-(6-oxo-6-phenylhex-4-en-1-yl)cyclohexane-1-carboxylate (9)

An oven dried 100 mL round bottom flask was charged with 18-crown-6 (610 mg, 2.31 mmol, 1.3 equiv). To this a solution of bis(2,2,2-trifluoroethyl) (2-oxo-2-phenylethyl)phosphonate[7] (714 mg, 1.96 mmol, 1.1 equiv) in THF (10 mL, 0.2 M) was added. This was cooled to –78 °C for 10 minutes before a solution of KHMDS (373 mg, 1.87 mmol, 1.05 equiv) in THF (2 mL, 0.9 M) was added dropwise. The solution was stirred for 20 minutes at –78 °C before a solution of **S3a** (500 mg, 1.78 mmol, 1.0 equiv) in THF (10 mL, 0.18 M) was added dropwise. The reaction was warmed gradually to 23 °C. Upon complete consumption of starting material by TLC, the reaction mixture was cooled to 0 °C, diluted with a saturated solution of NH_4Cl (20 mL), and extracted with Et_2O (3x). The combined organic layers were dried over Na_2SO_4, filtered, and concentrated under reduced pressure. The crude reaction mixture was purified by silica gel flash column chromatography (15% Et_2O/hexanes) to afford **9** as a colorless oil (101 mg, 0.264 mmol, 15% yield).

^{1}H NMR (400 MHz, $CDCl_3$): δ 7.91 – 7.84 (m, 2H), 7.13 – 7.02 (m, 3H), 6.53 (dt, J = 11.5, 1.7 Hz, 1H), 6.05 (dt, J = 11.6, 7.4 Hz, 1H), 5.31 (tdt, J = 7.2, 2.9, 1.4 Hz, 1H), 4.55 (d, J = 7.2 Hz, 2H), 2.82 – 2.65 (m, 2H), 2.48 – 2.26 (m, 3H), 2.14 – 2.01 (m, 1H), 1.75 – 1.46 (m, 8H), 1.44 (s, 3H), 1.37 – 1.06 (m, 3H).

^{13}C NMR (100 MHz, C_6D_6): δ 206.2, 191.0, 172.1, 148.7, 139.4, 139.2, 132.4, 128.6, 128.6, 124.8, 119.0, 61.8, 61.1, 41.2, 36.4, 34.9, 30.5, 27.7, 25.6, 24.6, 22.9, 17.9.

*Note: It was noted that upon standing in basified (K_2CO_3) or neutral deuterochloroform, Z-enone **9** isomerizes to 7**a**.*

IR (Neat Film, NaCl): 2938, 2865, 1711, 1664, 1610, 1448, 1227, 1174, 1134 cm^{-1}.

HRMS (ESI+): *m/z* calc'd for $C_{24}H_{34}NO_4$ $[M+NH_4]^+$: 400.2482, found 400.2490.

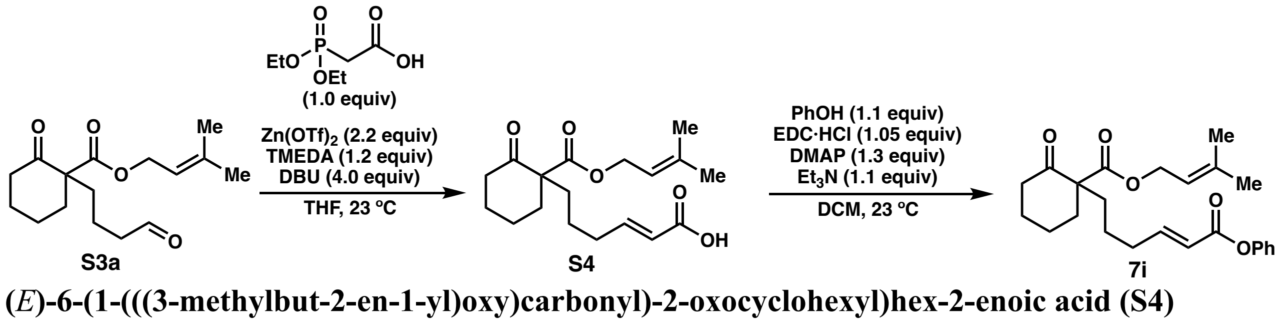

(*E*)-6-(1-(((3-methylbut-2-en-1-yl)oxy)carbonyl)-2-oxocyclohexyl)hex-2-enoic acid (S4)

To a flame dried 50 mL round bottom flask was added zinc triflate (2.09 g, 5.74 mmol, 2.2 equiv) followed by THF (9.5 mL, 0.6 M). 2-(diethoxyphosphoryl)acetic acid was then added (0.42 mL, 2.61 mmol, 1.0 equiv) followed by TMEDA (0.47 mL, 3.13 mmol, 1.2 equiv) and DBU (1.56 mL, 10.4 mmol, 4.0 equiv). To this a solution of **S3a** (805 mg, 2.87 mmol, 1.1 equiv) in THF (6.6 mL, 0.43 M) was added dropwise. Upon complete consumption of starting material by TLC, the reaction was diluted with 1 M HCl (27 mL), extracted with CH_2Cl_2 (3x). The combined organic layers were dried over Na_2SO_4, filtered, and concentrated under reduced pressure. The crude

reaction mixture was purified by silica gel flash column chromatography (0–50% EtOAc/hexanes) to afford **S4** as an amorphous white solid (610 mg, 1.89 mmol, 72% yield).

^{1}H NMR (400 MHz, $CDCl_3$): δ 7.06 (dt, *J* = 15.6, 6.8 Hz, 1H), 5.84 (dt, *J* = 15.7, 1.6 Hz, 1H), 5.38 – 5.30 (m, 1H), 4.66 (d, *J* = 7.3 Hz, 2H), 2.59 – 2.40 (m, 3H), 2.33 – 2.18 (m, 2H), 2.08 – 1.97 (m, 1H), 1.90 (td, *J* = 12.5, 3.5 Hz, 1H), 1.83 – 1.72 (m, 7H), 1.72 – 1.23 (m, 6H).

*Note: OH proton not observed for **S4** in ^{1}H NMR.*

^{13}C NMR (100 MHz, $CDCl_3$): δ 208.1, 172.1, 171.2, 151.6, 140.2, 120.9, 118.1, 62.2, 60.9, 41.3, 36.6, 34.5, 32.6, 27.8, 25.8, 22.9, 22.8, 18.2.

IR (Neat Film, NaCl): 2937, 2865, 1711, 1653, 1437, 1275, 1208, 1168 cm^{-1}.

HRMS (ESI+): *m/z* calc'd for $C_{18}H_{25}O_5$ $[M–H]^-$: 321.1707, found 321.1710.

3-methylbut-2-en-1-yl (*E*)-2-oxo-1-(6-oxo-6-phenoxyhex-4-en-1-yl)cyclohexane-1-carboxylate (7i)

To a flame dried scint vial was added **S4** (220 mg, 0.68 mmol, 1.0 equiv), phenol (70.6 mg, 0.75 mmol, 1.1 equiv), EDC·HCl (144 mg, 0.75 mmol, 1.1 equiv), followed by CH_2Cl_2 (3.4 mL, 0.2 M). Following this, Et_3N (0.10 mL, 0.75 mmol, 1.1 equiv) and DMAP (8.3 mg, 0.068 mmol, 0.1 equiv) were added successively. Upon complete consumption of starting material by TLC, the reaction was diluted with CH_2Cl_2, followed by a 1 M HCl (4 mL) rinse, and sat. $NaHCO_3$ solution (4 mL) rinse, and then rinsed with brine. The combined organic layers were dried over Na_2SO_4, filtered, and concentrated under reduced pressure. The crude reaction mixture was purified by silica gel flash column chromatography (0–35% Et_2O/hexanes) to afford **7i** as an amorphous white solid (144 mg, 0.36 mmol, 53% yield).

^{1}H NMR (400 MHz, $CDCl_3$): δ 7.42 – 7.34 (m, 2H), 7.25 – 7.19 (m, 1H), 7.17 – 7.07 (m, 3H), 6.02 (dt, *J* = 15.7, 1.6 Hz, 1H), 5.37 – 5.29 (m, 1H), 4.65 (d, *J* = 6.7 Hz, 2H), 2.58 – 2.38 (m, 3H), 2.34 – 2.20 (m, 2H), 2.07 – 1.97 (m, 1H), 1.91 (td, *J* = 12.5, 3.4 Hz, 1H), 1.80 – 1.71 (m, 7H), 1.70 – 1.57 (m, 3H), 1.55 – 1.32 (m, 3H).

^{13}C NMR (100 MHz, $CDCl_3$): δ 208.1, 172.1, 165.1, 151.1, 150.9, 140.2, 129.5, 125.8, 121.8, 121.0, 118.1, 62.2, 60.9, 41.3, 36.6, 34.5, 32.8, 27.8, 25.8, 23.0, 22.8, 18.2.

IR (Neat Film, NaCl): 2938, 2864, 1734, 1711, 1651, 1593, 1491, 1450, 1310, 1243, 1197 cm^{-1}.

HRMS (ESI+): *m/z* calc'd for $C_{24}H_{34}NO_5$ $[M+NH_4]^+$: 416.2431, found 416.2433.

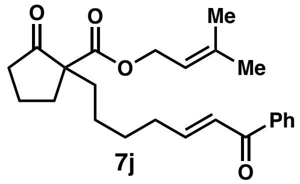

7j

3-methylbut-2-en-1-yl (*E*)-2-oxo-1-(7-oxo-7-phenylhept-5-en-1-yl)cyclopentane-1-carboxylate (7j)
Prepared from **S3f** and dimethyl (2-oxo-2-phenylethyl)phosphonate[6] following General Procedure D. Purification by flash column chromatography (0–50% EtOAc/hexanes) afforded the title compound as a colorless oil (392 mg, 1.03 mmol, 58% yield).

^{1}H NMR (400 MHz, CDCl$_3$): δ 7.96 – 7.88 (m, 2H), 7.59 – 7.53 (m, 1H), 7.51 – 7.41 (m, 2H), 7.03 (dt, *J* = 15.4, 6.8 Hz, 1H), 6.87 (dt, *J* = 15.4, 1.4 Hz, 1H), 5.34 – 5.22 (m, 1H), 4.58 (dt, *J* = 7.2, 1.0 Hz, 2H), 2.57 – 2.48 (m, 1H), 2.47 – 2.37 (m, 1H), 2.36 – 2.16 (m, 3H), 2.07 – 1.82 (m, 4H), 1.73 (s, 3H), 1.68 (s, 3H), 1.64 – 1.47 (m, 3H), 1.46 – 1.34 (m, 1H), 1.33 – 1.20 (m, 1H).

^{13}C NMR (100 MHz, CDCl$_3$): δ 215.1, 191.0, 171.2, 149.5, 139.5, 138.1, 132.8, 128.7, 128.7, 126.2, 118.3, 62.5, 60.6, 38.1, 33.7, 33.0, 32.6, 28.6, 25.9, 24.6, 19.8, 18.2.

IR (Neat Film, NaCl): 2930, 2859, 1750, 1719, 1670, 1618, 1597, 1447, 1335, 1278, 1223, 1166 cm^{-1}.

HRMS (ESI+): *m/z* calc'd for $C_{24}H_{30}O_4Na$ [M+Na]$^+$: 405.2036, found 405.2043.

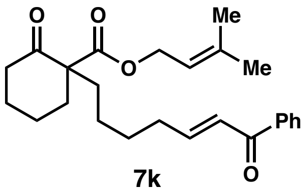

7k

3-methylbut-2-en-1-yl (*E*)-2-oxo-1-(7-oxo-7-phenylhept-5-en-1-yl)cyclohexane-1-carboxylate (7k)
Prepared from **S3g** and dimethyl (2-oxo-2-phenylethyl)phosphonate[6] following General Procedure D. Purification by flash column chromatography (0–50% EtOAc/hexanes) afforded the title compound as a colorless oil (273 mg, 0.69 mmol, 69% yield).

^{1}H NMR (400 MHz, CDCl$_3$): δ 7.95 – 7.89 (m, 2H), 7.59 – 7.52 (m, 1H), 7.50 – 7.43 (m, 2H), 7.03 (dt, *J* = 15.4, 6.8 Hz, 1H), 6.87 (dt, *J* = 15.4, 1.4 Hz, 1H), 5.34 – 5.26 (m, 1H), 4.67 – 4.55 (m, 2H), 2.56 – 2.37 (m, 3H), 2.36 – 2.23 (m, 2H), 2.07 – 1.95 (m, 1H), 1.94 – 1.80 (m, 1H), 1.77 – 1.68 (m, 7H), 1.67 – 1.59 (m, 2H), 1.57 – 1.47 (m, 3H), 1.46 – 1.37 (m, 1H), 1.36 – 1.21 (m, 2H).

^{13}C NMR (100 MHz, CDCl$_3$): δ 208.2, 191.1, 172.2, 149.7, 140.0, 138.1, 132.7, 128.69, 128.65, 126.2, 118.2, 62.1, 61.0, 41.3, 36.4, 34.7, 32.7, 28.7, 27.8, 25.8, 24.1, 22.8, 18.2.

IR (Neat Film, NaCl): 2929, 2854, 1728, 1683, 1596, 1447, 1291, 1209, 1185, 1160 cm^{-1}.

HRMS (ESI+): *m/z* calc'd for $C_{25}H_{32}O_4Na$ [M+Na]$^+$: 419.2193, found 419.2201.

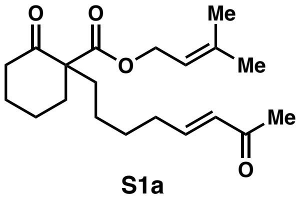

S1a

3-methylbut-2-en-1-yl (*E*)-2-oxo-1-(7-oxooct-5-en-1-yl)cyclohexane-1-carboxylate (S1a)
Prepared from **S3g** and acetylmethylphosphonate following General Procedure D. Purification by flash column chromatography (0–50% EtOAc/hexanes) afforded the title compound as a colorless oil (222 mg, 0.66 mmol, 68% yield).

^{1}H NMR (400 MHz, $CDCl_3$): δ 6.77 (dt, *J* = 15.9, 6.9 Hz, 1H), 6.05 (dt, *J* = 16.0, 1.5 Hz, 1H), 5.35 – 5.26 (m, 1H), 4.69 – 4.55 (m, 2H), 2.54 – 2.35 (m, 3H), 2.26 – 2.15 (m, 5H), 2.06 – 1.95 (m, 1H), 1.85 (ddd, *J* = 13.6, 12.2, 4.7 Hz, 1H), 1.78 – 1.67 (m, 7H), 1.67 – 1.36 (m, 6H), 1.35 – 1.13 (m, 2H).

^{13}C NMR (100 MHz, $CDCl_3$): δ 208.2, 198.9, 172.2, 148.3, 140.0, 131.5, 118.1, 62.1, 61.0, 41.3, 36.4, 34.6, 32.4, 28.6, 27.8, 27.0, 25.8, 24.1, 22.8, 18.2.

IR (Neat Film, NaCl): 2938, 2863, 1709, 1674, 1626, 1449, 1361, 1309, 1253, 1208, 1170, 1135, 1100 cm^{-1}.

HRMS (ESI+): *m/z* calc'd for $C_{20}H_{30}O_4Na$ $[M+Na]^+$: 357.2036, found 357.2035.

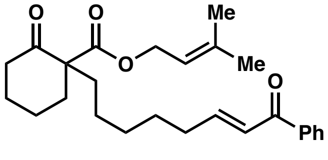

S1b

3-methylbut-2-en-1-yl (*E*)-2-oxo-1-(8-oxo-8-phenyloct-6-en-1-yl)cyclohexane-1-carboxylate (S1b)
Prepared from **S3h** and dimethyl (2-oxo-2-phenylethyl)phosphonate[6] following General Procedure D. Purification by flash column chromatography (0–50% EtOAc/hexanes) afforded the title compound as a colorless oil (493 mg, 1.20 mmol, 74% yield).

^{1}H NMR (400 MHz, $CDCl_3$): δ 7.97 – 7.89 (m, 2H), 7.59 – 7.51 (m, 1H), 7.50 – 7.42 (m, 2H), 7.04 (dt, *J* = 15.4, 6.9 Hz, 1H), 6.86 (dt, *J* = 15.4, 1.4 Hz, 1H), 5.36 – 5.25 (m, 1H), 4.68 – 4.56 (m, 2H), 2.56 – 2.36 (m, 3H), 2.33 – 2.24 (m, 2H), 2.03 – 1.93 (m, 1H), 1.86 (ddd, *J* = 13.5, 12.0, 4.5 Hz, 1H), 1.78 – 1.68 (m, 7H), 1.67 – 1.47 (m, 5H), 1.46 – 1.30 (m, 3H), 1.29 – 1.13 (m, 2H).

^{13}C NMR (100 MHz, $CDCl_3$): δ 208.3, 191.1, 172.2, 150.0, 139.9, 138.2, 132.7, 128.7, 128.6, 126.1, 118.2, 62.1, 61.0, 41.3, 36.4, 34.8, 32.9, 29.7, 28.1, 27.8, 25.9, 24.1, 22.8, 18.2.

IR (Neat Film, NaCl): 2935, 2859, 1710, 1670, 1618, 1447, 1378, 1340, 1279, 1221, 1167 cm^{-1}.

HRMS (ESI+): *m/z* calc'd for $C_{26}H_{34}O_4Na$ $[M+Na]^+$: 433.2349, found 433.2360.

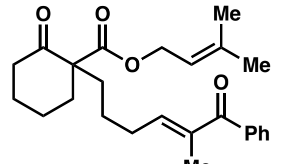

S1c

3-methylbut-2-en-1-yl (*E*)-1-(5-methyl-6-oxo-6-phenylhex-4-en-1-yl)-2-oxocyclohexane-1-carboxylate (S1c)

Prepared from **S3a** and dimethyl (1-oxo-1-phenylpropan-2-yl)phosphonate[8] following General Procedure D. Purification by flash column chromatography (20% Et_2O/hexanes) afforded the title compound as a colorless oil (72.4 mg, 0.18 mmol, 26% yield).

^{1}H NMR (400 MHz, $CDCl_3$): δ 7.64 – 7.59 (m, 2H), 7.52 – 7.46 (m, 1H), 7.44 – 7.38 (m, 2H), 6.24 (td, J = 7.3, 1.4 Hz, 1H), 5.33 – 5.23 (m, 1H), 4.68 – 4.52 (m, 2H), 2.54 – 2.35 (m, 3H), 2.31 – 2.22 (m, 2H), 2.05 – 1.93 (m, 4H), 1.93 – 1.82 (m, 1H), 1.79 – 1.66 (m, 8H), 1.66 – 1.57 (m, 2H), 1.48 – 1.36 (m, 2H), 1.35 – 1.22 (m, 1H).

^{13}C NMR (100 MHz, $CDCl_3$): δ 208.1, 199.1, 172.1, 146.1, 140.1, 138.8, 136.9, 131.5, 129.5, 128.2, 118.1, 62.2, 60.9, 41.3, 36.6, 34.8, 29.6, 27.8, 25.8, 23.7, 22.8, 18.2, 12.7.

IR (Neat Film, NaCl): 2938, 2864, 1711, 1647, 1445, 1381, 1280, 1208, 1185 cm^{-1}.

HRMS (ESI+): *m/z* calc'd for $C_{25}H_{32}O_4Na$ [M+Na]$^+$: 419.2193, found 419.2203.

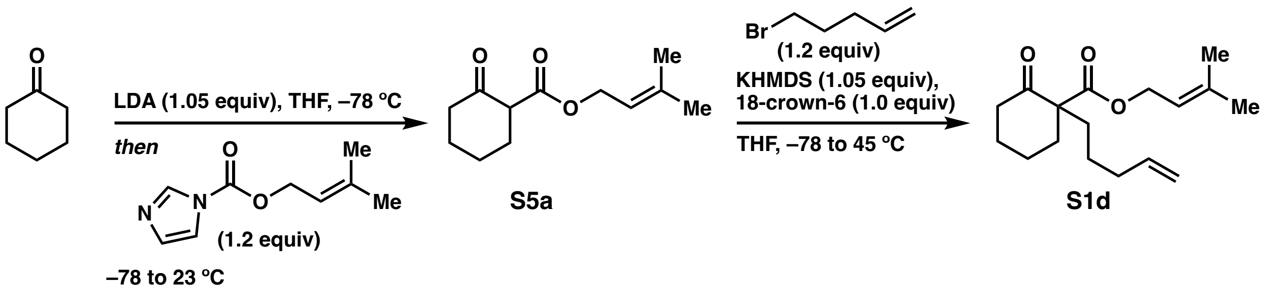

3-methylbut-2-en-1-yl 2-oxo-1-(pent-4-en-1-yl)cyclohexane-1-carboxylate (S1d)

To a flame dried 250 mL round bottom flask was added *i*Pr_2NH (4.6 mL, 33 mmol, 1.1 equiv) followed by THF (17 mL, 1.9 M) before the solution was cooled to 0 °C. To this, *n*-BuLi (2.5 M in hexanes, 12.6 mL, 31.5 mmol, 1.05 equiv) was added dropwise before the solution was allowed to stir at 0 °C for 15 minutes. To this, a solution of cyclohexanone (3.1 mL, 30 mmol, 1.0 equiv) in THF (24 mL, 1.25 M) was added dropwise before the solution was allowed to stir at 0 °C for 30 minutes. The solution was then cooled to –78 °C, and a solution of 3-methylbut-2-en-1-yl 1*H*-imidazole-1-carboxylate[9] (6.48 g, 36 mmol, 1.2 equiv) in THF (9 mL, 4 M) was added dropwise. The reaction was warmed gradually to 23 °C. Upon complete consumption of starting material by TLC, the reaction was cooled to 0 °C, the pH was adjusted to 1.0 using 1 M HCl, and the mixture was extracted with EtOAc (3x). The combined organic layers were rinsed with brine, dried over Na_2SO_4, filtered, and concentrated under reduced pressure. The crude reaction mixture was purified by silica gel flash column chromatography (0–20% EtOAc/hexanes) to afford **S5a** as a mixture of tautomers (4.18 g, 19.9 mmol, 66% yield), which was used directly in the next step.

To a flame dried 250 mL round bottom flask was added KHMDS (796 mg, 4.0 mmol, 1.05 equiv), and 18-crown-6 (1.00 g, 3.8 mmol, 1.0 equiv) followed by THF (20 mL, 0.19 M) before the solution was cooled to –78 °C. A solution of β-ketoester **S5a** (884 mg, 3.8 mmol, 1.0 equiv) in THF (10 mL, 0.38 M) was added dropwise at –78 °C. The solution was stirred at this temperature for 15 minutes before 5-bromopent-1-ene was added neat (0.54 mL, 4.6 mmol, 1.2 equiv). The reaction was then warmed to 45 °C. Upon complete consumption of starting material by TLC, the reaction was cooled to 0 °C, and diluted with EtOAc (10 mL), followed by a saturated solution of NH_4Cl (20 mL). This mixture was then extracted with EtOAc (3x). The combined organic layers were rinsed with brine, dried over Na_2SO_4, filtered, and concentrated under reduced pressure. The crude reaction mixture was purified by silica gel flash column chromatography (0–20% EtOAc/hexanes) to afford **S1d** as a colorless oil (601 mg, 2.16 mmol, 57% yield).

^{1}H NMR (400 MHz, CDCl$_3$): δ 5.77 (ddt, *J* = 16.9, 10.2, 6.6 Hz, 1H), 5.37 – 5.24 (m, 1H), 5.04 – 4.89 (m, 2H), 4.62 (d, *J* = 7.3 Hz, 2H), 2.56 – 2.35 (m, 3H), 2.08 – 1.94 (m, 3H), 1.86 (ddd, *J* = 13.7, 12.3, 4.6 Hz, 1H), 1.78 – 1.47 (m, 10H), 1.46 – 1.19 (m, 3H).

^{13}C NMR (100 MHz, CDCl$_3$): δ 208.2, 172.2, 140.0, 138.5, 118.2, 114.8, 62.1, 61.0, 41.3, 36.4, 34.4, 34.2, 27.8, 25.8, 23.7, 22.8, 18.2.

IR (Neat Film, NaCl): 2938, 2864, 1713, 1639, 1449, 1379, 1339, 1308, 1210, 1186, 1143 cm^{-1}.

HRMS (ESI+): *m/z* calc'd for $C_{17}H_{26}O_3Na$ [M+Na]$^+$: 301.1774, found 301.1761.

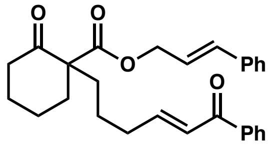

11a

cinnamyl 2-oxo-1-((*E*)-6-oxo-6-phenylhex-4-en-1-yl)cyclohexane-1-carboxylate (11a)

Prepared from **S3i** and dimethyl (2-oxo-2-phenylethyl)phosphonate[6] following General Procedure D. Purification by flash column chromatography (0–50% EtOAc/hexanes) afforded the title compound as a colorless oil (533 mg, 1.24 mmol, 81% yield).

^{1}H NMR (400 MHz, CDCl$_3$): δ 7.95 – 7.87 (m, 2H), 7.58 – 7.51 (m, 1H), 7.49 – 7.42 (m, 2H), 7.40 – 7.34 (m, 2H), 7.33 – 7.27 (m, 2H), 7.26 – 7.21 (m, 1H), 7.00 (dt, *J* = 15.4, 6.7 Hz, 1H), 6.86 (dt, *J* = 15.4, 1.4 Hz, 1H), 6.71 – 6.62 (m, 1H), 6.26 (dt, *J* = 15.9, 6.6 Hz, 1H), 4.87 – 4.73 (m, 2H), 2.59 – 2.39 (m, 3H), 2.38 – 2.23 (m, 2H), 2.08 – 1.87 (m, 2H), 1.82 – 1.57 (m, 4H), 1.55 – 1.36 (m, 3H).

^{13}C NMR (100 MHz, CDCl$_3$): δ 207.9, 191.0, 171.9, 149.0, 138.1, 136.1, 135.4, 132.7, 128.8, 128.7, 128.6, 128.4, 126.8, 126.3, 122.4, 66.0, 61.0, 41.3, 36.4, 34.5, 33.2, 27.7, 23.2, 22.8.

IR (Neat Film, NaCl): 2942, 2865, 1711, 1669, 1619, 1448, 1339, 1288, 1218, 1170, 1135, 1101 cm^{-1}.

HRMS (ESI+): *m/z* calc'd for $C_{28}H_{30}O_4Na$ [M+Na]$^+$: 453.2036, found 453.2031.

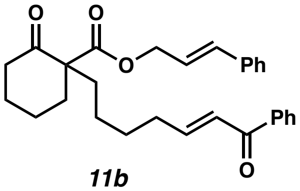

11b

cinnamyl 2-oxo-1-((*E*)-7-oxo-7-phenylhept-5-en-1-yl)cyclohexane-1-carboxylate (11b)
Prepared from **S3j** and dimethyl (2-oxo-2-phenylethyl)phosphonate[6] following General Procedure D. Purification by flash column chromatography (0–50% EtOAc/hexanes) afforded the title compound as a colorless oil (504 mg, 1.13 mmol, 76% yield).

^{1}H NMR (400 MHz, $CDCl_3$): δ 7.95 – 7.87 (m, 2H), 7.59 – 7.51 (m, 1H), 7.50 – 7.41 (m, 2H), 7.39 – 7.35 (m, 2H), 7.35 – 7.29 (m, 2H), 7.30 – 7.23 (m, 1H), 7.00 (dt, *J* = 15.4, 6.8 Hz, 1H), 6.82 (dt, *J* = 15.4, 1.5 Hz, 1H), 6.65 (dt, *J* = 15.8, 1.4 Hz, 1H), 6.25 (dt, *J* = 15.8, 6.5 Hz, 1H), 4.78 (dd, *J* = 6.6, 1.3 Hz, 2H), 2.59 – 2.50 (m, 1H), 2.49 – 2.38 (m, 2H), 2.34 – 2.20 (m, 2H), 2.05 – 1.87 (m, 2H), 1.81 – 1.59 (m, 4H), 1.57 – 1.41 (m, 3H), 1.38 – 1.20 (m, 2H).

^{13}C NMR (100 MHz, $CDCl_3$): δ 208.0, 191.0, 172.0, 149.6, 138.1, 136.1, 135.3, 132.7, 128.8, 128.7, 128.6, 128.4, 126.8, 126.1, 122.5, 65.8, 61.1, 41.3, 36.3, 34.6, 32.7, 28.6, 27.7, 24.1, 22.7.

IR (Neat Film, NaCl): 2939, 2862, 1711, 1668, 1620, 1578, 1495, 1447, 1338, 1284, 1214, 1168 cm^{-1}.

HRMS (ESI+): *m/z* calc'd for $C_{29}H_{32}O_4$ $[M+Na]^+$: 467.2193, found 467.2200.

Preparation of Aldehyde Precursors:
General Procedure E: Acylation, Alkylation of Carbonyls and Hydrolysis of Acetals

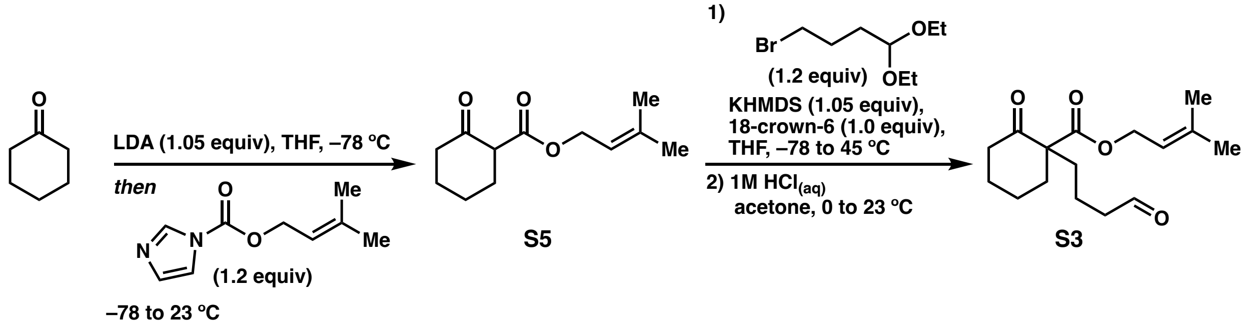

To a flame dried 250 mL round bottom flask was added *i*Pr_2NH (1.1 equiv) followed by THF (1.9 M) before the solution was cooled to 0 °C. To this, *n*-BuLi (2.5 M in hexanes, 1.05 equiv) was added dropwise before the solution was allowed to stir at 0 °C for 15 minutes. To this, a solution of the appropriate carbonyl compound (1.0 equiv) in THF (1.25 M) was added dropwise before the solution was allowed to stir at 0 °C for 30 minutes. The solution was then cooled to –78 °C, before a solution of 3-methylbut-2-en-1-yl 1*H*-imidazole-1-carboxylate[9] (1.2 equiv) in THF (4 M) was added dropwise. The reaction was warmed gradually to 23 °C. Upon complete consumption of starting material by TLC, the reaction was cooled to 0 °C, the pH was adjusted to 1.0 using 1 M HCl and the mixture was extracted with EtOAc (3x). The combined organic layers were rinsed

with brine, dried over Na_2SO_4, filtered, and concentrated under reduced pressure. The crude reaction mixture was purified by silica gel flash column chromatography to afford **S5** as a mixture of tautomers, which was used directly in the next step.

To a flame dried 250 mL round bottom flask was added KHMDS (1.05 equiv), and 18-crown-6 (1.0 equiv) followed by THF (0.19 M) before the solution was cooled to –78 °C. A solution of β-ketoester **S5** (1.0 equiv) in THF (0.38 M) was added dropwise at –78 °C. The solution was stirred at this temperature for 15 minutes before the appropriate alkyl bromide was added neat (1.2 equiv). The reaction was then warmed to 45 °C. Upon complete consumption of starting material by TLC, the reaction was cooled to 0 °C, and diluted with EtOAc followed by a saturated solution of NH_4Cl. This mixture was then extracted with EtOAc (3x). The combined organic layers were rinsed with brine, dried over Na_2SO_4, filtered, and concentrated under reduced pressure to afford the corresponding diethyl acetal which was used directly in the next step. A round bottom flask was charged with the corresponding diethyl acetal as a solution in acetone (0.5 M) before being cooled to 0 °C, and 1 M HCl was added (in a 2:1 volume ratio of 1 M HCl:acetone). The reaction was gradually warmed to 23 °C. Upon complete consumption of starting material by TLC, the reaction was diluted with EtOAc, extracted with EtOAc (3x), rinsed with brine, dried with Na_2SO_4, filtered, and concentrated under reduced pressure. The crude reaction mixture was purified by silica gel flash column chromatography to afford **S3**. *Note: Yields are reported based on alkylation and hydrolysis sequence. This is done because the corresponding β-keto esters were sometimes used to prepare multiple substrates.*

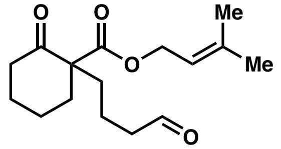

S3a

3-methylbut-2-en-1-yl 2-oxo-1-(4-oxobutyl)cyclohexane-1-carboxylate (S3a)

Prepared from cyclohexanone and 4-bromo-1,1-diethoxybutane[10] according to General Procedure E. Purification by flash column chromatography (0–60% EtOAc/hexanes) afforded the title compound as a colorless oil (715 mg, 2.55 mmol, 43% yield).

^{1}H NMR (400 MHz, $CDCl_3$): δ 9.73 (t, J = 1.6 Hz, 1H), 5.36 – 5.28 (m, 1H), 4.70 – 4.57 (m, 2H), 2.56 – 2.37 (m, 5H), 2.05 – 1.95 (m, 1H), 1.91 – 1.79 (m, 1H), 1.79 – 1.73 (m, 4H), 1.71 (s, 3H), 1.69 – 1.49 (m, 5H), 1.49 – 1.39 (m, 1H).

^{13}C NMR (100 MHz, $CDCl_3$): δ 208.0, 202.2, 172.0, 140.2, 118.1, 62.3, 60.9, 44.2, 41.2, 36.4, 34.3, 27.8, 25.8, 22.7, 18.2, 17.3.

IR (Neat Film, NaCl): 2939, 2866, 1713, 1450, 1380, 1211, 1179, 1137, 1096 cm^{-1}.

HRMS (ESI+): *m/z* calc'd for $C_{16}H_{24}O_4Na$ $[M+Na]^+$: 303.1567, found 393.1564.

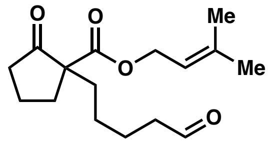

S3b

3-methylbut-2-en-1-yl 2-oxo-1-(5-oxopentyl)cyclopentane-1-carboxylate (S3b)
Prepared from cyclopentanone and 4-bromo-1,1-diethoxybutane[10] according to General Procedure E. Purification by flash column chromatography (0–50% EtOAc/hexanes) afforded the title compound as a colorless oil (631 mg, 2.37 mmol, 47% yield).

^{1}H NMR (400 MHz, $CDCl_3$): δ 9.75 (t, *J* = 1.4 Hz, 1H), 5.35 – 5.24 (m, 1H), 4.63 – 4.54 (m, 2H), 2.60 – 2.35 (m, 4H), 2.32 – 2.17 (m, 1H), 2.09 – 1.84 (m, 4H), 1.74 (s, 3H), 1.72 – 1.49 (m, 6H).

^{13}C NMR (100 MHz, $CDCl_3$): δ 214.9, 201.9, 171.0, 139.7, 118.2, 62.5, 60.4, 44.1, 38.1, 33.3, 33.0, 25.9, 19.8, 18.2, 17.6.

IR (Neat Film, NaCl): 2960, 1748, 1719, 1448, 1380, 1335, 1157 cm^{-1}.

HRMS (ESI+): *m/z* calc'd for $C_{15}H_{22}O_4Na$ $[M+Na]^+$: 289.1410, found 289.1408.

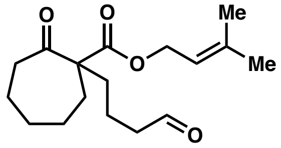

S3c

3-methylbut-2-en-1-yl 2-oxo-1-(4-oxobutyl)cycloheptane-1-carboxylate (S3c)
Prepared from cycloheptanone and 4-bromo-1,1-diethoxybutane[10] according to General Procedure E. Purification by flash column chromatography (0–50% EtOAc/hexanes) afforded the title compound as a colorless oil (1.34 g, 4.56 mmol, 57% yield).

^{1}H NMR (400 MHz, $CDCl_3$): δ 9.73 (t, *J* = 1.6 Hz, 1H), 5.37 – 5.24 (m, 1H), 4.62 (dd, *J* = 7.3, 3.1 Hz, 2H), 2.71 – 2.56 (m, 1H), 2.52 – 2.34 (m, 3H), 2.22 – 2.06 (m, 1H), 2.06 – 1.87 (m, 1H), 1.83 – 1.72 (m, 5H), 1.72 – 1.49 (m, 10H), 1.48 – 1.35 (m, 1H).

^{13}C NMR (100 MHz, $CDCl_3$): δ 209.6, 202.2, 172.4, 139.9, 118.2, 62.8, 62.2, 44.2, 42.3, 34.9, 33.0, 29.9, 25.8, 25.6, 25.0, 18.2, 17.6.

IR (Neat Film, NaCl): 2931, 2862, 1708, 1457, 1379, 1220, 1168 cm^{-1}.

HRMS (ESI+): *m/z* calc'd for $C_{17}H_{26}O_4Na$ $[M+Na]^+$: 317.1723, found 317.1719.

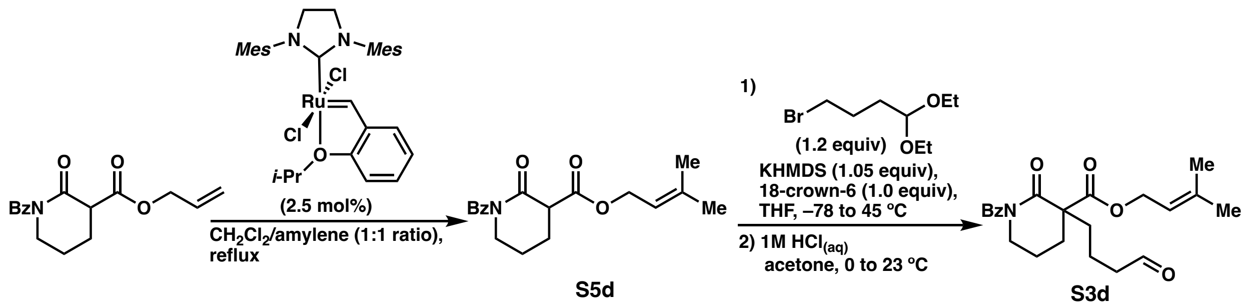

3-methylbut-2-en-1-yl 1-benzoyl-2-oxo-3-(4-oxobutyl)piperidine-3-carboxylate (S3d)

To a flame dried 2-neck 250 mL round bottom flask equipped with a reflux condenser was added HG-II (54.5 mg, 0.09 mmol, 0.025 equiv). Allyl 1-benzoyl-2-oxopiperidine-3-carboxylate[11] (1.00 g, 3.5 mmol, 1.0 equiv) was added as a solution in CH_2Cl_2 (35 mL, 0.1 M) followed by amylene (35 mL, 1:1 volume ratio with CH_2Cl_2). The reaction mixture was heated to reflux and monitored via NMR. Upon complete consumption of allyl 1-benzoyl-2-oxopiperidine-3-carboxylate, the reaction was cooled to 23 °C and concentrated under reduced pressure. The crude reaction mixture was purified by silica gel flash column chromatography (0–50% EtOAc/hexanes) to afford **S5d** (953 mg, 3.02 mmol, 87% yield), which was used directly in the next step.

To a flame dried 100 mL round bottom flask was added KHMDS (622 mg, 3.1 mmol, 1.05 equiv), and 18-crown-6 (784 mg, 3.0 mmol, 1.0 equiv) followed by THF (15 mL, 0.2 M) before the solution was cooled to –78 °C. A solution of β-ketoester **S5d** (936 mg, 3.0 mmol, 1.0 equiv) in THF (7.5 mL, 0.4 M) was added dropwise at –78 °C. The solution was stirred at this temperature for 15 minutes before 4-bromo-1,1-diethoxybutane[10] was added neat (801 mg, 3.6 mmol, 1.2 equiv). The reaction was then warmed to 45 °C. Upon complete consumption of starting material by TLC, the reaction was cooled to 0 °C, and diluted with EtOAc (10 mL) followed by a saturated solution of NH_4Cl (20 mL). This mixture was then extracted with EtOAc (3x). The combined organic layers were rinsed with brine, dried over Na_2SO_4, filtered, and concentrated under reduced pressure to afford the corresponding diethyl acetal which was used directly in the next step. A 50 mL round bottom flask was charged with the corresponding diethyl acetal as a solution in acetone (6 mL, 0.5 M) before being cooled to 0 °C, and 1 M HCl was added (in a 1:1 volume ratio of 1 M HCl:acetone). The reaction was gradually warmed to 23 °C. Upon complete consumption of starting material by TLC, the reaction was diluted with EtOAc, extracted with EtOAc (3x), rinsed with brine, dried with Na_2SO_4, filtered, and concentrated under reduced pressure. The crude reaction mixture was purified by silica gel flash column chromatography (0–50% EtOAc/hexanes) to afford **S3d** as a colorless oil (409 mg, 1.06 mmol, 36% yield).

^{1}H NMR (400 MHz, $CDCl_3$): δ 9.71 (t, *J* = 1.4 Hz, 1H), 7.78 – 7.68 (m, 2H), 7.51 – 7.43 (m, 1H), 7.41 – 7.34 (m, 2H), 5.47 – 5.38 (m, 1H), 4.81 – 4.65 (m, 2H), 3.88 – 3.69 (m, 2H), 2.54 – 2.30 (m, 3H), 2.07 – 1.95 (m, 2H), 1.93 – 1.85 (m, 2H), 1.84 – 1.79 (m, 3H), 1.79 – 1.69 (m, 4H), 1.67 – 1.43 (m, 2H).

^{13}C NMR (100 MHz, $CDCl_3$): δ 201.9, 175.1, 172.1, 172.1, 140.7, 136.0, 131.8, 128.2, 128.2, 117.8, 63.0, 56.7, 46.6, 44.0, 35.0, 30.7, 26.0, 20.5, 18.3, 17.5.

IR (Neat Film, NaCl): 2941, 1718, 1684, 1448, 1387, 1272, 1190, 1148 cm^{-1}.

HRMS (ESI+): *m/z* calc'd for $C_{22}H_{27}NO_5Na$ $[M+Na]^+$: 408.1781, found 408.1786.

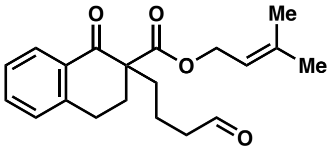

S3e

3-methylbut-2-en-1-yl 1-oxo-2-(4-oxobutyl)-1,2,3,4-tetrahydronaphthalene-2-carboxylate (S3e)

Prepared from 1-tetralone and 4-bromo-1,1-diethoxybutane[10] according to General Procedure E. Purification by flash column chromatography (0–50% EtOAc/hexanes) afforded the title compound as a colorless oil (531 mg, 1.62 mmol, 20% yield).

^{1}H NMR (400 MHz, $CDCl_3$): δ 9.76 (t, *J* = 1.5 Hz, 1H), 8.03 (dd, *J* = 7.9, 1.5 Hz, 1H), 7.46 (td, *J* = 7.5, 1.4 Hz, 1H), 7.30 (t, *J* = 7.6 Hz, 1H), 7.21 (d, *J* = 7.7 Hz, 1H), 5.26 – 5.17 (m, 1H), 4.64 – 4.49 (m, 2H), 3.07 (ddd, *J* = 17.4, 10.1, 4.7 Hz, 1H), 2.93 (dt, *J* = 17.4, 5.0 Hz, 1H), 2.57 (dt, *J* = 13.8, 5.0 Hz, 1H), 2.48 (t, *J* = 7.1 Hz, 2H), 2.23 – 2.11 (m, 1H), 2.03 – 1.86 (m, 2H), 1.86 – 1.72 (m, 1H), 1.72 – 1.62 (m, 4H), 1.58 (s, 3H).

^{13}C NMR (100 MHz, $CDCl_3$): δ 202.2, 195.6, 171.9, 143.2, 139.9, 133.6, 132.2, 128.8, 128.2, 126.9, 118.2, 62.4, 57.6, 44.2, 33.5, 30.9, 26.1, 25.8, 18.1, 17.7.

IR (Neat Film, NaCl): 2935, 2724, 1724, 1687, 1600, 1453, 1453, 1379, 1294, 1231, 1184, 1096 cm^{-1}.

HRMS (ESI+): *m/z* calc'd for $C_{20}H_{24}O_4Na$ $[M+Na]^+$: 351.1567, found 351.1571.

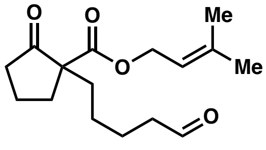

S3f

3-methylbut-2-en-1-yl 2-oxo-1-(5-oxopentyl)cyclopentane-1-carboxylate (S3f)

Prepared from cyclopentanone and 5-bromo-1,1-diethoxypentane[10] according to General Procedure E. Purification by flash column chromatography (0–40% EtOAc/hexanes) afforded the title compound as a colorless oil (886 mg, 3.16 mmol, 63% yield).

^{1}H NMR (400 MHz, $CDCl_3$): δ 9.75 (t, *J* = 1.7 Hz, 1H), 5.34 – 5.23 (m, 1H), 4.62 – 4.53 (m, 2H), 2.56 – 2.36 (m, 4H), 2.29 – 2.17 (m, 1H), 2.07 – 1.80 (m, 4H), 1.74 (s, 3H), 1.69 (s, 3H), 1.67 – 1.53 (m, 3H), 1.44 – 1.32 (m, 1H), 1.31 – 1.18 (m, 1H).

^{13}C NMR (100 MHz, $CDCl_3$): δ 215.0, 202.4, 171.1, 139.6, 118.3, 62.5, 60.5, 43.7, 38.1, 33.6, 33.1, 25.9, 24.5, 22.4, 19.8, 18.2.

IR (Neat Film, NaCl): 2947, 2725, 1748, 1722, 1451, 1380, 1224, 1159, 1127 cm^{-1}.

HRMS (ESI+): *m/z* calc'd for $C_{16}H_{24}O_4Na$ $[M+Na]^+$: 303.1567, found 303.1571.

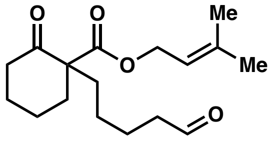

S3g

3-methylbut-2-en-1-yl 2-oxo-1-(5-oxopentyl)cyclohexane-1-carboxylate (S3g)
Prepared from cyclohexanone and 5-bromo-1,1-diethoxypentane[10] according to General Procedure E. Purification by flash column chromatography (0–60% EtOAc/hexanes) afforded the title compound as a colorless oil (617 mg, 2.10 mmol, 44% yield).

^{1}H NMR (400 MHz, CDCl$_3$): δ 9.74 (t, *J* = 1.7 Hz, 1H), 5.35 – 5.25 (m, 1H), 4.70 – 4.53 (m, 2H), 2.56 – 2.33 (m, 5H), 2.05 – 1.95 (m, 1H), 1.92 – 1.80 (m, 1H), 1.74 (s, 3H), 1.73 – 1.67 (m, 4H), 1.66 – 1.48 (m, 5H), 1.47 – 1.35 (m, 1H), 1.34 – 1.14 (m, 2H).

^{13}C NMR (100 MHz, CDCl$_3$): δ 208.2, 202.6, 172.1, 140.0, 118.1, 62.2, 60.9, 43.8, 41.3, 36.4, 34.6, 27.8, 25.8, 24.0, 22.7, 22.5, 18.2.

IR (Neat Film, NaCl): 2939, 2865, 2724, 1712, 1449, 1380, 1297, 1173, 1136, 1097 cm^{-1}.

HRMS (ESI+): *m/z* calc'd for $C_{17}H_{26}O_4Na$ [M+Na]$^+$: 317.1723, found 317.1719.

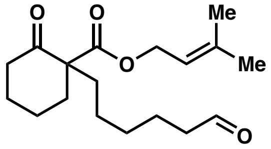

S3h

3-methylbut-2-en-1-yl 2-oxo-1-(6-oxohexyl)cyclohexane-1-carboxylate (S3h)
Prepared from cyclohexanone and 6-bromo-1,1-diethoxyhexane[10] according to General Procedure E. Purification by flash column chromatography (0–60% EtOAc/hexanes) afforded the title compound as a colorless oil (515 mg, 1.67 mmol, 44% yield).

^{1}H NMR (400 MHz, CDCl$_3$): δ 9.75 (t, *J* = 1.8 Hz, 1H), 5.40 – 5.23 (m, 1H), 4.70 – 4.54 (m, 2H), 2.57 – 2.32 (m, 5H), 2.06 – 1.95 (m, 1H), 1.85 (ddd, *J* = 13.3, 12.1, 4.1 Hz, 1H), 1.79 – 1.67 (m, 7H), 1.67 – 1.56 (m, 4H), 1.54 – 1.37 (m, 2H), 1.36 – 1.11 (m, 4H).

^{13}C NMR (100 MHz, CDCl$_3$): δ 208.3, 202.8, 172.2, 140.0, 118.2, 62.1, 61.0, 43.9, 41.3, 36.5, 34.7, 29.6, 27.8, 25.9, 24.1, 22.8, 21.9, 18.2.

IR (Neat Film, NaCl): 2936, 2862, 2720, 1712, 1450, 1380, 1270, 1200, 1170, 1136, 1098 cm^{-1}.

HRMS (ESI+): *m/z* calc'd for $C_{18}H_{28}O_4Na$ [M+Na]$^+$: 331.1880, found 331.1880.

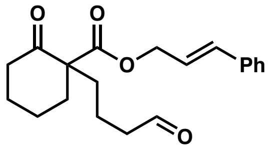

S3i

cinnamyl 2-oxo-1-(4-oxobutyl)cyclohexane-1-carboxylate (S3i)
Prepared from cyclohexanone, cinnamyl 1*H*-imidazole-1-carboxylate (in place of 3-methylbut-2-en-1-yl 1*H*-imidazole-1-carboxylate), and 4-bromo-1,1-diethoxybutane[10] according to General Procedure E. Purification by flash column chromatography (0–60% EtOAc/hexanes) afforded the title compound as a colorless oil (1.81 g, 5.50 mmol, 37% yield).

^{1}H NMR (400 MHz, CDCl$_3$): δ 9.77 – 9.65 (m, 1H), 7.41 – 7.36 (m, 2H), 7.36 – 7.30 (m, 2H), 7.30 – 7.26 (m, 1H), 6.67 (d, *J* = 15.8 Hz, 1H), 6.27 (dtd, *J* = 15.9, 6.6, 0.8 Hz, 1H), 4.87 – 4.74 (m, 2H), 2.59 – 2.36 (m, 5H), 2.04 – 1.96 (m, 1H), 1.92 – 1.82 (m, 1H), 1.82 – 1.43 (m, 7H).

^{13}C NMR (100 MHz, CDCl$_3$): δ 207.8, 202.1, 171.8, 136.1, 135.4, 128.8, 128.4, 126.8, 122.4, 66.0, 61.0, 44.1, 41.2, 36.2, 34.2, 27.7, 22.7, 17.3.

IR (Neat Film, NaCl): 2944, 2866, 1713, 1495, 1450, 1242, 1206, 1178, 1137, 1098 cm^{-1}.

HRMS (ESI+): *m/z* calc'd for $C_{20}H_{24}O_4Na$ [M+Na]$^+$: 351.1567, found 351.1562.

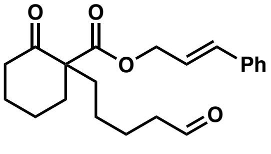

S3j

cinnamyl 2-oxo-1-(5-oxopentyl)cyclohexane-1-carboxylate (S3j)
Prepared from cyclohexanone, cinnamyl 1*H*-imidazole-1-carboxylate (in place of 3-methylbut-2-en-1-yl 1*H*-imidazole-1-carboxylate), and 5-bromo-1,1-diethoxypentane[10] according to General Procedure E. Purification by flash column chromatography (0–60% EtOAc/hexanes) afforded the title compound as a colorless oil (767 mg, 2.24 mmol, 45% yield).

^{1}H NMR (600 MHz, CDCl$_3$): δ 9.69 (t, *J* = 1.7 Hz, 1H), 7.41 – 7.36 (m, 2H), 7.36 – 7.31 (m, 2H), 7.30 – 7.26 (m, 1H), 6.66 (dd, *J* = 15.9, 1.6 Hz, 1H), 6.26 (dt, *J* = 15.9, 6.6 Hz, 1H), 4.79 (dd, *J* = 6.6, 1.3 Hz, 2H), 2.56 – 2.41 (m, 3H), 2.40 – 2.31 (m, 2H), 2.04 – 1.96 (m, 1H), 1.90 (ddd, *J* = 13.5, 12.2, 4.6 Hz, 1H), 1.80 – 1.72 (m, 1H), 1.71 – 1.53 (m, 5H), 1.46 (ddd, *J* = 13.6, 12.0, 4.2 Hz, 1H), 1.34 – 1.18 (m, 2H).

^{13}C NMR (100 MHz, CDCl$_3$): δ 207.9, 202.5, 171.9, 136.1, 135.4, 128.8, 128.5, 126.8, 122.4, 65.9, 61.0, 43.7, 41.3, 36.3, 34.6, 27.7, 24.1, 22.7, 22.5.

IR (Neat Film, NaCl): 2944, 2865, 1713, 1495, 1451, 1240, 1310, 1240, 1206, 1174, 1135, 1098 cm^{-1}.

HRMS (ESI+): *m/z* calc'd for $C_{21}H_{26}O_4Na$ [M+Na]$^+$: 365.1723, found 365.1721.

Product Derivatizations:

General Procedure F: Samarium Mediated Pinacol Coupling[12]

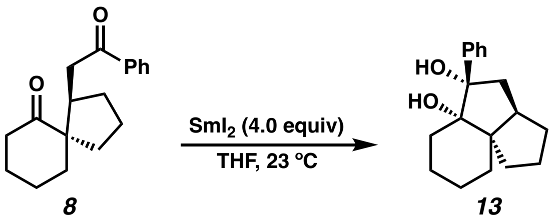

To a flame dried round bottom flask was added a solution of SmI_2 (0.1 M in THF, 4.0 equiv), followed by a solution of the corresponding diketone (1.0 equiv) in THF (0.1 M). Upon complete consumption of starting material by TLC, the reaction was diluted with Et_2O, followed by further dilution with a saturated aqueous solution of $NaHCO_3$. The mixture was extracted with Et_2O (3x), rinsed with brine, dried with Na_2SO_4, filtered, and concentrated under reduced pressure. The crude reaction mixture was purified by silica gel flash column chromatography or preparative TLC to afford the corresponding diol.

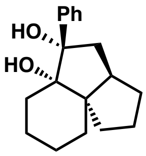

13

(3a*R*,5*S*,5a*S*,9a*S*)-5-phenyldecahydro-5a*H*-cyclopenta[*c*]indene-5,5a-diol (13)

Prepared from diketone **8a** according to General Procedure F. Purification by flash column chromatography (0–40% Et_2O/hexanes) afforded the title compound as a colorless oil (23 mg, 0.084 mmol, 56% yield). Structure assigned via 1D ^1H and ^{13}C NMR, 2D NMR studies (see below), and comparison with compounds found in the literature. Cis configuration was assigned via carbonate formation[12] (see **14** below).

^{1}H NMR (400 MHz, CDCl$_3$): δ 7.64 – 7.58 (m, 2H), 7.38 – 7.31 (m, 2H), 7.29 – 7.22 (m, 1H), 4.12 (s, 1H), 2.73 (s, 1H), 2.65 – 2.47 (m, 2H), 2.12 – 1.94 (m, 2H), 1.72 – 1.58 (m, 3H), 1.55 – 1.47 (m, 1H), 1.41 – 1.11 (m, 7H), 1.07 – 0.96 (m, 1H), 0.12 – -0.05 (m, 1H).

^{13}C NMR (100 MHz, CDCl$_3$): δ 144.3, 127.9, 127.6, 127.2, 90.7, 79.3, 58.6, 47.0, 37.6, 35.3, 26.4, 25.7, 25.0, 22.3, 21.9, 20.8.

IR (Neat Film, NaCl): 3307, 2946, 2871, 1494, 1445, 1394, 1221, 1050, 1019 cm^{-1}.

HRMS (FD+): *m/z* calc'd for $C_{18}H_{24}O_2$ $[M]^{+\cdot}$: 272.17708, found 272.17608.

Optical Rotation: $[\alpha]_D^{23}$ –30.0 (c = 1.53, $CHCl_3$).

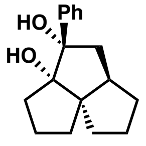

15

(3a*S*,4*S*,5a*R*,8a*R*)-4-phenyloctahydrocyclopenta[*c*]pentalene-3a,4(1*H*)-diol (15)
Prepared from diketone **8c** according to General Procedure F. Purification by preparative TLC (40% Et_2O/hexanes) afforded the title compound as a colorless oil (7.0 mg, 0.027 mmol, 68% yield).

^{1}H NMR (400 MHz, $CDCl_3$): δ 7.49 – 7.42 (m, 2H), 7.38 – 7.31 (m, 2H), 7.31 – 7.27 (m, 1H), 3.29 (s, 1H), 3.12 (s, 1H), 2.52 – 2.36 (m, 1H), 2.16 (dd, *J* = 13.5, 12.1 Hz, 1H), 2.12 – 2.02 (m, 1H), 2.01 – 1.88 (m, 1H), 1.82 (dd, *J* = 12.2, 4.5 Hz, 1H), 1.72 – 1.58 (m, 2H), 1.57 – 1.48 (m, 2H), 1.47 – 1.35 (m, 2H), 1.34 – 1.21 (m, 3H), 1.16 – 1.01 (m, 1H).

^{13}C NMR (100 MHz, $CDCl_3$): δ 144.5, 128.3, 127.6, 126.5, 90.0, 87.3, 64.3, 47.4, 39.5, 35.9, 34.0, 31.1, 26.6, 22.6, 22.1.

Note: A grease and water signal are observed in the ^{1}H and ^{13}C spectra.

IR (Neat Film, NaCl): 3334, 2953, 2873, 1446, 1376, 1128, 1100, 1046 cm^{-1}.

HRMS (FD+): *m/z* calc'd for $C_{17}H_{22}O_2$ $[M]^{+\cdot}$: 258.16143, found 258.16203.

Optical Rotation: $[\alpha]_D^{23}$ –36.0 (c = 0.58, $CHCl_3$).

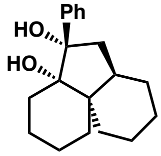

16

(4a*S*,5*S*,6a*R*,10a*R*)-5-phenyldecahydrobenzo[*c*]indene-4a,5(5*H*)-diol (16)
Prepared from diketone **8k** according to General Procedure F. Purification by preparative TLC (40% Et_2O/hexanes) afforded the title compound as a colorless crystalline solid (5.2 mg, 0.018 mmol, 37% yield). Absolute and relative stereochemistry were assigned by X-ray crystallography (vide infra).

^{1}H NMR (400 MHz, $CDCl_3$): δ 7.64 – 7.55 (m, 2H), 7.38 – 7.30 (m, 2H), 7.30 – 7.21 (m, 1H), 3.92 (s, 1H), 2.77 (s, 1H), 2.58 (t, *J* = 13.4 Hz, 1H), 2.20 – 2.05 (m, 1H), 1.82 – 1.66 (m, 4H), 1.61 (dd, *J* = 11.6, 2.9 Hz, 1H), 1.55 – 1.43 (m, 3H), 1.43 – 1.14 (m, 6H), 1.13 – 0.96 (m, 2H), 0.20 – 0.02 (m, 1H).

^{13}C NMR (100 MHz, $CDCl_3$): δ 144.6, 127.9, 127.6, 127.2, 83.7, 83.2, 46.6, 42.2, 41.7, 34.1, 28.2, 26.3, 24.7, 22.2, 21.4, 20.9, 19.2.

IR (Neat Film, NaCl): 3310, 2924, 2857, 1495, 1444, 1391, 1143, 1038, 1028 cm^{-1}.

HRMS (FD+): *m/z* calc'd for $C_{19}H_{26}O_2$ $[M]^{+\cdot}$: 286.19273, found 286.19351.

Optical Rotation: $[\alpha]_D^{23}$ –27.4 (c = 0.43, $CHCl_3$).

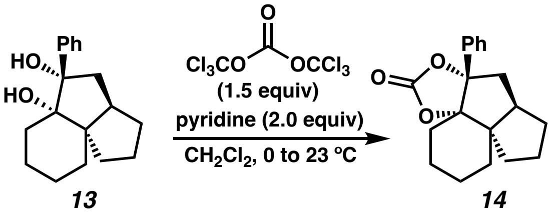

(3a*S*,4a*R*,7a*S*,11a*S*)-3a-phenyldecahydrocyclopenta[3,3a]indeno[1,7a-*d*][1,3]dioxol-2-onediol (14)

To a flame dried 2-dram vial was added a solution of diol **13** (5.0 mg, 0.02 mmol, 1.0 equiv) in CH_2Cl_2 (0.02 M, 1.0 mL) then the solution was cooled to 0 °C. Pyridine (3 µL, 0.04 mmol, 2.0 equiv) was added before a solution of triphosgene (8.9 mg, 0.03 mmol, 1.5 equiv) in CH_2Cl_2 (0.06 M, 0.5 mL) was added dropwise at 0 °C. *Note: serious caution must be taken when working with triphosgene due to its high toxicity. Review MSDS information before using.* Upon complete consumption of starting material by TLC, the reaction was diluted with saturated aqueous NH_4Cl, extracted with Et_2O (3x), rinsed with 1 M HCl, saturated aqueous $NaHCO_3$, and brine. Following this the organic layer was dried with Na_2SO_4, filtered, and concentrated under reduced pressure. Purification of the crude compound via flash column chromatography (0–50% Et_2O/hexanes) afforded the title compound as a colorless oil (4.1 mg, 0.014 mmol, 69% yield). Successful formation of the carbonate (assigned via ^1H and ^{13}C NMR) is consistent with **13** being the cis-diol. This is due to the high degree of ring strain associated with the corresponding trans-fused [3.3.0] ring system.[12]

^{1}H NMR (400 MHz, $CDCl_3$): δ 7.54 – 7.48 (m, 2H), 7.46 – 7.34 (m, 3H), 2.59 (t, *J* = 13.6 Hz, 1H), 2.48 – 2.36 (m, 1H), 2.19 – 2.03 (m, 3H), 1.76 – 1.65 (m, 2H), 1.64 – 1.56 (m, 2H), 1.54 – 1.30 (m, 4H), 1.29 – 1.15 (m, 3H), 0.36 – 0.20 (m, 1H).

Note: A grease and water signal are observed in the ^1H spectrum.

^{13}C NMR (100 MHz, $CDCl_3$): δ 154.9, 136.4, 129.0, 128.5, 127.0, 102.4, 91.7, 58.2, 47.1, 35.2, 30.3, 26.6, 25.9, 25.2, 22.3, 21.0, 20.4.

IR (Neat Film, NaCl): 2948, 2877, 1794, 1447, 1298, 1272, 1241, 1060 cm^{-1}.

HRMS (FD+): *m/z* calc'd for $C_{19}H_{22}O_3$ $[M]^{+\cdot}$: 298.15635, found 298.15641.

Optical Rotation: $[\alpha]_D^{23}$ –12.2 (c = 0.34, $CHCl_3$).

General Procedure G: Aldol Condensation[13]

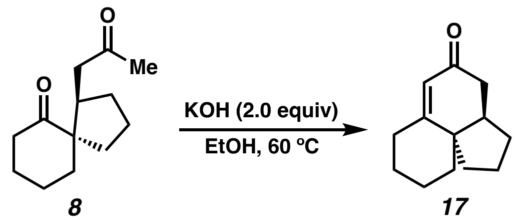

To a flame dried round bottom flask was added a solution of KOH (2.3 equiv) in EtOH (0.14 M) followed by a solution of the corresponding ketone (1.0 equiv) in EtOH (0.1 M). The reaction was heated to 60 °C. Upon complete consumption of starting material by TLC, the reaction was quenched with AcOH (2.3 equiv) and concentrated under reduced pressure. The crude reaction mixture was purified by silica gel flash column chromatography to afford the corresponding enone.

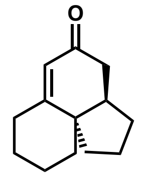

17

(3a*R*,10a*S*)-2,3,3a,4,7,8,9,10-octahydrocyclopenta[*d*]naphthalen-5(1*H*)-one (17)
Prepared from diketone **8b** according to General Procedure G. Purification by flash column chromatography (0–45% Et_2O/hexanes) afforded the title compound as a colorless oil (13.1 mg, 0.069 mmol, 72% yield).

^{1}H NMR (400 MHz, $CDCl_3$): δ 5.77 (s, 1H), 2.42 (dd, J = 16.5, 3.7 Hz, 1H), 2.35 – 2.15 (m, 3H), 2.15 – 1.94 (m, 3H), 1.83 – 1.67 (m, 5H), 1.62 – 1.53 (m, 1H), 1.51 – 1.27 (m, 4H).

^{13}C NMR (100 MHz, $CDCl_3$): δ 200.6, 175.7, 123.2, 48.4, 44.8, 37.9, 35.1, 32.3, 31.7, 30.6, 26.2, 21.9, 20.0.

IR (Neat Film, NaCl): 2937, 2870, 1668, 1607, 1445, 1369, 1277, 1250, 1213, 1164, 1133, 1101 cm^{-1}.

HRMS (FI+): *m/z* calc'd for $C_{13}H_{18}O$ $[M]^{+\cdot}$: 190.13522, found 190.13552.

Optical Rotation: $[\alpha]_D^{23}$ +127.5 (c = 1.09, $CHCl_3$).

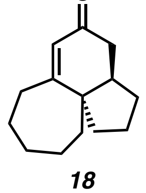

18

(3a*R*,11a*S*)-2,3,3a,4,8,9,10,11-octahydro-1*H*-cyclohepta[*d*]inden-5(7*H*)-one (18)
Prepared from diketone **8f** according to General Procedure G. Purification by preparative TLC (30% Et_2O/hexanes) afforded the title compound as a colorless oil (6.1 mg, 0.030 mmol, 66% yield).

^{1}H NMR (400 MHz, CDCl$_3$): δ 5.71 (s, 1H), 2.63 – 2.52 (m, 1H), 2.45 (dd, J = 17.6, 4.8 Hz, 1H), 2.39 – 2.27 (m, 2H), 2.19 – 1.99 (m, 2H), 1.87 – 1.53 (m, 8H), 1.53 – 1.36 (m, 3H), 1.32 – 1.15 (m, 2H).

^{13}C NMR (100 MHz, CDCl$_3$): δ 201.4, 174.0, 127.1, 49.4, 47.7, 38.2, 34.9, 30.7, 30.7, 26.6, 26.3, 26.2, 25.5, 20.5.

Note: A grease and water signal are observed in the ^{1}H and ^{13}C spectra.

IR (Neat Film, NaCl): 2929, 2870, 1665, 1600, 1443, 1368, 1283, 1254 cm^{-1}.

HRMS (FI+): *m/z* calc'd for $C_{13}H_{18}O$ [M]$^{+\cdot}$: 204.15087, found 204.15113.

Optical Rotation: $[\alpha]_D^{23}$ +41.7 (c = 0.51, $CHCl_3$).

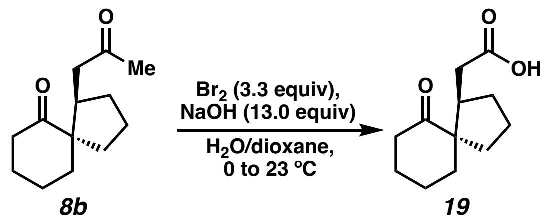

2-((1*R*,5*S*)-6-oxospiro[4.5]decan-1-yl)acetic acid (19)
To a dried 2-dram vial was added a solution of diketone **8b** (20 mg, 0.096 mmol, 1.0 equiv) in 1,4-dioxane (0.1 M, 1.0 mL) and water (0.5 M, 0.2 mL) before being cooled to 0 °C. A solution of NaOBr in water was then prepared: NaOH (50 mg, 1.25 mmol, 13.0 equiv) was dissolved in water (2.6 M, 0.48 mL) and cooled to 0 °C. Bromine (16.5 µL, 0.32 mmol, 3.3 equiv) was then added dropwise at 0 °C. The NaOBr solution was then added dropwise to the solution of **8b** at 0 °C. The reaction was allowed to gradually warm to 23 °C. Upon complete consumption of starting material by TLC, the reaction was diluted with 2 mL of water and then quenched by slow addition of concentrated HCl (until pH was 1.0). The mixture was then extracted with EtOAc (3x), rinsed with brine, dried with Na_2SO_4, filtered, and concentrated under reduced pressure. Purification of the crude mixture via preparative TLC purification (79% Et_2O, 20% hexanes, 1% AcOH) afforded the title compound as a white solid (6.9 mg, 0.033 mmol, 34% yield).

^{1}H NMR (400 MHz, CDCl$_3$): δ 2.95 – 2.82 (m, 1H), 2.50 – 2.35 (m, 3H), 2.11 (dd, J = 15.0, 10.4 Hz, 1H), 2.04 – 1.92 (m, 2H), 1.92 – 1.80 (m, 1H), 1.80 – 1.48 (m, 8H), 1.39 – 1.26 (m, 1H).

^{13}C NMR (100 MHz, CDCl$_3$): δ 213.9, 178.6, 58.3, 40.1, 39.3, 35.1, 35.0, 31.7, 29.9, 27.1, 21.8, 21.8.

IR (Neat Film, NaCl): 3021, 2941, 2865, 1705, 1444, 1420, 1281, 1235, 1164, 1129 cm^{-1}.

HRMS (FD+): *m/z* calc'd for $C_{12}H_{18}O_3$ [M]$^{+\cdot}$: 210.12505, found 210.12518.

Optical Rotation: $[\alpha]_D^{23}$ +7.7 (c = 0.58, $CHCl_3$).

General Notes – Quantum Mechanics Calculations:

All quantum mechanics calculations were carried out with the ORCA program.[14] Geometry optimizations, harmonic frequency calculations, and single-point energy evaluations were carried out with density functional theory (DFT). The PBE0 hybrid functional[15] paired with Becke–Johnson damped D4 dispersion corrections[16], henceforth referred to as PBE0-D4, was used in this study. For geometry optimization and harmonic frequency calculations, Pd is described by the def2-TZVP all electron basis set[17] and the ECP28MWB small-core (18 explicit valence electrons) quasi-relativistic pseudopotential,[18] while C, H, N, and P are assigned the def2-SVP basis. Diffuse functions are added to oxygen (ma-def2-SVP). Herein, we refer to this composite basis set as BS1. This combination of functional and basis set was chosen as it has been shown to be reliable in prior benchmarking studies of similar systems,[19b] and has subsequently been employed to study various Pd-catalyzed reactions reported by our group and has remained robust, providing results in good agreement with experimental observations.[19] Geometry optimization and harmonic frequency calculations were carried out with the CPCM implicit solvation model for toluene (PhMe, $\varepsilon = 2.4$). For all calculations employing CPCM, surface charges are described by the improved Gaussian charge scheme of Neese and coworkers with a scaled Van der Waals cavity ($\alpha = 1.2$).[20] All Hessians were computed analytically. Stationary points are characterized by the correct number of imaginary vibrational modes (zero for minima and one for saddle points). Intrinsic reaction coordinate (IRC) analysis confirms the nature of transition states.[21] Cartesian coordinates of all optimized structures are included as ".xyz" files, available online in a compressed zip file format.

Electronic energies are further refined with single-point calculations employing the PBE0-D4 functional[22] and the def2-TZVPP basis set on all atoms (with the ECP28MWB pseudopotential for Pd) with additional diffuse functions on O (ma-def2-TZVPP). This mixed basis is henceforth referred to as BS2. Solvation was accounted for with CPCM as mentioned above (PhMe, $\varepsilon = 2.4$). Final Gibbs free energies were obtained by applying thermodynamic corrections to the calculated electronic energies. Thermodynamic corrections from harmonic frequency calculations employ the quasi-ridged rotor harmonic oscillator approach to correct for the breakdown of the harmonic oscillator approximation at low vibrational frequencies.[23] The translational (S_{trans}) and rotational entropy (S_{rot}) contributions to the Gibbs free energy calculated for a complex in condensed phase are *ca.* 40–60% of the values obtained assuming an ideal gas.[24] As suggested in the literature, S_{trans} and S_{rot} obtained by ideal gas treatment are scaled by a factor of 0.5 to obtain the final condensed phase values.[25] Hence, the final Gibbs free energy at 313.15 K is calculated as:

$$G_{solv}^{*} = E_{el,solv}^{BS2} + ZPE + E_{trans} + E_{rot} + E_{vib} + k_bT - T\left(S_{el} + S_{vib} + \frac{1}{2}S_{trans} + \frac{1}{2}S_{rot}\right)$$

The resolution of identity (RI) and Chain-of-Spheres (COS) approximations are employed for efficient evaluation of Coulomb and exchange integrals, respectively.[26] The def2/J auxiliary basis[27] is employed for all atoms except oxygen, for which a suitable auxiliary was obtained via the automatic generation algorithm in the ORCA program (keyword: *AutoAux*).[28] Very fine grid settings are employed in all calculations (optimization/frequency calculations: DefGrid2, single point calculations: DefGrid3).

Conformer searching was carried out for each stationary point individually using the meta-dynamics-based CREST program (using GNF-FF) from the Grimme group.[29] Duplicate conformers were removed, low energy conformers were subsequently optimized, and energies were evaluated at the cheaper PBE0-D4/def2-TZVPP (Pd), ma-def2-TZVPP (O), def2-TZVPP/CPCM(PhMe)//PBE-D4/def2-TZVP (Pd), ma-def2-SV(P) (O), def2-SV(P) level of theory. The final low energy conformers were further optimized at the level of theory mentioned

prior, and the free energies and the optimized structures of the lowest-energy conformers are reported. It is assumed that interconversion of conformers of a single stationary point proceeds with a low barrier compared to bond formation and breakage processes and is inconsequential to the evaluation of reaction pathways.

Comparison of Michael Addition Transition State Barriers:

Explicit conformer searching for rotation about the Pd–O–C–C(enolate) dihedral was performed for the Michael addition transition states (Figure S1). A strong preference for placement of the enolate electrophile tether *syn* to the ligand *t*-Bu group is observed for transition states to form *S* stereocenters at the spirocyclic quaternary carbon (Figure S1 **TS2a** and **TS2c**). This arrangement not only avoids undesired steric interactions of the rather bulky cyclohexyl group and the ligand *t*-Bu group but also places the *Re* face in the more sterically accessible orientation, external of the Pd complex.

In contrast, to form the *R* spirocyclic stereocenters in products **23b** and **23d**, either internal electrophile approach to the sterically hindered *Si* face of the enolate or positioning the bulky cyclohexyl group *syn* to the ligand *t*-Bu substituent is required. These destabilizing steric interactions contribute to the increase in energy barriers of **TS2b**, **TS2d**, **TS-S2**, and **TS-S4**. Between **TS2b** and **TS-S2**, steric clash resulting from internal electrophile approach outweighs preference for placement of electrophile tether *syn* to the ligand *t*-Bu group, leading to **TS2b** being the lower energy conformer. On the other hand, these two steric interactions cause energy increase of similar magnitudes in both **TS2d** and **TS-S4**.

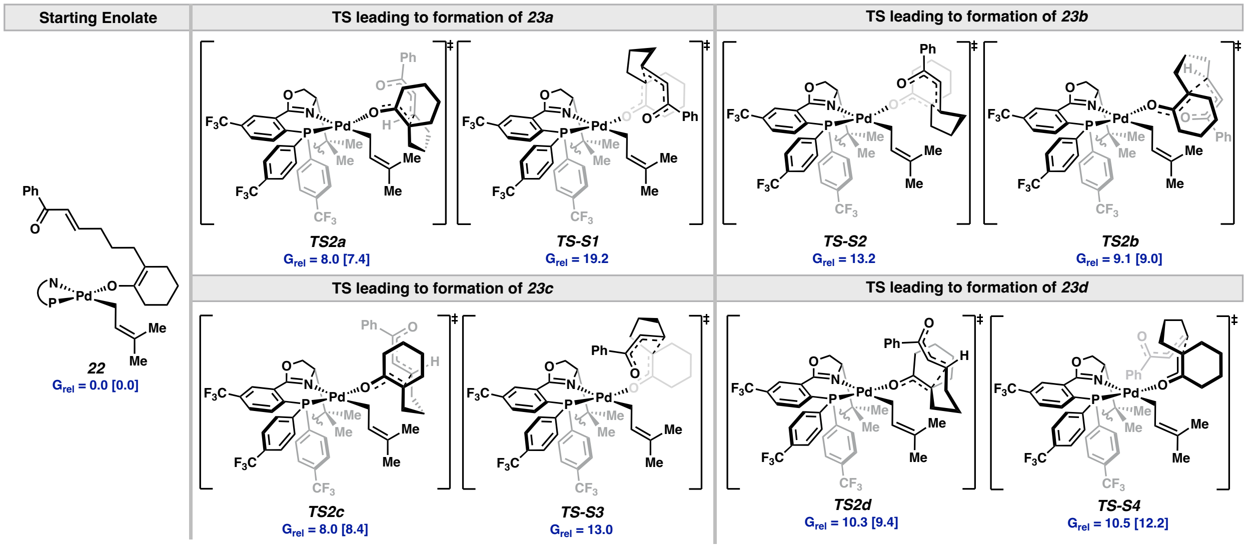

Figure S1. Relative free energies for transition states explored for the Michael addition process. Gibbs free energies in kcal/mol computed at the PBE0-D4/BS2/CPCM(PhMe)//PBE-D4/def2-TZVP (Pd), ma-def2-SV(P) (O), def2-SV(P) level of theory, and in brackets are energies calculated at the PBE0-D4/BS2/CPCM(PhMe)//PBE0-D4/BS1/CPCM(PhMe) level of theory at 313.15 K.

The major stereoisomer predicted based on these calculations is consistent with the major stereoisomer observed in all compounds which were structurally elucidated via x-ray crystallography (**8a**, **8b**, **12a**, **16**). Although additional computational and experimental evidence would be required to explain other observations regarding distinct substrate classes, we hypothesize that more general insights can potentially be taken from this stereochemical model. For instance, N-benzoyl lactam **8g** is modestly enantioselective but has good diastereoselectivity.

Looking at **TS2a**, we imagine that the N-benzoyl moiety might have a destabilizing steric interaction with our ligand, potentially raising the energy of that transition state and thus decreasing the energy gap with the transition state leading to formation of the enantiomeric product. Tetralone **8h** has good diastereoselectivity but poor enantioselectivity for both diastereomers. Electronically and sterically, tetralones are a unique substrate class and it is speculated that this fused aryl ring could influence the energies of many of the transition structures investigated. In addition, phenyl ester **8i** is formed in poor diastereoselectivity with decent levels of enantioselectivity, which is potentially due to altered geometrical elements (i.e. enolate conformation) and steric interactions with the ligand.

Computed Energies for Intermediates Post Michael Addition:

As apically bound enolate intermediate **23a** is an unusual and high-energy intermediate, we propose that following its formation, isomerization to lower-energy Pd intermediates can occur. In addition to intermediates **24** and **26** (Figure S4), various Pd intermediates were explored and are shown in Figure S2.

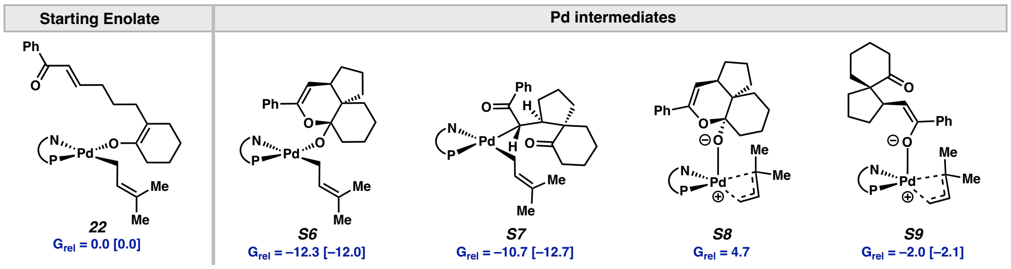

Figure S2. Relative free energies for Pd intermediates explored. Gibbs free energies in kcal/mol computed at the PBE0-D4/BS2/CPCM(PhMe)//PBE-D4/def2-TZVP (Pd), ma-def2-SV(P) (O), def2-SV(P) level of theory, and in brackets are energies calculated at the PBE0-D4/BS2/CPCM(PhMe)//PBE0-D4/BS1/CPCM(PhMe) level of theory at 313.15 K.

Investigation of the Mechansim of Proton Transfer:

We hypothesized that in analogy to previously investigated systems,[30] catalyst turnover can be achieved through the proton transfer from the prenyl group to a Pd enolate and the concomitant reduction of Pd. We therefore sampled the inner-sphere, outer-sphere, and N-detached inner-sphere proton transfer transition states (Figure S3) analogous to the lowest energy pathways of each type of mechanism observed in previously investigated systems.[30] Proton transfer to both *Si* and *Re* face of the enolates were sampled for the N-detached inner-sphere pathway.

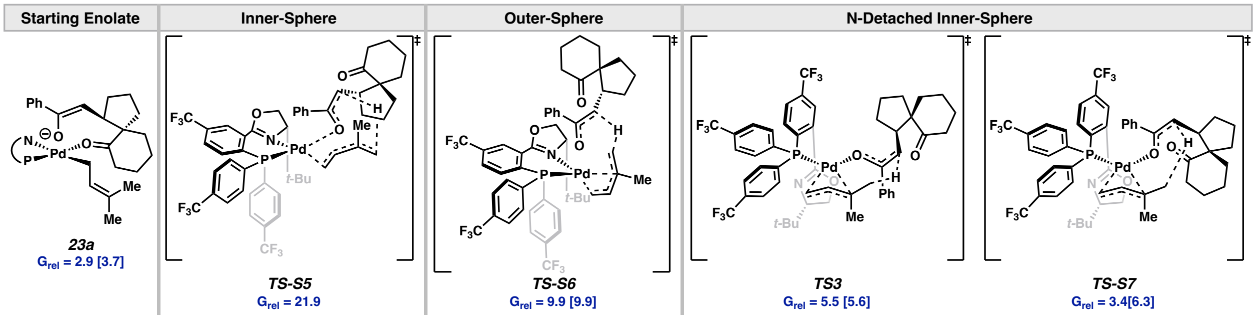

Figure S3. Relative free energies for proton transfer pathways studied. Gibbs free energies in kcal/mol computed at the PBE0-D4/BS2/CPCM(PhMe)//PBE-D4/def2-TZVP (Pd), ma-def2-SV(P) (O), def2-SV(P) level of theory, and in brackets are energies calculated at the PBE0-D4/BS2/CPCM(PhMe)//PBE0-D4/BS1/CPCM(PhMe) level of theory at 313.15 K. The lowest-energy conformer for TS-S7 at the PBE0-D4/BS2/CPCM(PhMe)//PBE-

D4/def2-TZVP (Pd), ma-def2-SV(P) (O), def2-SV(P) level of theory failed to converge in geometry optimization at the PBE0-D4/BS2/CPCM(PhMe)//PBE0-D4/BS1/CPCM(PhMe) level of theory (due to a small imaginary mode corresponding to CF_3 rotation that we were unable to eliminate), and therefore the TS-S7 energy shown in brackets is of a different conformer.

Preliminary Computational Investigation of the Thermodynamics of the Reaction Post Decarboxylation:

We propose that simultaneous control of vicinal stereocenters can occur either via an irreversible Michael addition or a reversible C–C bond formation coupled to a subsequent irreversible process. We began by investigating the energetics of the Michael addition (Figure S4). Computationally, we found that the Michael addition of Pd enolate **22** to form spirocycle **23a** proceeds with a $\Delta G^{\ddagger}$ of 7.4 kcal/mol. Further isomerization of Pd-bound ketone **23a** to Pd enolate **24** releases an additional 16.2 kcal/mol of energy (Figure S4), leading to an overall exothermic process with a ΔG of –12.5 kcal/mol from intermediate **22**. The reverse process displays a 19.9 kcal/mol energy barrier, which is likely still achievable at the reaction temperature. Hence, we began to study select potential mechanisms for the subsequent proton transfer process. Among the pathways studied (Figure S3), an *N*-detached inner-sphere pathway was found to be the most energetically favorable, with $\Delta G^{\ddagger}$ of 5.6 kcal/mol and ΔG of –9.5 kcal/mol. However, further computational investigation of the isomerizations of Pd intermediates **23a**, **24**, and **26** (along with all corresponding stereoisomers such as **23b**–**d**, etc.) is required to determine whether these isomerizations are important to understanding the thermodynamics/kinetics of the overall reaction. These areas are currently being investigated by our group and will be reported in due course.

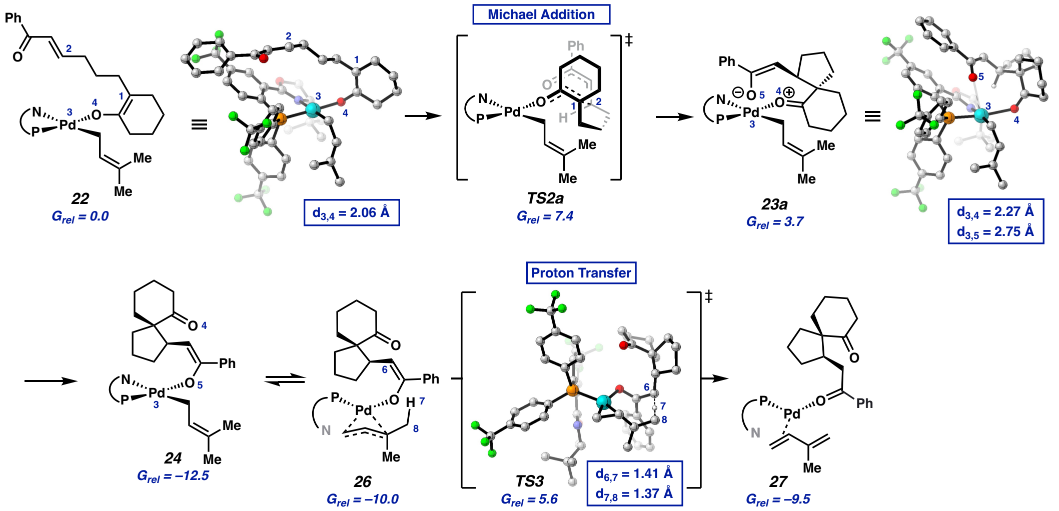

Figure S4. Proposed reaction pathway for Michael addition and proton transfer and the computed relative energies of relevant intermediates and transition states. Gibbs free energies in kcal/mol computed at the PBE0-D4/BS2/CPCM(PhMe)//PBE0-D4/BS1/CPCM(PhMe) level of theory at 313.15 K.

2D NMR Analysis of Select Compounds:

Compound **13**

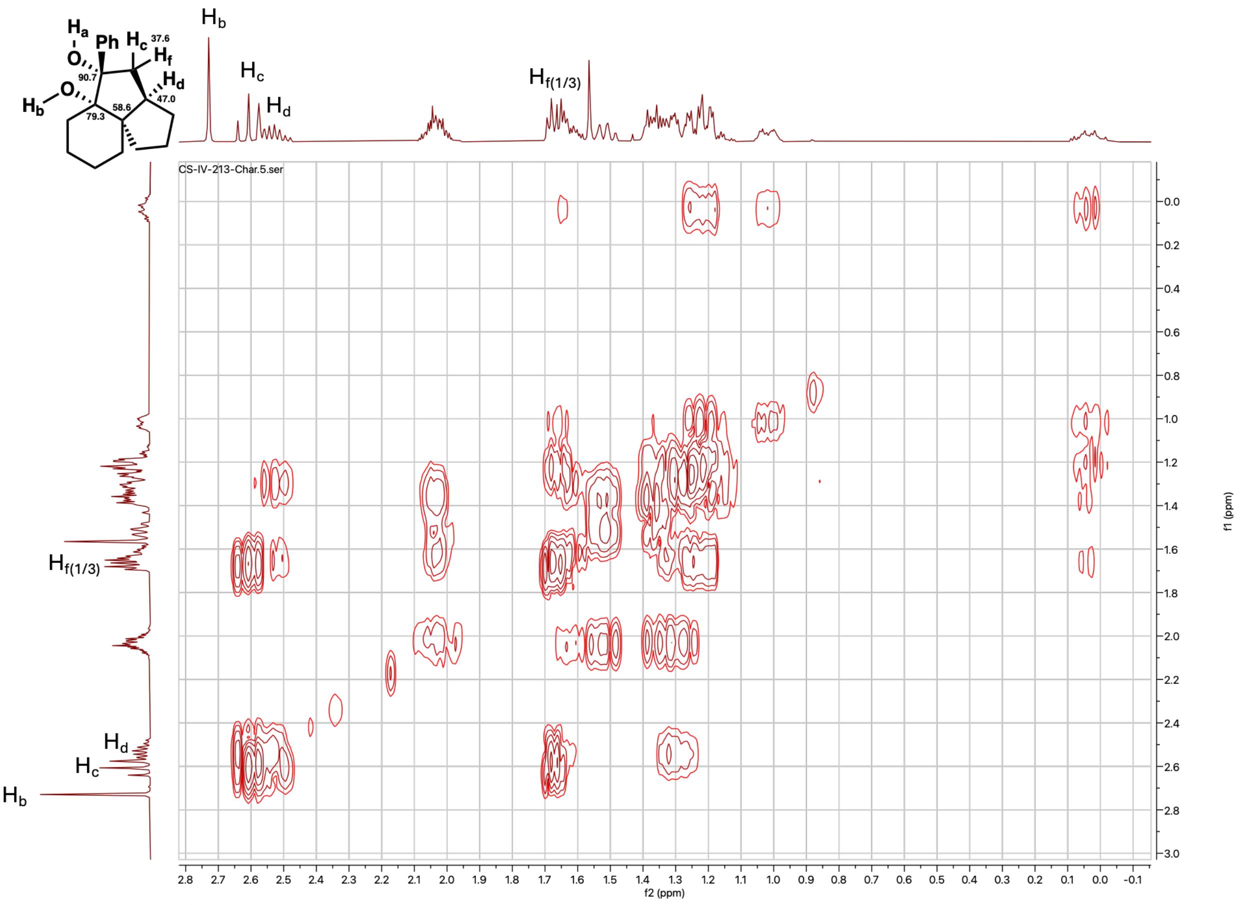

Figure S5. ^{1}H–^{1}H COSY NMR spectrum of **13** (400 MHz, $CDCl_3$)

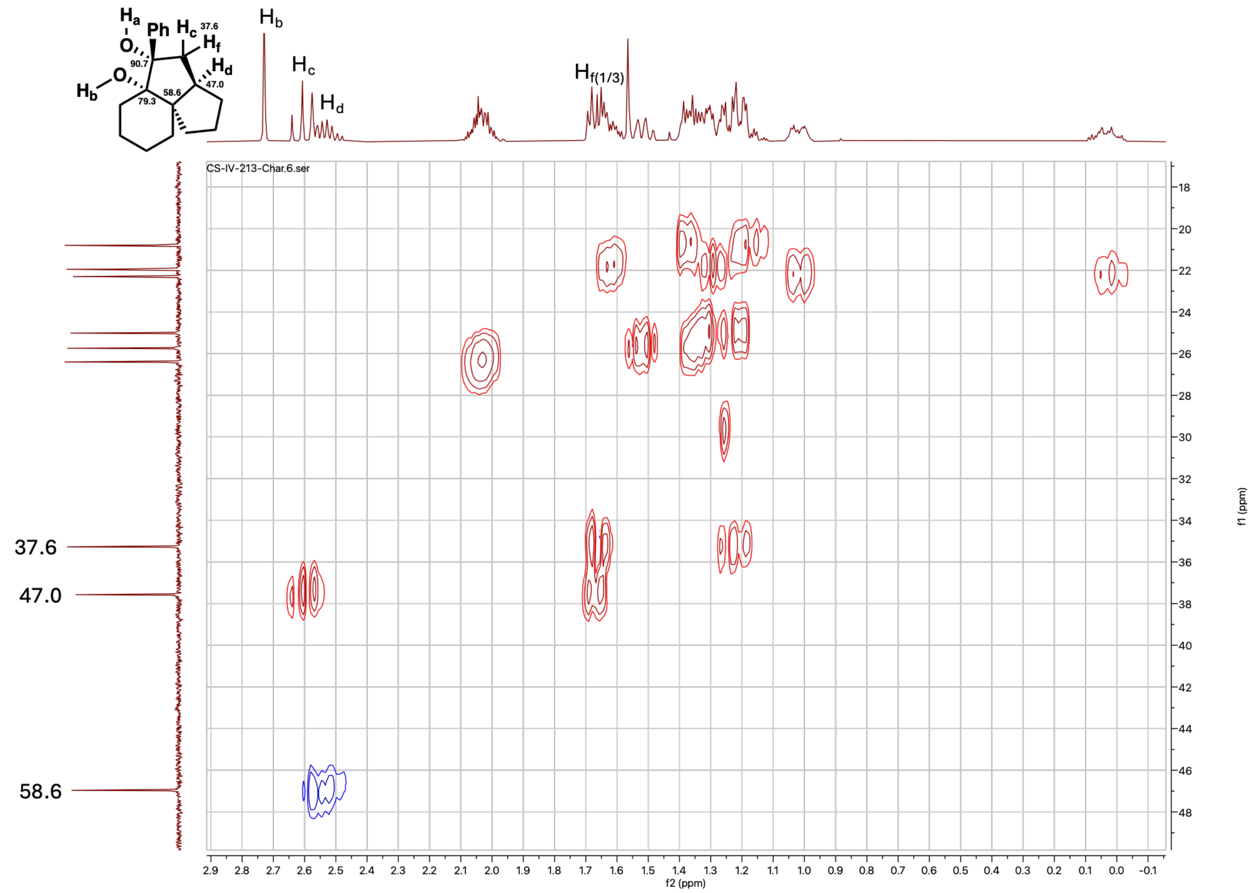

Figure S6. ^{1}H–^{13}C HSQC NMR spectrum of **13** (400 MHz, $CDCl_3$)

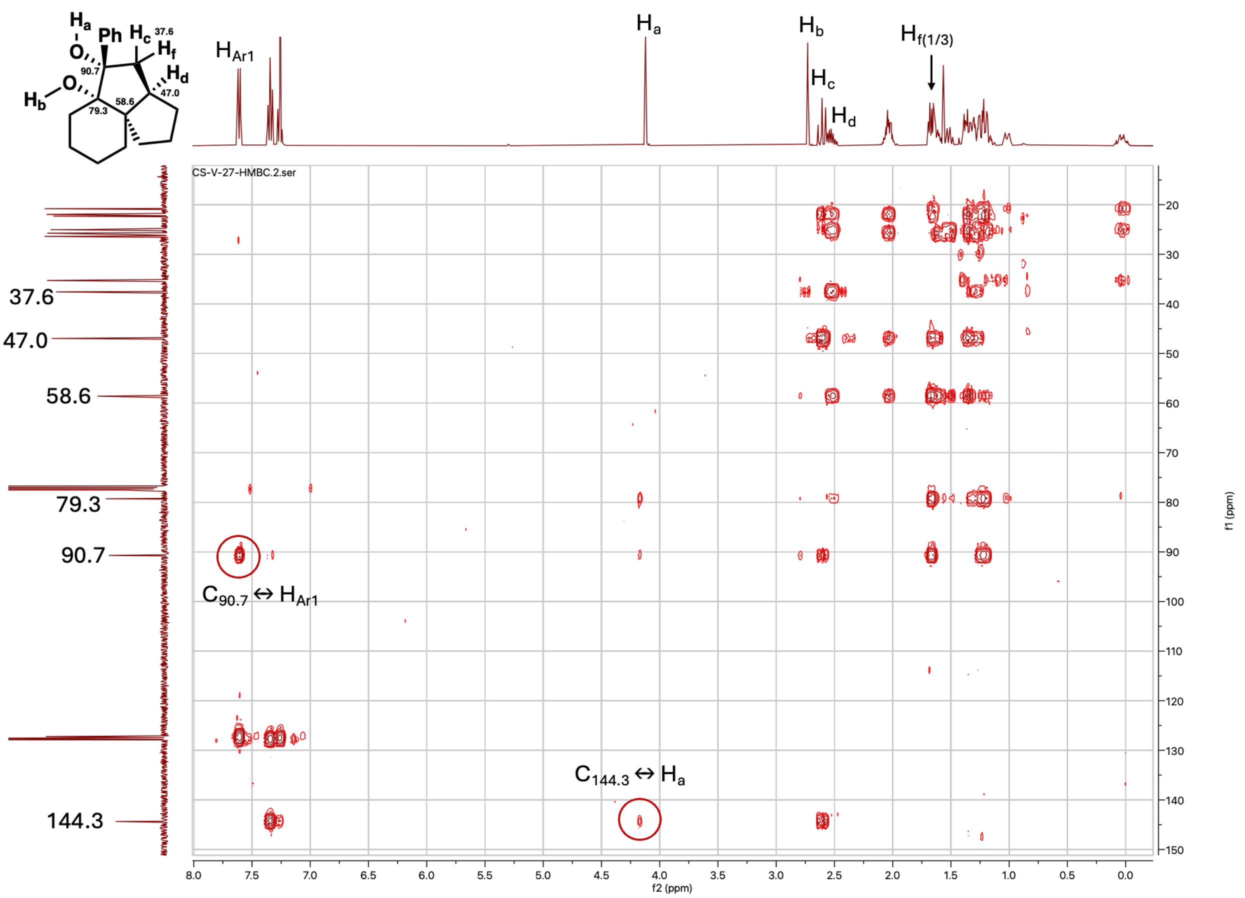

Figure S7. ^{1}H–^{13}C HMBC NMR spectrum of **13** (400 MHz, $CDCl_3$)

Compound **17**

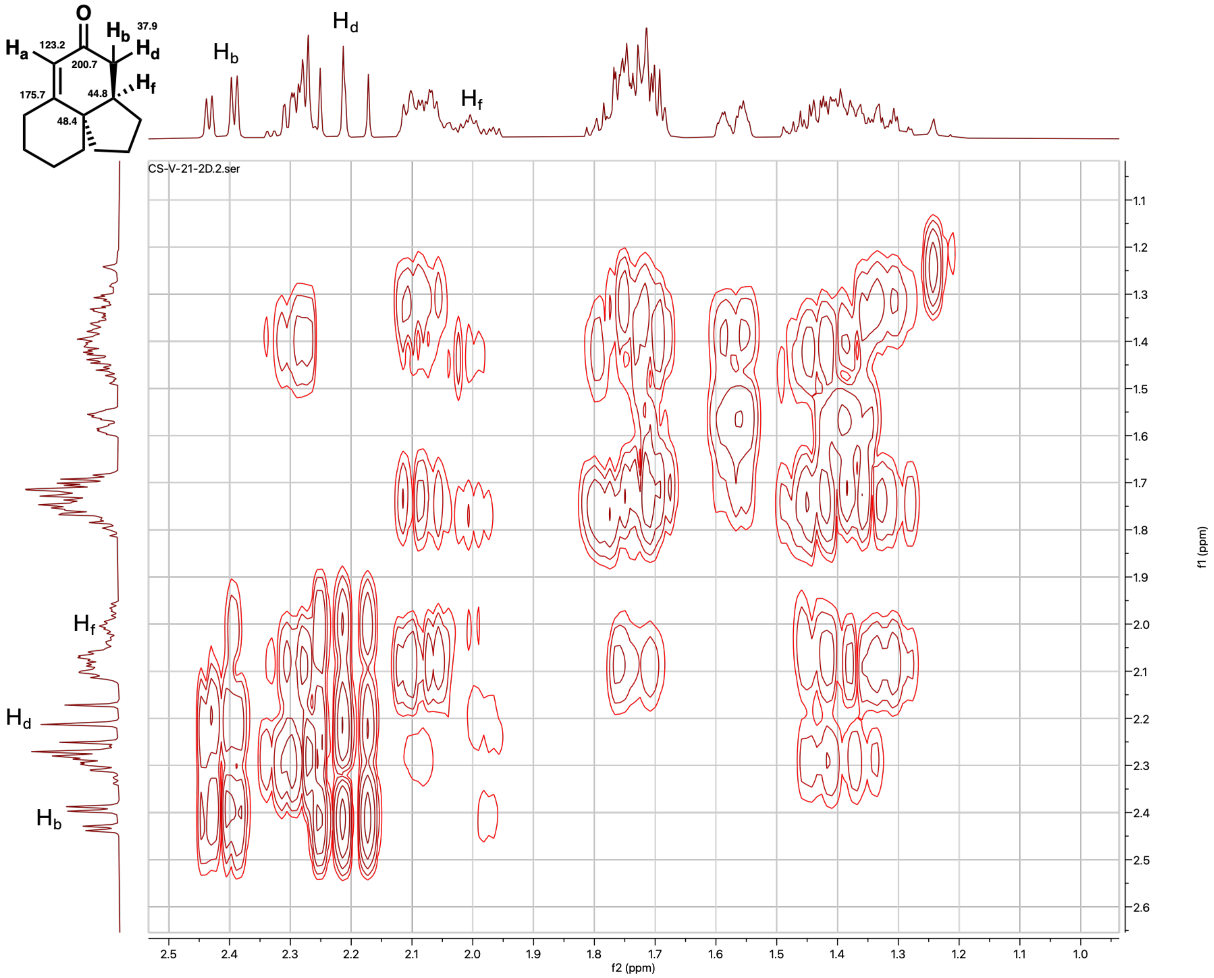

Figure S8. ^1H–^1H COSY NMR spectrum of **17** (400 MHz, $CDCl_3$)

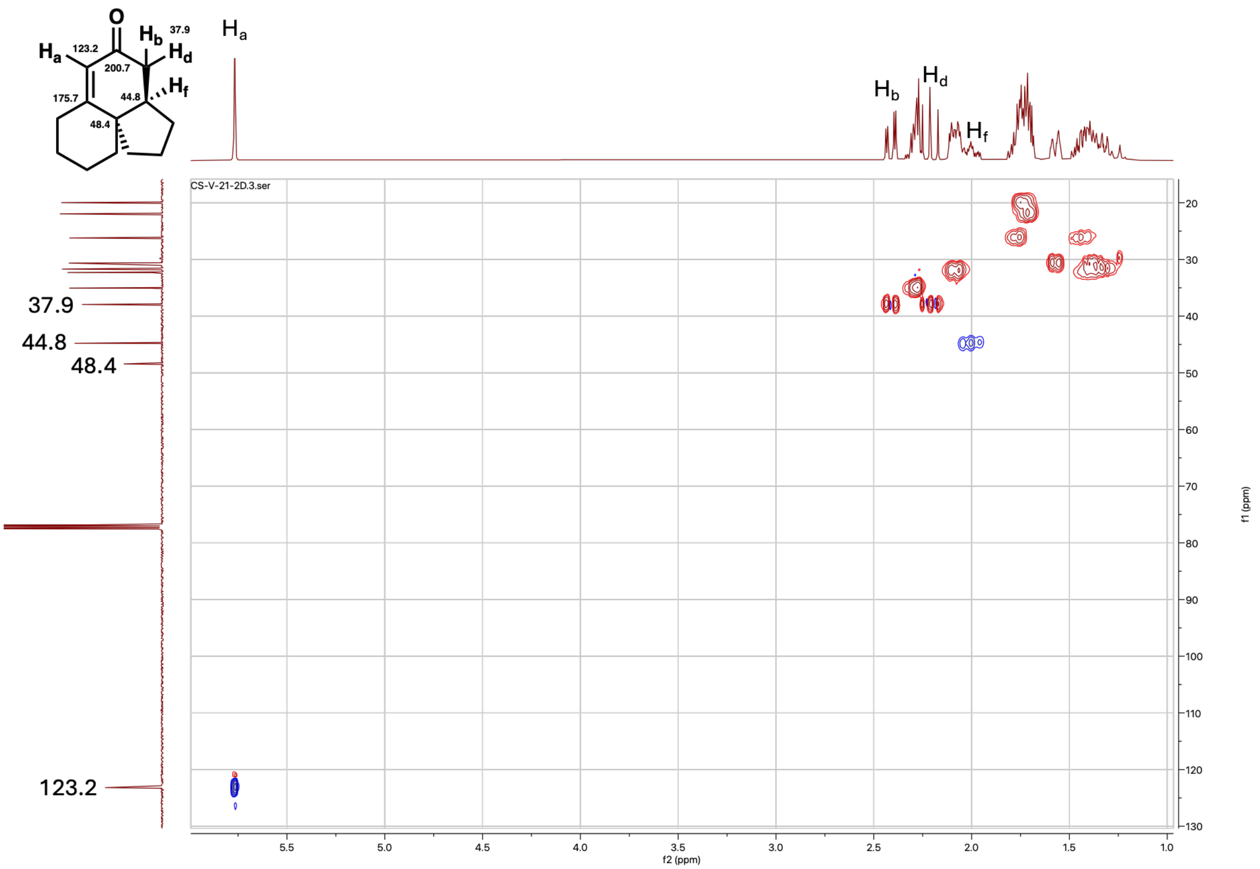

Figure S9. ^{1}H–^{13}C HSQC NMR spectrum of **17** (400 MHz, $CDCl_3$)

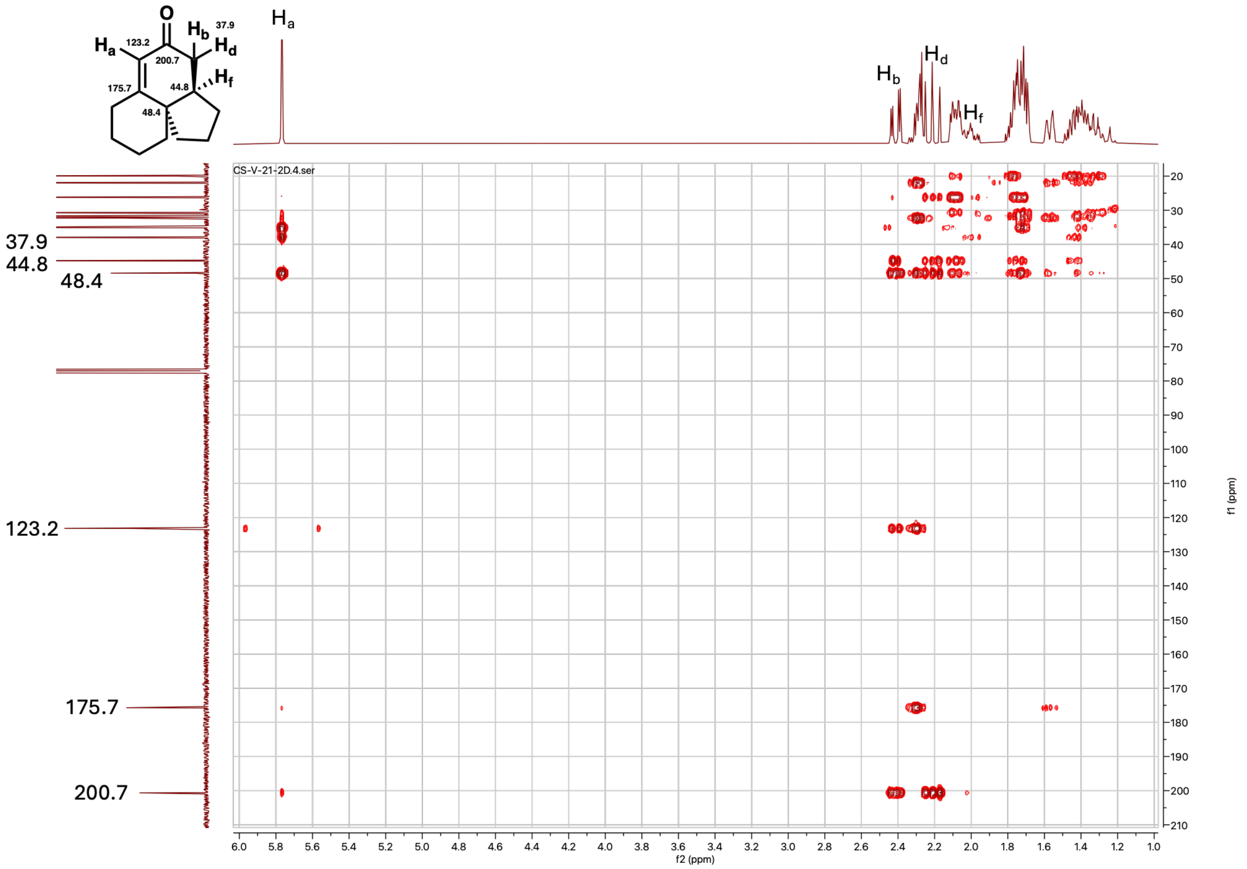

Figure S10. ^{1}H–^{13}C HMBC NMR spectrum of **17** (400 MHz, $CDCl_3$)

Preliminary Experimental Mechanistic Investigations:

^{31}P NMR Studies and ^{1}H Time course:

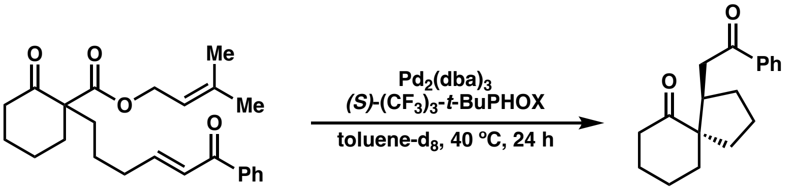

7a **8a**

NMR Sample preparation ^{31}P NMR monitoring and ^{1}H time course:

In a nitrogen filled glovebox, an oven-dried 4 mL vial was charged with a stir bar, $Pd_2(dba)_3$ (1.4 mg, 0.0015 mmol, 2.5 mol %), *(S)*-$(CF_3)_3$-*t*-BuPHOX (2.3 mg, 0.0039 mmol, 6.5 mol %), and toluene-d_8 (0.8 mL). The catalyst solution was stirred at 23 ºC for 20 min. A solution of substrate **7** (0.06 mmol, 1 equiv) in toluene-d_8 (0.2 mL) was prepared and added to an NMR tube with a threaded top. To this solution was added 0.4 mL of the catalyst solution before the tube was sealed with a Teflon-lined lid and brought to the NMR instrument. At 25 ºC, initial ^{31}P and ^{1}H NMRs were taken of this mixture followed by additional ^{31}P and ^{1}H NMR spectra taken upon heating to 40 ºC. ^{1}H NMR spectra were taken (at 40 ºC) every 5 minutes for 1 hour followed by an additional ^{31}P NMR spectrum. ^{1}H NMR spectra were then taken (at 40 ºC) every 5 minutes for an additional ~4 hours. The reaction was then heated overnight at 40 ºC in an oil bath. A final ^{31}P and ^{1}H NMRs were taken at 25 ºC following completion of the reaction (after 24 hours). Purification by flash column chromatography (0–60% Et_2O/hexanes) afforded the title compound as a white crystalline solid (9.1 mg, 0.034 mmol, 56% yield, 93% ee). The full characterization for this material is above.

In a separate NMR tube, the remaining 0.4 mL of catalyst solution prepared above were added followed by 0.2 mL of toluene- d_8. At 25 ºC, ^{31}P and ^{1}H NMR were taken of this mixture.

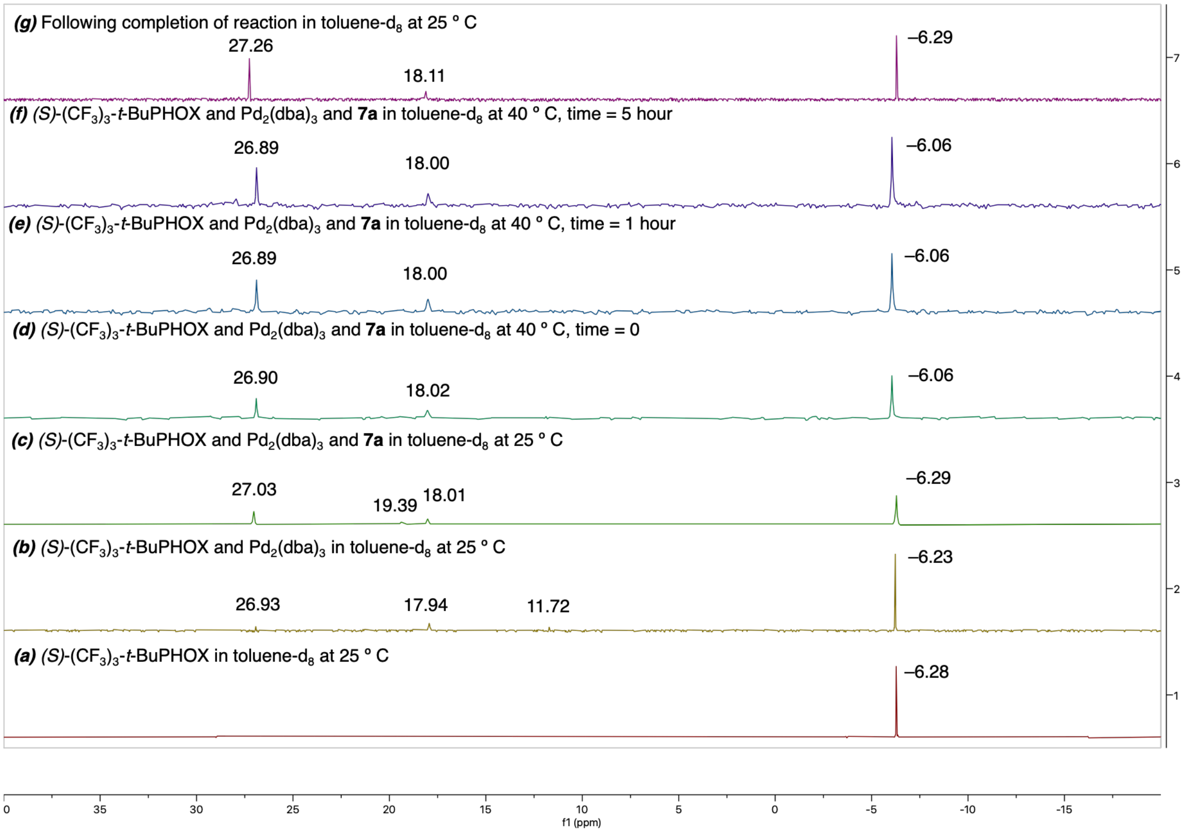

Figure S11. ^{31}P spectra of various relevant mixtures (400 MHz, toluene-d$_8$).

This displays the peaks observed in the ^{31}P NMR spectra of various species and reaction mixtures throughout the course of the reaction. Apparently, *(S)*-$(CF_3)_3$-*t*-BuPHOX alone in toluene-d$_8$ appears at –6.28 ppm as a sharp singlet (spectrum a). Upon formation of the precatalyst solution a similar peak is observed (at –6.23 ppm) which is logical considering the excess stoichiometry of ligand (spectrum b). A peak with a similar chemical shift is observed throughout the duration of the experiment. Three new species are observed at 11.72, 17.94, and 26.93 ppm respectively. Following addition of substrate **7a** and at 25 ºC, the peak around 11.72 ppm is no longer observable although a new broad peak is observed at 19.39 ppm (spectrum c). When this mixture is heated to 40 ºC the signal believed to correspond to free ligand shifts modestly to –6.06 ppm whereas the two species around 18.02 and 26.90 ppm persist (spectrum d). At 40 ºC and over 5 hours the relative intensity of the species around 26.89 ppm appears to grow in slightly (spectra e and f). Following completion of the reaction at 40 ºC it was cooled to 25 ºC to give spectrum g which shows three similar signals as previous spectra.

Further experimentation is required to elucidate the structures of these species and determine their relevance to catalysis. This is an active area of investigation in our group and will be reported in due course.

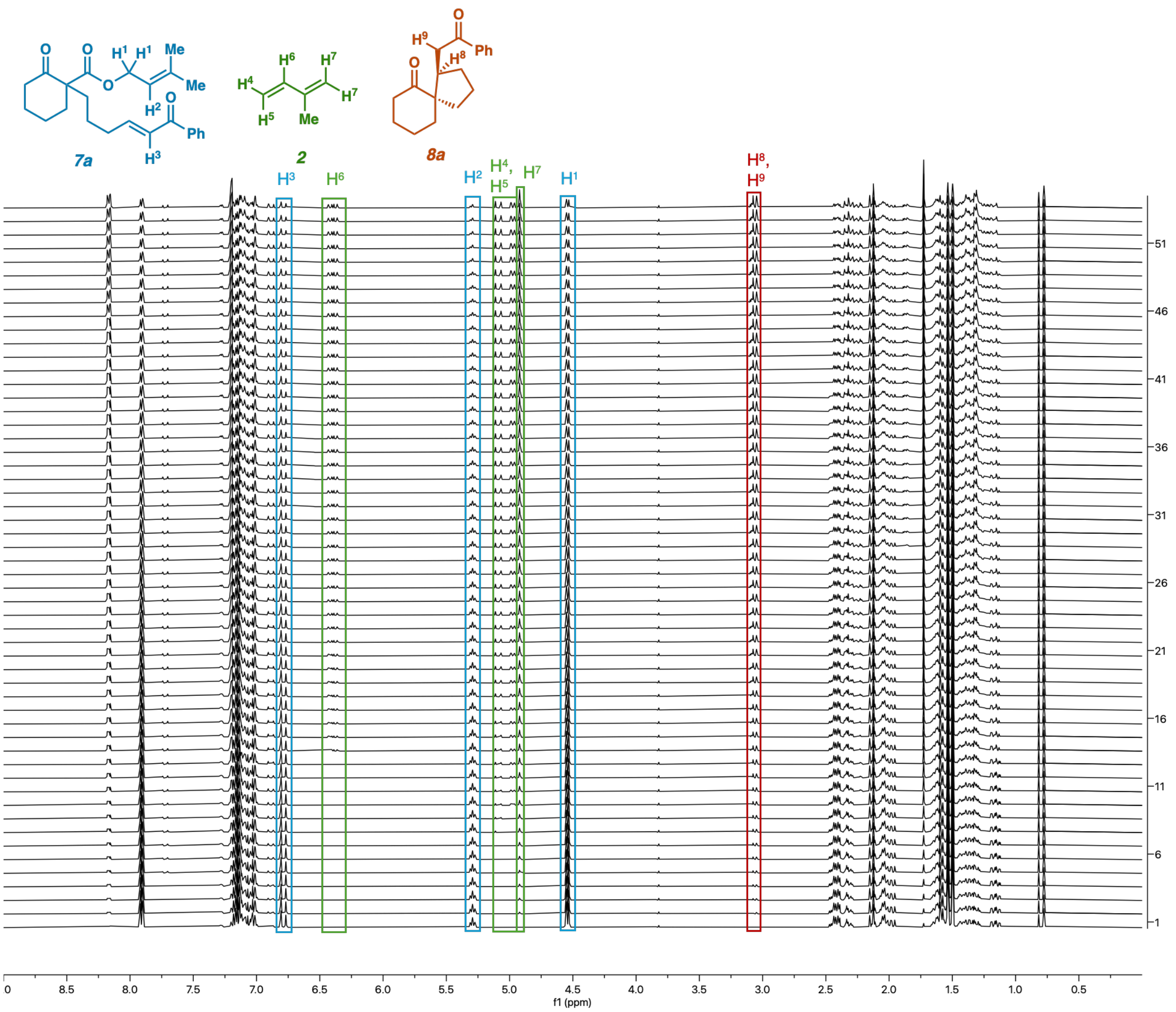

Figure S12. ^{1}H spectra timecourse over ~ 5 hours (400 MHz, toluene-d_8).

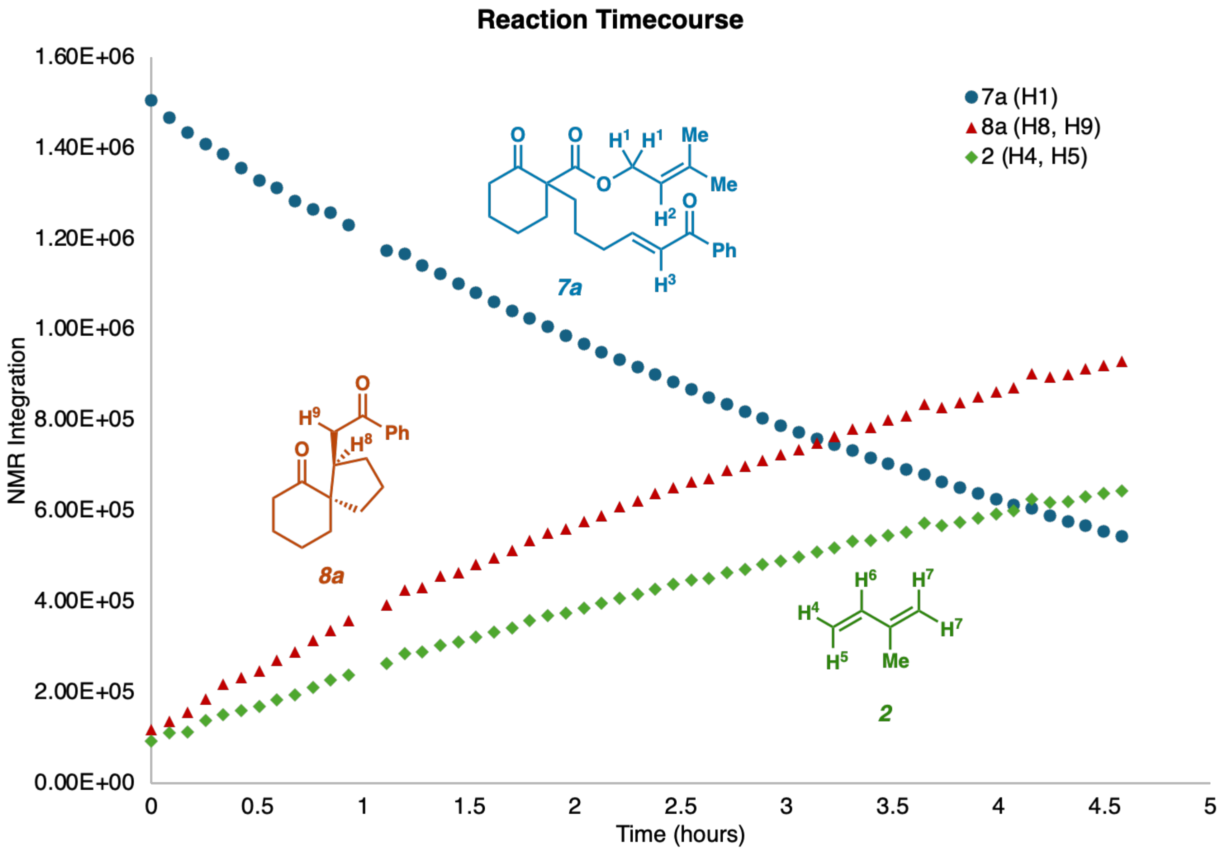

Figure S13. ^{1}H NMR timecourse plot of integration vs time.

These data (presented in Figure S12 and S13) display the evolution of products from this reaction over time as monitored by proton NMR. These data present the time points at 40 ºC that were taken approximately every 5 minutes. It is worth noting that minimal conversion does occur at 25 ºC, although it is quite sluggish. A set of two protons was selected for each of the three major species involved during the course of this reaction. For **7a**, the allylic protons H1 were utilized. For **8a**, the methine H8 and one of the protons α to the phenyl ketone, H9, was used. Finally, for isoprene two vinylic protons, H4 and H5 were used. The deflated integration of isoprene's vinylic protons might be due to it evaporating into the headspace during the reaction (boiling point = 34 ºC). Further studies related to elucidating the mechanism of this reaction and its kinetic profile are currently ongoing.

References:

(1) (a) Krout, M. R.; Mohr, J. T.; Stoltz, B. M. Preparation of (*S*)-*tert*-ButylPHOX (Oxazole, 4-(1,1-dimethylethyl)-2-[2-(diphenylphosphino)phenyl]-4,5- dihydro- (4*S*)-). *Org. Synth.* **2009**, *86*, 181–193. (b) McDougal, N. T.; Streuff, J.; Mukherjee, H.; Virgil, S. C.; Stoltz, B. M. Rapid synthesis of an electron-deficient *t*-BuPHOX ligand: cross-coupling of aryl bromides with secondary phosphine oxides. *Tetrahedron Lett*. **2010**, *51*, 5550–5554.
(2) Wagen, C. C.; McMinn, S. E.; Kwan, E. E.; Jacobsen, E. N. Screening for Generality in Asymmetric Catalysis. *Nature*, **2022**, *610*, 680–686.
(3) Sheldrick, G. M. *Acta Cryst.* **1990**, A46, 467-473.
(4) Sheldrick, G. M. *Acta Cryst.* **2015**, C71, 3-8.
(5) Müller, P. *Crystallography Reviews* **2009**, *15*, 57-83.
(6) Milburn, R. R; McRae, K.; Chan, Johann, Tedrow, J.; Larsen, R.; Faul, M. A practical preparation of aryl β-ketophosphonates. *Tetrahedron Lett.* **2009**, *50*, 870–872.
(7) Sano, S.; Okubo, Y.; Hanada, A.; Nakao, M.; Kitaike, S.; Nagao, Y.; Nakegawa, H. Reinvestigation of the Synthesis of Isoliquiritigenin: Application of Horner–Wadsworth–Emmons Reaction and Claisen–Schmidt Condensation. *Chem. Pharm. Bull.* **2011**, *59*, 885–888.
(8) Murai, M.; Nakamura, M.; Takai, K. Rhenium-Catalyzed Synthesis of 2H-1,2-Oxaphosphorin 2-Oxides via the Regio- and Stereoselective Addition Reaction of β-Keto Phosphonates with Alkynes. *Org. Lett.* **2014**, *16*, 5784–5787.
(9) Trost, B. M.; Xu, J. The O-Acylation of Ketone Enolates by Allyl 1H-Imidazole-1-carboxylate Mediated with Boron Trifluoride Etherates–A Convenient Procedure for the Synthesis of Substituted Allyl Enol Carbonates. *J. Org. Chem.* **2007**, *72*, 9372–9375.
(10) Inanaga, K.; Wollenburg, M.; Bachman, S.; Hafeman, N. J.; Stoltz, B. M. Catalytic Enantioselective Synthesis of Carbocyclic and Heterocyclic Spiranes via a Decarboxylative Aldol Cyclization. *Chem. Sci.* **2020**, *11*, 7390–7395.
(11) Behenna, D. C.; Liu, Y.; Yurino, T.; Kim, J.; White, D. E.; Virgil, S. C.; Stoltz, B. M. Enantioselective Construction of Quaternary N-heterocycles by Palladium-Catalysed Decarboxylative Allylic Alkylation of Lactams. *Nat. Chem.* **2012**, *4*, 130–133.
(12) Kawato, Y.; Takahashi, N.; Kumagai, N.; Shibasaki, M. Catalytic Asymmetric Conjugate Addition of α-Cyanoketones for the Construction of a Quaternary Stereogenic Center. *Org. Lett.* **2010**, *12*, 1484–1487.
(13) Behenna, D. C.; Mohr, J. T.; Sherden N. H.; Marinescu, S. C., Harned, A. M.; Tani, K.; Seto, M.; Ma, S.; Novák, Z.; Krout, M. R.; McFadden, R. M.; Roizen, J. L.; Enquist jr.; J. A.; White, D. E.; Levine, S. R.; Petrova, K. V.; Iwashita, A.; Virgil, S. C.; Stoltz, B. M. Enantioselective Decarboxylative Alkylation Reactions: Catalyst Development, Substrate Scope, and Mechanistic Studies. *Chem. Eur. J.* **2011**, *17*, 14199–14223.
(14) (a) Neese, F. Software Update: The ORCA Program System, Version 5.0. *Wiley Interdiscip. Rev.: Comput. Mol. Sci.* **2022,** *12*, No. e1606. (b) Neese, F. The ORCA Program System. *Wiley Interdiscip. Rev.: Comput. Mol. Sci.* **2012**, *2*, 73−78.
(15) Adamo, C.; Barone, V. Toward Reliable Density Functional Methods without Adjustable Parameters: The PBE0 Model. *J. Chem. Phys.* **1999**, *110*, 6158−6170.
(16) (a) Caldeweyher, E.; Ehlert, S.; Hansen, A.; Neugebauer, H.; Spicher, S.; Bannwarth, C.; Grimme, S. A Generally Applicable Atomic-Charge Dependent London Dispersion Correction. *J.*

Chem. Phys. **2019**, *150* (15), 154122. (b) Caldeweyher, E.; Bannwarth, C.; Grimme, S. Extension of the D3 Dispersion Coefficient Model. *J. Chem. Phys.* **2017**, *147*, 034112.

(17) Weigend, F.; Ahlrichs, R. Balanced Basis Sets of Split Valence, Triple Zeta Valence and Quadruple Zeta Valence Quality for H to Rn: Design and Assessment of Accuracy. *Phys. Chem. Chem. Phys.* **2005**, *7*, 3297–3305.

(18) Peterson, K. A.; Figgen, D.; Goll, E.; Stoll, H.; Dolg, M. Systematically Convergent Basis Sets with Relativistic Pseudopotentials. II. Small-Core Pseudopotentials and Correlation Consistent Basis Sets for the Post- *d* Group 16–18 Elements. *The Journal of Chemical Physics* **2003**, *119*, 11113–11123.

(19) (A) Cusumano, A. Q.; Goddard, W. A. I.; Stoltz, B. M. The Transition Metal Catalyzed [π2s + π2s + s2s + s2s] Pericyclic Reaction: Woodward–Hoffmann Rules, Aromaticity, and Electron Flow. *J. Am. Chem. Soc.* **2020**, *142*, 19033–19039. (B) Cusumano, A. Q.; Stoltz, B. M.; Goddard, W. A. Reaction Mechanism, Origins of Enantioselectivity, and Reactivity Trends in Asymmetric Allylic Alkylation: A Comprehensive Quantum Mechanics Investigation of a C(sp^3)–C(sp^3) Cross-Coupling. *J. Am. Chem. Soc.* **2020**, *142*, 13917–13933. (C) Flesch, K. N.; Cusumano, A. Q.; Chen, P.-J.; Strong, C. S.; Sardini, S. R.; Du, Y. E.; Bartberger, M. D.; Goddard, W. A.; Stoltz, B. M. Divergent Catalysis: Catalytic Asymmetric [4+2] Cycloaddition of Palladium Enolates. *J. Am. Chem. Soc.* **2023**, *145*, 11301–11310.

(20) Garcia-Ratés, M.; Neese, F. Effect of the Solute Cavity on the Solvation Energy and Its Derivatives within the Framework of the Gaussian Charge Scheme. *J. Comput. Chem.* **2020**, *41*, 922–939.

(21) Ishida, K.; Morokuma, K.; Komornicki, A. The Intrinsic Reaction Coordinate. An Ab Initio Calculation for HNC→HCN and H−+CH4→CH4+H−. *J. Chem. Phys.* **1977**, *66 (5)*, 2153−2156.

(22) Zhao, Y.; Truhlar, D. G. The M06 Suite of Density Functionals for Main Group Thermochemistry, Thermochemical Kinetics, Noncovalent Interactions, Excited States, and Transition Elements: Two New Functionals and Systematic Testing of Four M06-Class Functionals and 12 Other Functionals. *Theor Chem Account* **2008**, *120* (1), 215–241.

(23) Grimme, S. Supramolecular Binding Thermodynamics by Dispersion-Corrected Density Functional Theory. *Chem. Eur. J.* **2012**, *18*, 9955–9964.

(24) Izato, Y.; Matsugi, A.; Koshi, M.; Miyake, A. A Simple Heuristic Approach to Estimate the Thermochemistry of Condensed-Phase Molecules Based on the Polarizable Continuum Model. *Phys. Chem. Chem. Phys.* **2019**, *21*, 18920–18929.

(25) Finkelstein, A. V.; Janin, J. The Price of Lost Freedom: Entropy of Bimolecular Complex Formation. *Protein Engineering, Design and Selection* **1989**, *3*, 1–3.

(26) Neese, F.; Wennmohs, F.; Hansen, A.; Becker, U. Efficient, Approximate and Parallel Hartree-Fock and Hybrid DFT Calculations. A 'Chain-of-Spheres' Algorithm for the Hartree-Fock Exchange. *Chemical Physics* **2009**, *356*, 98–109.

(27) Weigend, F. Accurate Coulomb-Fitting Basis Sets for H to Rn. *Phys. Chem. Chem. Phys.* **2006**, *8*, 1057–1065.

(28) Stoychev, G. L.; Auer, A. A.; Neese, F. Automatic Generation of Auxiliary Basis Sets. *J. Chem. Theory Comput.* **2017**, *13*, 554–562.

(29) Grimme, S. Exploration of Chemical Compound, Conformer, and Reaction Space with Meta-Dynamics Simulations Based on Tight-Binding Quantum Chemical Calculations. *J. Chem. Theory Comput.* **2019**, *15*, 2847–2862.

(30) Flesch, K. N.; Cusumano, A. Q.; Chen, P.-J.; Strong, C. S.; Sardini, S. R.; Du, Y. E.; Bartberger, M. D.; Goddard, W. A.; Stoltz, B. M. Divergent Catalysis: Catalytic Asymmetric [4+2] Cycloaddition of Palladium Enolates. *J. Am. Chem. Soc.* **2023**, *145*, 11301–11310.

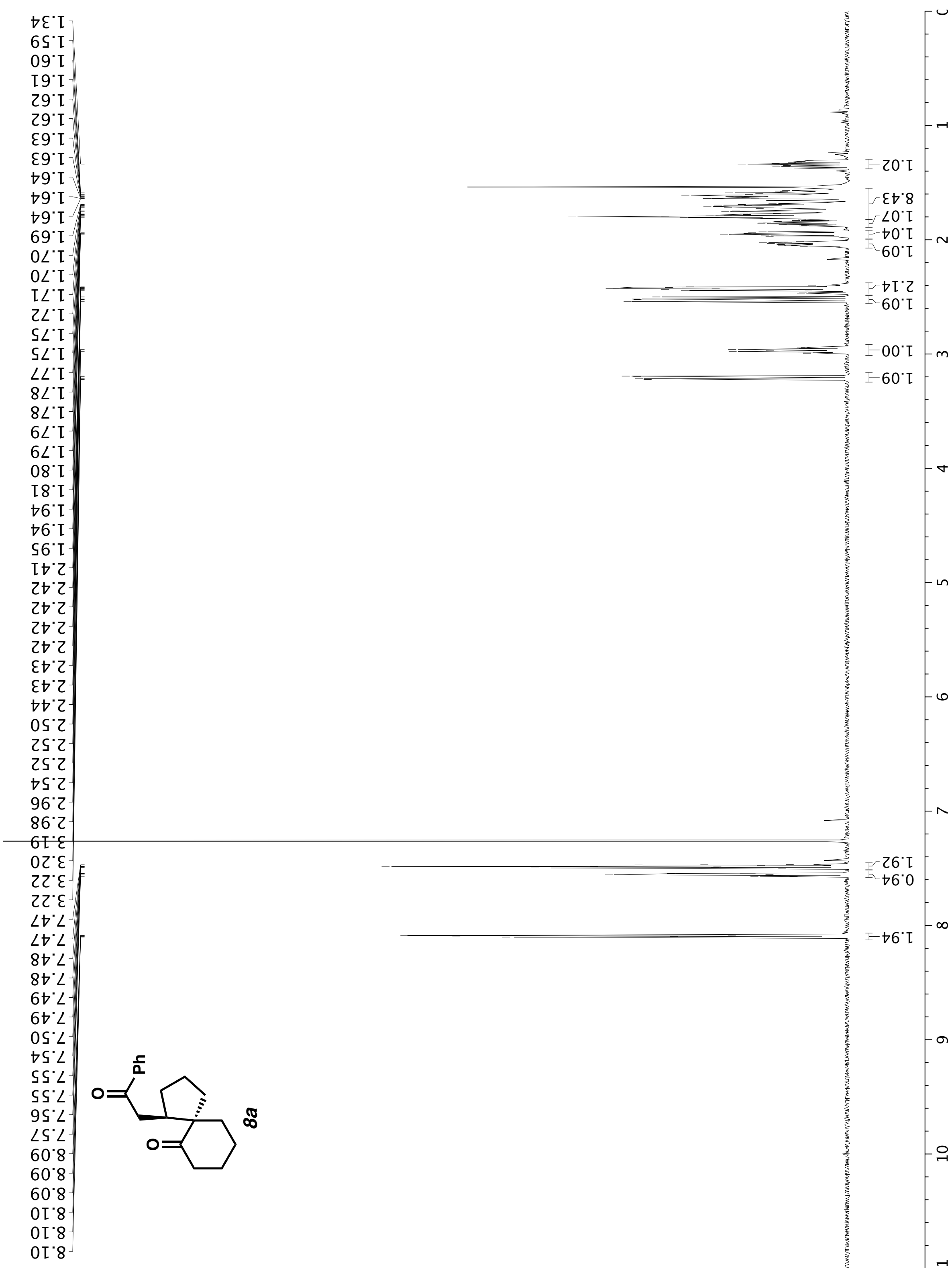

^{1}H NMR (600 MHz, $CDCl_3$) of compound **8a**.

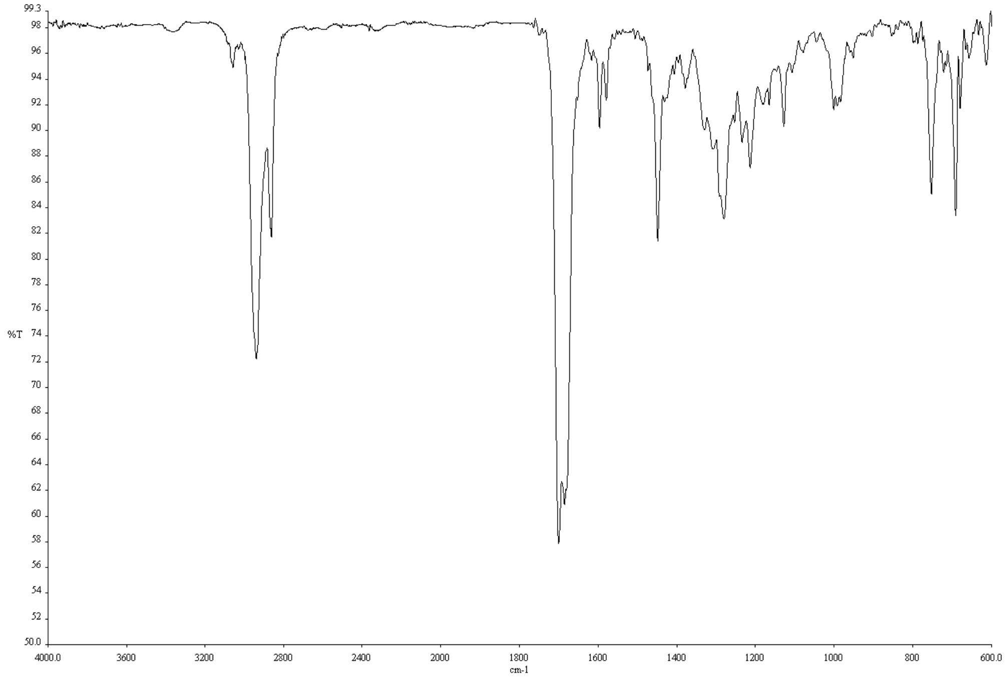

Infrared spectrum (Thin Film, NaCl) of compound **8a**.

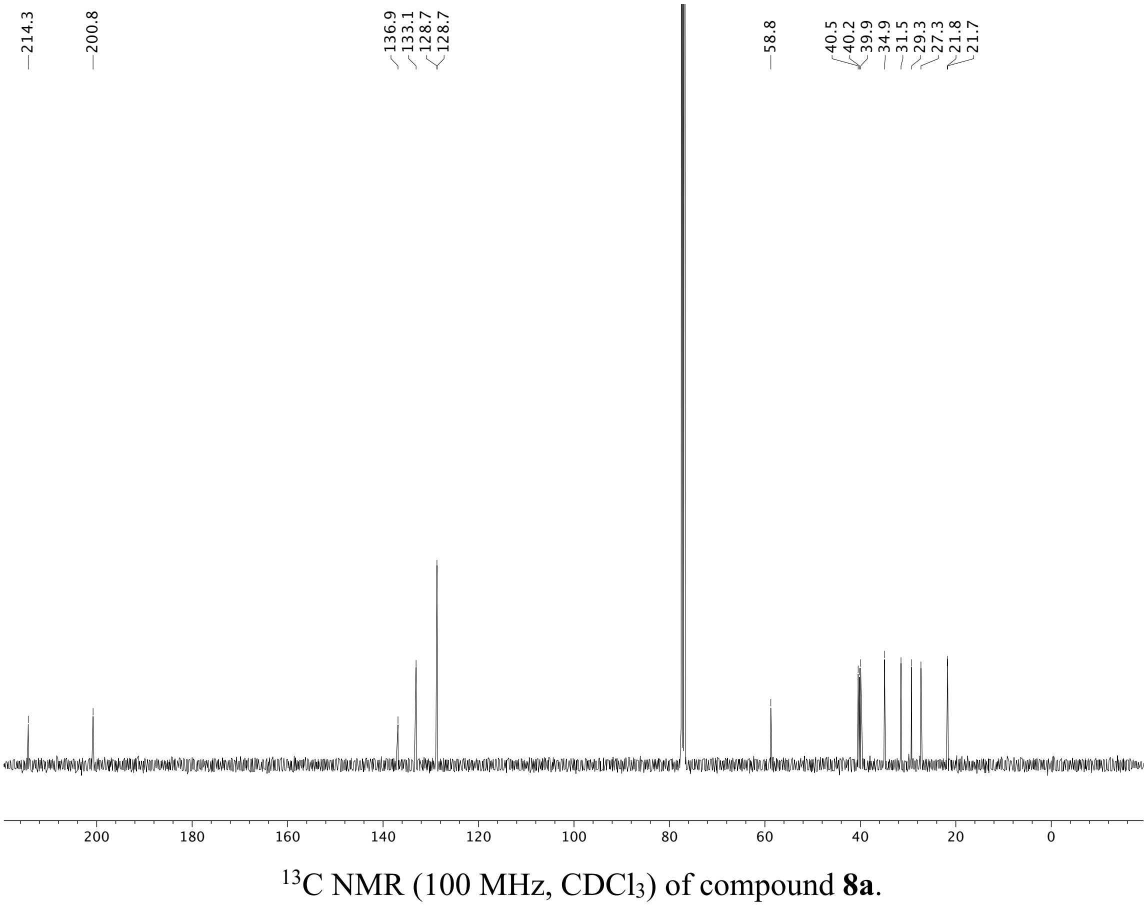

^{13}C NMR (100 MHz, $CDCl_3$) of compound **8a**.

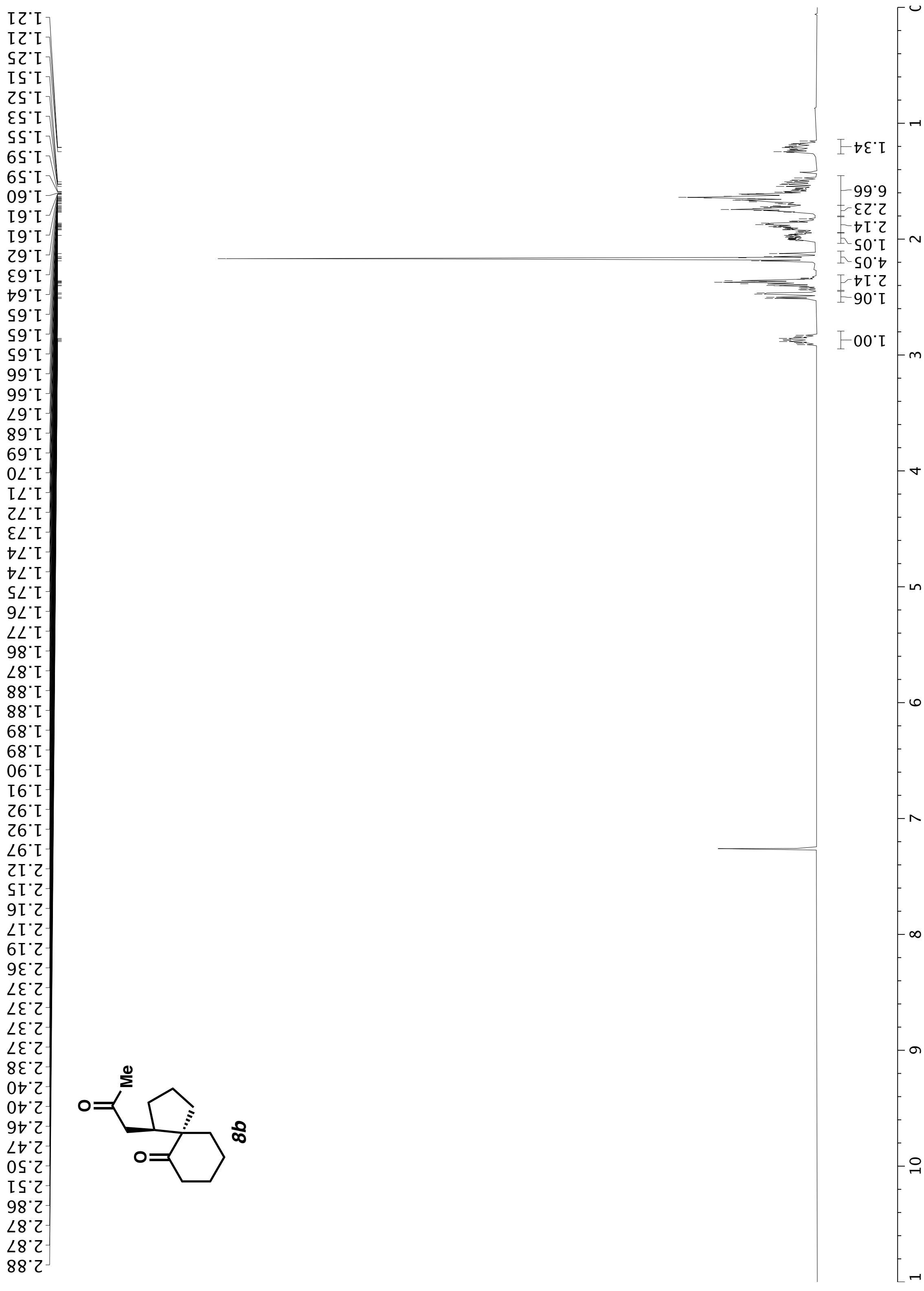

^{1}H NMR (400 MHz, $CDCl_3$) of compound **8b**.

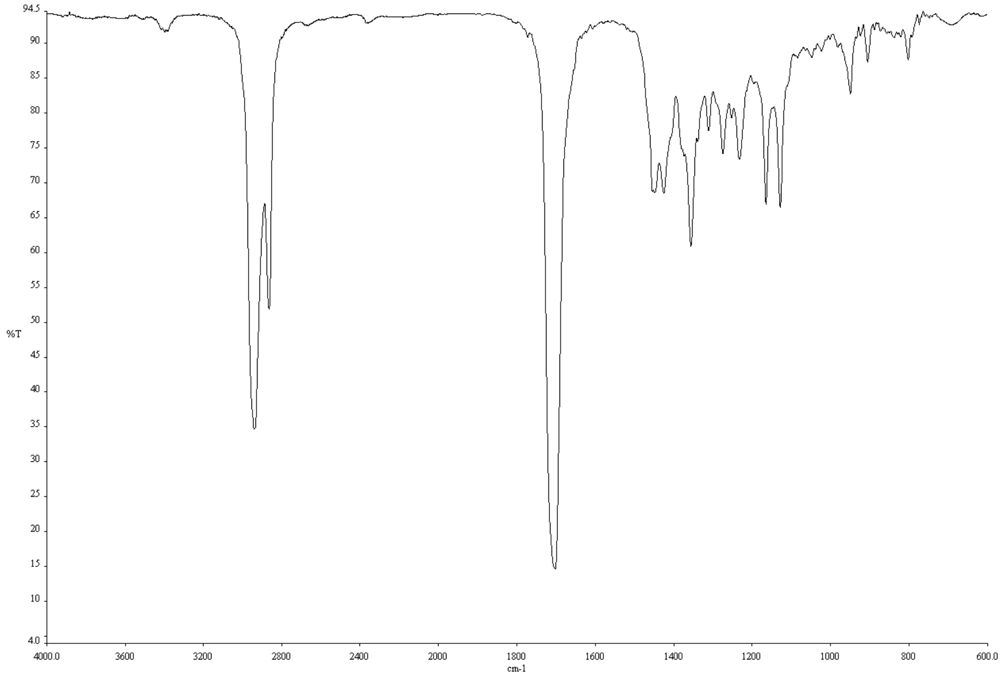

Infrared spectrum (Thin Film, NaCl) of compound **8b**.

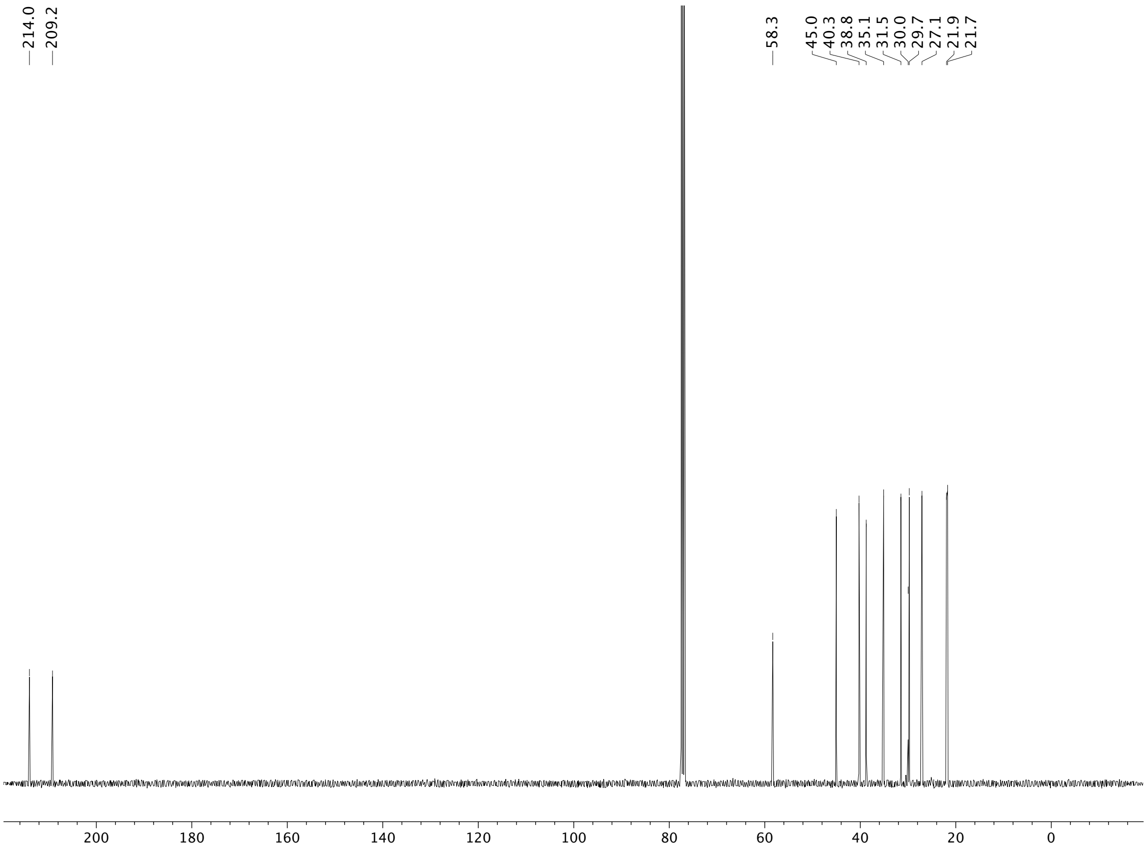

^{13}C NMR (100 MHz, CDCl$_3$) of compound **8b**.

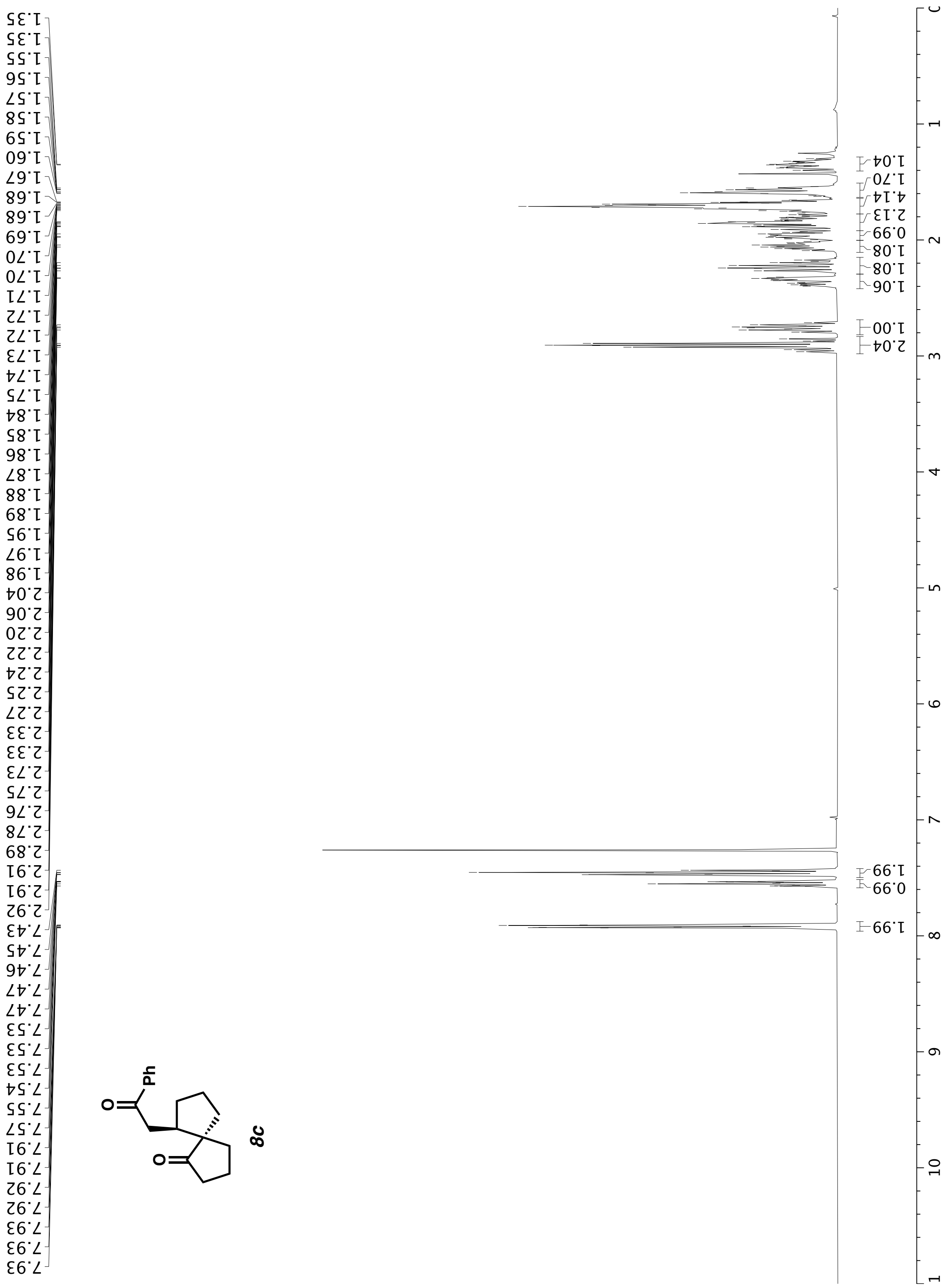

^{1}H NMR (400 MHz, $CDCl_3$) of compound **8c**.

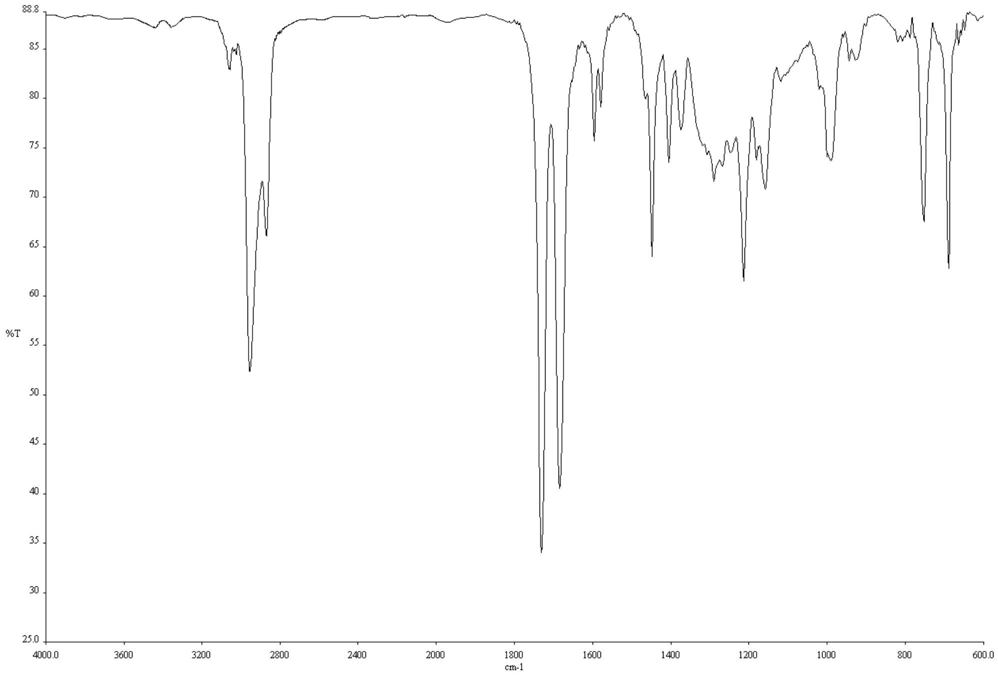

Infrared spectrum (Thin Film, NaCl) of compound **8c**.

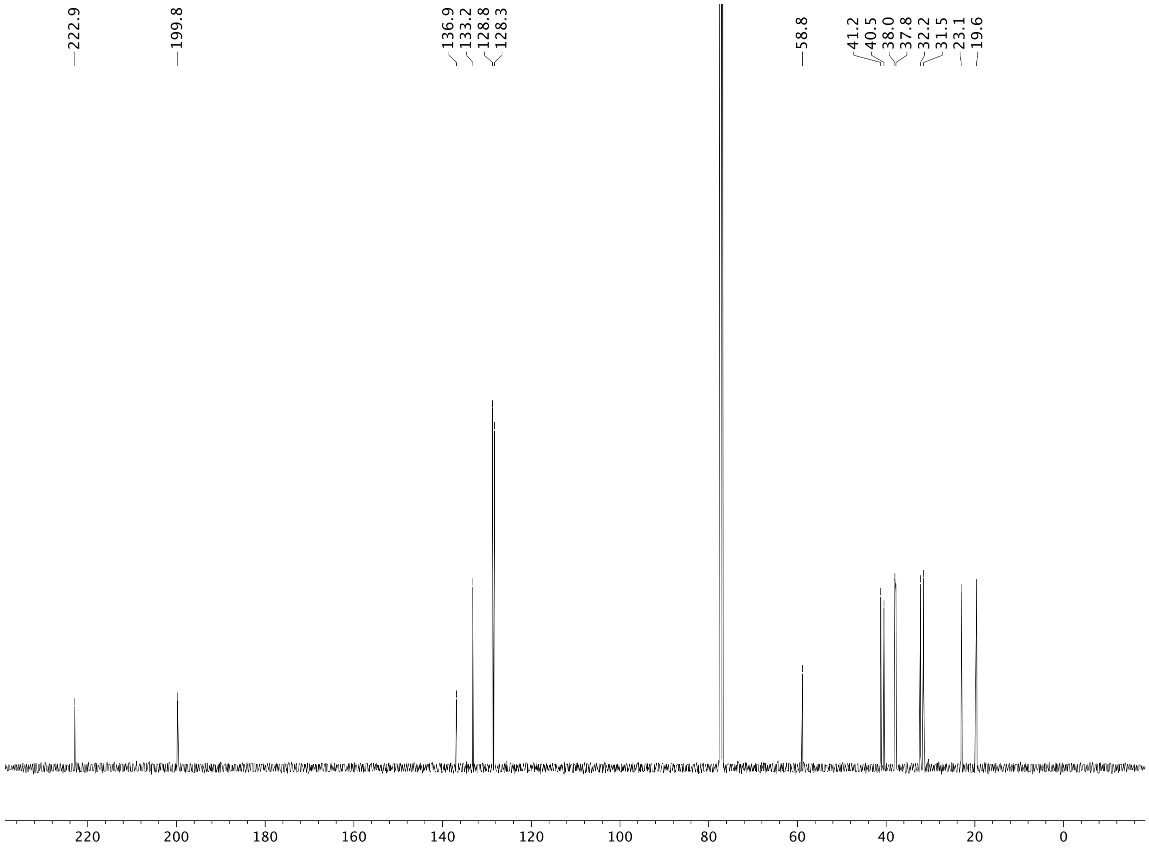

^{13}C NMR (100 MHz, $CDCl_3$) of compound **8c**.

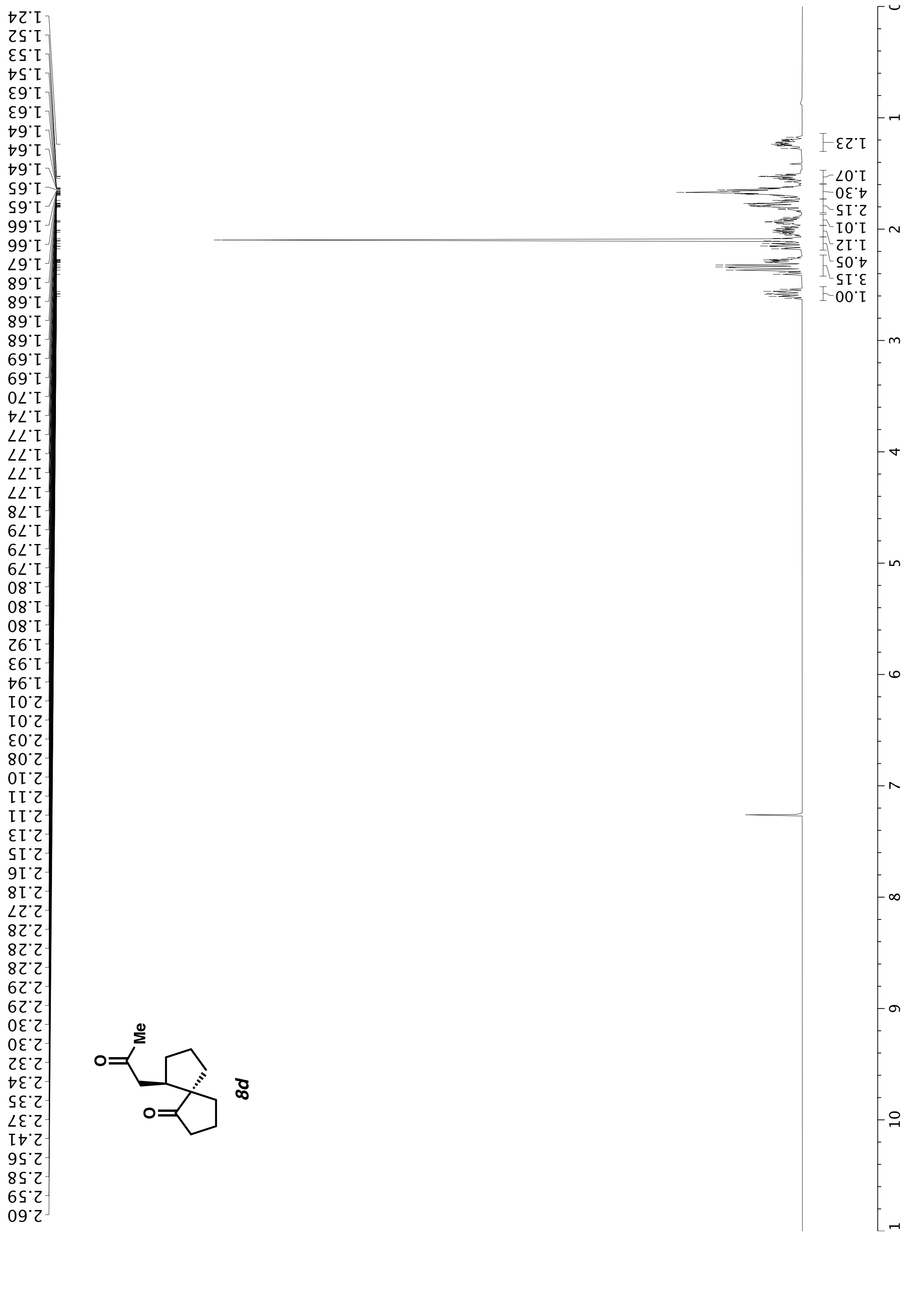

^{1}H NMR (400 MHz, $CDCl_3$) of compound **8d**.

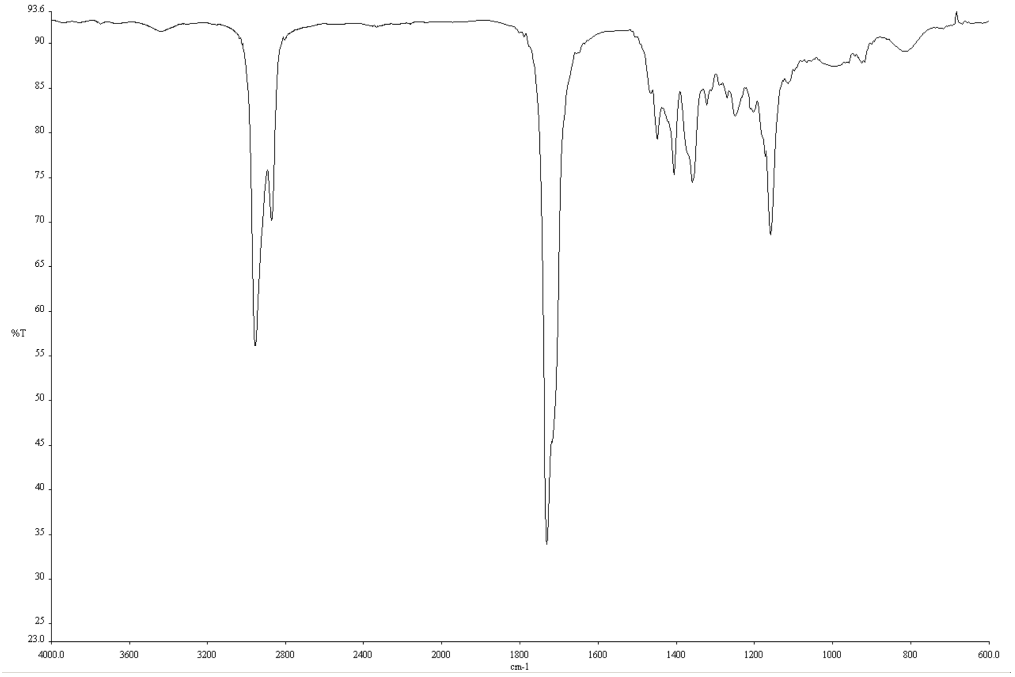

Infrared spectrum (Thin Film, NaCl) of compound **8d**.

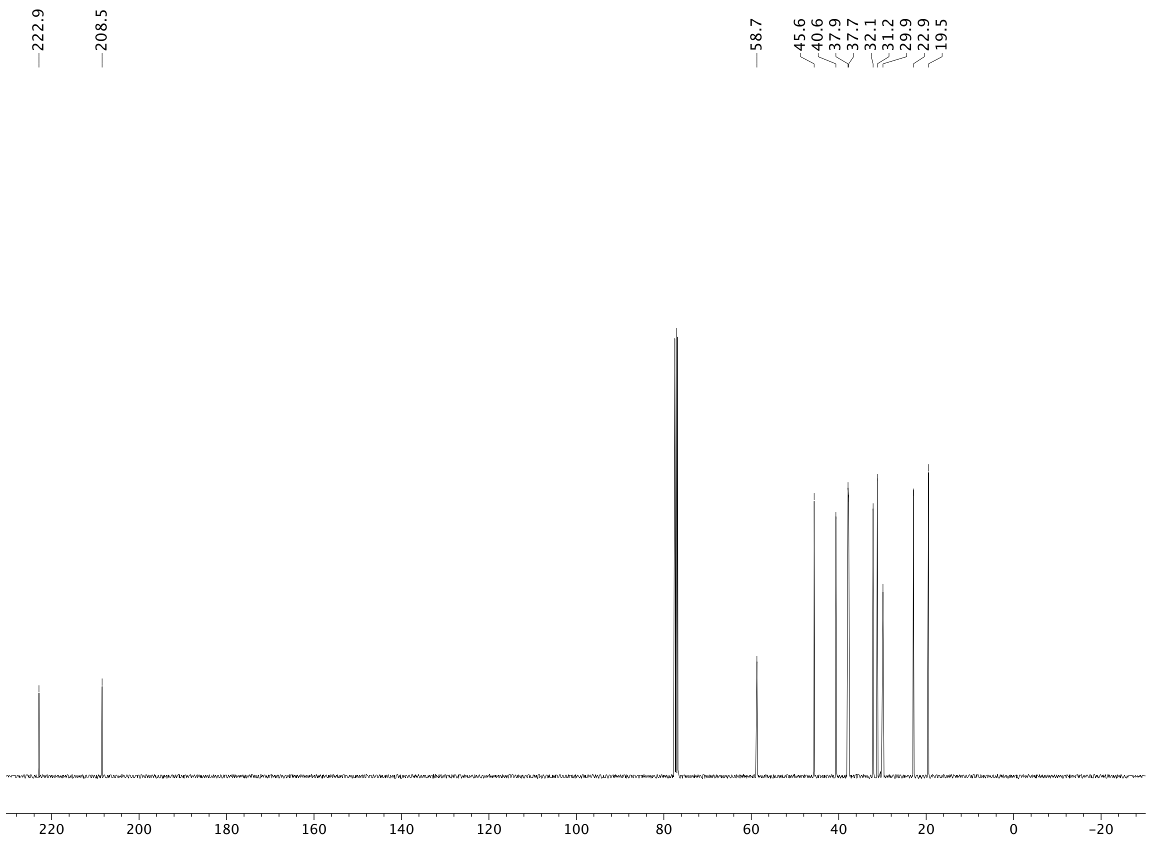

^{13}C NMR (100 MHz, $CDCl_3$) of compound **8d**.

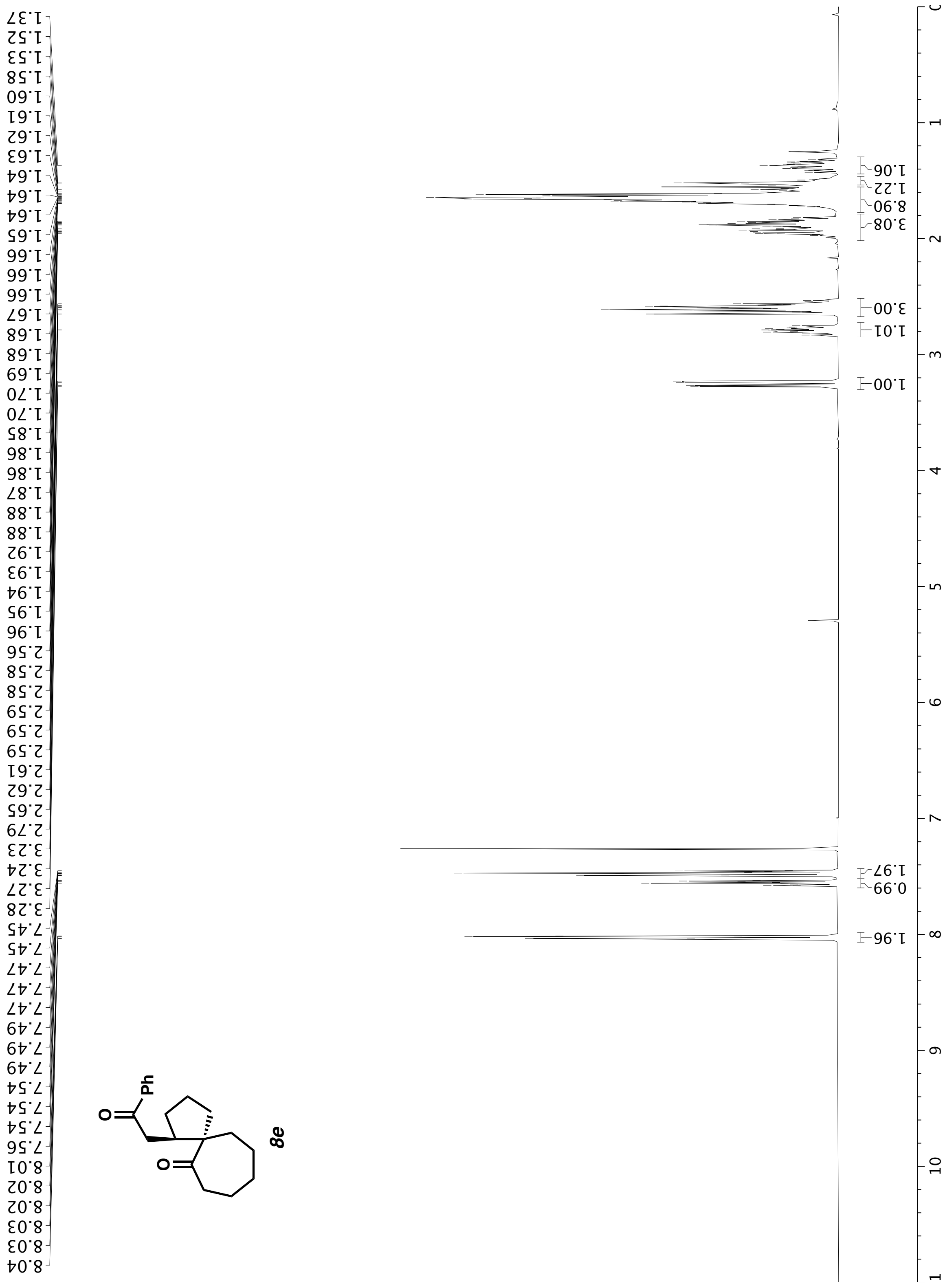

^{1}H NMR (400 MHz, CDCl$_3$) of compound **8e**.

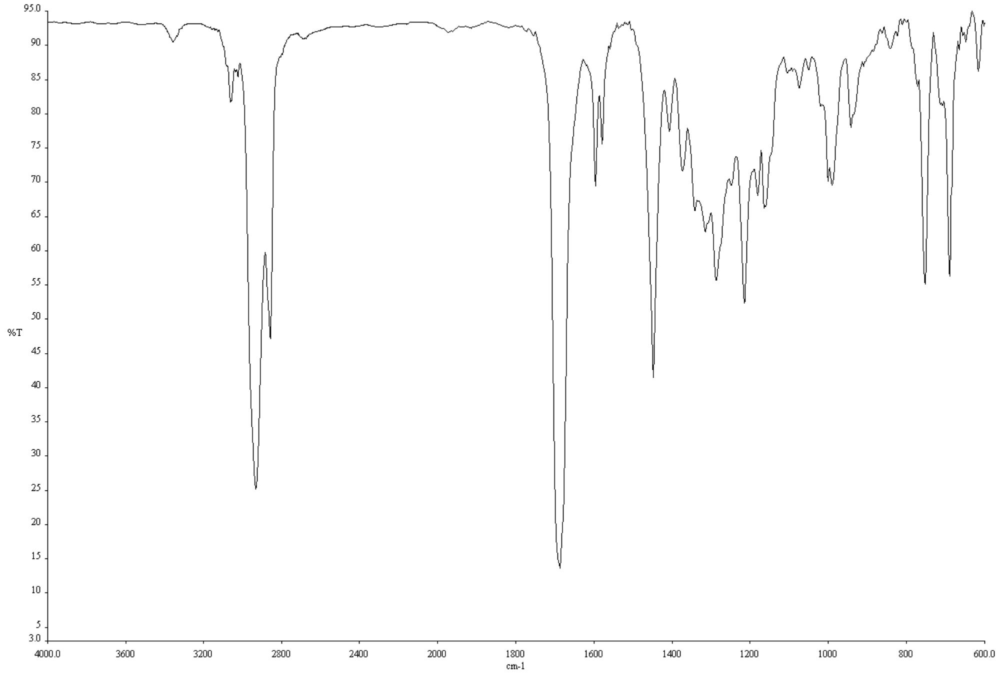

Infrared spectrum (Thin Film, NaCl) of compound **8e**.

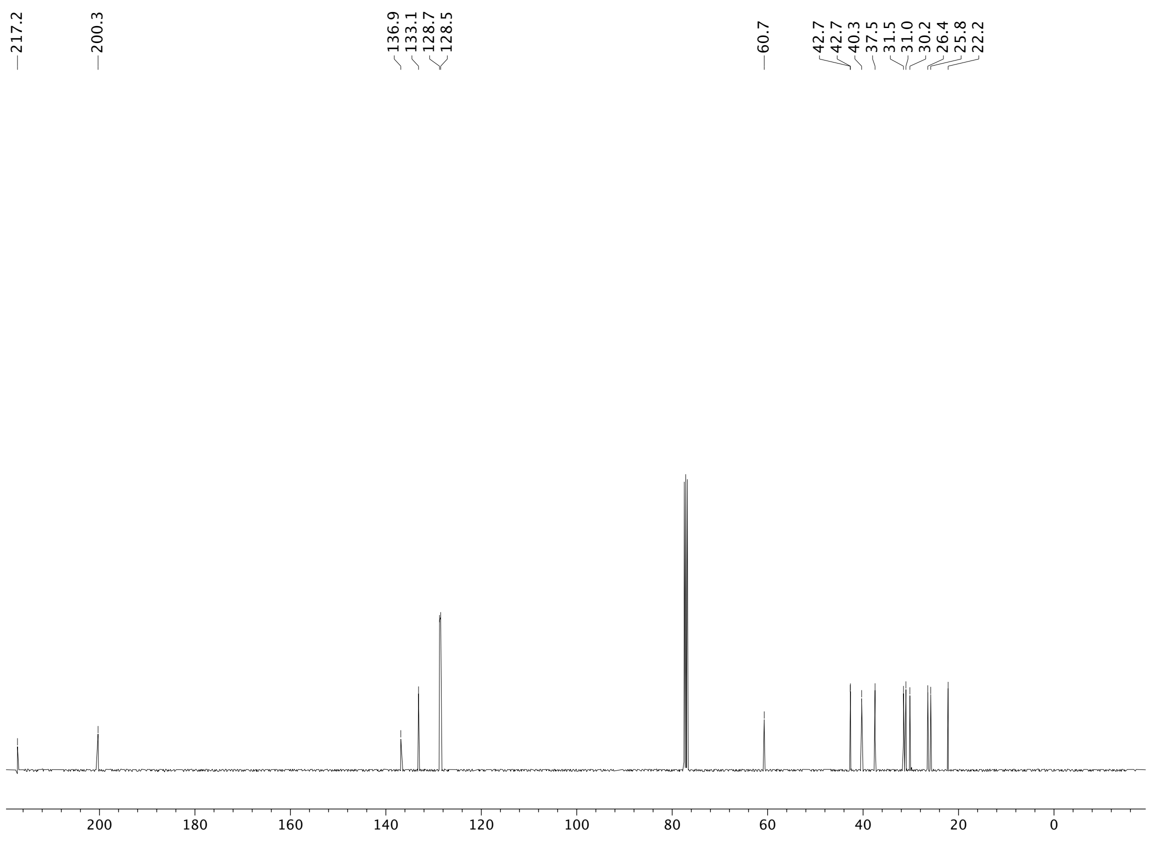

^{13}C NMR (100 MHz, $CDCl_3$) of compound **8e**.

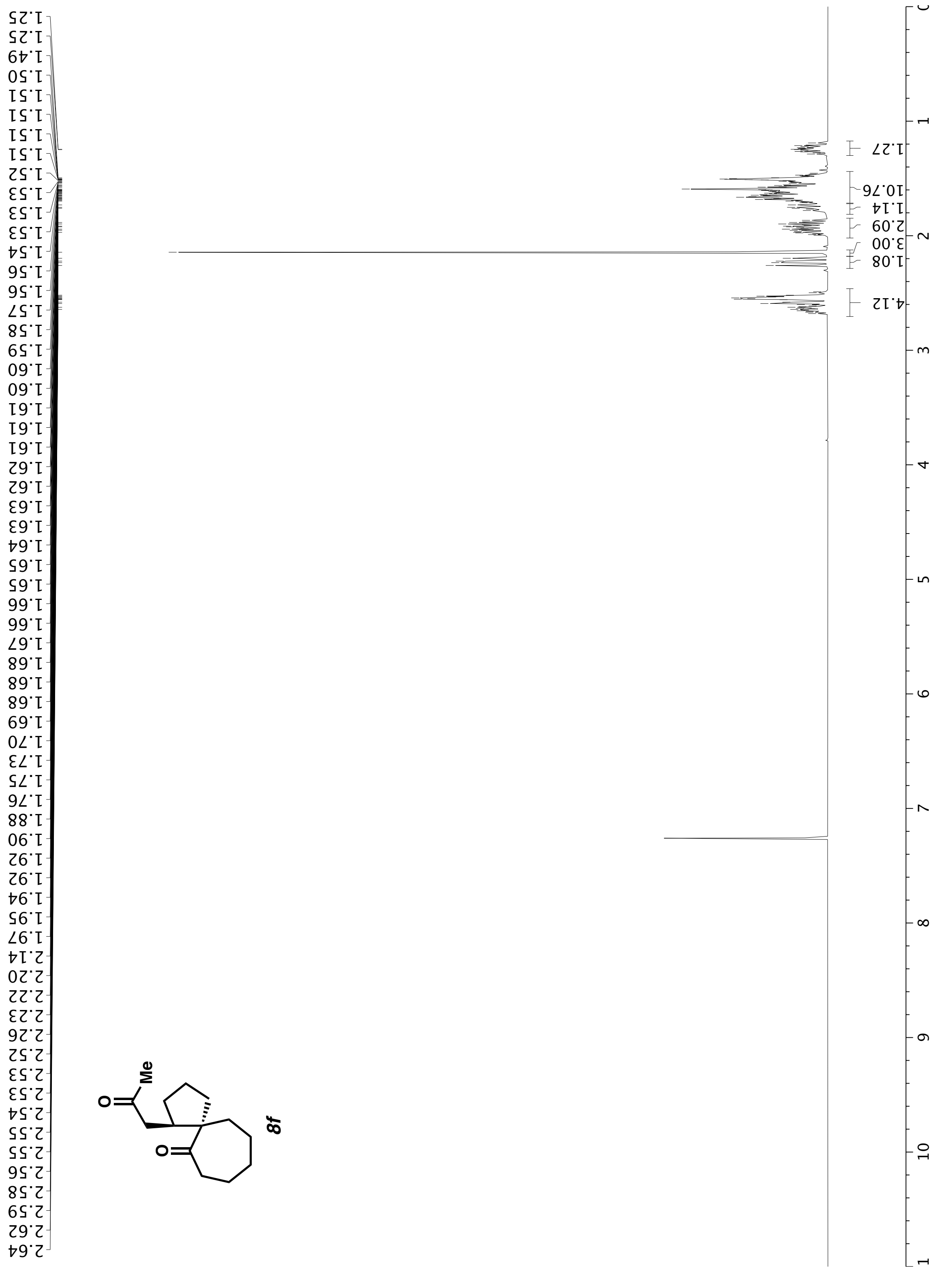

^{1}H NMR (400 MHz, $CDCl_3$) of compound **8f**.

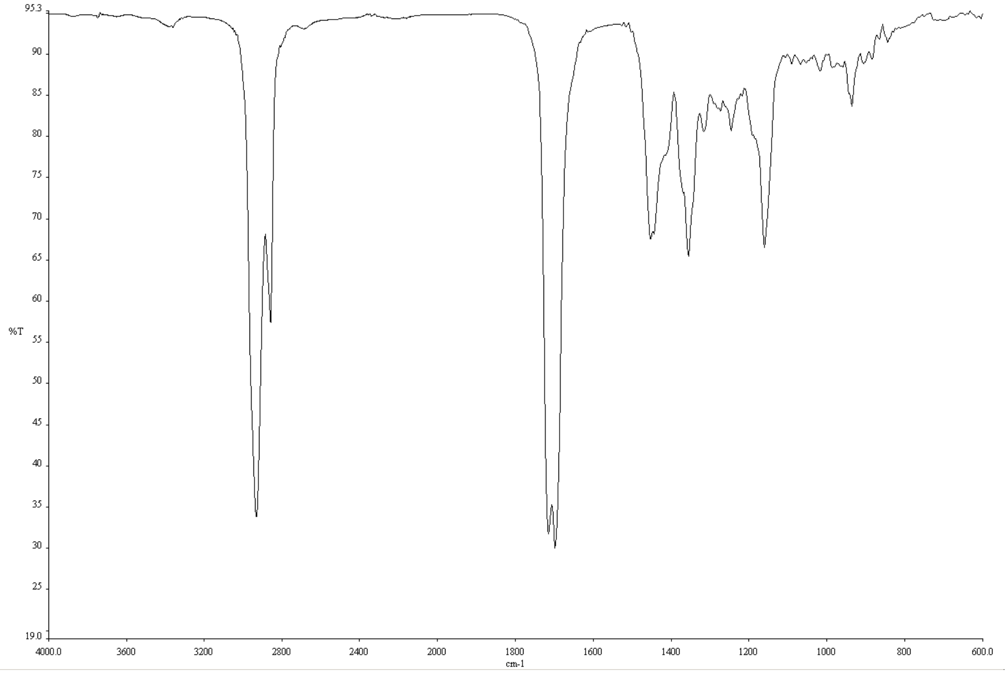

Infrared spectrum (Thin Film, NaCl) of compound **8f**.

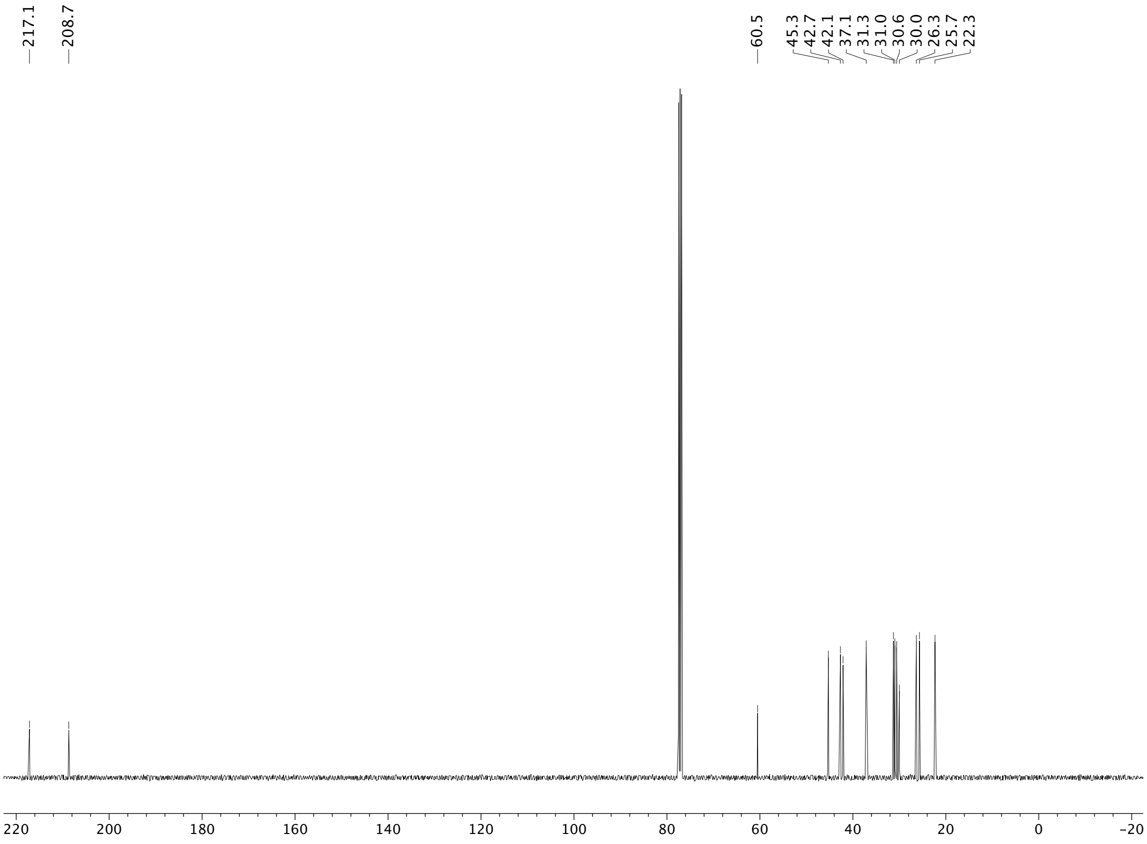

^{13}C NMR (100 MHz, $CDCl_3$) of compound **8f**.

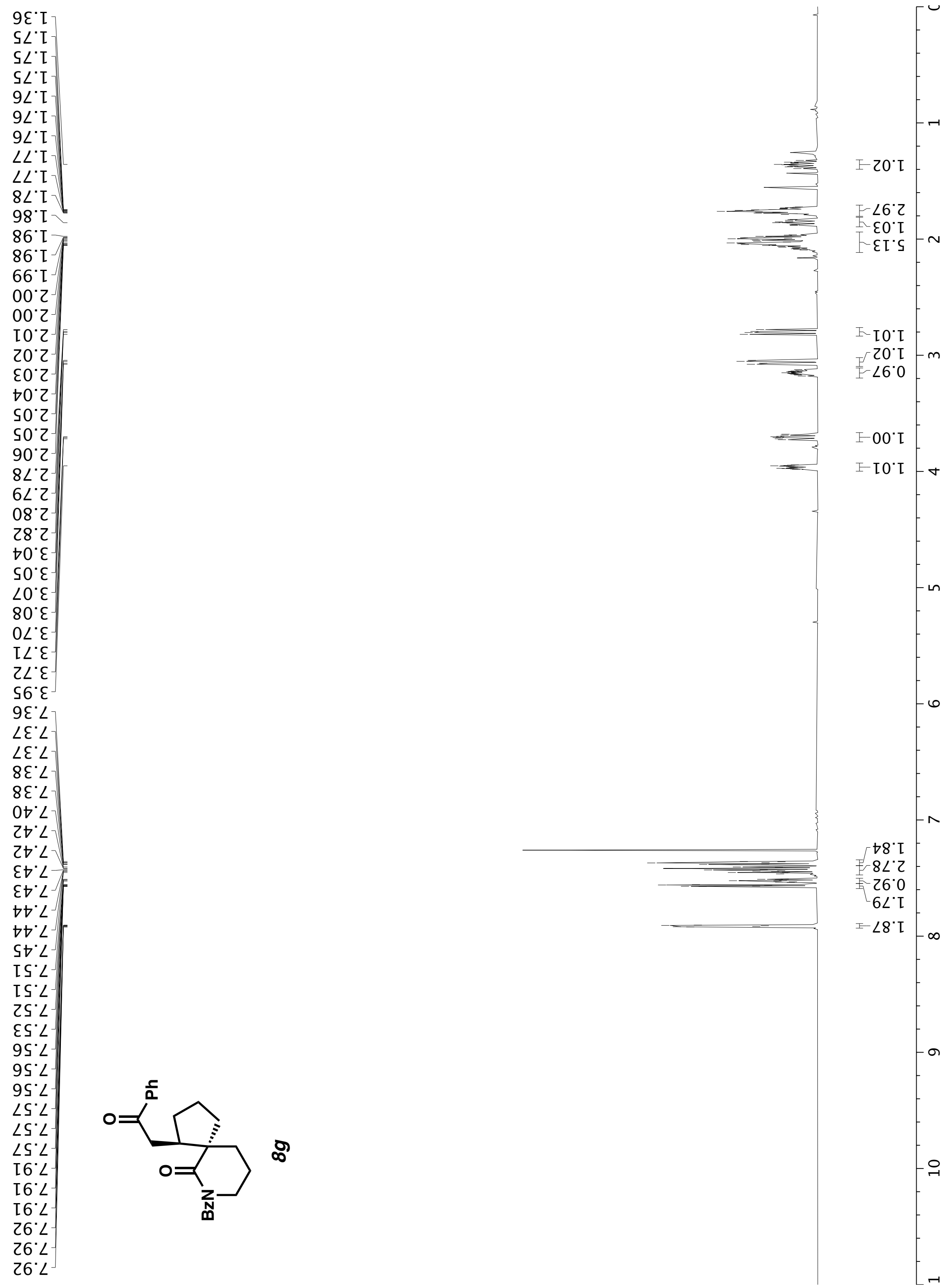

^{1}H NMR (600 MHz, $CDCl_3$) of compound **8g**.

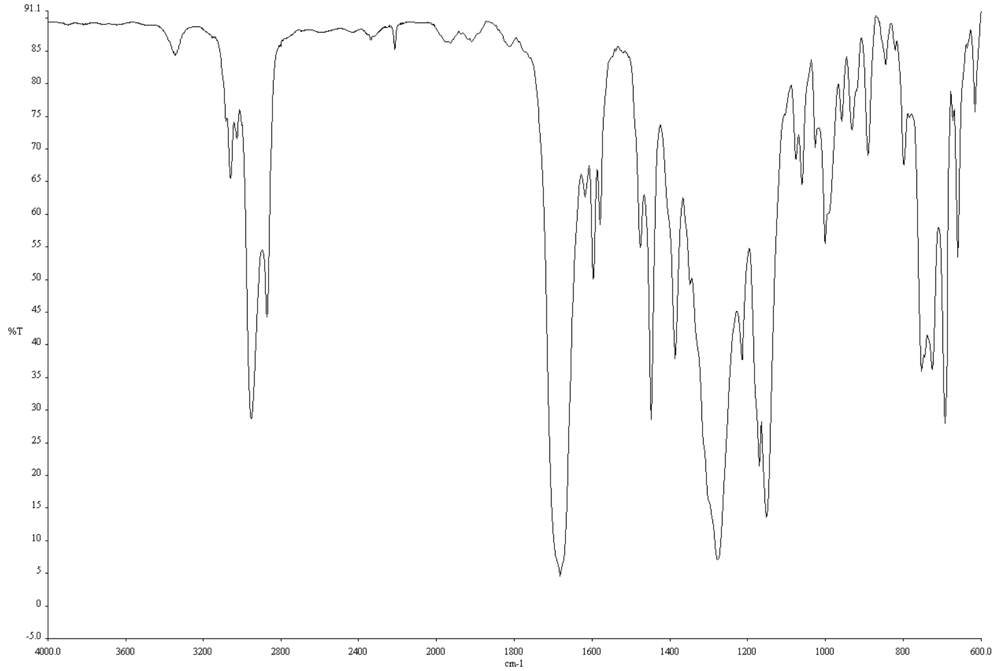

Infrared spectrum (Thin Film, NaCl) of compound **8g**.

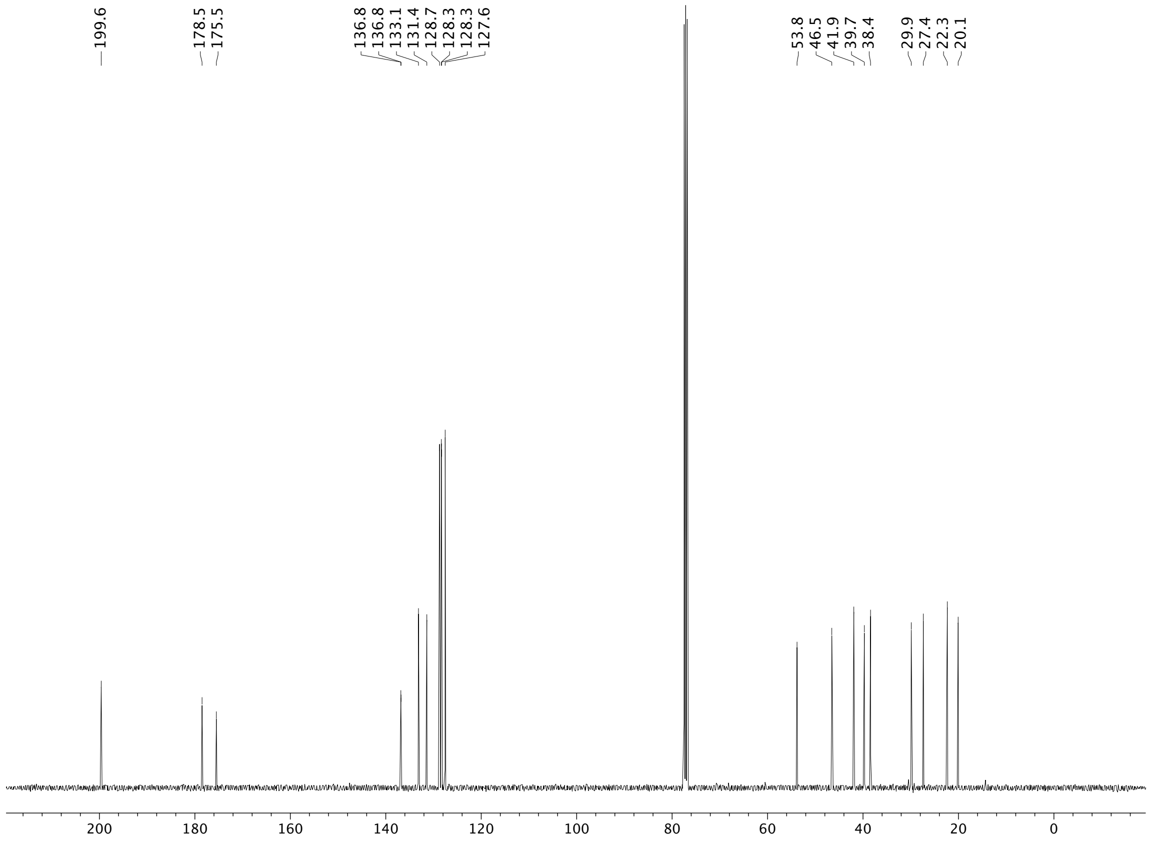

^{13}C NMR (100 MHz, $CDCl_3$) of compound **8g**.

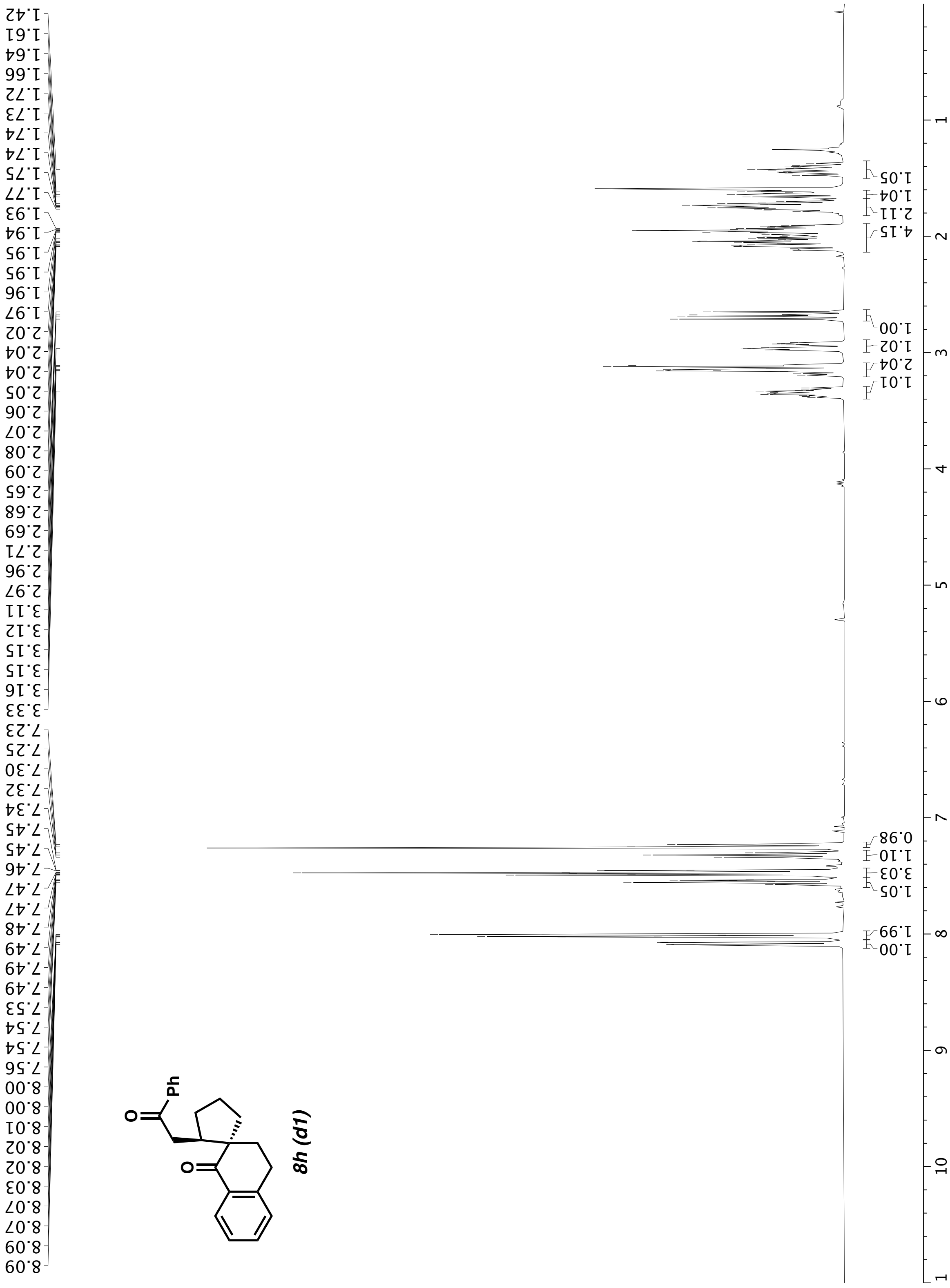

^{1}H NMR (400 MHz, $CDCl_3$) of compound **8h (*d1*)**.

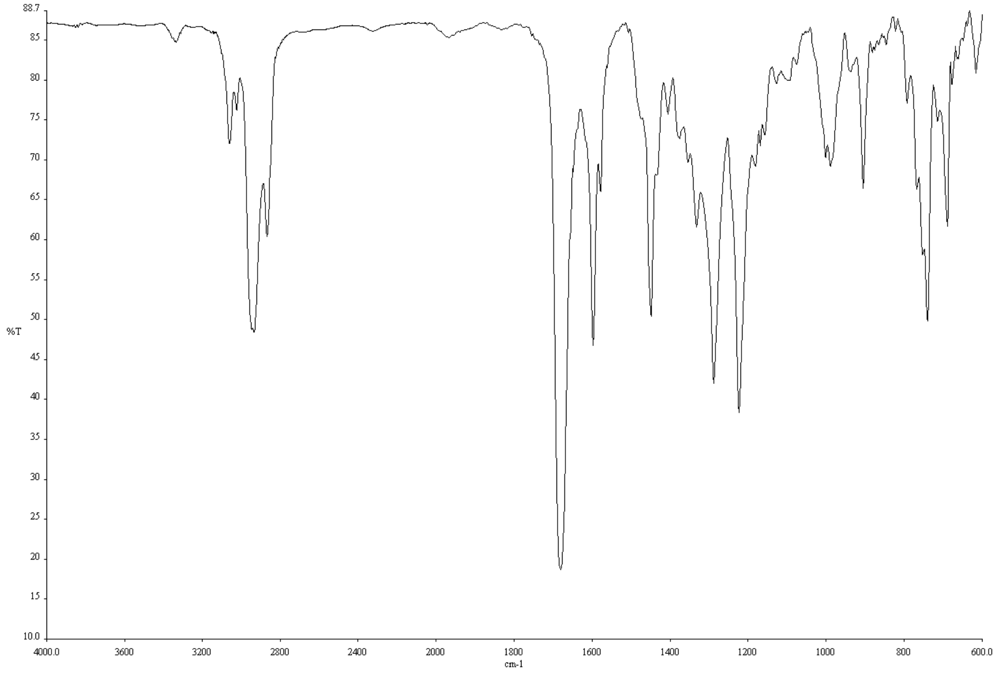

Infrared spectrum (Thin Film, NaCl) of compound **8h (*d1*)**.

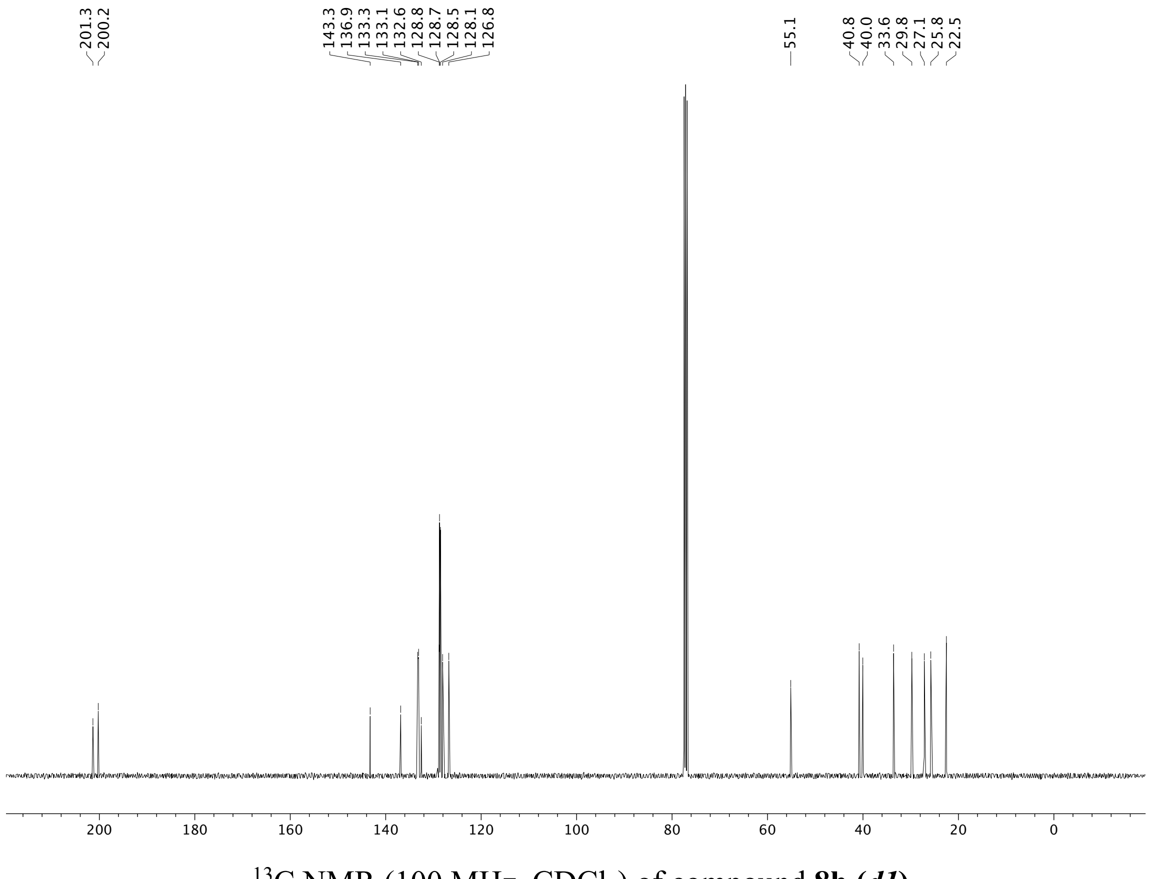

^{13}C NMR (100 MHz, $CDCl_3$) of compound **8h (*d1*)**.

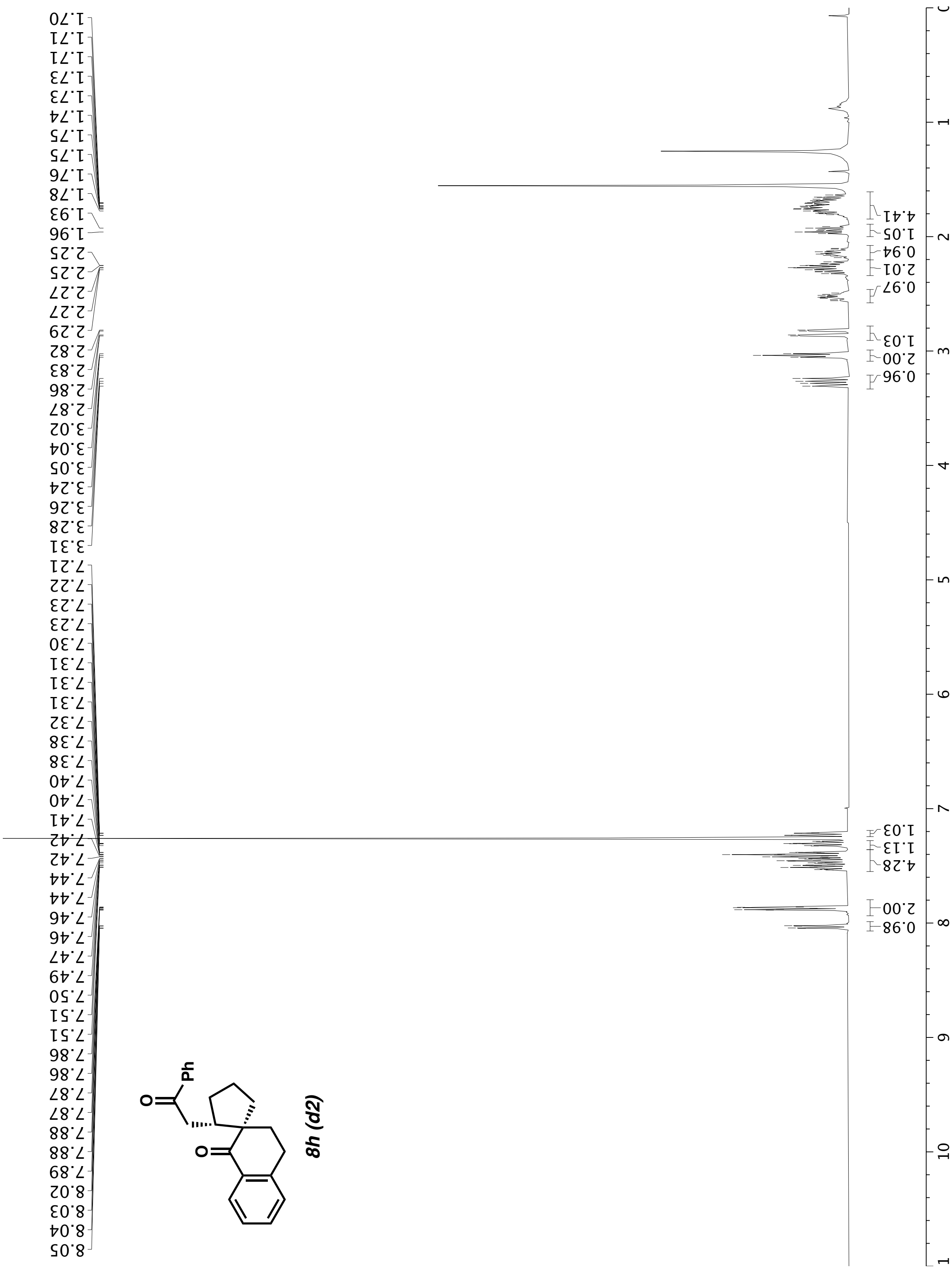

^{1}H NMR (400 MHz, $CDCl_3$) of compound **8h (*d2*)**.

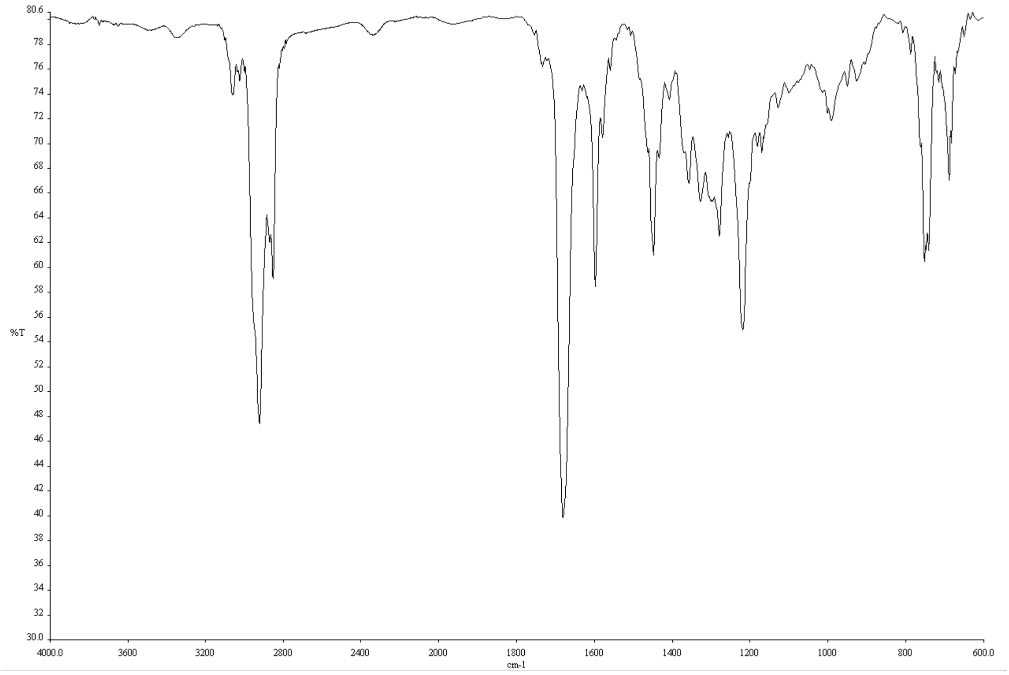

Infrared spectrum (Thin Film, NaCl) of compound **8h** (***d2***).

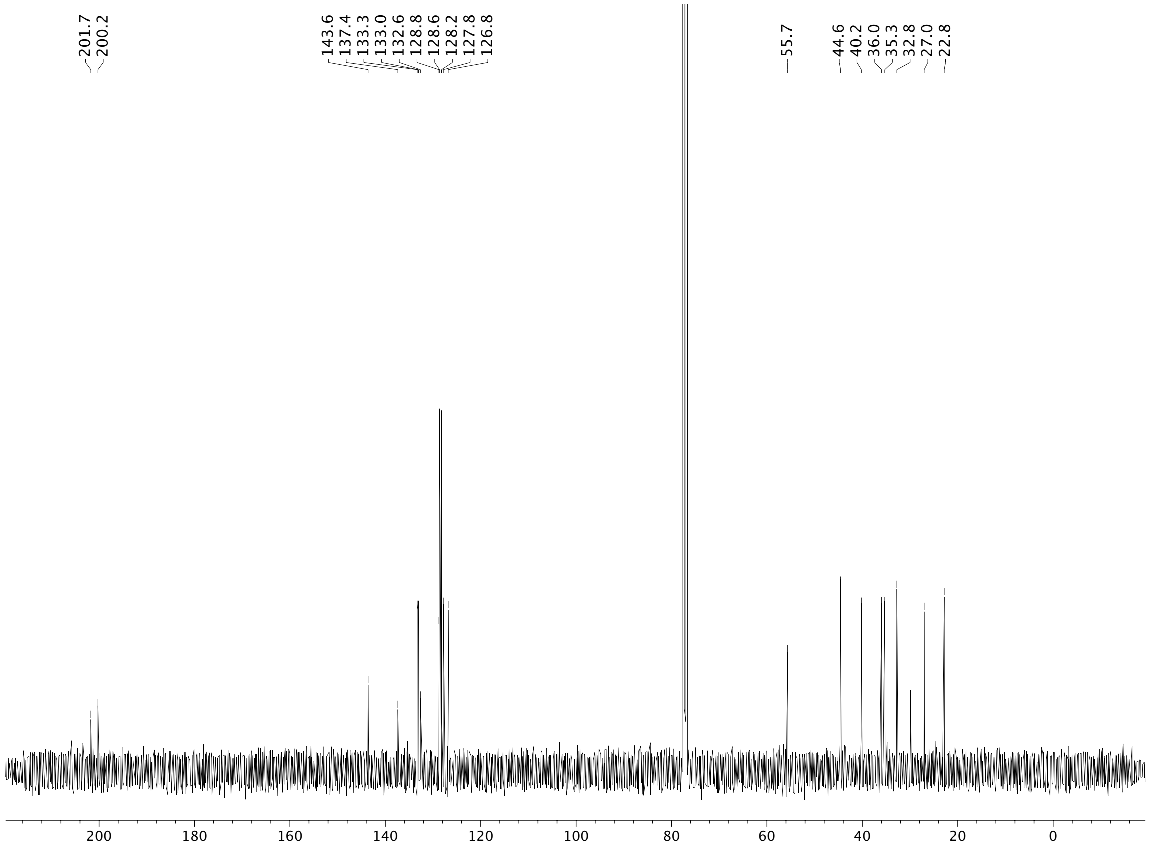

^{13}C NMR (100 MHz, $CDCl_3$) of compound **8h** (***d2***).

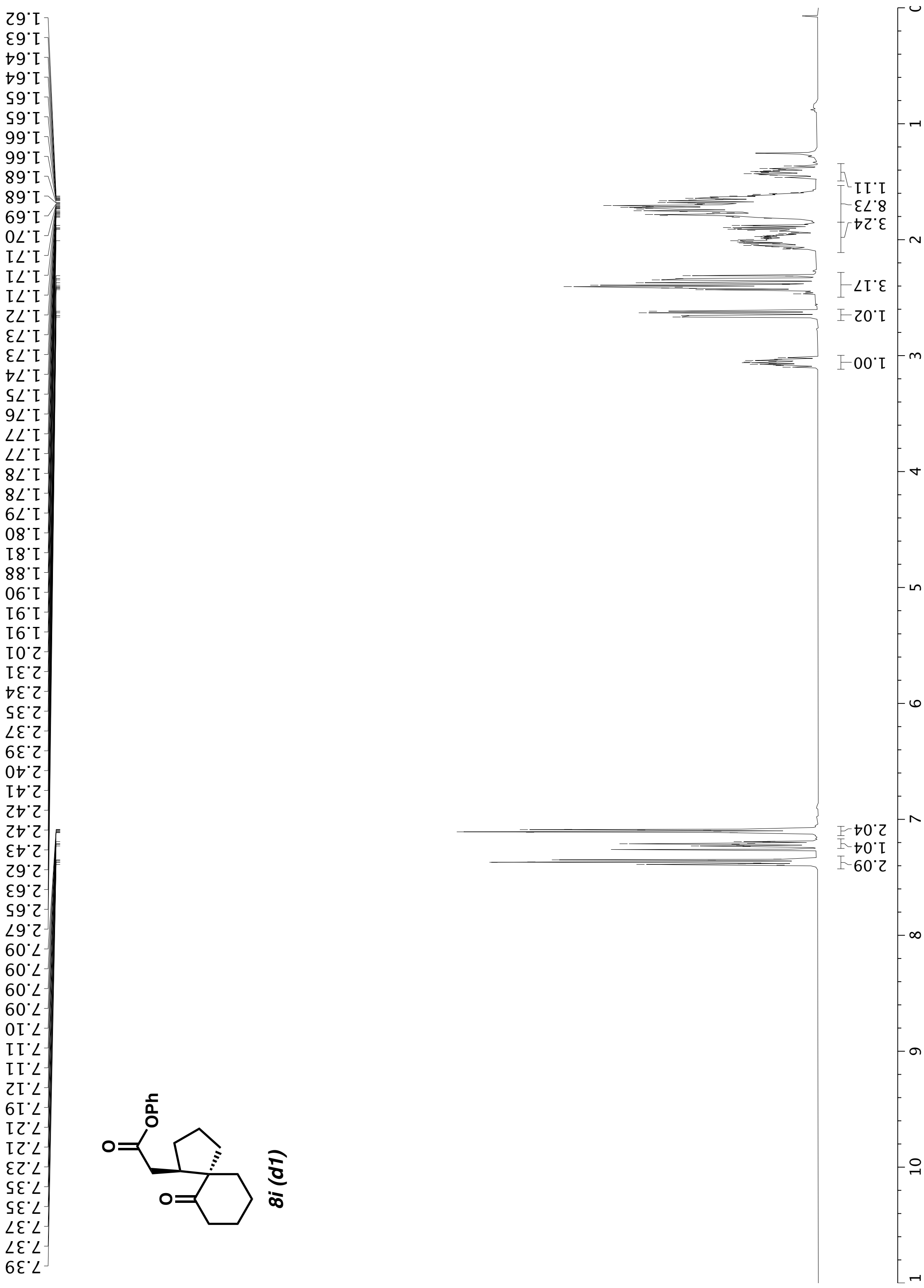

^{1}H NMR (400 MHz, $CDCl_3$) of compound **8i (*d1*)**.

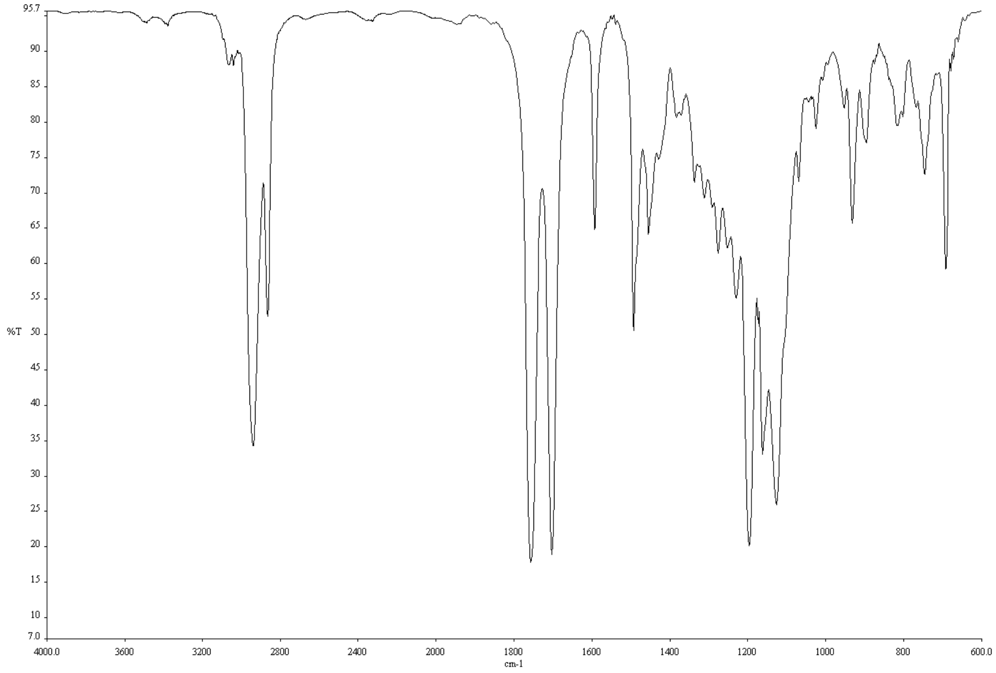

Infrared spectrum (Thin Film, NaCl) of compound **8i (*d1*)**.

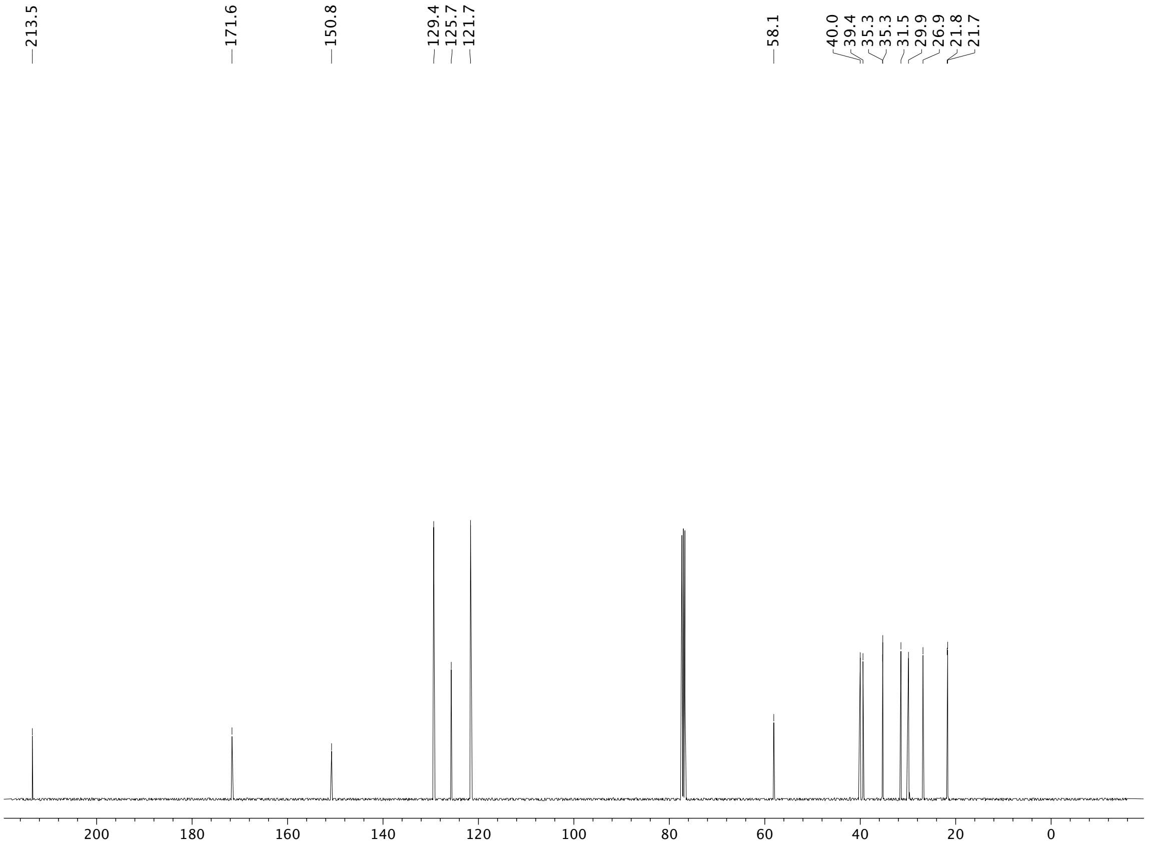

^{13}C NMR (100 MHz, $CDCl_3$) of compound **8i (*d1*)**.

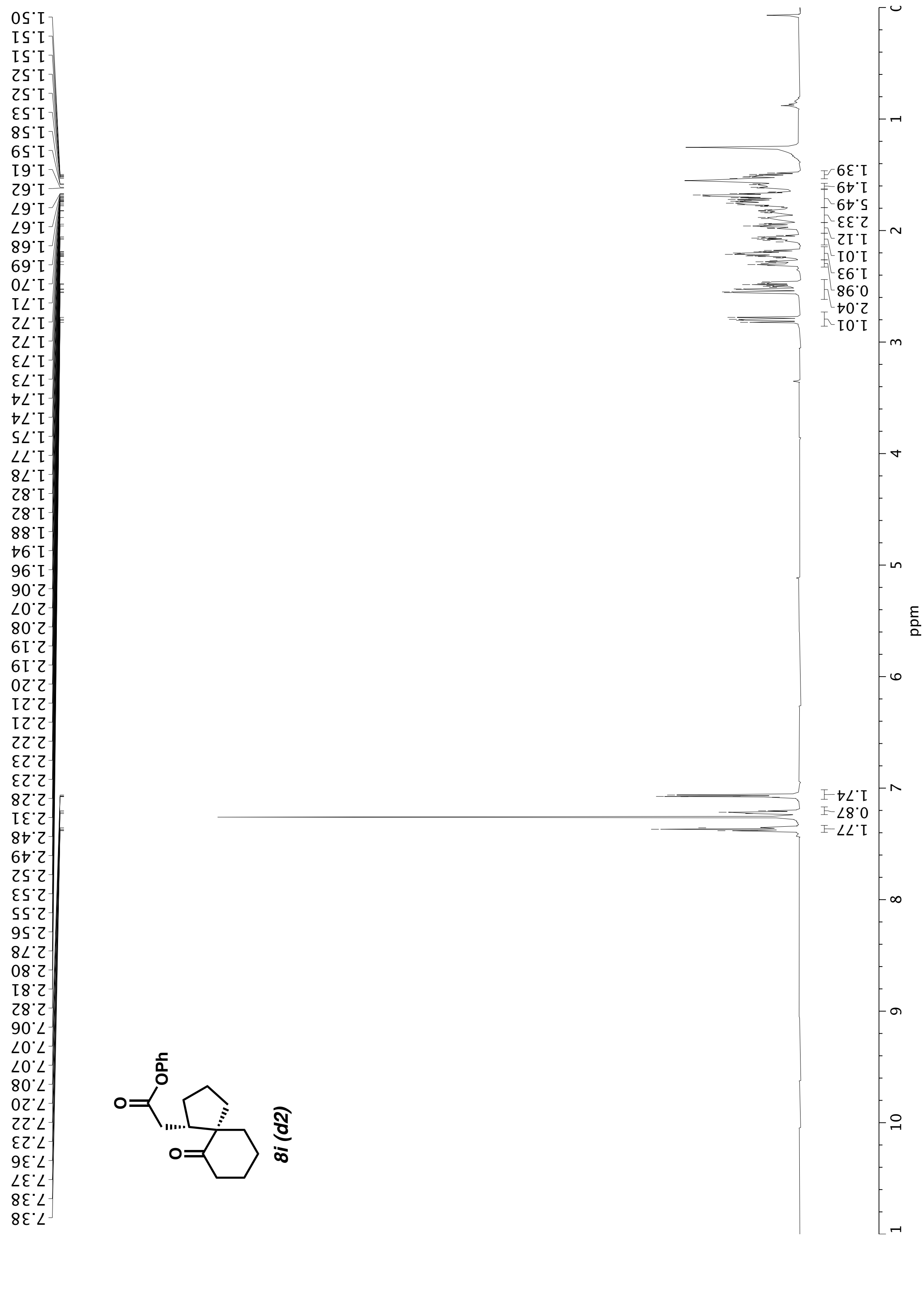

^{1}H NMR (600 MHz, $CDCl_3$) of compound **8i (*d2*)**.

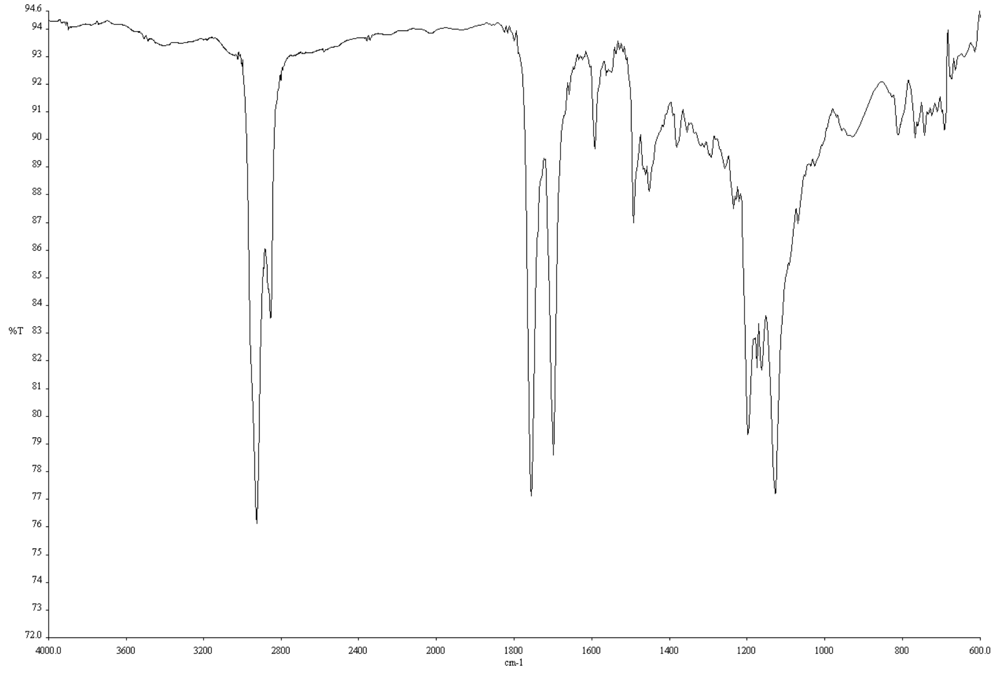

Infrared spectrum (Thin Film, NaCl) of compound **8i** (***d2***).

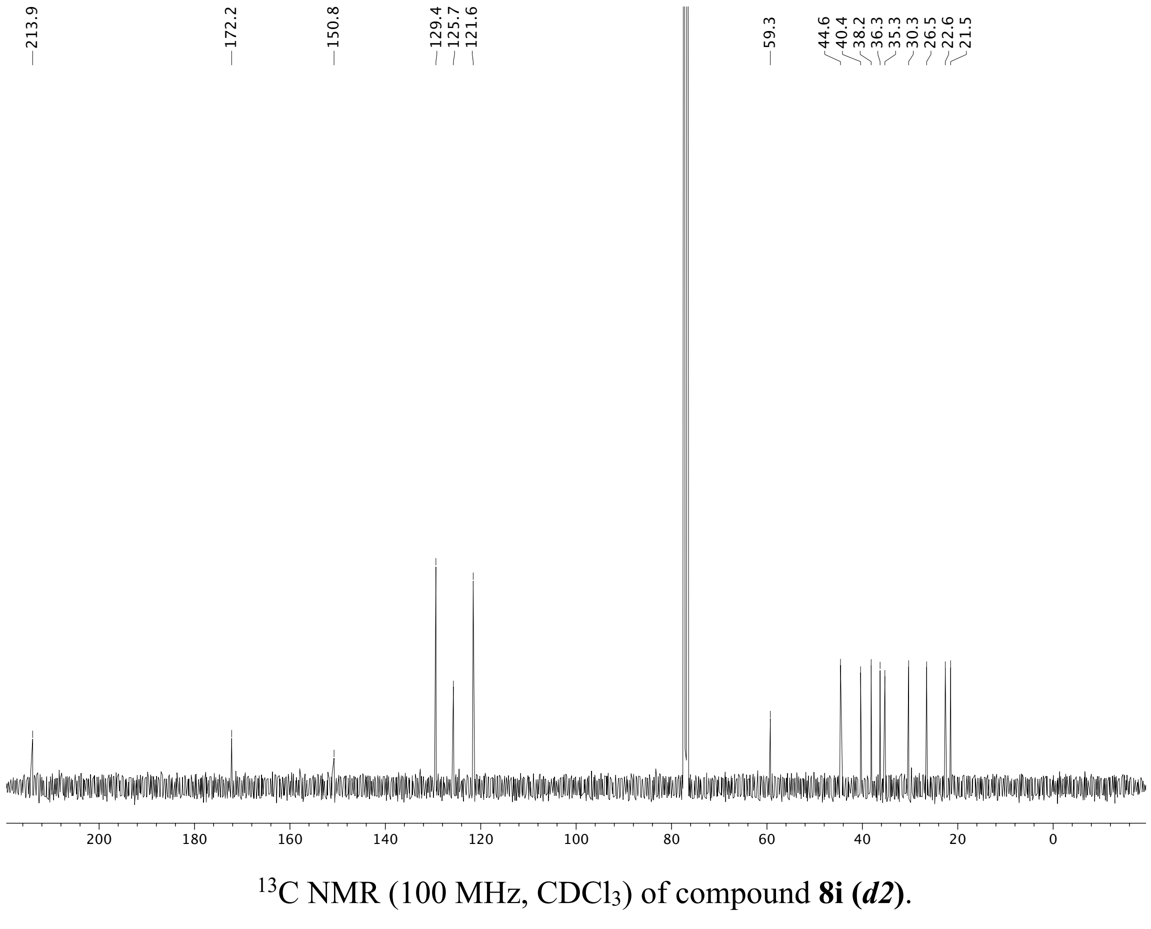

^{13}C NMR (100 MHz, $CDCl_3$) of compound **8i** (***d2***).

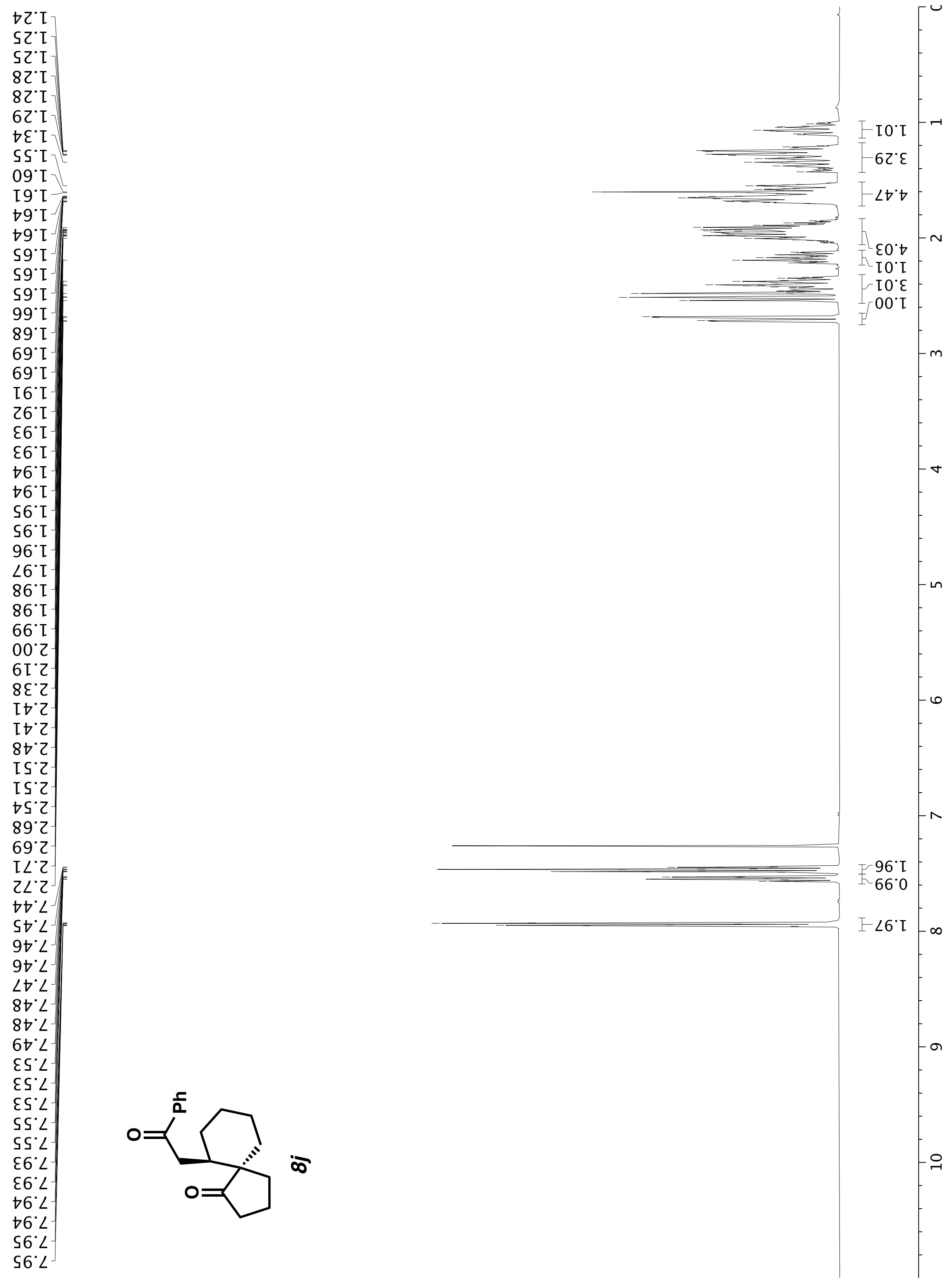

^{1}H NMR (400 MHz, $CDCl_3$) of compound **8j**.

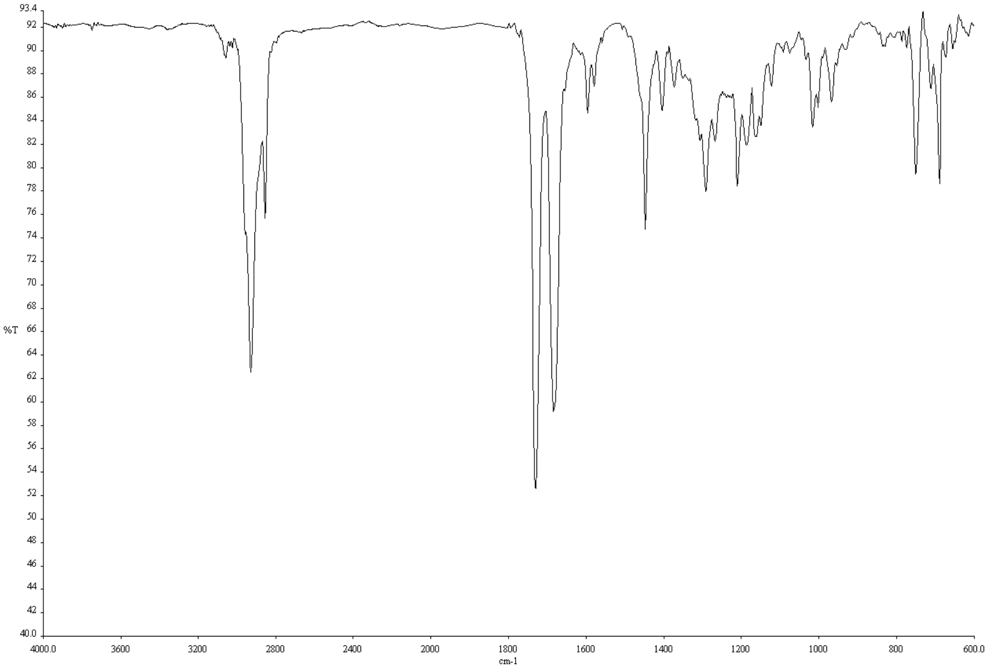

Infrared spectrum (Thin Film, NaCl) of compound **8j**.

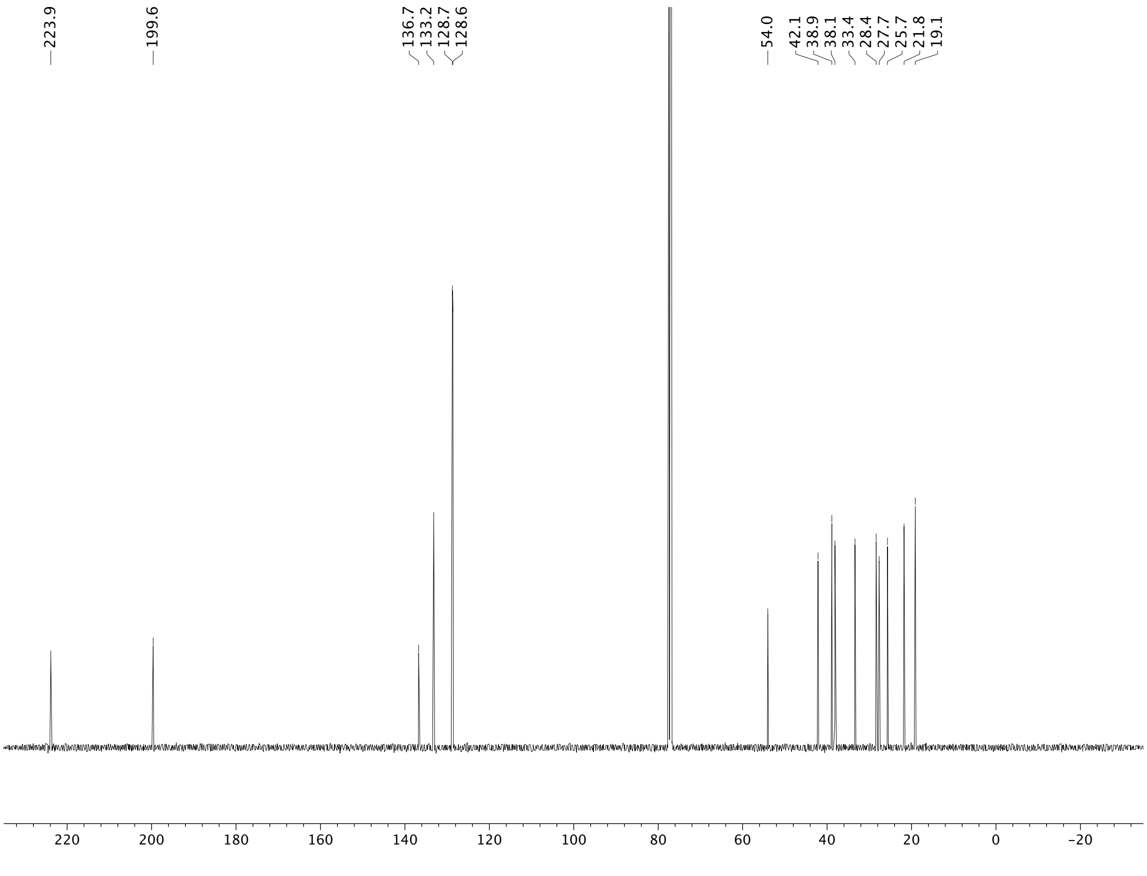

^{13}C NMR (100 MHz, $CDCl_3$) of compound **8j**.

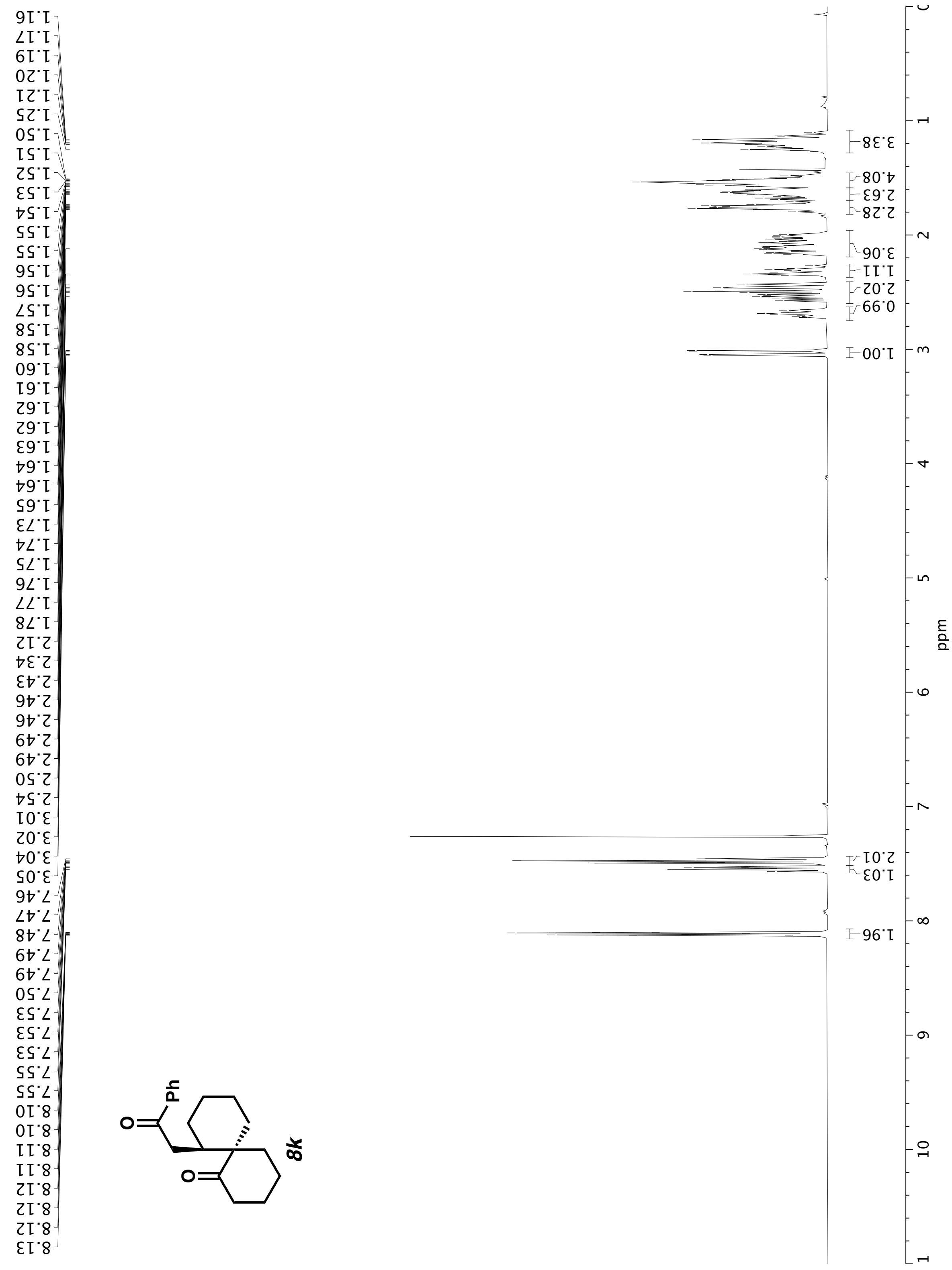

^{1}H NMR (400 MHz, $CDCl_3$) of compound **8k**.

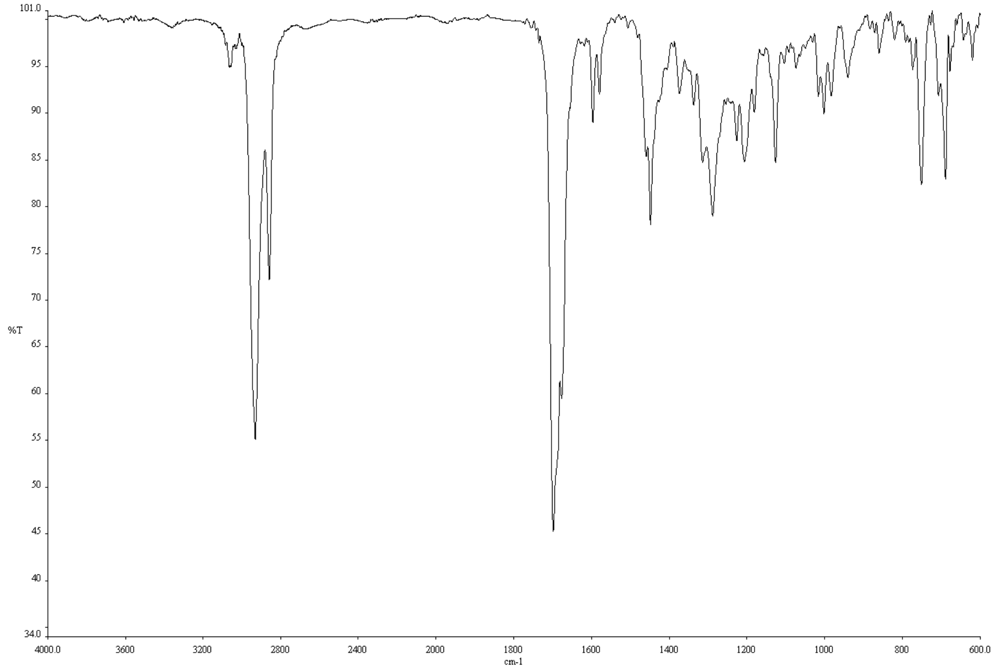

Infrared spectrum (Thin Film, NaCl) of compound **8k**.

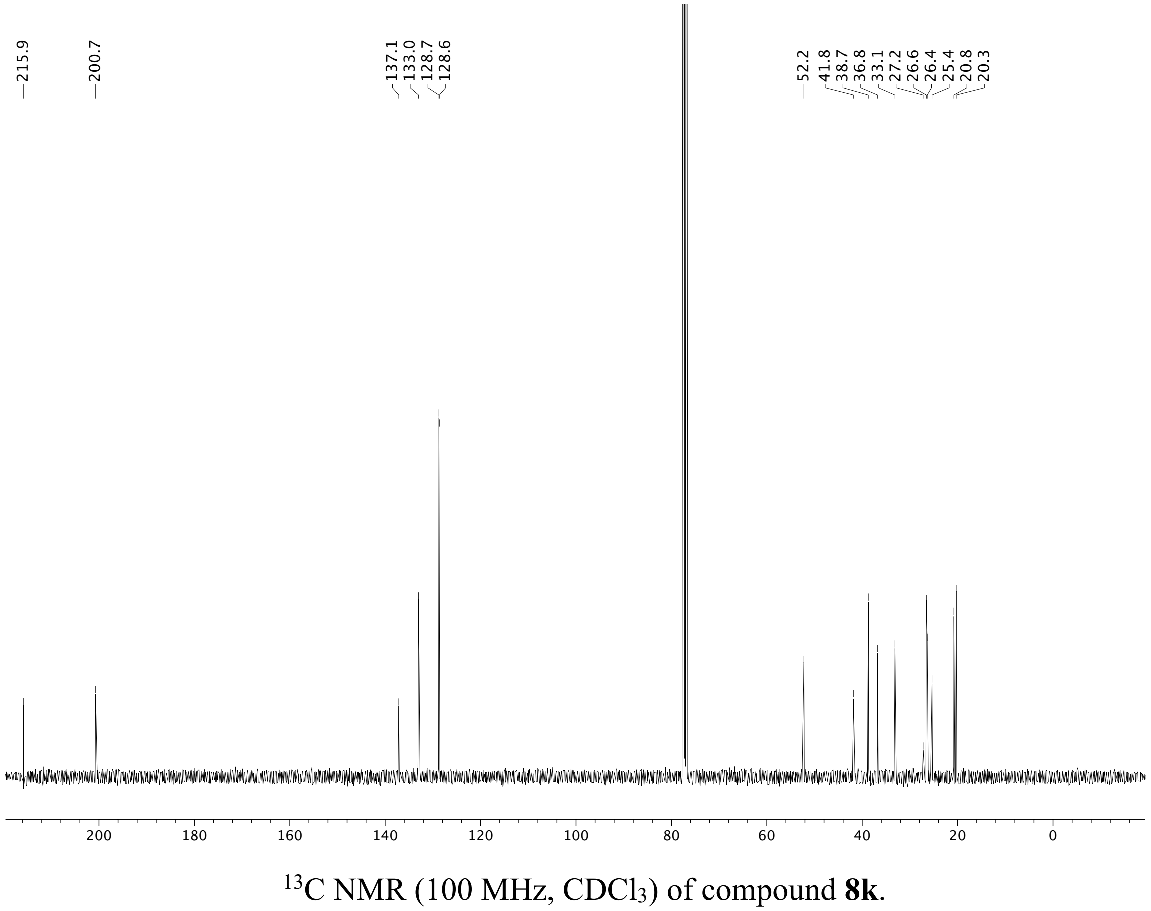

^{13}C NMR (100 MHz, $CDCl_3$) of compound **8k**.

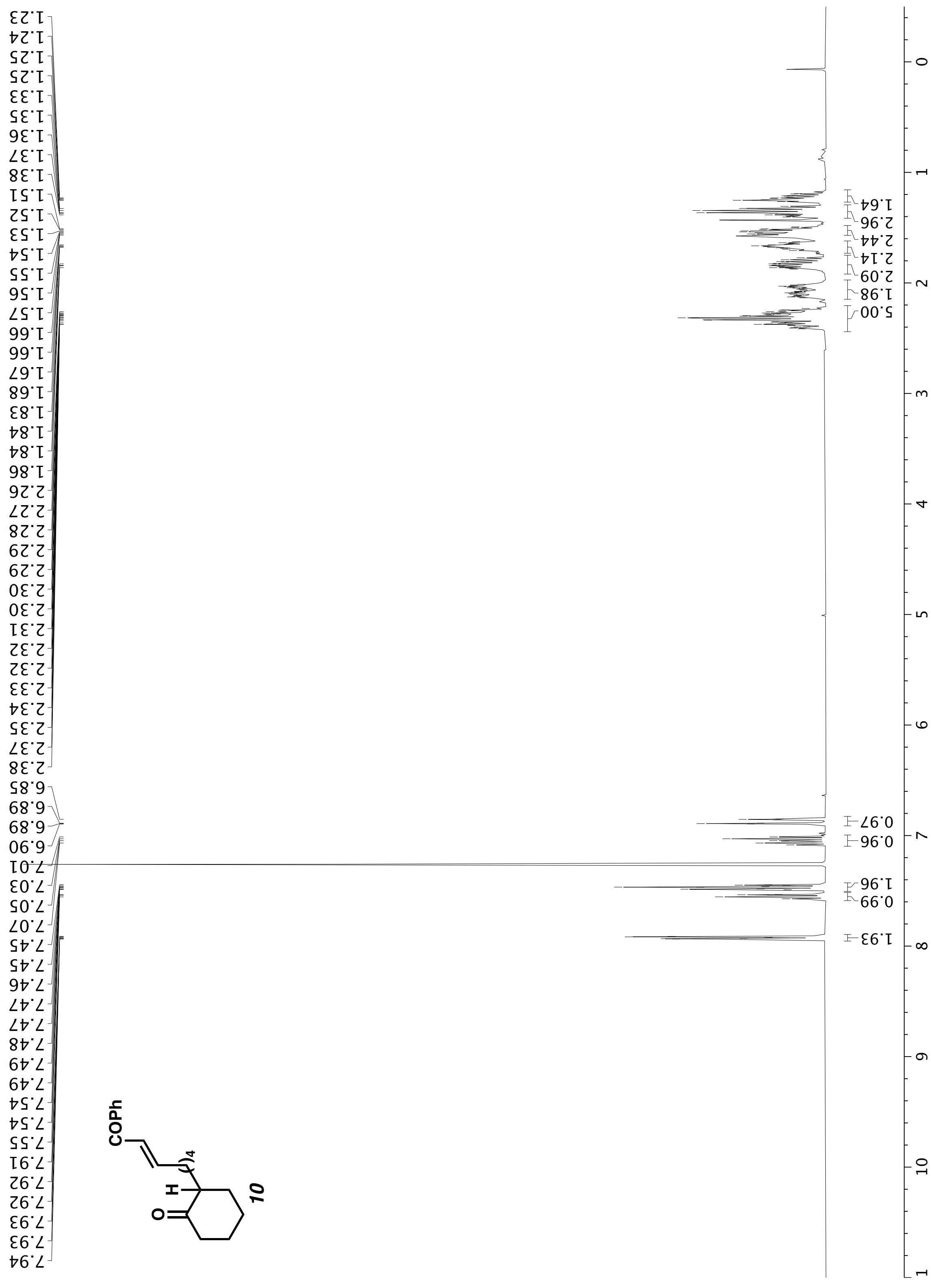

^{1}H NMR (400 MHz, $CDCl_3$) of compound **10**.

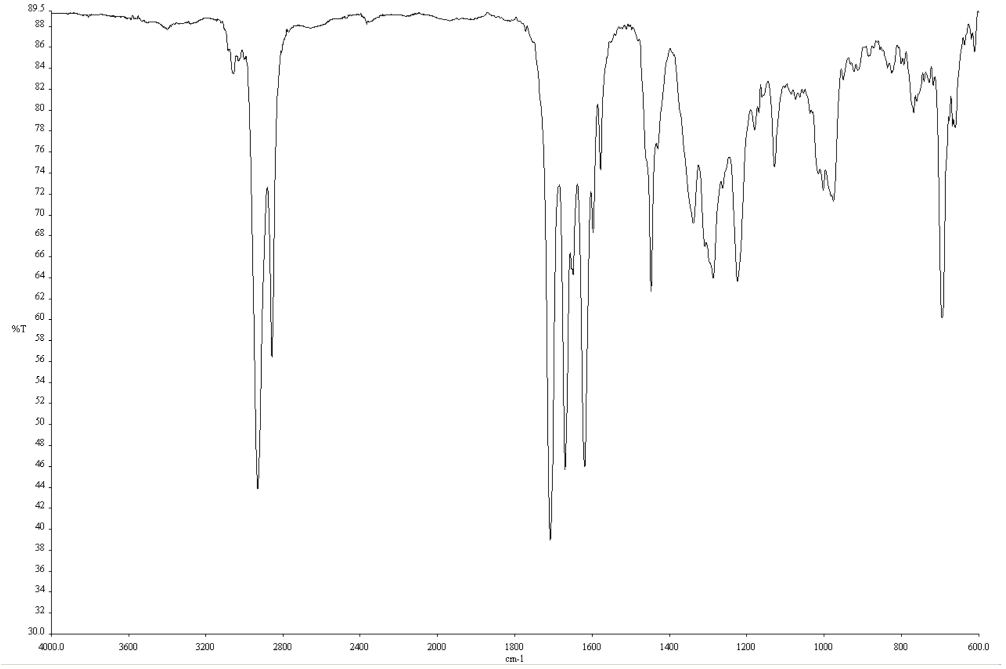

Infrared spectrum (Thin Film, NaCl) of compound **10**.

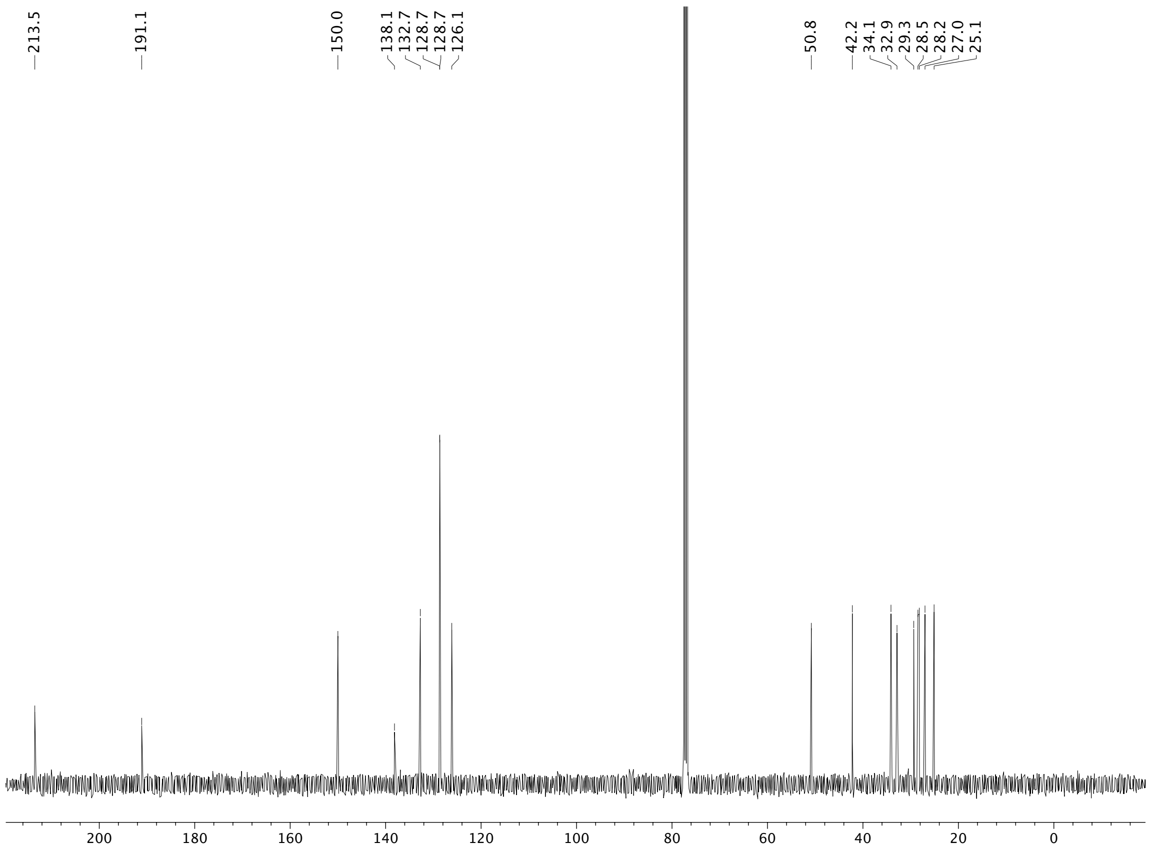

^{13}C NMR (100 MHz, $CDCl_3$) of compound **10**.

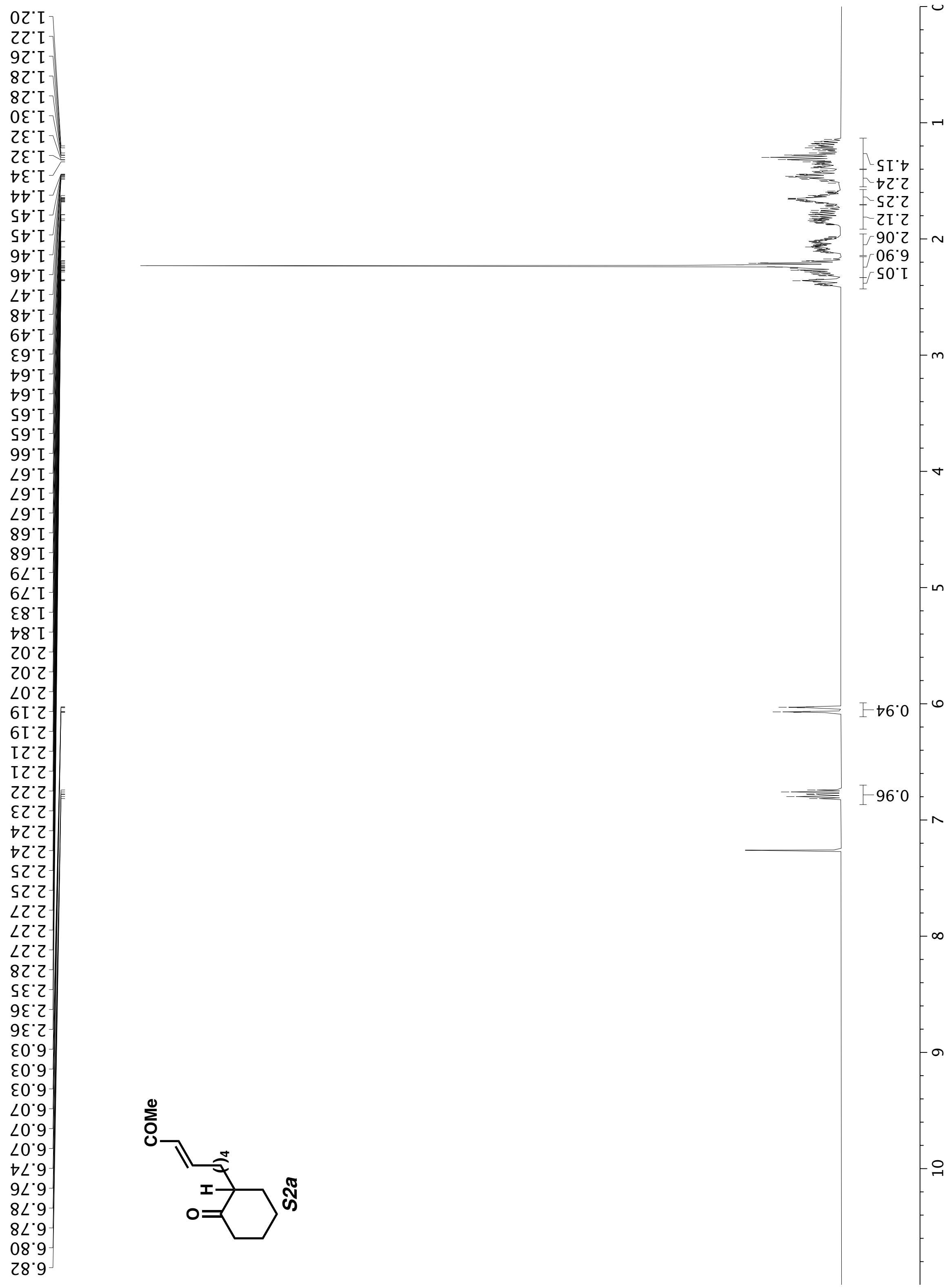

^{1}H NMR (400 MHz, $CDCl_3$) of compound **S2a**.

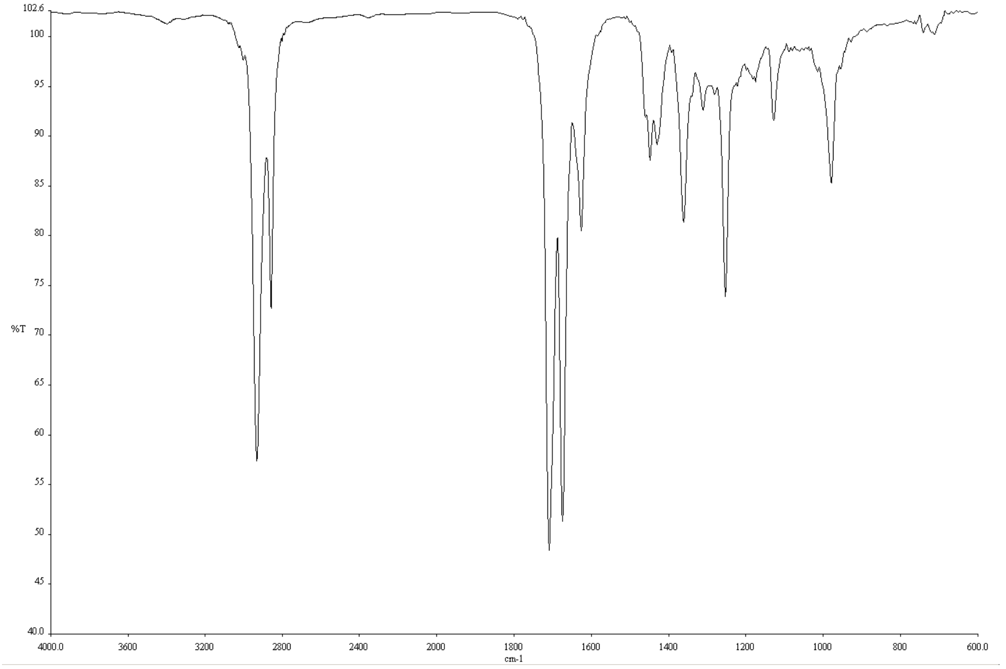

Infrared spectrum (Thin Film, NaCl) of compound **S2a**.

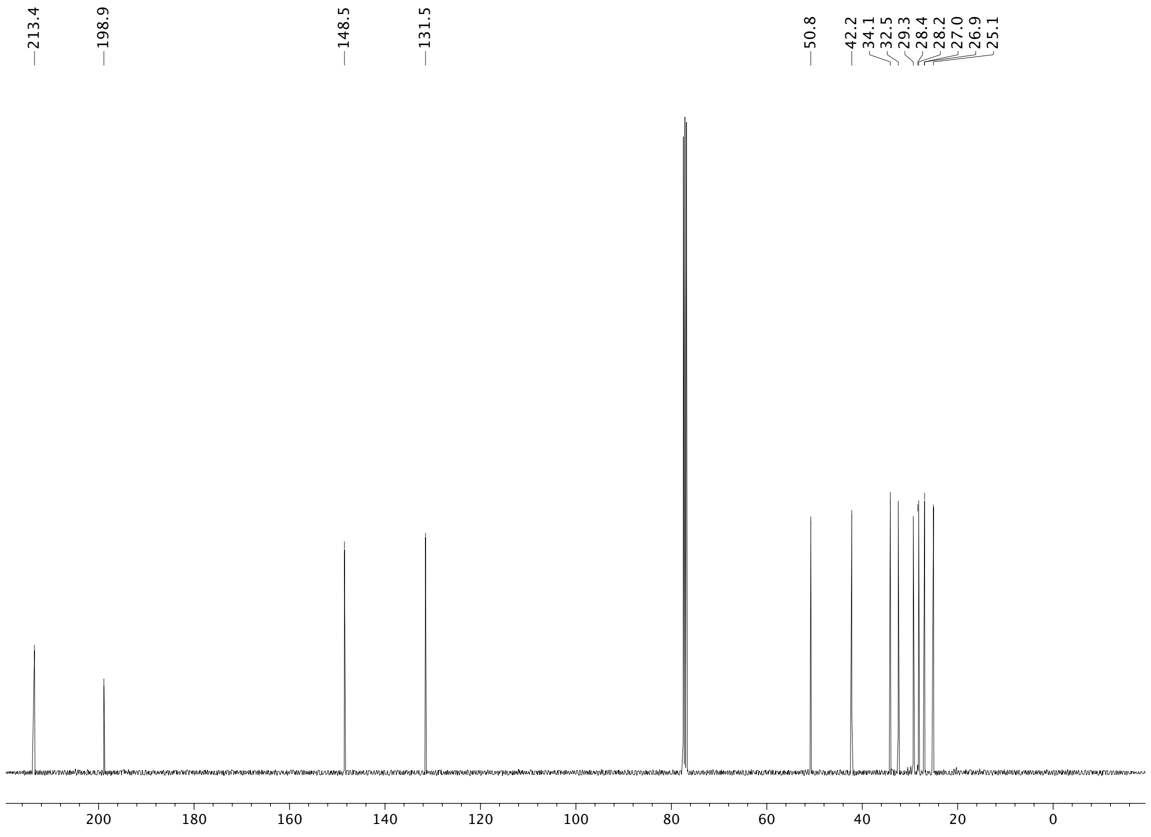

^{13}C NMR (100 MHz, $CDCl_3$) of compound **S2a**.

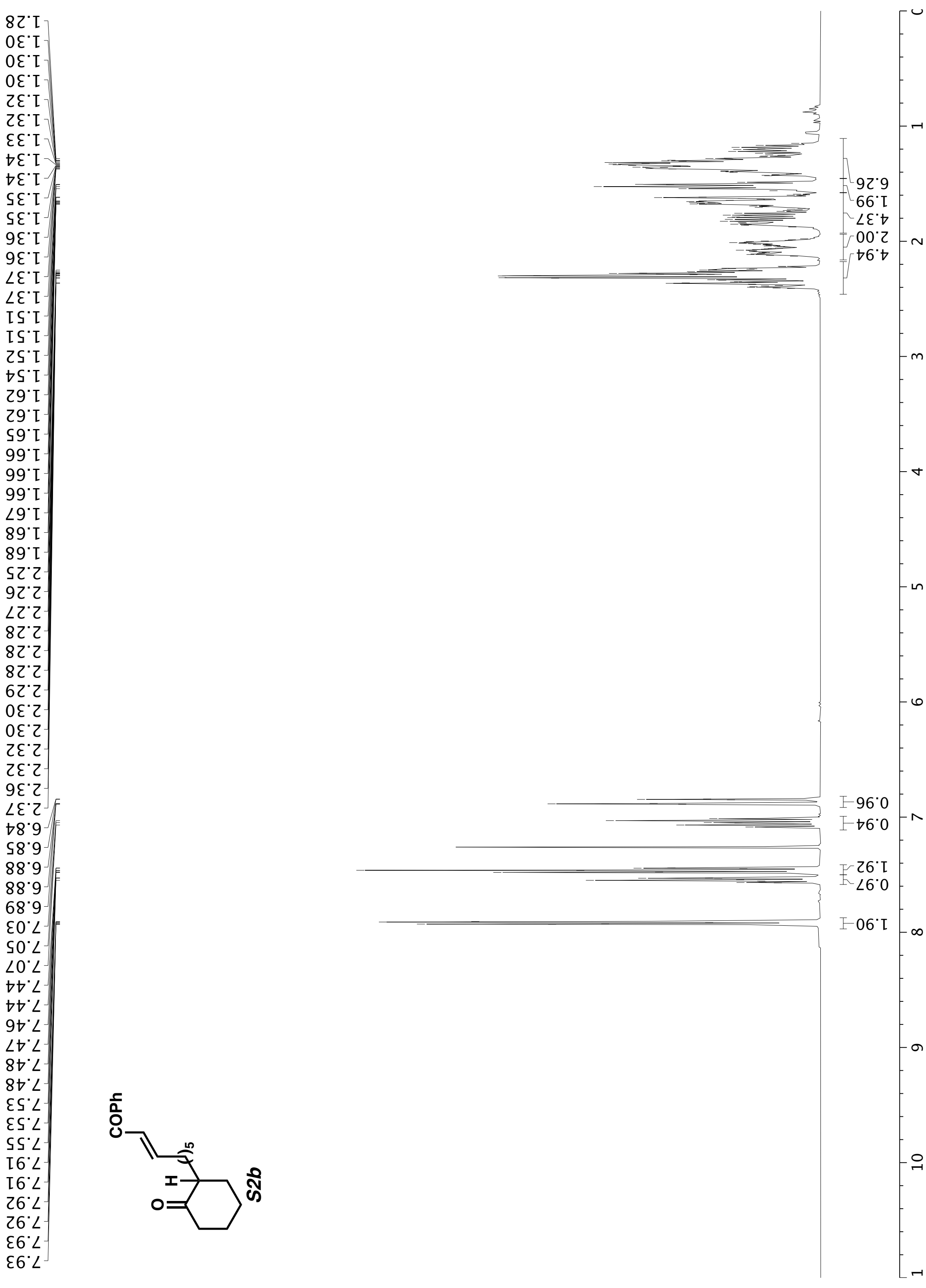

^{1}H NMR (400 MHz, $CDCl_3$) of compound **S2b**.

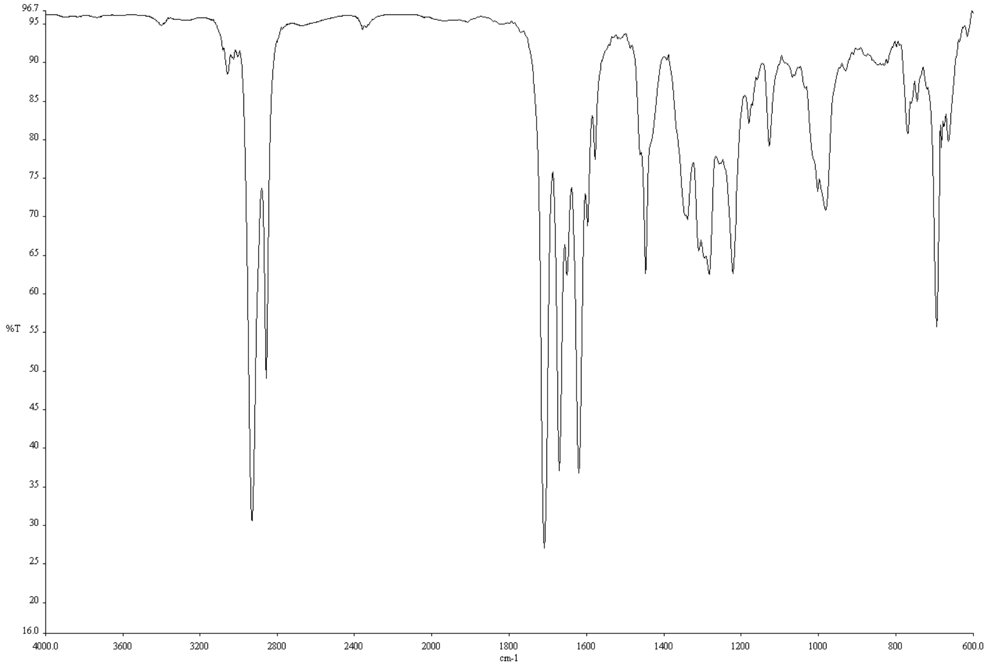

Infrared spectrum (Thin Film, NaCl) of compound **S2b**.

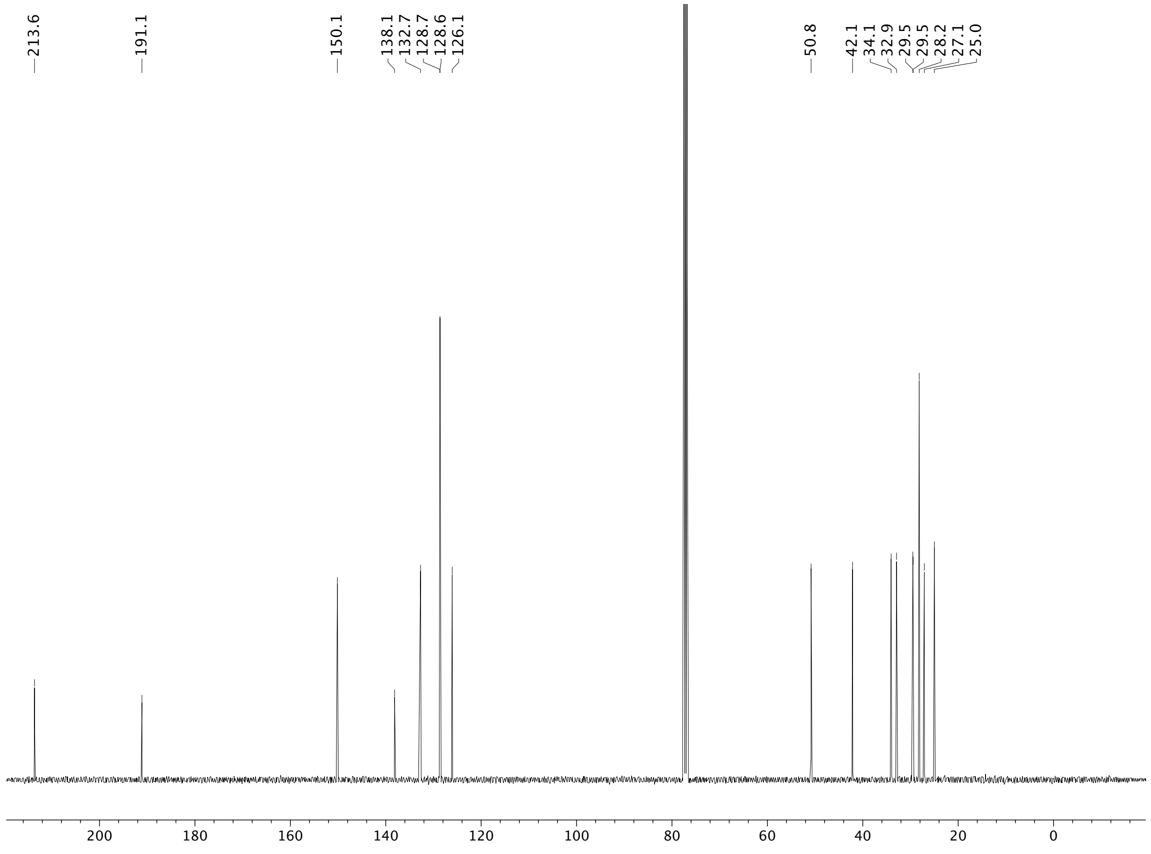

^{13}C NMR (100 MHz, $CDCl_3$) of compound **S2b**.

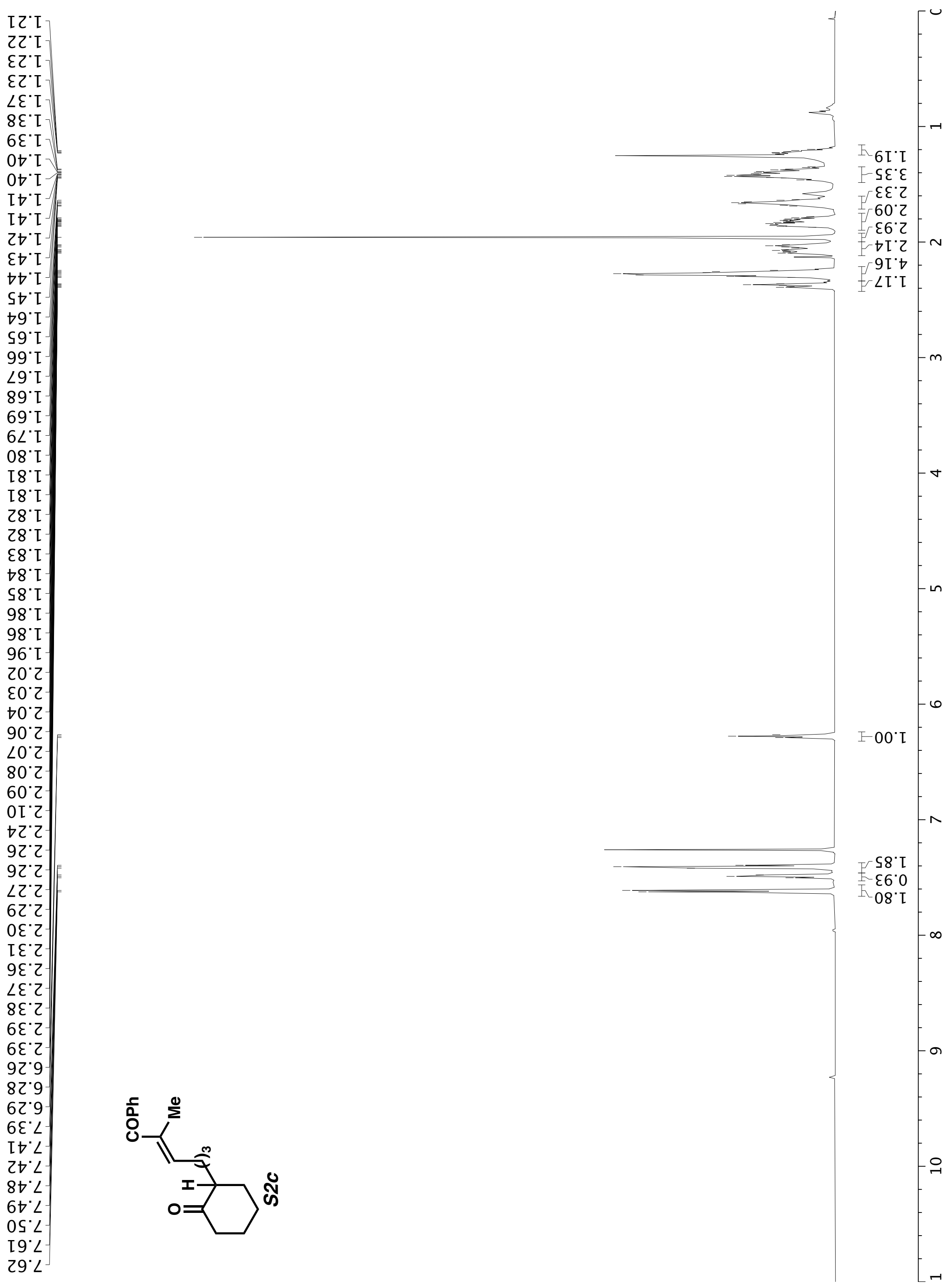

^{1}H NMR (400 MHz, $CDCl_3$) of compound **S2c**.

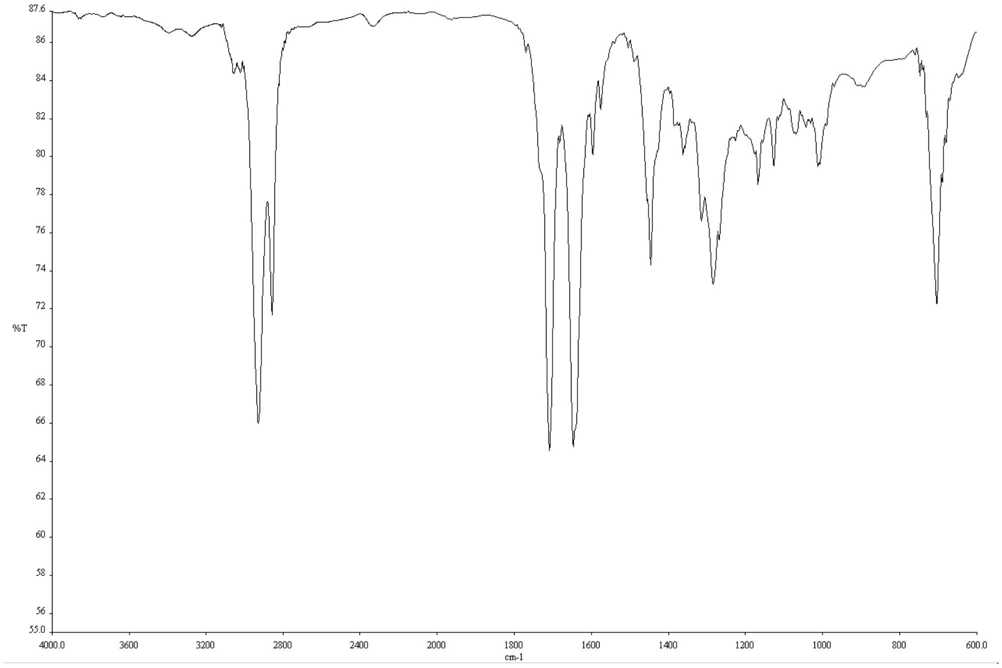

Infrared spectrum (Thin Film, NaCl) of compound **S2c**.

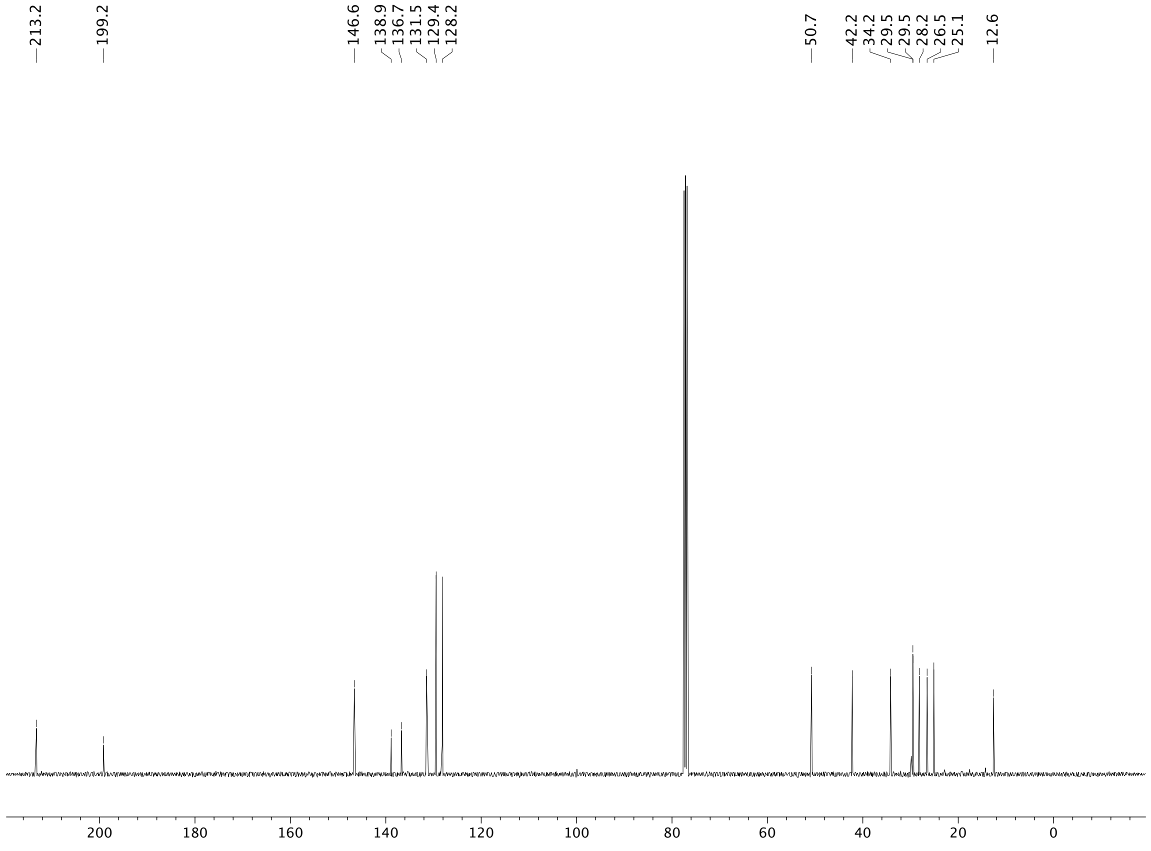

^{13}C NMR (100 MHz, $CDCl_3$) of compound **S2c**.

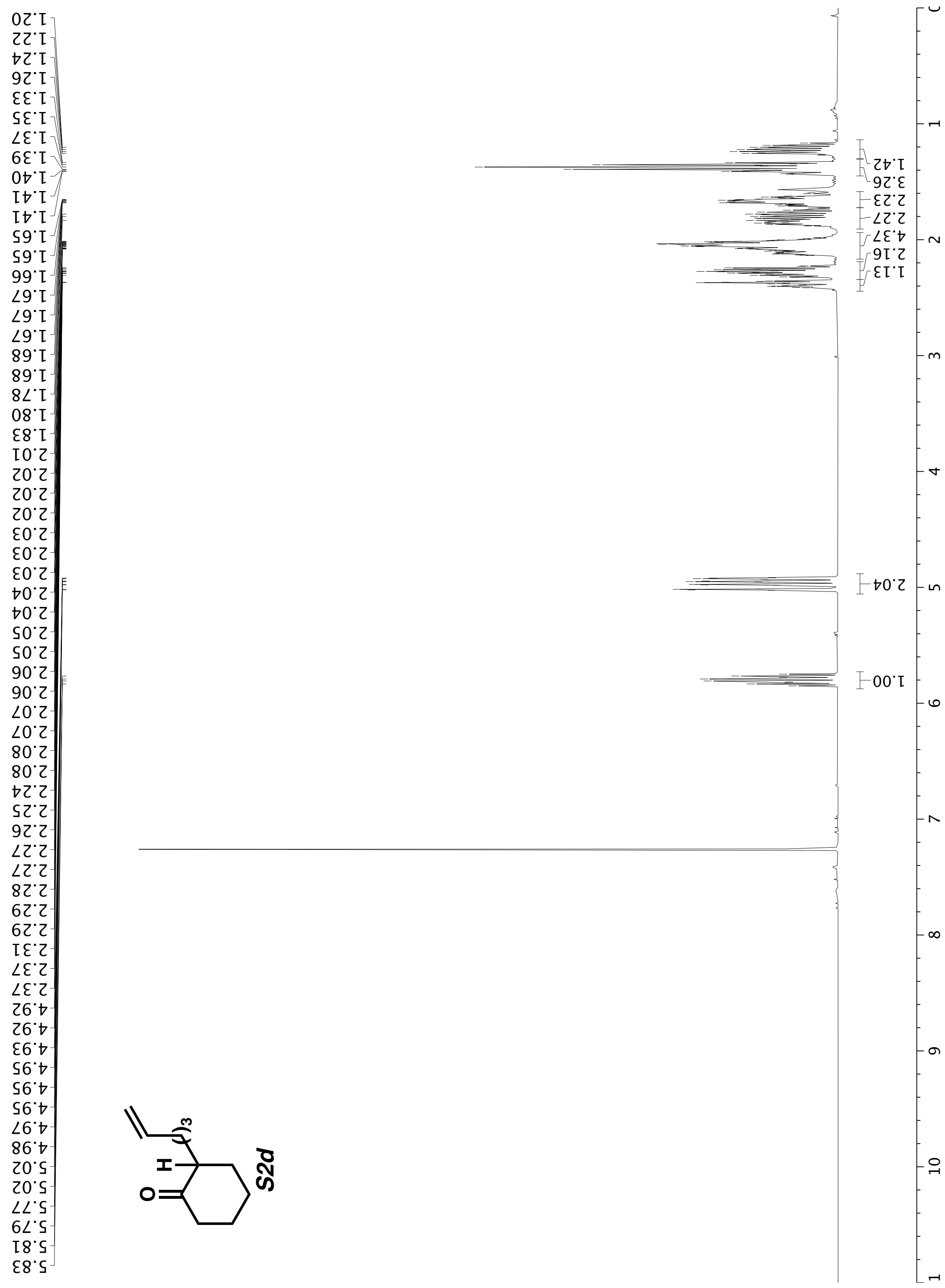

^{1}H NMR (600 MHz, $CDCl_3$) of compound **S2d**.

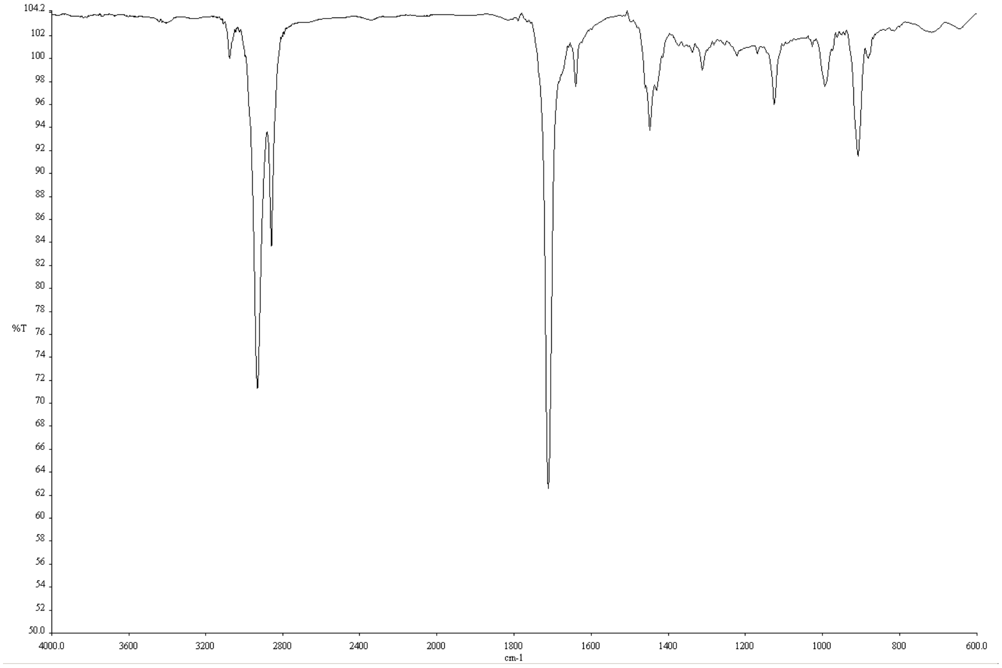

Infrared spectrum (Thin Film, NaCl) of compound **S2d**.

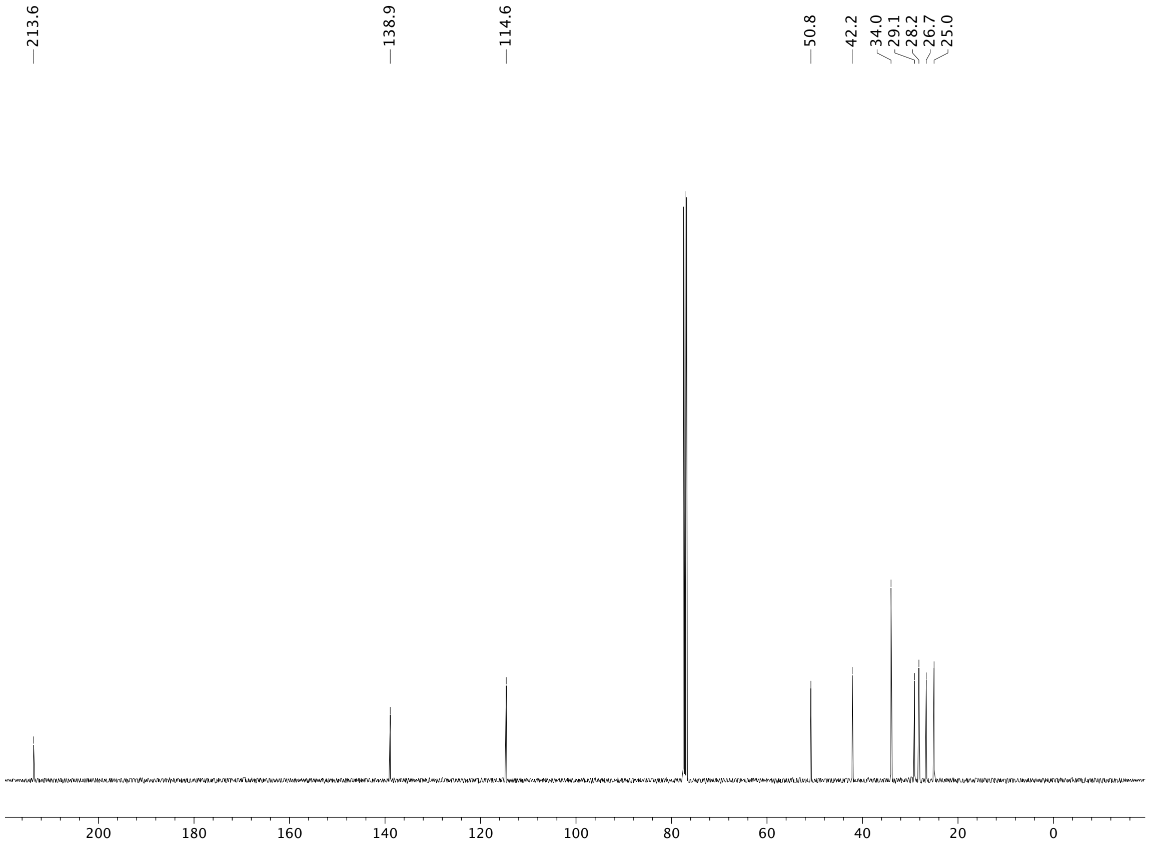

^{13}C NMR (100 MHz, $CDCl_3$) of compound **S2d**.

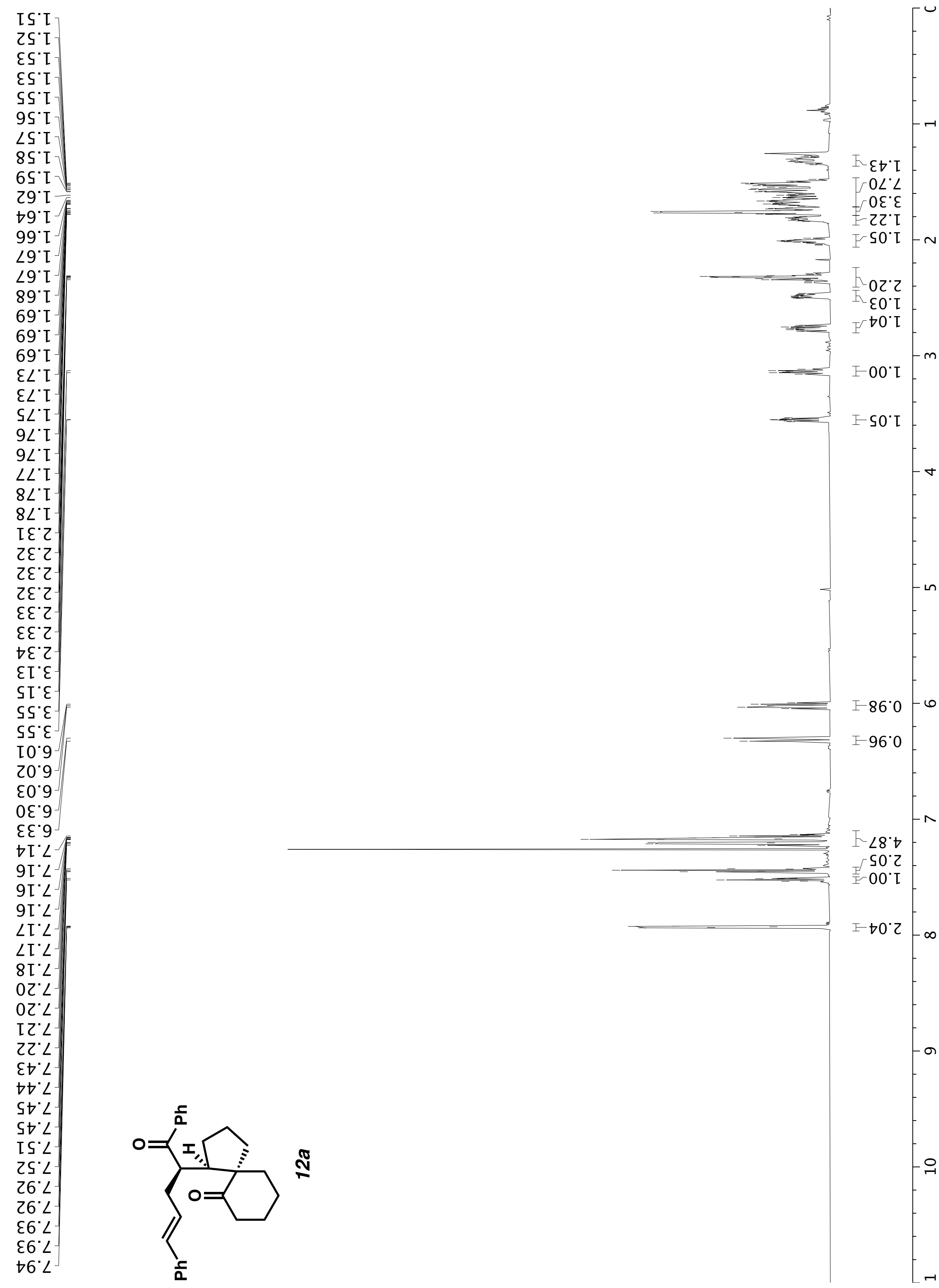

12a

^{1}H NMR (600 MHz, $CDCl_3$) of compound **12a**.

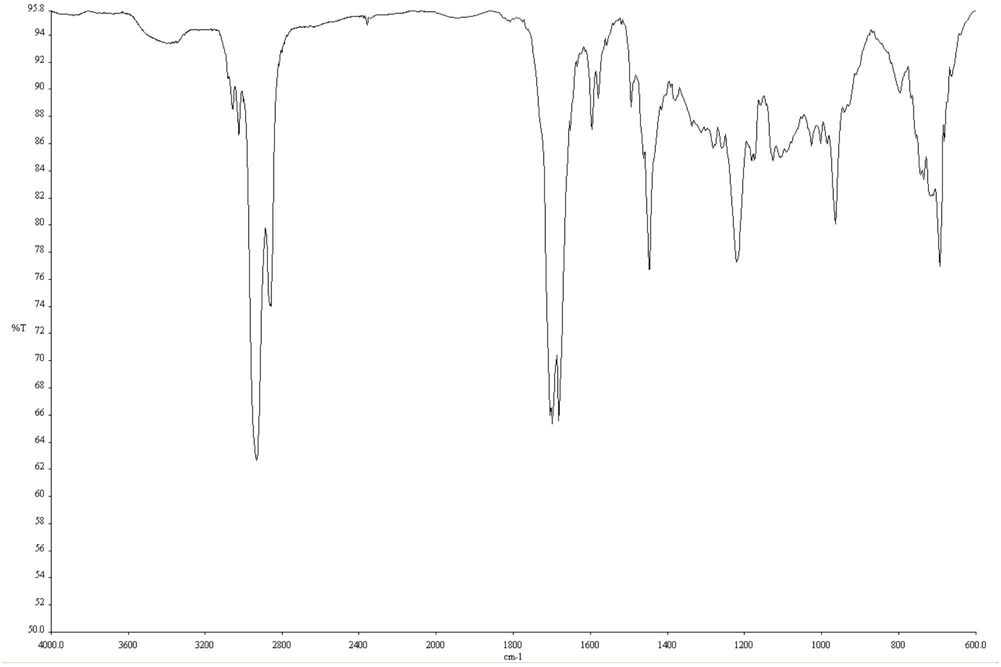

Infrared spectrum (Thin Film, NaCl) of compound **12a**.

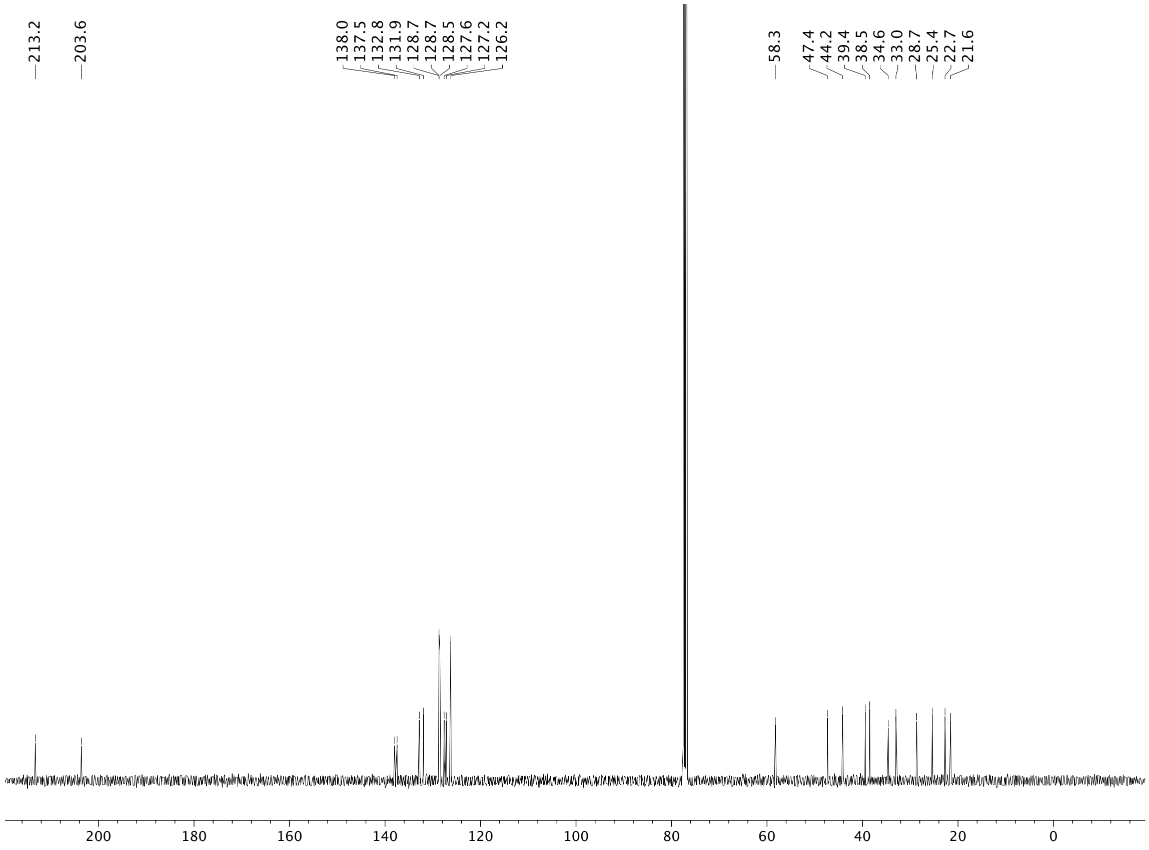

^{13}C NMR (100 MHz, $CDCl_3$) of compound **12a**.

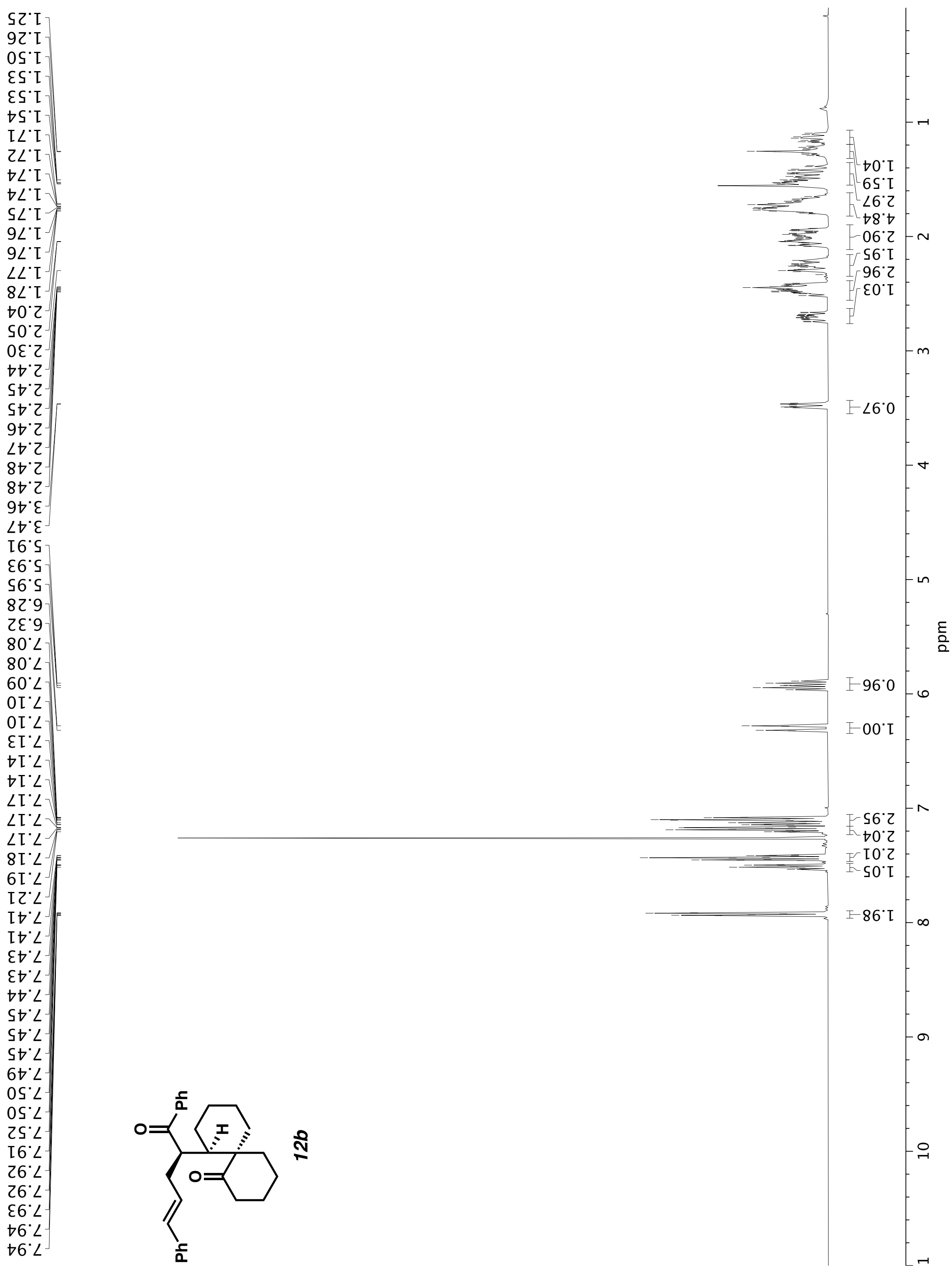

^{1}H NMR (400 MHz, $CDCl_3$) of compound **12b**.

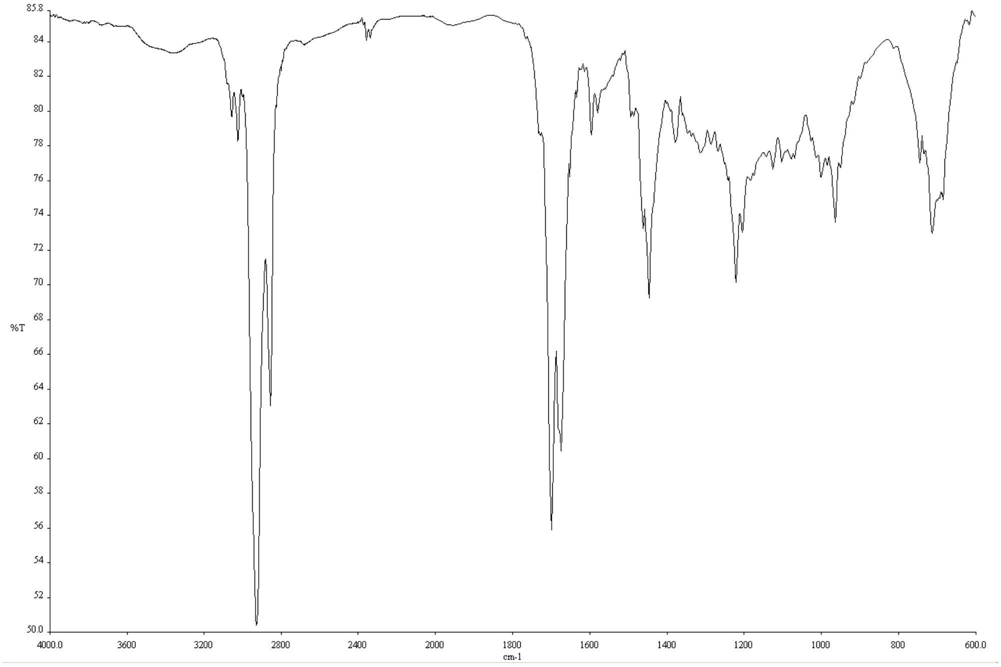

Infrared spectrum (Thin Film, NaCl) of compound **12b**.

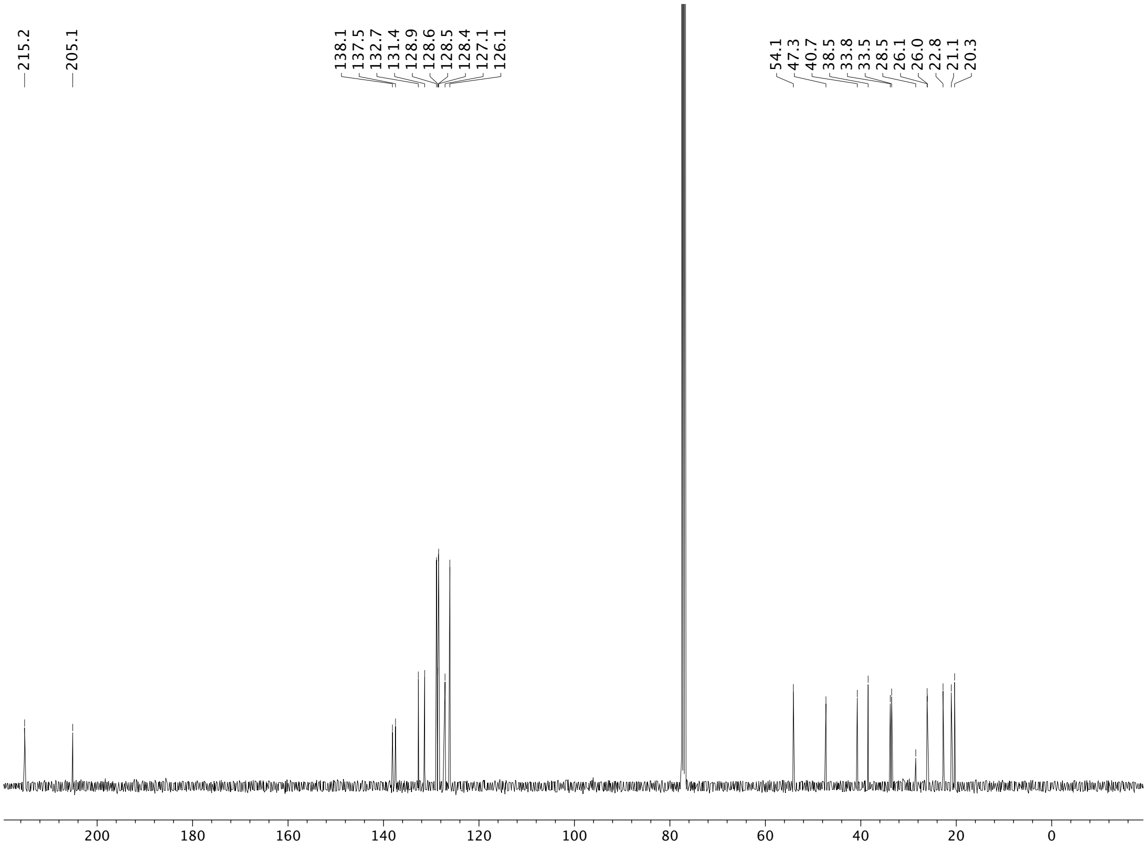

^{13}C NMR (100 MHz, $CDCl_3$) of compound **12b**.

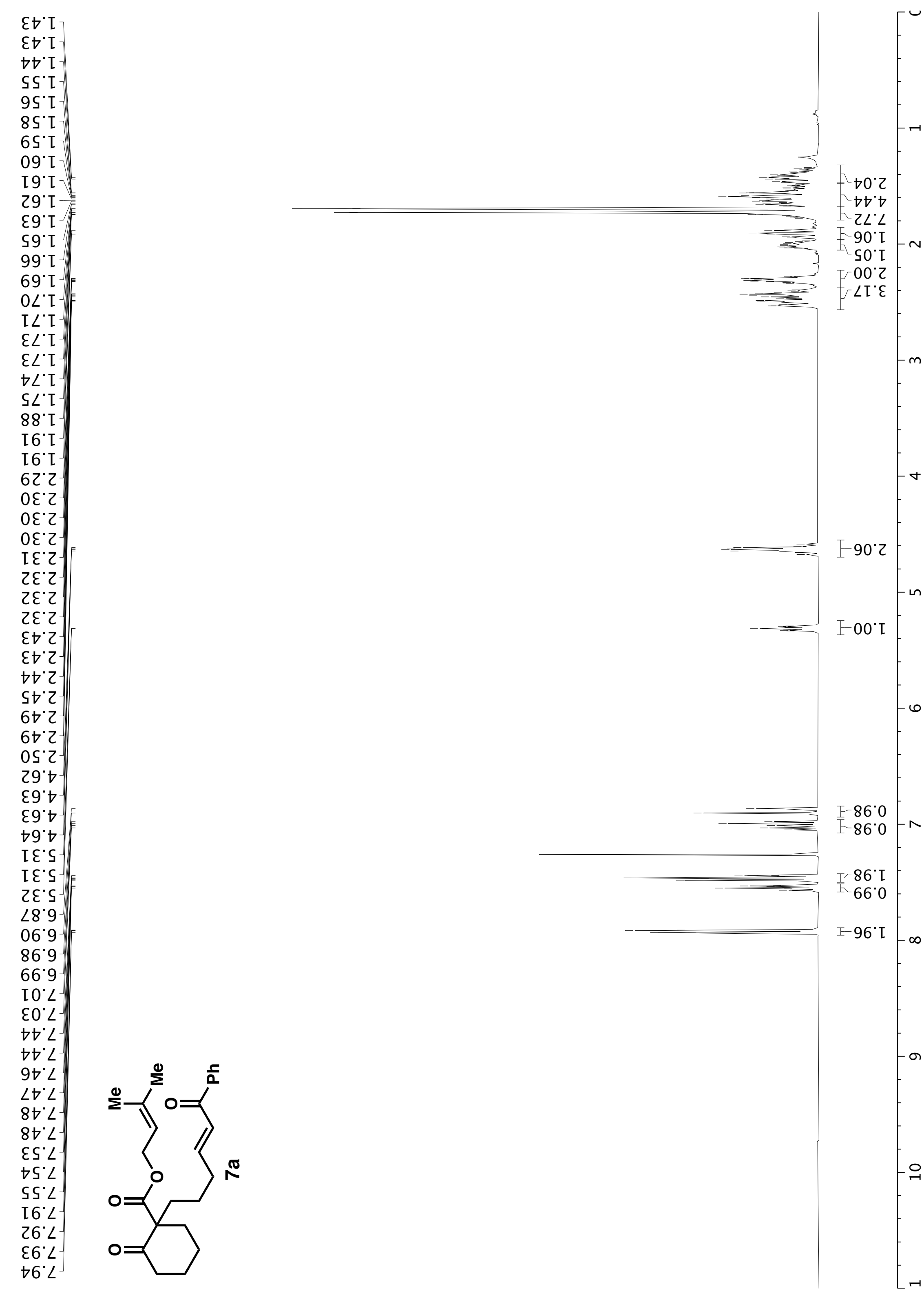

^{1}H NMR (400 MHz, $CDCl_3$) of compound **7a**.

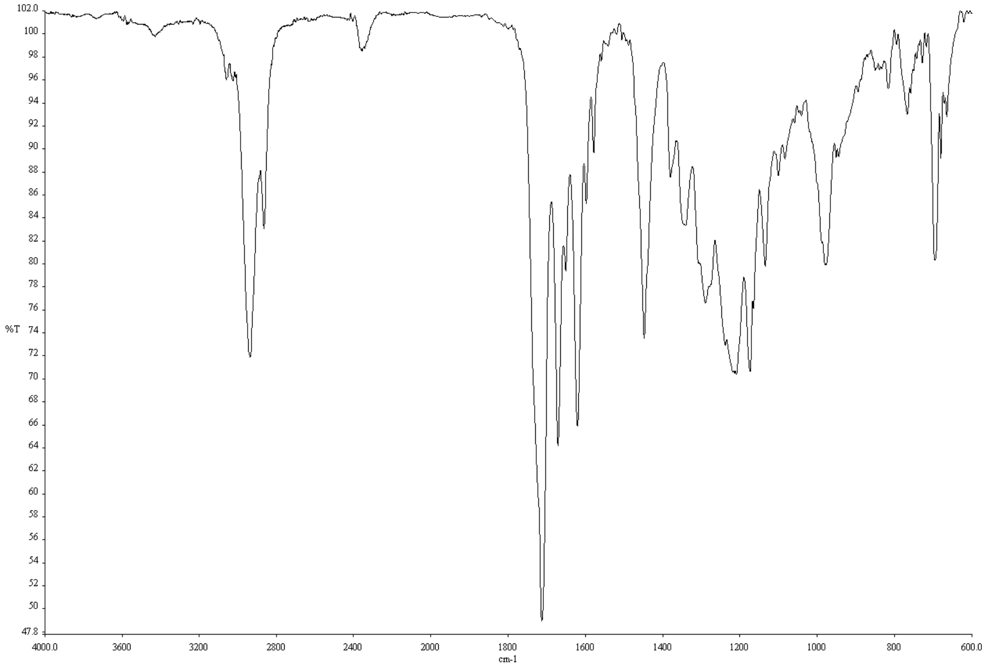

Infrared spectrum (Thin Film, NaCl) of compound **7a**.

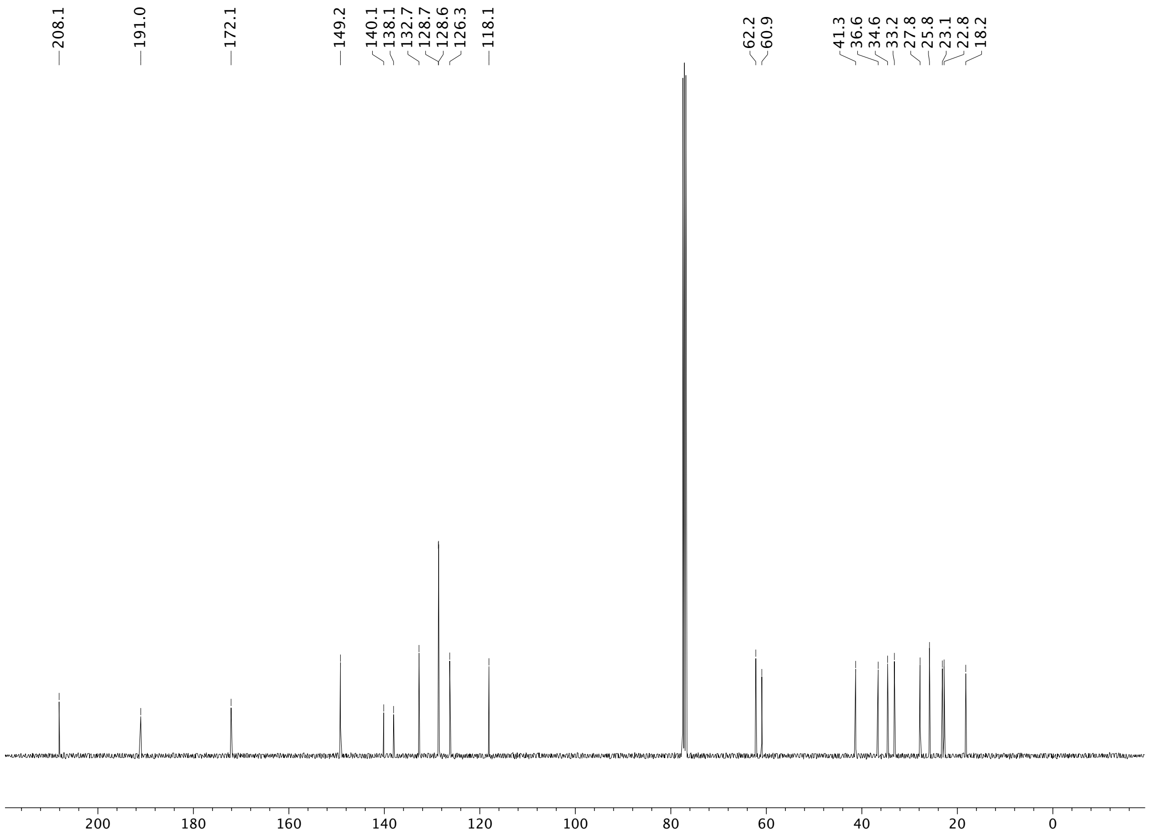

^{13}C NMR (100 MHz, $CDCl_3$) of compound **7a**.

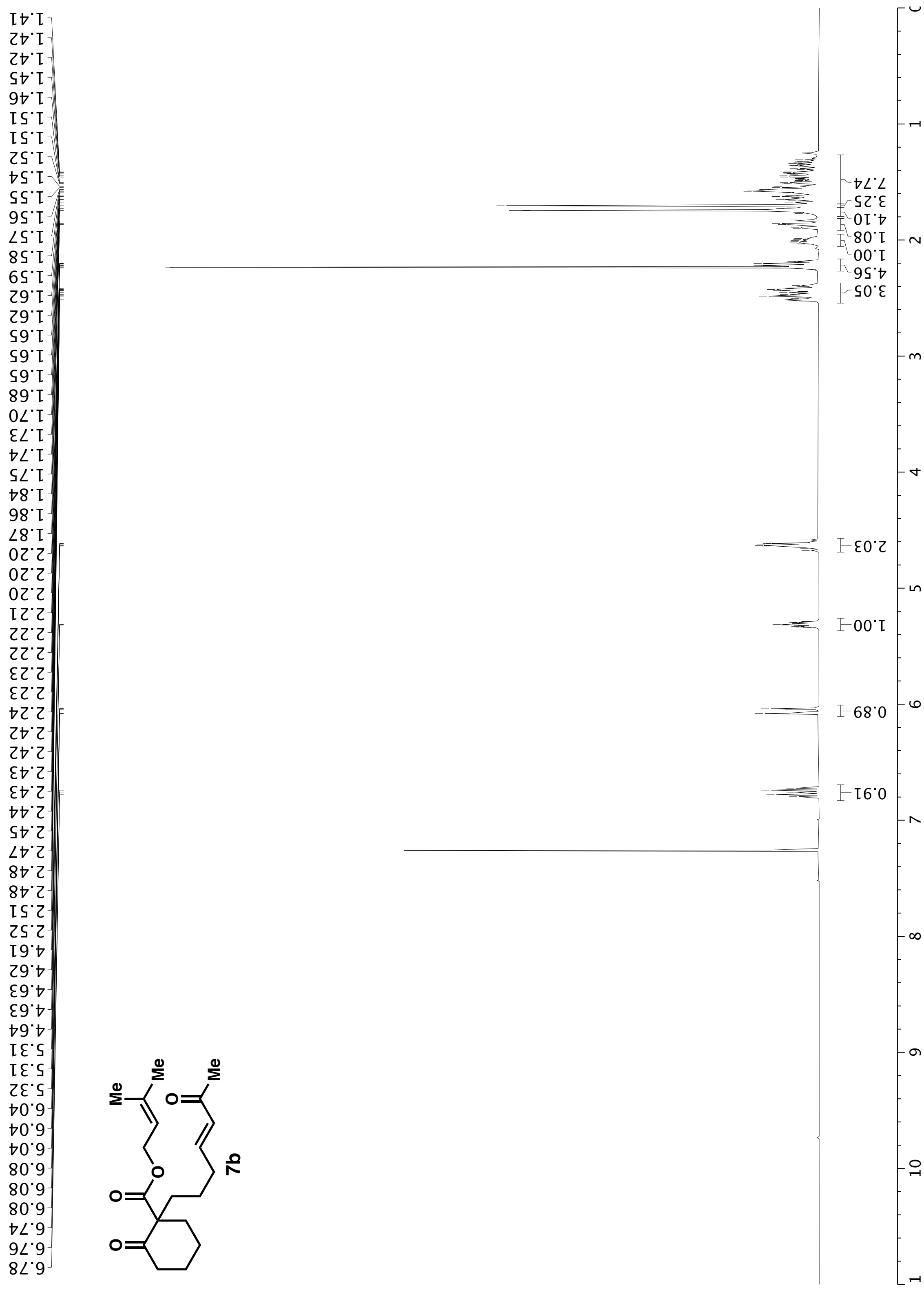

^{1}H NMR (400 MHz, $CDCl_3$) of compound **7b**.

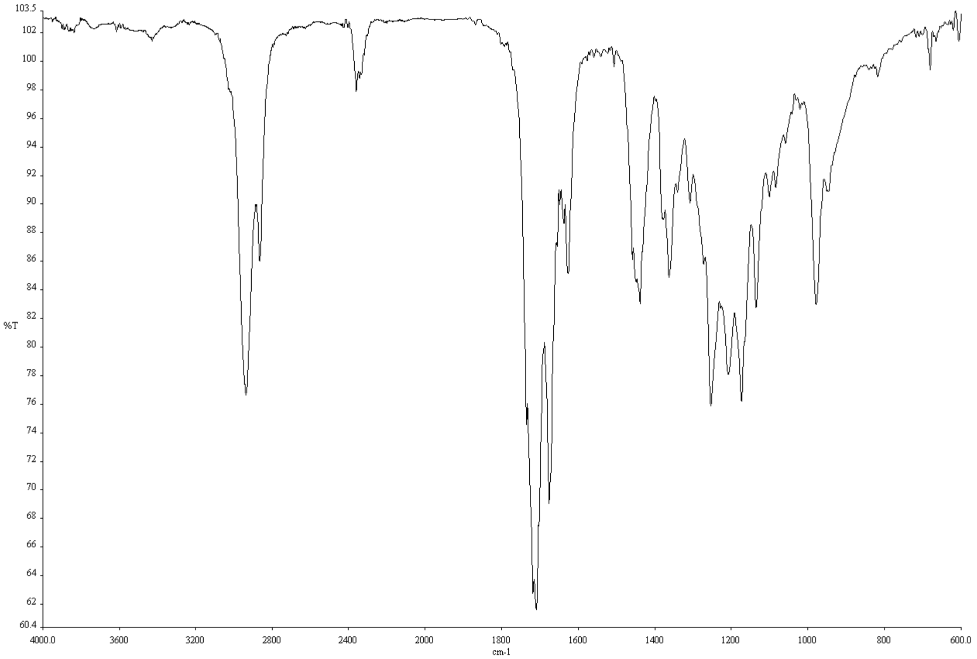

Infrared spectrum (Thin Film, NaCl) of compound **7b**.

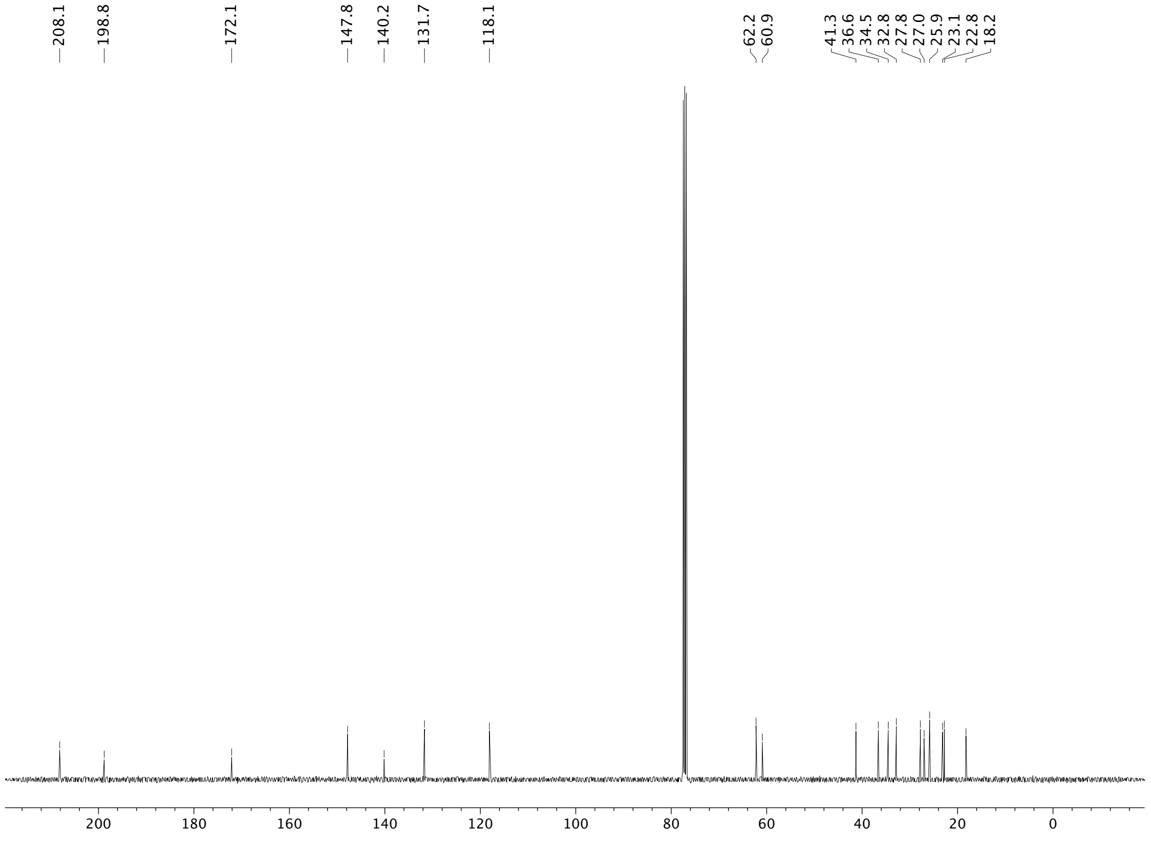

^{13}C NMR (100 MHz, $CDCl_3$) of compound **7b**.

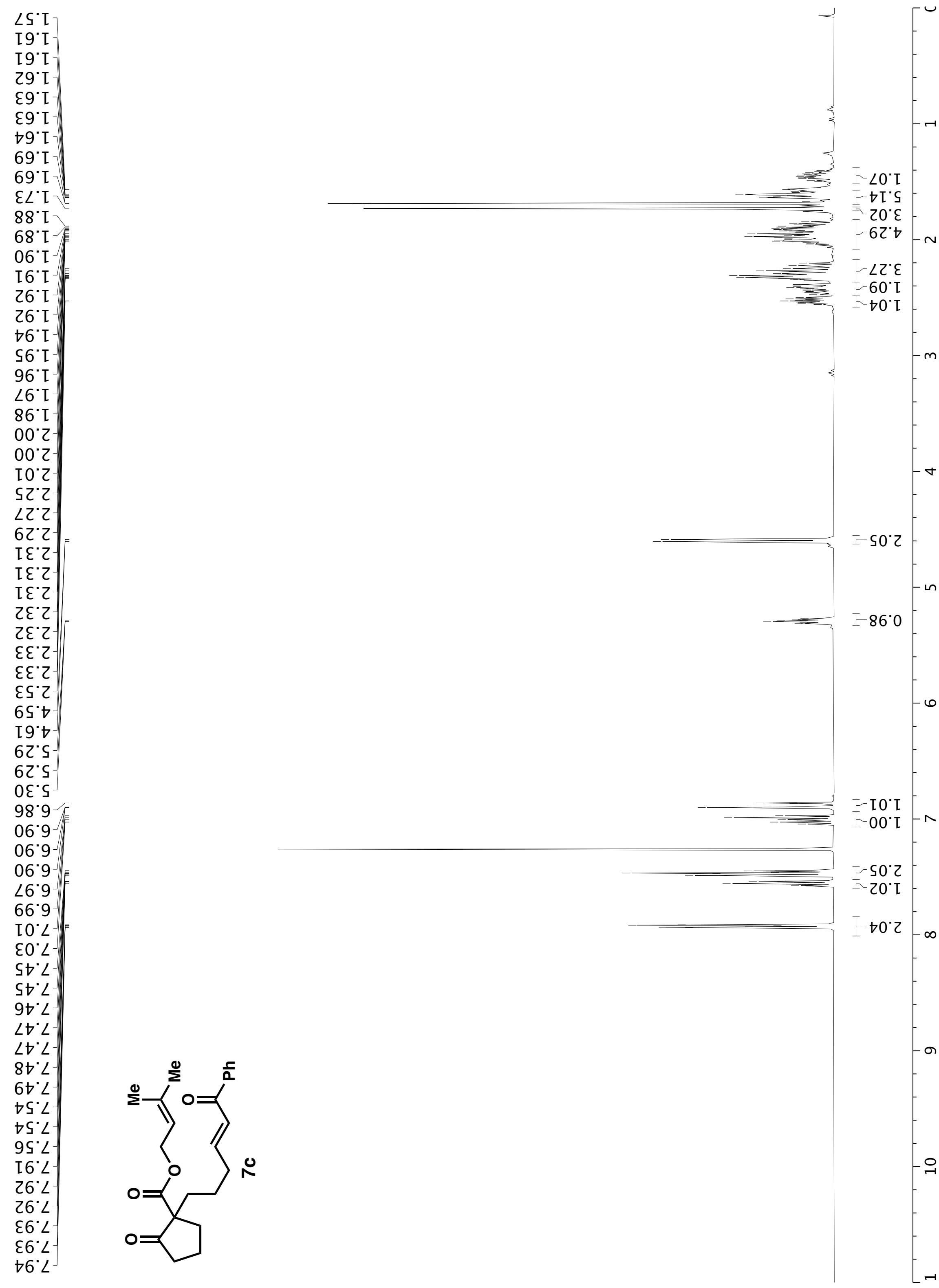

^{1}H NMR (400 MHz, $CDCl_3$) of compound **7c**.

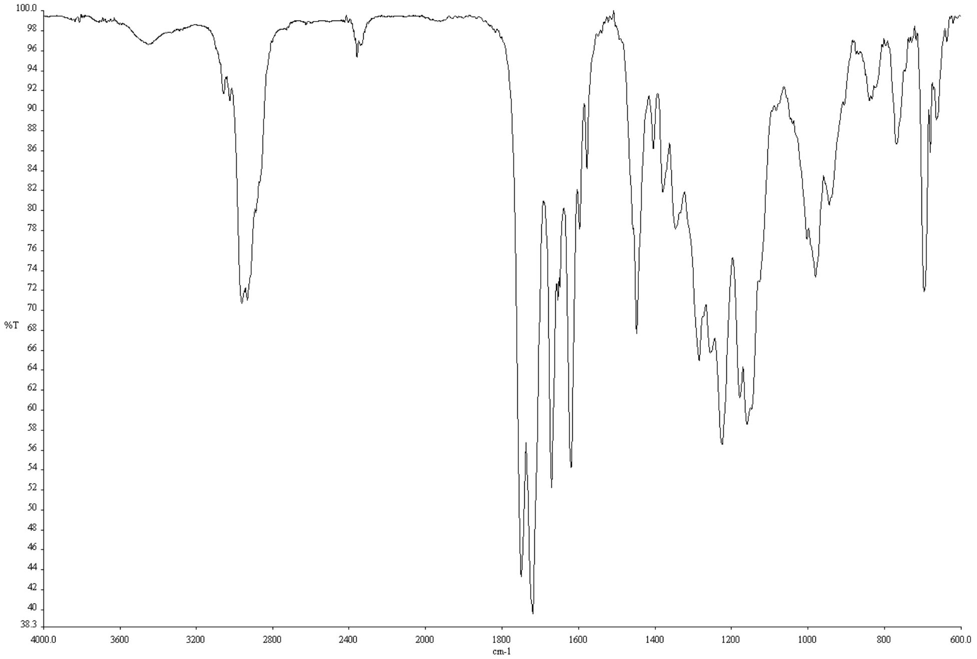

Infrared spectrum (Thin Film, NaCl) of compound **7c**.

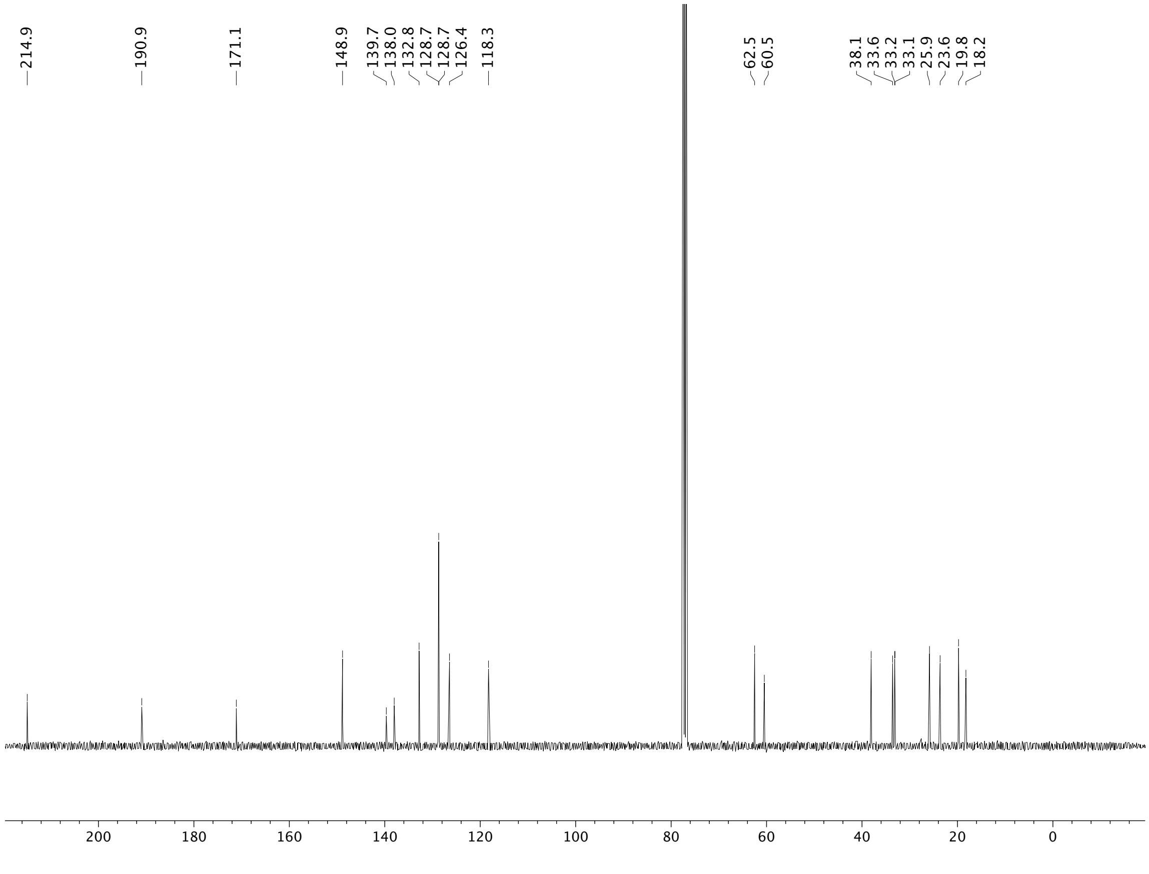

^{13}C NMR (100 MHz, $CDCl_3$) of compound **7c**.

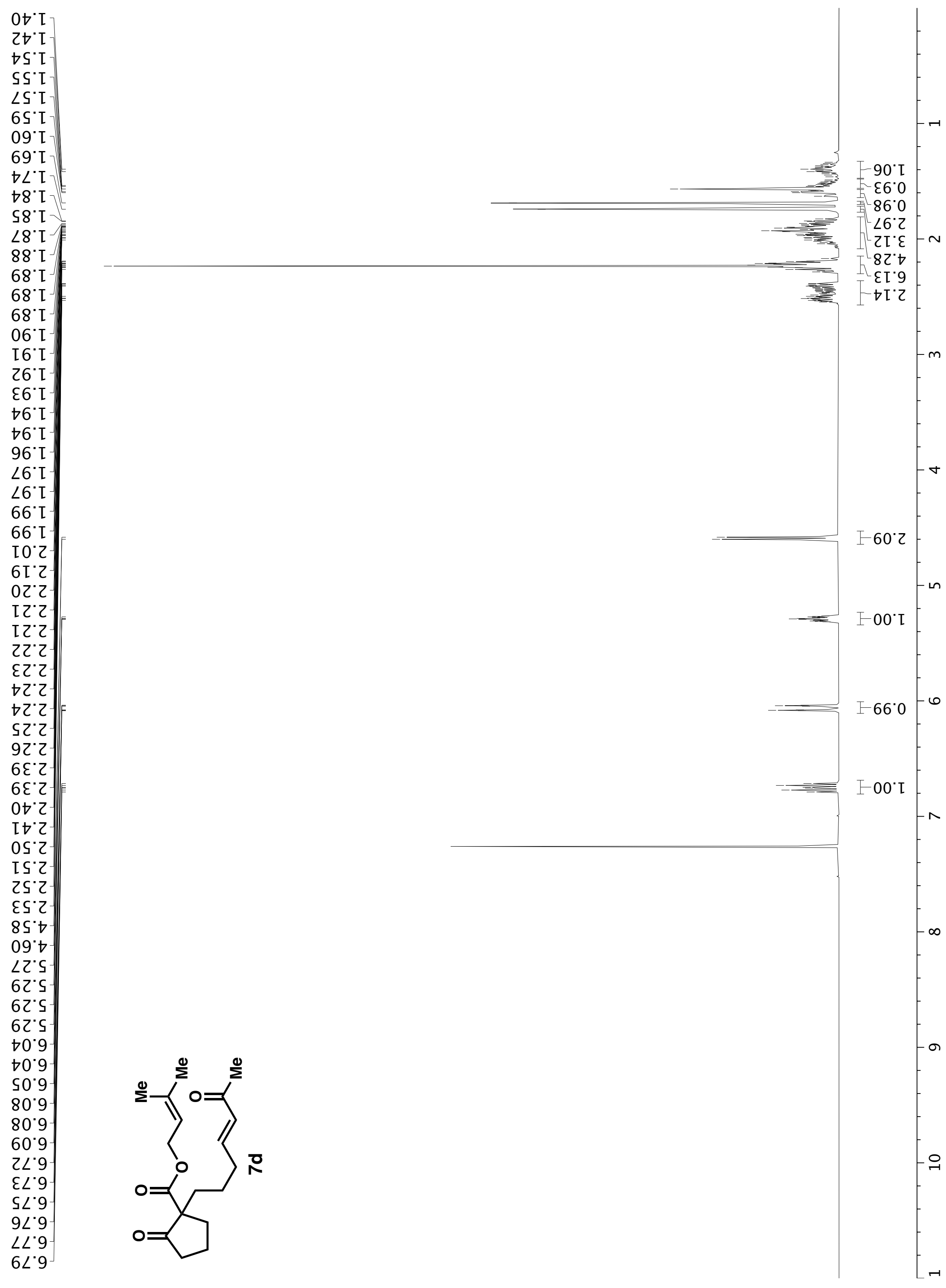

^{1}H NMR (400 MHz, $CDCl_3$) of compound **7d**.

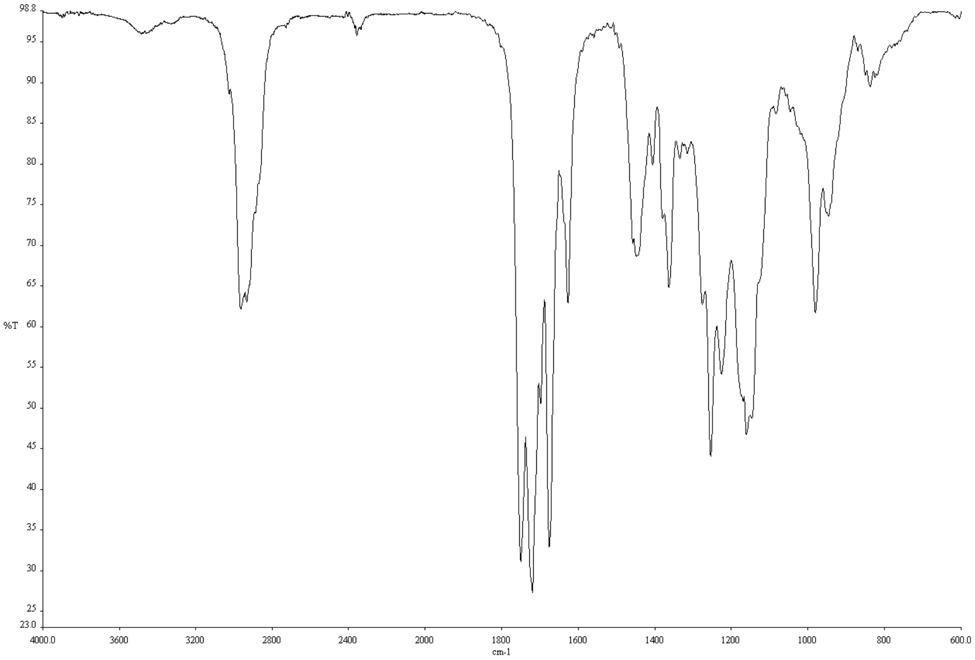

Infrared spectrum (Thin Film, NaCl) of compound **7d**.

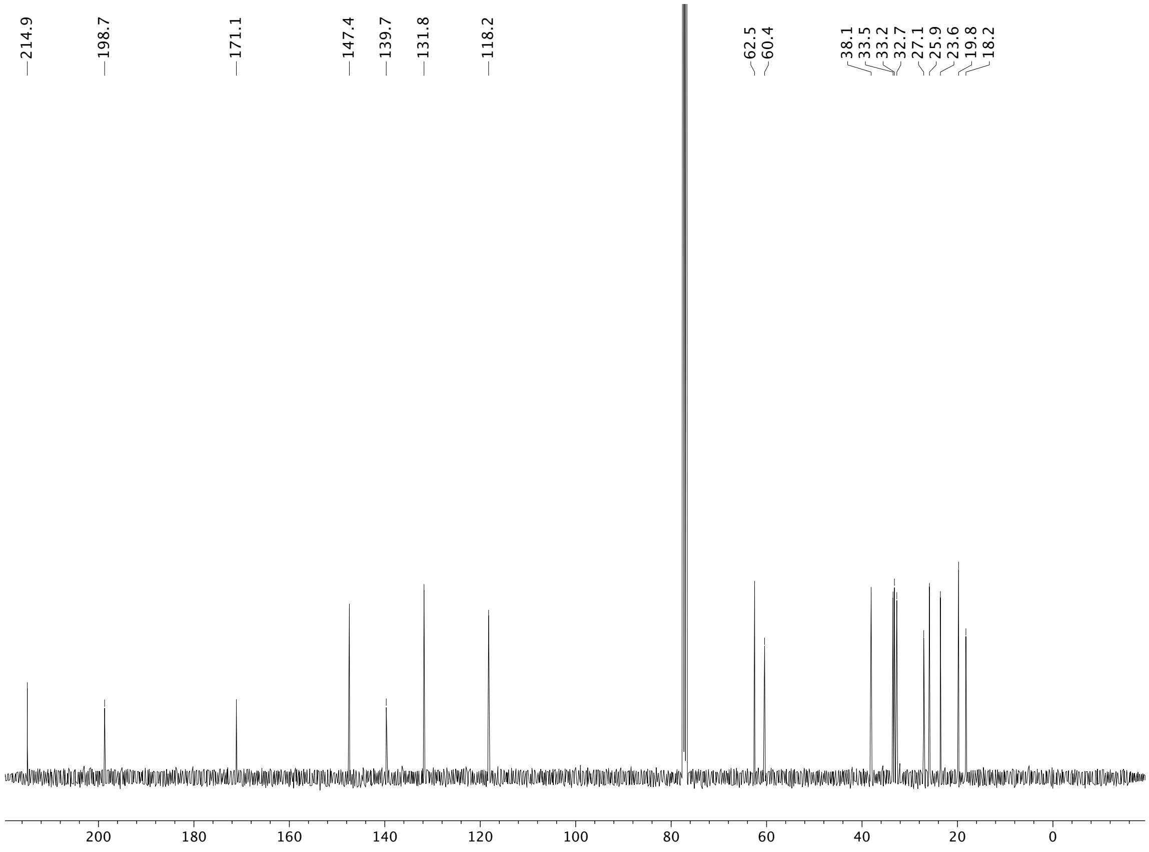

^{13}C NMR (100 MHz, $CDCl_3$) of compound **7d**.

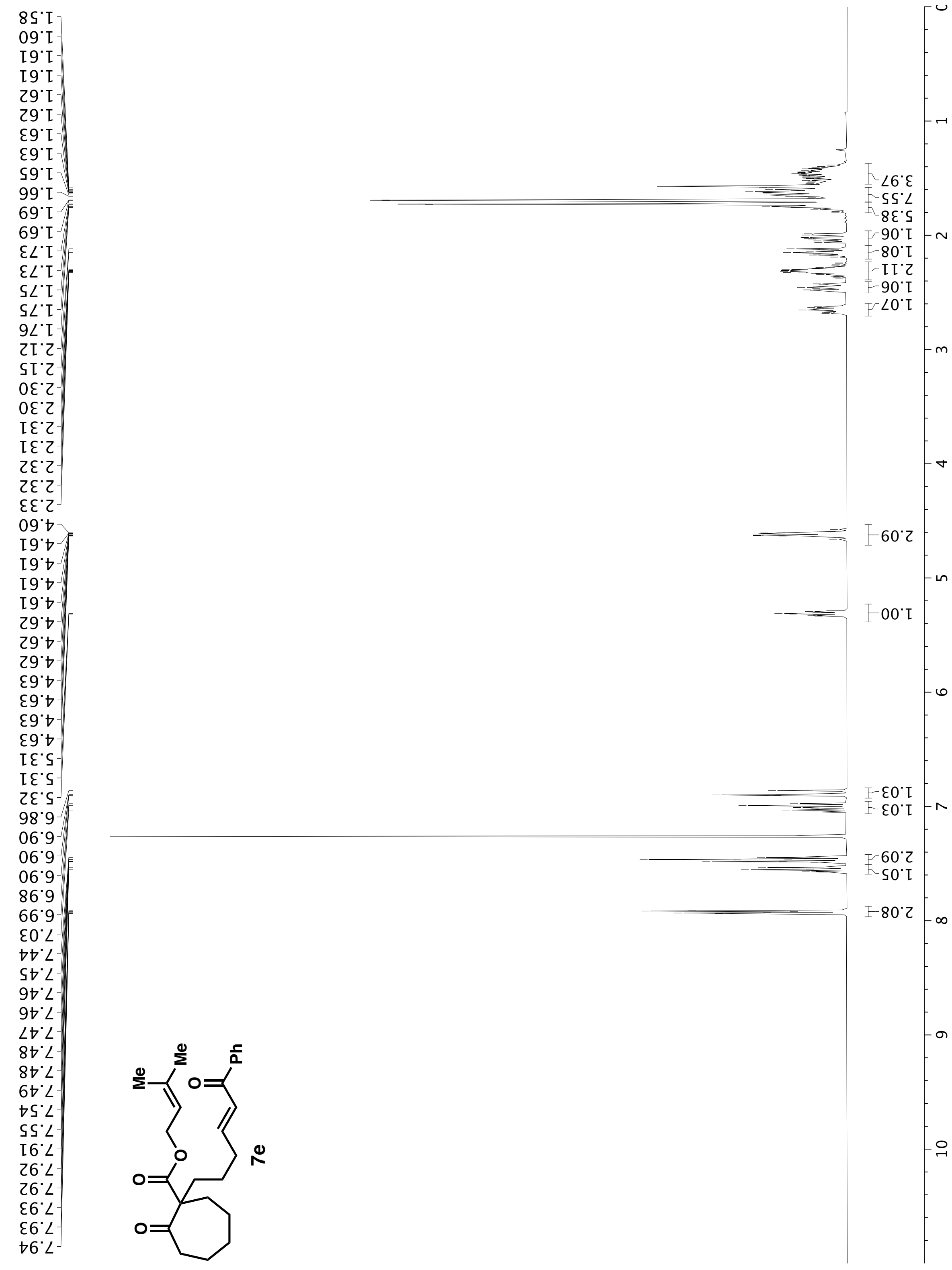

^{1}H NMR (400 MHz, $CDCl_3$) of compound **7e**.

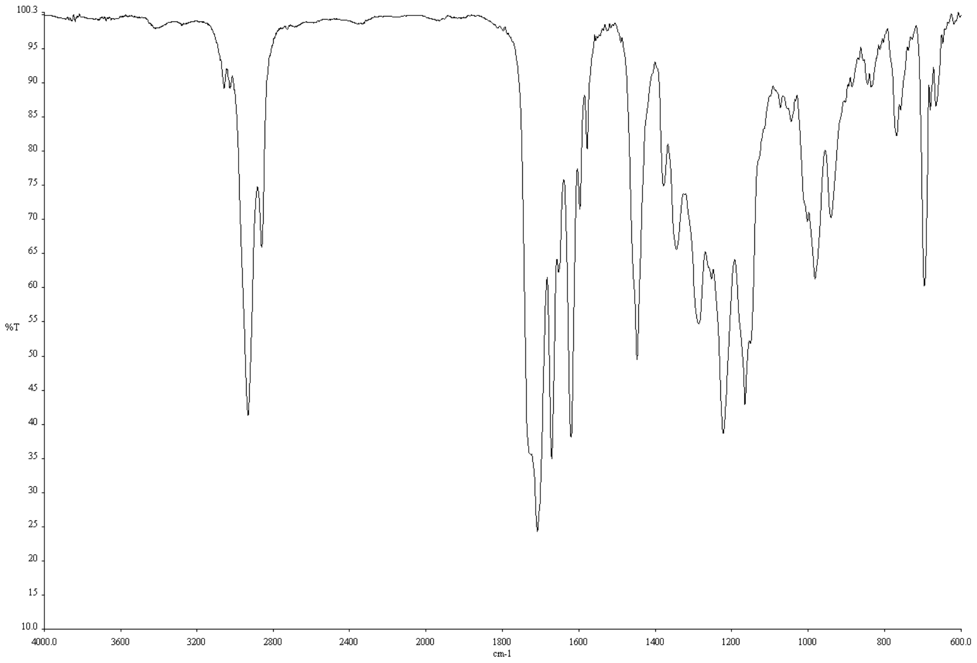

Infrared spectrum (Thin Film, NaCl) of compound **7e**.

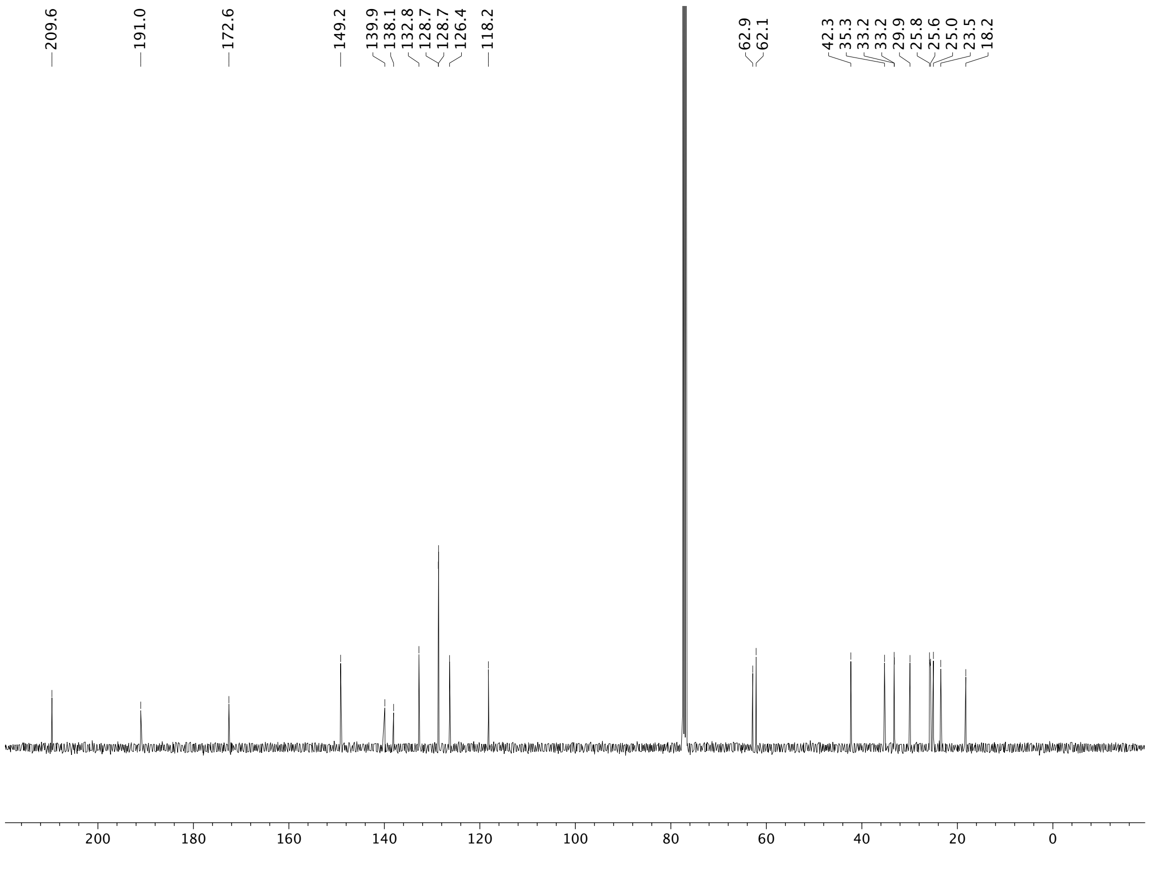

^{13}C NMR (100 MHz, $CDCl_3$) of compound **7e**.

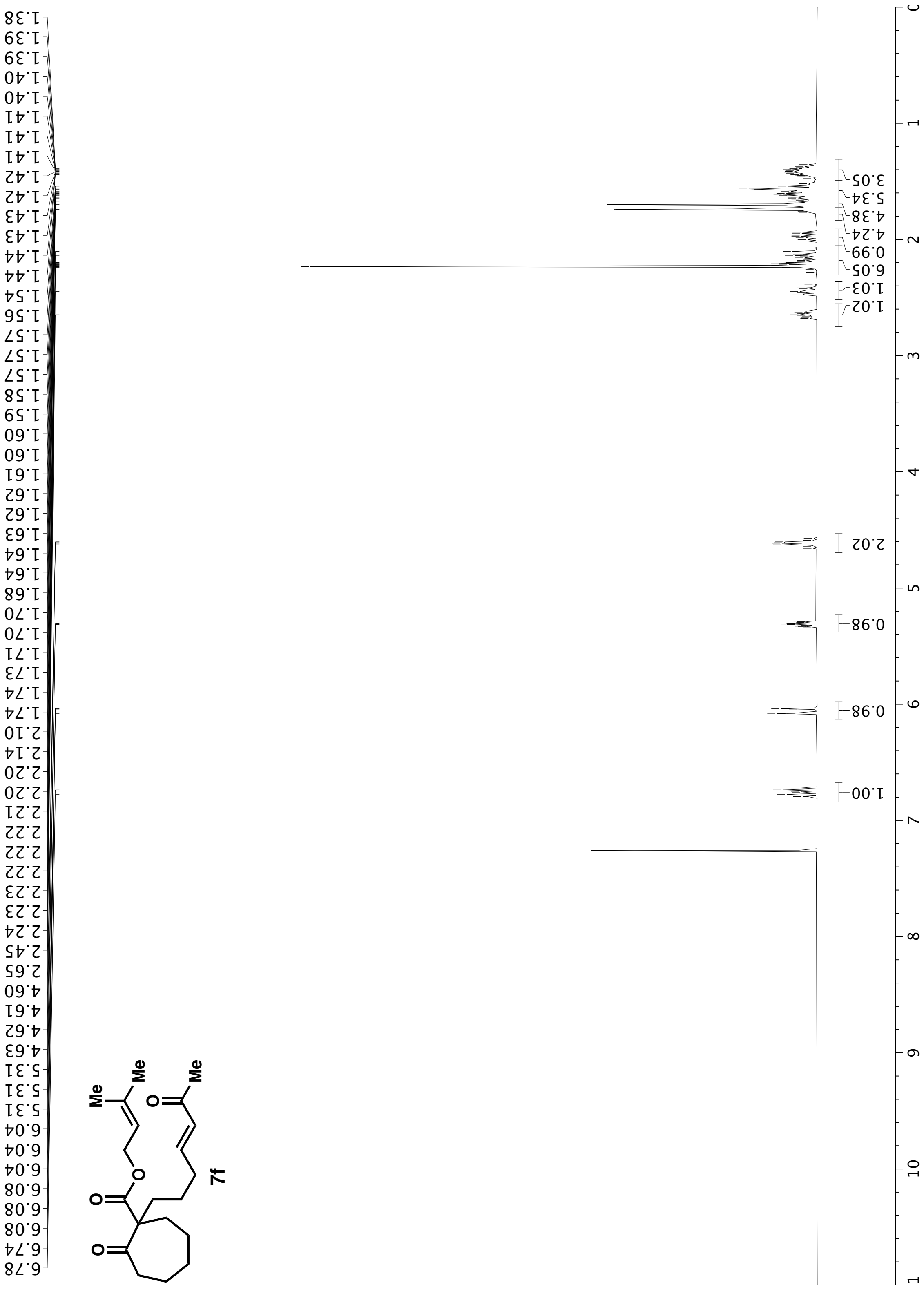

^{1}H NMR (400 MHz, $CDCl_3$) of compound **7f**.

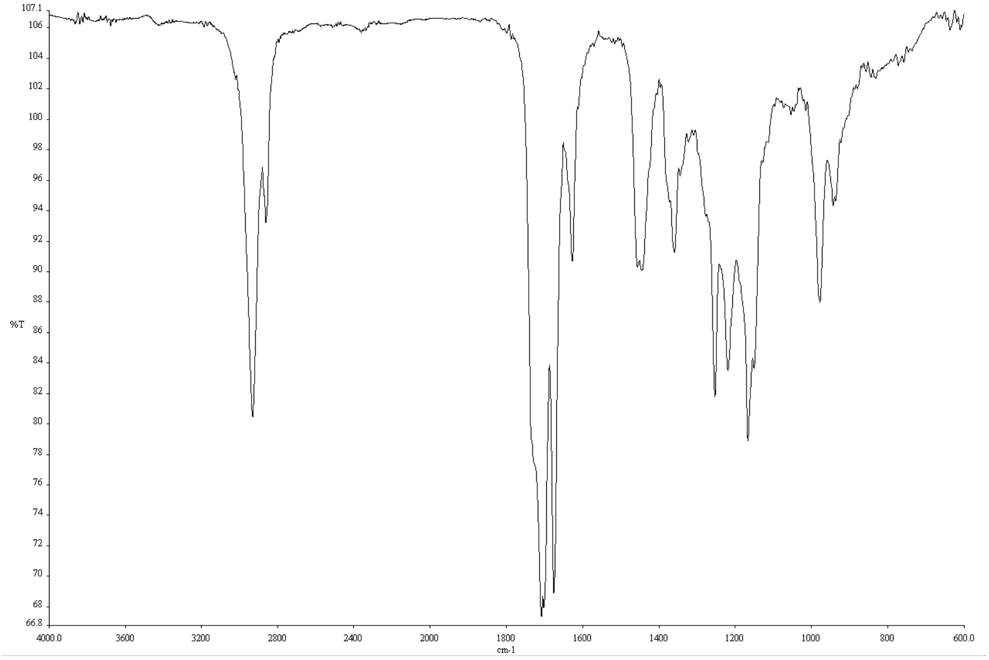

Infrared spectrum (Thin Film, NaCl) of compound **7f**.

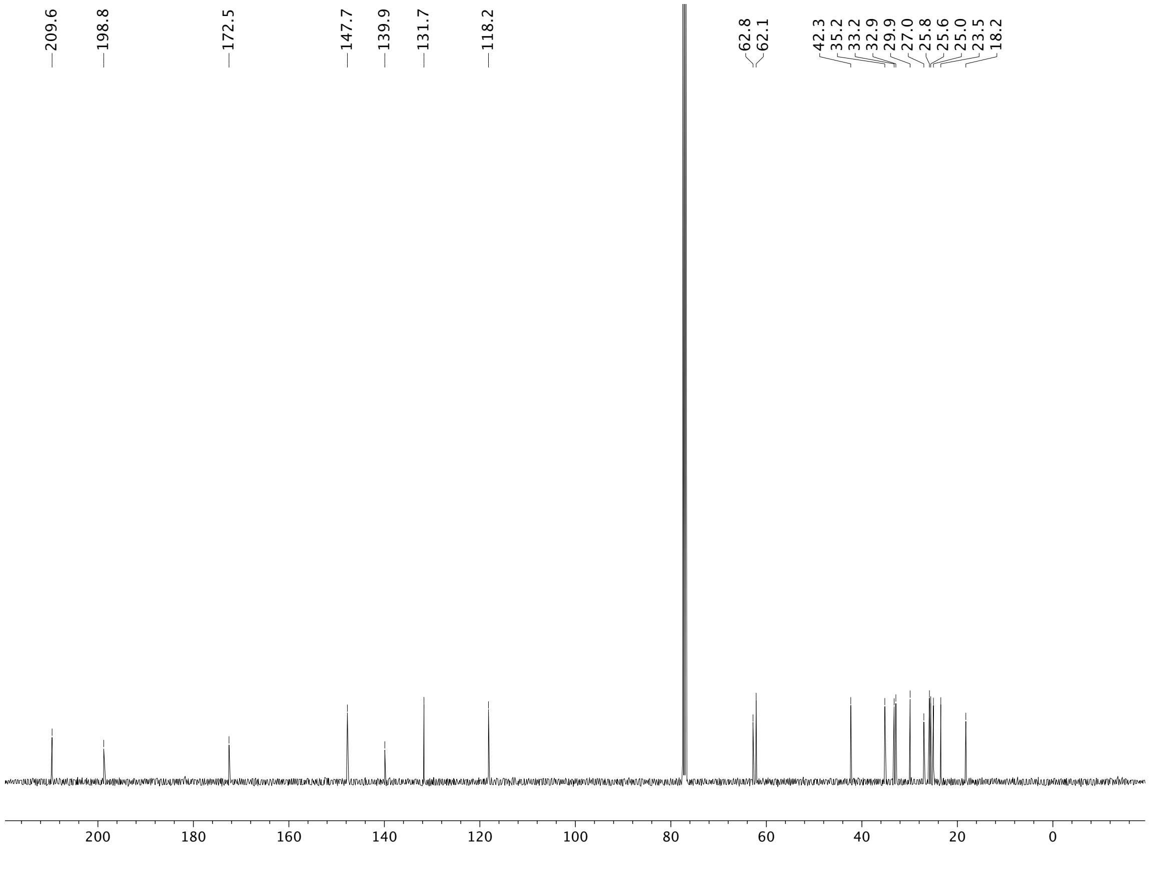

^{13}C NMR (100 MHz, $CDCl_3$) of compound **7f**.

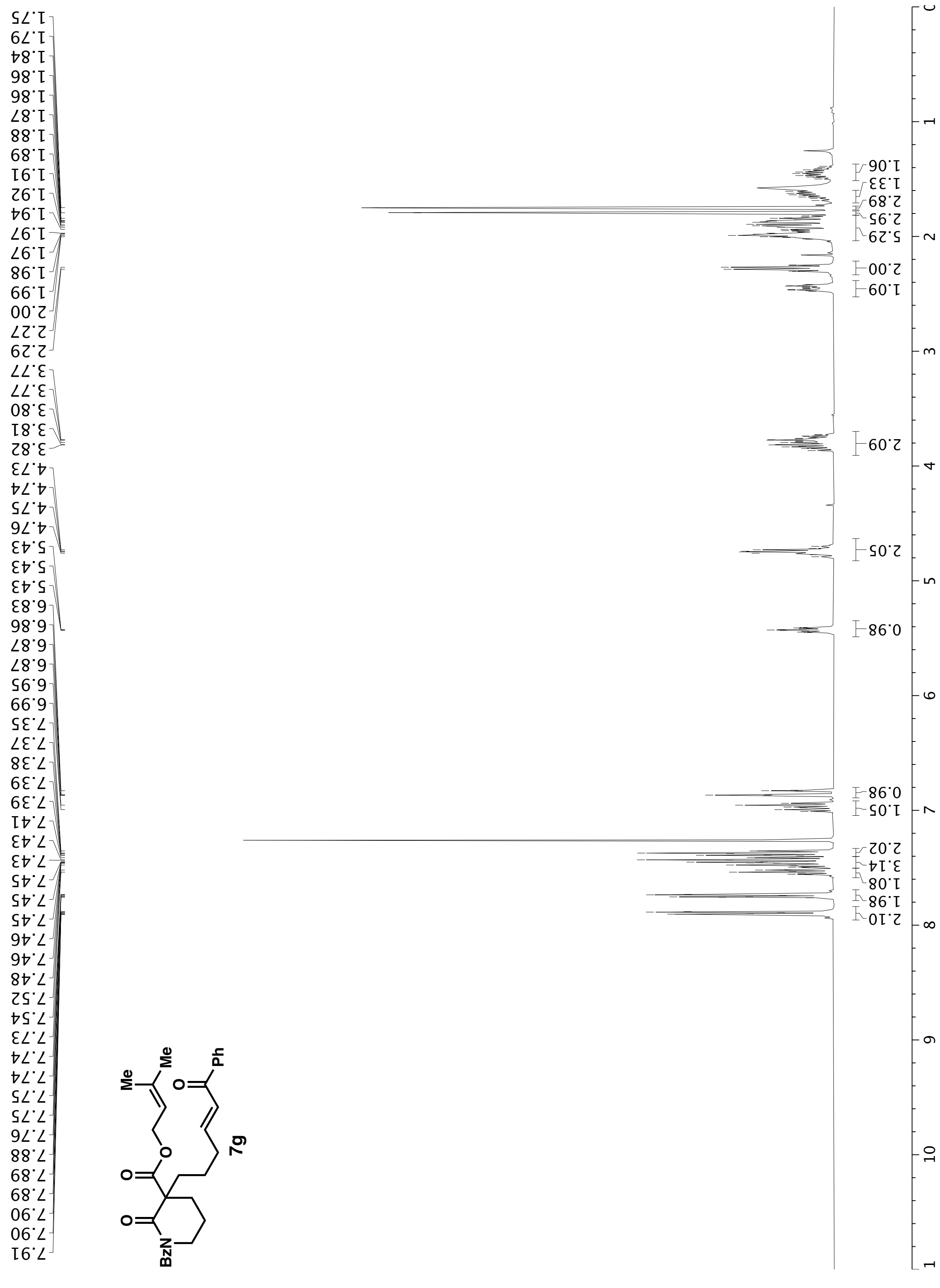

^{1}H NMR (400 MHz, $CDCl_3$) of compound **7g**.

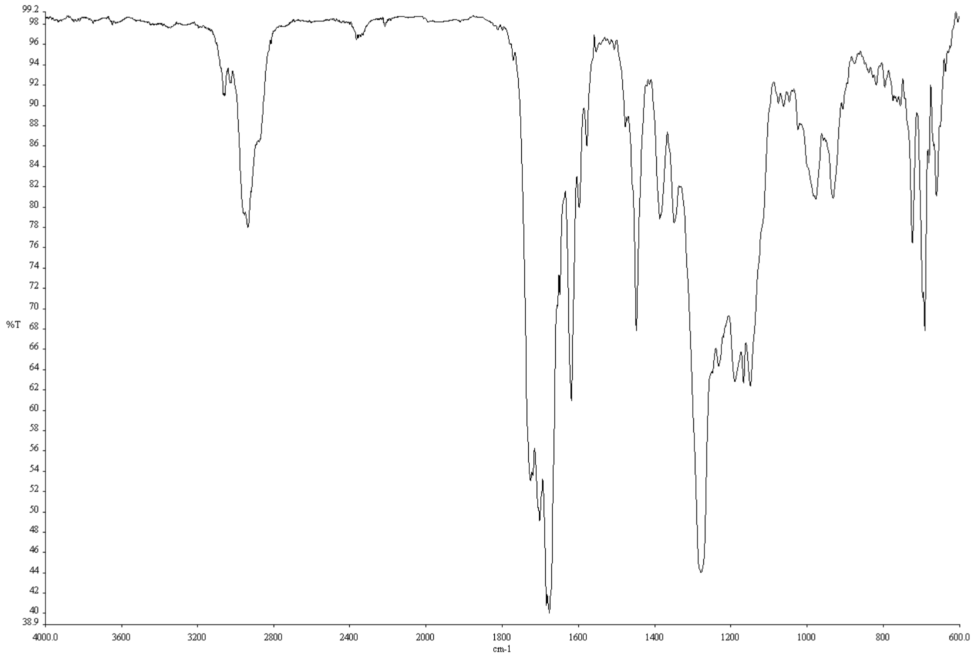

Infrared spectrum (Thin Film, NaCl) of compound **7g**.

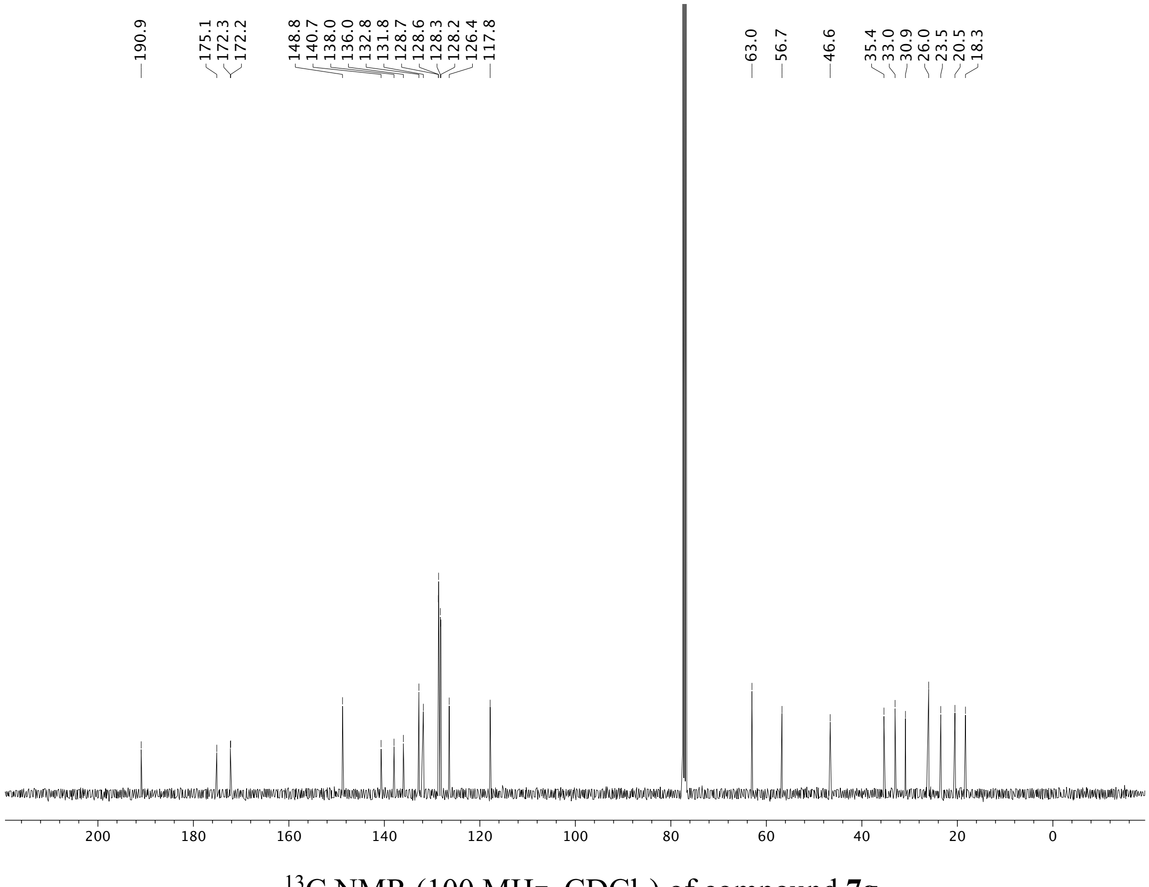

^{13}C NMR (100 MHz, $CDCl_3$) of compound **7g**.

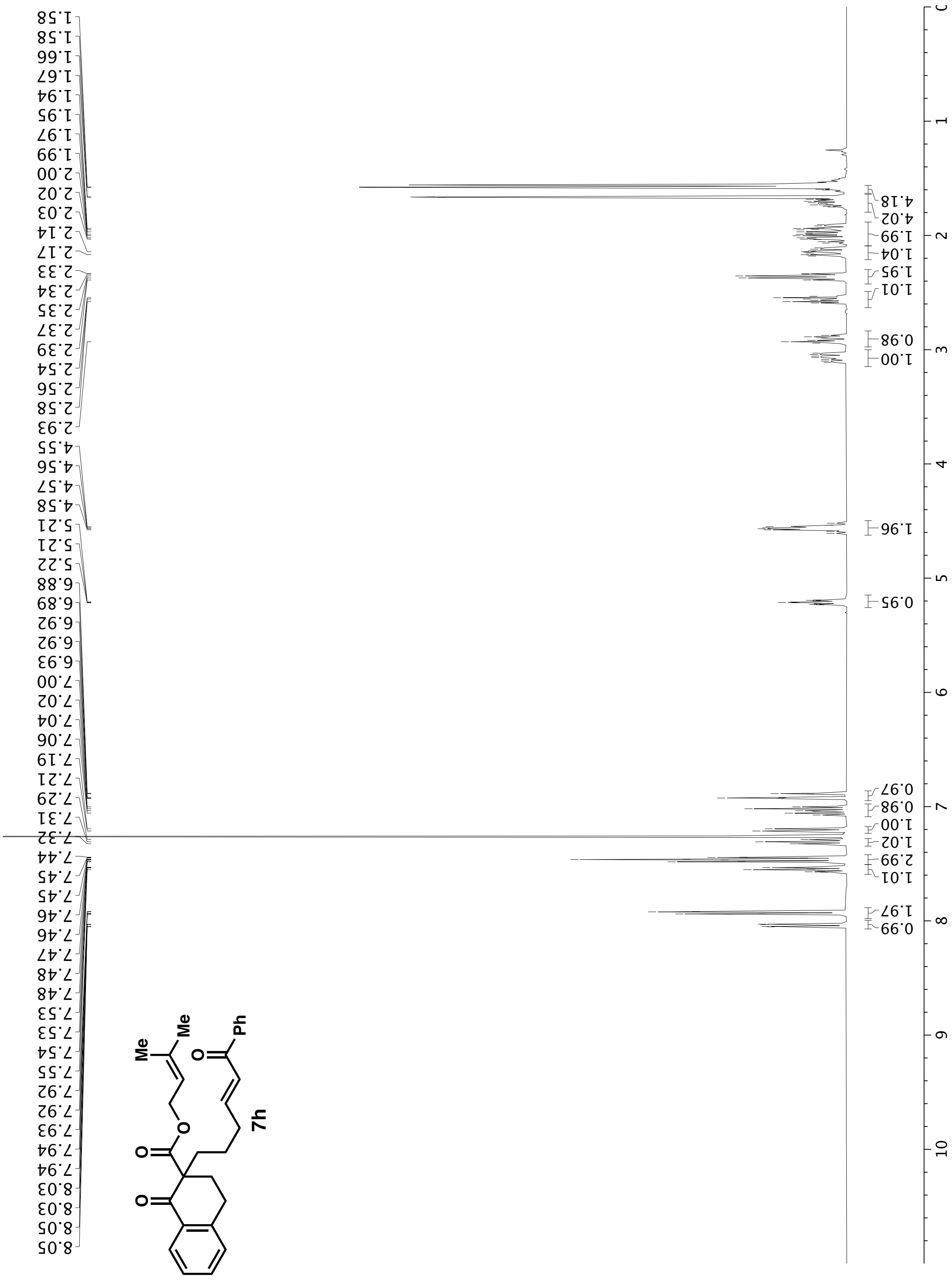

^{1}H NMR (400 MHz, $CDCl_3$) of compound **7h**.

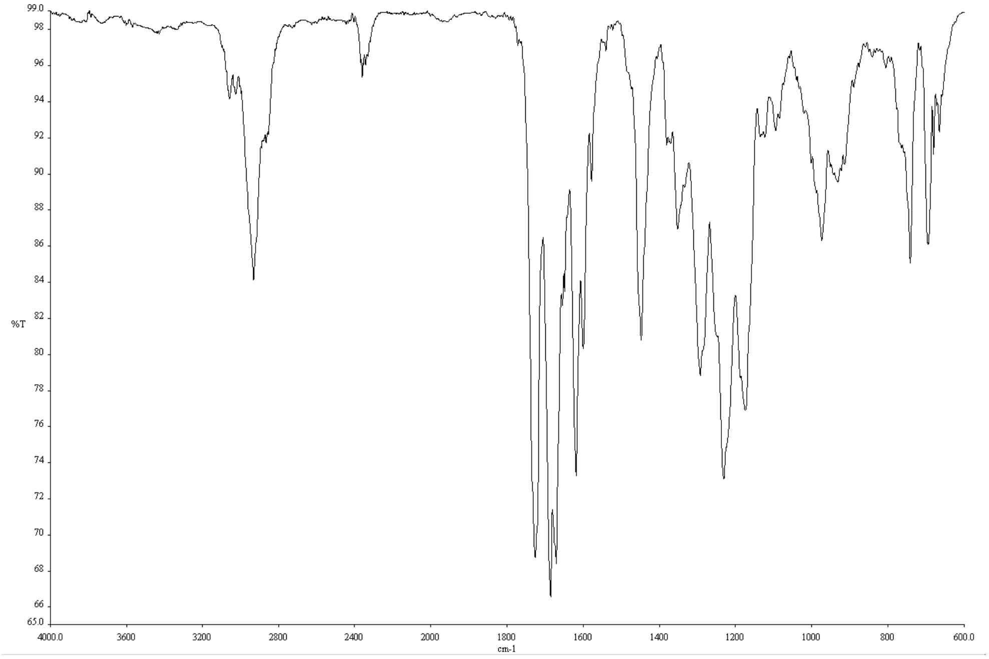

Infrared spectrum (Thin Film, NaCl) of compound **7h**.

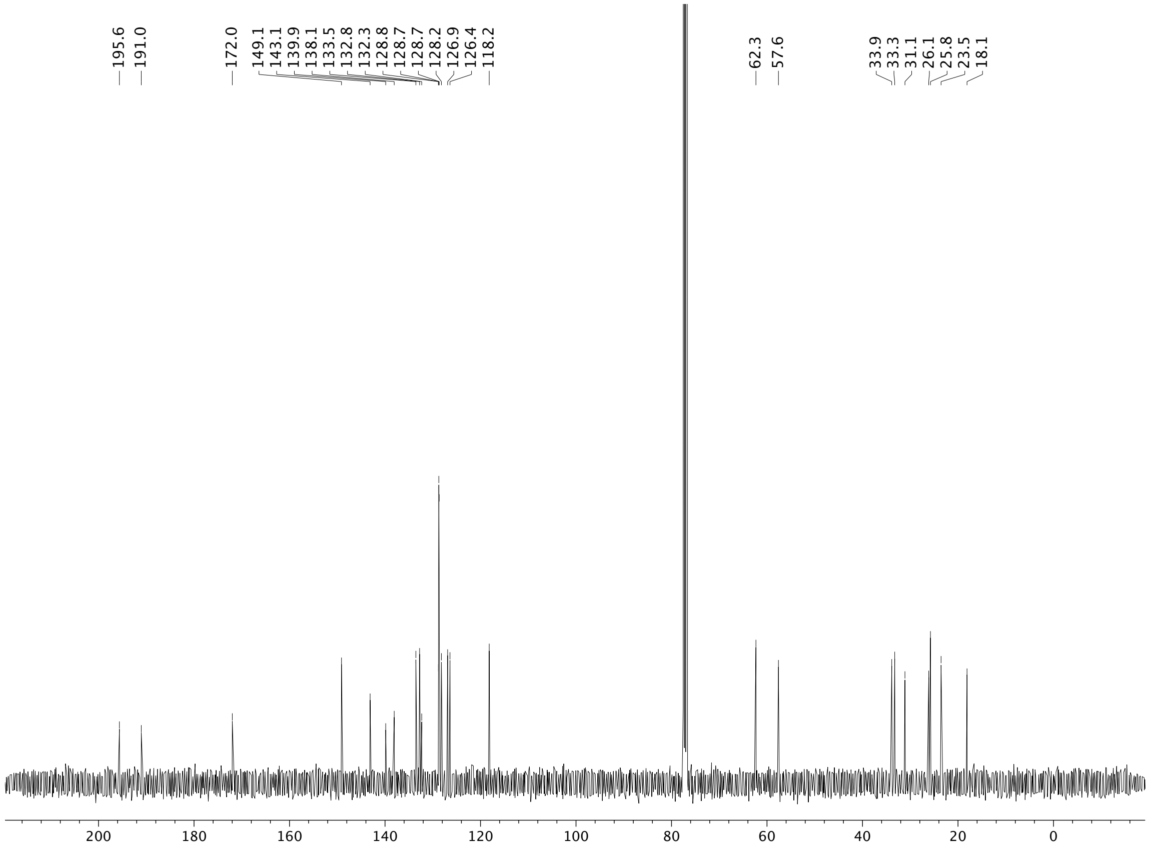

^{13}C NMR (100 MHz, $CDCl_3$) of compound **7h**.

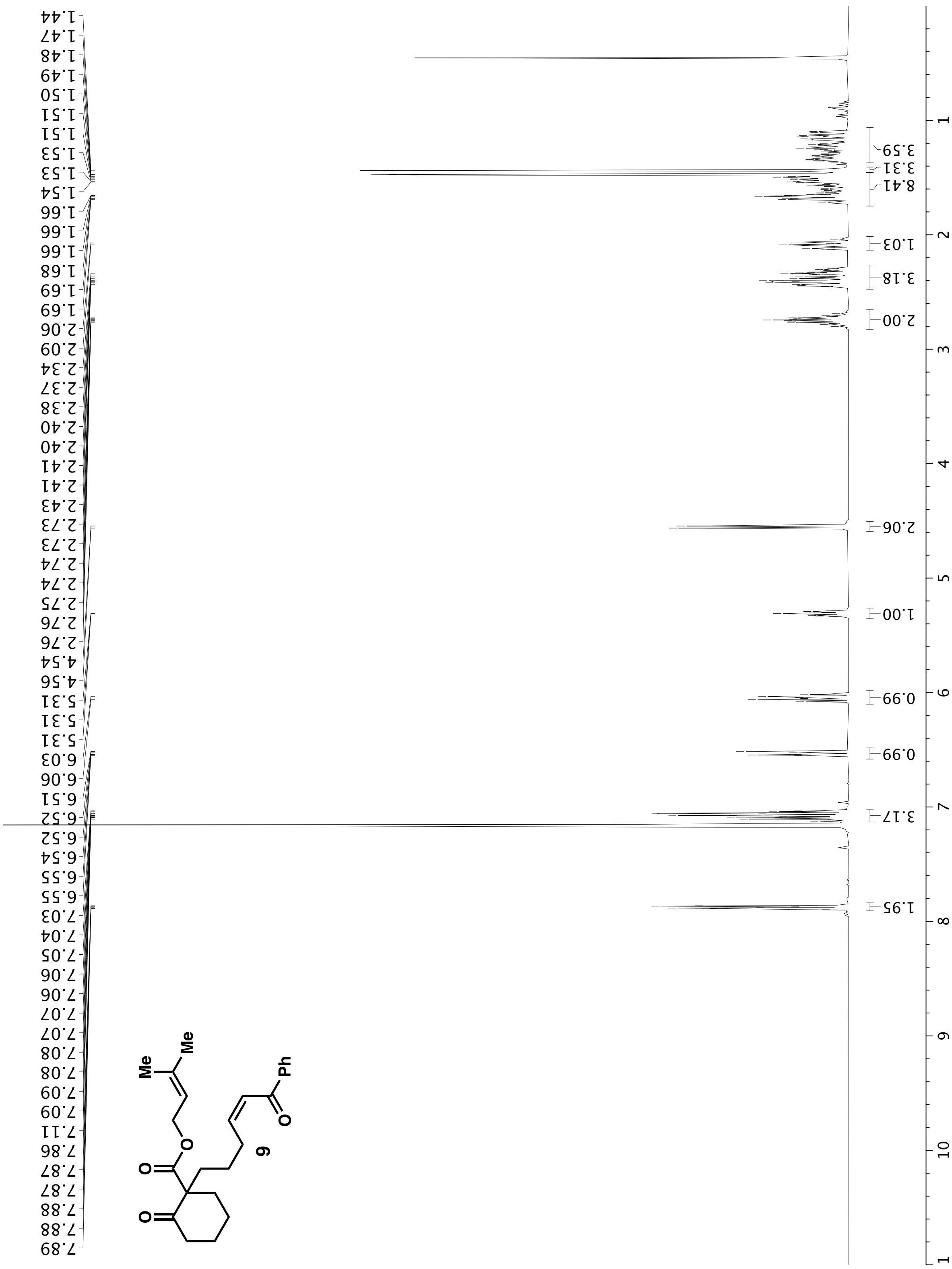

^{1}H NMR (400 MHz, C_6D_6) of compound **9**.

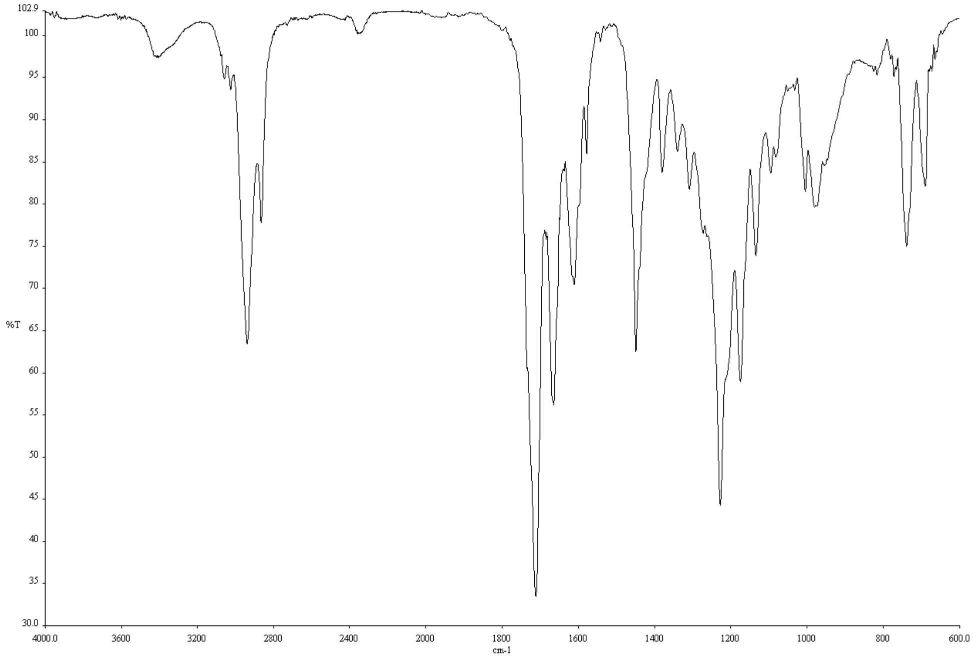

Infrared spectrum (Thin Film, NaCl) of compound **9**.

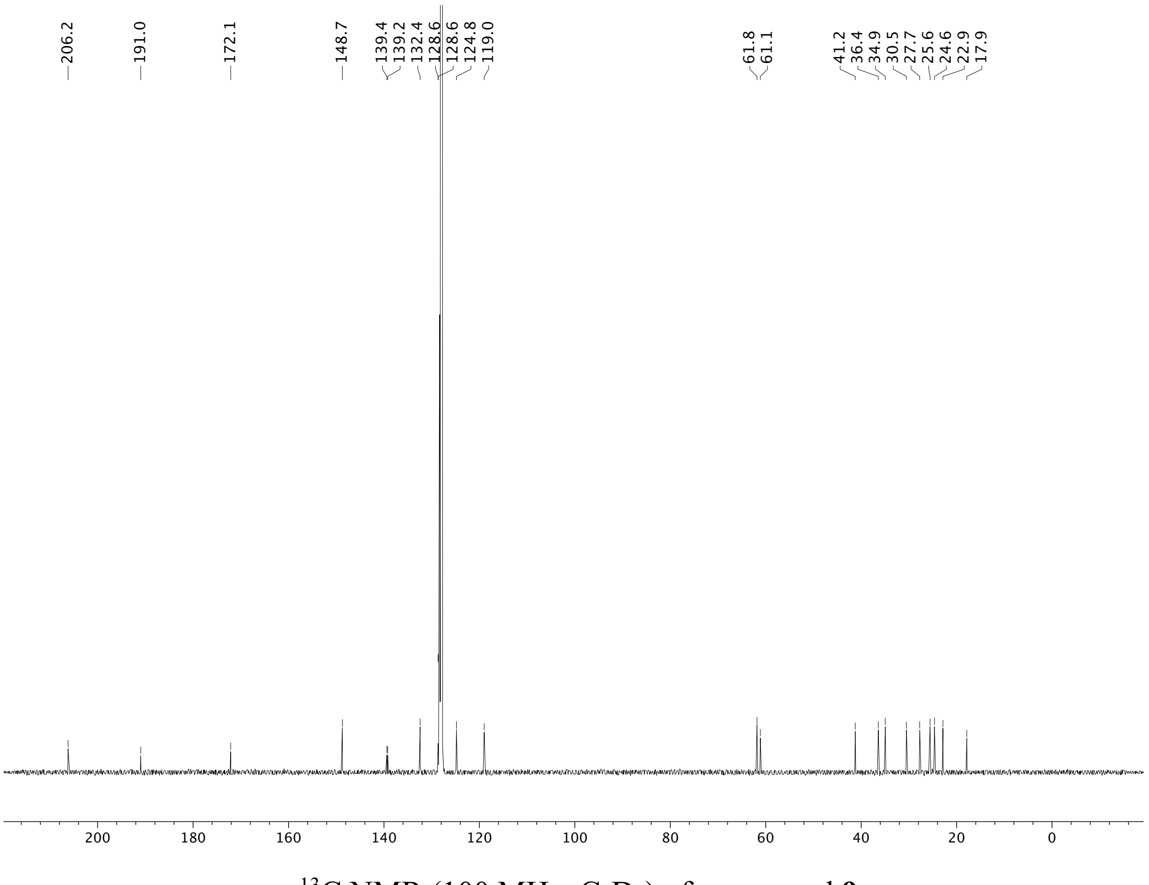

^{13}C NMR (100 MHz, C_6D_6) of compound **9**.

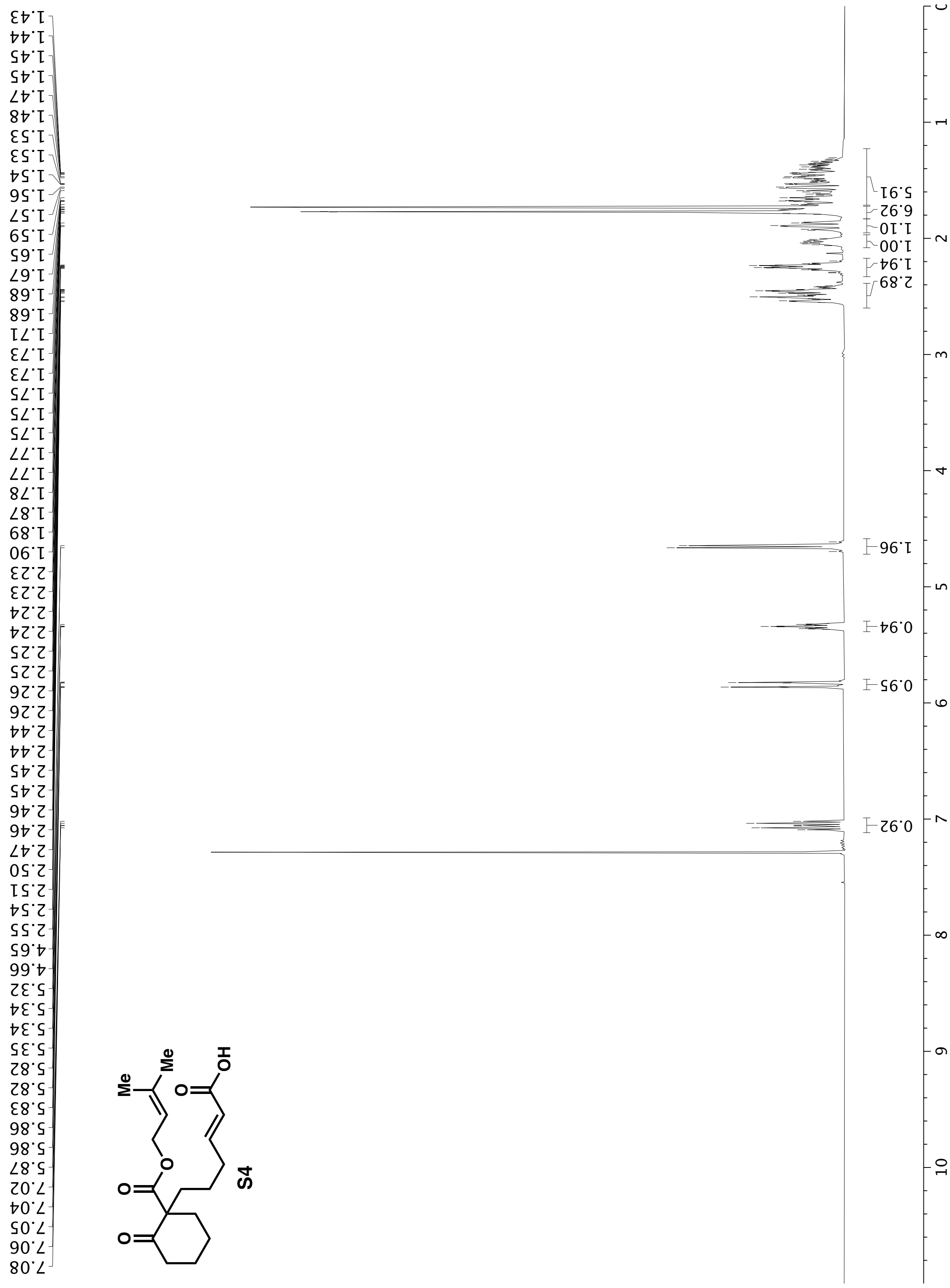

^{1}H NMR (400 MHz, $CDCl_3$) of compound **S4**.

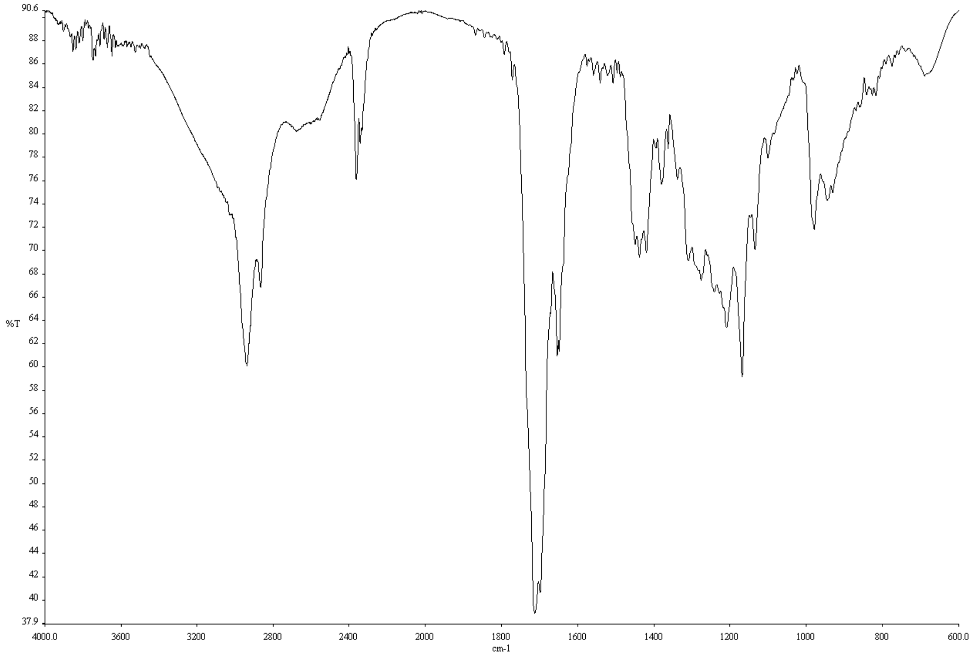

Infrared spectrum (Thin Film, NaCl) of compound **S4**.

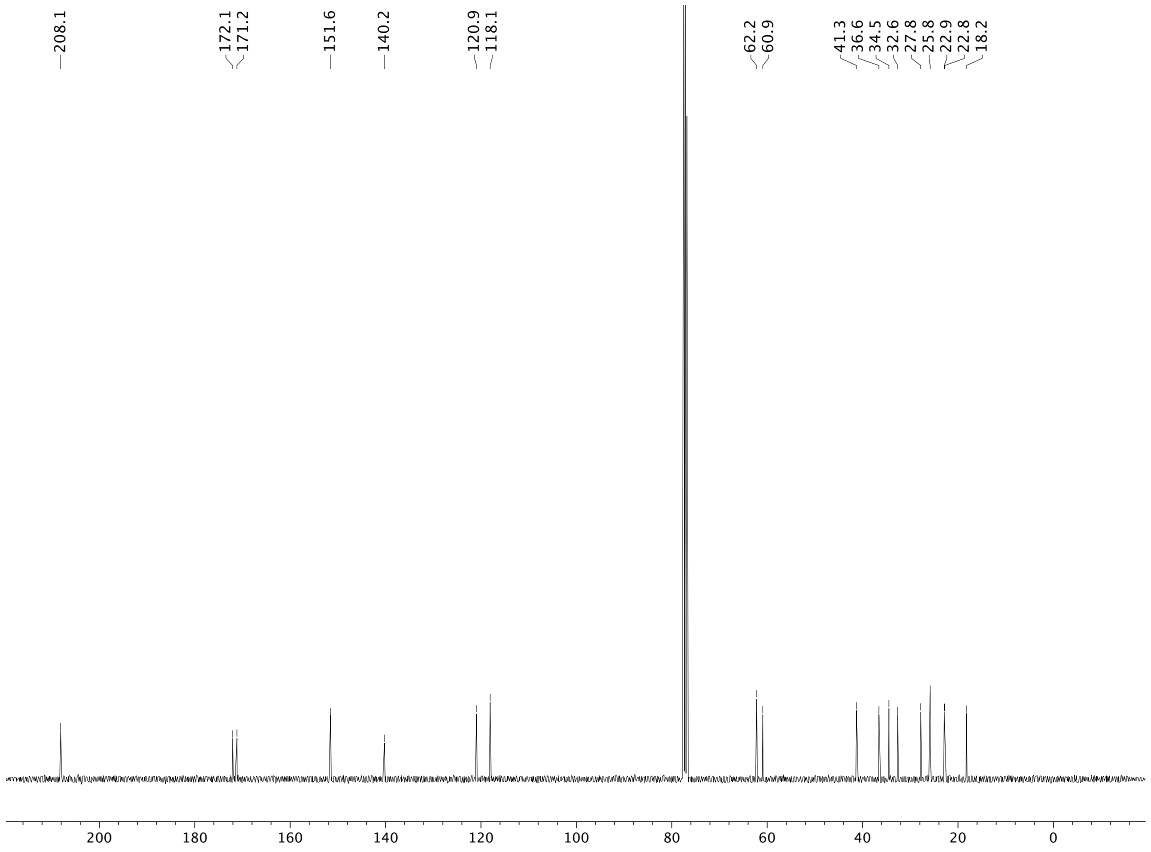

^{13}C NMR (100 MHz, $CDCl_3$) of compound **S4**.

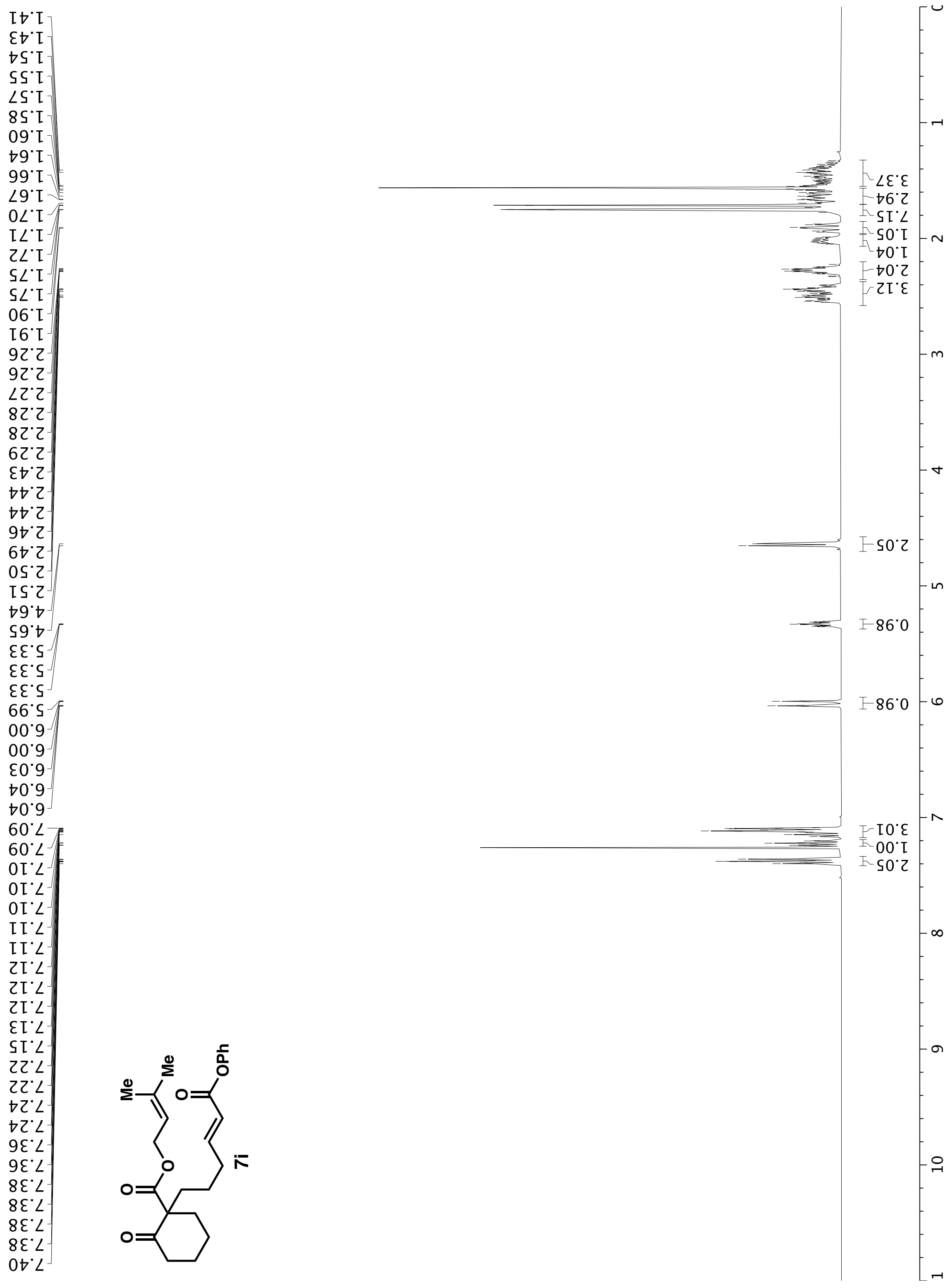

^{1}H NMR (400 MHz, $CDCl_3$) of compound **7i**.

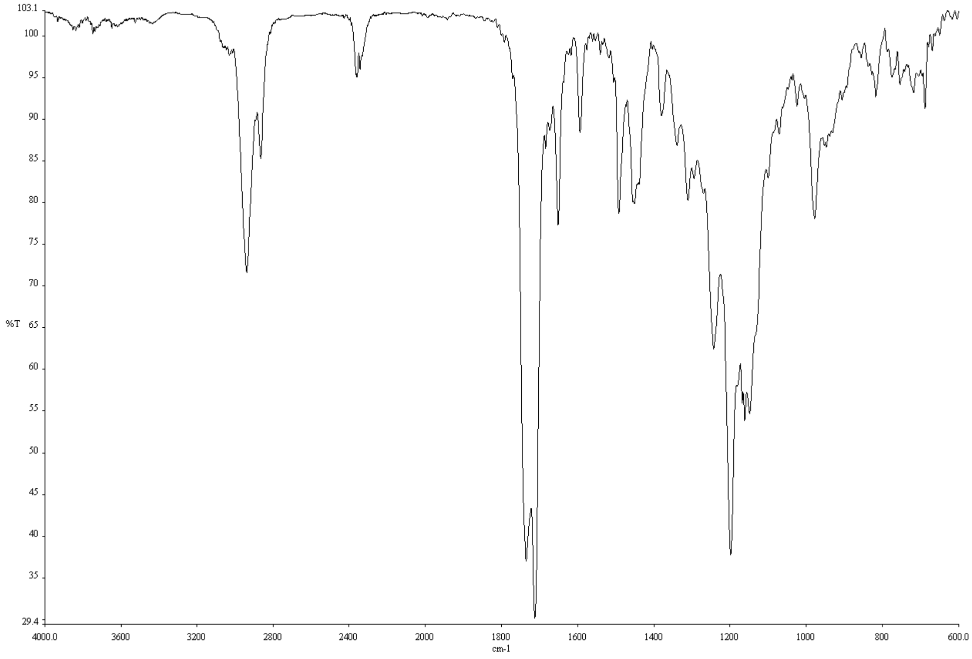

Infrared spectrum (Thin Film, NaCl) of compound **7i**.

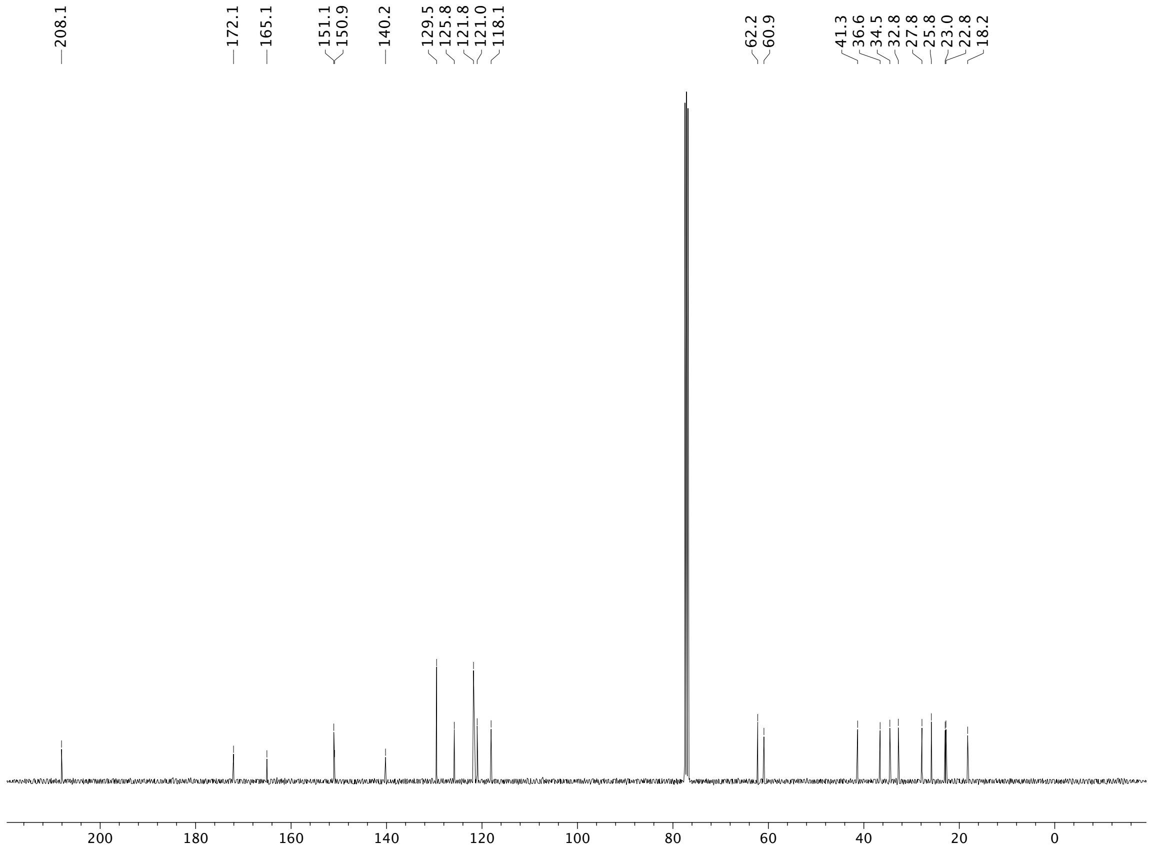

^{13}C NMR (100 MHz, $CDCl_3$) of compound **7i**.

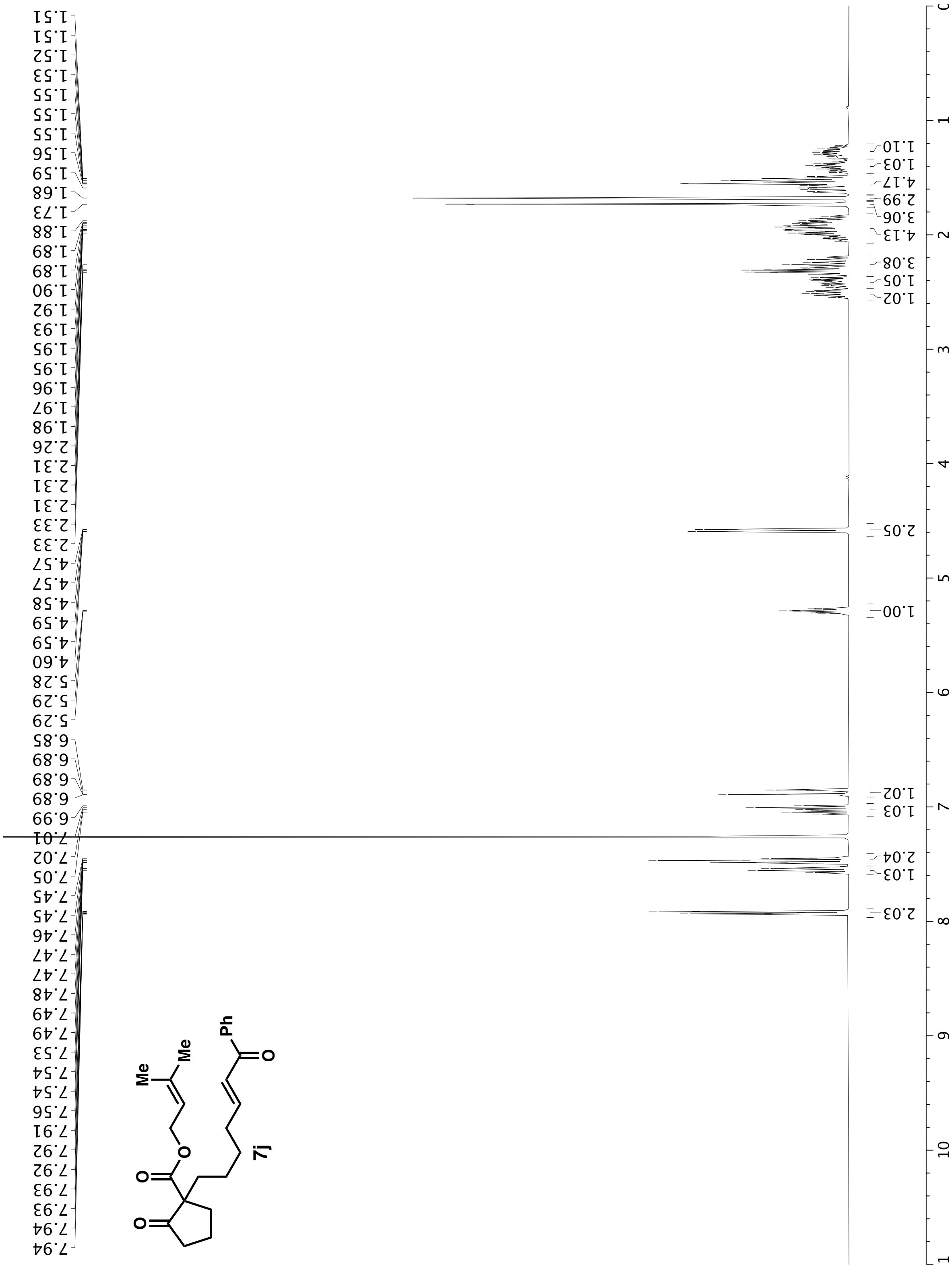

^{1}H NMR (400 MHz, $CDCl_3$) of compound **7j**.

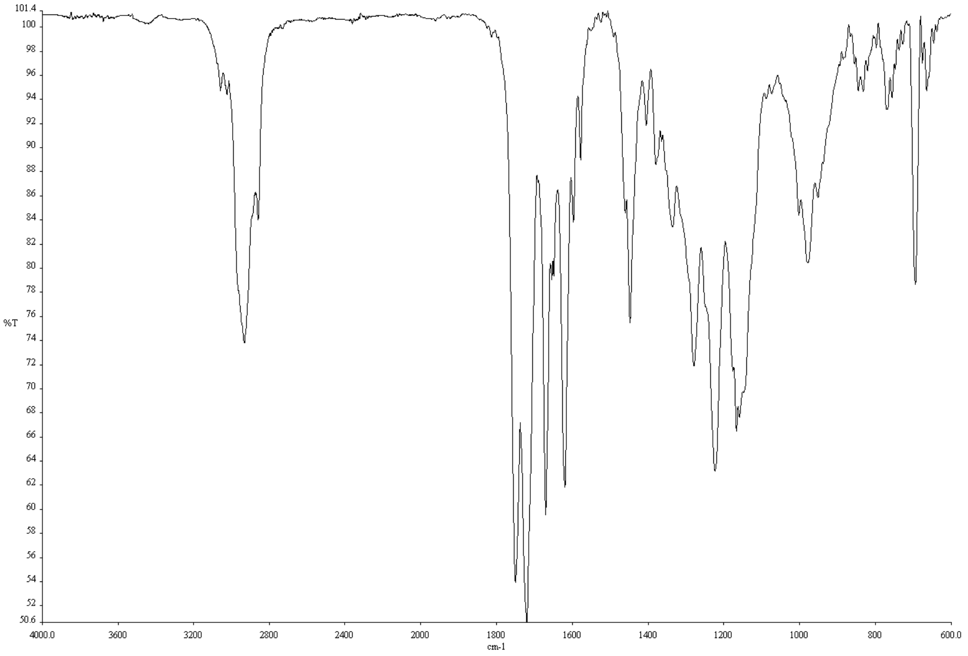

Infrared spectrum (Thin Film, NaCl) of compound **7j**.

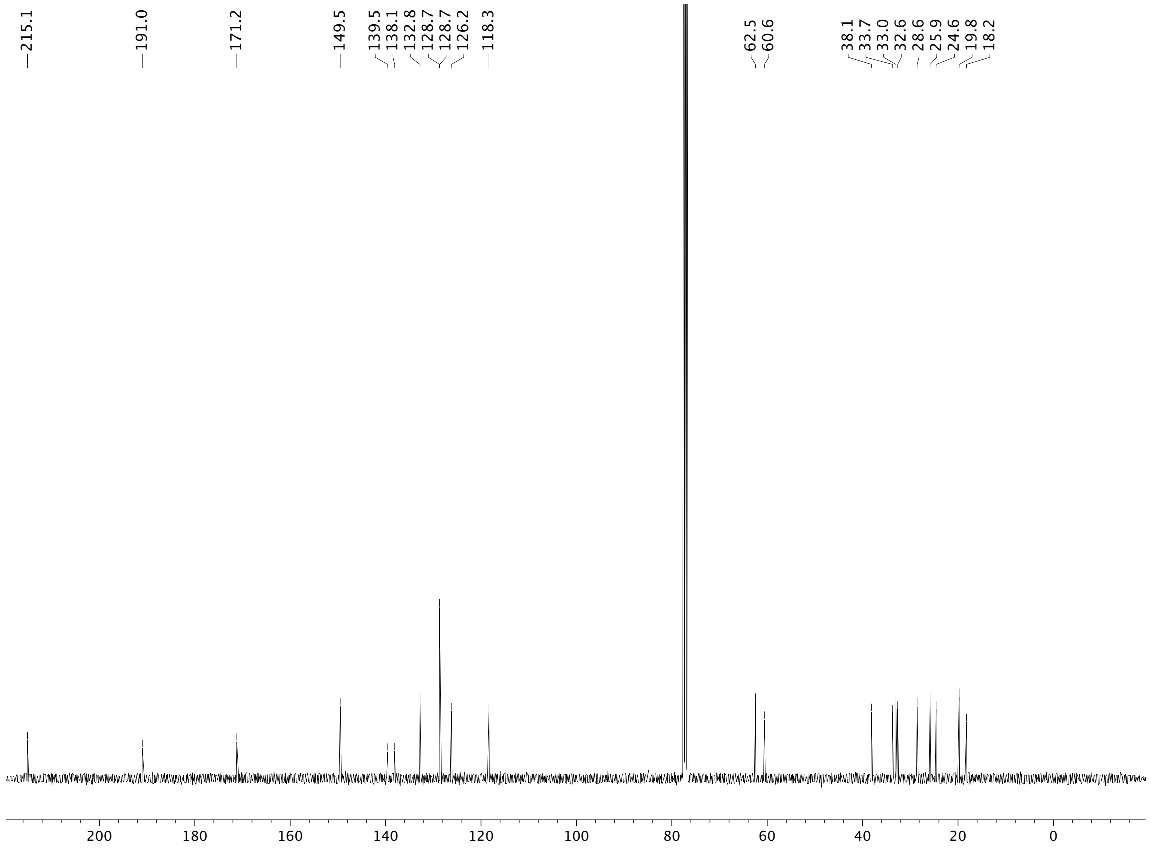

^{13}C NMR (100 MHz, $CDCl_3$) of compound **7j**.

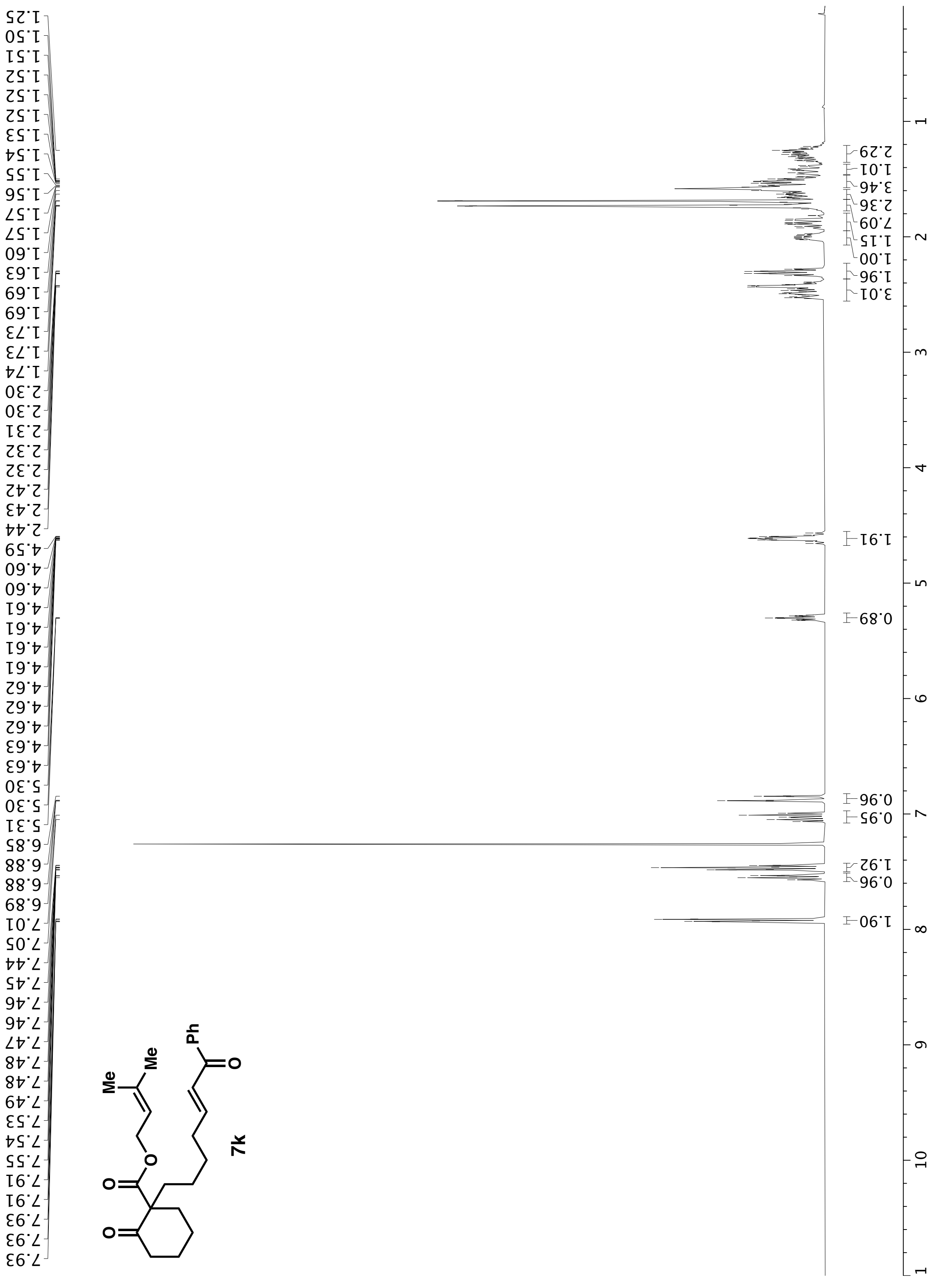

^{1}H NMR (400 MHz, $CDCl_3$) of compound **7k**.

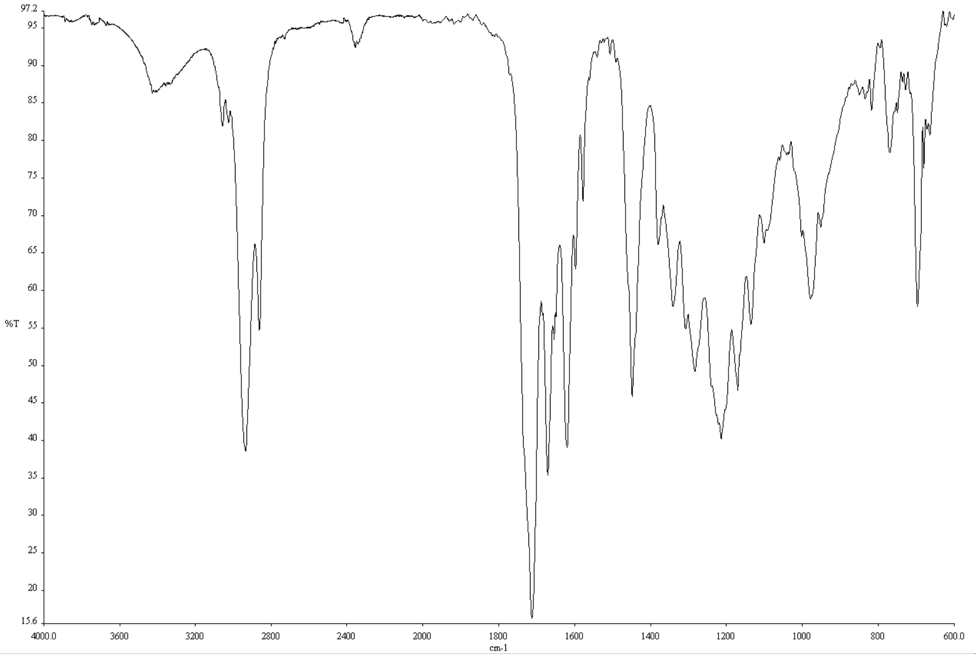

Infrared spectrum (Thin Film, NaCl) of compound **7k**.

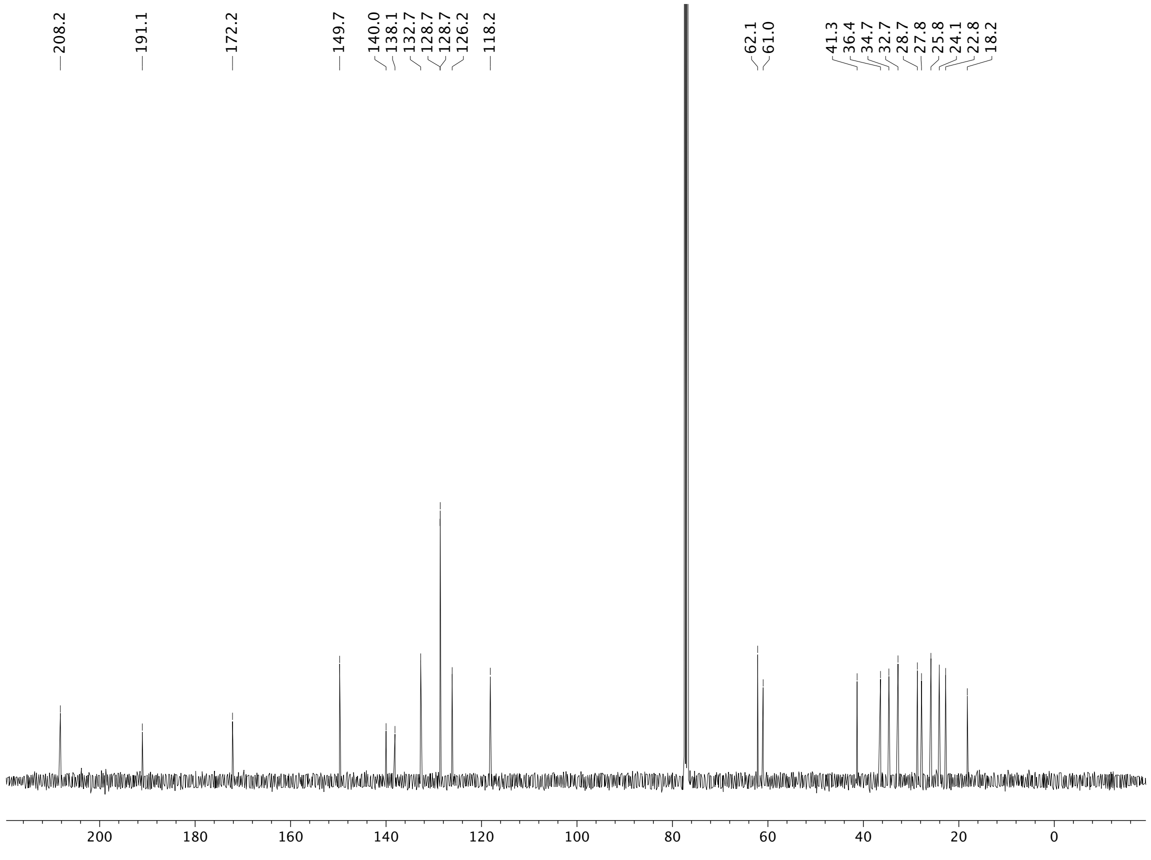

^{13}C NMR (100 MHz, $CDCl_3$) of compound **7k**.

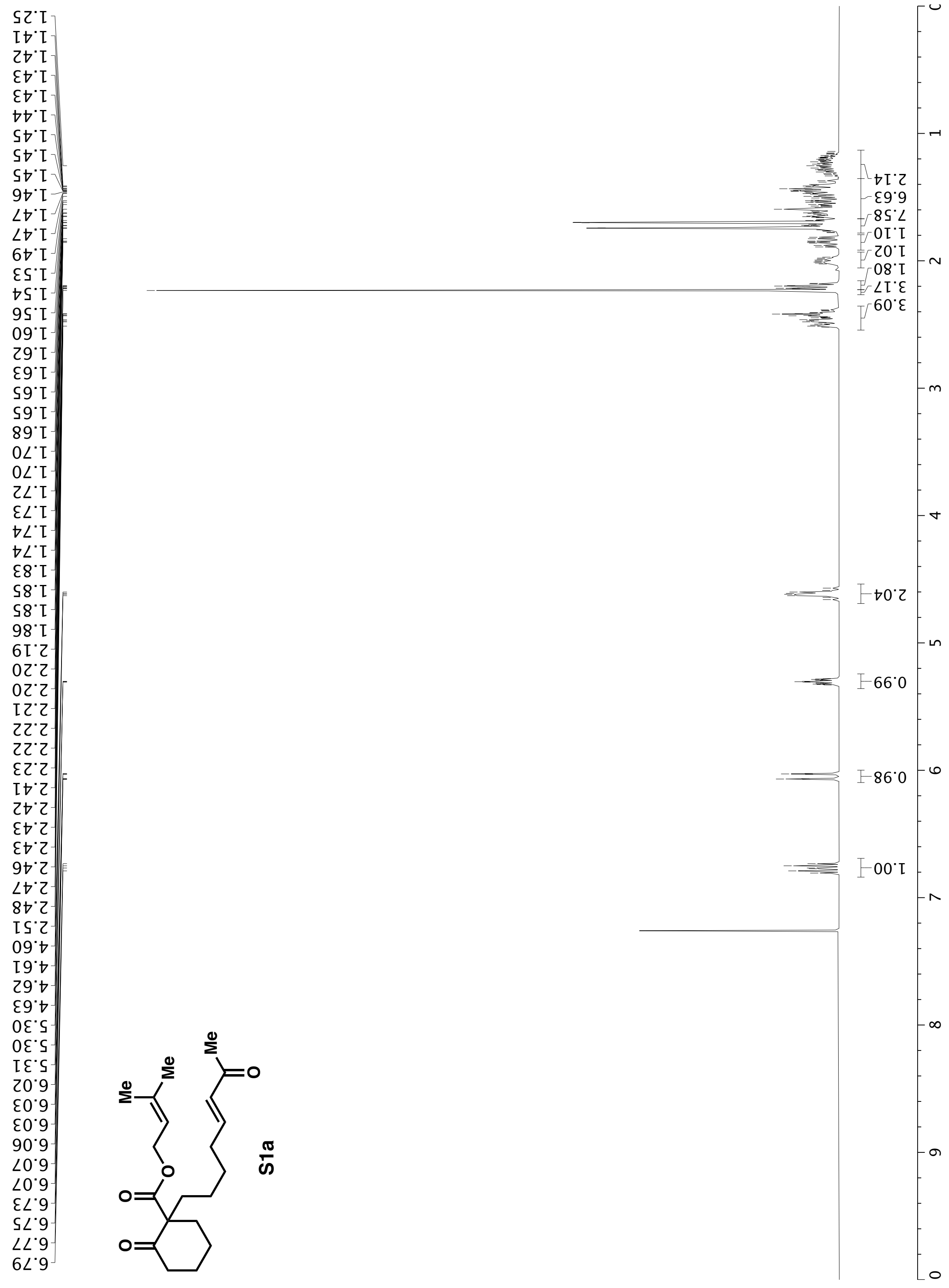

^{1}H NMR (400 MHz, $CDCl_3$) of compound **S1a**.

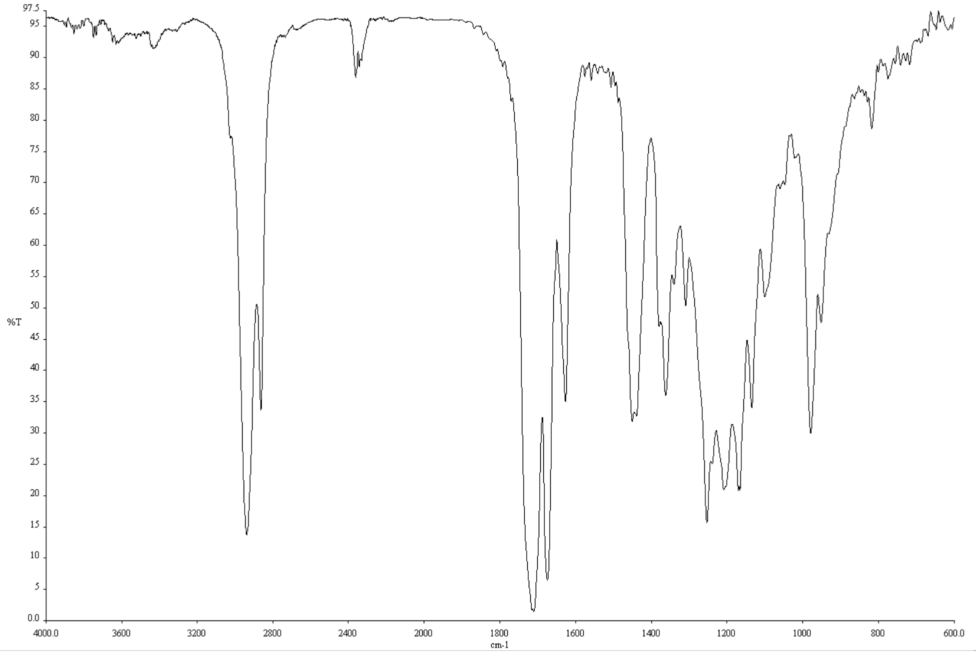

Infrared spectrum (Thin Film, NaCl) of compound **S1a**.

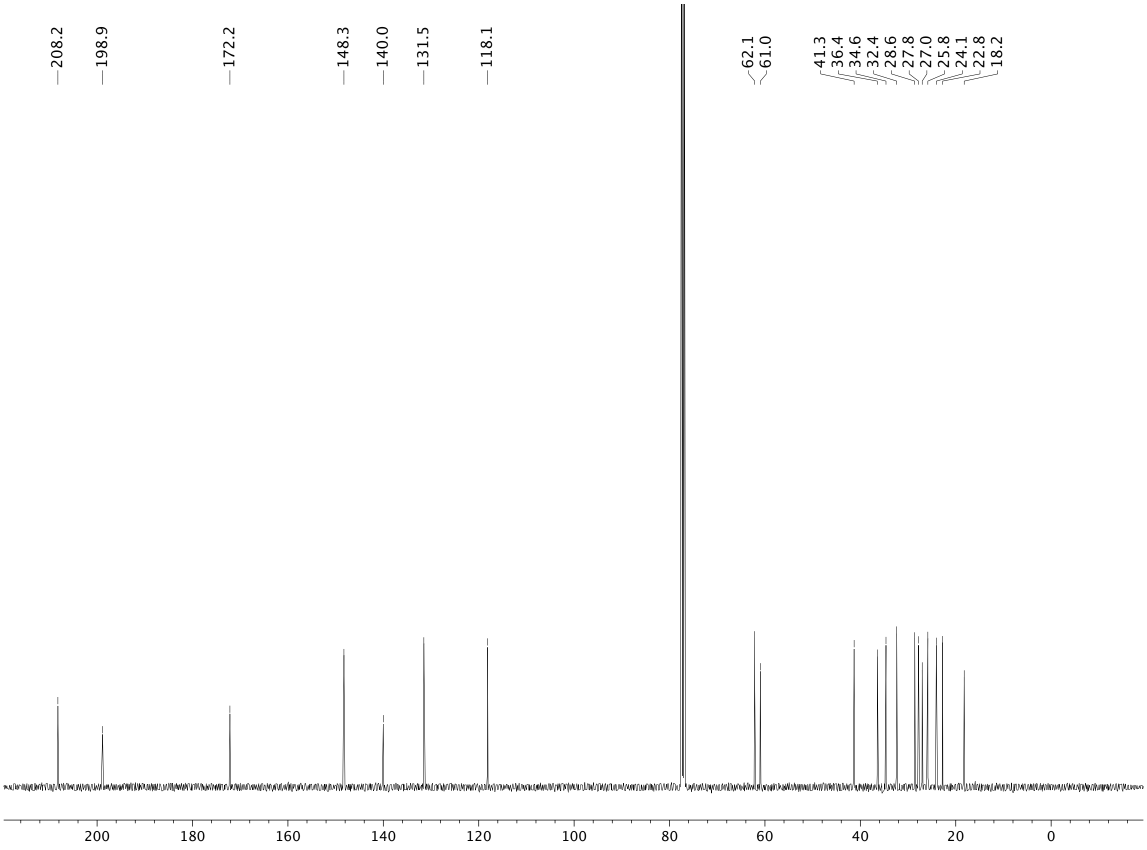

^{13}C NMR (100 MHz, $CDCl_3$) of compound **S1a**.

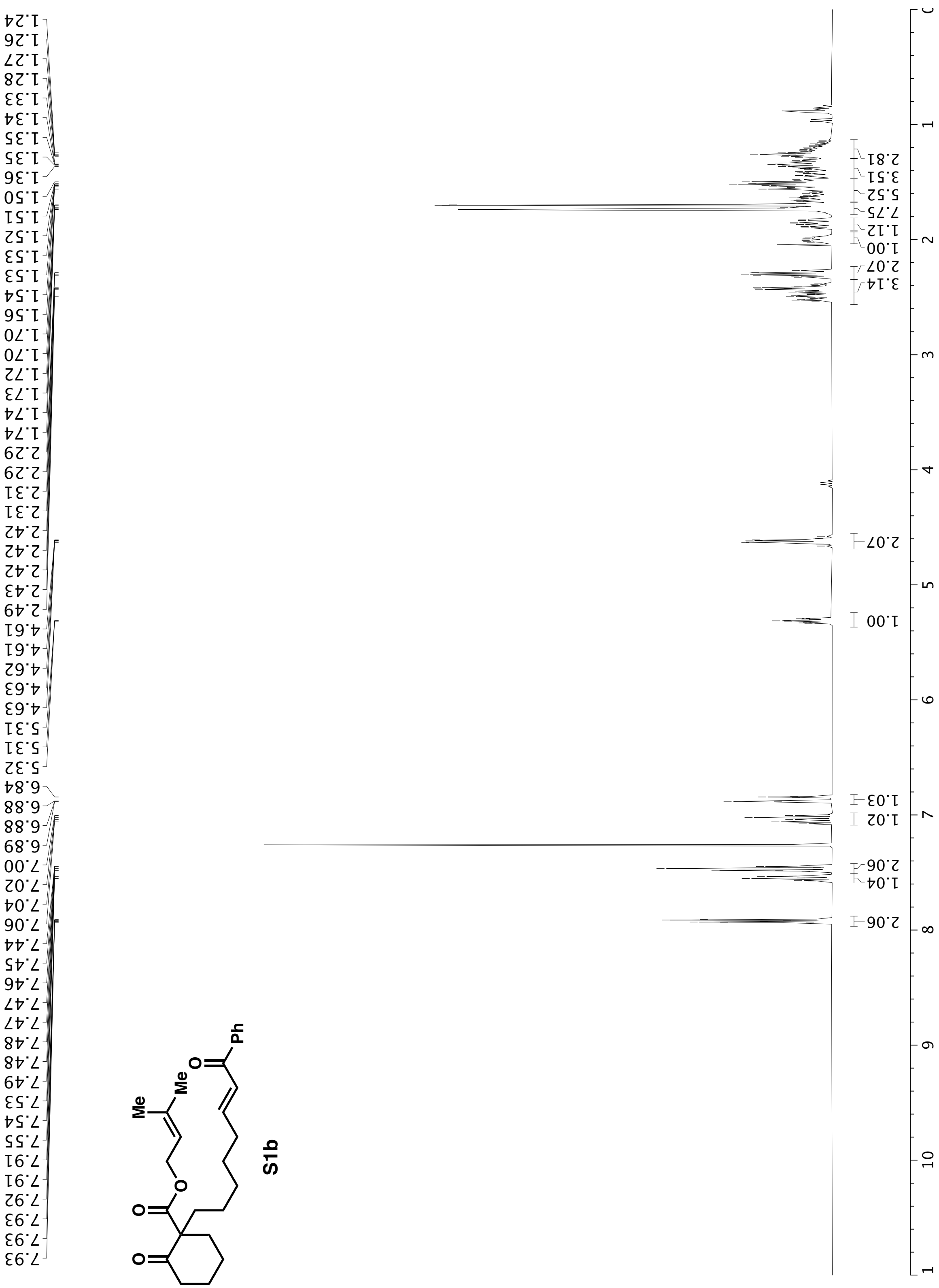

^{1}H NMR (400 MHz, $CDCl_3$) of compound **S1b**.

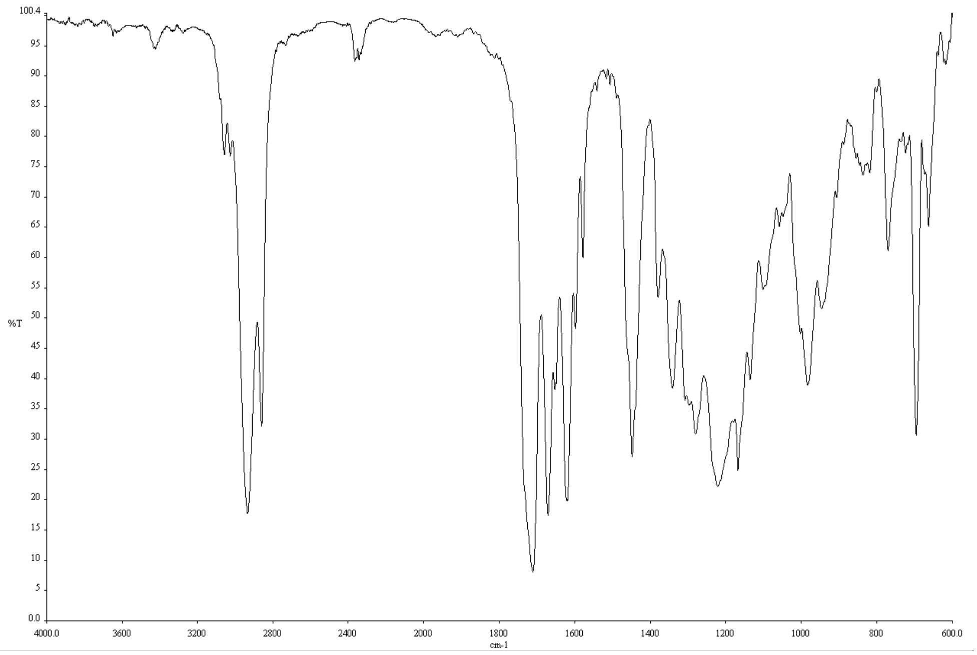

Infrared spectrum (Thin Film, NaCl) of compound **S1b**.

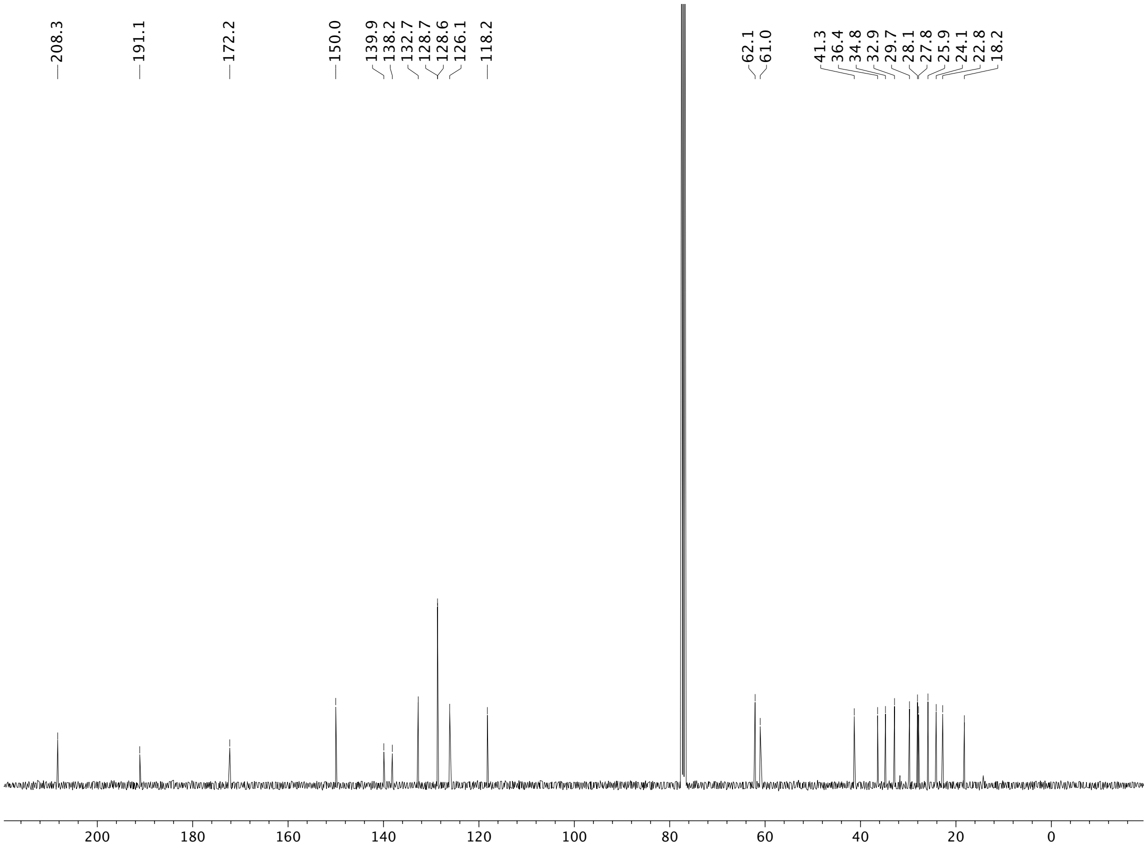

^{13}C NMR (100 MHz, $CDCl_3$) of compound **S1b**.

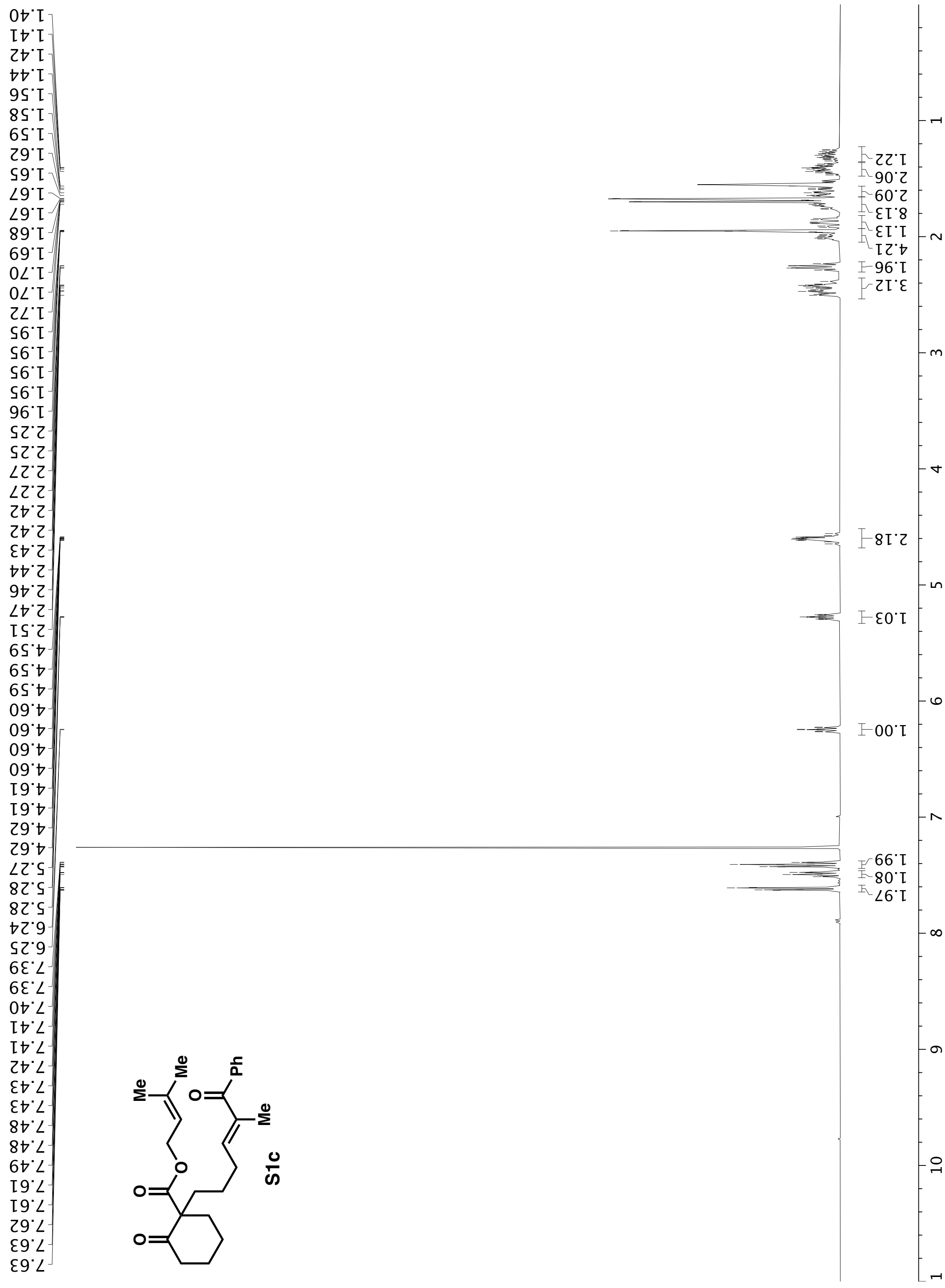

^{1}H NMR (400 MHz, $CDCl_3$) of compound **S1c**.

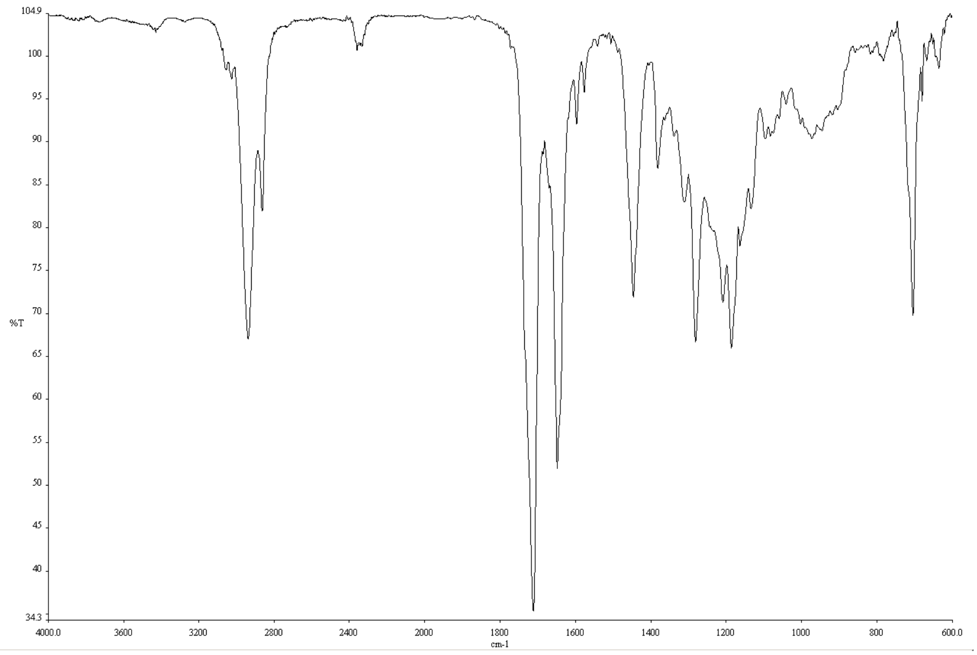

Infrared spectrum (Thin Film, NaCl) of compound **S1c**.

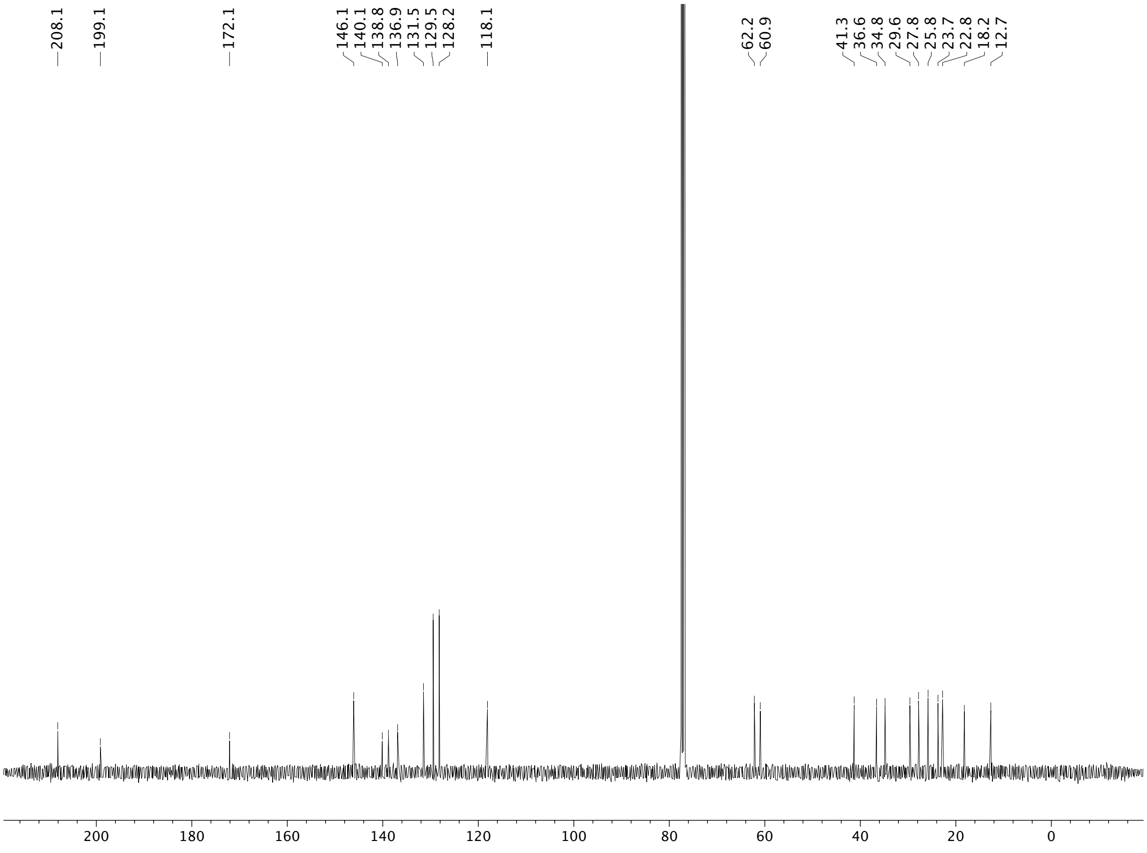

^{13}C NMR (100 MHz, $CDCl_3$) of compound **S1c**.

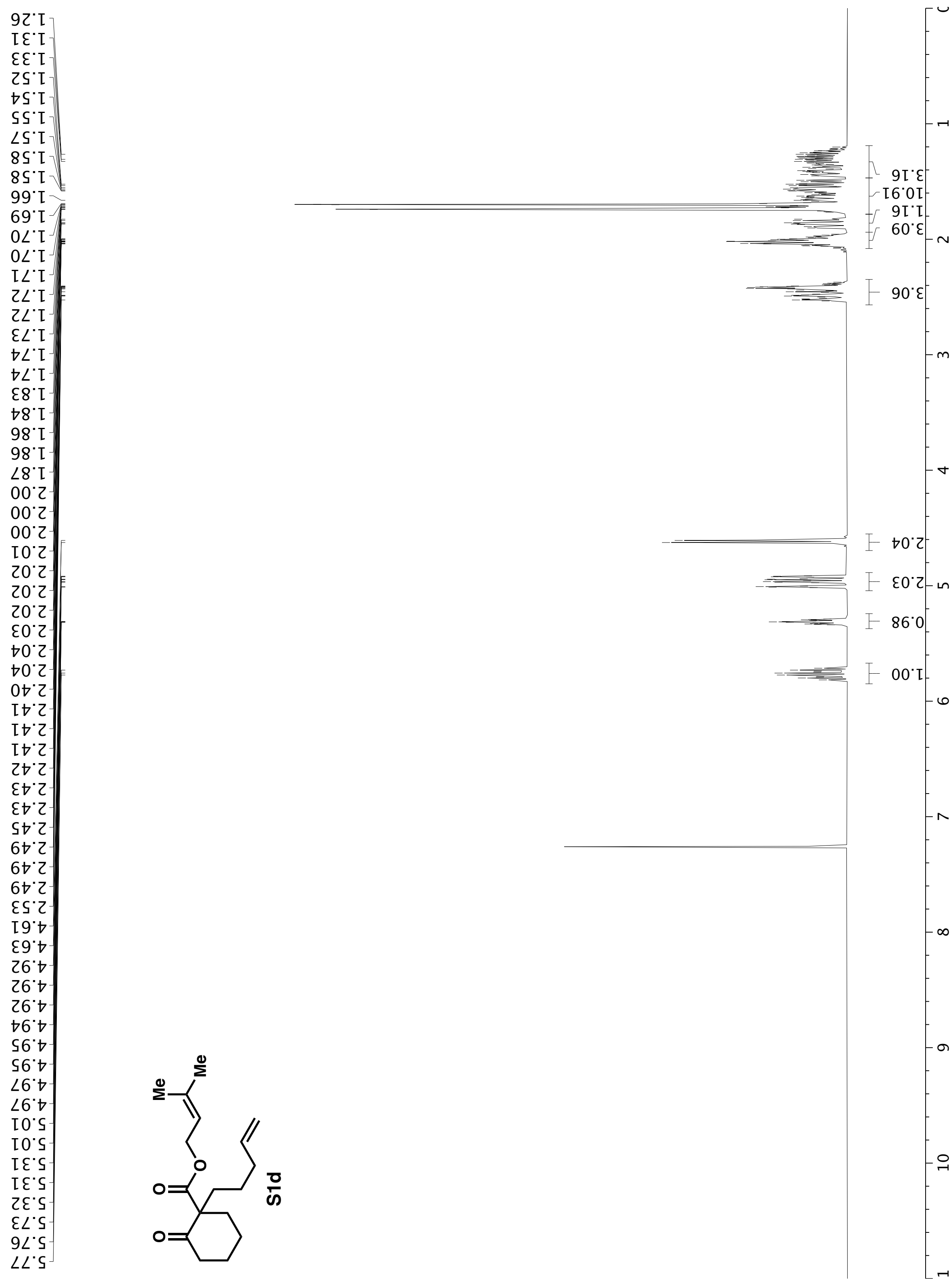

¹H NMR (400 MHz, $CDCl_3$) of compound **S1d**.

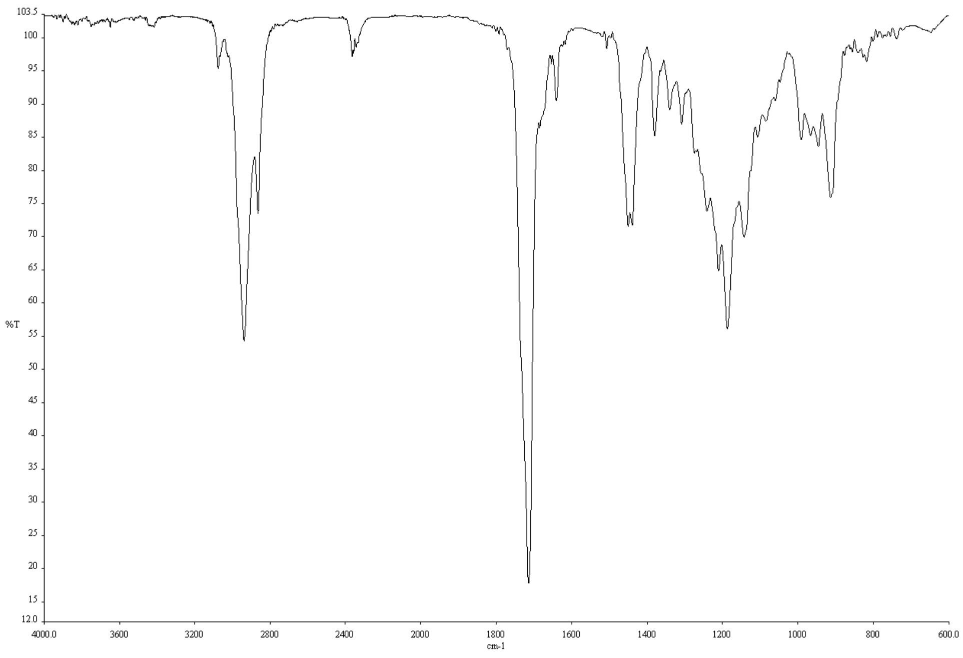

Infrared spectrum (Thin Film, NaCl) of compound **S1d**.

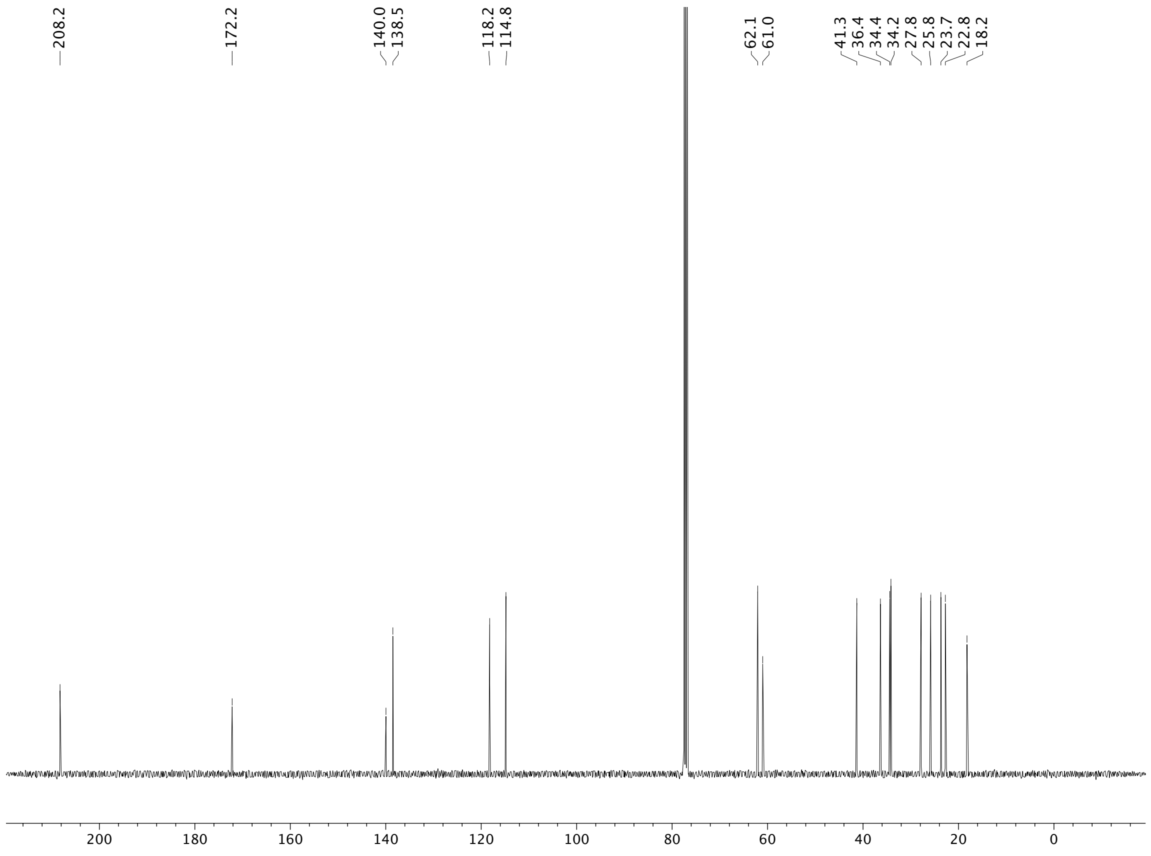

^{13}C NMR (100 MHz, $CDCl_3$) of compound **S1d**.

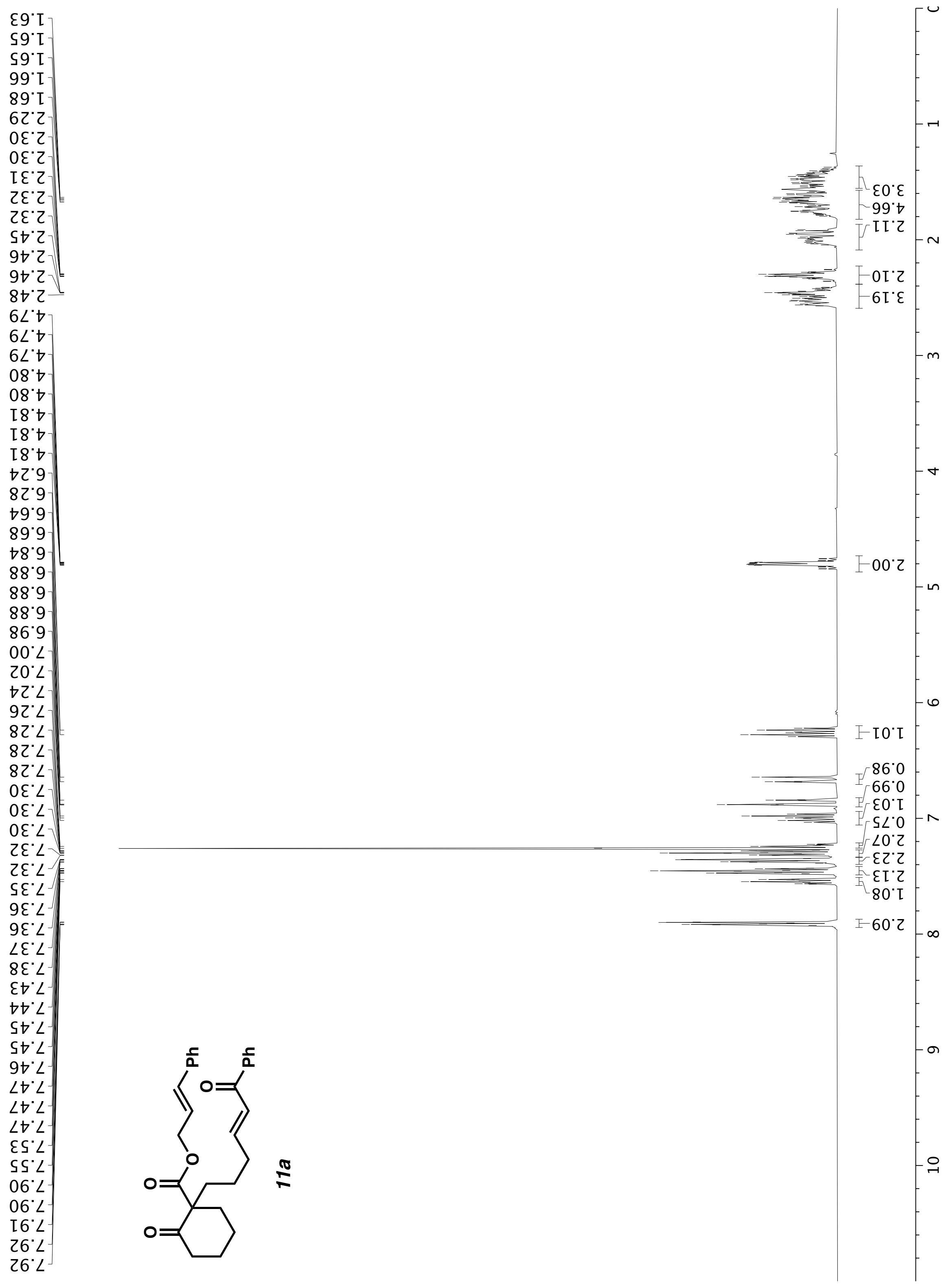

^{1}H NMR (400 MHz, $CDCl_3$) of compound **11a**.

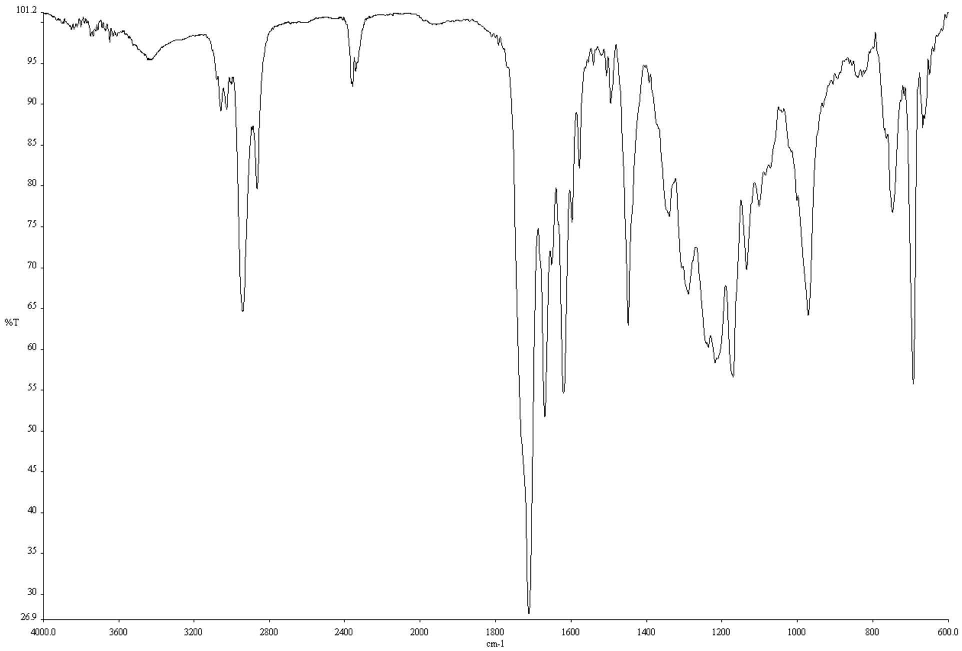

Infrared spectrum (Thin Film, NaCl) of compound **11a**.

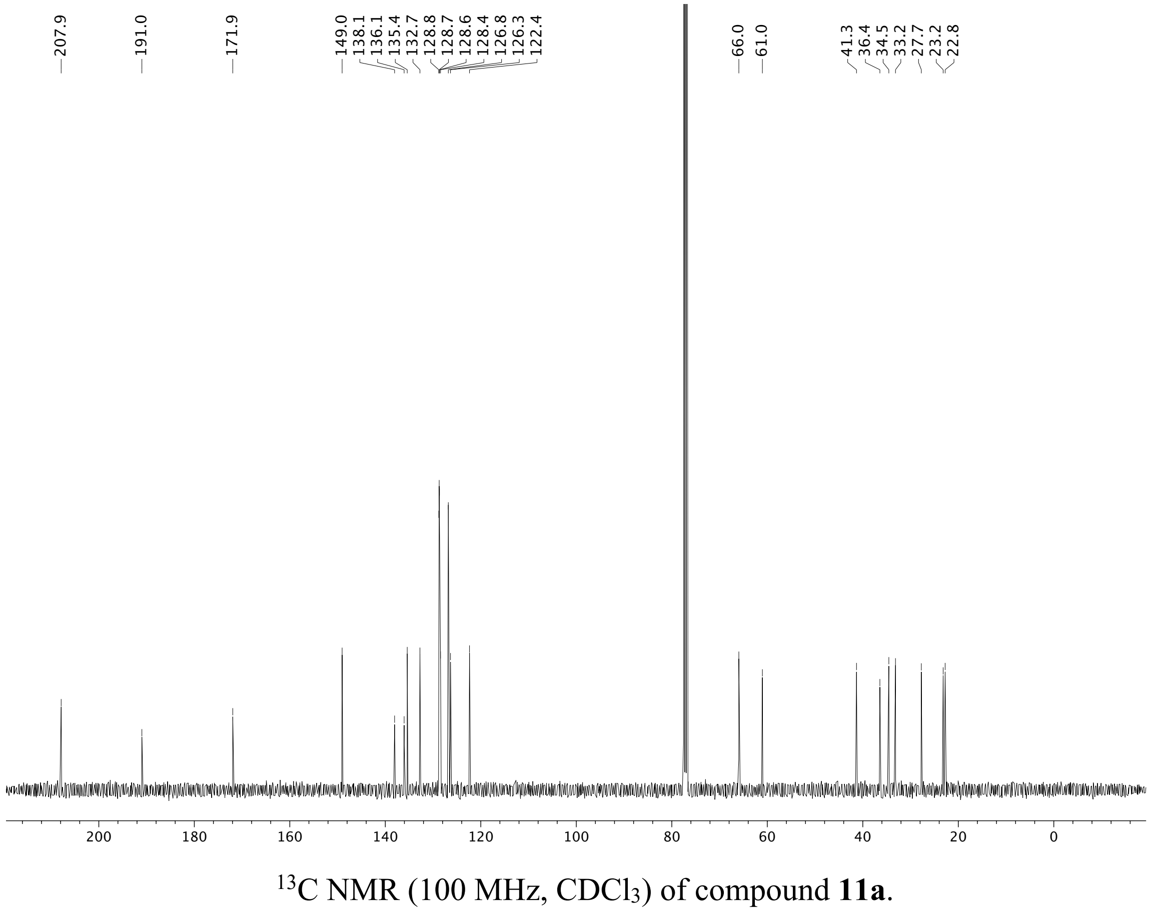

^{13}C NMR (100 MHz, $CDCl_3$) of compound **11a**.

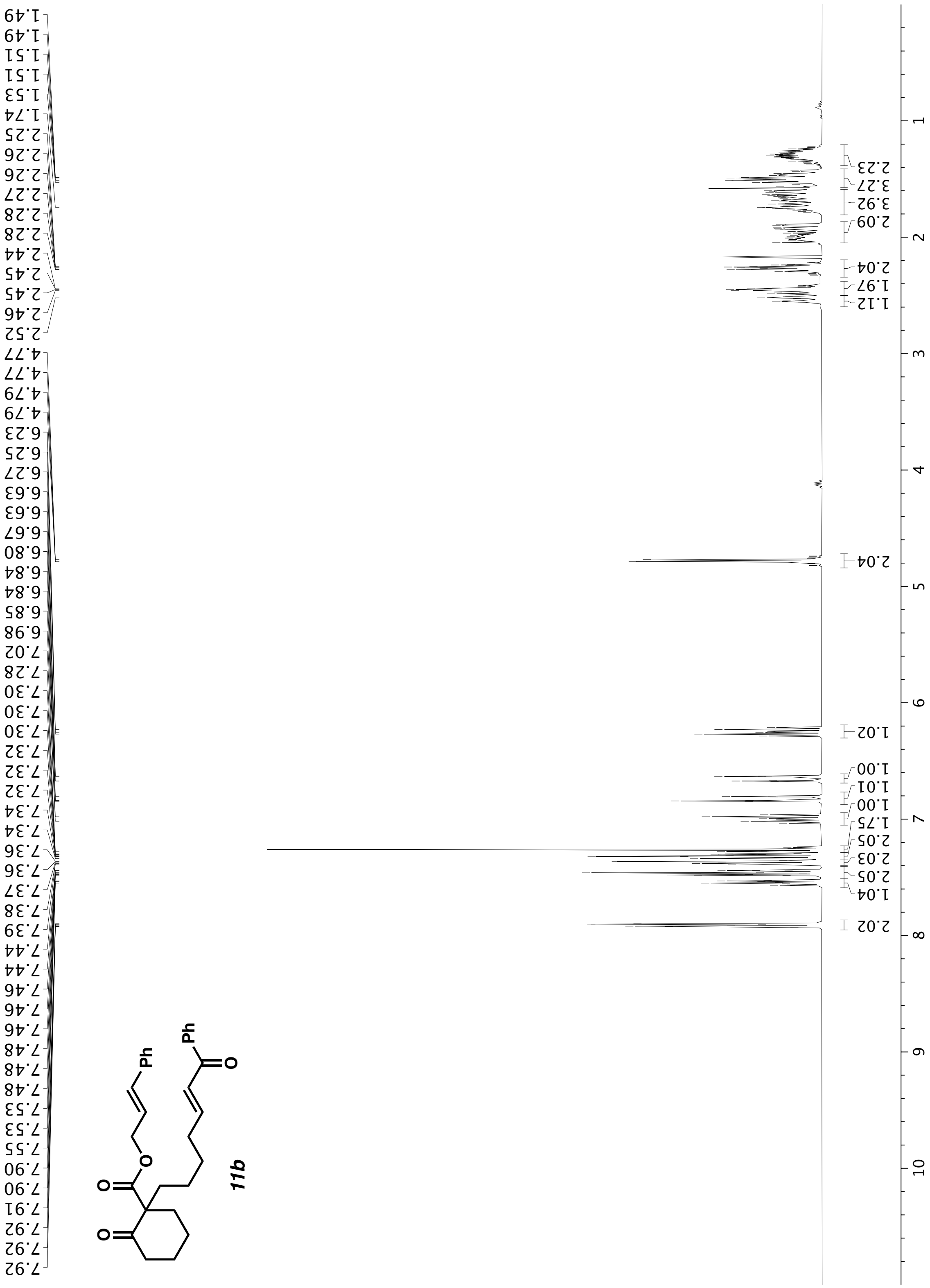

^{1}H NMR (400 MHz, $CDCl_3$) of compound **11b**.

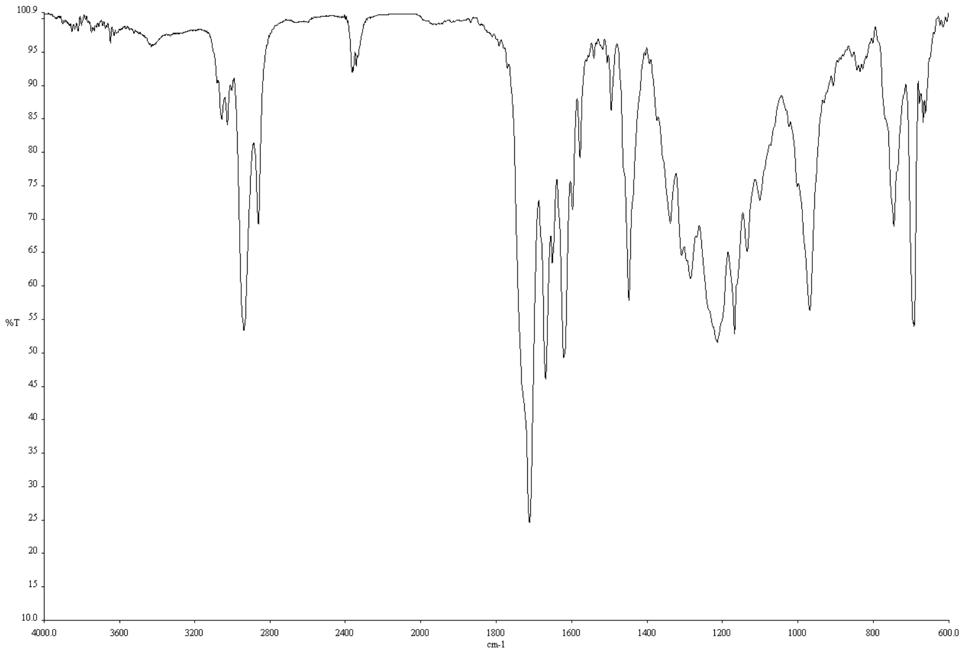

Infrared spectrum (Thin Film, NaCl) of compound **11b**.

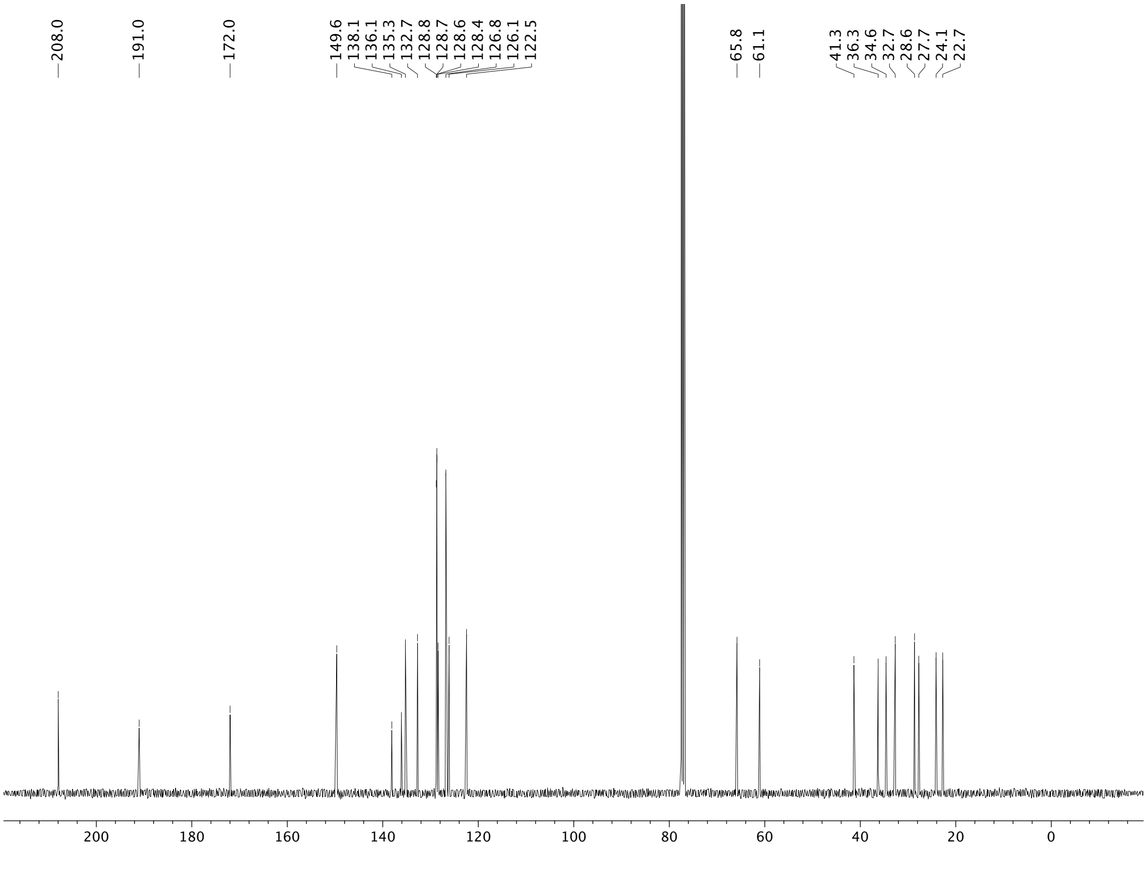

^{13}C NMR (100 MHz, $CDCl_3$) of compound **11b**.

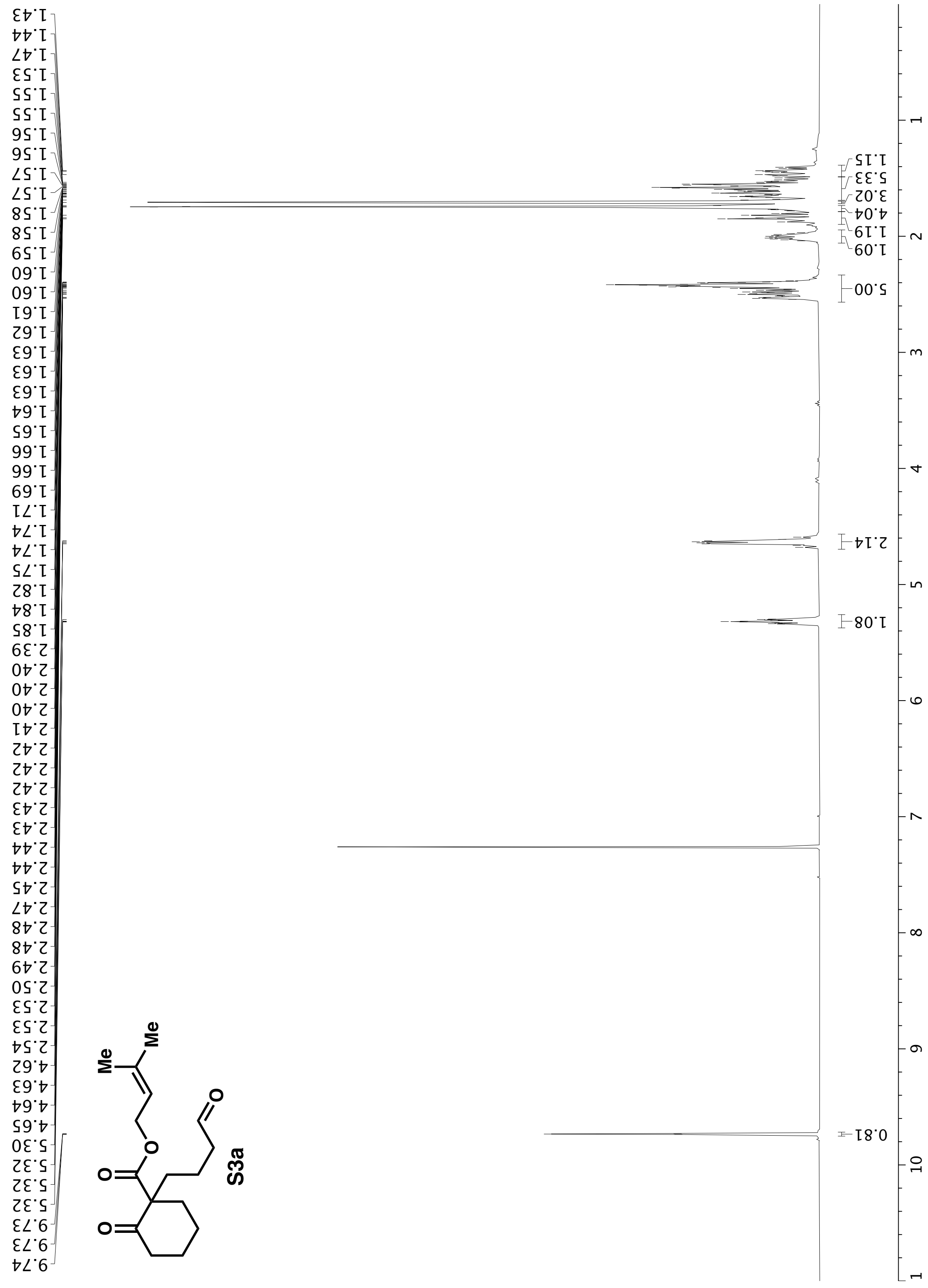

^{1}H NMR (400 MHz, $CDCl_3$) of compound **S3a**.

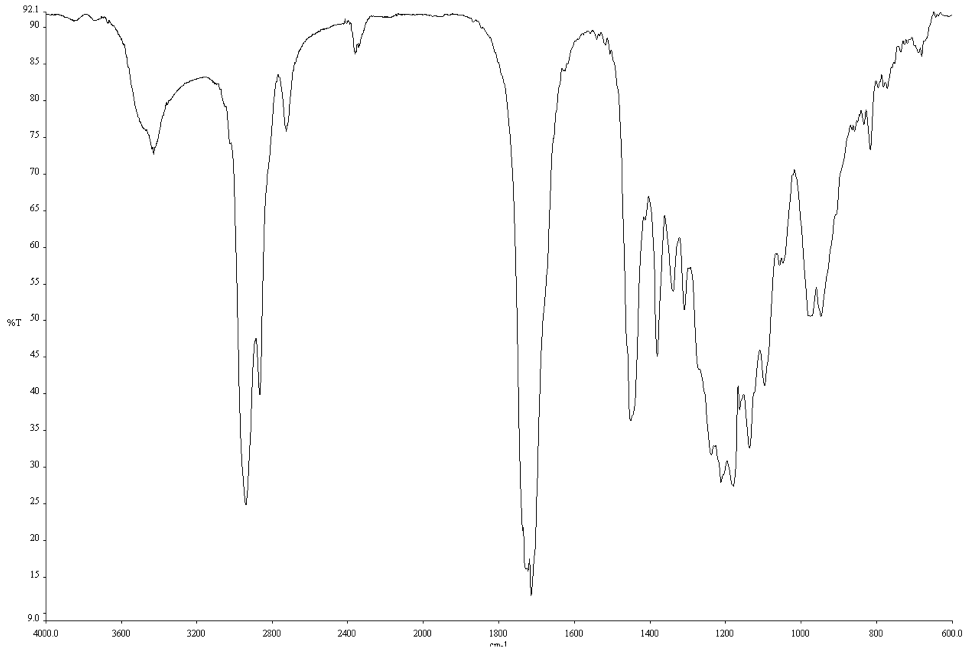

Infrared spectrum (Thin Film, NaCl) of compound **S3a**.

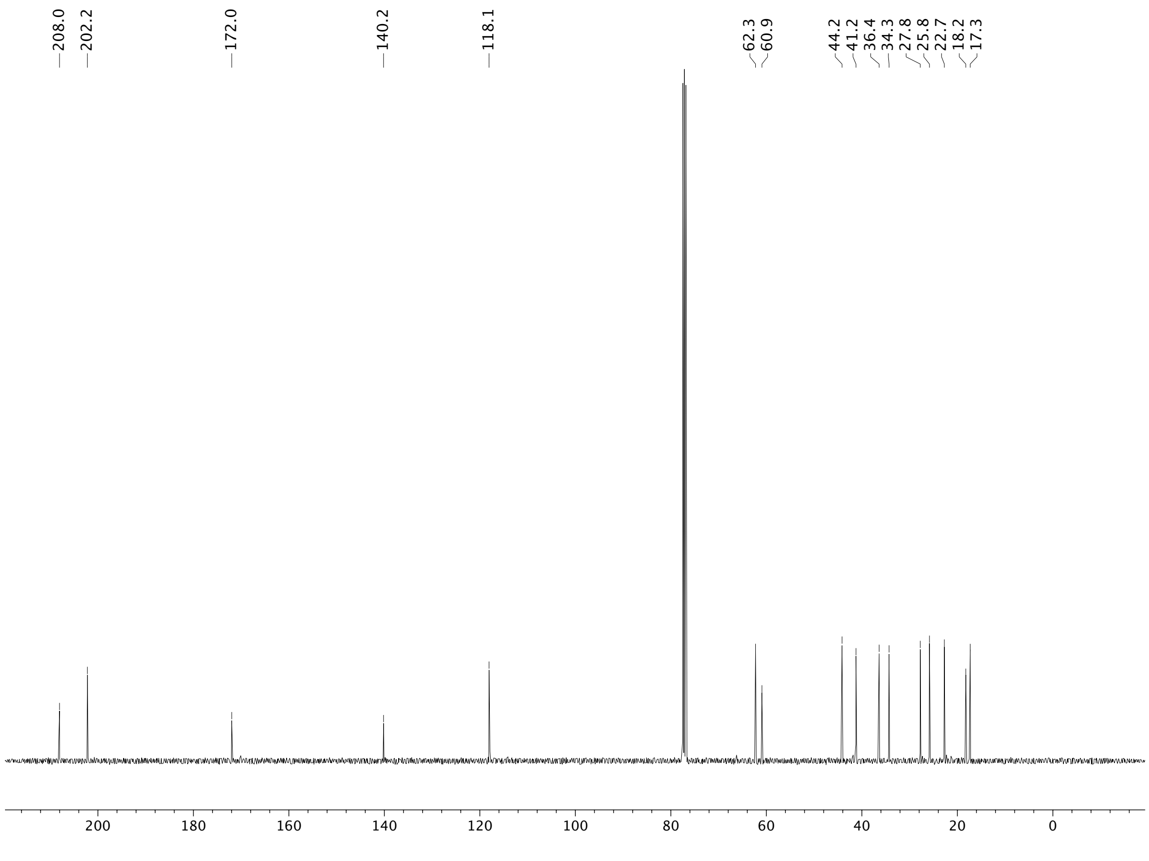

^{13}C NMR (100 MHz, $CDCl_3$) of compound **S3a**.

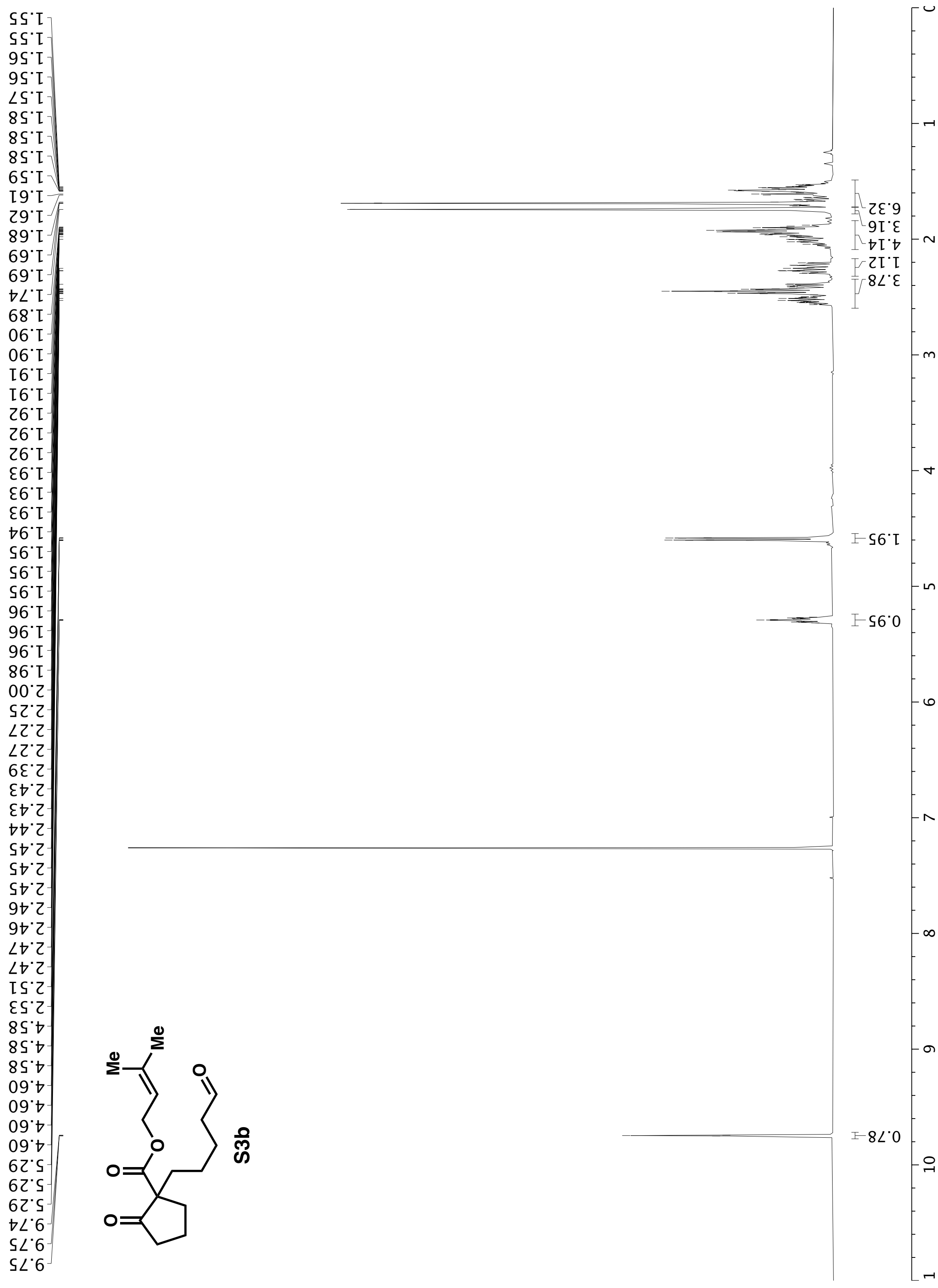

^1H NMR (400 MHz, $CDCl_3$) of compound **S3b**.

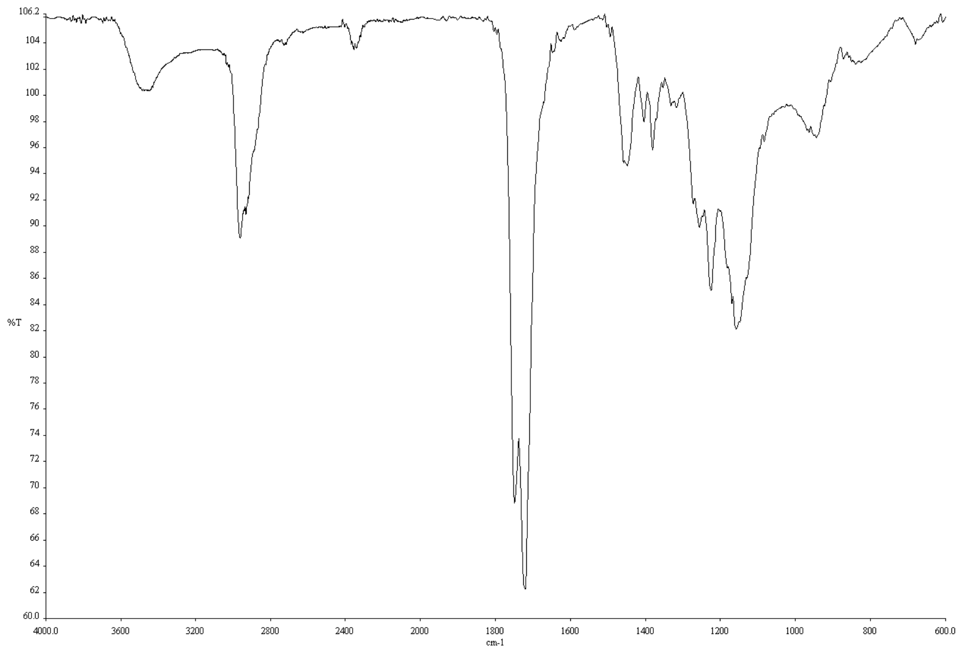

Infrared spectrum (Thin Film, NaCl) of compound **S3b**.

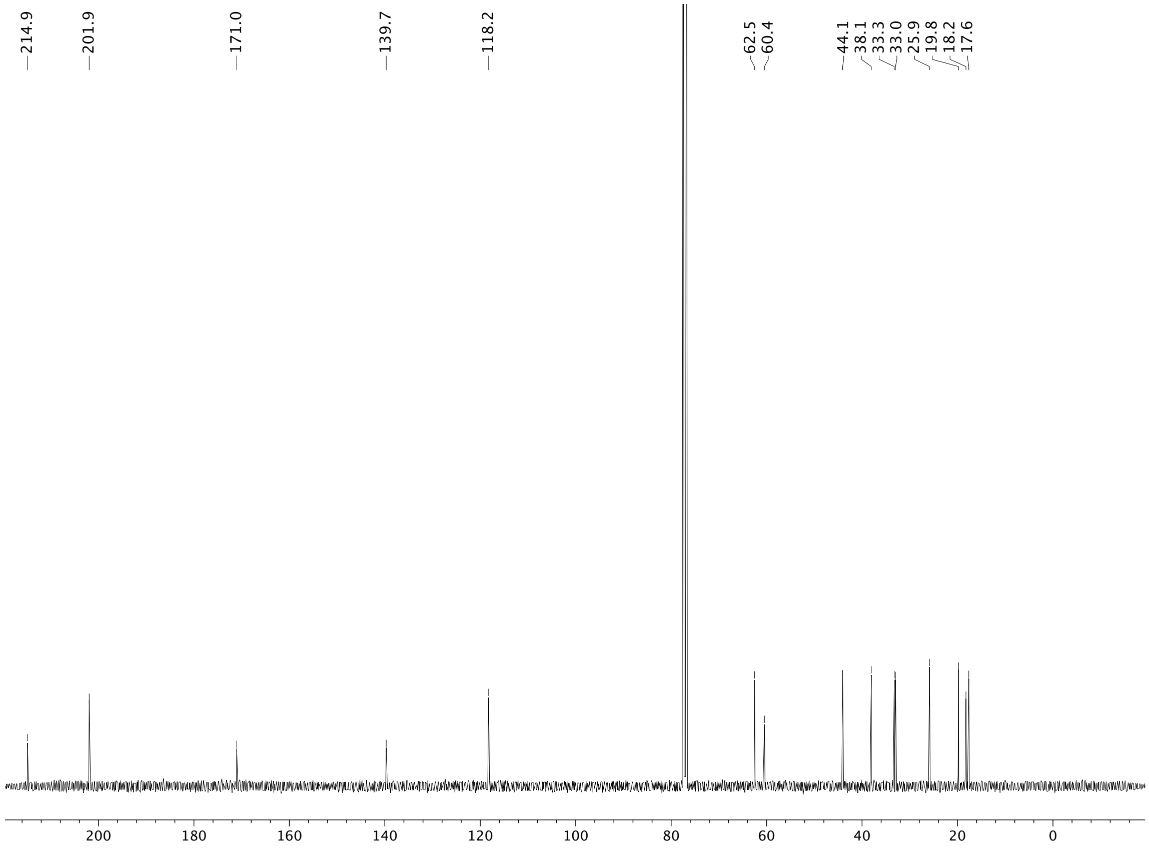

^{13}C NMR (100 MHz, $CDCl_3$) of compound **S3b**.

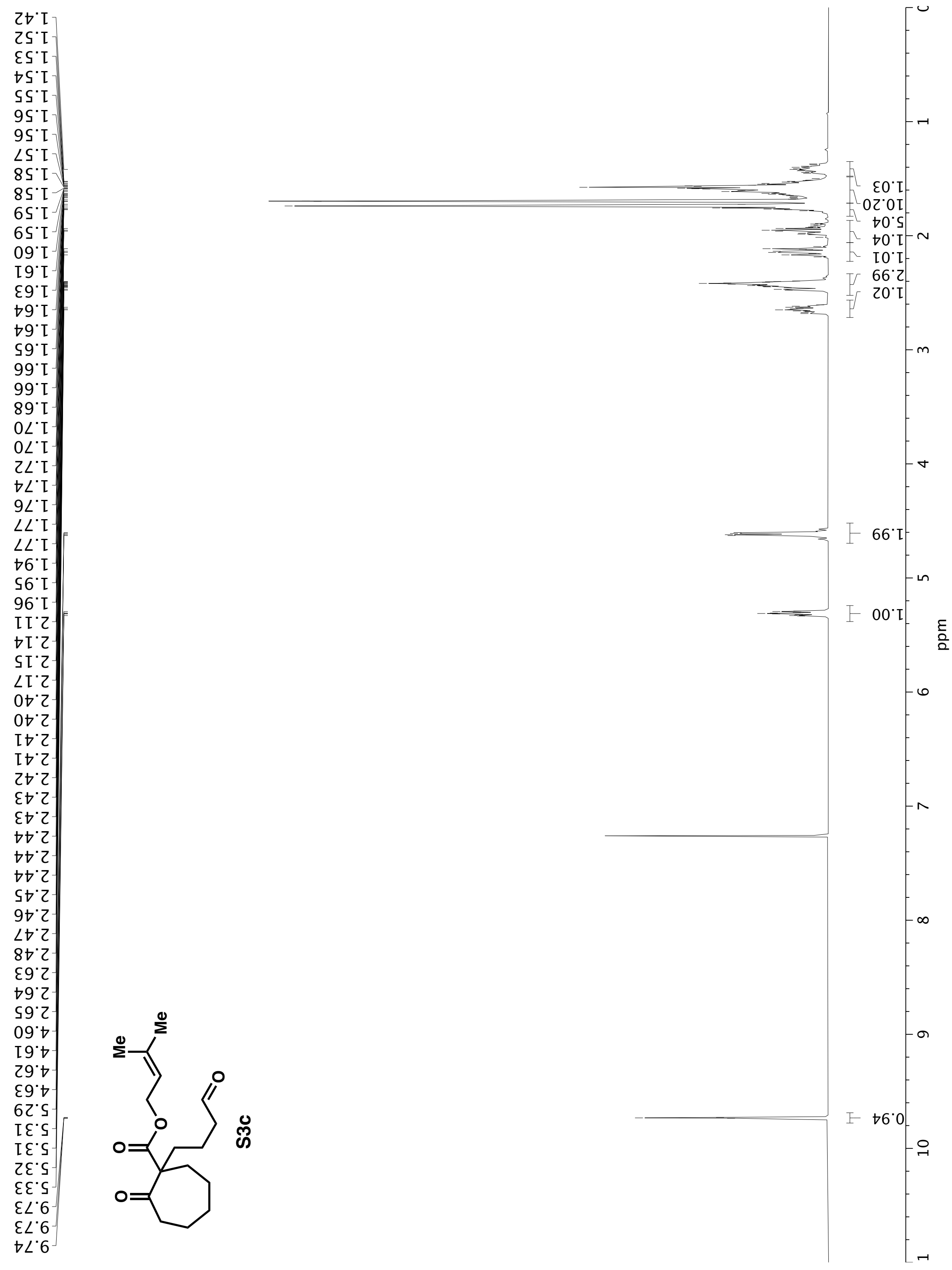

^{1}H NMR (400 MHz, $CDCl_3$) of compound **S3c**.

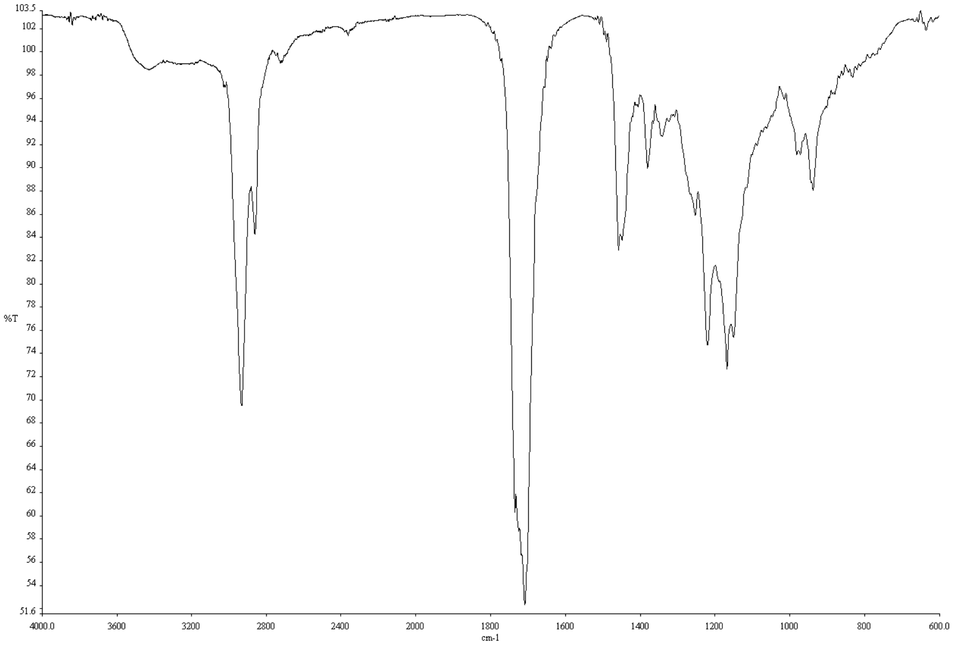

Infrared spectrum (Thin Film, NaCl) of compound **S3c**.

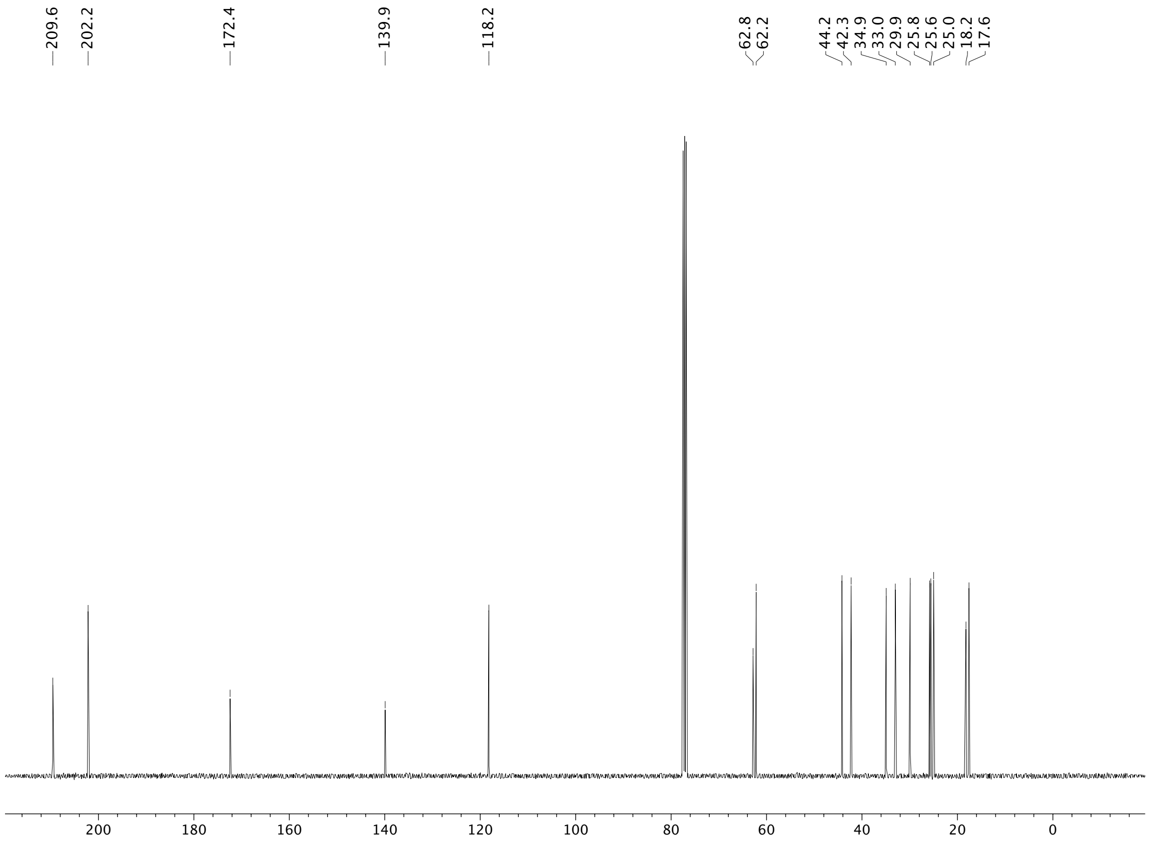

^{13}C NMR (100 MHz, $CDCl_3$) of compound **S3c**.

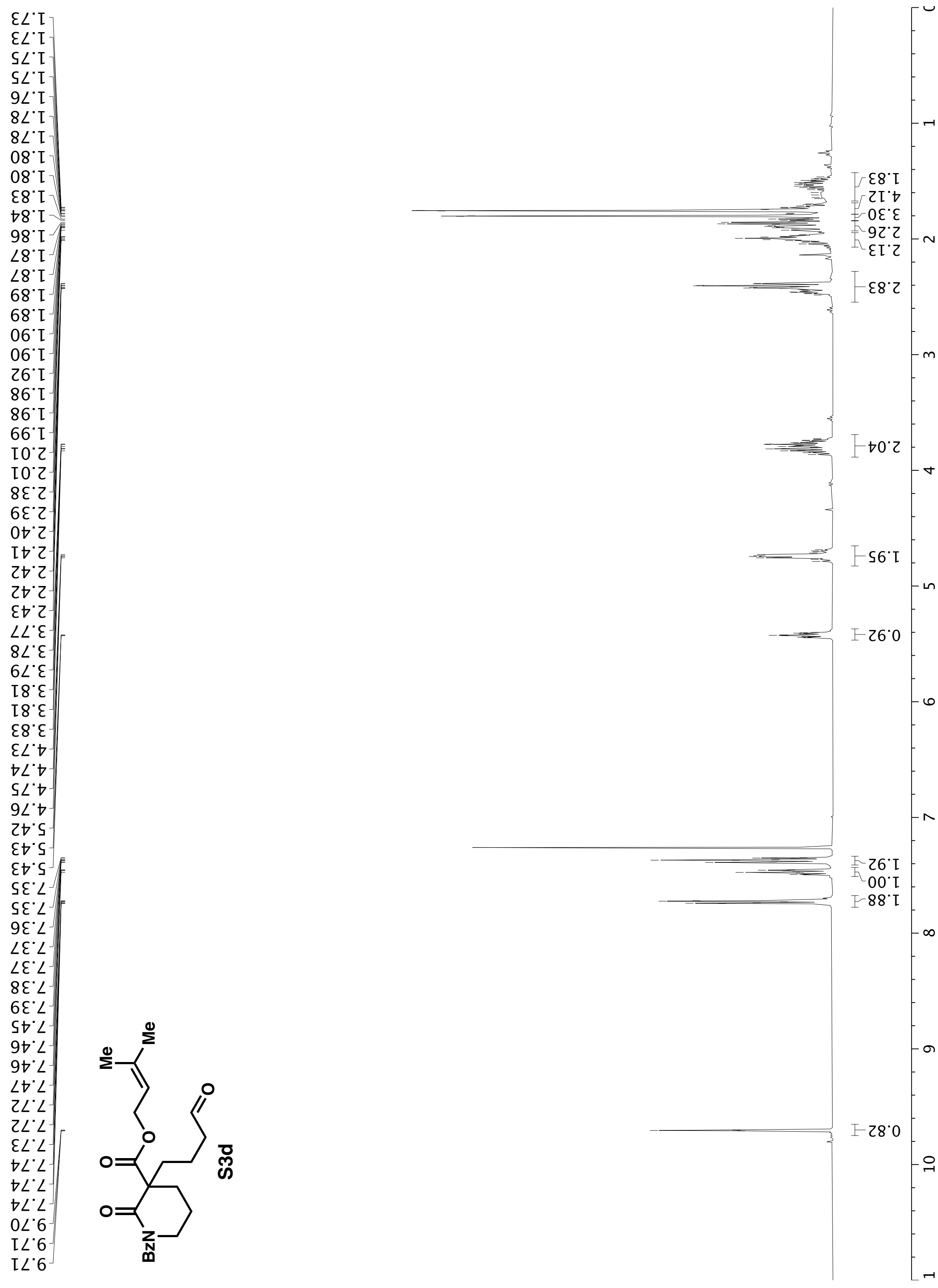

^{1}H NMR (400 MHz, $CDCl_3$) of compound **S3d**.

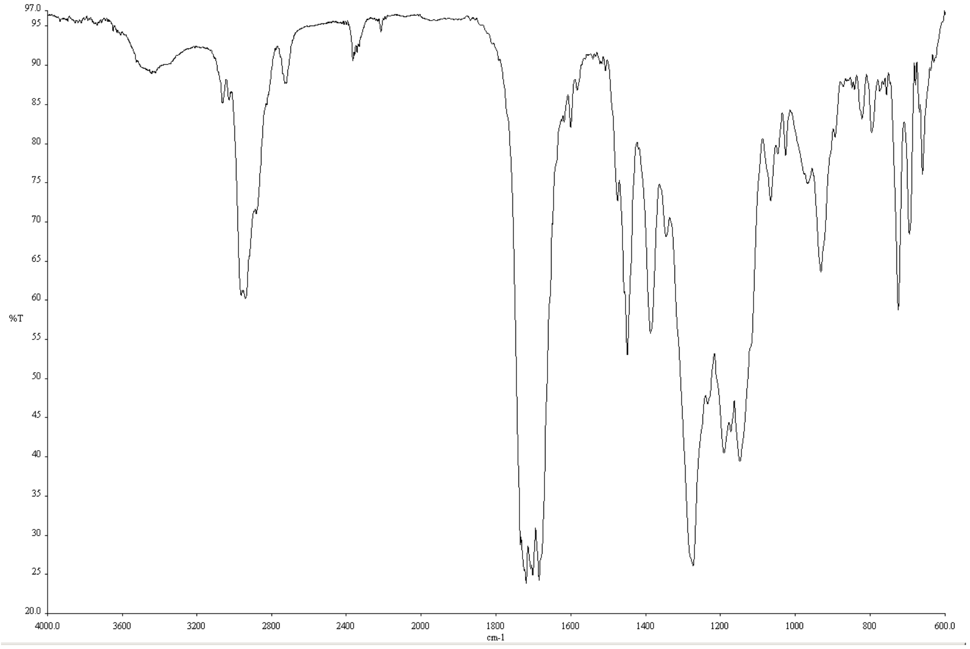

Infrared spectrum (Thin Film, NaCl) of compound **S3d**.

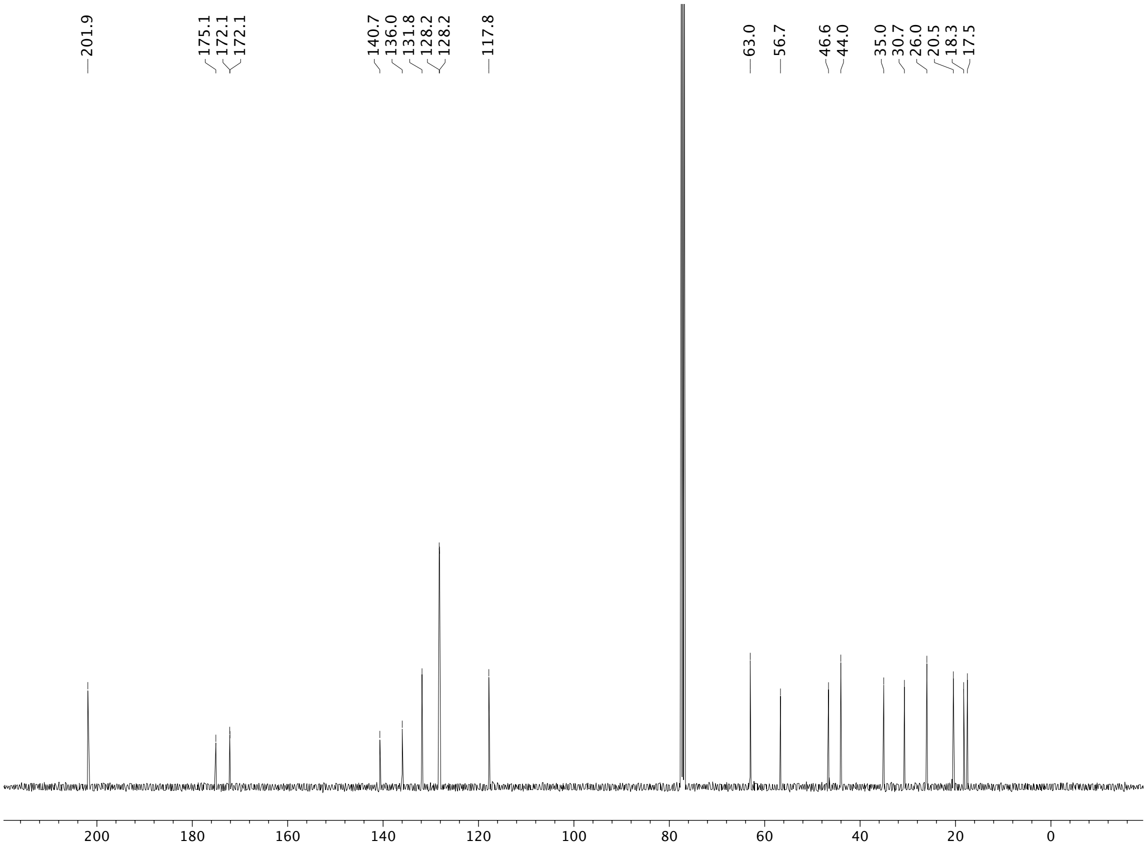

^{13}C NMR (100 MHz, $CDCl_3$) of compound **S3d**.

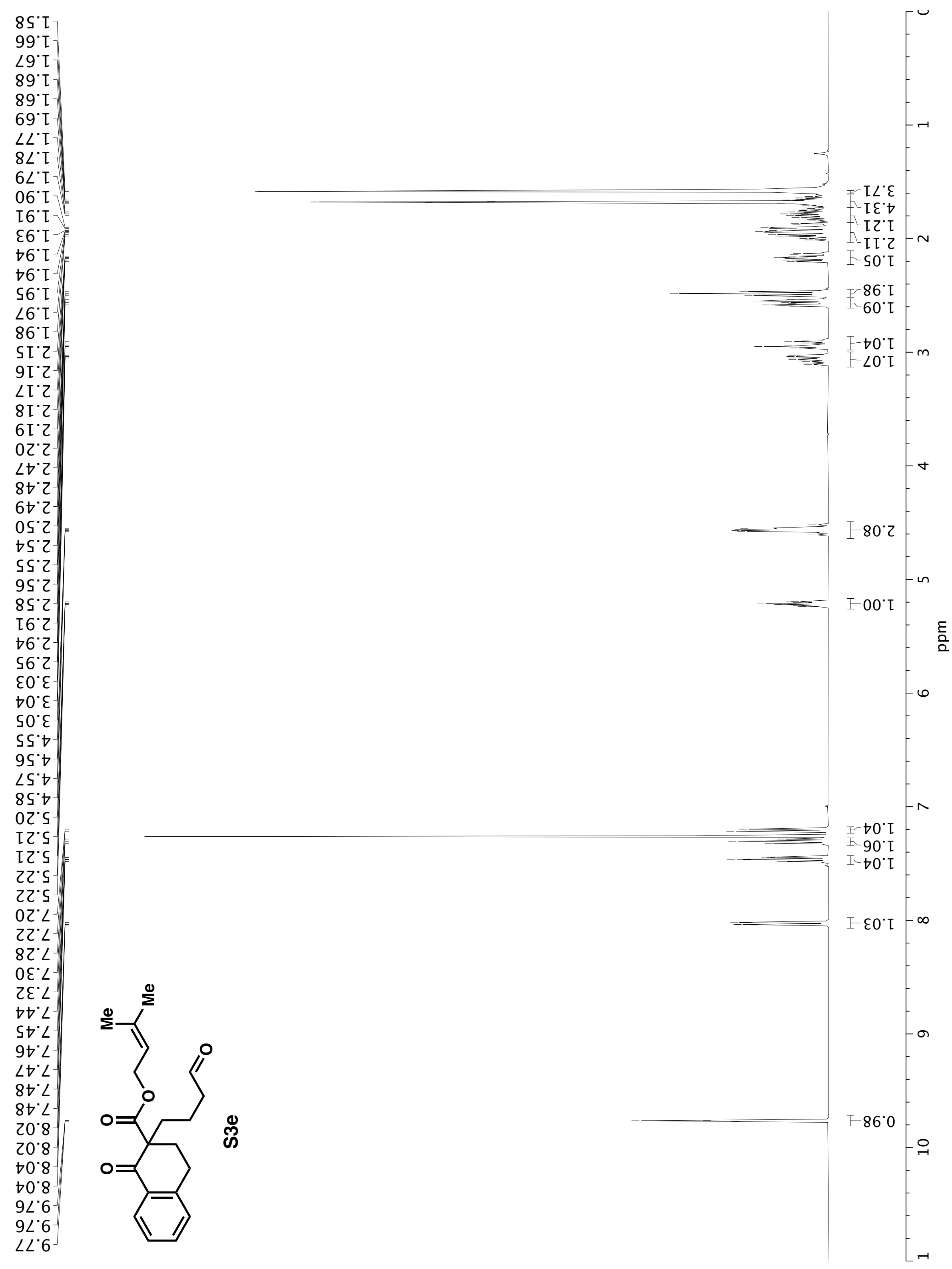

^{1}H NMR (400 MHz, $CDCl_3$) of compound **S3e**.

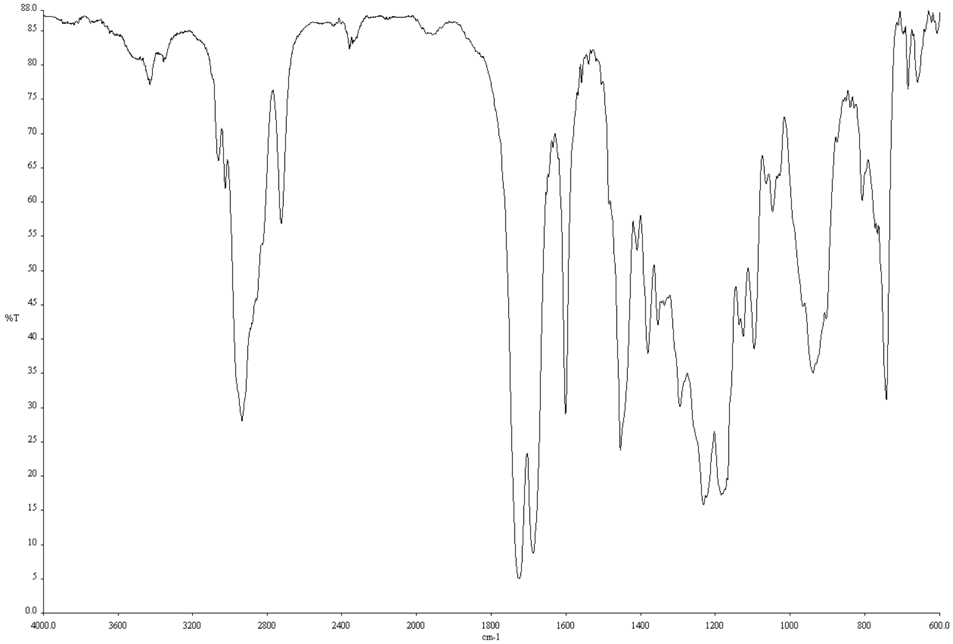

Infrared spectrum (Thin Film, NaCl) of compound **S3e**.

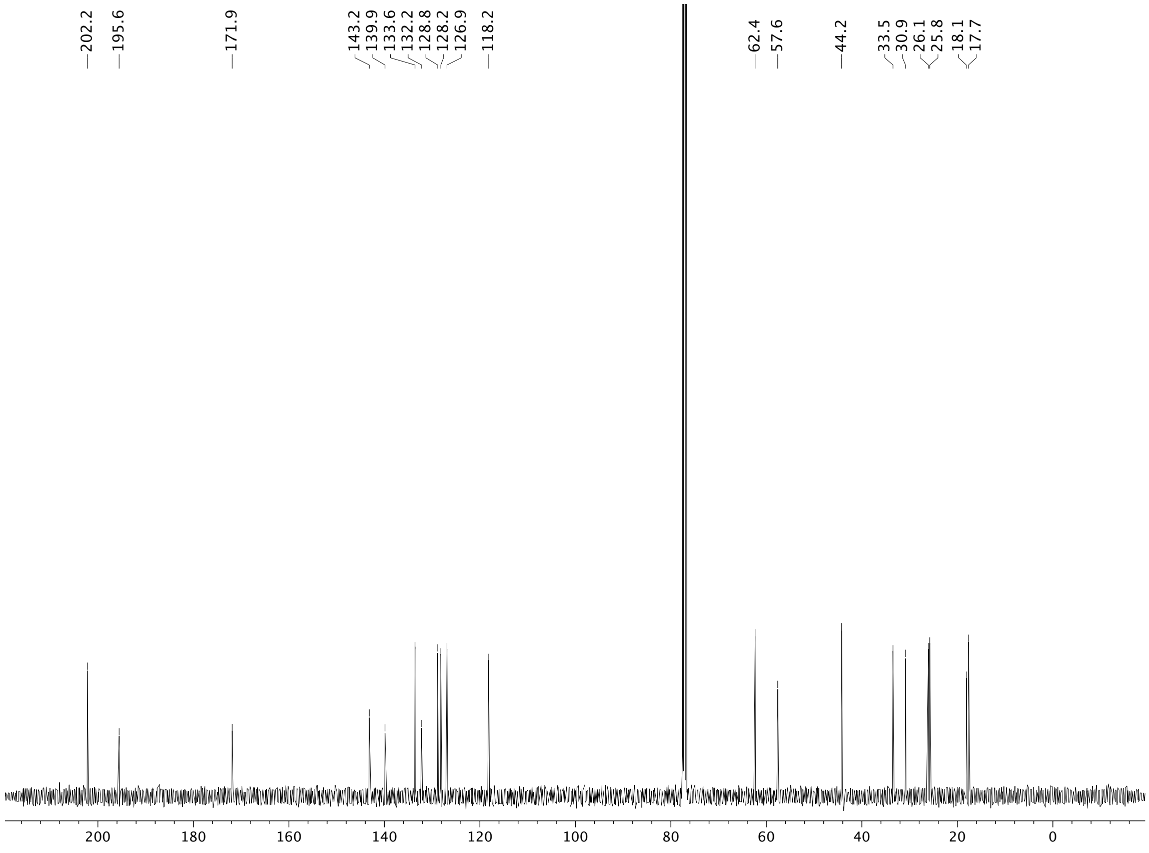

^{13}C NMR (100 MHz, $CDCl_3$) of compound **S3e**.

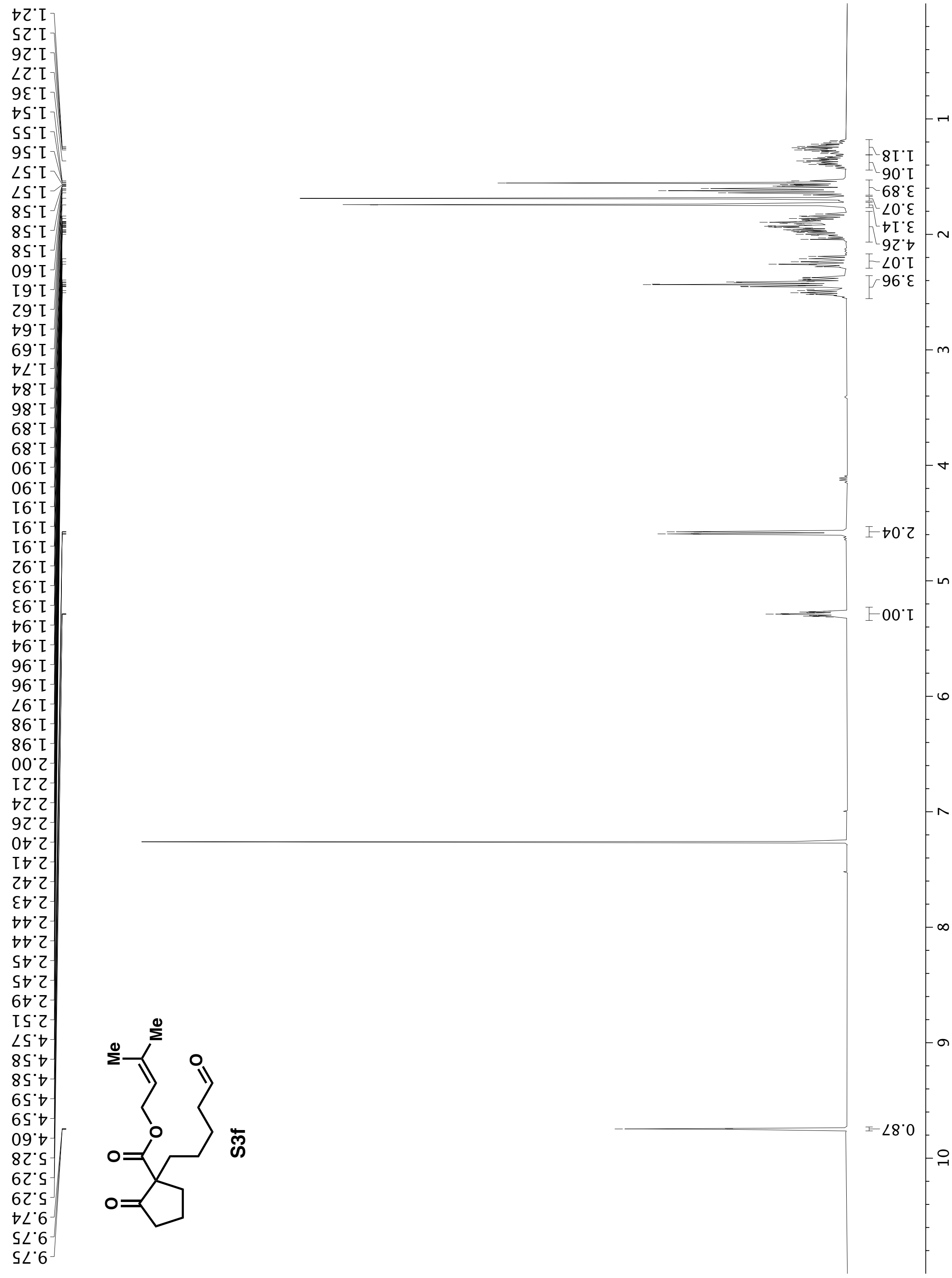

^{1}H NMR (400 MHz, $CDCl_3$) of compound **S3f**.

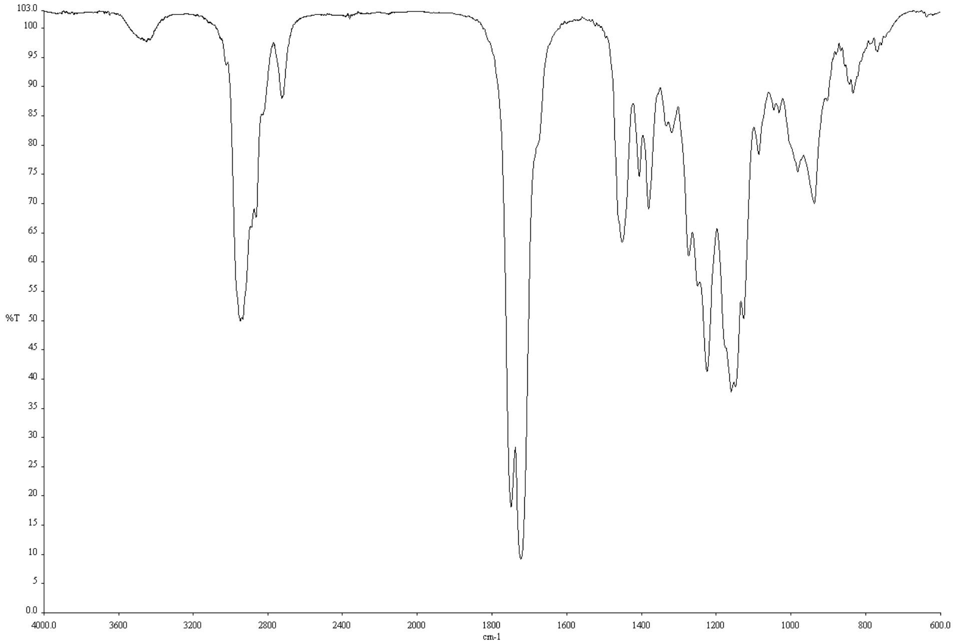

Infrared spectrum (Thin Film, NaCl) of compound **S3f**.

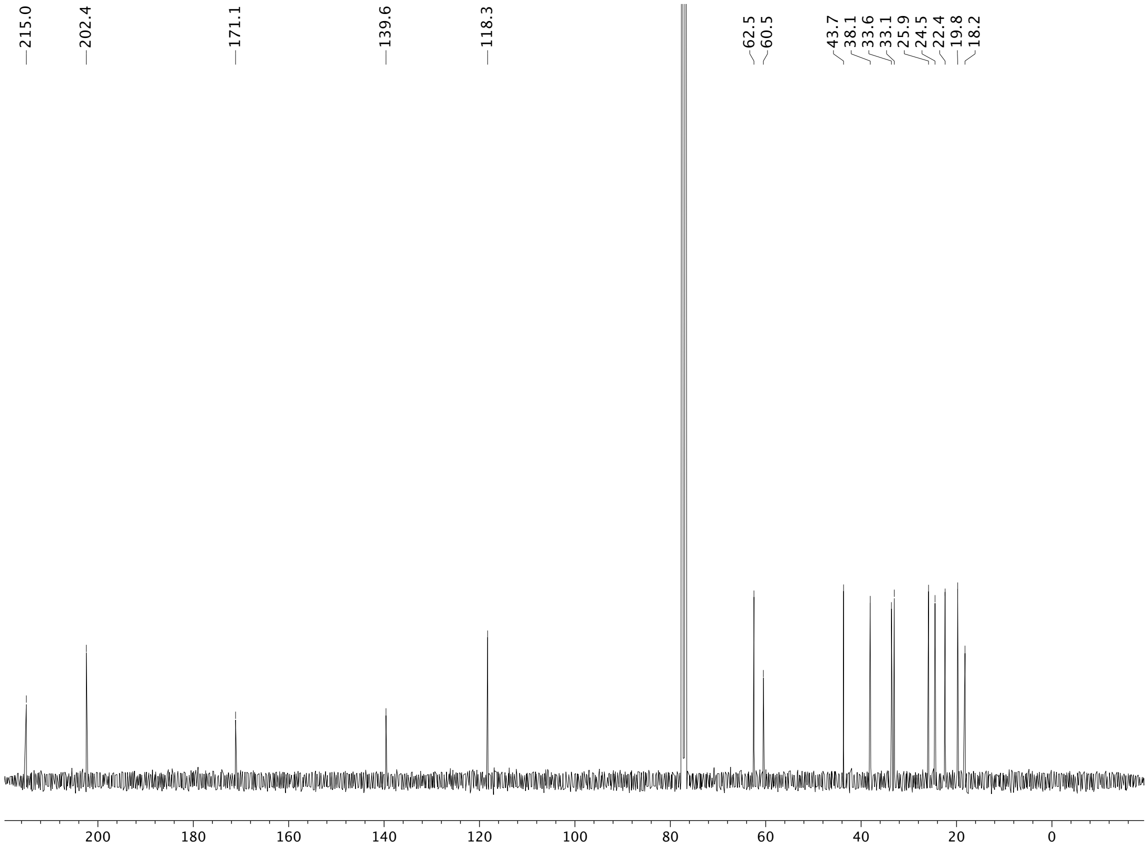

^{13}C NMR (100 MHz, $CDCl_3$) of compound **S3f**.

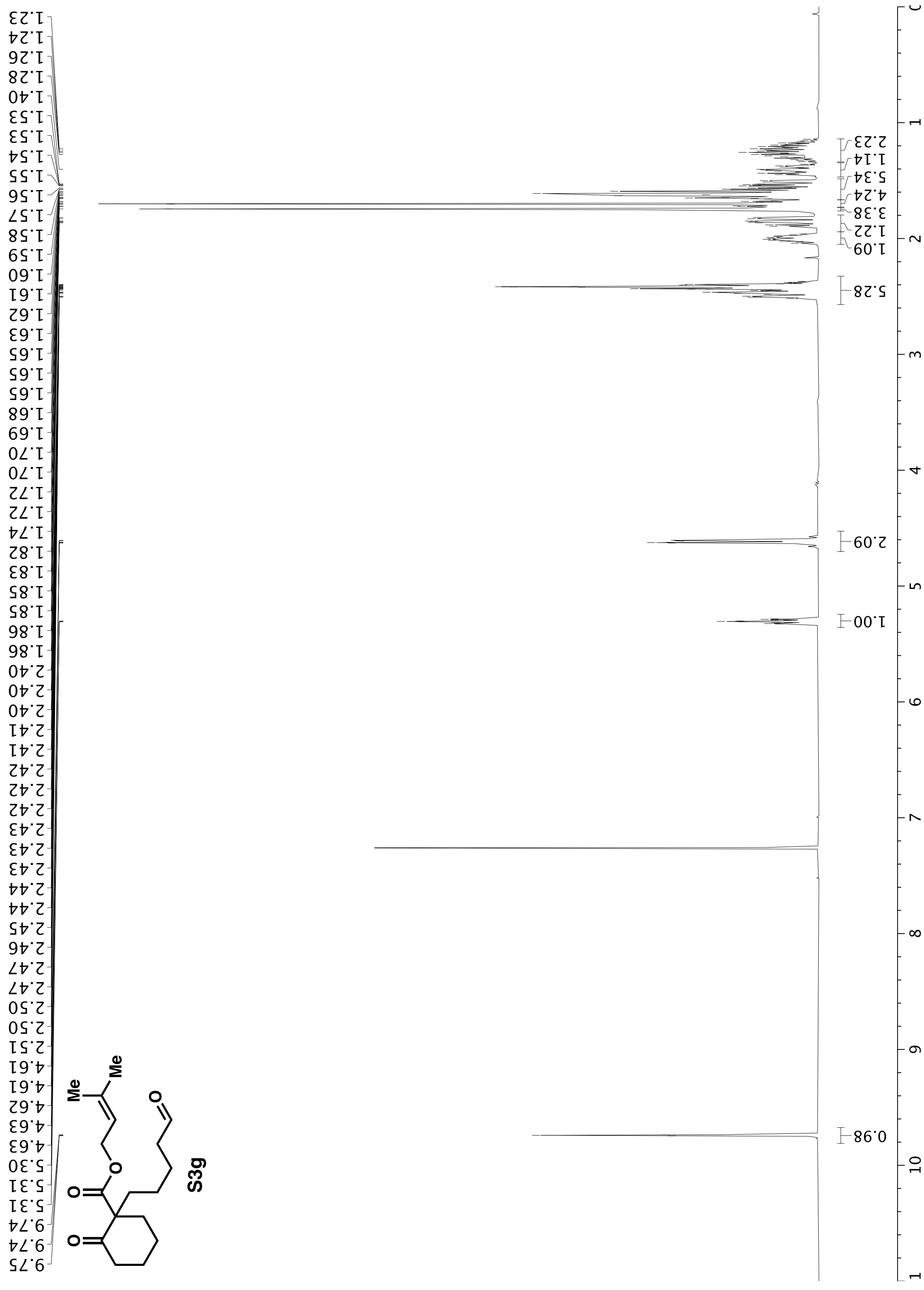

^{1}H NMR (400 MHz, $CDCl_3$) of compound **S3g**.

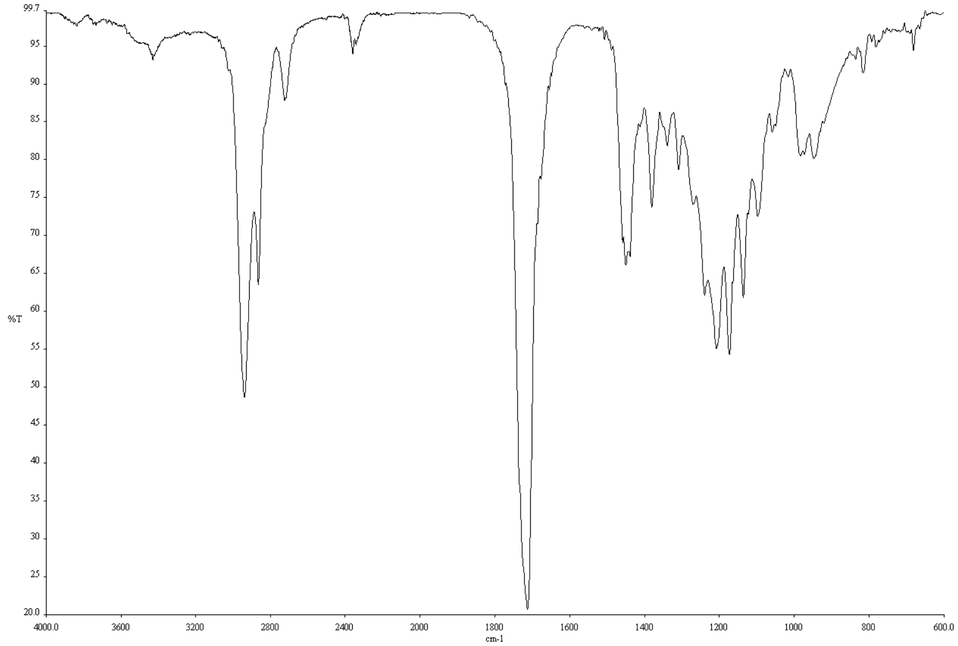

Infrared spectrum (Thin Film, NaCl) of compound **S3g**.

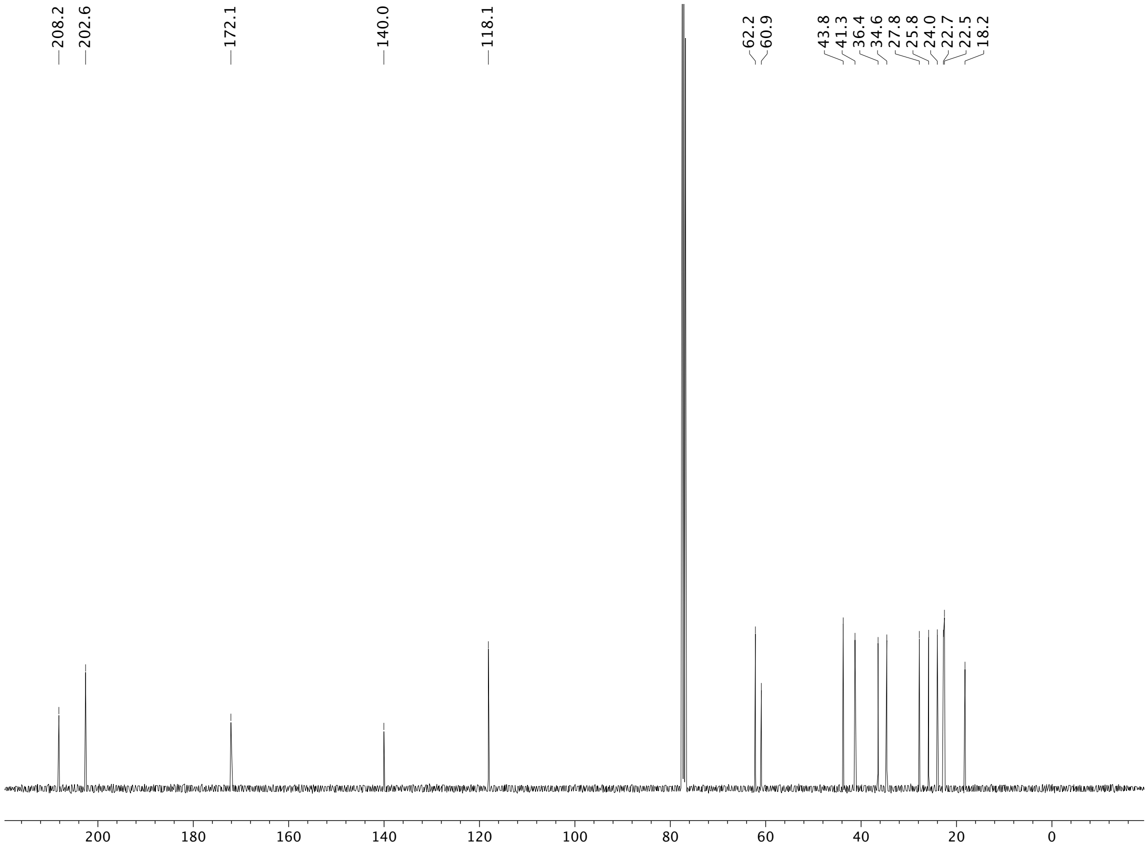

^{13}C NMR (100 MHz, $CDCl_3$) of compound **S3g**.

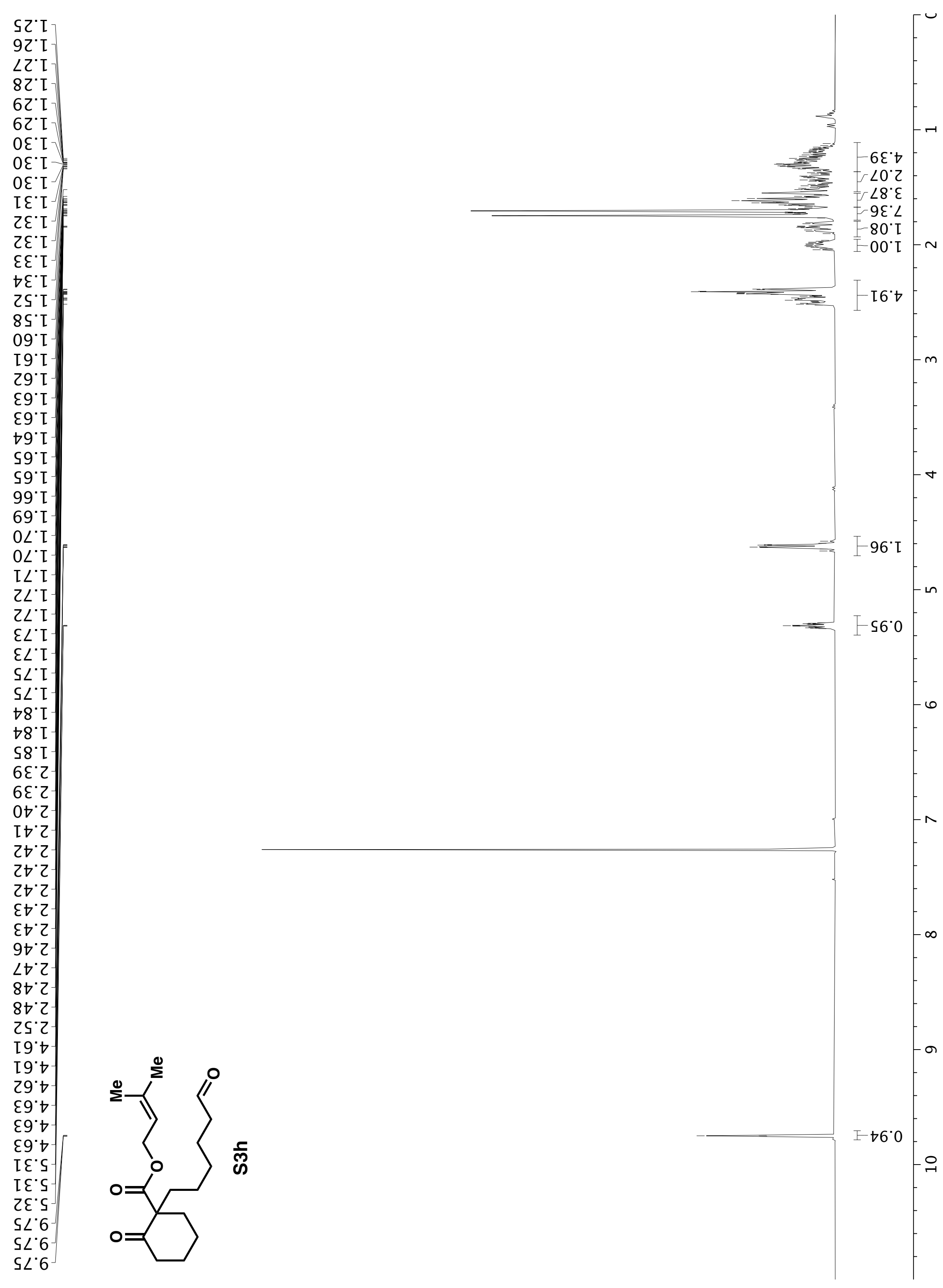

^1H NMR (400 MHz, $CDCl_3$) of compound **S3h**.

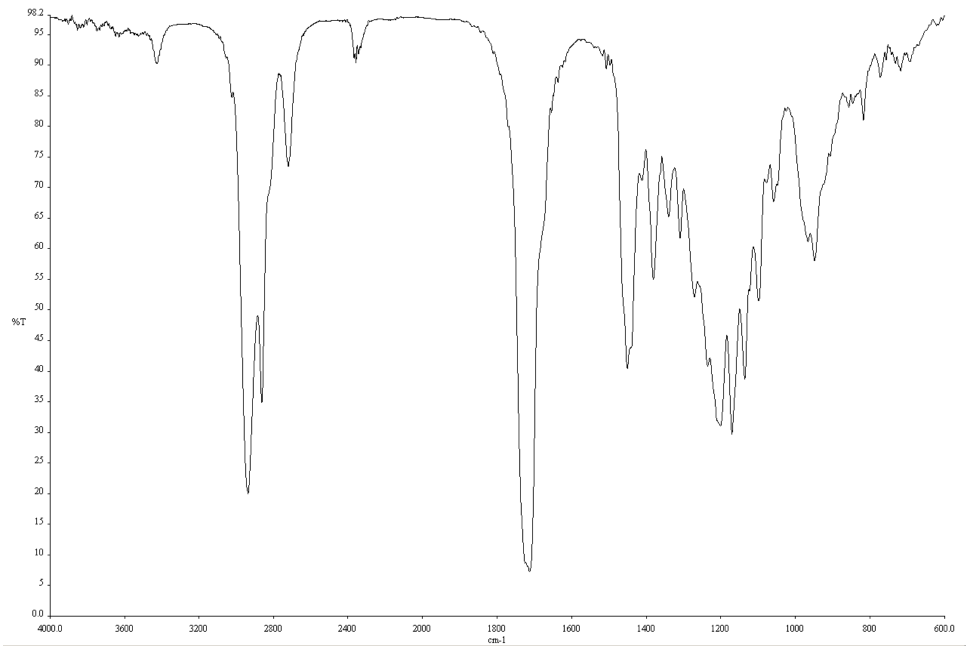

Infrared spectrum (Thin Film, NaCl) of compound **S3h**.

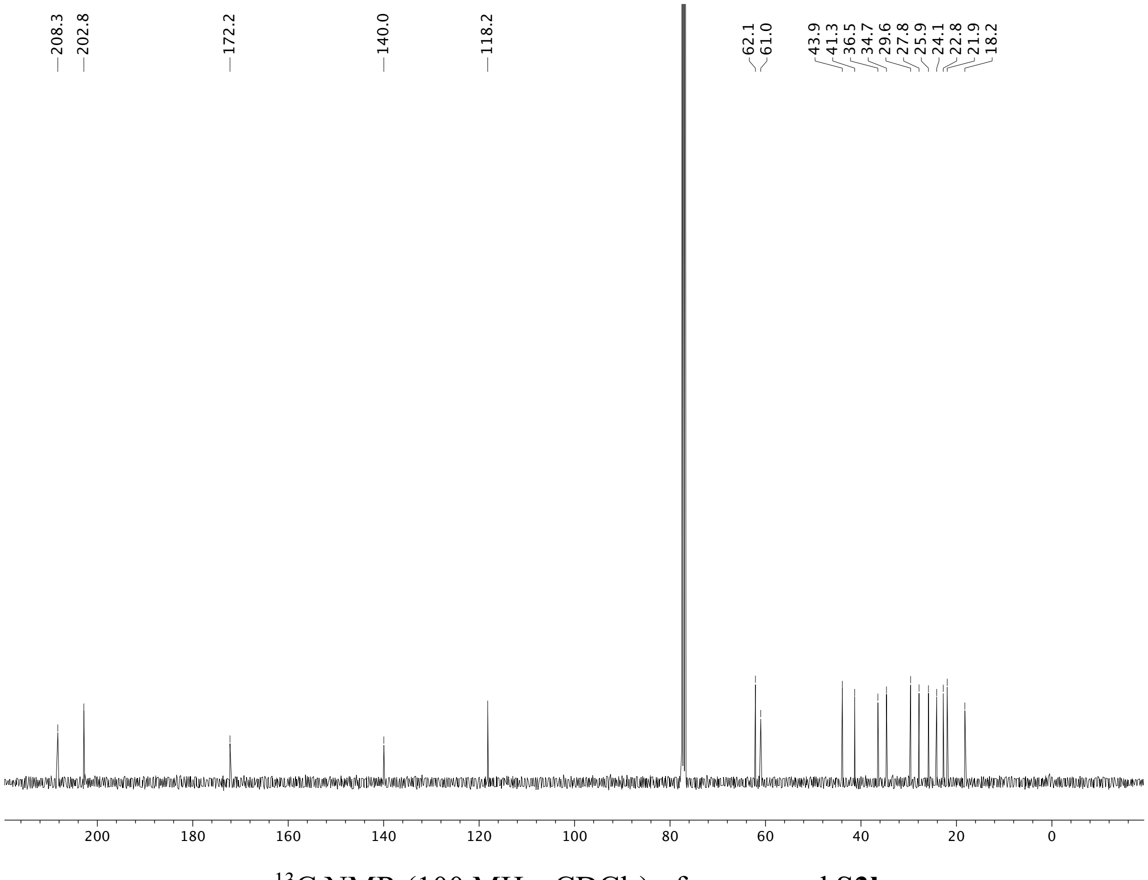

^{13}C NMR (100 MHz, $CDCl_3$) of compound **S3h**.

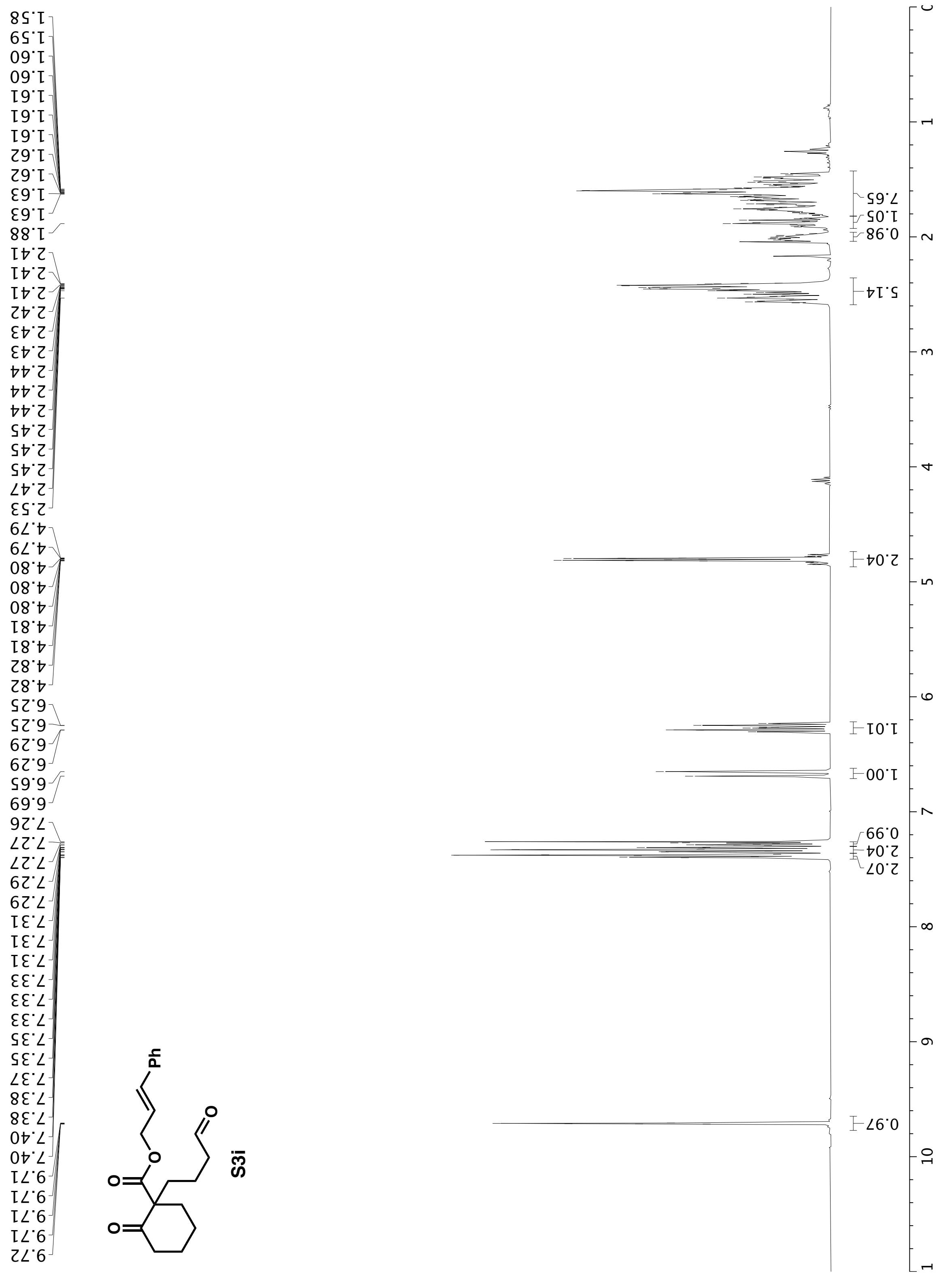

^{1}H NMR (400 MHz, $CDCl_3$) of compound **S3i**.

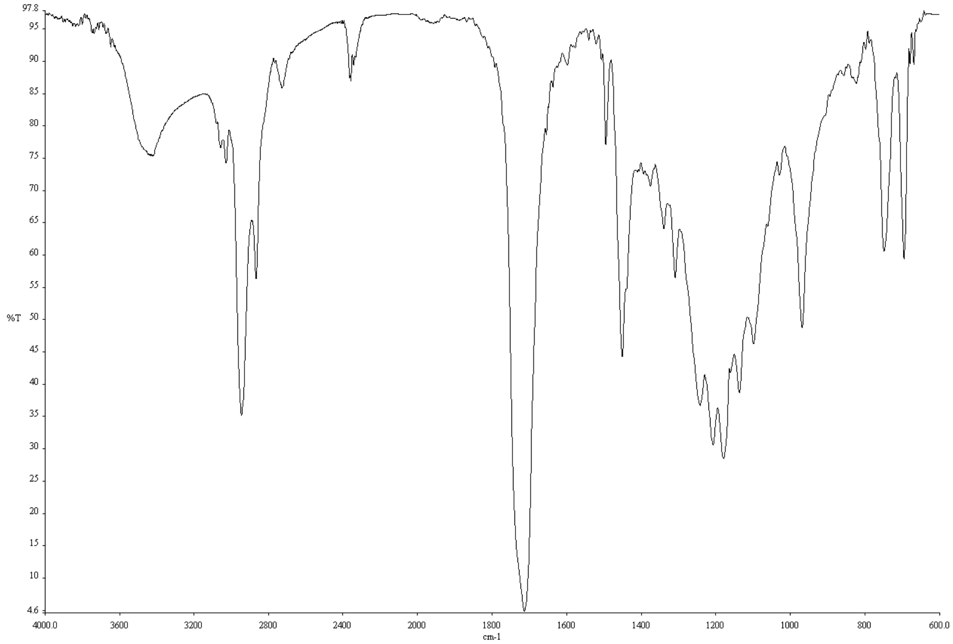

Infrared spectrum (Thin Film, NaCl) of compound **S3i**.

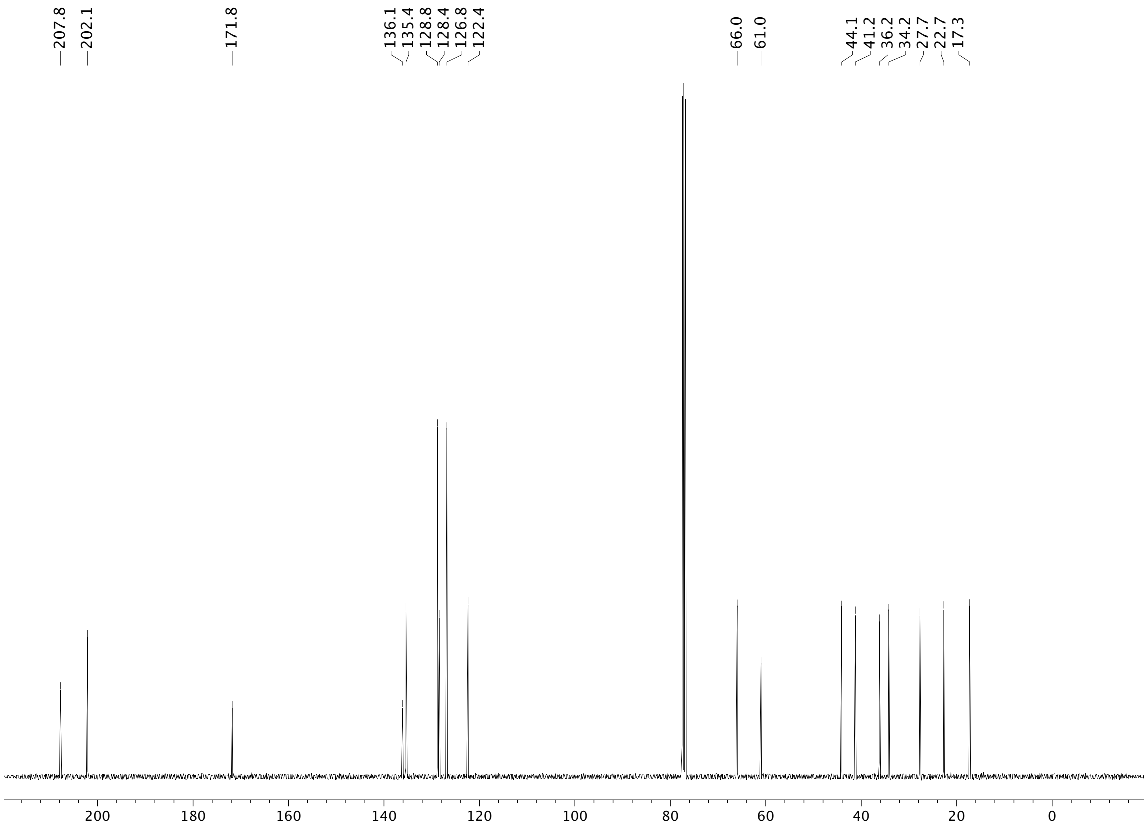

^{13}C NMR (100 MHz, $CDCl_3$) of compound **S3i**.

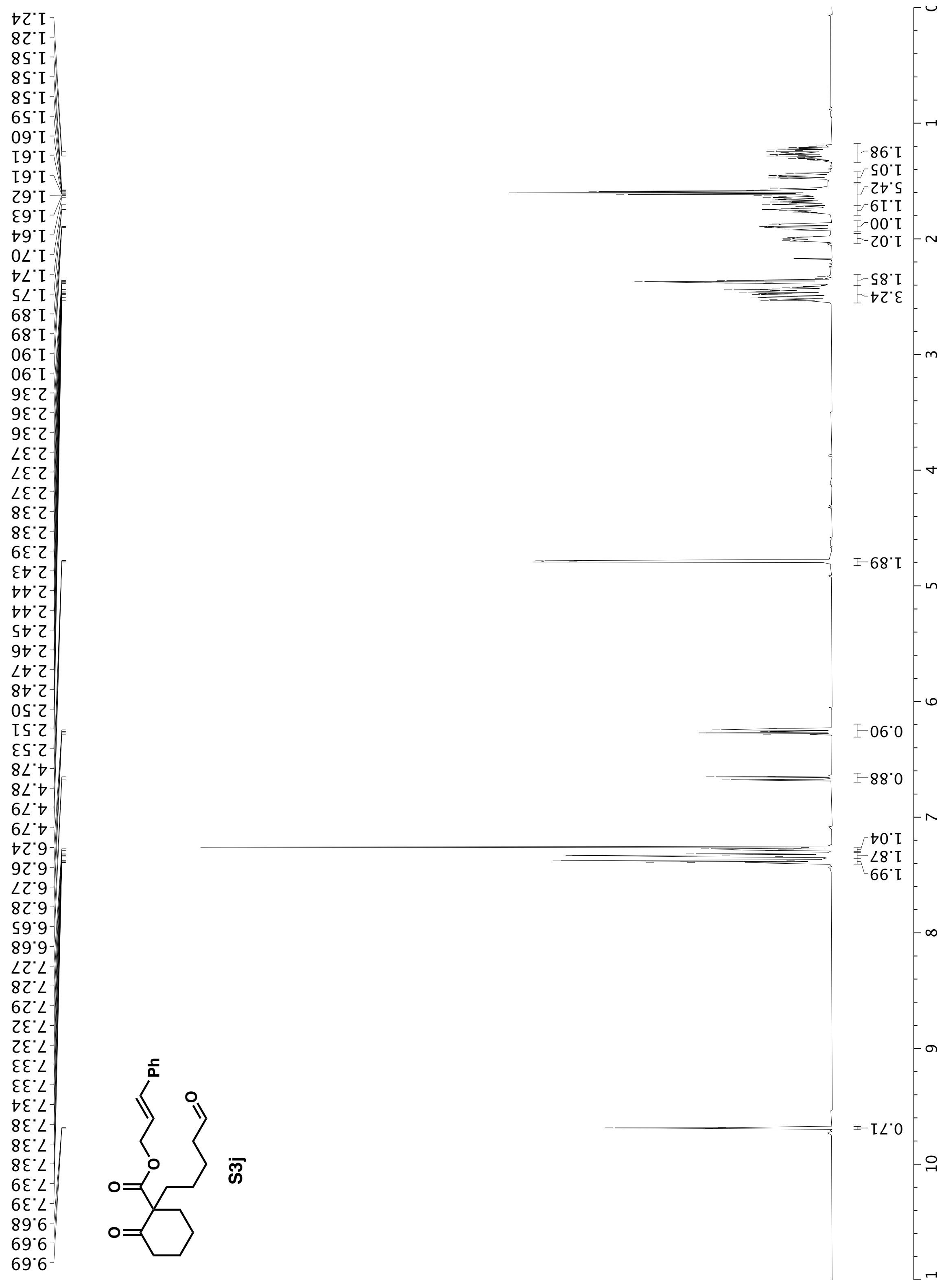

^{1}H NMR (600 MHz, $CDCl_3$) of compound **S3j**.

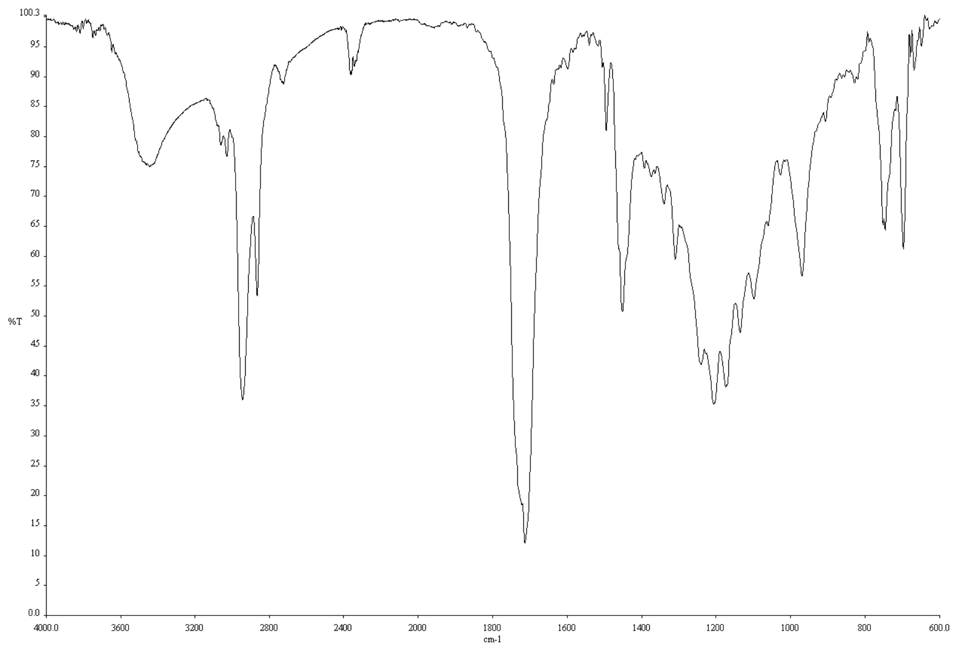

Infrared spectrum (Thin Film, NaCl) of compound **S3j**.

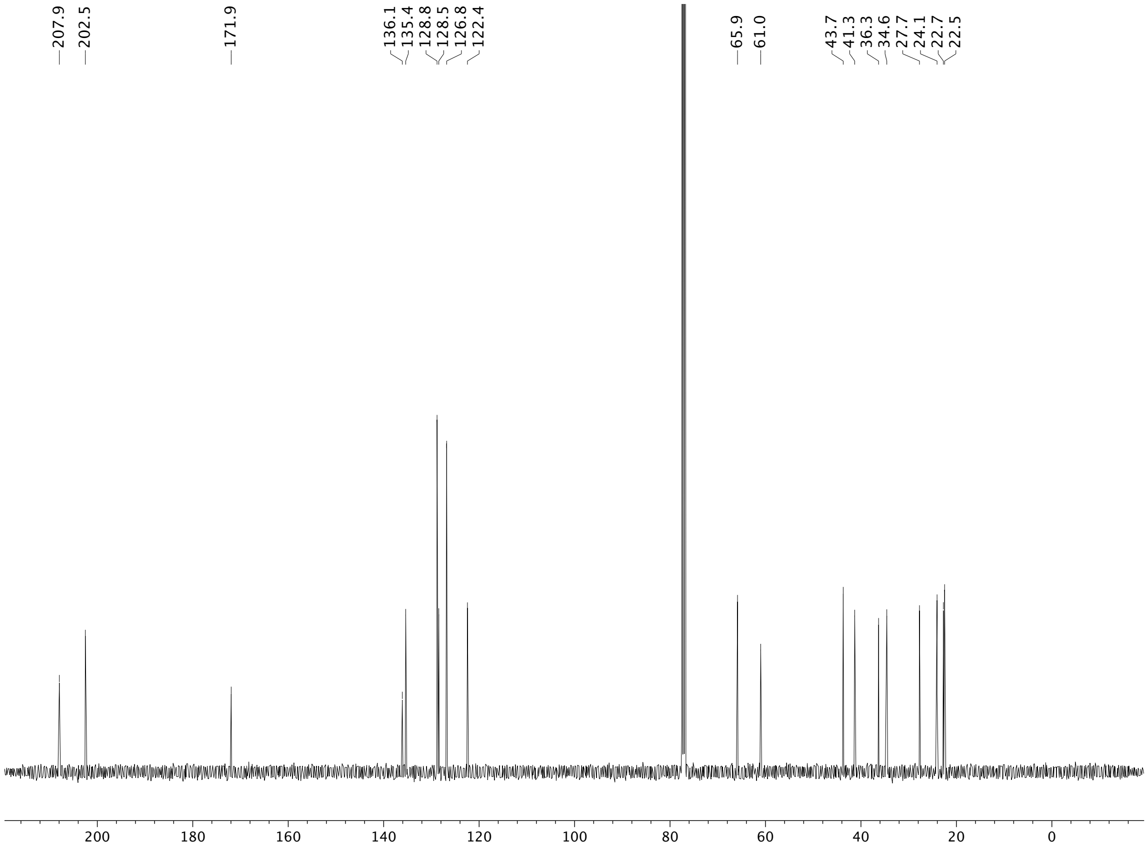

^{13}C NMR (100 MHz, $CDCl_3$) of compound **S3j**.

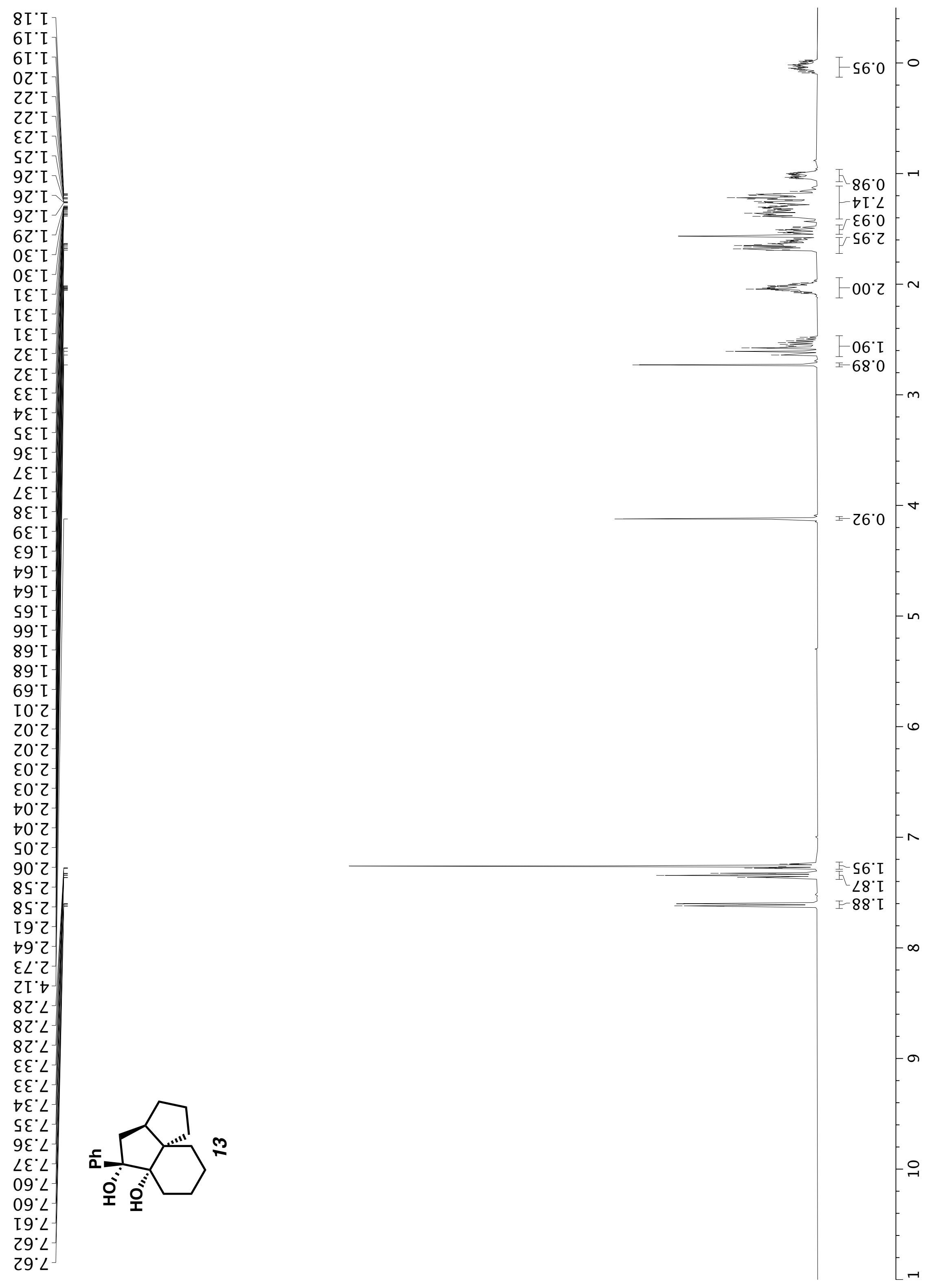

^{1}H NMR (400 MHz, $CDCl_3$) of compound **13**.

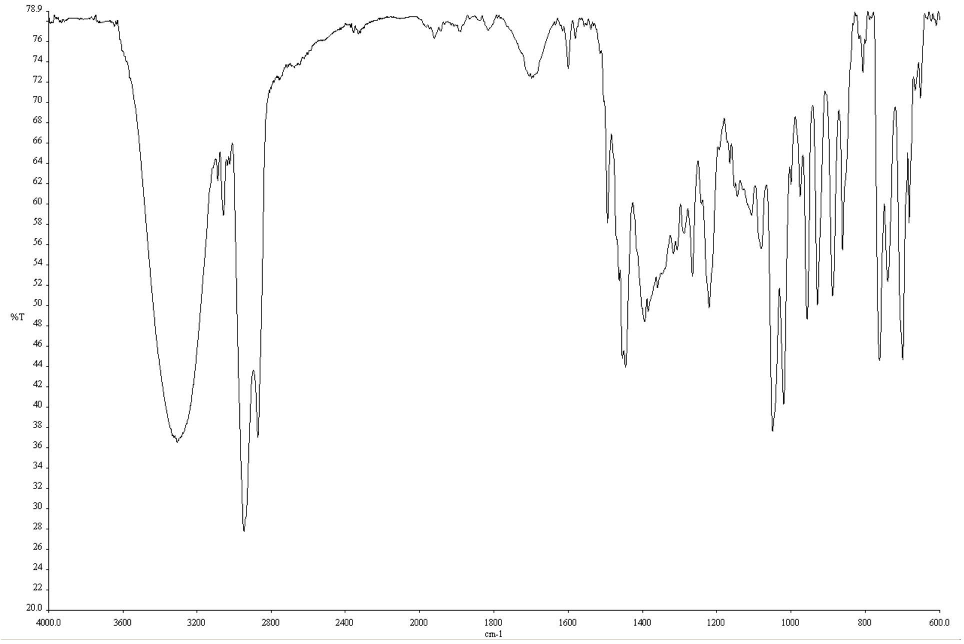

Infrared spectrum (Thin Film, NaCl) of compound **13**.

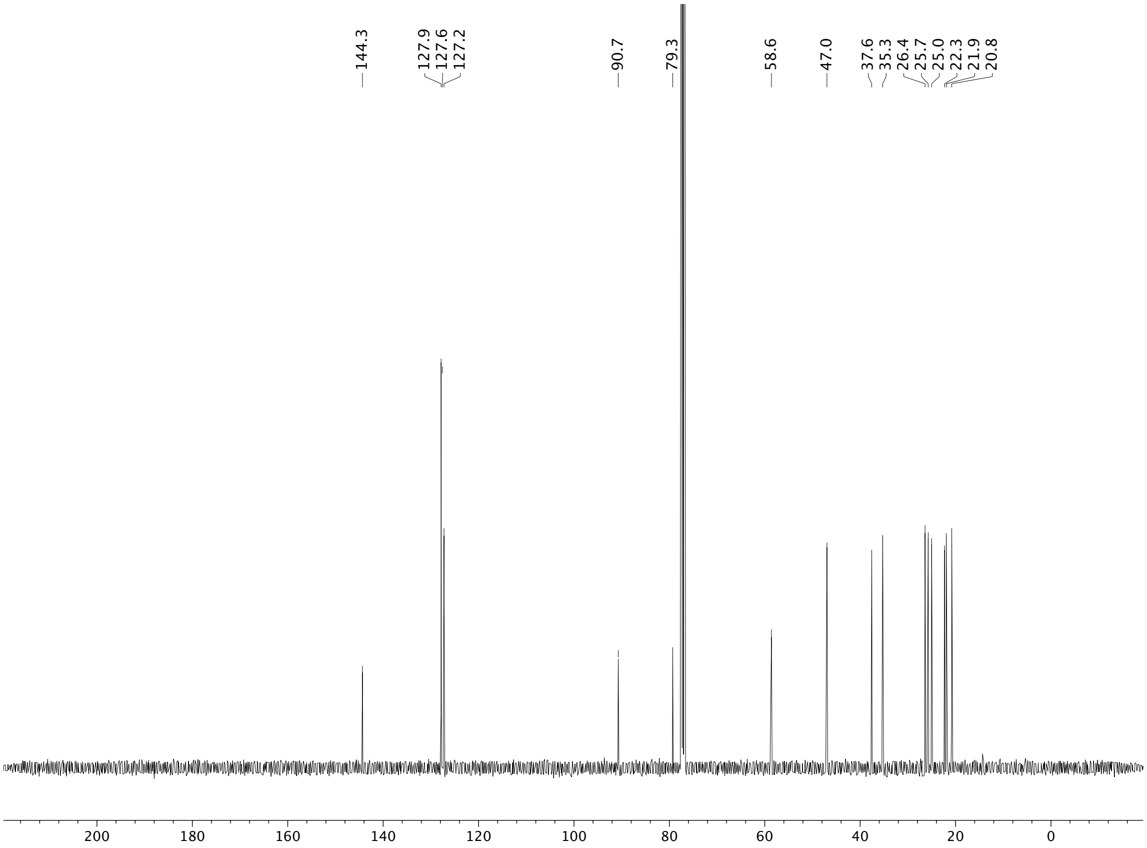

^{13}C NMR (100 MHz, $CDCl_3$) of compound **13**.

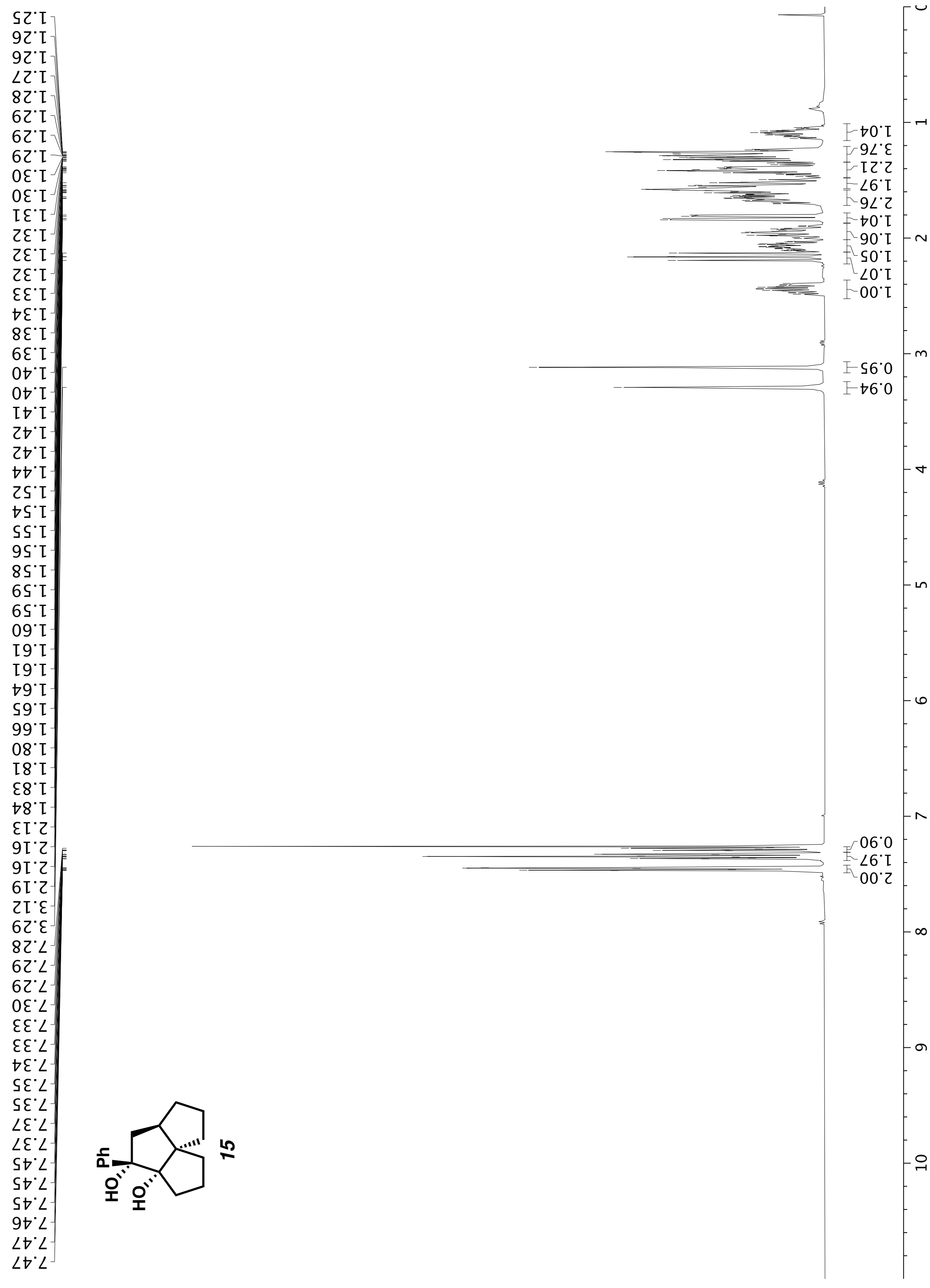

^{1}H NMR (400 MHz, $CDCl_3$) of compound **15**.

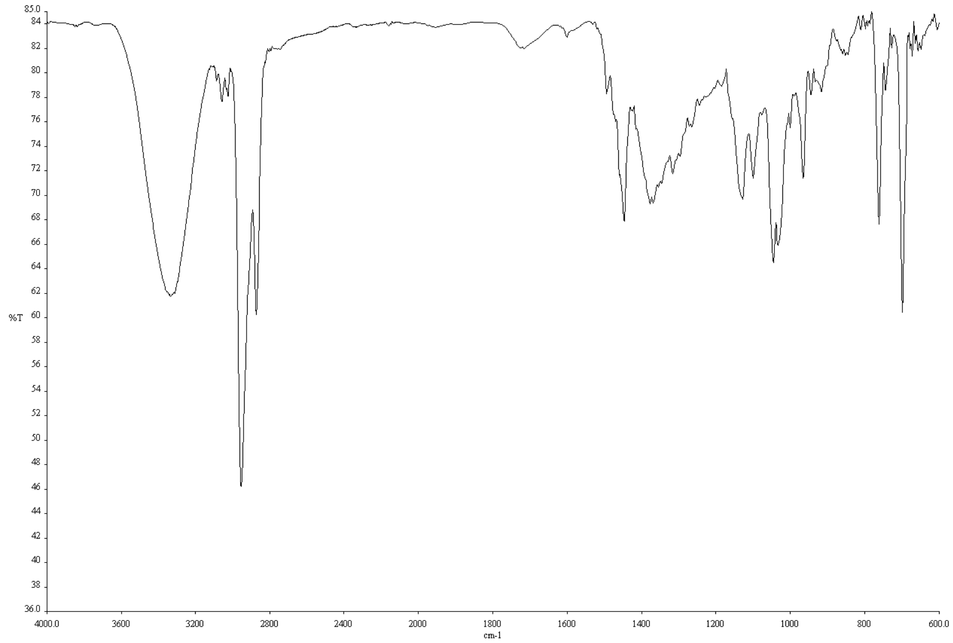

Infrared spectrum (Thin Film, NaCl) of compound **15**.

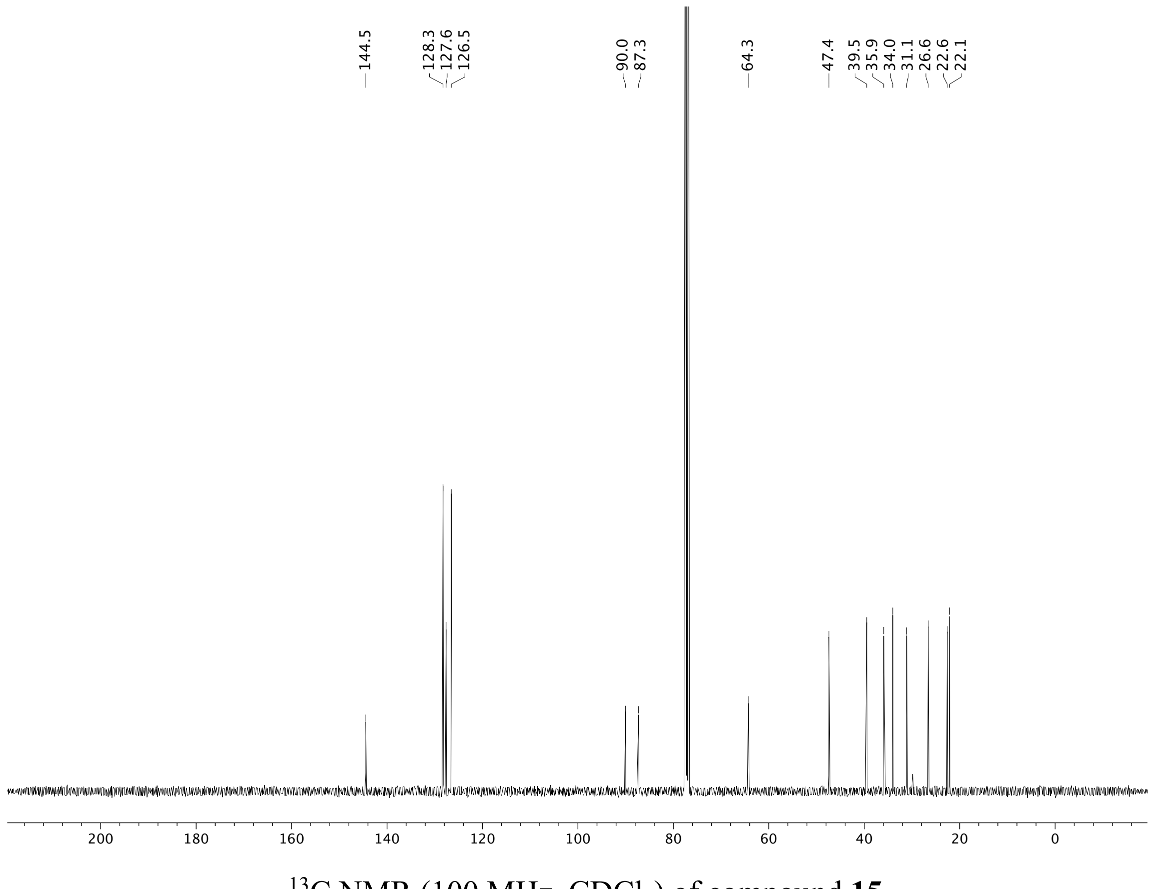

^{13}C NMR (100 MHz, $CDCl_3$) of compound **15**.

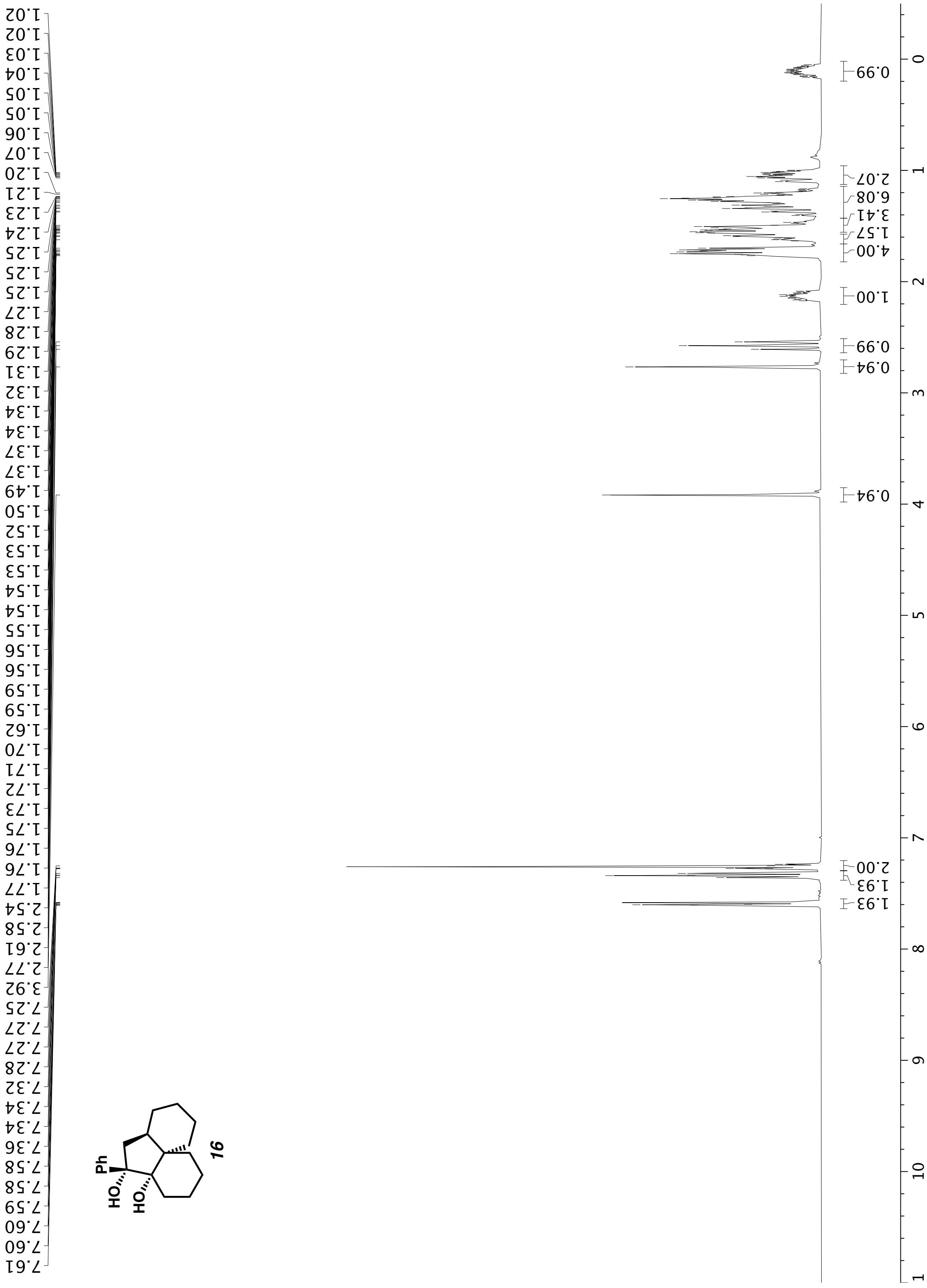

^{1}H NMR (400 MHz, CDCl$_3$) of compound **16**.

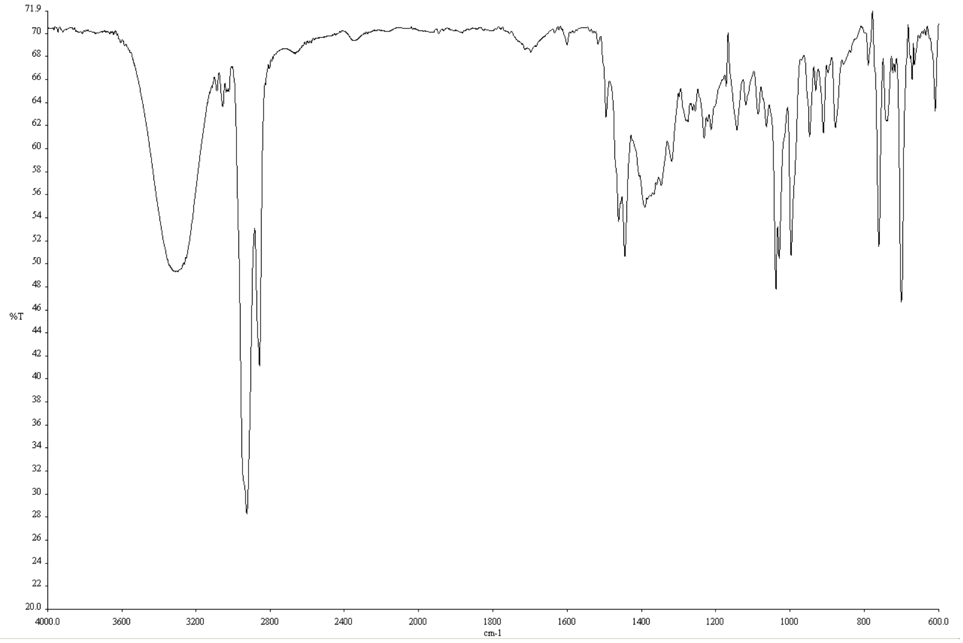

Infrared spectrum (Thin Film, NaCl) of compound **16**.

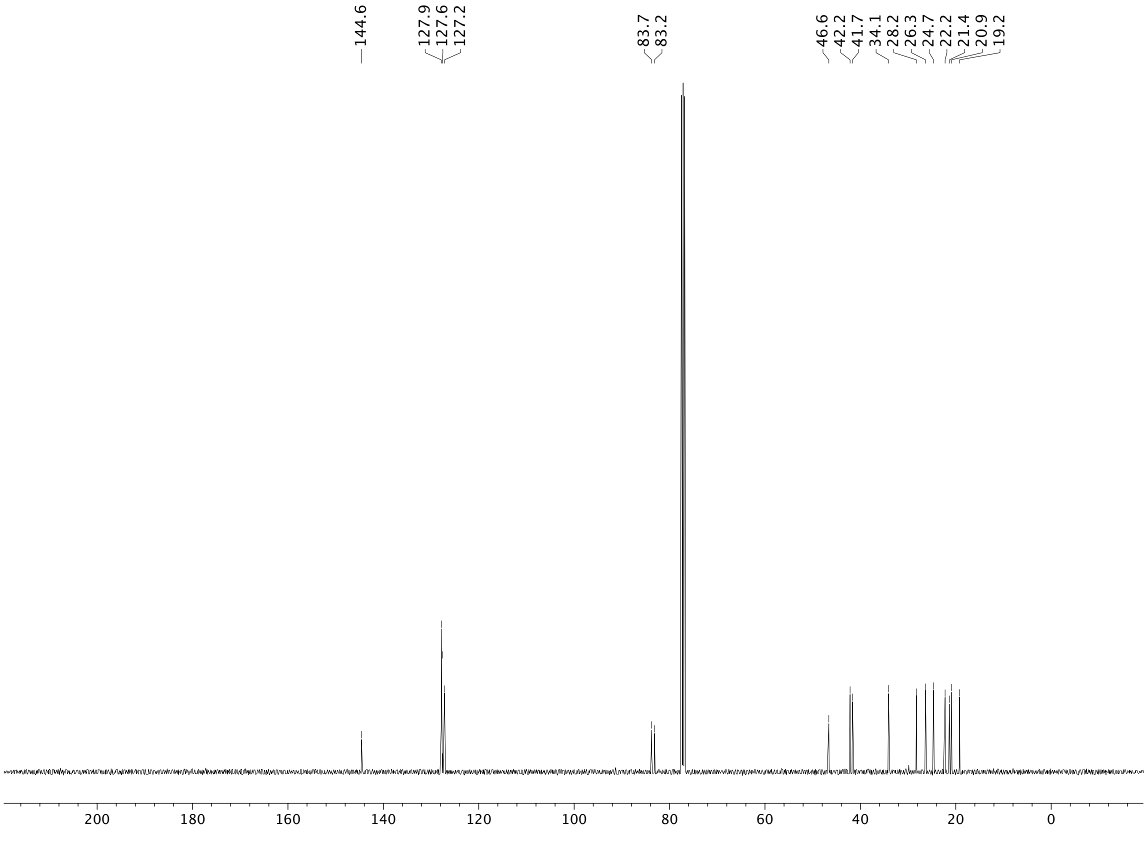

^{13}C NMR (100 MHz, $CDCl_3$) of compound **16**.

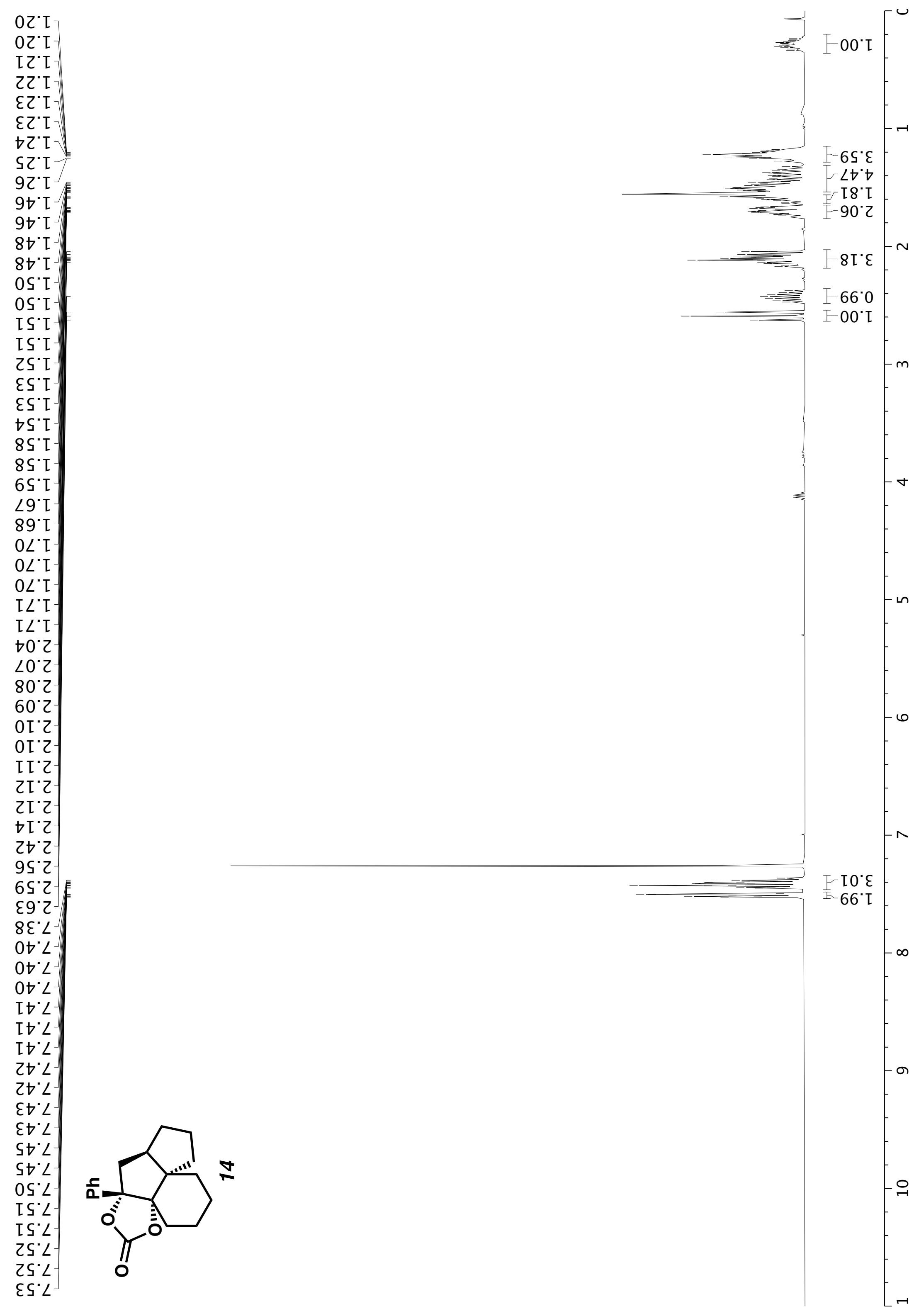

^{1}H NMR (400 MHz, $CDCl_3$) of compound **14**.

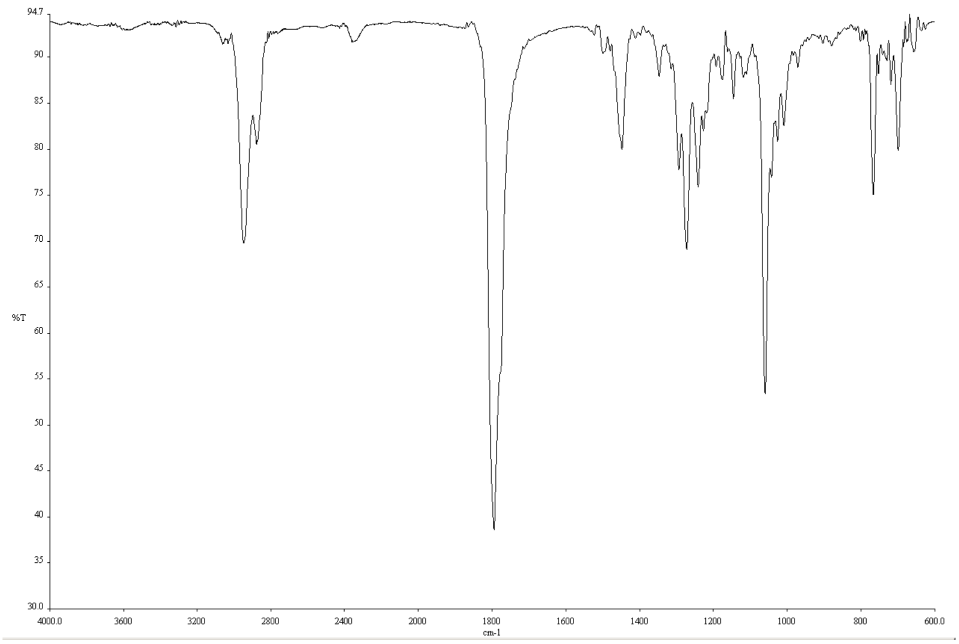

Infrared spectrum (Thin Film, NaCl) of compound **14**.

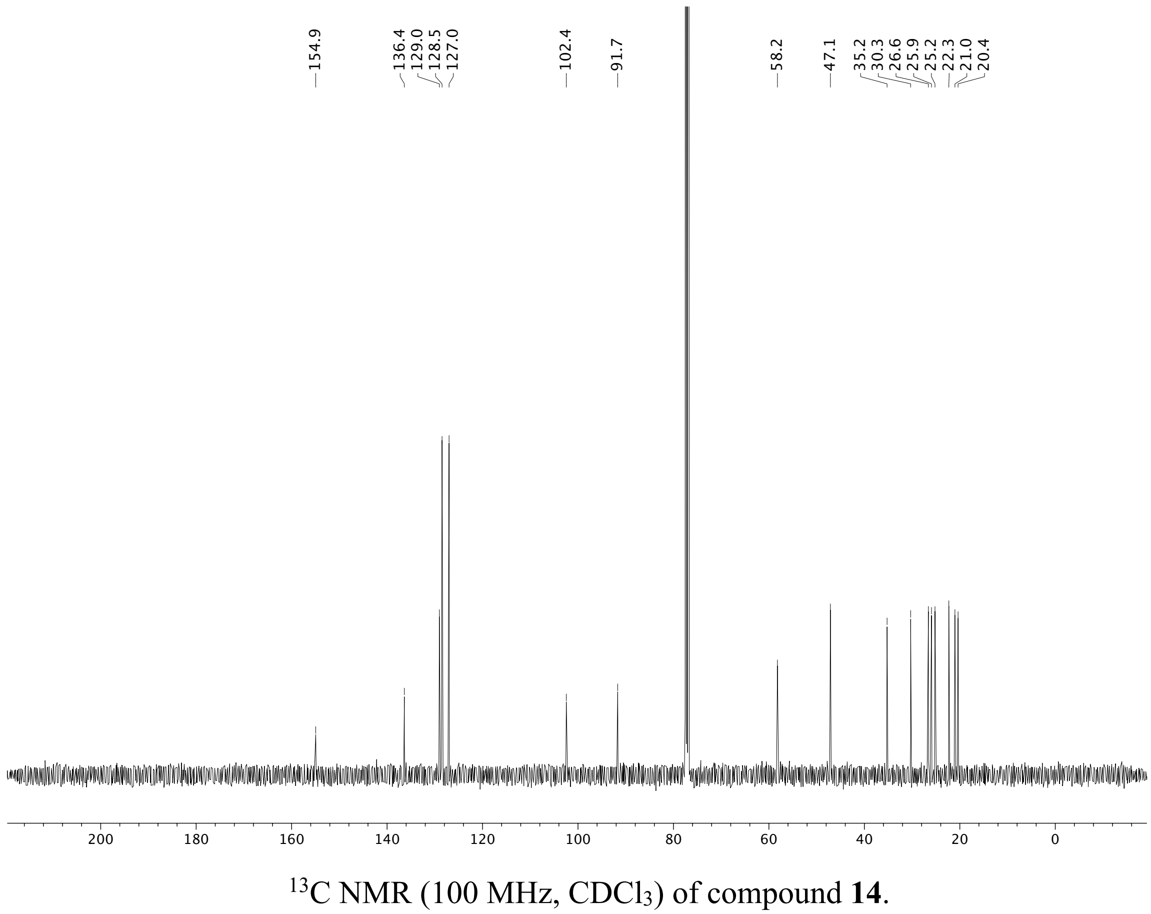

^{13}C NMR (100 MHz, $CDCl_3$) of compound **14**.

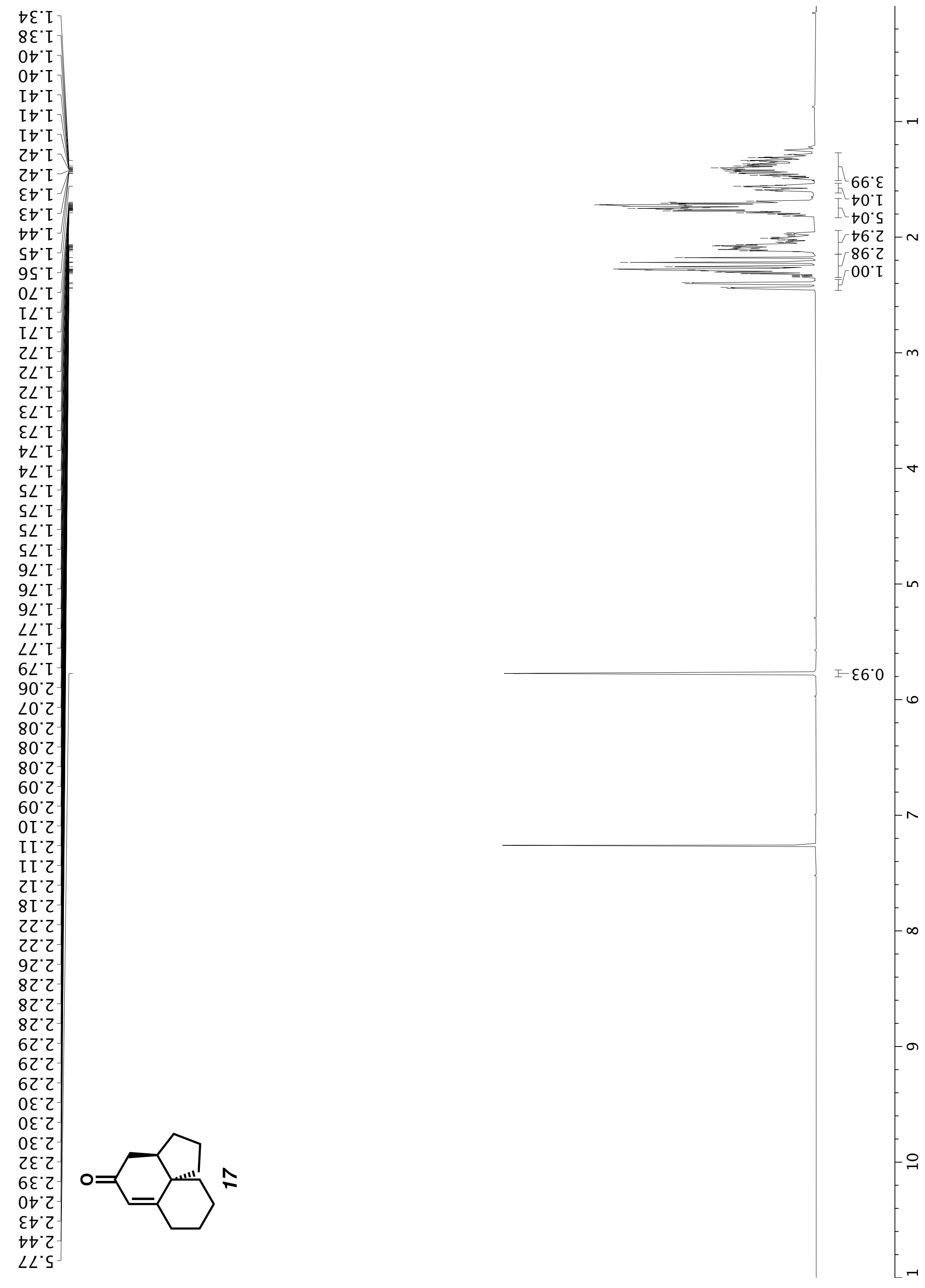

^{1}H NMR (400 MHz, $CDCl_3$) of compound **17**.

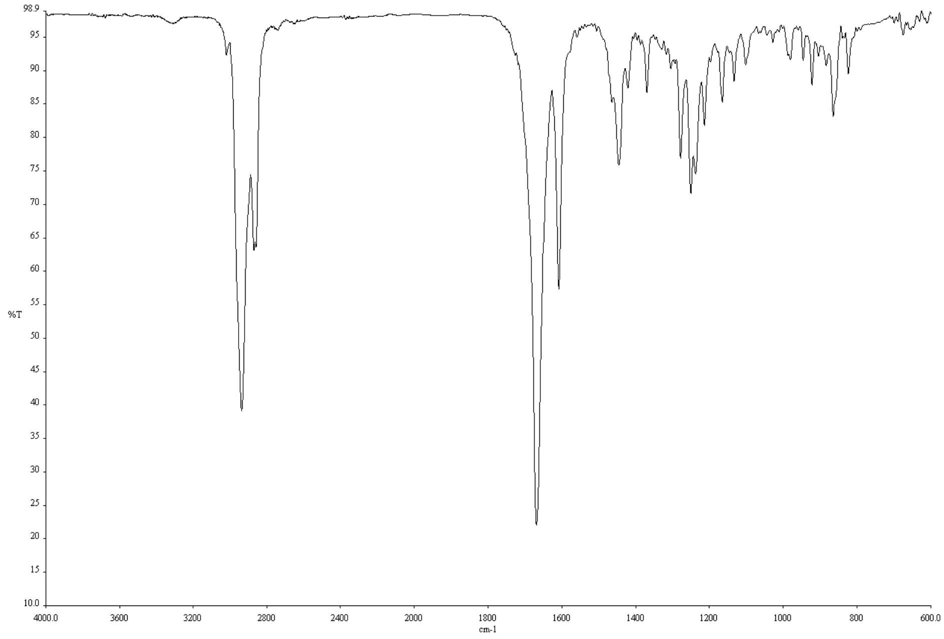

Infrared spectrum (Thin Film, NaCl) of compound **17**.

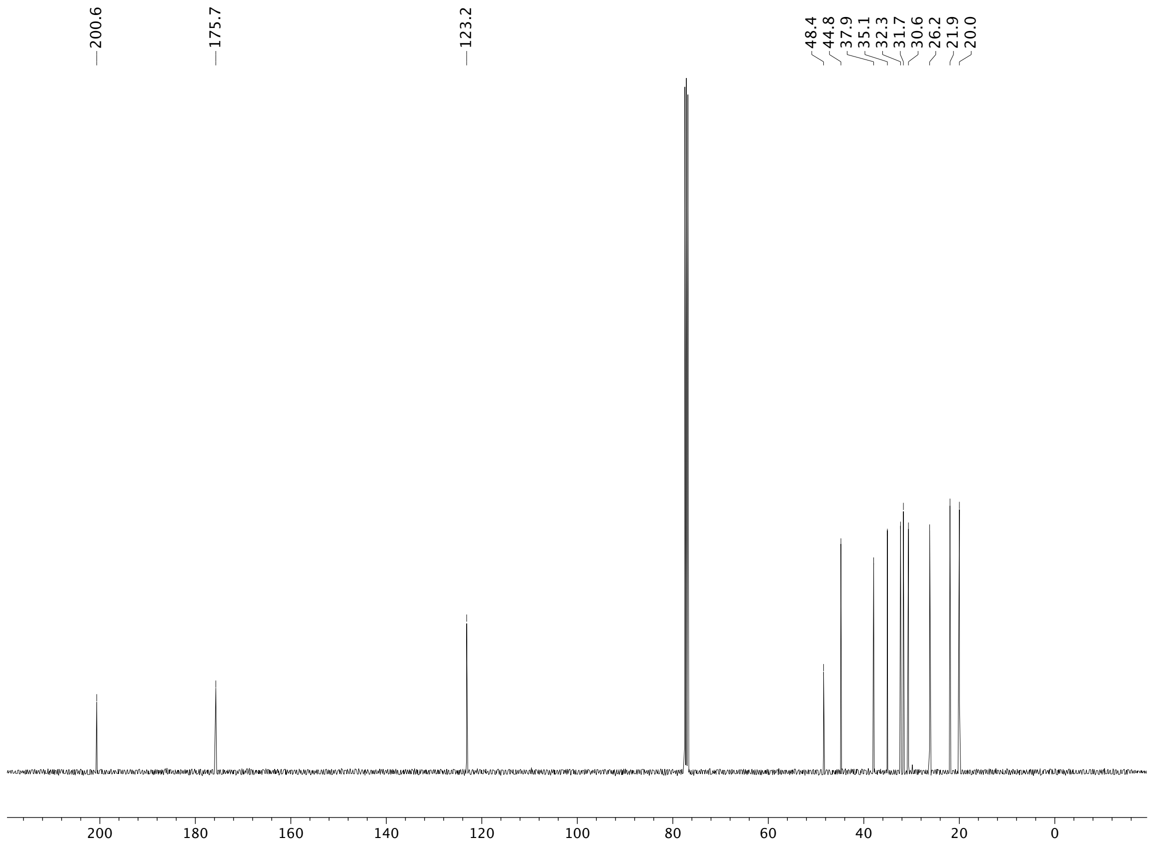

^{13}C NMR (100 MHz, $CDCl_3$) of compound **17**.

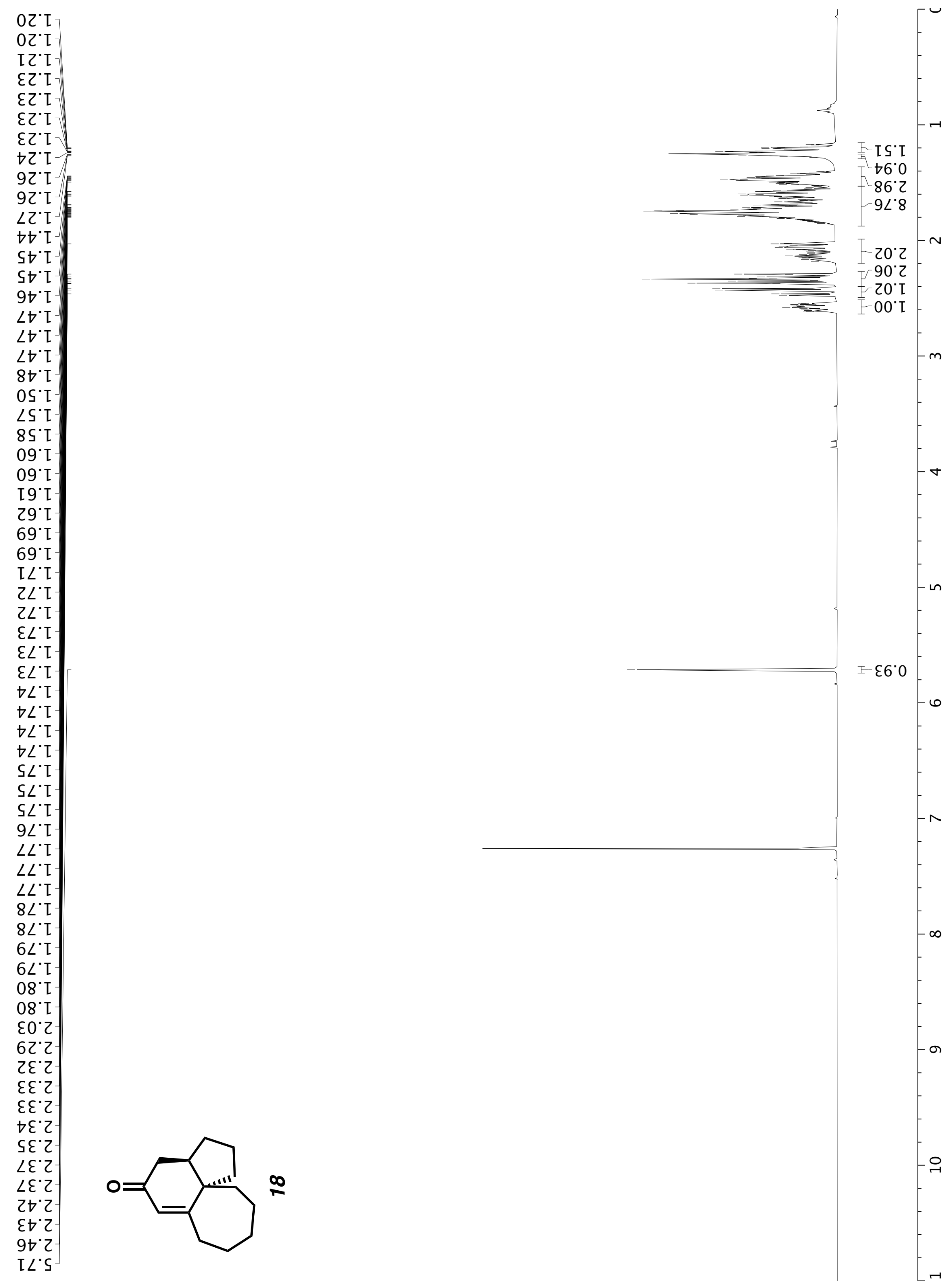

^{1}H NMR (400 MHz, $CDCl_3$) of compound **18**.

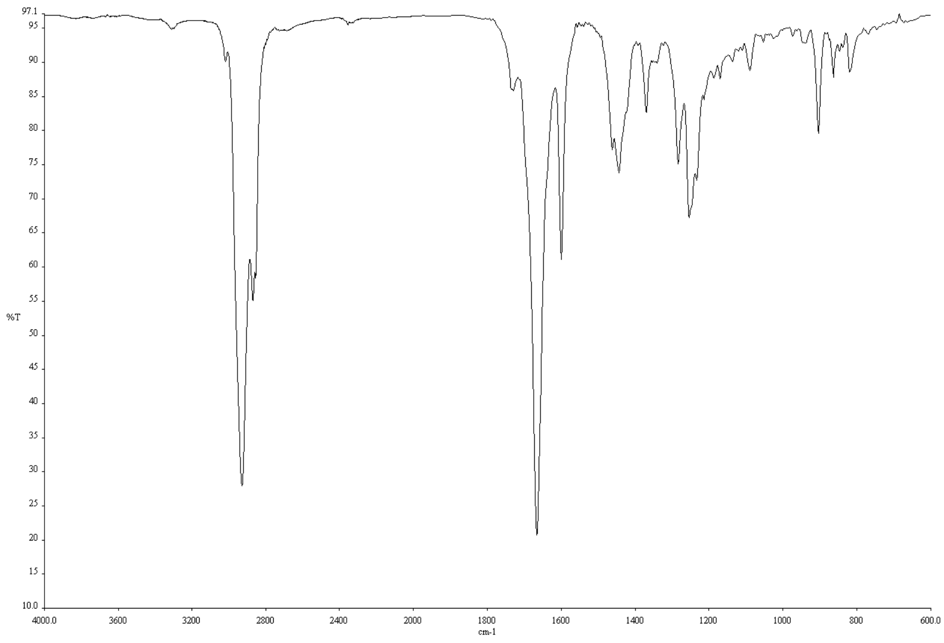

Infrared spectrum (Thin Film, NaCl) of compound **18**.

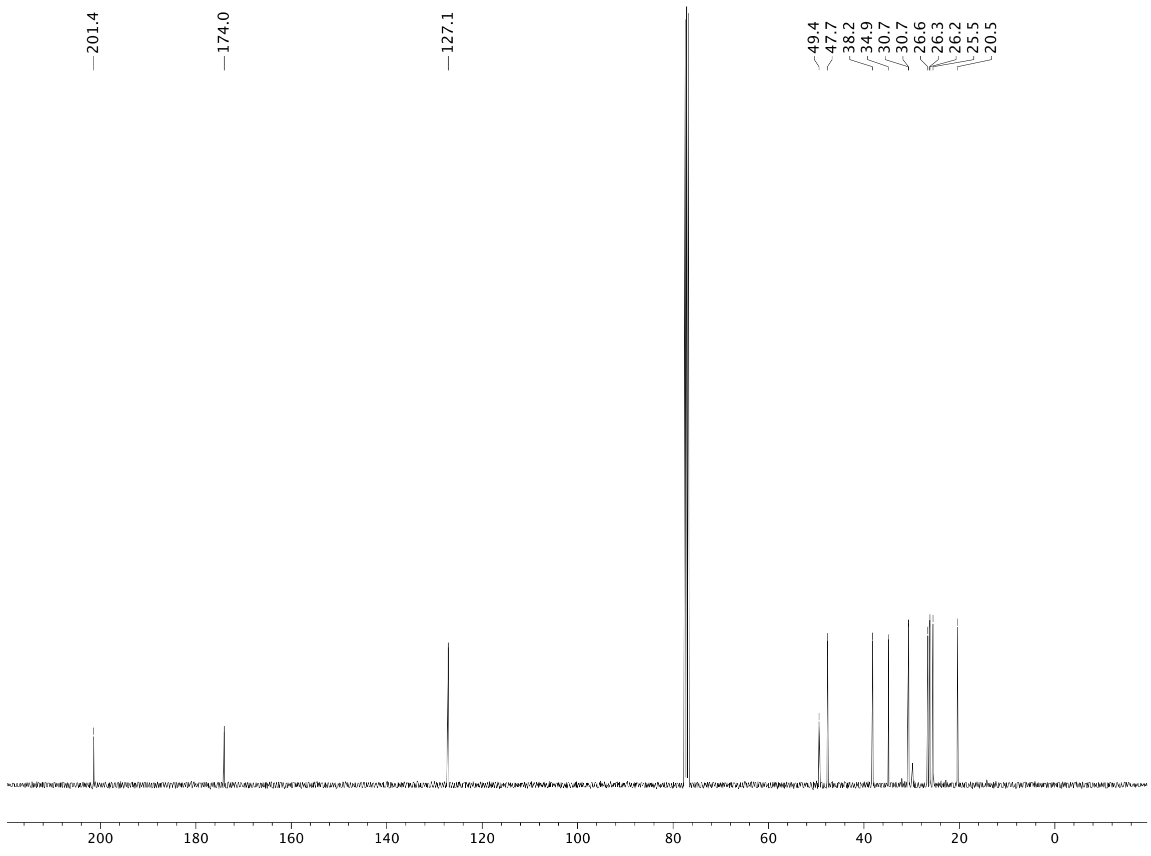

^{13}C NMR (100 MHz, $CDCl_3$) of compound **18**.

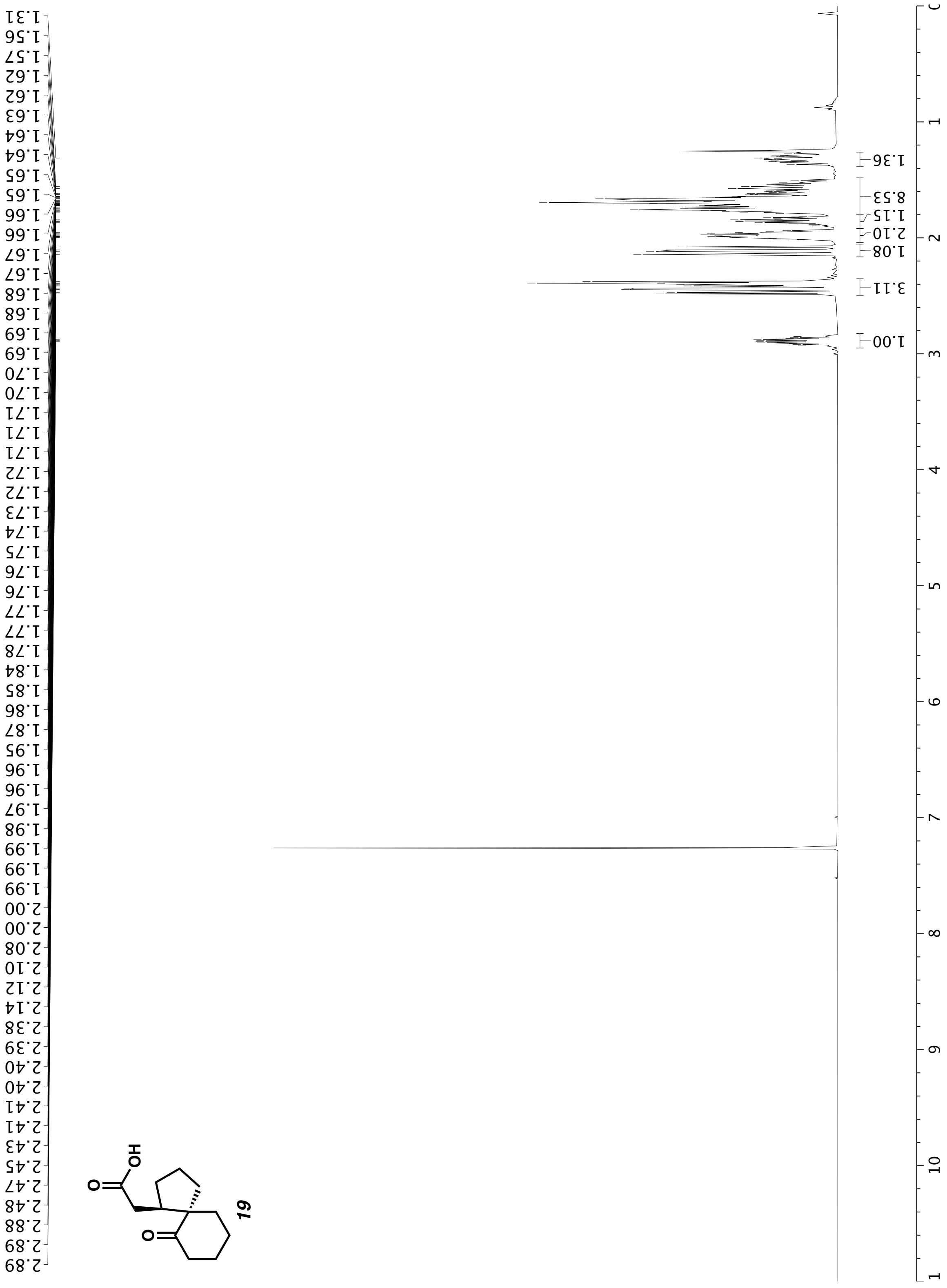

^{1}H NMR (400 MHz, $CDCl_3$) of compound **19**.

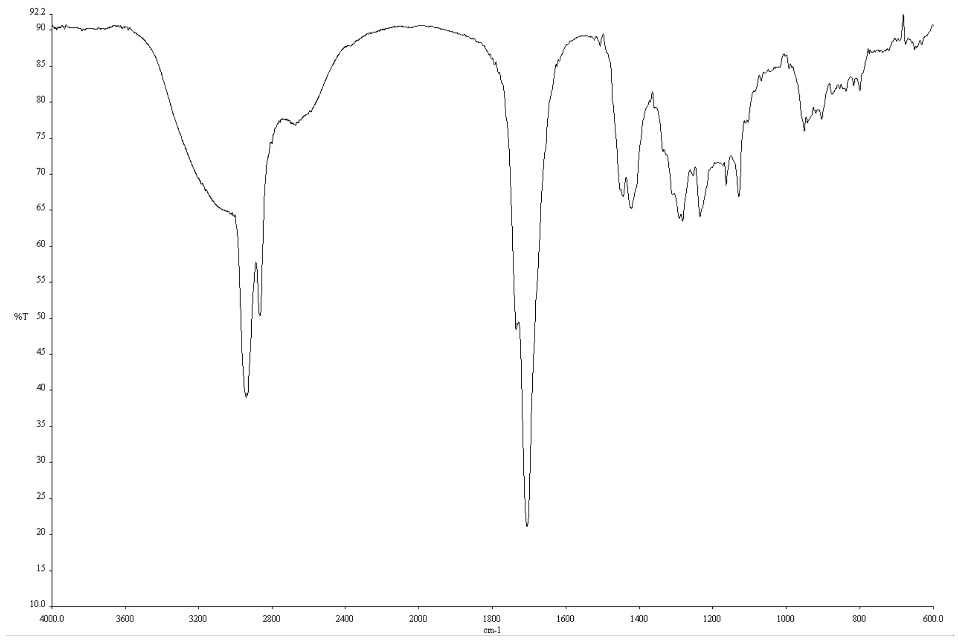

Infrared spectrum (Thin Film, NaCl) of compound **19**.

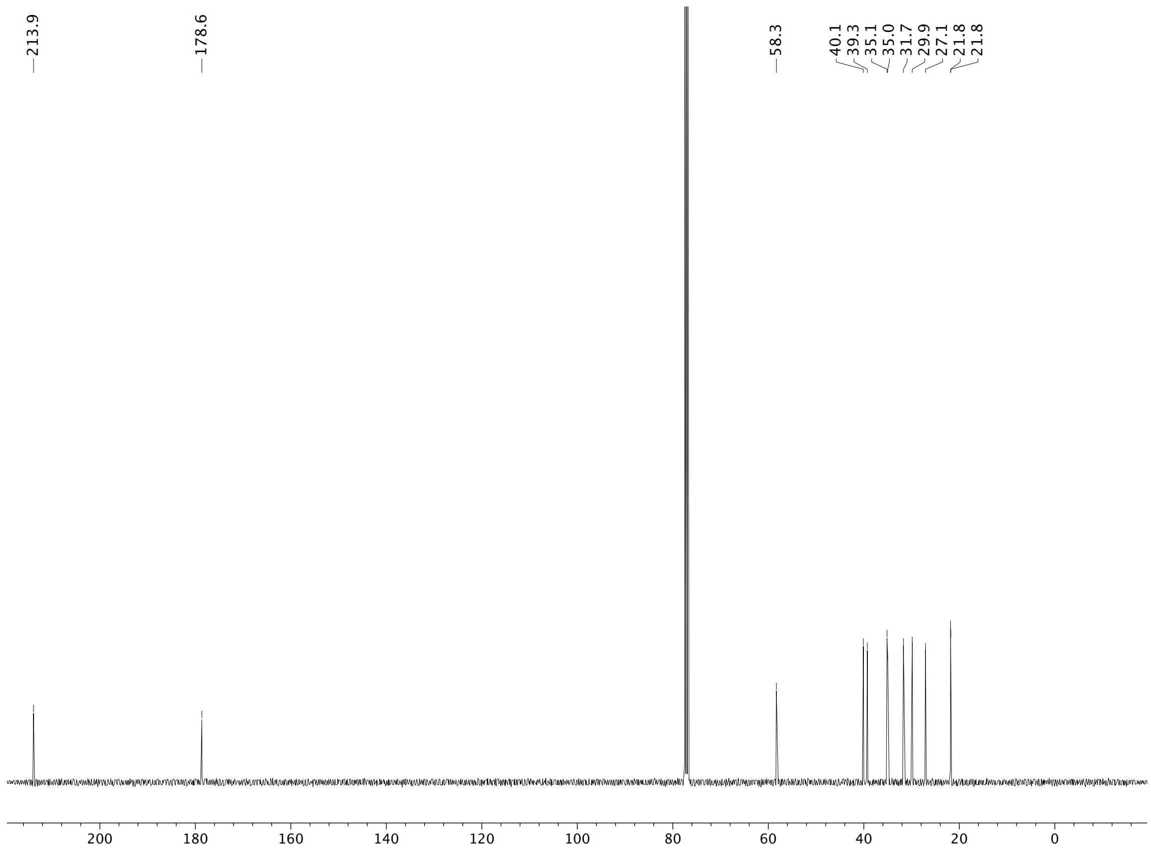

^{13}C NMR (100 MHz, $CDCl_3$) of compound **19**.

X-ray Crystal Structure Data

Crystal Structure Analysis of Product 8a (sample No.: V25038)

Spirocycle **8a** was crystalized by layering pentane onto a saturated solution of **8a** in Et_2O at 23 °C to provide crystalline needles suitable for X-ray analysis.

Compound V25038 (CCDC 2421482) crystallizes in the monoclinic space group $P2_1$ with two molecules in the asymmetric unit. Absolute configuration was assigned via Flack (Flack = 0.0(2)) and Parsons (Parsons = –0.14(9)) parameters. Bayesian Statistics further confirmed the absolute stereochemistry: P2(true) = 1.000, P3(true) = 1.000, P3(rac-twin) = 0.7×10^{-5}, and P3(false) = 0.7×10^{-17}.

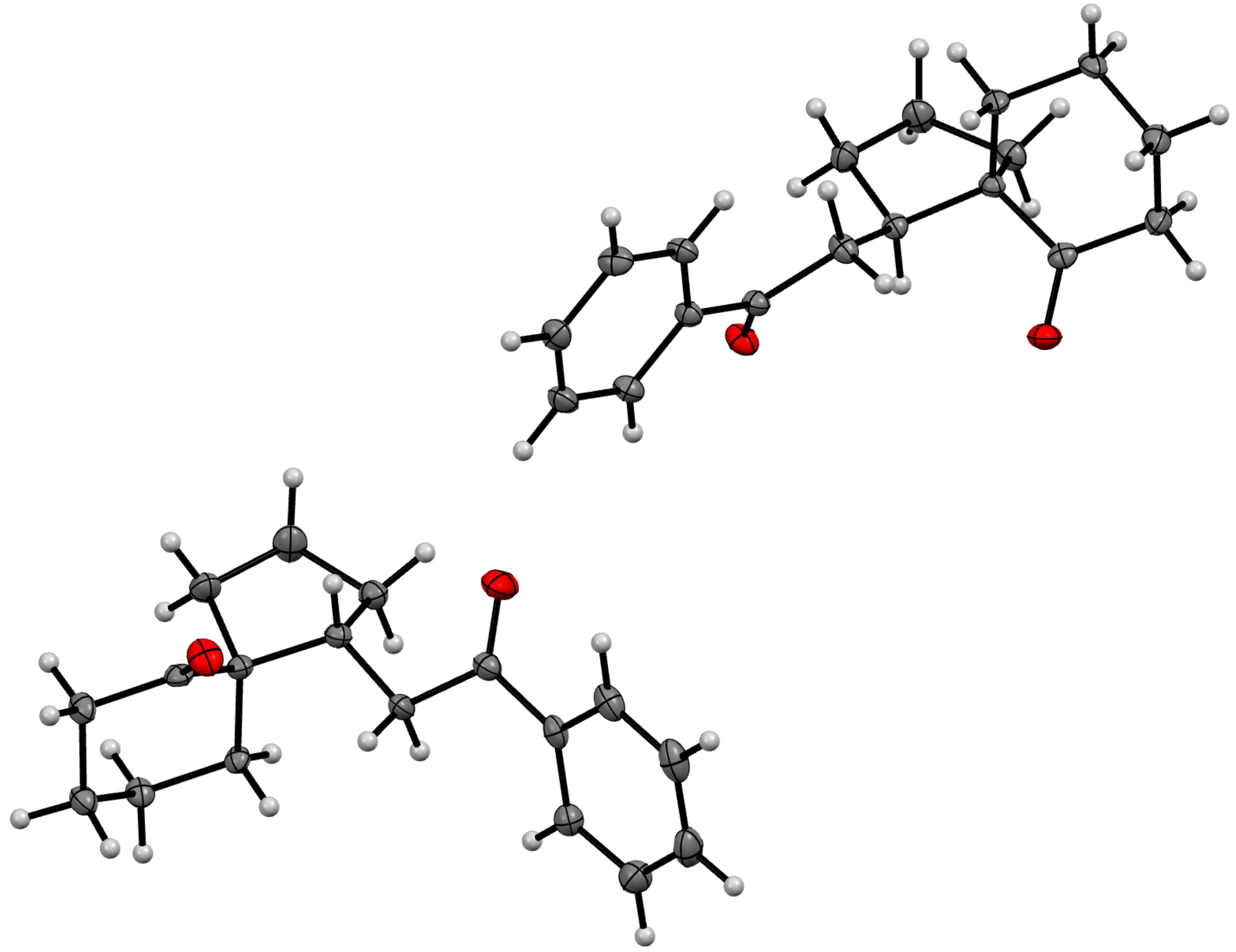

Table S1. Crystal data and structure refinement for V25038.

Identification code	V25038
Empirical formula	C18 H22 O2
Formula weight	270.35
Temperature	100(2) K
Wavelength	1.54178 Å

Crystal system	Monoclinic	
Space group	$P2_1$	
Unit cell dimensions	a = 5.9068(7) Å	a= 90°.
	b = 15.569(2) Å	b= 95.830(9)°.
	c = 15.9353(19) Å	g = 90°.
Volume	1457.9(3) $Å^3$	
Z	4	
Density (calculated)	1.232 Mg/m^3	
Absorption coefficient	0.615 mm^{-1}	
F(000)	584	
Crystal size	0.200 x 0.050 x 0.050 mm^3	
Theta range for data collection	2.787 to 74.590°.	
Index ranges	-7<=h<=7, -19<=k<=19, -19<=l<=19	
Reflections collected	37754	
Independent reflections	5928 [R(int) = 0.0777]	
Completeness to theta = 67.679°	100.0 %	
Absorption correction	Semi-empirical from equivalents	
Max. and min. transmission	0.7538 and 0.5453	
Refinement method	Full-matrix least-squares on F^2	
Data / restraints / parameters	5928 / 1 / 361	
Goodness-of-fit on F^2	1.019	
Final R indices [I>2sigma(I)]	R1 = 0.0470, wR2 = 0.1204	
R indices (all data)	R1 = 0.0502, wR2 = 0.1243	
Absolute structure parameter (Flack)	0.0(2)	
Absolute structure parameter (Parsons)	–0.14(9)	
Extinction coefficient	n/a	
Largest diff. peak and hole	0.184 and -0.225 e.$Å^{-3}$	

Table S2. Atomic coordinates (x 10^4) and equivalent isotropic displacement parameters ($Å^2$x 10^3) for V25038. U(eq) is defined as one third of the trace of the orthogonalized U^{ij} tensor.

	x	y	z	U(eq)
O(1)	8508(3)	8282(1)	9038(1)	24(1)
C(1)	7524(4)	8967(2)	8976(2)	16(1)

C(2)	8233(4)	9702(2)	9567(2)	21(1)
C(3)	6181(5)	10074(2)	9961(2)	23(1)
C(4)	4326(4)	10332(2)	9272(2)	20(1)
C(5)	3576(4)	9563(2)	8713(2)	18(1)
C(6)	5562(4)	9140(2)	8302(2)	15(1)
C(7)	6385(5)	9725(2)	7607(2)	20(1)
C(8)	4659(5)	9567(2)	6824(2)	25(1)
C(9)	3538(5)	8694(2)	6969(2)	21(1)
C(10)	4843(4)	8330(2)	7778(2)	15(1)
C(11)	3515(5)	7648(2)	8206(2)	18(1)
C(12)	3238(4)	6817(2)	7696(2)	16(1)
O(2)	4370(3)	6680(1)	7115(1)	22(1)
C(13)	1592(4)	6152(2)	7944(2)	16(1)
C(14)	1914(5)	5294(2)	7715(2)	19(1)
C(15)	380(5)	4668(2)	7906(2)	22(1)
C(16)	-1508(5)	4893(2)	8317(2)	21(1)
C(17)	-1828(5)	5738(2)	8548(2)	21(1)
C(18)	-286(4)	6368(2)	8372(2)	18(1)
O(3)	-381(3)	980(1)	5999(1)	26(1)
C(21)	291(4)	912(2)	5305(2)	18(1)
C(22)	-483(4)	180(2)	4719(2)	21(1)
C(23)	1581(5)	-318(2)	4456(2)	21(1)
C(24)	3222(5)	291(2)	4076(2)	19(1)
C(25)	3977(4)	1017(2)	4690(2)	16(1)
C(26)	1961(4)	1540(2)	4976(2)	16(1)
C(27)	743(5)	2066(2)	4224(2)	25(1)
C(28)	1758(6)	2967(2)	4286(2)	28(1)
C(29)	3510(4)	2978(2)	5068(2)	20(1)
C(30)	2717(4)	2256(2)	5618(2)	17(1)
C(31)	4459(5)	2003(2)	6348(2)	20(1)
C(32)	4685(5)	2658(2)	7058(2)	18(1)
O(4)	3478(4)	3285(1)	7058(1)	31(1)
C(33)	6420(5)	2481(2)	7794(2)	19(1)
C(34)	6013(5)	2791(2)	8591(2)	24(1)
C(35)	7596(6)	2652(2)	9280(2)	29(1)
C(36)	9609(6)	2225(2)	9190(2)	29(1)

C(37)	10029(5)	1915(2)	8401(2)	27(1)
C(38)	8432(5)	2035(2)	7707(2)	21(1)

Table S3. Bond lengths [Å] and angles [°] for V25038.

O(1)-C(1)	1.215(3)
C(1)-C(2)	1.513(4)
C(1)-C(6)	1.522(3)
C(2)-C(3)	1.534(4)
C(2)-H(2A)	0.9900
C(2)-H(2B)	0.9900
C(3)-C(4)	1.524(4)
C(3)-H(3A)	0.9900
C(3)-H(3B)	0.9900
C(4)-C(5)	1.531(4)
C(4)-H(4A)	0.9900
C(4)-H(4B)	0.9900
C(5)-C(6)	1.548(3)
C(5)-H(5A)	0.9900
C(5)-H(5B)	0.9900
C(6)-C(10)	1.548(3)
C(6)-C(7)	1.550(4)
C(7)-C(8)	1.548(4)
C(7)-H(7A)	0.9900
C(7)-H(7B)	0.9900
C(8)-C(9)	1.539(4)
C(8)-H(8A)	0.9900
C(8)-H(8B)	0.9900
C(9)-C(10)	1.541(4)
C(9)-H(9A)	0.9900
C(9)-H(9B)	0.9900
C(10)-C(11)	1.522(3)
C(10)-H(10)	1.0000
C(11)-C(12)	1.527(3)
C(11)-H(11A)	0.9900

C(11)-H(11B)	0.9900
C(12)-O(2)	1.215(3)
C(12)-C(13)	1.500(3)
C(13)-C(18)	1.401(4)
C(13)-C(14)	1.403(4)
C(14)-C(15)	1.386(4)
C(14)-H(14)	0.9500
C(15)-C(16)	1.394(4)
C(15)-H(15)	0.9500
C(16)-C(17)	1.385(4)
C(16)-H(16)	0.9500
C(17)-C(18)	1.385(4)
C(17)-H(17)	0.9500
C(18)-H(18)	0.9500
O(3)-C(21)	1.216(3)
C(21)-C(22)	1.515(4)
C(21)-C(26)	1.519(4)
C(22)-C(23)	1.539(4)
C(22)-H(22A)	0.9900
C(22)-H(22B)	0.9900
C(23)-C(24)	1.526(4)
C(23)-H(23A)	0.9900
C(23)-H(23B)	0.9900
C(24)-C(25)	1.532(4)
C(24)-H(24A)	0.9900
C(24)-H(24B)	0.9900
C(25)-C(26)	1.549(3)
C(25)-H(25A)	0.9900
C(25)-H(25B)	0.9900
C(26)-C(30)	1.548(4)
C(26)-C(27)	1.565(4)
C(27)-C(28)	1.526(4)
C(27)-H(27A)	0.9900
C(27)-H(27B)	0.9900
C(28)-C(29)	1.537(4)
C(28)-H(28A)	0.9900

C(28)-H(28B)	0.9900
C(29)-C(30)	1.528(4)
C(29)-H(29A)	0.9900
C(29)-H(29B)	0.9900
C(30)-C(31)	1.525(4)
C(30)-H(30)	1.0000
C(31)-C(32)	1.519(4)
C(31)-H(31A)	0.9900
C(31)-H(31B)	0.9900
C(32)-O(4)	1.209(4)
C(32)-C(33)	1.503(4)
C(33)-C(38)	1.395(4)
C(33)-C(34)	1.403(4)
C(34)-C(35)	1.385(5)
C(34)-H(34)	0.9500
C(35)-C(36)	1.383(5)
C(35)-H(35)	0.9500
C(36)-C(37)	1.392(4)
C(36)-H(36)	0.9500
C(37)-C(38)	1.391(4)
C(37)-H(37)	0.9500
C(38)-H(38)	0.9500

O(1)-C(1)-C(2)	120.9(2)
O(1)-C(1)-C(6)	122.4(2)
C(2)-C(1)-C(6)	116.7(2)
C(1)-C(2)-C(3)	111.1(2)
C(1)-C(2)-H(2A)	109.4
C(3)-C(2)-H(2A)	109.4
C(1)-C(2)-H(2B)	109.4
C(3)-C(2)-H(2B)	109.4
H(2A)-C(2)-H(2B)	108.0
C(4)-C(3)-C(2)	110.1(2)
C(4)-C(3)-H(3A)	109.6
C(2)-C(3)-H(3A)	109.6
C(4)-C(3)-H(3B)	109.6

C(2)-C(3)-H(3B)	109.6
H(3A)-C(3)-H(3B)	108.1
C(3)-C(4)-C(5)	111.0(2)
C(3)-C(4)-H(4A)	109.4
C(5)-C(4)-H(4A)	109.4
C(3)-C(4)-H(4B)	109.4
C(5)-C(4)-H(4B)	109.4
H(4A)-C(4)-H(4B)	108.0
C(4)-C(5)-C(6)	113.1(2)
C(4)-C(5)-H(5A)	109.0
C(6)-C(5)-H(5A)	109.0
C(4)-C(5)-H(5B)	109.0
C(6)-C(5)-H(5B)	109.0
H(5A)-C(5)-H(5B)	107.8
C(1)-C(6)-C(5)	109.4(2)
C(1)-C(6)-C(10)	112.6(2)
C(5)-C(6)-C(10)	113.4(2)
C(1)-C(6)-C(7)	109.7(2)
C(5)-C(6)-C(7)	111.0(2)
C(10)-C(6)-C(7)	100.50(19)
C(8)-C(7)-C(6)	104.7(2)
C(8)-C(7)-H(7A)	110.8
C(6)-C(7)-H(7A)	110.8
C(8)-C(7)-H(7B)	110.8
C(6)-C(7)-H(7B)	110.8
H(7A)-C(7)-H(7B)	108.9
C(9)-C(8)-C(7)	106.1(2)
C(9)-C(8)-H(8A)	110.5
C(7)-C(8)-H(8A)	110.5
C(9)-C(8)-H(8B)	110.5
C(7)-C(8)-H(8B)	110.5
H(8A)-C(8)-H(8B)	108.7
C(8)-C(9)-C(10)	105.4(2)
C(8)-C(9)-H(9A)	110.7
C(10)-C(9)-H(9A)	110.7
C(8)-C(9)-H(9B)	110.7

C(10)-C(9)-H(9B)	110.7
H(9A)-C(9)-H(9B)	108.8
C(11)-C(10)-C(9)	113.3(2)
C(11)-C(10)-C(6)	116.9(2)
C(9)-C(10)-C(6)	103.7(2)
C(11)-C(10)-H(10)	107.5
C(9)-C(10)-H(10)	107.5
C(6)-C(10)-H(10)	107.5
C(10)-C(11)-C(12)	112.6(2)
C(10)-C(11)-H(11A)	109.1
C(12)-C(11)-H(11A)	109.1
C(10)-C(11)-H(11B)	109.1
C(12)-C(11)-H(11B)	109.1
H(11A)-C(11)-H(11B)	107.8
O(2)-C(12)-C(13)	120.1(2)
O(2)-C(12)-C(11)	121.1(2)
C(13)-C(12)-C(11)	118.8(2)
C(18)-C(13)-C(14)	119.3(2)
C(18)-C(13)-C(12)	122.0(2)
C(14)-C(13)-C(12)	118.7(2)
C(15)-C(14)-C(13)	120.3(2)
C(15)-C(14)-H(14)	119.9
C(13)-C(14)-H(14)	119.9
C(14)-C(15)-C(16)	119.9(2)
C(14)-C(15)-H(15)	120.1
C(16)-C(15)-H(15)	120.1
C(17)-C(16)-C(15)	120.0(2)
C(17)-C(16)-H(16)	120.0
C(15)-C(16)-H(16)	120.0
C(16)-C(17)-C(18)	120.6(2)
C(16)-C(17)-H(17)	119.7
C(18)-C(17)-H(17)	119.7
C(17)-C(18)-C(13)	119.8(2)
C(17)-C(18)-H(18)	120.1
C(13)-C(18)-H(18)	120.1
O(3)-C(21)-C(22)	121.3(2)

O(3)-C(21)-C(26)	122.8(2)
C(22)-C(21)-C(26)	115.9(2)
C(21)-C(22)-C(23)	110.4(2)
C(21)-C(22)-H(22A)	109.6
C(23)-C(22)-H(22A)	109.6
C(21)-C(22)-H(22B)	109.6
C(23)-C(22)-H(22B)	109.6
H(22A)-C(22)-H(22B)	108.1
C(24)-C(23)-C(22)	110.4(2)
C(24)-C(23)-H(23A)	109.6
C(22)-C(23)-H(23A)	109.6
C(24)-C(23)-H(23B)	109.6
C(22)-C(23)-H(23B)	109.6
H(23A)-C(23)-H(23B)	108.1
C(23)-C(24)-C(25)	111.1(2)
C(23)-C(24)-H(24A)	109.4
C(25)-C(24)-H(24A)	109.4
C(23)-C(24)-H(24B)	109.4
C(25)-C(24)-H(24B)	109.4
H(24A)-C(24)-H(24B)	108.0
C(24)-C(25)-C(26)	113.2(2)
C(24)-C(25)-H(25A)	108.9
C(26)-C(25)-H(25A)	108.9
C(24)-C(25)-H(25B)	108.9
C(26)-C(25)-H(25B)	108.9
H(25A)-C(25)-H(25B)	107.8
C(21)-C(26)-C(30)	112.9(2)
C(21)-C(26)-C(25)	108.0(2)
C(30)-C(26)-C(25)	113.2(2)
C(21)-C(26)-C(27)	109.7(2)
C(30)-C(26)-C(27)	102.0(2)
C(25)-C(26)-C(27)	110.9(2)
C(28)-C(27)-C(26)	106.6(2)
C(28)-C(27)-H(27A)	110.4
C(26)-C(27)-H(27A)	110.4
C(28)-C(27)-H(27B)	110.4

C(26)-C(27)-H(27B)	110.4
H(27A)-C(27)-H(27B)	108.6
C(27)-C(28)-C(29)	106.8(2)
C(27)-C(28)-H(28A)	110.4
C(29)-C(28)-H(28A)	110.4
C(27)-C(28)-H(28B)	110.4
C(29)-C(28)-H(28B)	110.4
H(28A)-C(28)-H(28B)	108.6
C(30)-C(29)-C(28)	103.7(2)
C(30)-C(29)-H(29A)	111.0
C(28)-C(29)-H(29A)	111.0
C(30)-C(29)-H(29B)	111.0
C(28)-C(29)-H(29B)	111.0
H(29A)-C(29)-H(29B)	109.0
C(31)-C(30)-C(29)	113.9(2)
C(31)-C(30)-C(26)	116.4(2)
C(29)-C(30)-C(26)	103.6(2)
C(31)-C(30)-H(30)	107.5
C(29)-C(30)-H(30)	107.5
C(26)-C(30)-H(30)	107.5
C(32)-C(31)-C(30)	113.3(2)
C(32)-C(31)-H(31A)	108.9
C(30)-C(31)-H(31A)	108.9
C(32)-C(31)-H(31B)	108.9
C(30)-C(31)-H(31B)	108.9
H(31A)-C(31)-H(31B)	107.7
O(4)-C(32)-C(33)	120.2(2)
O(4)-C(32)-C(31)	122.4(3)
C(33)-C(32)-C(31)	117.3(2)
C(38)-C(33)-C(34)	119.2(3)
C(38)-C(33)-C(32)	122.3(2)
C(34)-C(33)-C(32)	118.5(3)
C(35)-C(34)-C(33)	120.0(3)
C(35)-C(34)-H(34)	120.0
C(33)-C(34)-H(34)	120.0
C(36)-C(35)-C(34)	120.8(3)

C(36)-C(35)-H(35)	119.6
C(34)-C(35)-H(35)	119.6
C(35)-C(36)-C(37)	119.7(3)
C(35)-C(36)-H(36)	120.2
C(37)-C(36)-H(36)	120.2
C(38)-C(37)-C(36)	120.2(3)
C(38)-C(37)-H(37)	119.9
C(36)-C(37)-H(37)	119.9
C(37)-C(38)-C(33)	120.2(3)
C(37)-C(38)-H(38)	119.9
C(33)-C(38)-H(38)	119.9

Symmetry transformations used to generate equivalent atoms:

Table S4. Anisotropic displacement parameters ($Å^2$x 10^3) for V25038. The anisotropic displacement factor exponent takes the form: $-2p^2[h^2 a^{*2}U^{11} + ... + 2 h k a^* b^* U^{12}]$

	U^{11}	U^{22}	U^{33}	U^{23}	U^{13}	U^{12}
O(1)	23(1)	22(1)	26(1)	-2(1)	0(1)	9(1)
C(1)	13(1)	20(1)	17(1)	1(1)	5(1)	1(1)
C(2)	16(1)	24(1)	23(1)	-7(1)	0(1)	1(1)
C(3)	18(1)	25(1)	24(1)	-9(1)	3(1)	1(1)
C(4)	16(1)	16(1)	28(1)	-6(1)	6(1)	2(1)
C(5)	13(1)	19(1)	23(1)	-3(1)	3(1)	2(1)
C(6)	13(1)	13(1)	18(1)	-1(1)	4(1)	0(1)
C(7)	22(1)	18(1)	22(1)	2(1)	5(1)	-2(1)
C(8)	33(2)	21(1)	20(1)	3(1)	-1(1)	-1(1)
C(9)	22(1)	21(1)	19(1)	-1(1)	-2(1)	-1(1)
C(10)	14(1)	16(1)	17(1)	-1(1)	4(1)	-1(1)
C(11)	19(1)	16(1)	21(1)	-2(1)	9(1)	-2(1)
C(12)	16(1)	14(1)	18(1)	0(1)	3(1)	1(1)
O(2)	23(1)	20(1)	23(1)	-4(1)	11(1)	-1(1)
C(13)	17(1)	14(1)	17(1)	1(1)	3(1)	0(1)
C(14)	21(1)	17(1)	20(1)	-1(1)	4(1)	2(1)
C(15)	25(1)	14(1)	25(1)	0(1)	2(1)	2(1)

C(16)	22(1)	17(1)	25(1)	5(1)	2(1)	-4(1)
C(17)	19(1)	22(1)	24(1)	4(1)	8(1)	1(1)
C(18)	20(1)	13(1)	21(1)	1(1)	5(1)	2(1)
O(3)	25(1)	30(1)	27(1)	-5(1)	14(1)	-3(1)
C(21)	11(1)	17(1)	24(1)	-1(1)	4(1)	3(1)
C(22)	16(1)	20(1)	28(1)	-4(1)	6(1)	-4(1)
C(23)	20(1)	18(1)	26(1)	-5(1)	5(1)	-1(1)
C(24)	17(1)	21(1)	20(1)	-4(1)	6(1)	-1(1)
C(25)	12(1)	18(1)	18(1)	-2(1)	3(1)	-1(1)
C(26)	13(1)	15(1)	20(1)	0(1)	1(1)	2(1)
C(27)	24(1)	21(1)	29(2)	4(1)	-6(1)	1(1)
C(28)	34(2)	25(2)	25(1)	7(1)	-3(1)	-4(1)
C(29)	18(1)	17(1)	26(1)	1(1)	2(1)	-1(1)
C(30)	15(1)	15(1)	20(1)	-1(1)	2(1)	1(1)
C(31)	22(1)	15(1)	21(1)	-4(1)	-3(1)	4(1)
C(32)	19(1)	16(1)	21(1)	-1(1)	4(1)	1(1)
O(4)	33(1)	28(1)	31(1)	-10(1)	0(1)	12(1)
C(33)	24(1)	15(1)	19(1)	-2(1)	3(1)	-6(1)
C(34)	29(1)	19(1)	23(1)	-5(1)	9(1)	-5(1)
C(35)	40(2)	25(2)	20(1)	-4(1)	4(1)	-10(1)
C(36)	36(2)	26(1)	22(1)	-1(1)	-6(1)	-6(1)
C(37)	26(1)	25(1)	29(2)	1(1)	-1(1)	0(1)
C(38)	22(1)	20(1)	21(1)	-1(1)	2(1)	-2(1)

Table S5. Hydrogen coordinates (x 10^4) and isotropic displacement parameters (Å^{2}x 10 ^{3})
for V25038.

	x	y	z	U(eq)
H(2A)	8949	10159	9252	25
H(2B)	9374	9495	10020	25

H(3A)	5577	9641	10335	27
H(3B)	6663	10583	10307	27
H(4A)	4905	10792	8921	24
H(4B)	3001	10562	9534	24
H(5A)	2406	9755	8263	22
H(5B)	2868	9129	9058	22
H(7A)	6378	10336	7779	24
H(7B)	7944	9566	7488	24
H(8A)	3500	10029	6768	30
H(8B)	5446	9552	6305	30
H(9A)	3673	8306	6485	25
H(9B)	1906	8769	7044	25
H(10)	6261	8053	7613	19
H(11A)	1991	7876	8290	22
H(11B)	4314	7520	8768	22
H(14)	3189	5142	7428	23
H(15)	614	4086	7757	26
H(16)	-2576	4466	8439	26
H(17)	-3116	5888	8829	26
H(18)	-502	6945	8543	22
H(22A)	-1384	410	4211	25
H(22B)	-1470	-213	5007	25
H(23A)	2374	-609	4955	25
H(23B)	1058	-764	4038	25
H(24A)	2469	539	3547	23
H(24B)	4574	-34	3935	23
H(25A)	4995	1410	4416	19
H(25B)	4861	769	5193	19
H(27A)	1008	1797	3680	30
H(27B)	-917	2088	4266	30
H(28A)	552	3398	4344	34
H(28B)	2503	3103	3773	34
H(29A)	5062	2864	4911	24
H(29B)	3503	3537	5362	24
H(30)	1328	2464	5865	20
H(31A)	4013	1443	6576	24

H(31B)	5960	1927	6132	24
H(34)	4651	3097	8659	28
H(35)	7296	2853	9821	34
H(36)	10699	2143	9664	34
H(37)	11410	1621	8336	32
H(38)	8713	1813	7172	26

Table S6. Torsion angles [°] for V25038.

O(1)-C(1)-C(2)-C(3)	-129.1(3)
C(6)-C(1)-C(2)-C(3)	51.8(3)
C(1)-C(2)-C(3)-C(4)	-54.6(3)
C(2)-C(3)-C(4)-C(5)	58.1(3)
C(3)-C(4)-C(5)-C(6)	-57.0(3)
O(1)-C(1)-C(6)-C(5)	132.6(3)
C(2)-C(1)-C(6)-C(5)	-48.3(3)
O(1)-C(1)-C(6)-C(10)	5.6(3)
C(2)-C(1)-C(6)-C(10)	-175.4(2)
O(1)-C(1)-C(6)-C(7)	-105.5(3)
C(2)-C(1)-C(6)-C(7)	73.6(3)
C(4)-C(5)-C(6)-C(1)	50.0(3)
C(4)-C(5)-C(6)-C(10)	176.6(2)
C(4)-C(5)-C(6)-C(7)	-71.1(3)
C(1)-C(6)-C(7)-C(8)	158.5(2)
C(5)-C(6)-C(7)-C(8)	-80.6(3)
C(10)-C(6)-C(7)-C(8)	39.7(2)
C(6)-C(7)-C(8)-C(9)	-21.0(3)
C(7)-C(8)-C(9)-C(10)	-6.3(3)
C(8)-C(9)-C(10)-C(11)	159.1(2)
C(8)-C(9)-C(10)-C(6)	31.4(3)
C(1)-C(6)-C(10)-C(11)	74.2(3)
C(5)-C(6)-C(10)-C(11)	-50.6(3)
C(7)-C(6)-C(10)-C(11)	-169.1(2)
C(1)-C(6)-C(10)-C(9)	-160.3(2)
C(5)-C(6)-C(10)-C(9)	74.8(3)

C(7)-C(6)-C(10)-C(9)	-43.7(2)
C(9)-C(10)-C(11)-C(12)	69.1(3)
C(6)-C(10)-C(11)-C(12)	-170.3(2)
C(10)-C(11)-C(12)-O(2)	14.1(4)
C(10)-C(11)-C(12)-C(13)	-168.0(2)
O(2)-C(12)-C(13)-C(18)	-156.3(3)
C(11)-C(12)-C(13)-C(18)	25.7(4)
O(2)-C(12)-C(13)-C(14)	21.7(4)
C(11)-C(12)-C(13)-C(14)	-156.2(2)
C(18)-C(13)-C(14)-C(15)	0.5(4)
C(12)-C(13)-C(14)-C(15)	-177.6(2)
C(13)-C(14)-C(15)-C(16)	0.8(4)
C(14)-C(15)-C(16)-C(17)	-1.2(4)
C(15)-C(16)-C(17)-C(18)	0.1(4)
C(16)-C(17)-C(18)-C(13)	1.2(4)
C(14)-C(13)-C(18)-C(17)	-1.5(4)
C(12)-C(13)-C(18)-C(17)	176.5(2)
O(3)-C(21)-C(22)-C(23)	-124.2(3)
C(26)-C(21)-C(22)-C(23)	55.0(3)
C(21)-C(22)-C(23)-C(24)	-54.6(3)
C(22)-C(23)-C(24)-C(25)	56.4(3)
C(23)-C(24)-C(25)-C(26)	-56.7(3)
O(3)-C(21)-C(26)-C(30)	0.7(4)
C(22)-C(21)-C(26)-C(30)	-178.6(2)
O(3)-C(21)-C(26)-C(25)	126.6(3)
C(22)-C(21)-C(26)-C(25)	-52.6(3)
O(3)-C(21)-C(26)-C(27)	-112.4(3)
C(22)-C(21)-C(26)-C(27)	68.4(3)
C(24)-C(25)-C(26)-C(21)	52.5(3)
C(24)-C(25)-C(26)-C(30)	178.2(2)
C(24)-C(25)-C(26)-C(27)	-67.7(3)
C(21)-C(26)-C(27)-C(28)	144.9(2)
C(30)-C(26)-C(27)-C(28)	25.0(3)
C(25)-C(26)-C(27)-C(28)	-95.9(3)
C(26)-C(27)-C(28)-C(29)	-1.0(3)
C(27)-C(28)-C(29)-C(30)	-23.9(3)

C(28)-C(29)-C(30)-C(31)	167.4(2)
C(28)-C(29)-C(30)-C(26)	39.9(3)
C(21)-C(26)-C(30)-C(31)	76.6(3)
C(25)-C(26)-C(30)-C(31)	-46.6(3)
C(27)-C(26)-C(30)-C(31)	-165.8(2)
C(21)-C(26)-C(30)-C(29)	-157.5(2)
C(25)-C(26)-C(30)-C(29)	79.4(3)
C(27)-C(26)-C(30)-C(29)	-39.9(2)
C(29)-C(30)-C(31)-C(32)	73.7(3)
C(26)-C(30)-C(31)-C(32)	-165.8(2)
C(30)-C(31)-C(32)-O(4)	4.7(4)
C(30)-C(31)-C(32)-C(33)	-177.9(2)
O(4)-C(32)-C(33)-C(38)	-151.3(3)
C(31)-C(32)-C(33)-C(38)	31.3(4)
O(4)-C(32)-C(33)-C(34)	27.3(4)
C(31)-C(32)-C(33)-C(34)	-150.1(3)
C(38)-C(33)-C(34)-C(35)	-0.2(4)
C(32)-C(33)-C(34)-C(35)	-178.8(2)
C(33)-C(34)-C(35)-C(36)	1.4(4)
C(34)-C(35)-C(36)-C(37)	-1.3(5)
C(35)-C(36)-C(37)-C(38)	0.0(5)
C(36)-C(37)-C(38)-C(33)	1.2(4)
C(34)-C(33)-C(38)-C(37)	-1.1(4)
C(32)-C(33)-C(38)-C(37)	177.5(3)

Symmetry transformations used to generate equivalent atoms:

Crystal Structure Analysis of Product 8b (sample No.: V25125)

Spirocycle **8b** was crystalized by layering pentane onto a saturated solution of **8b** in diethyl ether at 23 °C to provide crystalline blocks suitable for X-ray analysis.

Compound V25125 (CCDC 2444207) crystallizes in the orthorhombic space group $P2_12_12_1$ with one molecule in the asymmetric unit. One carbon atom and the associated hydrogen atoms were disordered over two positions. Absolute configuration was assigned via Flack (Flack = -0.06(5)) parameter.

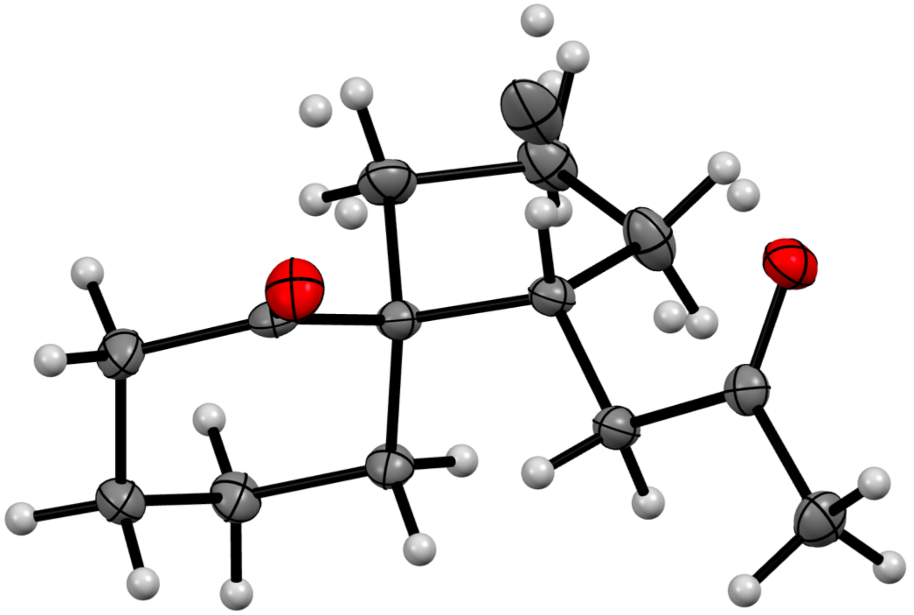

Table S7. Crystal data and structure refinement for V25125.

Identification code	V25125	
Empirical formula	C13 H20 O2	
Formula weight	208.29	
Temperature	100(2) K	
Wavelength	1.54178 Å	
Crystal system	Orthorhombic	
Space group	$P2_12_12_1$	
Unit cell dimensions	a = 6.0311(6) Å	a= 90°.
	b = 13.5742(13) Å	b= 90°.
	c = 14.2929(13) Å	g = 90°.
Volume	1170.12(19) Å^3	
Z	4	
Density (calculated)	1.182 Mg/m^3	

Absorption coefficient	0.612 mm^{-1}
F(000)	456
Crystal size	0.400 x 0.150 x 0.100 mm^3
Theta range for data collection	4.492 to 74.468°.
Index ranges	-7<=h<=7, -16<=k<=16, -17<=l<=17
Reflections collected	17358
Independent reflections	2402 [R(int) = 0.0414]
Completeness to theta = 67.679°	100.0 %
Absorption correction	Semi-empirical from equivalents
Max. and min. transmission	0.7538 and 0.6398
Refinement method	Full-matrix least-squares on F^2
Data / restraints / parameters	2402 / 47 / 147
Goodness-of-fit on F^2	1.051
Final R indices [I>2sigma(I)]	R1 = 0.0287, wR2 = 0.0751
R indices (all data)	R1 = 0.0296, wR2 = 0.0753
Absolute structure parameter	-0.06(5)
Extinction coefficient	n/a
Largest diff. peak and hole	0.158 and -0.155 e.$Å^{-3}$

Table S8. Atomic coordinates (x 10^4) and equivalent isotropic displacement parameters ($Å^2$x 10^3) for V25125. U(eq) is defined as one third of the trace of the orthogonalized U^{ij} tensor.

	x	y	z	U(eq)
C(1)	7886(2)	6404(1)	2188(1)	19(1)
O(1)	8477(2)	6891(1)	2857(1)	29(1)
C(2)	8676(2)	6636(1)	1208(1)	24(1)
C(3)	6709(2)	6745(1)	534(1)	22(1)
C(4)	5266(3)	5827(1)	562(1)	22(1)
C(5)	4436(2)	5629(1)	1557(1)	19(1)
C(6)	6323(2)	5527(1)	2284(1)	17(1)
C(7)	5457(2)	5403(1)	3294(1)	17(1)
C(11)	3665(2)	6123(1)	3598(1)	19(1)
C(12)	3251(2)	6139(1)	4640(1)	19(1)
O(2)	4340(2)	5656(1)	5185(1)	25(1)
C(13)	1393(3)	6799(1)	4967(1)	26(1)
C(8)	4755(3)	4316(1)	3349(1)	28(1)
C(9)	6153(8)	3759(2)	2645(2)	27(1)
C(9A)	7050(30)	3850(8)	2883(10)	36(3)
C(10)	7624(3)	4548(1)	2147(1)	26(1)

Table S9. Bond lengths [Å] and angles [°] for V25125.

C(1)-O(1)	1.2155(18)
C(1)-C(2)	1.5130(19)
C(1)-C(6)	1.5243(18)
C(2)-C(3)	1.535(2)
C(2)-H(2A)	0.9900
C(2)-H(2B)	0.9900
C(3)-C(4)	1.522(2)
C(3)-H(3A)	0.9900
C(3)-H(3B)	0.9900
C(4)-C(5)	1.5322(19)
C(4)-H(4A)	0.9900
C(4)-H(4B)	0.9900
C(5)-C(6)	1.5477(17)
C(5)-H(5A)	0.9900
C(5)-H(5B)	0.9900
C(6)-C(7)	1.5436(17)
C(6)-C(10)	1.5549(18)
C(7)-C(11)	1.5199(18)
C(7)-C(8)	1.5378(18)
C(7)-H(7)	1.0000
C(11)-C(12)	1.5110(18)
C(11)-H(11A)	0.9900
C(11)-H(11B)	0.9900
C(12)-O(2)	1.2119(18)
C(12)-C(13)	1.5082(19)
C(13)-H(13A)	0.9800
C(13)-H(13B)	0.9800
C(13)-H(13C)	0.9800
C(8)-C(9)	1.515(3)
C(8)-C(9A)	1.661(11)
C(8)-H(8A)	0.9900
C(8)-H(8B)	0.9900
C(8)-H(8C)	0.9900
C(8)-H(8D)	0.9900

C(9)-C(10)	1.563(3)
C(9)-H(9A)	0.9900
C(9)-H(9B)	0.9900
C(9A)-C(10)	1.458(9)
C(9A)-H(9A1)	0.9900
C(9A)-H(9A2)	0.9900
C(10)-H(10A)	0.9900
C(10)-H(10B)	0.9900
C(10)-H(10C)	0.9900
C(10)-H(10D)	0.9900

O(1)-C(1)-C(2)	121.42(12)
O(1)-C(1)-C(6)	122.41(13)
C(2)-C(1)-C(6)	116.17(11)
C(1)-C(2)-C(3)	110.95(12)
C(1)-C(2)-H(2A)	109.4
C(3)-C(2)-H(2A)	109.4
C(1)-C(2)-H(2B)	109.4
C(3)-C(2)-H(2B)	109.4
H(2A)-C(2)-H(2B)	108.0
C(4)-C(3)-C(2)	110.28(12)
C(4)-C(3)-H(3A)	109.6
C(2)-C(3)-H(3A)	109.6
C(4)-C(3)-H(3B)	109.6
C(2)-C(3)-H(3B)	109.6
H(3A)-C(3)-H(3B)	108.1
C(3)-C(4)-C(5)	110.73(11)
C(3)-C(4)-H(4A)	109.5
C(5)-C(4)-H(4A)	109.5
C(3)-C(4)-H(4B)	109.5
C(5)-C(4)-H(4B)	109.5
H(4A)-C(4)-H(4B)	108.1
C(4)-C(5)-C(6)	113.53(11)
C(4)-C(5)-H(5A)	108.9
C(6)-C(5)-H(5A)	108.9
C(4)-C(5)-H(5B)	108.9

C(6)-C(5)-H(5B)	108.9
H(5A)-C(5)-H(5B)	107.7
C(1)-C(6)-C(7)	112.23(11)
C(1)-C(6)-C(5)	108.90(11)
C(7)-C(6)-C(5)	112.86(11)
C(1)-C(6)-C(10)	110.09(11)
C(7)-C(6)-C(10)	101.32(11)
C(5)-C(6)-C(10)	111.29(11)
C(11)-C(7)-C(8)	114.01(12)
C(11)-C(7)-C(6)	115.98(11)
C(8)-C(7)-C(6)	104.21(11)
C(11)-C(7)-H(7)	107.4
C(8)-C(7)-H(7)	107.4
C(6)-C(7)-H(7)	107.4
C(12)-C(11)-C(7)	114.14(11)
C(12)-C(11)-H(11A)	108.7
C(7)-C(11)-H(11A)	108.7
C(12)-C(11)-H(11B)	108.7
C(7)-C(11)-H(11B)	108.7
H(11A)-C(11)-H(11B)	107.6
O(2)-C(12)-C(13)	121.69(13)
O(2)-C(12)-C(11)	122.42(13)
C(13)-C(12)-C(11)	115.90(12)
C(12)-C(13)-H(13A)	109.5
C(12)-C(13)-H(13B)	109.5
H(13A)-C(13)-H(13B)	109.5
C(12)-C(13)-H(13C)	109.5
H(13A)-C(13)-H(13C)	109.5
H(13B)-C(13)-H(13C)	109.5
C(9)-C(8)-C(7)	106.95(16)
C(7)-C(8)-C(9A)	96.6(5)
C(9)-C(8)-H(8A)	110.3
C(7)-C(8)-H(8A)	110.3
C(9)-C(8)-H(8B)	110.3
C(7)-C(8)-H(8B)	110.3
H(8A)-C(8)-H(8B)	108.6

C(7)-C(8)-H(8C)	112.4
C(9A)-C(8)-H(8C)	112.4
C(7)-C(8)-H(8D)	112.4
C(9A)-C(8)-H(8D)	112.4
H(8C)-C(8)-H(8D)	110.0
C(8)-C(9)-C(10)	106.06(18)
C(8)-C(9)-H(9A)	110.5
C(10)-C(9)-H(9A)	110.5
C(8)-C(9)-H(9B)	110.5
C(10)-C(9)-H(9B)	110.5
H(9A)-C(9)-H(9B)	108.7
C(10)-C(9A)-C(8)	103.9(6)
C(10)-C(9A)-H(9A1)	111.0
C(8)-C(9A)-H(9A1)	111.0
C(10)-C(9A)-H(9A2)	111.0
C(8)-C(9A)-H(9A2)	111.0
H(9A1)-C(9A)-H(9A2)	109.0
C(9A)-C(10)-C(6)	110.1(4)
C(6)-C(10)-C(9)	103.98(16)
C(6)-C(10)-H(10A)	111.0
C(9)-C(10)-H(10A)	111.0
C(6)-C(10)-H(10B)	111.0
C(9)-C(10)-H(10B)	111.0
H(10A)-C(10)-H(10B)	109.0
C(9A)-C(10)-H(10C)	109.6
C(6)-C(10)-H(10C)	109.6
C(9A)-C(10)-H(10D)	109.6
C(6)-C(10)-H(10D)	109.6
H(10C)-C(10)-H(10D)	108.1

Symmetry transformations used to generate equivalent atoms:

Table S10. Anisotropic displacement parameters ($Å^2$x 10^3) for V25125. The anisotropic displacement factor exponent takes the form: $-2p^2[h^2 a^{*2}U^{11} + ... + 2 h k a^* b^* U^{12}]$

	U^{11}	U^{22}	U^{33}	U^{23}	U^{13}	U^{12}
C(1)	13(1)	24(1)	21(1)	2(1)	-4(1)	2(1)
O(1)	27(1)	35(1)	24(1)	-3(1)	-5(1)	-9(1)
C(2)	17(1)	30(1)	24(1)	4(1)	0(1)	-3(1)
C(3)	22(1)	25(1)	19(1)	4(1)	-1(1)	1(1)
C(4)	23(1)	28(1)	16(1)	-1(1)	-3(1)	-2(1)
C(5)	17(1)	23(1)	17(1)	-1(1)	-3(1)	-1(1)
C(6)	15(1)	18(1)	17(1)	0(1)	-1(1)	3(1)
C(7)	18(1)	18(1)	16(1)	2(1)	-2(1)	2(1)
C(11)	20(1)	21(1)	17(1)	2(1)	0(1)	4(1)
C(12)	20(1)	18(1)	20(1)	-1(1)	0(1)	-3(1)
O(2)	29(1)	28(1)	19(1)	2(1)	-2(1)	3(1)
C(13)	27(1)	26(1)	25(1)	-2(1)	6(1)	4(1)
C(8)	41(1)	18(1)	23(1)	2(1)	5(1)	0(1)
C(9)	38(2)	20(1)	24(1)	0(1)	1(1)	5(1)
C(9A)	57(7)	20(4)	30(5)	1(4)	16(5)	16(4)
C(10)	27(1)	24(1)	27(1)	0(1)	2(1)	10(1)

Table S11. Hydrogen coordinates (x 10^4) and isotropic displacement parameters ($Å^2$x 10^3) for V25125.

	x	y	z	U(eq)
H(2A)	9660	6101	986	28
H(2B)	9544	7255	1216	28
H(3A)	5815	7327	714	27
H(3B)	7266	6850	-110	27
H(4A)	6133	5253	339	26
H(4B)	3982	5914	138	26
H(5A)	3547	5016	1557	23
H(5B)	3449	6176	1750	23
H(7)	6747	5493	3725	21
H(11A)	4093	6793	3393	23
H(11B)	2265	5950	3276	23
H(13A)	-27	6456	4890	39
H(13B)	1385	7405	4594	39
H(13C)	1612	6964	5628	39
H(8A)	5011	4055	3987	33
H(8B)	3161	4246	3198	33
H(8C)	3423	4174	2968	33
H(8D)	4518	4093	4001	33
H(9A)	5196	3417	2186	33
H(9B)	7092	3264	2964	33
H(9A1)	8248	3806	3355	43
H(9A2)	6774	3187	2621	43
H(10A)	9109	4587	2440	31
H(10B)	7799	4394	1474	31
H(10C)	7259	4262	1529	31
H(10D)	9237	4682	2163	31

Table S12. Torsion angles [°] for V25125.

O(1)-C(1)-C(2)-C(3)	-127.07(14)
C(6)-C(1)-C(2)-C(3)	53.10(16)
C(1)-C(2)-C(3)-C(4)	-55.29(16)
C(2)-C(3)-C(4)-C(5)	57.61(16)
C(3)-C(4)-C(5)-C(6)	-56.67(15)
O(1)-C(1)-C(6)-C(7)	4.99(18)
C(2)-C(1)-C(6)-C(7)	-175.18(11)
O(1)-C(1)-C(6)-C(5)	130.69(14)
C(2)-C(1)-C(6)-C(5)	-49.49(14)
O(1)-C(1)-C(6)-C(10)	-107.04(15)
C(2)-C(1)-C(6)-C(10)	72.78(14)
C(4)-C(5)-C(6)-C(1)	50.52(15)
C(4)-C(5)-C(6)-C(7)	175.86(11)
C(4)-C(5)-C(6)-C(10)	-71.02(15)
C(1)-C(6)-C(7)-C(11)	75.92(14)
C(5)-C(6)-C(7)-C(11)	-47.59(16)
C(10)-C(6)-C(7)-C(11)	-166.68(12)
C(1)-C(6)-C(7)-C(8)	-157.90(11)
C(5)-C(6)-C(7)-C(8)	78.59(14)
C(10)-C(6)-C(7)-C(8)	-40.50(13)
C(8)-C(7)-C(11)-C(12)	72.66(16)
C(6)-C(7)-C(11)-C(12)	-166.28(11)
C(7)-C(11)-C(12)-O(2)	3.6(2)
C(7)-C(11)-C(12)-C(13)	-176.48(12)
C(11)-C(7)-C(8)-C(9)	154.3(2)
C(6)-C(7)-C(8)-C(9)	26.9(2)
C(11)-C(7)-C(8)-C(9A)	175.4(7)
C(6)-C(7)-C(8)-C(9A)	48.0(7)
C(7)-C(8)-C(9)-C(10)	-2.2(3)
C(7)-C(8)-C(9A)-C(10)	-38.5(12)
C(8)-C(9A)-C(10)-C(6)	15.4(14)
C(1)-C(6)-C(10)-C(9A)	133.0(10)
C(7)-C(6)-C(10)-C(9A)	14.0(10)C(5)-C(6)-C(10)-C(9A)
	-106.2(10)

C(1)-C(6)-C(10)-C(9)	157.9(2)
C(7)-C(6)-C(10)-C(9)	39.0(2)
C(5)-C(6)-C(10)-C(9)	-81.2(2)
C(8)-C(9)-C(10)-C(6)	-23.1(3)

Symmetry transformations used to generate equivalent atoms:

Crystal Structure Analysis of Product 12a (sample No.: V24254)

Spirocycle **12a** was crystalized by layering pentane onto a saturated solution of **12a** in benzene at 23 °C to provide crystalline blocks suitable for X-ray analysis.

Compound V24254 (CCDC 2421483) crystallizes in the orthorhombic space group $P2_12_12_1$ with one molecule in the asymmetric unit. Absolute configuration was assigned via Flack (Flack = 0.01(9)) parameter.

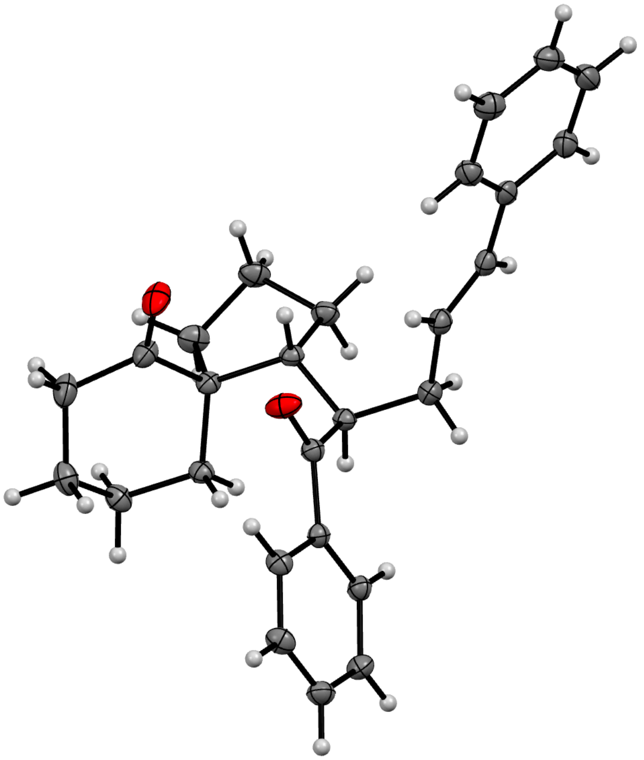

Table S13. Crystal data and structure refinement for V24254.

Identification code	V24254	
Empirical formula	C27 H30 O2	
Formula weight	386.51	
Temperature	100(2) K	
Wavelength	1.54178 Å	
Crystal system	Orthorhombic	
Space group	$P2_12_12_1$	
Unit cell dimensions	a = 8.3129(6) Å	a= 90°.
	b = 14.2383(15) Å	b= 90°.
	c = 17.763(2) Å	g = 90°.
Volume	2102.4(4) $Å^3$	

Z	4
Density (calculated)	1.221 Mg/m^3
Absorption coefficient	0.581 mm^{-1}
F(000)	832
Crystal size	0.200 x 0.100 x 0.100 mm^3
Theta range for data collection	3.979 to 74.369°.
Index ranges	-10<=h<=10, -17<=k<=17, -22<=l<=22
Reflections collected	37667
Independent reflections	4303 [R(int) = 0.0447]
Completeness to theta = 67.679°	100.0 %
Absorption correction	Semi-empirical from equivalents
Max. and min. transmission	0.7538 and 0.6890
Refinement method	Full-matrix least-squares on F^2
Data / restraints / parameters	4303 / 0 / 262
Goodness-of-fit on F^2	1.070
Final R indices [I>2sigma(I)]	R1 = 0.0292, wR2 = 0.0703
R indices (all data)	R1 = 0.0312, wR2 = 0.0720
Absolute structure parameter	0.01(9)
Extinction coefficient	n/a
Largest diff. peak and hole	0.186 and -0.160 e.Å^{-3}

Table S14. Atomic coordinates (x 10^4) and equivalent isotropic displacement parameters (Å^{2}x 10^3) for V24254. U(eq) is defined as one third of the trace of the orthogonalized U^{ij} tensor.

	x	y	z	U(eq)
O(1)	1331(2)	2536(1)	1678(1)	34(1)
C(1)	569(2)	3260(1)	1732(1)	22(1)
C(2)	239(2)	3847(1)	1036(1)	29(1)
C(3)	229(2)	4904(1)	1177(1)	26(1)
C(4)	-868(2)	5135(1)	1834(1)	22(1)
C(5)	-240(2)	4649(1)	2541(1)	19(1)
C(6)	-206(2)	3565(1)	2473(1)	17(1)
C(7)	-1973(2)	3176(1)	2445(1)	25(1)
C(8)	-1971(2)	2304(1)	2944(1)	25(1)
C(9)	-841(2)	2593(1)	3579(1)	22(1)

C(10)	576(2)	3053(1)	3166(1)	17(1)
C(11)	1633(2)	3674(1)	3668(1)	17(1)
C(21)	3139(2)	3996(1)	3245(1)	18(1)
O(2)	3831(2)	3457(1)	2822(1)	28(1)
C(22)	3782(2)	4969(1)	3365(1)	17(1)
C(23)	5022(2)	5272(1)	2890(1)	20(1)
C(24)	5640(2)	6173(1)	2954(1)	23(1)
C(25)	5022(2)	6783(1)	3491(1)	23(1)
C(26)	3798(2)	6488(1)	3965(1)	22(1)
C(27)	3173(2)	5584(1)	3905(1)	19(1)
C(12)	2171(2)	3183(1)	4407(1)	21(1)
C(13)	3016(2)	2259(1)	4312(1)	21(1)
C(14)	2624(2)	1501(1)	4713(1)	21(1)
C(15)	3431(2)	580(1)	4725(1)	21(1)
C(16)	2981(2)	-72(1)	5277(1)	24(1)
C(17)	3707(2)	-955(1)	5314(1)	28(1)
C(18)	4869(2)	-1200(1)	4792(1)	27(1)
C(19)	5315(2)	-564(1)	4235(1)	27(1)
C(20)	4617(2)	320(1)	4208(1)	23(1)

Table S15. Bond lengths [Å] and angles [°] for V24254.

O(1)-C(1)	1.215(2)
C(1)-C(2)	1.517(2)
C(1)-C(6)	1.528(2)
C(2)-C(3)	1.526(3)
C(2)-H(2A)	0.9900
C(2)-H(2B)	0.9900
C(3)-C(4)	1.517(2)
C(3)-H(3A)	0.9900
C(3)-H(3B)	0.9900
C(4)-C(5)	1.526(2)
C(4)-H(4A)	0.9900
C(4)-H(4B)	0.9900
C(5)-C(6)	1.549(2)

C(5)-H(5A)	0.9900
C(5)-H(5B)	0.9900
C(6)-C(10)	1.571(2)
C(6)-C(7)	1.571(2)
C(7)-C(8)	1.525(2)
C(7)-H(7A)	0.9900
C(7)-H(7B)	0.9900
C(8)-C(9)	1.523(2)
C(8)-H(8A)	0.9900
C(8)-H(8B)	0.9900
C(9)-C(10)	1.534(2)
C(9)-H(9A)	0.9900
C(9)-H(9B)	0.9900
C(10)-C(11)	1.532(2)
C(10)-H(10)	1.0000
C(11)-C(21)	1.530(2)
C(11)-C(12)	1.553(2)
C(11)-H(11)	1.0000
C(21)-O(2)	1.218(2)
C(21)-C(22)	1.501(2)
C(22)-C(27)	1.395(2)
C(22)-C(23)	1.400(2)
C(23)-C(24)	1.387(2)
C(23)-H(23)	0.9500
C(24)-C(25)	1.388(2)
C(24)-H(24)	0.9500
C(25)-C(26)	1.386(3)
C(25)-H(25)	0.9500
C(26)-C(27)	1.392(2)
C(26)-H(26)	0.9500
C(27)-H(27)	0.9500
C(12)-C(13)	1.501(2)
C(12)-H(12A)	0.9900
C(12)-H(12B)	0.9900
C(13)-C(14)	1.334(2)
C(13)-H(13)	0.9500

C(14)-C(15)	1.473(2)
C(14)-H(14)	0.9500
C(15)-C(20)	1.398(3)
C(15)-C(16)	1.401(2)
C(16)-C(17)	1.395(3)
C(16)-H(16)	0.9500
C(17)-C(18)	1.384(3)
C(17)-H(17)	0.9500
C(18)-C(19)	1.392(3)
C(18)-H(18)	0.9500
C(19)-C(20)	1.386(2)
C(19)-H(19)	0.9500
C(20)-H(20)	0.9500
O(1)-C(1)-C(2)	119.87(15)
O(1)-C(1)-C(6)	121.90(16)
C(2)-C(1)-C(6)	118.01(14)
C(1)-C(2)-C(3)	114.22(15)
C(1)-C(2)-H(2A)	108.7
C(3)-C(2)-H(2A)	108.7
C(1)-C(2)-H(2B)	108.7
C(3)-C(2)-H(2B)	108.7
H(2A)-C(2)-H(2B)	107.6
C(4)-C(3)-C(2)	110.05(15)
C(4)-C(3)-H(3A)	109.7
C(2)-C(3)-H(3A)	109.7
C(4)-C(3)-H(3B)	109.7
C(2)-C(3)-H(3B)	109.7
H(3A)-C(3)-H(3B)	108.2
C(3)-C(4)-C(5)	109.24(13)
C(3)-C(4)-H(4A)	109.8
C(5)-C(4)-H(4A)	109.8
C(3)-C(4)-H(4B)	109.8
C(5)-C(4)-H(4B)	109.8
H(4A)-C(4)-H(4B)	108.3
C(4)-C(5)-C(6)	113.17(14)

C(4)-C(5)-H(5A)	108.9
C(6)-C(5)-H(5A)	108.9
C(4)-C(5)-H(5B)	108.9
C(6)-C(5)-H(5B)	108.9
H(5A)-C(5)-H(5B)	107.8
C(1)-C(6)-C(5)	110.95(14)
C(1)-C(6)-C(10)	111.66(13)
C(5)-C(6)-C(10)	114.13(13)
C(1)-C(6)-C(7)	105.48(13)
C(5)-C(6)-C(7)	109.67(13)
C(10)-C(6)-C(7)	104.38(13)
C(8)-C(7)-C(6)	105.49(13)
C(8)-C(7)-H(7A)	110.6
C(6)-C(7)-H(7A)	110.6
C(8)-C(7)-H(7B)	110.6
C(6)-C(7)-H(7B)	110.6
H(7A)-C(7)-H(7B)	108.8
C(9)-C(8)-C(7)	102.20(14)
C(9)-C(8)-H(8A)	111.3
C(7)-C(8)-H(8A)	111.3
C(9)-C(8)-H(8B)	111.3
C(7)-C(8)-H(8B)	111.3
H(8A)-C(8)-H(8B)	109.2
C(8)-C(9)-C(10)	103.60(13)
C(8)-C(9)-H(9A)	111.0
C(10)-C(9)-H(9A)	111.0
C(8)-C(9)-H(9B)	111.0
C(10)-C(9)-H(9B)	111.0
H(9A)-C(9)-H(9B)	109.0
C(11)-C(10)-C(9)	114.13(13)
C(11)-C(10)-C(6)	115.17(12)
C(9)-C(10)-C(6)	104.78(13)
C(11)-C(10)-H(10)	107.5
C(9)-C(10)-H(10)	107.5
C(6)-C(10)-H(10)	107.5
C(21)-C(11)-C(10)	110.92(13)

C(21)-C(11)-C(12)	108.29(13)
C(10)-C(11)-C(12)	113.40(13)
C(21)-C(11)-H(11)	108.0
C(10)-C(11)-H(11)	108.0
C(12)-C(11)-H(11)	108.0
O(2)-C(21)-C(22)	120.00(15)
O(2)-C(21)-C(11)	120.07(14)
C(22)-C(21)-C(11)	119.93(13)
C(27)-C(22)-C(23)	119.22(14)
C(27)-C(22)-C(21)	123.25(15)
C(23)-C(22)-C(21)	117.50(14)
C(24)-C(23)-C(22)	120.56(15)
C(24)-C(23)-H(23)	119.7
C(22)-C(23)-H(23)	119.7
C(23)-C(24)-C(25)	119.86(16)
C(23)-C(24)-H(24)	120.1
C(25)-C(24)-H(24)	120.1
C(26)-C(25)-C(24)	119.99(16)
C(26)-C(25)-H(25)	120.0
C(24)-C(25)-H(25)	120.0
C(25)-C(26)-C(27)	120.53(15)
C(25)-C(26)-H(26)	119.7
C(27)-C(26)-H(26)	119.7
C(26)-C(27)-C(22)	119.85(15)
C(26)-C(27)-H(27)	120.1
C(22)-C(27)-H(27)	120.1
C(13)-C(12)-C(11)	115.78(13)
C(13)-C(12)-H(12A)	108.3
C(11)-C(12)-H(12A)	108.3
C(13)-C(12)-H(12B)	108.3
C(11)-C(12)-H(12B)	108.3
H(12A)-C(12)-H(12B)	107.4
C(14)-C(13)-C(12)	122.37(16)
C(14)-C(13)-H(13)	118.8
C(12)-C(13)-H(13)	118.8
C(13)-C(14)-C(15)	128.06(16)

C(13)-C(14)-H(14)	116.0
C(15)-C(14)-H(14)	116.0
C(20)-C(15)-C(16)	118.20(16)
C(20)-C(15)-C(14)	123.24(15)
C(16)-C(15)-C(14)	118.56(16)
C(17)-C(16)-C(15)	120.98(17)
C(17)-C(16)-H(16)	119.5
C(15)-C(16)-H(16)	119.5
C(18)-C(17)-C(16)	119.82(17)
C(18)-C(17)-H(17)	120.1
C(16)-C(17)-H(17)	120.1
C(17)-C(18)-C(19)	119.87(17)
C(17)-C(18)-H(18)	120.1
C(19)-C(18)-H(18)	120.1
C(20)-C(19)-C(18)	120.29(18)
C(20)-C(19)-H(19)	119.9
C(18)-C(19)-H(19)	119.9
C(19)-C(20)-C(15)	120.83(16)
C(19)-C(20)-H(20)	119.6
C(15)-C(20)-H(20)	119.6

Symmetry transformations used to generate equivalent atoms:

Table S16. Anisotropic displacement parameters ($Å^2$x 10^3) for V24254. The anisotropic displacement factor exponent takes the form: -2p^2[h^2 a*^{2}U^{11} + ... + 2 h k a* b* U^{12}]

	U^{11}	U^{22}	U^{33}	U^{23}	U^{13}	U^{12}
O(1)	43(1)	35(1)	25(1)	-10(1)	-5(1)	20(1)
C(1)	21(1)	25(1)	21(1)	-6(1)	-4(1)	4(1)
C(2)	33(1)	37(1)	17(1)	-3(1)	-3(1)	7(1)
C(3)	22(1)	33(1)	23(1)	6(1)	-1(1)	5(1)
C(4)	18(1)	23(1)	26(1)	4(1)	-1(1)	3(1)
C(5)	19(1)	16(1)	20(1)	-1(1)	-1(1)	3(1)
C(6)	15(1)	17(1)	19(1)	-2(1)	-1(1)	3(1)
C(7)	17(1)	24(1)	35(1)	0(1)	-4(1)	-1(1)

C(8)	21(1)	22(1)	33(1)	1(1)	-2(1)	-4(1)
C(9)	23(1)	20(1)	24(1)	0(1)	2(1)	-3(1)
C(10)	17(1)	14(1)	19(1)	-1(1)	-1(1)	2(1)
C(11)	18(1)	14(1)	18(1)	0(1)	-1(1)	1(1)
C(21)	17(1)	17(1)	19(1)	0(1)	-2(1)	1(1)
O(2)	26(1)	23(1)	35(1)	-9(1)	10(1)	-2(1)
C(22)	15(1)	18(1)	18(1)	0(1)	-3(1)	1(1)
C(23)	17(1)	20(1)	22(1)	1(1)	2(1)	3(1)
C(24)	19(1)	23(1)	27(1)	3(1)	3(1)	-2(1)
C(25)	24(1)	18(1)	27(1)	0(1)	-3(1)	-2(1)
C(26)	26(1)	20(1)	21(1)	-4(1)	-1(1)	0(1)
C(27)	21(1)	20(1)	17(1)	-1(1)	2(1)	-2(1)
C(12)	25(1)	19(1)	19(1)	-1(1)	-2(1)	-1(1)
C(13)	22(1)	22(1)	19(1)	-1(1)	-4(1)	1(1)
C(14)	22(1)	24(1)	16(1)	-2(1)	-4(1)	-1(1)
C(15)	22(1)	21(1)	19(1)	1(1)	-8(1)	-4(1)
C(16)	28(1)	26(1)	19(1)	2(1)	-4(1)	-2(1)
C(17)	35(1)	22(1)	27(1)	6(1)	-8(1)	-5(1)
C(18)	28(1)	19(1)	35(1)	0(1)	-11(1)	0(1)
C(19)	22(1)	25(1)	33(1)	-2(1)	-4(1)	-1(1)
C(20)	22(1)	22(1)	25(1)	2(1)	-2(1)	-4(1)

Table S17. Hydrogen coordinates (x 10^4) and isotropic displacement parameters (Å^{2}x 10^3) for V24254.

	x	y	z	U(eq)
H(2A)	-817	3661	826	35
H(2B)	1068	3703	653	35
H(3A)	1335	5121	1289	31
H(3B)	-151	5235	720	31
H(4A)	-1975	4917	1724	27
H(4B)	-898	5822	1913	27
H(5A)	-929	4826	2973	22

H(5B)	862	4877	2647	22
H(7A)	-2741	3649	2638	30
H(7B)	-2277	3011	1922	30
H(8A)	-3063	2164	3137	30
H(8B)	-1556	1749	2670	30
H(9A)	-482	2039	3871	26
H(9B)	-1369	3044	3923	26
H(10)	1270	2537	2965	20
H(11)	998	4245	3806	20
H(23)	5444	4856	2521	24
H(24)	6484	6373	2631	28
H(25)	5438	7402	3534	27
H(26)	3382	6906	4334	27
H(27)	2333	5387	4232	23
H(12A)	2895	3614	4683	25
H(12B)	1208	3082	4724	25
H(13)	3861	2212	3954	25
H(14)	1710	1562	5031	25
H(16)	2169	88	5631	29
H(17)	3404	-1386	5697	34
H(18)	5362	-1801	4814	33
H(19)	6100	-736	3872	32
H(20)	4949	754	3833	28

Table S18. Torsion angles [°] for V24254.

O(1)-C(1)-C(2)-C(3)	-145.22(18)
C(6)-C(1)-C(2)-C(3)	40.1(2)
C(1)-C(2)-C(3)-C(4)	-51.0(2)
C(2)-C(3)-C(4)-C(5)	61.25(18)
C(3)-C(4)-C(5)-C(6)	-61.33(18)
O(1)-C(1)-C(6)-C(5)	148.18(17)
C(2)-C(1)-C(6)-C(5)	-37.3(2)
O(1)-C(1)-C(6)-C(10)	19.6(2)
C(2)-C(1)-C(6)-C(10)	-165.80(15)

O(1)-C(1)-C(6)-C(7)	-93.14(19)
C(2)-C(1)-C(6)-C(7)	81.42(18)
C(4)-C(5)-C(6)-C(1)	47.77(18)
C(4)-C(5)-C(6)-C(10)	174.96(13)
C(4)-C(5)-C(6)-C(7)	-68.35(18)
C(1)-C(6)-C(7)-C(8)	102.34(16)
C(5)-C(6)-C(7)-C(8)	-138.12(15)
C(10)-C(6)-C(7)-C(8)	-15.46(17)
C(6)-C(7)-C(8)-C(9)	37.04(17)
C(7)-C(8)-C(9)-C(10)	-44.80(17)
C(8)-C(9)-C(10)-C(11)	161.94(13)
C(8)-C(9)-C(10)-C(6)	35.06(16)
C(1)-C(6)-C(10)-C(11)	108.41(15)
C(5)-C(6)-C(10)-C(11)	-18.42(19)
C(7)-C(6)-C(10)-C(11)	-138.12(14)
C(1)-C(6)-C(10)-C(9)	-125.36(14)
C(5)-C(6)-C(10)-C(9)	107.81(15)
C(7)-C(6)-C(10)-C(9)	-11.89(15)
C(9)-C(10)-C(11)-C(21)	172.19(13)
C(6)-C(10)-C(11)-C(21)	-66.53(17)
C(9)-C(10)-C(11)-C(12)	50.08(18)
C(6)-C(10)-C(11)-C(12)	171.36(13)
C(10)-C(11)-C(21)-O(2)	-38.8(2)
C(12)-C(11)-C(21)-O(2)	86.24(18)
C(10)-C(11)-C(21)-C(22)	142.37(13)
C(12)-C(11)-C(21)-C(22)	-92.58(16)
O(2)-C(21)-C(22)-C(27)	-172.12(16)
C(11)-C(21)-C(22)-C(27)	6.7(2)
O(2)-C(21)-C(22)-C(23)	9.8(2)
C(11)-C(21)-C(22)-C(23)	-171.36(14)
C(27)-C(22)-C(23)-C(24)	-0.1(2)
C(21)-C(22)-C(23)-C(24)	178.06(15)
C(22)-C(23)-C(24)-C(25)	-0.2(2)
C(23)-C(24)-C(25)-C(26)	0.4(3)
C(24)-C(25)-C(26)-C(27)	-0.3(3)
C(25)-C(26)-C(27)-C(22)	0.0(3)

C(23)-C(22)-C(27)-C(26)	0.2(2)
C(21)-C(22)-C(27)-C(26)	-177.86(15)
C(21)-C(11)-C(12)-C(13)	-68.62(17)
C(10)-C(11)-C(12)-C(13)	54.95(19)
C(11)-C(12)-C(13)-C(14)	-132.36(17)
C(12)-C(13)-C(14)-C(15)	-174.46(15)
C(13)-C(14)-C(15)-C(20)	-10.8(3)
C(13)-C(14)-C(15)-C(16)	169.78(17)
C(20)-C(15)-C(16)-C(17)	0.6(2)
C(14)-C(15)-C(16)-C(17)	-179.92(16)
C(15)-C(16)-C(17)-C(18)	-1.2(3)
C(16)-C(17)-C(18)-C(19)	0.4(3)
C(17)-C(18)-C(19)-C(20)	1.0(3)
C(18)-C(19)-C(20)-C(15)	-1.6(3)
C(16)-C(15)-C(20)-C(19)	0.8(2)
C(14)-C(15)-C(20)-C(19)	-178.69(16)

Symmetry transformations used to generate equivalent atoms:

Crystal Structure Analysis of Product 16 (sample No.: V24156)

Pinacol **16** was crystalized by layering pentane onto a saturated solution of **16** in diethyl ether at 23 °C to provide crystalline blocks suitable for X-ray analysis.

Compound V25146 (CCDC 2444208) crystallizes in the orthorhombic space group $P2_12_12_1$ with one molecule in the asymmetric unit. The coordinates for the hydrogen atoms bound to O1 and O2 were located in the difference Fourier synthesis and refined semi-freely with the help of a restraint on the O-H distance (0.84(4) Å). Absolute configuration was assigned via Flack (Flack = 0.08(5)) parameter.

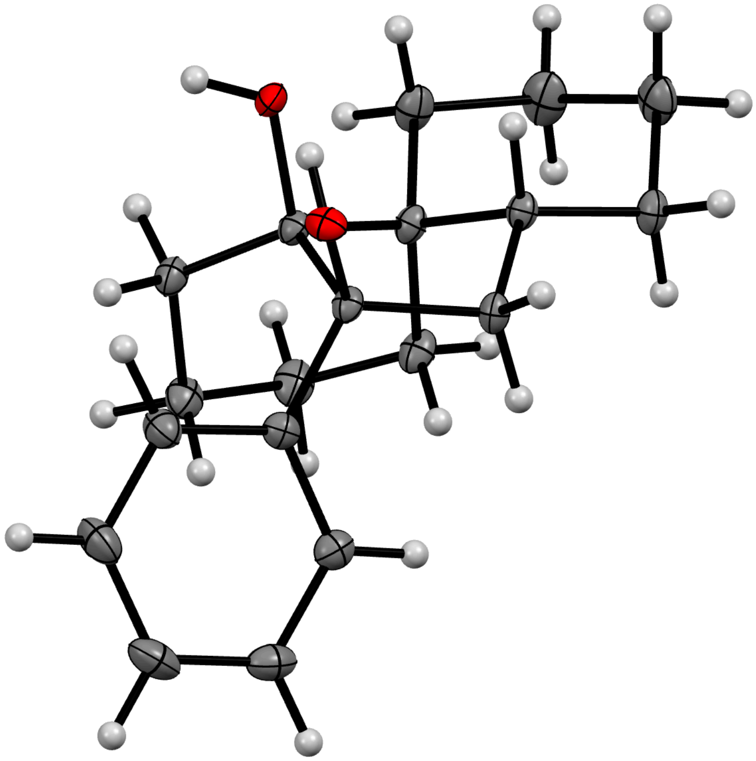

Table S19. Crystal data and structure refinement for V25146.

Identification code	V25146	
Empirical formula	C19 H26 O2	
Formula weight	286.40	
Temperature	100(2) K	
Wavelength	1.54178 Å	
Crystal system	Orthorhombic	
Space group	$P2_12_12_1$	
Unit cell dimensions	a = 7.4117(6) Å	a= 90°.
	b = 8.4258(9) Å	b= 90°.

	c = 24.689(3) Å	g = 90°.
Volume	1541.8(3) Å^3	
Z	4	
Density (calculated)	1.234 Mg/m^3	
Absorption coefficient	0.605 mm^{-1}	
F(000)	624	
Crystal size	0.200 x 0.200 x 0.150 mm^3	
Theta range for data collection	3.580 to 74.555°.	
Index ranges	-9<=h<=9, -10<=k<=9, -30<=l<=30	
Reflections collected	24262	
Independent reflections	3148 [R(int) = 0.0479]	
Completeness to theta = 67.679°	100.0 %	
Absorption correction	Semi-empirical from equivalents	
Max. and min. transmission	0.7538 and 0.6822	
Refinement method	Full-matrix least-squares on F^2	
Data / restraints / parameters	3148 / 0 / 196	
Goodness-of-fit on F^2	1.065	
Final R indices [I>2sigma(I)]	R1 = 0.0280, wR2 = 0.0694	
R indices (all data)	R1 = 0.0287, wR2 = 0.0697	
Absolute structure parameter	0.08(5)	
Extinction coefficient	n/a	
Largest diff. peak and hole	0.174 and -0.205 e.Å^{-3}	

Table S20. Atomic coordinates (x 10^4) and equivalent isotropic displacement parameters (Å^{2}x 10^3) for V25146. U(eq) is defined as one third of the trace of the orthogonalized U^{ij} tensor.

	x	y	z	U(eq)
C(1)	4966(2)	6727(2)	5938(1)	13(1)
O(1)	3972(2)	7531(1)	5518(1)	15(1)
C(14)	4440(2)	4984(2)	5896(1)	14(1)
C(15)	4248(2)	4297(2)	5384(1)	19(1)
C(16)	3711(3)	2728(2)	5324(1)	25(1)
C(17)	3370(2)	1795(2)	5776(1)	25(1)
C(18)	3584(2)	2450(2)	6287(1)	22(1)

C(19)	4110(2)	4024(2)	6347(1)	17(1)
C(2)	7108(2)	7110(2)	5839(1)	13(1)
O(2)	7142(1)	8365(1)	5444(1)	15(1)
C(3)	8314(2)	5767(2)	5630(1)	16(1)
C(4)	8823(2)	4490(2)	6045(1)	19(1)
C(5)	9566(2)	5270(2)	6556(1)	20(1)
C(6)	8202(2)	6446(2)	6786(1)	17(1)
C(7)	7736(2)	7816(2)	6390(1)	14(1)
C(8)	9299(2)	9008(2)	6348(1)	20(1)
C(9)	9585(2)	9887(2)	6890(1)	26(1)
C(10)	7849(2)	10662(2)	7102(1)	24(1)
C(11)	6260(2)	9498(2)	7125(1)	17(1)
C(12)	6040(2)	8733(2)	6569(1)	14(1)
C(13)	4512(2)	7547(2)	6484(1)	14(1)

Table S21. Bond lengths [Å] and angles [°] for V25146.

C(1)-O(1)	1.4407(16)
C(1)-C(14)	1.5230(19)
C(1)-C(13)	1.5524(18)
C(1)-C(2)	1.638(2)
O(1)-H(1O)	0.79(2)
C(14)-C(19)	1.397(2)
C(14)-C(15)	1.398(2)
C(15)-C(16)	1.389(2)
C(15)-H(15)	0.9500
C(16)-C(17)	1.390(2)
C(16)-H(16)	0.9500
C(17)-C(18)	1.385(2)
C(17)-H(17)	0.9500
C(18)-C(19)	1.390(2)
C(18)-H(18)	0.9500
C(19)-H(19)	0.9500
C(2)-O(2)	1.4386(16)
C(2)-C(3)	1.5310(19)
C(2)-C(7)	1.5563(18)
O(2)-H(2O)	0.83(2)
C(3)-C(4)	1.5327(19)
C(3)-H(3A)	0.9900
C(3)-H(3B)	0.9900
C(4)-C(5)	1.526(2)
C(4)-H(4A)	0.9900
C(4)-H(4B)	0.9900
C(5)-C(6)	1.525(2)
C(5)-H(5A)	0.9900
C(5)-H(5B)	0.9900
C(6)-C(7)	1.5515(19)
C(6)-H(6A)	0.9900
C(6)-H(6B)	0.9900
C(7)-C(8)	1.536(2)
C(7)-C(12)	1.5399(19)

C(8)-C(9)	1.544(2)
C(8)-H(8A)	0.9900
C(8)-H(8B)	0.9900
C(9)-C(10)	1.536(2)
C(9)-H(9A)	0.9900
C(9)-H(9B)	0.9900
C(10)-C(11)	1.534(2)
C(10)-H(10A)	0.9900
C(10)-H(10B)	0.9900
C(11)-C(12)	1.5251(18)
C(11)-H(11A)	0.9900
C(11)-H(11B)	0.9900
C(12)-C(13)	1.525(2)
C(12)-H(12)	1.0000
C(13)-H(13A)	0.9900
C(13)-H(13B)	0.9900

O(1)-C(1)-C(14)	105.93(11)
O(1)-C(1)-C(13)	107.70(11)
C(14)-C(1)-C(13)	115.59(12)
O(1)-C(1)-C(2)	107.26(10)
C(14)-C(1)-C(2)	115.37(11)
C(13)-C(1)-C(2)	104.55(11)
C(1)-O(1)-H(1O)	105.5(15)
C(19)-C(14)-C(15)	117.56(13)
C(19)-C(14)-C(1)	123.36(13)
C(15)-C(14)-C(1)	119.07(13)
C(16)-C(15)-C(14)	121.37(14)
C(16)-C(15)-H(15)	119.3
C(14)-C(15)-H(15)	119.3
C(15)-C(16)-C(17)	120.28(15)
C(15)-C(16)-H(16)	119.9
C(17)-C(16)-H(16)	119.9
C(18)-C(17)-C(16)	119.07(14)
C(18)-C(17)-H(17)	120.5
C(16)-C(17)-H(17)	120.5

C(17)-C(18)-C(19)	120.59(15)
C(17)-C(18)-H(18)	119.7
C(19)-C(18)-H(18)	119.7
C(18)-C(19)-C(14)	121.12(14)
C(18)-C(19)-H(19)	119.4
C(14)-C(19)-H(19)	119.4
O(2)-C(2)-C(3)	107.76(11)
O(2)-C(2)-C(7)	107.79(11)
C(3)-C(2)-C(7)	113.73(12)
O(2)-C(2)-C(1)	105.19(10)
C(3)-C(2)-C(1)	118.05(12)
C(7)-C(2)-C(1)	103.64(10)
C(2)-O(2)-H(2O)	111.4(14)
C(2)-C(3)-C(4)	115.94(12)
C(2)-C(3)-H(3A)	108.3
C(4)-C(3)-H(3A)	108.3
C(2)-C(3)-H(3B)	108.3
C(4)-C(3)-H(3B)	108.3
H(3A)-C(3)-H(3B)	107.4
C(5)-C(4)-C(3)	109.78(12)
C(5)-C(4)-H(4A)	109.7
C(3)-C(4)-H(4A)	109.7
C(5)-C(4)-H(4B)	109.7
C(3)-C(4)-H(4B)	109.7
H(4A)-C(4)-H(4B)	108.2
C(6)-C(5)-C(4)	110.39(12)
C(6)-C(5)-H(5A)	109.6
C(4)-C(5)-H(5A)	109.6
C(6)-C(5)-H(5B)	109.6
C(4)-C(5)-H(5B)	109.6
H(5A)-C(5)-H(5B)	108.1
C(5)-C(6)-C(7)	113.36(12)
C(5)-C(6)-H(6A)	108.9
C(7)-C(6)-H(6A)	108.9
C(5)-C(6)-H(6B)	108.9
C(7)-C(6)-H(6B)	108.9

H(6A)-C(6)-H(6B)	107.7
C(8)-C(7)-C(12)	107.88(12)
C(8)-C(7)-C(6)	111.20(11)
C(12)-C(7)-C(6)	111.98(11)
C(8)-C(7)-C(2)	114.59(11)
C(12)-C(7)-C(2)	101.44(11)
C(6)-C(7)-C(2)	109.39(11)
C(7)-C(8)-C(9)	111.00(12)
C(7)-C(8)-H(8A)	109.4
C(9)-C(8)-H(8A)	109.4
C(7)-C(8)-H(8B)	109.4
C(9)-C(8)-H(8B)	109.4
H(8A)-C(8)-H(8B)	108.0
C(10)-C(9)-C(8)	112.63(13)
C(10)-C(9)-H(9A)	109.1
C(8)-C(9)-H(9A)	109.1
C(10)-C(9)-H(9B)	109.1
C(8)-C(9)-H(9B)	109.1
H(9A)-C(9)-H(9B)	107.8
C(11)-C(10)-C(9)	112.56(13)
C(11)-C(10)-H(10A)	109.1
C(9)-C(10)-H(10A)	109.1
C(11)-C(10)-H(10B)	109.1
C(9)-C(10)-H(10B)	109.1
H(10A)-C(10)-H(10B)	107.8
C(12)-C(11)-C(10)	108.62(12)
C(12)-C(11)-H(11A)	110.0
C(10)-C(11)-H(11A)	110.0
C(12)-C(11)-H(11B)	110.0
C(10)-C(11)-H(11B)	110.0
H(11A)-C(11)-H(11B)	108.3
C(11)-C(12)-C(13)	118.70(12)
C(11)-C(12)-C(7)	112.50(11)
C(13)-C(12)-C(7)	103.77(11)
C(11)-C(12)-H(12)	107.1
C(13)-C(12)-H(12)	107.1

C(7)-C(12)-H(12)	107.1
C(12)-C(13)-C(1)	104.50(11)
C(12)-C(13)-H(13A)	110.9
C(1)-C(13)-H(13A)	110.9
C(12)-C(13)-H(13B)	110.9
C(1)-C(13)-H(13B)	110.9
H(13A)-C(13)-H(13B)	108.9

Symmetry transformations used to generate equivalent atoms:

Table S22. Anisotropic displacement parameters ($Å^2$x 10^3) for V25146. The anisotropic displacement factor exponent takes the form: $-2p^2[h^2 a^{*2}U^{11} + ... + 2 h k a^* b^* U^{12}]$

	U^{11}	U^{22}	U^{33}	U^{23}	U^{13}	U^{12}
C(1)	13(1)	16(1)	10(1)	0(1)	-1(1)	2(1)
O(1)	17(1)	14(1)	15(1)	3(1)	-6(1)	0(1)
C(14)	11(1)	15(1)	16(1)	0(1)	-1(1)	1(1)
C(15)	24(1)	16(1)	18(1)	-1(1)	-3(1)	2(1)
C(16)	32(1)	17(1)	26(1)	-6(1)	-5(1)	1(1)
C(17)	24(1)	13(1)	39(1)	0(1)	-2(1)	-1(1)
C(18)	18(1)	19(1)	28(1)	8(1)	2(1)	1(1)
C(19)	14(1)	20(1)	18(1)	2(1)	0(1)	2(1)
C(2)	14(1)	15(1)	10(1)	1(1)	0(1)	1(1)
O(2)	19(1)	16(1)	10(1)	1(1)	4(1)	2(1)
C(3)	16(1)	19(1)	13(1)	0(1)	2(1)	3(1)
C(4)	19(1)	19(1)	19(1)	1(1)	1(1)	6(1)
C(5)	17(1)	25(1)	18(1)	2(1)	-2(1)	8(1)
C(6)	15(1)	24(1)	12(1)	2(1)	-2(1)	4(1)
C(7)	12(1)	19(1)	11(1)	0(1)	0(1)	1(1)
C(8)	14(1)	27(1)	18(1)	-4(1)	1(1)	-4(1)
C(9)	19(1)	35(1)	24(1)	-9(1)	-2(1)	-9(1)
C(10)	26(1)	27(1)	19(1)	-9(1)	-2(1)	-4(1)
C(11)	17(1)	22(1)	13(1)	-4(1)	0(1)	1(1)
C(12)	12(1)	17(1)	11(1)	-1(1)	-1(1)	1(1)
C(13)	12(1)	18(1)	12(1)	-3(1)	0(1)	1(1)

Table S23. Hydrogen coordinates (x 10^4) and isotropic displacement parameters ($Å^2$x 10^3) for V25146.

	x	y	z	U(eq)
H(1O)	4660(30)	8130(30)	5386(8)	18
H(15)	4490	4916	5071	23
H(16)	3576	2290	4971	30
H(17)	2995	723	5736	30
H(18)	3370	1817	6599	26
H(19)	4247	4454	6700	21
H(2O)	7670(30)	8090(20)	5164(8)	18
H(3A)	9440	6241	5487	19
H(3B)	7692	5246	5323	19
H(4A)	7746	3849	6136	23
H(4B)	9744	3773	5888	23
H(5A)	9834	4445	6830	24
H(5B)	10704	5831	6470	24
H(6A)	8692	6903	7125	21
H(6B)	7079	5869	6878	21
H(8A)	9033	9788	6059	24
H(8B)	10419	8439	6248	24
H(9A)	10038	9126	7163	31
H(9B)	10516	10717	6840	31
H(10A)	8072	11088	7470	28
H(10B)	7525	11564	6864	28
H(11A)	6493	8671	7401	21
H(11B)	5142	10068	7226	21
H(12)	5857	9612	6302	16
H(13A)	3333	8097	6464	17
H(13B)	4473	6763	6782	17

Table S24. Torsion angles [°] for V25146.

O(1)-C(1)-C(14)-C(19)	-138.81(14)
C(13)-C(1)-C(14)-C(19)	-19.65(19)
C(2)-C(1)-C(14)-C(19)	102.72(15)
O(1)-C(1)-C(14)-C(15)	40.32(17)
C(13)-C(1)-C(14)-C(15)	159.48(13)
C(2)-C(1)-C(14)-C(15)	-78.15(16)
C(19)-C(14)-C(15)-C(16)	1.4(2)
C(1)-C(14)-C(15)-C(16)	-177.79(14)
C(14)-C(15)-C(16)-C(17)	-0.7(3)
C(15)-C(16)-C(17)-C(18)	-0.4(3)
C(16)-C(17)-C(18)-C(19)	0.8(2)
C(17)-C(18)-C(19)-C(14)	-0.2(2)
C(15)-C(14)-C(19)-C(18)	-0.9(2)
C(1)-C(14)-C(19)-C(18)	178.20(13)
O(1)-C(1)-C(2)-O(2)	11.89(14)
C(14)-C(1)-C(2)-O(2)	129.62(11)
C(13)-C(1)-C(2)-O(2)	-102.29(12)
O(1)-C(1)-C(2)-C(3)	-108.30(13)
C(14)-C(1)-C(2)-C(3)	9.43(17)
C(13)-C(1)-C(2)-C(3)	137.53(12)
O(1)-C(1)-C(2)-C(7)	124.94(11)
C(14)-C(1)-C(2)-C(7)	-117.33(13)
C(13)-C(1)-C(2)-C(7)	10.76(13)
O(2)-C(2)-C(3)-C(4)	166.10(12)
C(7)-C(2)-C(3)-C(4)	46.68(17)
C(1)-C(2)-C(3)-C(4)	-75.06(16)
C(2)-C(3)-C(4)-C(5)	-50.84(17)
C(3)-C(4)-C(5)-C(6)	56.35(16)
C(4)-C(5)-C(6)-C(7)	-60.91(17)
C(5)-C(6)-C(7)-C(8)	-73.43(16)
C(5)-C(6)-C(7)-C(12)	165.79(12)
C(5)-C(6)-C(7)-C(2)	54.15(15)
O(2)-C(2)-C(7)-C(8)	-39.64(16)
C(3)-C(2)-C(7)-C(8)	79.77(16)

C(1)-C(2)-C(7)-C(8)	-150.80(11)
O(2)-C(2)-C(7)-C(12)	76.29(12)
C(3)-C(2)-C(7)-C(12)	-164.31(12)
C(1)-C(2)-C(7)-C(12)	-34.87(13)
O(2)-C(2)-C(7)-C(6)	-165.29(11)
C(3)-C(2)-C(7)-C(6)	-45.88(15)
C(1)-C(2)-C(7)-C(6)	83.55(13)
C(12)-C(7)-C(8)-C(9)	56.76(16)
C(6)-C(7)-C(8)-C(9)	-66.40(16)
C(2)-C(7)-C(8)-C(9)	168.90(13)
C(7)-C(8)-C(9)-C(10)	-53.29(19)
C(8)-C(9)-C(10)-C(11)	51.7(2)
C(9)-C(10)-C(11)-C(12)	-53.55(17)
C(10)-C(11)-C(12)-C(13)	-178.67(13)
C(10)-C(11)-C(12)-C(7)	59.97(16)
C(8)-C(7)-C(12)-C(11)	-62.00(15)
C(6)-C(7)-C(12)-C(11)	60.69(15)
C(2)-C(7)-C(12)-C(11)	177.24(11)
C(8)-C(7)-C(12)-C(13)	168.46(11)
C(6)-C(7)-C(12)-C(13)	-68.85(13)
C(2)-C(7)-C(12)-C(13)	47.70(13)
C(11)-C(12)-C(13)-C(1)	-166.74(12)
C(7)-C(12)-C(13)-C(1)	-41.06(13)
O(1)-C(1)-C(13)-C(12)	-95.90(13)
C(14)-C(1)-C(13)-C(12)	145.92(12)
C(2)-C(1)-C(13)-C(12)	17.96(13)

Symmetry transformations used to generate equivalent atoms:

Table S25. Hydrogen bonds for V25146 [Å and °].

D-H...A	d(D-H)	d(H...A)	d(D...A)	<(DHA)
O(1)-H(1O)...O(2)	0.79(2)	1.85(2)	2.4591(15)	132.6(19)
O(2)-H(2O)...O(1)#1	0.83(2)	2.01(2)	2.8384(14)	179(2)

Symmetry transformations used to generate equivalent atoms:
#1 x+1/2,-y+3/2,-z+1
